# Dynamic adaptation of myocardial proteome during heart failure development

**DOI:** 10.1371/journal.pone.0185915

**Published:** 2017-10-03

**Authors:** Julia Rüdebusch, Alexander Benkner, Axel Poesch, Marcus Dörr, Uwe Völker, Karina Grube, Elke Hammer, Stephan B. Felix

**Affiliations:** 1 Department of Internal Medicine B, University Medicine Greifswald, Greifswald, Germany; 2 DZHK (German Centre for Cardiovascular Research, partner site Greifswald), Greifswald, Germany; 3 Interfaculty Institute for Genetics and Functional Genomics, University Medicine Greifswald, Greifswald, Germany; Scuola Superiore Sant'Anna, ITALY

## Abstract

Heart failure (HF) development is characterized by huge structural changes that are crucial for disease progression. Analysis of time dependent global proteomic adaptations during HF progression offers the potential to gain deeper insights in the disease development and identify new biomarker candidates. Therefore, hearts of TAC (transverse aortic constriction) and sham mice were examined by cardiac MRI on either day 4, 14, 21, 28, 42, and 56 after surgery (n = 6 per group/time point). At each time point, proteomes of the left (LV) and right ventricles (RV) of TAC and sham mice were analyzed by mass spectrometry (MS). In TAC mice, systolic LV heart function worsened from day 4 to day 14, remained on a stable level from day 14 to day 42, and showed a further pronounced decline at day 56. MS analysis identified in the LV 330 and in RV 246 proteins with altered abundance over time (TAC vs. sham, fc≥±2). Functional categorization of proteins disclosed the time-dependent alteration of different pathways. Heat shock protein beta-7 (HSPB7) displayed differences in abundance in tissue and serum at an early stage of HF. This study not only provides an overview of the time dependent molecular alterations during transition to HF, but also identified HSPB7 as a novel blood biomarker candidate for the onset of cardiac remodeling.

## Introduction

Heart failure (HF) is a complex clinical syndrome resulting from structural and functional impairment of ventricular filling or ejection of blood. Coronary artery disease and arterial hypertension with sustained increase in left ventricular (LV) afterload are common causes of systolic HF. Disease initiation is characterized by subclinical myocardial hypertrophy, release of neurohumoral factors and activation of intracellular signaling pathways, but still preserved LV systolic function [1]. However, persisting severe hemodynamic overload induces left ventricular failure which is characterized by chamber dilation and continuous decrease in myocardial contractility. HF development is linked to several signaling cascades, but relatively little is known about the time dependent activation and inhibition of pathways and molecular events during initiation and progression of heart failure development [2–4]. To study the transition from hypertrophy to HF we used the highly reproducible mouse model of transverse aortic constriction (TAC) where a reduced lumen of the ascending aorta induces an increase of left ventricular afterload thereby simulating arterial hypertension as a common cause of HF development. In this model, we were able to examine the different disease stages from left ventricular hypertrophy to ventricular systolic dysfunction and heart failure. Since recent proteome profiling studies mainly focused on end-stage HF [5–7], we performed a longitudinal study by monitoring the systolic LV function, volume and mass with high resolution MR imaging and by profiling the myocardial proteome in short intervals during initiation and progression of disease. In considering differences between LV dysfunction in pressure overload and consecutive development of right ventricular dysfunction due to left ventricular backward failure, we profiled the left and the right ventricle (RV) separately to characterize similarities and differences between both ventricles in protein levels at the different disease stages beginning with left ventricular hypertrophy and, finally, ending with heart failure. We were mainly interested in changes of the protein profile that are associated with hypertrophy in the early phase of LV dysfunction as this is the first manifestation of a subclinical organ damage. Thereby we could identify HSPB7 as a novel biomarker candidate, which was present at higher level and highly abundant in heart tissue and in blood serum in the early phase of pressure overload. This protein could have the potential to be a new diagnostic marker for subclinical stage of beginning HF.

## Methods

A detailed description of methods (S1 Text) and a sampling scheme (S1 Fig) is provided in the supplement material.

### Ethics statement

All experimental procedures and protocols performed on animals were carried out in compliance with the Guide for the Care and Use of Laboratory Animals published by the U.S.NIH (NIH Publication no. 85–23, revised 1985). The protocols were approved by the local animal care committee (Landesamt für Landwirtschaft, Lebensmittelsicherheit und Fischerei Mecklenburg-Vorpommern; LALLF MV, number 7221.3–1.1-058/11).

### TAC surgery, MRI measurements and sample preparation

To study the transition from hypertrophy to heart failure the murine model of pressure overload by transverse aortic constriction (TAC) was used. Hence, 8 weeks old male C57BL/6N mice (Charles River Laboratories, Sulzfeld, Germany) underwent either TAC or sham surgery. For surgery, mice were initially anesthetized with 3% isoflurane, intubated and ventilated (2% isoflurane in air, 50 ml/min; rodent MiniVent, Harvard Apparatus, Germany). The thoracic cavity was opened by a *median sternotomy* until the 3^rd^ rip and a blunted needle (26G) was placed on the aortic arch between the innominate artery and left common carotid. A 7–0 suture was tied onto the needle. Immediately afterwards, the needle was removed and the thoracic cavity was closed. Similarly, the sham surgery was performed except knotting the suture. Before and for 24 h after surgery, the animals were treated with analgesic medication (Buprenorphine 0.02 mg/kg s.c.) and monitored daily until the end of the study. The animals were housed under standard conditions with a 12 h light-dark cycle and controlled temperature (21°C) regime. Water and food were provided ad libitum.

Cardiac function of sham and TAC mice was evaluated at day 4, 14, 21, 28, 42, and 56 after surgery by cardiac MRI. At each time point, 6 sham and 6 TAC mice were examined. Additionally, the heart function of 6 mice without surgery was determined. Immediately after MRI, the mice were euthanized with Thiopental (300 mg/kg) and blood was taken from the heart. The heart was removed, dissected in the left and right ventricles and snap-frozen.

### LC-MS/MS analysis

Protein extracts of individual samples were prepared in a bead mill and protein concentrations determined with a Bradford assay (BioRad, Munich, Germany). From each postoperative time point pooled samples were generated by mixing equal protein amounts of either 6 left ventricles or 6 right ventricles of TAC and sham animals. Additionally, pooled samples were generated from right or left ventricles of 6 control animals at baseline. Thus, a total of 26 different samples were subjected to global protein profiling (S1 Fig). After denaturation and digestion with LysC and trypsin [8], the resulting peptides were purified and subjected to liquid chromatography (LC)-electro spray ionization (ESI)- tandem mass spectrometry (MS/MS). Peptides and proteins were identified via a Sequest search (Sorcerer built 4.04, Sage-N Research) in a murine forward/reverse Uniprot/SProt database (rel. 06/2012). For relative quantification mass spectrometry (MS) data was processed by Rosetta Elucidator^®^ [9]. Proteins (≥1 peptide hits) displaying an at least two fold change in intensity were considered as altered in abundance in comparison to the sham group. A detailed description of the method is provided in S1 Text. Raw data are available via ProteomeXchange with identifier PXD007171.

### Western Blot and ELISA

For validation of MS protein data, Western Blot analysis of selected proteins of individual samples of TAC and sham mice at the different time points was performed. Detected intensities were normalized to total protein content. Accordingly, HSPB7 concentrations of serum samples were analyzed by ELISA according to manufacturer’s protocol (Mouse Heat Shock Protein Beta-7 ELISA Kit, Cusabio ^®^, USA).

### Statistical analysis

When data were determined for individual animals, mean values per group and the corresponding standard deviation (SD were calculated. Mann-Whitney-U-Test was used to compare the heart function of TAC and sham animals for each time point. For functional classification and analysis of proteins displaying significantly altered levels Ingenuity^®^Pathway Analysis (IPA^®^, QIAGEN Redwood City, www.qiagen.com/ingenuity) and GO enrichment analysis (www.geneontology.org) were used. Significance values associated with an enrichment of proteins within functional categories were calculated using the Fisher’s exact test. The ELISA results were tested with a two-way ANOVA and a Bonferroni post-test. All p-values less than 0.05 were considered statistically significant.

## Results

### Left ventricular systolic function and remodeling after TAC surgery

**Fig 1:**
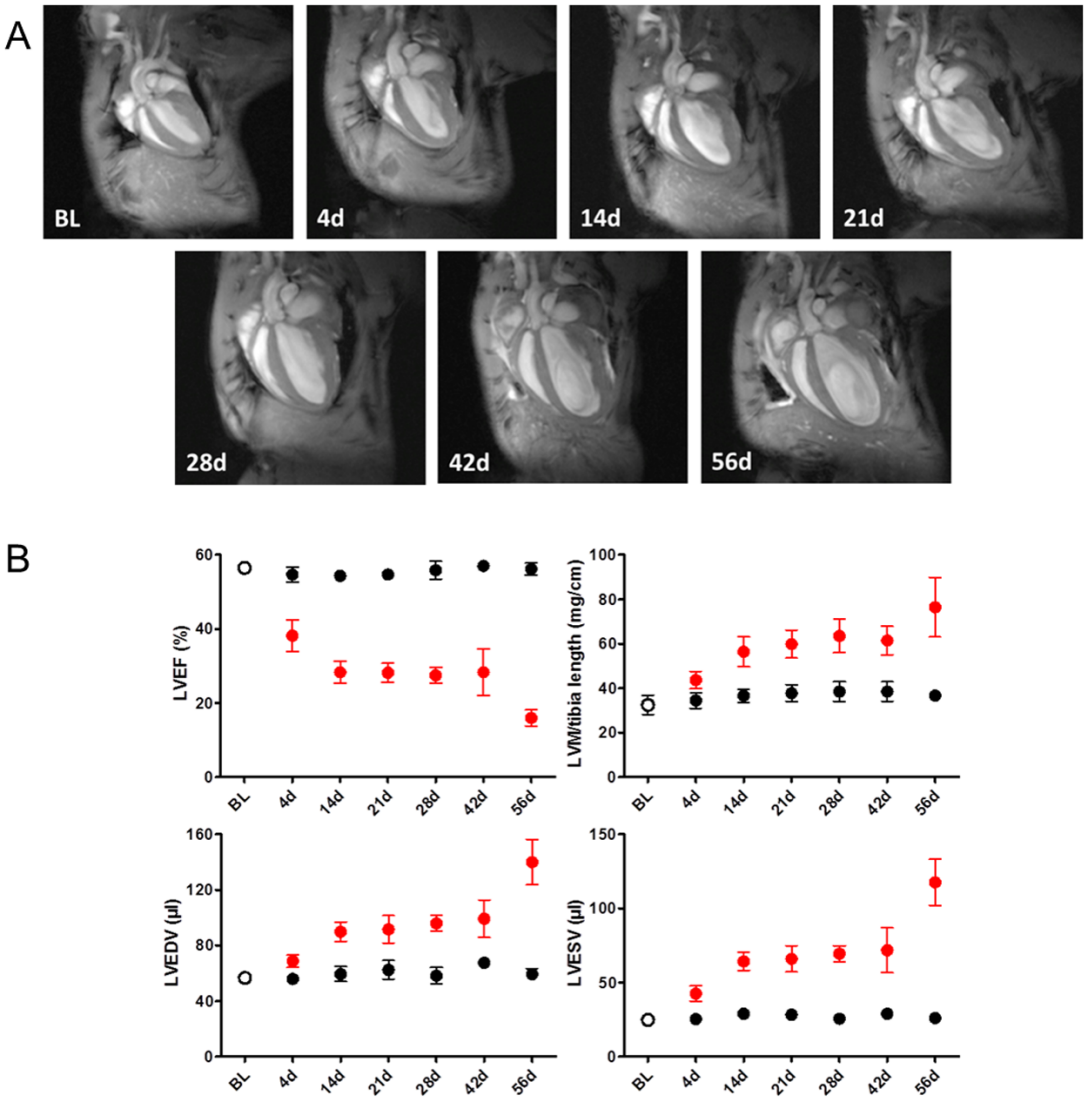
Kinetics of cardiac parameters in TAC and sham mice as determined by MRI. (A) The upper panel shows representative pictures of cardiac MRI measurements in mice that underwent transverse aortic constriction (TAC) surgery. Four chamber views in systole from baseline (BL) up to day 56 indicate worsening heart function and ventricle dilation over time. (B) The lower panel shows cardiac parameters examined by MRI of sham treated (black filled circles) and TAC (red filled circles) mice. Mice without surgery (open circle) served as controls at baseline (BL). Left ventricular ejection fraction (LVEF), left ventricular mass (LVM), left ventricular end-diastolic volume (LVEDV), left ventricular end-systolic volume (LVESV). Data are presented as group means ±SD (n = 6 for each time point).

In order to study the ventricular volumes, mass, and systolic function of the LVs after induction of pressure overload, the murine TAC model was used. The LV parameters were evaluated by cardiac MRI at day 4, 14, 21, 28, 42, and 56 after surgery. At each time point, 6 TAC and 6 sham mice were scanned (Fig 1A). Additionally, 6 mice without surgery served as control group at baseline. At each time point during follow-up, TAC mice exhibited a reproducible extent of LV hypertrophy within their group. In TAC mice, disease progression was mirrored by loss of LV systolic function and development of LV dilation and hypertrophy over time. Compared to sham controls, the LV systolic function of TAC mice was significantly reduced at all examined time points: LV ejection fraction (LVEF) decreased from 56.5±1.2% at baseline to 38.2±4.3% at day 4 and 28.3±2.9% at day 14, stayed stable thereafter until day 42 (28.1±3.6%) and finally further declined at day 56 (16±2.2%), whereas LVEF of sham mice remained unchanged over time. The decrease in LVEF was paralleled by an increase in LV end-diastolic volume (LVEDV), end-systolic volume (LVESV), and left ventricular mass-to-tibia-length ratio (LVM/TL) reflecting the time course from compensated LV hypertrophy to development of LV systolic dysfunction and remodeling during the second and the sixth week and, finally, to onset of decompensated HF after 56 days which was demonstrated by the pronounced decrease of LVEF as well as the excessive increase in LVEDV and LVESV (Fig 1B, S1 Table [including statistical analyses]).

### Time dependent proteomic profiling after TAC

**Fig 2:**
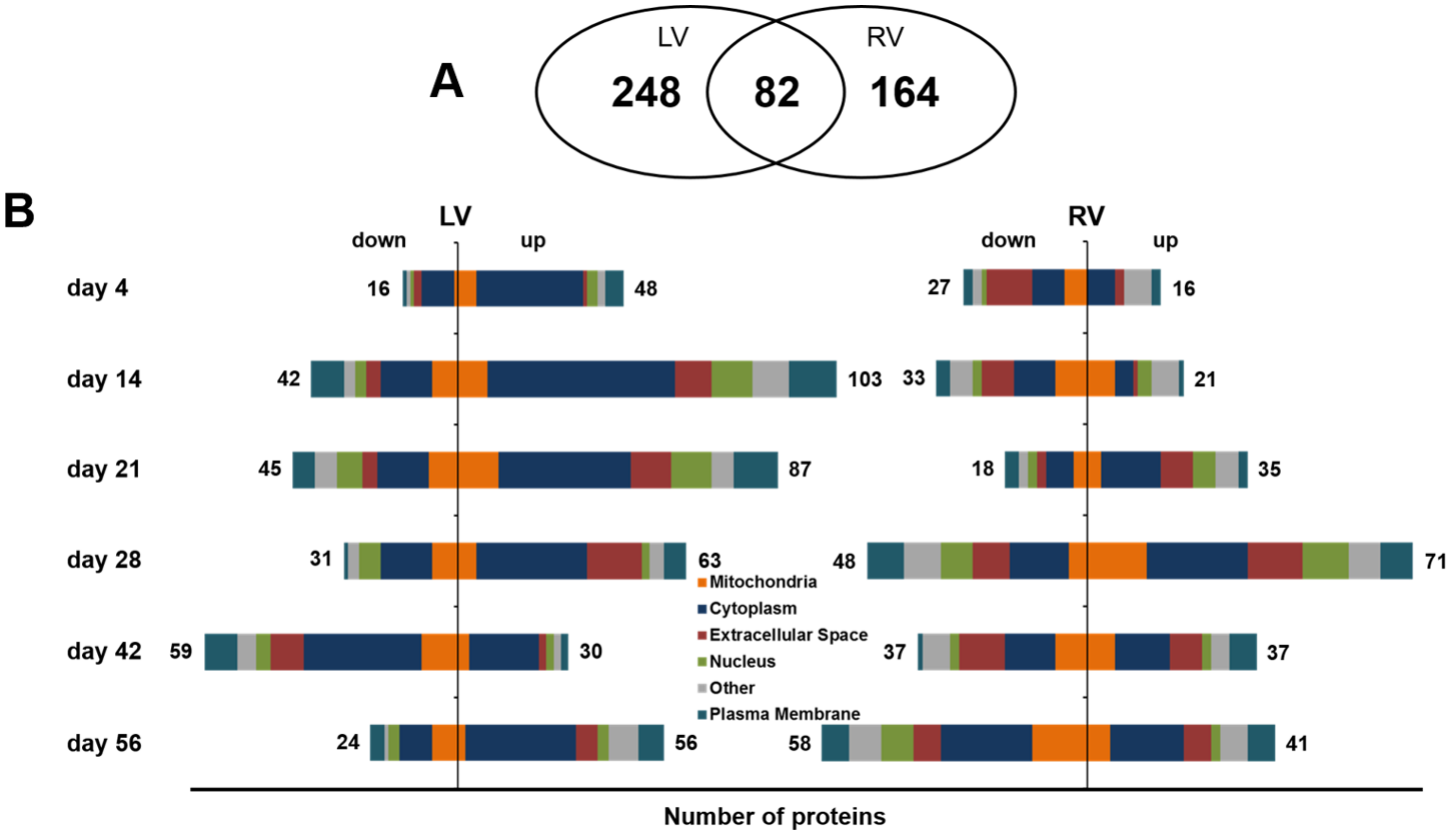
TAC-induced changes in protein abundance. The number of proteins with an at least 2-fold change in abundance TAC vs. sham are shown. (A**)** Over time, 330 different proteins in the left ventricle (LV) and 246 proteins in the right ventricle (RV) were altered after TAC, of which 82 displayed alterations in both ventricles. **(**B**)** The number of altered proteins per time point. The strongest changes in protein pattern in LV were observed in the early phase after TAC at day 14 and 21, whereas strongest differences in RV were seen at day 28, 42, and 56. Especially cytoplasmic and mitochondrial proteins were affected.

For the proteomic profiling, a tandem mass spectrometry (MS/MS) analysis was performed (Raw data are available via ProteomeXchange with identifier PXD007171). The analysis of four randomly picked left and right ventricles of control mice at baseline, as well as of individual TAC and sham mice after 21 and 56 days (n = 6 per group) revealed a low variance within each surgery group (S2 Fig). Hence, more than 80% of all proteins displayed a variance less than 20% being in the range of technical variation introduced by sample preparation. Therefore, further MS analysis was performed using pools of the 6 left as well as right ventricles of baseline, TAC and sham mice at each time point. For the identification of alterations in the TAC group in comparison to the age-matched sham group a strict fold change of >2 was applied to keep the false positive rate low. The proteome analysis covered in total 1297 proteins of the LV (Table A in S2 Table) and 1225 proteins of the RV (Table B in S2 Table). With an overlap of more than 80% in protein identifications, relative quantification of proteins revealed only minor differences between the protein pattern of the LV and RV at baseline (S3 Table). After TAC, 330 and 246 proteins displayed altered levels in LV and RV (S4 Table), respectively, compared to sham animals (TAC vs. sham), of which 82 proteins (S5 Table) showed altered abundance in both ventricles (Fig 2). In the phase between day 14 and 42, the most prominent changes in protein pattern could be observed. Dealing with the increase in afterload, LV showed the highest number of altered proteins in the early phase after TAC at day 14 (103↑, 42↓) and 21 (87↑, 45↓). In the RV, the changes in protein pattern in response to TAC were delayed compared to LV. Here, the strongest changes in the protein pattern were recorded after 28 days (71↑, 48↓) and at later time points 42 (37↑, 37↓) and 56 days (41↑, 58↓) after TAC (Fig 2).

Regarding the location of the proteins that showed differences in quantity between the TAC and the sham group, cytoplasmic proteins constituted the largest group in both ventricles at all examined time points (Fig 2).

### Functional categorization of changed proteins

**Fig 3:**
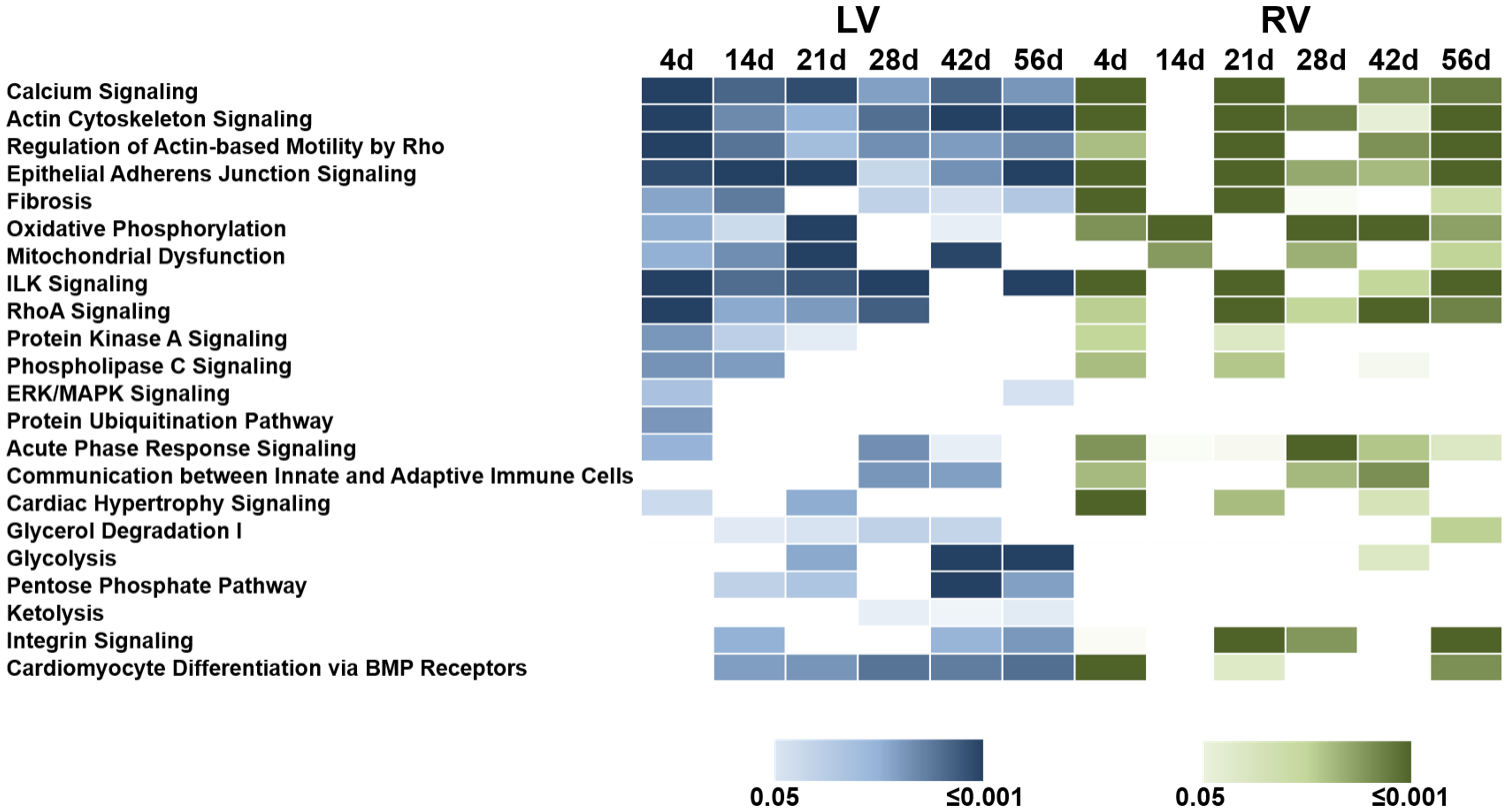
Heat map of canonical pathways affected during disease progression in LV and RV. Canonical pathways with a significant enrichment of altered proteins (TAC vs. sham; fc≥2) are displayed for each examined time point for LV and RV. Significance values were assessed using Fisher’s exact test and are shown for LV from light blue (p<0.05) to dark blue (p<0.001) and for RV from light green (p<0.05) to dark green (p<0.001).

To investigate the affected biological pathways during heart failure transition, a classification of all differentially abundant proteins in LV and RV using Ingenuity Pathway Analysis (IPA) was conducted. The pathways in which altered proteins in the LV and RV have been found significantly enriched (Fisher’s exact test, p<0.05) at one or more time points are shown in Fig 3. The top categories included *calcium signaling* (e.g. calmodulin (CAM), ryanodine receptor (RYR1) and myosin light chain (MYL1/4)), *actin signaling*, and the so-called *regulation of actin-based motility*. Interestingly, at the early phase after TAC at days 4 until 28, proteins assigned to the signaling *pathways of Rho A*-, *PLC* (phospholipase C) and *PKA* (protein kinase A) displayed significant changes of their abundance only in the LV of TAC mice. Pathways of mitochondrial function such as *oxidative phosphorylation* and *mitochondrial dysfunction* were strongly influenced in the LV from day 4 to day 21 and at day 42 after TAC, whereas in the RV an enrichment of proteins of these categories could be seen at day 14 and at later time points (28d, 42d, 56d). In LV, the later time points were dominated by changes in metabolic pathways including *glycolysis*, *ketolysis*, and *pentose phosphate pathway*. In particular, proteins of the fatty acid metabolism including the carboxylesterase 1D (CES1D) and the trans-2, 3-enoyl-CoA reductase (TECR) as well as enzymes of gluconeogenesis such as the fructose-1,6-bisphosphatase (F16P2) were found in far less amounts compared to sham, whereas proteins of glycolysis like 6-phosphofructokinase (K6PP) -highly expressed in the fetal heart- were highly abundant in LV (S4 Table). In contrast to LV, proteins of metabolic processes have not been altered significantly in the RV at this time (Fig 3).

**Fig 4:**
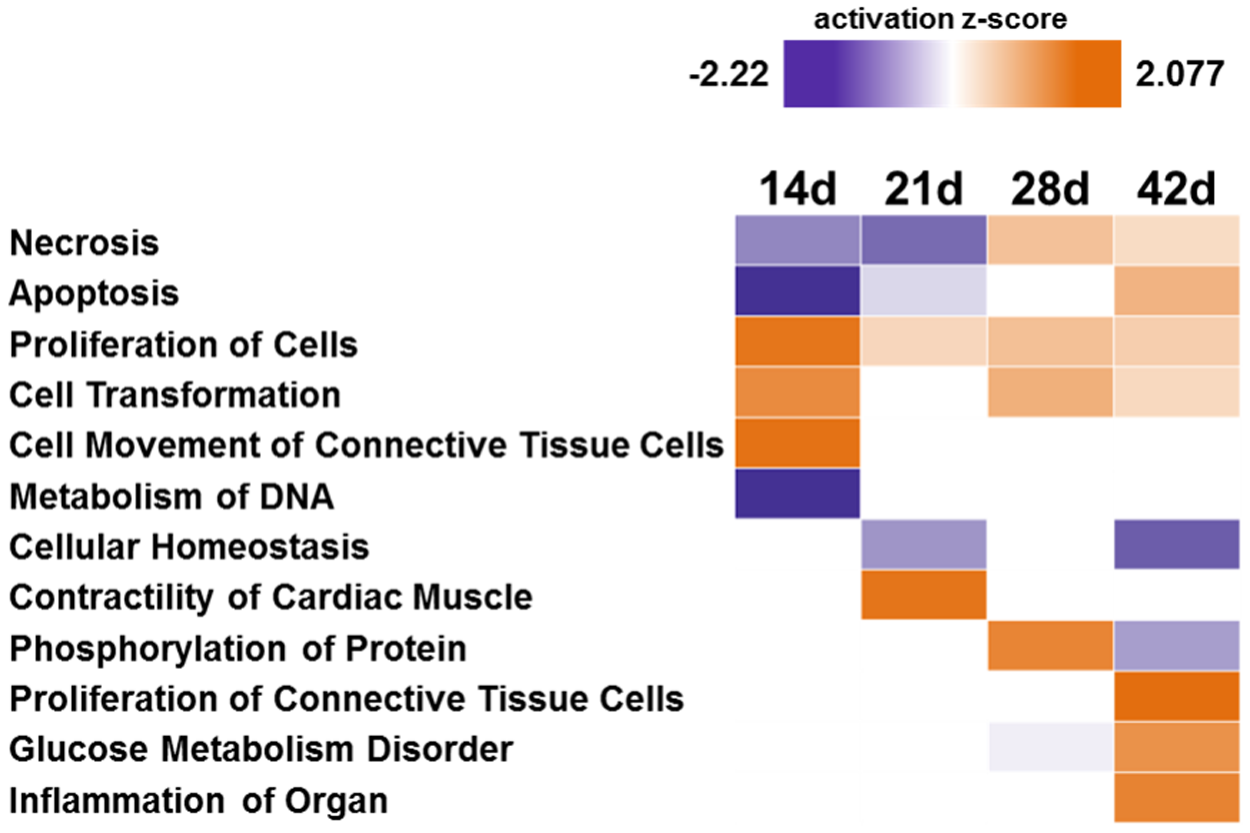
IPA based biological function activity analysis. For analysis all proteins of LV which showed differential abundance (TAC vs. sham, fc≥2) only in the time period between day 14 and 42 after TAC were used. A positive z-score predicts activation (orange), a negative score (purple) predicts inhibition of the biological process.

The strong changes in protein levels in the LV between day 14 and 42 point to processes those are necessary for the stabilization of heart function, which was seen in the stage of stable LVEF. To investigate which biological processes and modifications were involved especially in this period, an IPA based analysis of LV proteins altered at these time points regarding their impact on the activation or inhibition of biological functions was conducted. The IPA-derived activity z-score (Fig 4) revealed an initially activated proliferation (e.g. FGF1 ↑, COL1A2 ↑, full list in S6 Table) and an inhibition of cell death (e.g. NOL3 ↑, EIF3B ↑, CAST ↑, full list in S6 Table) at day 14 and 21. Mass spectrometric results have been exemplarily verified by Western Blot analysis of individual samples for the eukaryotic translation initiation factor 3 subunit B (EIF3B, S3 Fig).

### Biomarker candidates

**Fig 5:**
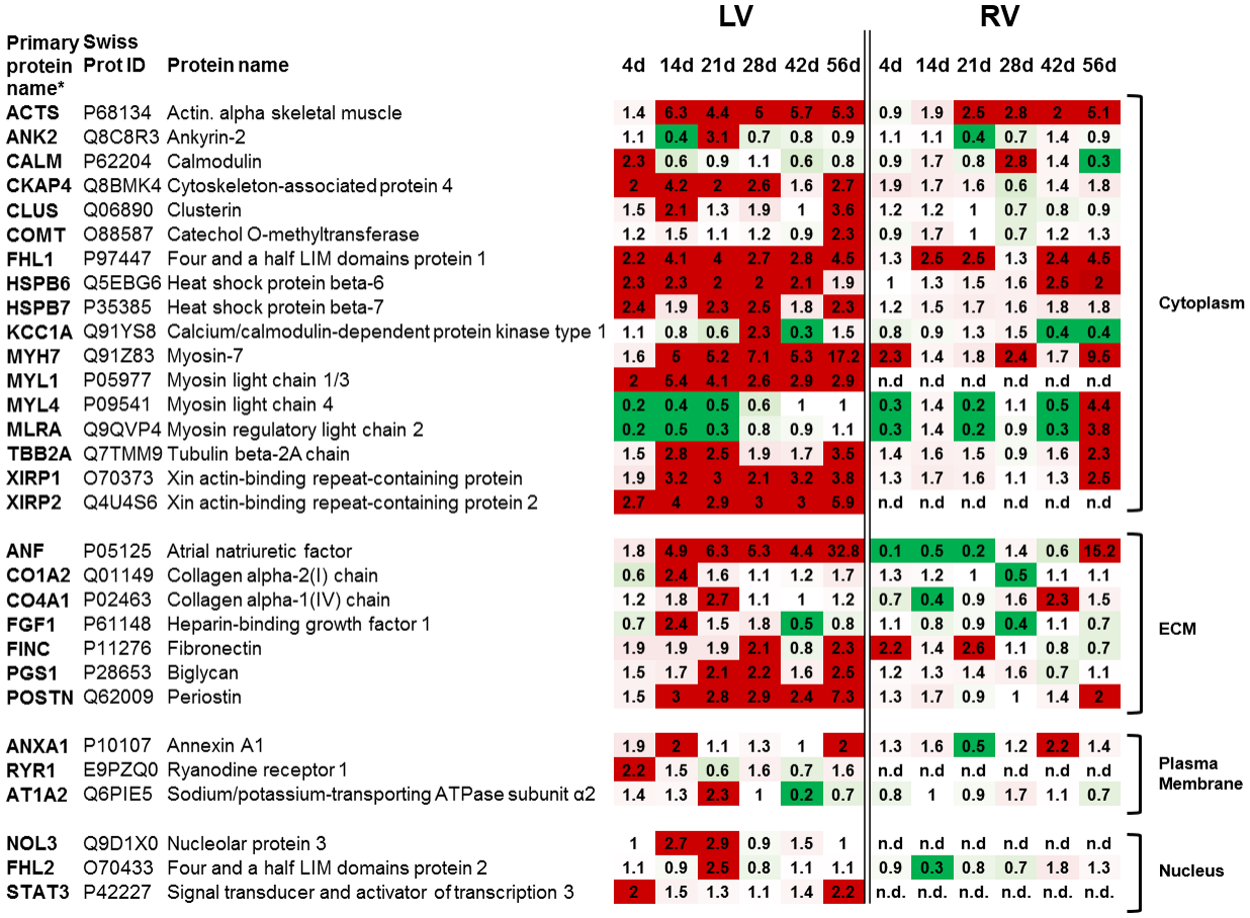
Heat map of abundance changes of proteins associated with cardiovascular diseases. Ratios (TAC/sham) of proteins in left (LV) and right ventricle (RV) with a ≥2 fold change on at least one examined time point. The changes of common HF markers like ANF (ANP), MYH7 (β-MHC) and ACTS (α-actin) were markedly higher in LV than in RV especially at the early time points. After 56 days in both ventricles comparable alterations regarding these proteins were observed. ECM (extracellular membrane), n.d. (not detected) * Labeling as exported from Rosetta Elucidator^®^ package.

**Fig 6:**
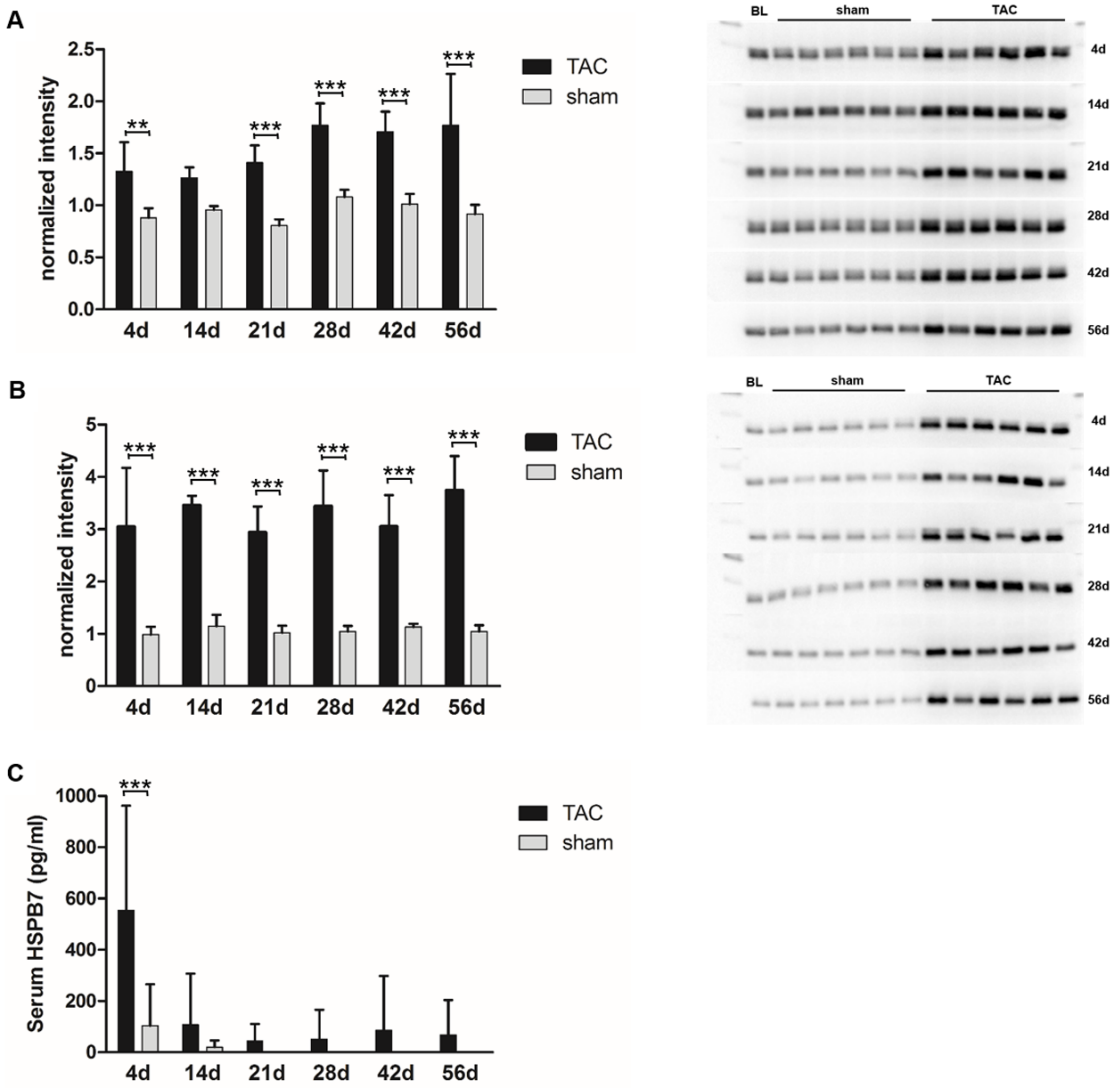
Abundances of small heat shock proteins. (A) Intensities (normalized to total protein) of tissue HSPB6 detected by Western Blot analysis on individual sample level (n = 6 per group/time point). Over all time points HSPB6 abundances are significantly higher in TAC mice compared to sham mice (two-way ANOVA, Bonferroni post-test, *** p<0.001; ** p<0.01). (B) Intensities (normalized to total protein) of HSPB7 detected by western Blot analysis on individual sample level (n = 6 per group/time point). Already at day 4 HSPB7 could be found in significantly higher levels in the heart tissue of TAC mice when compared to sham (two-way ANOVA, Bonferroni post-test, *** p<0.001). The high expression remained over all time points and is comparable to the proteome analysis. (C) Serum concentrations of HSPB7 analyzed by ELISA on individual sample level (n = 5–6 per group/time point) showed a significant (p<0.001, two-way ANOVA, Bonferroni post-test) increase in TAC mice after 4 days of pressure overload. Higher amounts of protein could be found also at all other time points in TAC mice, whereas HSPB7 was not detectable in the sham group.

Many previously described HF related proteins were also detected by this approach; a selection is shown in Fig 5. The abundance of the known HF markers, the so-called fetal proteins such as ANP, β-MHC, and α-actin in LV of TAC mice was already higher at day 4, further increased at day 14 and finally showed the strongest differences 56 days after surgery. In the RV ANP, β-MHC, and α-actin showed far less or no alterations (TAC vs. sham) even until day 42 when compared to the LV. Only at day 56 after TAC, the abundance of these proteins increased in RV but did not reach the extent observed in the LV. These known HF markers showed distinct changes in LV over time, but were not the strongest indicators of the emerging disease. Early indicators of HF are the xin actin-binding proteins 1 and 2 (XIRP1, XIRP2). In particular, XIRP2 was strongly up-regulated from day 4 until day 56 in LV. Both XIRPs showed high abundances in LV but were either not detectable or unchanged until day 56 in RV. With regard to the extracellular matrix, the non-collagen proteins periostin and biglycan showed increasing levels over time, whereas the collagens showed a higher abundance only at day 14 and 21. Further representatives of early indicator proteins are the heat shock proteins B6 and B7 (HSPB6, HSPB7), belonging to the group of proteins that showed a prominent increase already at day 4 with a continuing high abundance over time. This increase was again specific for LV. In RV, HSPB6 and 7 could not be found in higher amounts before day 42, which underlines the delayed manifestation of backward failure. The dynamic changes of HSPB6 and 7 over time were tested on single sample level by Western Blot analysis and showed results consistent with the time resolved proteome analysis (Fig 6A and 6B). Because of the high expression of HSPB7 and the finding that HSPB7 is secreted to the blood [10], serum samples of TAC and sham animals were analyzed by ELISA at the different time points. Despite the small sample size and the resulting high standard deviations, we detected a significant increase in serum HSPB7 4 days after TAC and elevated serum levels even after 14 days (Fig 6C). Furthermore, a trend to higher amounts of HSPB7 serum levels up to 56 days could be observed in TAC vs. sham animals.

## Discussion

Heart failure is characterized by specific changes in structure, cellular functions, and metabolism, which contribute to the process of remodeling and the phenotype of reduced ventricular contractility. This study provides an overview of changes in the protein profiles during the time course of development of LV systolic dysfunction and remodeling to HF progression. In contrast to previous profiling studies [5–7, 11], we considered the time dependent response in protein levels from early to late disease stages of both the LV and RV to the increased afterload generated by TAC separately. The MRI based evaluation of LV function in TAC and sham mice at the different time points after surgery enabled us to exactly define the disease stages during the longitudinal follow-up. In our experimental setup, we could observe systolic LV function worsening from day 4 to 14 in TAC mice. Systolic LV function remained on a stable level from day 14 to 42 reflecting the compensated phase of HF and then showed a pronounced deterioration at day 56, which represents the decompensated end-stage of HF.

Already in the initial phase in the LV molecular pathways that are known to be involved in progression of hypertrophy and HF [2, 12, 13] like calcium handling and actin cytoskeleton signaling were altered and remained affected across all time points. In parallel, small heat shock proteins (sHSP) were accumulated in the LV, but remained unaffected in RV until day 42, which gives a hint to the delayed onset of LV backward failure. The production of small HSPs is enhanced in ischemic myocytes and is regarded to increase the tolerance against stress induced cell damage [6, 14]. HSPB7 (cvHSP) is selectively expressed in cardiac muscle and assumed as an early (1-3h) biomarker of myocardial infarction [10, 15]. Genome wide association studies identified *HSPB7* as a SNP associated risk gene for idiopathic cardiomyopathies and heart failure [16–18]. However, little is known about the function of this protein. HSPB7 has been described to protect against tachycardia remodeling by attenuation of the Rho A GTPase pathways in vitro [19]. In the present study, we could demonstrate for the first time that HSPB7 is upregulated in LV myocardial tissue after TAC, which is paralleled by an increase in serum levels. Therefore, it can be deduced that this protein is not only an acute biomarker of myocardial damage and necrosis, but also of myocardial stress as a consequence of pressure overload up to 14 days after induction. In line with these findings we observed an enrichment of proteins altered at early time points assigned to the RhoA signaling pathway. HSPB7 is assumed to directly prevent F-actin stress fiber formation, which is a downstream consequence of the maladaptive Rho A GTPase activation [19]. In combination with other clinical biomarkers, like NT-pro BNP, HSPB7 could have the potential to disclose the onset of HF. An early indicator in a subclinical stage before damage occurs, would allow an early pharmaceutical intervention. Further HSPB7 is an interesting target, which may counteract the maladaptive processes during the subclinical phase of HF. But further investigation of its cardioprotective potential and the clinical association of HSPB7 are needed.

The largest number of changed proteins could be detected between day 14 and day 42 in LV pointing to the importance of this period for disease progression. Extensive changes were observed for extracellular matrix proteins, underlining the important role of the reorganization of existing structures in the myocardium during HF development. The increased amount of collagens and secreted factors leads to changes in composition, distribution and an overall enlargement of the ECM, which is known to have a direct negative impact on the left ventricular performance [20, 21]. The accumulation of collagen during myocardial remodeling is complex. In the LV, collagen deposition was found only at days 14 and 21, whereas other matrix proteins like periostin, fibronectin, and biglycan maintained high levels up to the decompensation phase. Hence, the remodeling of the extracellular matrix seems not only to be dependent on the abundance of collagen, but also of other matricellular proteins. The finding that collagen accumulation does not keep pace with the stage of cardiac hypertrophy has also been reported by other groups using different models of HF [22–24].

In the period of already impaired, but stable systolic heart function, an inhibition of necrotic and apoptotic processes and an activation of cell proliferation took place. Together with the high abundance of fetal proteins and the increased amount of cytoskeleton and structural proteins displaying a significant and constant alteration of the actin-cytoskeleton signaling and cell proliferation indicates a hypertrophic growth and might reflect a compensatory mechanism thus providing support for the heart in response to pressure overload. Hence, the first phase of compensation in HF (14d-21d) is rather determined by cell and tissue growth than by metabolic adaptation.

In the second phase of compensation at day 28 and 42, a shift to activated cell death was observed. This was paralleled by a disturbance of metabolic processes, inflammation, and proliferation of connective tissue. The explicit activation of connective tissue growth reflects the excessive increase of extracellular matrix, which impairs the adequate oxygen supply of tissue finally leading to changes in energy metabolism and cell death [25]. This could be a hint to the upcoming deterioration of heart function and progressive ventricle dilation. The initial adaptive process of hypertrophy turns to a maladaptive process at this stage suggesting that the heart shows different stages of hypertrophy, not seen in functional parameters (such as LVEF) but only on the molecular level. When the timeline of the early compensated phase is exceeded and metabolic changes occur, the transition to the end-stage of HF is initiated.

Metabolic remodeling in HF is characterized by a reduced energy production, which possibly leads to limited substrate utilization and mitochondrial dysfunction [25]. In our study, impairment of metabolic processes such as glycolysis and pentose phosphate pathway gained importance over time but was limited to the LV only. Nevertheless the findings underline the entry in the decompensatory stage. The regulation of the ‘metabolic’ proteins implies a shift from fatty acid to glucose metabolism in the failing heart. The reversion to the “fetal metabolic phenotype” of glucose utilization that has a greater efficiency in producing high energy phosphates is considered to be adaptive [26, 27]. The late onset of metabolic conversion seemed to be a response to the detrimental structural and molecular changes of the heart and, hence, is consequently the last resort of adaption in HF. This metabolic shift seen in LV may not be reached in RV in the experimental time frame due to prematurely LV failure.

In this study we could provide a comprehensive overview of alterations in the ventricular protein profile during the different phases of transition to heart failure. The data have been recorded on pooled samples and thus do not display biological variation. Due to the applied cut-off value of two-fold difference, rather small changes might not have been identified. Therefore, also other indicators of early heart failure might exist, but could not be addressed in the current study. We identified small heat shock protein HSPB7 as a promising biomarker candidate for the onset of HF and could demonstrate that this protein is also detectable in serum during the early subclinical stage of HF. Additional proteins also displaying disease stage dependent changes in level might be further biomarker candidates (e.g. the XIRPs) or may not only constitute new early makers of heart failure development but also new targets of novel therapeutic approaches.

## Supporting information

S1 Text Material and methods. (DOCX)

S1 Fig Sampling scheme and analysis strategy. (A) Displayed are the numbers of individual samples used for MRI, estimation of biological variance (Estimat. of BV), LC-MS/MS (proteomics) and Western Blot analysis. (B) Shown is the analysis strategy.
(TIF)

S2 Fig Distribution of the coefficient of variation of all identified proteins on single sample level. All identified proteins of the individual left ventricles of each TAC mice at day 21 and 56 (TAC21d LV, TAC 56d LV) and each left and right ventricle of baseline mice (Base LV, Base RV) are shown in consecutive numbering along the x-axis. The corresponding coefficient of variation is displayed on the y-axis.
(TIF)

S3 Fig Western Blot analysis of EIF3B. Intensities (normalized to total protein) of eukaryotic translation initiation factor 3 subunit B in tissue (EIF3B) detected by Western Blot analysis on individual sample level (n = 6 per group and time point). Especially, at day 14 EIF3B showed a significantly higher abundance in TAC compared to sham left ventricular tissue (two-way ANOVA, Bonferroni post-test, *** p<0.001).
(TIF)

S1 Table Cardiac parameters of sham and TAC treated mice examined by MRI. Left ventricular ejection fraction (LVEF), left ventricular mass (LVM) normalized to tibia length, left ventricular end-diastolic volume (LVEDV) and left ventricular end-systolic volume (LVESV) of baseline (BL), sham and TAC mice after 4, 14, 21, 28, 42, and 56 days after surgery. All examined cardiac parameters showed a significant deterioration of the values of TAC mice compared to their sham group (**p≤0.01, * p≤0.05; Mann-Whitney-U-Test, TAC vs. sham).
(PDF)

S2 Table Proteins identified in the left ventricle (A) and the right ventricle (B). (PDF)

S3 Table Proteins of left and right ventricle at baseline. In the table all proteins, which show under basal conditions an at least two fold difference (FC) of abundance in the left ventricle when compared to the right ventricle, are listed. * Labeling as exported from Rosetta Elucidator^®^ package.
(PDF)

S4 Table Changes in protein abundance after transverse aortic constriction (TAC). The table displays the protein alterations in the TAC group in comparison to the age matched sham group (ratios TAC/sham) in the left ventricle (LV) and/or right ventricle (RV) with an at least two fold change (≥1 peptide hits) in abundance at one or more of the examined time points day 4, 14, 21, 28, 42, and 56. Red color shows increased and green color shows decreased protein levels in TAC mice in comparison to sham animals. * Labeling as exported from Rosetta Elucidator^®^ package.
(PDF)

S5 Table Proteins altered in LV and RV after transverse aortic constriction (TAC). The table displays the ratios (TAC/sham) of proteins which showed altered abundance in both ventricles. * Labeling as exported from Rosetta Elucidator^®^ package.
(PDF)

S6 Table Proteins of IPA activity z-score analysis. The table shows the subset of proteins, which was assigned to the IPA z-Score categories *proliferation of cells*, *necrosis*, and *apoptosis*. * Labeling as exported from Rosetta Elucidator^®^ package.
(PDF)
