# Supplementary material for: Dynamic adaptation of myocardial proteome during heart failure development
Source: PLoS One. 2017 Oct 3;12(10):e0185915. doi: 10.1371/journal.pone.0185915 (PMC5626523; doi:10.1371/journal.pone.0185915)
Supplement: S1 Text — (DOCX) [file pone.0185915.s001.docx]

**S1 Text. Material and Methods**

**Cardiac MRI measurement and sample preparation**

Cardiac function of sham and TAC mice was evaluated by magnetic resonance imaging (MRI) (7 Tesla ClinScan small animal MRI, Bruker, Billerica, MA, USA). MRIs were performed at day 4, 14, 21, 28, 42 and 56 after surgery. At each time point, measurements took place in 6 sham and 6 TAC mice. In 6 mice which served as baseline controls imaging was additionally performed without previous surgery. Image data were generated through 2D-gradient echo sequence (repetition time: 5.7 ms, echo time: 2.25 ms, flip angle: 25°, field of view (FOV): 35×35 mm, slice thickness: 1 mm, gap: 0.2 mm, resolution: 192×192 pixel). Images were recorded in 2- and 4- chamber view (CV) and short axis view (SAX). Left ventricular ejection fraction was calculated using left ventricular volumes, which were analyzed by using Segment Software (Medviso, Lund, Sweden) according to Simpson’s rule. Left ventricular mass was calculated from left myocardial tissue volume and the density of myocardial tissue (1.05 g/cm^3^).

Immediately after MRI, the mice were euthanised; the blood was taken from heart and the hearts were perfused with ice cold saline (0.9%) before sampling. Thereafter, the left and right ventricles were dissected and snap-frozen. Under liquid nitrogen, the tissue of each ventricle was separately grounded into powder. For serum preparation, blood was allowed to clot for 30 min at room temperature and centrifuge at 250g for 15 min. The serum supernantant was collected and snap-frozen.

**LC-MS/MS**

The tissue powder of the left and right ventricles was further homogenized in cold buffer (8 M urea, 2 M thiourea) using a bead mill [[1](#_ENREF_1)]. Tissue homogenates were centrifuged (1 h, 13000 rpm, 4°C) and the supernatants stored at -80°C until further use.

For each time point, protein extracts of 6 left and 6 right ventricles of TAC and sham animals as well as of control animals at baseline were pooled for global protein profiling resulting in 26 different samples (right and left ventricular samples at baseline, and 4, 14, 21, 28, 42, and 56 days after surgery from TAC and sham mice, a scheme for pooling is shown in S1 Fig). Samples were subsequently treated with 2.5 mM DTT (60°C, 1 hour) and 10 mM iodoacetamide (37°C, 30 minutes), followed by LysC digestion (10 ng per 1 µg protein) at 37°C for 3 hours and trypsin digestion (100 ng per 1 µg protein) at 37°C for 16-18 hours. The resulting peptides were cleaned using reversed phase material (µ-C18 ZipTips, Millipore, Billerica, MA, USA) [[2](#_ENREF_2)] and subjected to liquid chromatography (LC)-electro spray ionization (ESI)- tandem mass spectrometry (MS/MS). Separation of peptides was performed with a nanoAquity UPLC system (Waters Corporation, Manchester, UK) on C18 reverse phase material (10 cm length, 100 μm i.d., 1.7 μm particle size, Waters) in a non-linear gradient of 1–5% buffer B in 2 min, 5–25% B in 63 min, 25–60% in 25 min, and 60–99% B in 2 min (buffer A - 2% ACN in 0.1% acetic acid, buffer B - ACN in 0.1% acetic acid (99 min, flow: 400 nl/min). The detection was carried out with a LTQ-Orbitrap-Velos mass spectrometer (Thermo Electron, Bremen, Germany) in data-dependent mode selecting 20 precursor ions with the highest intensities for CID fragmentation per cycle. Exclusion time was set to 60 s to minimize redundant fragmentation of precursors. Peptides and proteins were identified via a Sequest search (Sorcerer built 4.04, Sage-N Research Inc., Milpitas, CA, U.S.A.) in a murine forward/reverse Uniprot/SProt database (rel. 06/2012). A statistical evaluation of these results was performed by analysis in peptide/protein prophet and the annotation of peptides was carried out at a false-positive rate of <1% that is equivalent to a peptide probability >0.94 (LV) or 0.95 (RV). For the left ventricle and right ventricle 1318 or 1234 proteins were identified respectively. For relative quantification mass spectrometry (MS) data were processed by Rosetta Elucidator® to extract peak intensities [[2](#_ENREF_2)]. After median normalization, annotated peaks of protein-specific peptides were summed up and used for relative quantification of proteins. Due to missing statistics only proteins identified with an at least two fold change were considered as altered in abundance in comparison to the age matched sham group. Proteins were assigned to biological processes, metabolic and signaling pathways through the use of IPA (Ingenuity® Systems, www.ingenuity.com). Information on the cellular localization was obtained from UniProtKB (Universal Protein Resource Knowledgebase).

**Western Blot analysis**

To validate the results of the MS analysis, relative abundances of selected proteins were evaluated by Western blotting. Individual samples of TAC and sham mice from each time point were used. Protein extracts (50µg each) were separated by PAGE using TGX Stain-Free^™^ FastCast^™^ acrylamide gels for in-gel protein labeling and transferred by Trans-Blot^®^ Turbo^™^ semidry blotting on nitrocellulose membrane (Biorad®) according to manufacturers protocol. Immunoreactive bands were detected by enhanced chemiluminescence with Clarity Western ECL substrate (Biorad®) and normalized to total protein.

The following antibodies and concentrations were used: rabbit monoclonal Anti-Hsp20 (HSPB6) (Abcam) 1:50000, rabbit monoclonal Anti-cvHSP (HSPB7) (Abcam) 1:10000, rabbit monoclonal Anti-eIF3B (Abcam) 1:10000, HRP-linked Anti-rabbit IgG (Cell Signaling) 1:5000.

REFERENCES

1. Hammer E, Phong TQ, Steil L, Klingel K, Salazar MG, Bernhardt J, et al. Viral myocarditis induced by Coxsackievirus B3 in A.BY/SnJ mice: analysis of changes in the myocardial proteome. Proteomics. 2010;10(9):1802-18. doi: 10.1002/pmic.200900734. PubMed PMID: 20213679.

2. Hammer E, Goritzka M, Ameling S, Darm K, Steil L, Klingel K, et al. Characterization of the human myocardial proteome in inflammatory dilated cardiomyopathy by label-free quantitative shotgun proteomics of heart biopsies. Journal of proteome research. 2011;10(5):2161-71. doi: 10.1021/pr1008042. PubMed PMID: 21417265.
