# Supplementary figures and images for: Dynamic adaptation of myocardial proteome during heart failure development
Source: PLoS One. 2017 Oct 3;12(10):e0185915. doi: 10.1371/journal.pone.0185915 (PMC5626523; doi:10.1371/journal.pone.0185915)

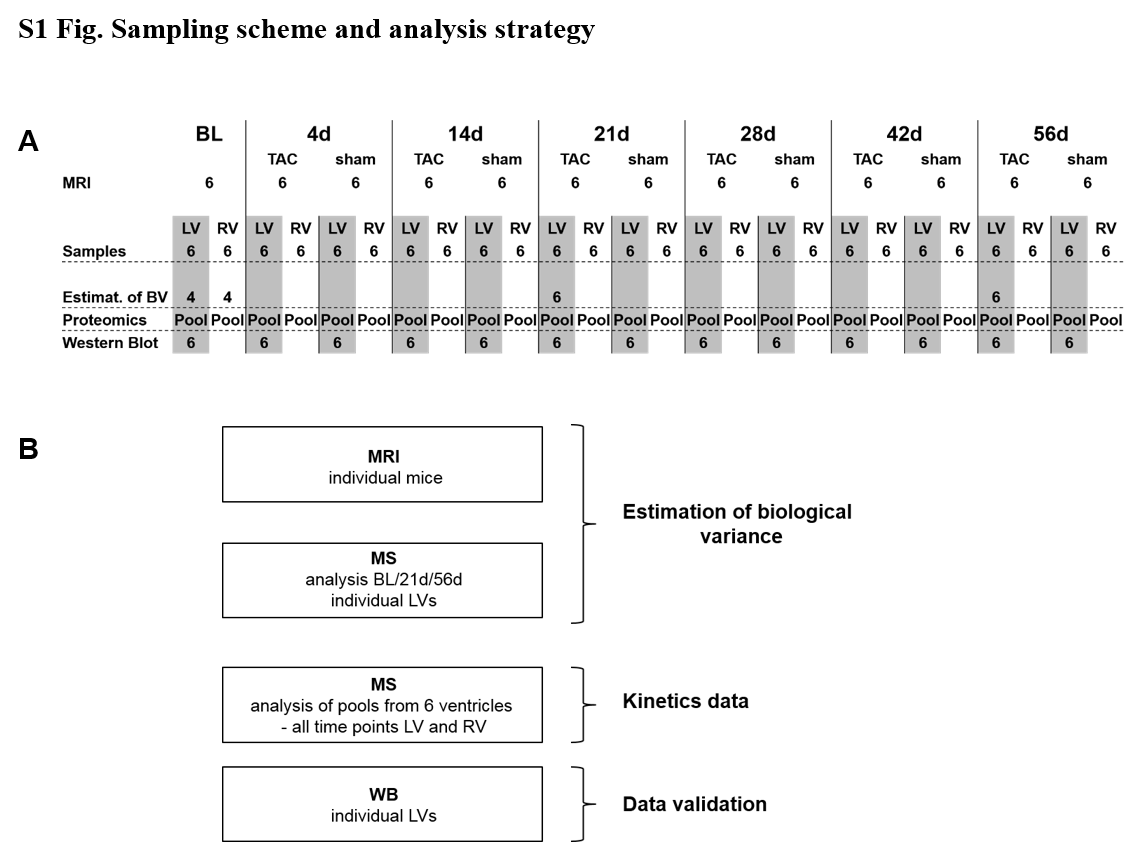

Supplement: S1 Fig — (A) Displayed are the numbers of individual samples used for MRI, estimation of biological variance (Estimat. of BV), LC-MS/MS (proteomics) and Western Blot analysis. (B) Shown is the analysis strategy. (TIF) [file pone.0185915.s002.tif]

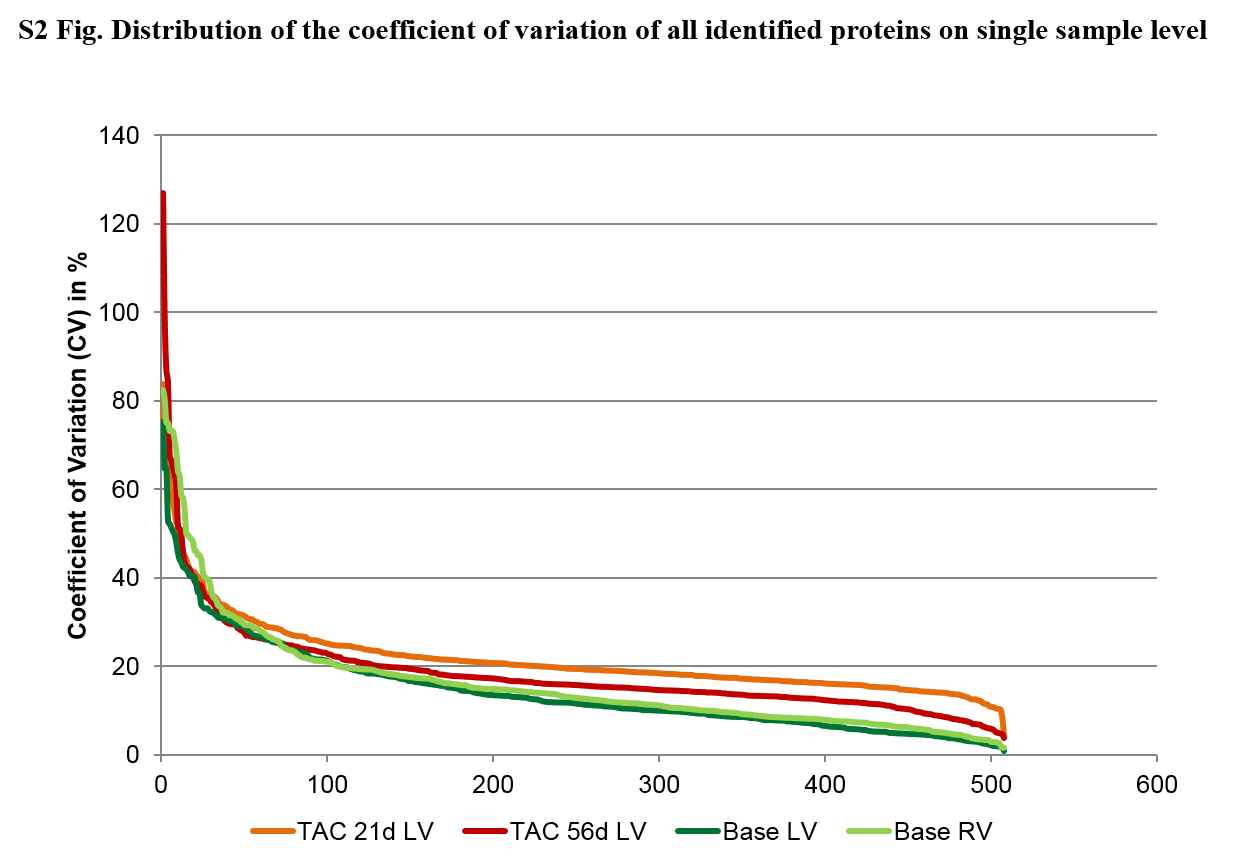

Supplement: S2 Fig — All identified proteins of the individual left ventricles of each TAC mice at day 21 and 56 (TAC21d LV, TAC 56d LV) and each left and right ventricle of baseline mice (Base LV, Base RV) are shown in consecutive numbering along the x-axis. The corresponding coefficient of variation is displayed on the y-axis. (TIF) [file pone.0185915.s003.tif]

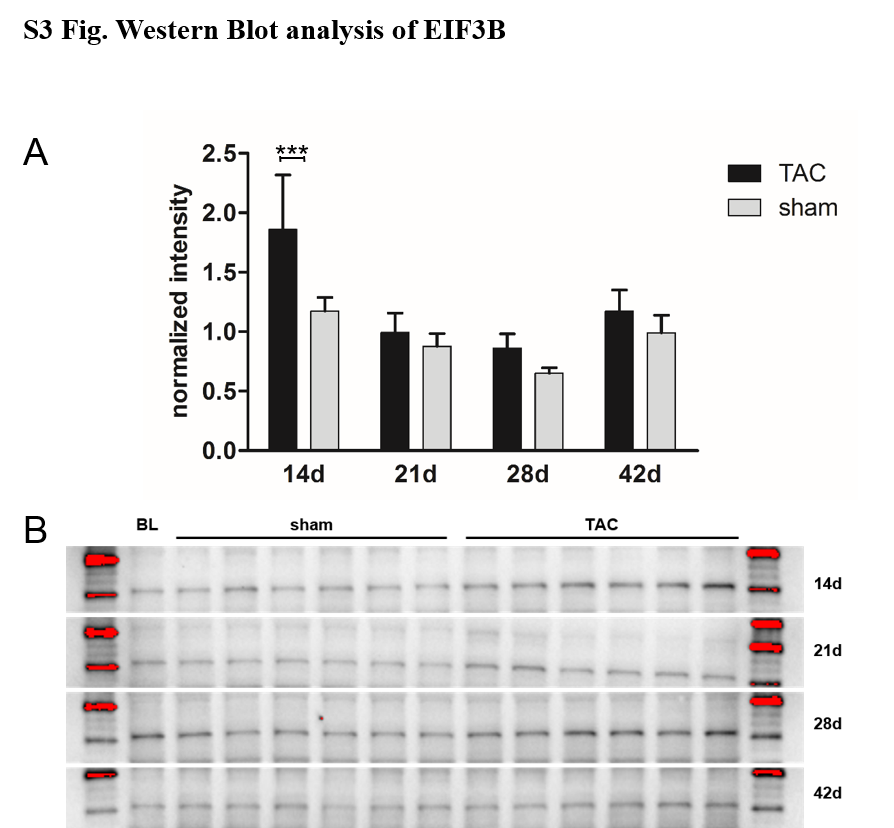

Supplement: S3 Fig — Intensities (normalized to total protein) of eukaryotic translation initiation factor 3 subunit B in tissue (EIF3B) detected by Western Blot analysis on individual sample level (n = 6 per group and time point). Especially, at day 14 EIF3B showed a significantly higher abundance in TAC compared to sham left ventricular tissue (two-way ANOVA, Bonferroni post-test, *** p<0.001). (TIF) [file pone.0185915.s004.tif]
