# Supplementary material for: Dynamic adaptation of myocardial proteome during heart failure development
Source: PLoS One. 2017 Oct 3;12(10):e0185915. doi: 10.1371/journal.pone.0185915 (PMC5626523; doi:10.1371/journal.pone.0185915)
Supplement: S1 Table — Left ventricular ejection fraction (LVEF), left ventricular mass (LVM) normalized to tibia length, left ventricular end-diastolic volume (LVEDV) and left ventricular end-systolic volume (LVESV) of baseline (BL), sham and TAC mice after 4, 14, 21, 28, 42, and 56 days after surgery. All examined cardiac parameters showed a significant deterioration of the values of TAC mice compared to their sham group (**p≤0.01, * p≤0.05; Mann-Whitney-U-Test, TAC vs. sham). (PDF) [file pone.0185915.s005.pdf]

S1 Table. Cardiac parameters of sham and TAC treated mice examined by MRI.

	LVEF (%)		LVM/Tibia length (mg/cm)		LVEDV(µl)		LVESV(µl)	
BL	56.5 ± 1.2		32.5 ± 4.3		56.7 ± 3		24.8 ±1.5	
	**sham**	**TAC**	**sham**	**TAC**	**sham**	**TAC**	**sham**	**TAC**
**4d**	54.7 ± 2	38.2 ± 4.3**	34.5 ± 3.5	43.7 ± 3.9**	56 ± 2.5	68.8 ± 4.4**	25.3 ± 2	42.7 ± 5.2**
**14d**	54.3 ± 1.4	28.3 ± 2.9**	36.6 ± 3	56.4 ± 6.7**	59.5 ± 5.3	90 ± 7**	29 ± 2.6	64.3 ±6.1**
**21d**	54.7 ± 1.2	28.2 ± 2.6**	37.8 ± 3.7	59.8 ± 6.3**	62.5 ± 6.7	91.7 ± 9.9**	28.3 ± 3	66 ± 8.9**
**28d**	55.8 ± 2.4	27.5 ± 2.2**	35.8 ± 1.6	61 ± 7.5**	58.3 ± 5.9	96 ± 5.7**	25.7 ± 2.7	69.5 ± 5.5**
**42d**	57 ± 1.3	28.3 ± 6.3**	38.5 ± 4.4	61.5 ± 6.5**	67.7 ± 1	99.3 ± 3.5**	29 ± 1	71.8 ± 15.1**
**56d**	56.2 ± 1.6	16 ± 2.2**	36.7 ± 1.8	76.4 ± 13.4**	59.3 ± 4.2	140 ± 16.2**	26.2 ± 2.5	117.7 ± 15.7**
