# Supplementary material for: Dynamic adaptation of myocardial proteome during heart failure development
Source: PLoS One. 2017 Oct 3;12(10):e0185915. doi: 10.1371/journal.pone.0185915 (PMC5626523; doi:10.1371/journal.pone.0185915)
Supplement: S3 Table — In the table all proteins, which show under basal conditions an at least two fold difference (FC) of abundance in the left ventricle when compared to the right ventricle, are listed. * Labeling as exported from Rosetta Elucidator® package. (PDF) [file pone.0185915.s007.pdf]

S3 Table. Proteins of left and right ventricle at baseline.

**Primary protein name***	**Swiss Prot ID**	**Protein name**	**FC**
NDUB5	Q9CQH3	NADH dehydrogenase [ubiquinone] 1 beta subcomplex subunit 5	2.39
ATPD	Q9D3D9	ATP synthase subunit delta	-2.04
LUM	P51885	Lumican	-2.20
MLRA	Q9QVP4	Myosin regulatory light chain 2, atrial isoform	-9.88
MYL4	P09541	Myosin light chain 4	-10.02
CAH2	P00920	Carbonic anhydrase 2	-3.07
HBA	P01942	Hemoglobin subunit alpha	-2.41
HBB1	P02088	Hemoglobin subunit beta-1	-2.23
