# Supplementary material for: Dynamic adaptation of myocardial proteome during heart failure development
Source: PLoS One. 2017 Oct 3;12(10):e0185915. doi: 10.1371/journal.pone.0185915 (PMC5626523; doi:10.1371/journal.pone.0185915)
Supplement: S4 Table — The table displays the protein alterations in the TAC group in comparison to the age matched sham group (ratios TAC/sham) in the left ventricle (LV) and/or right ventricle (RV) with an at least two fold change (≥1 peptide hits) in abundance at one or more of the examined time points day 4, 14, 21, 28, 42, and 56. Red color shows increased and green color shows decreased protein levels in TAC mice in comparison to sham animals. * Labeling as exported from Rosetta Elucidator® package. (PDF) [file pone.0185915.s008.pdf]

S 4 Table. Changes in protein abundance after transverse aortic constriction (TAC).

Primary protein name*	Swiss Prot ID	Protein name	LV 4d	LV 14d	LV 21d	LV 28d	LV 42d	LV 56d	RV 4d	RV 14d	RV 21d	RV 28d	RV 42d	RV 56d
1433T	P68254	14-3-3 protein theta	0.8	2.4	1.21	0.8	1.11	1.21	1.23	1.12	1.24	0.66	1.13	1.01
41	P48193	Protein 4.1	n.d.	n.d.	n.d.	n.d.	n.d.	n.d.	0.86	2.05	1.1	0.79	1.15	1.71
5HT1F	Q02284	5-hydroxytryptamine receptor 1F	0.69	0.16	1.17	0.99	0.81	0.46	n.d.	n.d.	n.d.	n.d.	n.d.	n.d.
6PGD	Q9DCD0	6-phosphogluconate dehydrogenase.decarboxylating	2.78	5.8	2.93	0.61	2.31	1.1	2.78	0.9	0.61	1.07	0.82	2.61
A1AG2	P07361	Alpha-1-acid glycoprotein 2	0.3	1.82	1.95	10.6	0.0	3.43	0.46	0.16	2.36	5.82	0.11	0.68
A1AT2	P22599	Alpha-1-antitrypsin 1-2	1.17	1.44	0.44	0.68	0.73	0.94	0.96	1.54	0.75	0.87	1.02	0.77
A2M	Q61838	Alpha-2-macroglobulin	1.47	0.71	0.88	2.77	0.73	0.74	0.74	0.88	1.12	1.06	0.72	0.66
AAKG1	O54950	5'-AMP-activated protein kinase subunit gamma-1	1.16	1.19	0.37	0.74	0.6	0.87	1.02	1.16	0.84	0.88	0.85	0.87
ABHD2	Q9QXM0	Abhydrolase domain-containing protein 2	0.74	3.21	1.06	1.18	0.45	0.48	0.97	1.73	0.53	1.12	1.42	1.52
ACHE	P20782	Acetylcholine receptor subunit epsilon	2.15	0.22	2.01	1.76	0.4	0.7	n.d.	n.d.	n.d.	n.d.	n.d.	n.d.
ACOT2	Q9QYR9	Acyl-coenzyme A thioesterase 2. mitochondrial	1.34	0.59	0.57	0.92	0.69	0.68	0.91	1	1.07	1.23	0.85	0.46
ACOT9	Q9R0X4	Acyl-coenzyme A thioesterase 9. mitochondrial	0.93	0.74	1.17	1.47	1.34	2.13	1.01	0.7	1.33	1.25	1.48	1.86
ACPM	Q9CR21	Acyl carrier protein. mitochondrial	1.52	0.37	2.16	1.29	0.62	0.64	0.86	1.11	1.02	0.7	1.36	0.98
ACTG	P63260	Actin. cytoplasmic 2	1.08	1.73	2.06	1.1	1.08	1.29	1.1	1.25	1.16	0.68	1.11	1.19
ACTN1	Q7TPR4	Alpha-actinin-1	0.99	1.45	0.95	1.78	1.4	2.3	1.19	1.71	2.19	1.74	1.2	2.33
ACTS	P68134	Actin. alpha skeletal muscle	1.37	6.31	4.44	4.99	5.7	5.31	0.92	1.88	2.5	2.83	2.05	5.09
ADA10	O35598	Disintegrin and metalloproteinase domain-containing protein 10	1.02	0.85	0.47	1.01	1.38	1.78	0.57	1.66	1.46	0.42	1.81	4
ADCY8	P97490	Adenylate cyclase type 8	n.d.	n.d.	n.d.	n.d.	n.d.	n.d.	1.45	0.98	1	1.52	0.74	0.41
ADHX	P28474	Alcohol dehydrogenase class-3	0.98	0.99	1.01	0.99	1.17	1.08	0.17	0.11	0.74	1.39	8.36	6.34
ADPRH	P54923	[Protein ADP-ribosylarginine] hydrolase	1.5	0.83	0.62	1.18	0.62	1.56	0.65	1.11	1.93	1.54	0.47	1.04
ADT4	Q3V132	ADP/ATP translocase 4	n.d.	n.d.	n.d.	n.d.	n.d.	n.d.	0.86	1.02	1.12	0.4	0.82	1.04
AL2S8	Q8VHI4	Amyotrophic lateral sclerosis 2 chromosomal region candidate gene 8 protein homolog	0.74	2.4	1.88	0.9	1.24	1.39	n.d.	n.d.	n.d.	n.d.	n.d.	n.d.
AL7A1	Q9DBF1	Alpha-aminoadipic semialdehyde dehydrogenase	0.78	0.84	1.17	1.32	0.91	1.28	1.08	0.69	1.67	1.51	0.44	0.46
ALK	P97793	ALK tyrosine kinase receptor	0.97	2.24	0.76	1.18	0.69	1.34	n.d.	n.d.	n.d.	n.d.	n.d.	n.d.
ALPK2	Q91ZB0	Alpha-protein kinase 2	n.d.	n.d.	n.d.	n.d.	n.d.	n.d.	0.64	1.52	1.14	2.73	0.8	0.38
AMRP	P55302	Alpha-2-macroglobulin receptor-associated protein	1.28	1.68	2.27	1.12	1.6	1.37	n.d.	n.d.	n.d.	n.d.	n.d.	n.d.

Primary protein name*	Swiss Prot ID	Protein name	LV 4d	LV 14d	LV 21d	LV 28d	LV 42d	LV 56d	RV 4d	RV 14d	RV 21d	RV 28d	RV 42d	RV 56d
AN32A	O35381	Acidic leucine-rich nuclear phosphoprotein 32 family member A	1.99	1.44	4.08	1.37	1.07	0.68	1.08	1.31	0.75	0.73	0.69	1.27
ANF	P05125	Atrial natriuretic factor	1.84	4.91	6.26	5.33	4.37	32.8	0.11	0.53	0.17	1.44	0.58	15.2
ANK1	Q02357	Ankyrin-1	1.28	0.32	1.72	2.11	0.12	0.4	0.67	1.31	0.76	0.79	1.55	0.5
ANK2	Q8C8R3	Ankyrin-2	1.07	0.4	3.11	0.71	0.83	0.86	1.14	1.12	0.44	0.65	1.36	0.91
ANKR1	Q9CR42	Ankyrin repeat domain-containing protein 1	1.06	2.57	3.09	2.24	6.41	2.6	n.d.	n.d.	n.d.	n.d.	n.d.	n.d.
ANXA1	P10107	Annexin A1	1.87	2.01	1.12	1.28	0.95	1.98	1.25	1.58	0.47	1.24	2.23	1.44
ANXA4	P97429	Annexin A4	1.22	4.06	1.36	1.36	1.79	2.07	1.25	1.49	2.01	0.54	1.38	1.27
AOFB	Q8BW75	Amine oxidase [flavin-containing] B	0.8	0.64	0.7	0.65	0.73	0.5	0.94	0.92	0.88	0.81	0.85	0.64
AP180	Q61548	Phosphatidylinositol-binding clathrin assembly protein	1.05	1.21	1.1	1.57	1.55	2.18	n.d.	n.d.	n.d.	n.d.	n.d.	n.d.
APOA4	P06728	Apolipoprotein A-IV	0.66	2.06	1.4	1.02	0.76	0.7	0.9	1.13	0.7	0.53	1.02	1.09
ARC1A	Q9R0Q6	Actin-related protein 2/3 complex subunit 1A	1.2	0.63	1.72	1.79	0.56	1.97	0.71	0.91	2.16	1.86	0.65	0.42
ARC1B	Q9WV32	Actin-related protein 2/3 complex subunit 1B	n.d.	n.d.	n.d.	n.d.	n.d.	n.d.	1.45	1.38	2.16	1.73	1.43	1.27
ARF5	P84084	ADP-ribosylation factor 5	1	3.85	1.92	1.13	1.01	2.08	n.d.	n.d.	n.d.	n.d.	n.d.	n.d.
ARHL1	Q8BGK2	[Protein ADP-ribosylarginine] hydrolase-like protein 1	1.31	1.46	3.29	1.8	1.24	1.36	0.79	0.93	1.61	1.68	1.77	1.75
ARI2	Q9Z1K6	Protein ariadne-2 homolog	0.55	0.15	1.16	1.02	1.28	0.69	n.d.	n.d.	n.d.	n.d.	n.d.	n.d.
ARL1	P61211	ADP-ribosylation factor-like protein 1	1.06	0.88	0.9	0.66	2.03	1.56	1.1	1.14	1.24	1.66	1.2	1.19
AS3MT	Q91WU5	Arsenite methyltransferase	2.17	0.51	0.5	0.95	0.59	0.94	n.d.	n.d.	n.d.	n.d.	n.d.	n.d.
ASPH	Q8BSY0	Aspartyl/asparaginyl beta-hydroxylase	1	1.16	0.65	0.79	1.04	1.17	1.4	0.83	2.98	0.79	0.65	0.85
AT11A	P98197	Probable phospholipid-transporting ATPase IH	0.69	2.07	1.82	0.7	1.49	0.92	n.d.	n.d.	n.d.	n.d.	n.d.	n.d.
AT1A2	Q6PIE5	Sodium/potassium-transporting ATPase subunit alpha-2	1.39	1.35	2.33	1.04	0.21	0.69	0.82	0.97	0.93	1.66	1.13	0.68
AT5G2	P56383	ATP synthase lipid-binding protein. mitochondrial	n.d.	n.d.	n.d.	n.d.	n.d.	n.d.	0.99	3.18	0.66	0.83	0.57	0.71
ATIF1	O35143	ATPase inhibitor. mitochondrial	n.d.	n.d.	n.d.	n.d.	n.d.	n.d.	1	0.89	1.12	0.31	1.56	0.77
ATP6	P00848	ATP synthase subunit a	1.9	0.27	2.41	1.74	0.47	0.66	0.74	0.8	0.79	0.95	1.75	0.97
ATPF2	Q91YY4	ATP synthase mitochondrial F1 complex assembly factor 2	n.d.	n.d.	n.d.	n.d.	n.d.	n.d.	0.57	0.59	2.6	2.04	1.04	0.62
ATS13	Q769J6	A disintegrin and metalloproteinase with thrombospondin motifs 13	n.d.	n.d.	n.d.	n.d.	n.d.	n.d.	0.34	0.1	0.75	2.39	5.36	2.86
AVIL	O88398	Advillin	0.84	0.49	0.16	0.54	0.26	1.1	n.d.	n.d.	n.d.	n.d.	n.d.	n.d.
B3AT	P04919	Band 3 anion transport protein	n.d.	n.d.	n.d.	n.d.	n.d.	n.d.	2.41	0.76	1.99	0.42	0.59	1.39
BAKOR	Q8CDJ3	Beclin 1-associated autophagy-related key regulator	0.23	1000	3.62	0.84	3.17	0.94	n.d.	n.d.	n.d.	n.d.	n.d.	n.d.

Primary protein name*	Swiss Prot ID	Protein name	LV 4d	LV 14d	LV 21d	LV 28d	LV 42d	LV 56d	RV 4d	RV 14d	RV 21d	RV 28d	RV 42d	RV 56d
BAP31	Q61335	B-cell receptor-associated protein 31	1.12	2.72	1.63	0.9	0.82	1.18	n.d.	n.d.	n.d.	n.d.	n.d.	n.d.
BBS10	Q9DBI2	Bardet-Biedl syndrome 10 protein homolog	1.2	2.07	2.82	0.91	1.43	1.05	n.d.	n.d.	n.d.	n.d.	n.d.	n.d.
BCL10	Q9Z0H7	B-cell lymphoma/leukemia 10	1.12	0.59	2.56	3.17	0.37	0.51	n.d.	n.d.	n.d.	n.d.	n.d.	n.d.
BDH	Q80XN0	D-beta-hydroxybutyrate dehydrogenase. mitochondrial	0.92	1.13	1.9	2.27	2.02	2	0.71	0.7	1.72	1.8	1.37	2
BLVRB	Q923D2	Flavin reductase	0.89	1.72	1.04	1.07	0.9	0.82	0.95	1.12	1.23	0.48	0.96	1.02
BPIB6	Q8BU51	BPI fold-containing family B member 6	2.29	0.28	1.43	1.74	0.86	0.84	n.d.	n.d.	n.d.	n.d.	n.d.	n.d.
BTF3	Q64152	Transcription factor BTF3	1.36	1.07	0.89	0.96	1.5	1.14	1.33	1.25	0.83	0.5	1.2	1.02
BZW2	Q91VK1	Basic leucine zipper and W2 domain-containing protein 2	0.55	0.93	0.69	0.45	1.29	1.09	0.7	0.9	0.59	1.7	0.78	0.31
C10	O35127	Protein C10	n.d.	n.d.	n.d.	n.d.	n.d.	n.d.	1.63	1.14	2.01	0.5	0.97	1.01
C1QBP	O35658	Complement component 1 Q subcomponent-binding protein. mitochondrial	4	0.31	0.76	0.73	0.22	0.37	0.72	0.92	1.08	2.74	1.45	0.61
C560	Q9CZB0	Succinate dehydrogenase cytochrome b560 subunit. mitochondrial	1.24	0.76	1.23	1.01	0.74	0.76	0.77	0.53	0.96	2.52	1.41	0.99
CAH2	P00920	Carbonic anhydrase 2	0.83	1.07	0.79	1.96	0.98	0.65	0.95	0.92	2.22	0.61	0.46	0.92
CALM	P62204	Calmodulin	2.3	0.62	0.94	1.08	0.64	0.84	0.89	1.69	0.77	2.83	1.4	0.27
CALU	O35887	Calumenin	1.38	1.58	1.54	1.39	1.65	1.97	1.55	1.32	2	0.91	1.04	1.9
CAN15	Q9JLG8	Calpain-15	2.32	0.37	1.73	1.82	0.52	0.58	n.d.	n.d.	n.d.	n.d.	n.d.	n.d.
CANB1	Q63810	Calcineurin subunit B type 1	0.5	1.2	0.95	0.77	1.49	2.8	n.d.	n.d.	n.d.	n.d.	n.d.	n.d.
CAPR1	Q60865	Caprin-1	n.d.	n.d.	n.d.	n.d.	n.d.	n.d.	1.25	1.1	0.94	1.1	3.03	0.86
CAPZB	P47757	F-actin-capping protein subunit beta	3.01	1.76	7.46	0.65	1.13	0.6	0.36	0.09	0.41	2.93	15.7	7.26
CATD	P18242	Cathepsin D	0.94	0.83	0.85	1.04	1.06	1.11	0.98	1.21	1.07	0.97	0.82	0.47
CBR4	Q91VT4	Carbonyl reductase family member 4	1.03	0.83	0.65	1.45	0.7	0.84	0.83	0.69	1.46	1.65	0.49	0.65
CC138	Q0VF22	Coiled-coil domain-containing protein 138	0.57	0.79	0.14	0.81	0.7	1.04	1.63	5.91	1.31	0.65	0.37	0.55
CC150	Q8CDI7	Coiled-coil domain-containing protein 150	n.d.	n.d.	n.d.	n.d.	n.d.	n.d.	1.55	3.04	2.58	0.39	0.2	0.17
CC154	Q6RUT8	Coiled-coil domain-containing protein 154	1.14	1.29	0.56	1.14	0.28	0.65	1.18	0.98	1.37	1.15	1.22	1.68
CC164	Q3USS3	Coiled-coil domain-containing protein 164	n.d.	n.d.	n.d.	n.d.	n.d.	n.d.	1.28	1.51	1.41	1.58	0.24	1.24
CC165	Q3UHU5	Coiled-coil domain-containing protein 165	1	1.03	0.49	0.4	0.87	1.06	n.d.	n.d.	n.d.	n.d.	n.d.	n.d.
CDC42	P60766	Cell division control protein 42 homolog	2.47	1.25	1.45	1.3	0.93	1.36	n.d.	n.d.	n.d.	n.d.	n.d.	n.d.
CDN2B	P55271	Cyclin-dependent kinase 4 inhibitor B	0.91	3.06	0.9	0.78	0.97	0.98	n.d.	n.d.	n.d.	n.d.	n.d.	n.d.
CDV3	Q4VAA2	Protein CDV3	0.93	2.82	1.34	1.13	0.95	1.68	1.28	1.41	1.42	1.02	1.19	1.38

Primary protein name*	Swiss Prot ID	Protein name	LV 4d	LV 14d	LV 21d	LV 28d	LV 42d	LV 56d	RV 4d	RV 14d	RV 21d	RV 28d	RV 42d	RV 56d
CES1D	Q8VCT4	Carboxylesterase 1D	0.75	0.36	0.3	0.55	0.47	0.3	0.93	0.92	0.92	0.65	0.76	0.47
CFAB	P04186	Complement factor B	n.d.	n.d.	n.d.	n.d.	n.d.	n.d.	0.7	1.33	0.96	2.32	0.52	0.19
CHCH2	Q9D1L0	Coiled-coil-helix-coiled-coil-helix domain-containing protein 2. mitochondrial	1.04	1.7	9.96	1.07	2.88	0.51	0.67	0.04	0.44	9.16	7.95	7.62
CHCH6	Q91VN4	Coiled-coil-helix-coiled-coil-helix domain-containing protein 6	0.37	1000	1.1	0.81	1.11	1.69	1.05	0.54	0.99	0.62	1.36	1.04
CI040	Q8VCE4	Uncharacterized protein C9orf40 homolog	n.d.	n.d.	n.d.	n.d.	n.d.	n.d.	0.72	2.1	0.98	0.47	1.19	0.63
CISD2	Q9CQB5	CDGSH iron sulfur domain-containing protein 2	2.42	1	0.58	0.72	1.29	0.97	n.d.	n.d.	n.d.	n.d.	n.d.	n.d.
CKAP4	Q8BMK4	Cytoskeleton-associated protein 4	1.98	4.2	2.04	2.57	1.6	2.68	1.9	1.66	1.62	0.58	1.44	1.81
CLIC1	Q9Z1Q5	Chloride intracellular channel protein 1	1.2	1.63	0.93	1.13	1.24	2.12	1.13	1.37	1.41	0.84	0.96	1.06
CLIC4	Q9QYB1	Chloride intracellular channel protein 4	0.67	1.79	1.26	0.83	1.78	1.56	1.1	0.88	1.16	1.24	2.07	1.65
CLN8	Q9QUK3	Protein CLN8	0.55	1.64	0.51	0.45	0.65	1.49	1.11	2.73	1.08	0.56	1.1	0.82
CLUL1	Q3ZRW6	Clusterin-like protein 1	n.d.	n.d.	n.d.	n.d.	n.d.	n.d.	0.41	0.12	0.89	1.82	2.37	3.03
CLUS	Q06890	Clusterin	1.47	2.14	1.27	1.88	1.02	3.65	1.16	1.17	1	0.7	0.8	0.92
CN159	Q8BH86	UPF0317 protein C14orf159 homolog. mitochondrial	0.96	0.57	0.6	0.79	0.54	0.41	0.72	0.84	0.71	0.77	0.65	0.49
CN159	Q8BH86	UPF0317 protein C14orf159 homolog.mitochondrial	0.96	0.57	0.6	0.79	0.54	0.41	0.72	0.84	0.71	0.77	0.65	0.49
CND2	Q8C156	Condensin complex subunit 2	0.84	1.03	2.42	1.39	0.72	0.71	0.83	0.95	1.45	2.99	1.15	0.85
CO044	Q8R3P6	UPF0464 protein C15orf44 homolog	0.68	0.49	3.56	0.27	1.38	0.6	n.d.	n.d.	n.d.	n.d.	n.d.	n.d.
CO1A2	Q01149	Collagen alpha-2(I) chain	0.59	2.38	1.57	1.14	1.21	1.65	1.31	1.16	0.97	0.46	1.12	1.14
CO3	P01027	Complement C3	1.23	0.76	0.76	1.32	1.24	0.97	0.82	0.85	1.25	1.29	0.89	0.4
CO4A1	P02463	Collagen alpha-1(IV) chain	1.2	1.78	2.66	1.09	1.01	1.19	0.74	0.44	0.92	1.59	2.26	1.49
CO6A4	A2AX52	Collagen alpha-4(VI) chain	n.d.	n.d.	n.d.	n.d.	n.d.	n.d.	0.68	0.66	1.58	1.27	0.91	0.31
CO6A6	Q8C6K9	Collagen alpha-6(VI) chain	n.d.	n.d.	n.d.	n.d.	n.d.	n.d.	1.18	1.18	1.49	1.44	1.22	0.3
COMT	O88587	Catechol O-methyltransferase	1.18	1.49	1.06	1.22	0.91	2.32	0.9	1.74	0.97	0.72	1.15	1.28
COR1C	Q9WUM4	Coronin-1C	n.d.	n.d.	n.d.	n.d.	n.d.	n.d.	1.13	1.45	2.32	1.47	0.91	1.1
COT2	P43135	COUP transcription factor 2	n.d.	n.d.	n.d.	n.d.	n.d.	n.d.	0.92	3.46	0.96	0.39	0.84	0.84
COX1	P00397	Cytochrome c oxidase subunit 1	1.19	0.38	2.82	1.26	0.73	0.73	0.73	0.81	0.78	2.82	1.33	1.06
COX20	Q9D7J4	Cytochrome c oxidase protein 20 homolog	1.09	0.67	0.88	0.45	0.73	0.81	0.92	1.28	0.89	2.04	0.76	0.47
COX3	P00416	Cytochrome c oxidase subunit 3	0.63	1.68	1.03	1.86	0.87	0.97	1.12	0.85	1.15	4.65	1.7	1.15
CP1A2	P00186	Cytochrome P450 1A2	1.1	0.72	0.96	1.32	0.49	0.95	n.d.	n.d.	n.d.	n.d.	n.d.	n.d.

Primary protein name*	Swiss Prot ID	Protein name	LV 4d	LV 14d	LV 21d	LV 28d	LV 42d	LV 56d	RV 4d	RV 14d	RV 21d	RV 28d	RV 42d	RV 56d
CP3AG	Q64481	Cytochrome P450 3A16	1.17	0.49	1.15	1.47	0.97	1.27	0.82	1.65	0.88	1	1.02	0.79
CP4AA	O88833	Cytochrome P450 4A10	1.7	0.88	0.66	1.95	0.49	0.94	1.06	1.06	1.73	0.47	1.22	0.46
CPIN1	Q8WTY4	Anamorsin	n.d.	n.d.	n.d.	n.d.	n.d.	n.d.	0.95	2.01	0.88	0.48	1.27	0.78
CQ061	Q5F285	UPF0451 protein C17orf61 homolog	0.76	0.68	0.6	0.85	0.9	1.01	0.81	0.6	1.2	2.01	0.78	0.77
CQ108	Q3UN90	UPF0631 protein C17orf108 homolog	0.52	2.72	0.97	1.16	0.75	0.95	n.d.	n.d.	n.d.	n.d.	n.d.	n.d.
CRGC	Q61597	Gamma-crystallin C	2.18	0.79	0.89	1.6	0.74	1.25	n.d.	n.d.	n.d.	n.d.	n.d.	n.d.
CSN1	Q99LD4	COP9 signalosome complex subunit 1	1.08	1.04	1.16	1.16	0.83	1.35	0.69	0.91	1.59	2.83	0.72	0.91
CSN2	P61202	COP9 signalosome complex subunit 2	0.97	2.25	0.82	0.49	1.03	1.43	0.97	1.2	0.73	0.99	0.78	0.63
CSN7A	Q9CZ04	COP9 signalosome complex subunit 7a	3.03	0.76	0.69	1.11	0.56	0.88	1.34	1.17	1.27	1.06	0.99	0.86
CSRP3	P50462	Cysteine and glycine-rich protein 3	1.33	1.58	2.08	1.63	1.39	1.83	0.74	0.9	1.42	1.53	1.93	1.94
CWC22	Q8C5N3	Pre-mRNA-splicing factor CWC22 homolog	n.d.	n.d.	n.d.	n.d.	n.d.	n.d.	1.7	1.14	8.86	1.41	0.1	0.21
CX6B1	P56391	Cytochrome c oxidase subunit 6B1	5.29	0.94	5.63	1.09	1.21	0.61	0.32	0.11	0.57	4.29	28.9	4.23
DC1I2	O88487	Cytoplasmic dynein 1 intermediate chain 2	0.84	2.9	1.67	0.97	1.18	0.81	0.82	1.07	0.88	0.53	1.76	0.92
DC1L1	Q8R1Q8	Cytoplasmic dynein 1 light intermediate chain 1	1.15	1.98	0.91	1.17	1.67	2.86	0.92	0.96	1.38	0.84	1.26	1.6
DCD2C	Q9D1B8	Doublecortin domain-containing protein 2C	1.52	0.85	0.94	1.48	2.47	1.16	n.d.	n.d.	n.d.	n.d.	n.d.	n.d.
DERM	Q9QZZ6	Dermatopontin	0.63	0.93	1.76	0.75	1.07	0.87	0.91	1.44	1.02	0.45	1.39	1.1
DHR11	Q3U0B3	Dehydrogenase/reductase SDR family member 11	n.d.	n.d.	n.d.	n.d.	n.d.	n.d.	0.93	1.36	0.99	0.67	1.13	0.44
DHRS7	Q9CXR1	Dehydrogenase/reductase SDR family member 7	0.67	1.32	0.66	1.04	0.33	0.75	n.d.	n.d.	n.d.	n.d.	n.d.	n.d.
DHSD	Q9CXV1	Succinate dehydrogenase [ubiquinone] cytochrome b small subunit. mitochondrial	0.94	0.62	1.04	1.03	0.49	0.59	1.01	0.83	1.01	0.99	1.04	1
DPEP1	P31428	Dipeptidase 1	0.79	0.65	1.04	1.4	0.89	1.07	1.04	0.77	1.28	1.63	0.45	0.52
DPYL3	Q62188	Dihydropyrimidinase-related protein 3	1.65	2.79	1.85	1.69	2.07	1.98	n.d.	n.d.	n.d.	n.d.	n.d.	n.d.
DSG2	O55111	Desmoglein-2	0.72	0.97	0.87	1.01	0.97	0.73	0.92	0.68	0.72	0.44	0.86	0.82
DUS3	Q9D7X3	Dual specificity protein phosphatase 3	0.79	1.4	0.82	0.87	1.32	1.68	4.24	0.77	0.86	1.09	0.78	1.52
DX39A	Q8VDW0	Spliceosome RNA helicase Ddx39b	n.d.	n.d.	n.d.	n.d.	n.d.	n.d.	0.81	0.92	2.57	1.05	0.59	0.71
DYH12	Q3V0Q1	Dynein heavy chain 12. axonemal	n.d.	n.d.	n.d.	n.d.	n.d.	n.d.	1000	0	0	1.95	0	1000
DYH8	Q9n.d.Q0	Dynein heavy chain 8. axonemal	1.32	0.83	2.96	2.25	0.42	1.07	n.d.	n.d.	n.d.	n.d.	n.d.	n.d.
DYL2	Q9D0M5	Dynein light chain 2. cytoplasmic	2.15	1.05	3.2	0.93	1.19	0.74	0.72	0.54	0.83	0.73	1.82	1.48
EF1A1	P10126	Elongation factor 1-alpha 1	1.63	1.48	1.62	1.32	1.19	2.03	1.07	1.12	1.09	1.58	0.65	1.24

Primary protein name*	Swiss Prot ID	Protein name	LV 4d	LV 14d	LV 21d	LV 28d	LV 42d	LV 56d	RV 4d	RV 14d	RV 21d	RV 28d	RV 42d	RV 56d
EH1L1	Q99MS7	EH domain-binding protein 1-like protein 1	1.11	2.07	1.36	2.13	0.23	0.74	n.d.	n.d.	n.d.	n.d.	n.d.	n.d.
EIF3A	P23116	Eukaryotic translation initiation factor 3 subunit A	n.d.	n.d.	n.d.	n.d.	n.d.	n.d.	0.62	0.94	1.25	0.78	0.27	0.3
EIF3B	Q8JZQ9	Eukaryotic translation initiation factor 3 subunit B	1.25	8.59	1.17	0.97	0.99	1.62	0.99	0.94	1.07	0.82	1.47	0.78
EIF3I	Q9QZD9	Eukaryotic translation initiation factor 3 subunit I	0.96	2.05	0.97	0.91	1.21	1.34	1.66	0.88	1.25	0.38	0.97	0.94
EIF3M	Q99JX4	Eukaryotic translation initiation factor 3 subunit M	n.d.	n.d.	n.d.	n.d.	n.d.	n.d.	0.72	0.96	1.23	1.76	1.81	0.37
ELOC	P83940	Transcription elongation factor B polypeptide 1	1.22	1.2	0.72	1.43	0.93	0.86	0.75	0.5	0.46	1.67	1.31	2.08
EMIL1	Q99K41	EMILIN-1	1.31	6.2	2.4	1.26	1.13	2.75	n.d.	n.d.	n.d.	n.d.	n.d.	n.d.
ENAH	Q03173	Protein enabled homolog	n.d.	n.d.	n.d.	n.d.	n.d.	n.d.	0.71	1.48	0.73	0.32	2.32	1.35
EST3A	Q63880	Carboxylesterase 3A	1.02	0.94	0.88	2.19	0.97	1.74	n.d.	n.d.	n.d.	n.d.	n.d.	n.d.
F120A	Q6A0A9	Constitutive coactivator of PPAR-gamma-like protein 1	n.d.	n.d.	n.d.	n.d.	n.d.	n.d.	1.14	1.85	1.18	2	0.73	1.97
F16P2	P70695	Fructose-1.6-bisphosphatase isozyme 2	1.18	0.41	0.48	0.33	0.36	0.4	1.18	1.13	1.06	0.88	0.75	0.53
F210A	Q8BGY7	Protein FAM210A	0.84	1.04	0.45	0.24	0.51	0.48	1.12	0.76	0.51	1.19	1.21	0.46
FA5	O88783	Coagulation factor V	1.45	0.8	0.54	0.26	0.49	0.66	n.d.	n.d.	n.d.	n.d.	n.d.	n.d.
FA50A	Q9WV03	Protein FAM50A	n.d.	n.d.	n.d.	n.d.	n.d.	n.d.	0.81	0.98	2.14	1.34	0.98	1.24
FA7	P70375	Coagulation factor VII	n.d.	n.d.	n.d.	n.d.	n.d.	n.d.	0.68	1.55	1.16	0.42	1.08	0.92
FA92B	Q3V2J0	Protein FAM92B	0.75	3.19	0.51	0.76	1.2	1.34	n.d.	n.d.	n.d.	n.d.	n.d.	n.d.
FAAA	P35505	Fumarylacetoacetase	0.84	1.44	0.57	0.33	0.7	0.52	0.96	1.02	0.92	0.66	0.82	0.51
FAHD1	Q8R0F8	Fumarylacetoacetate hydrolase domain-containing protein 1	0.9	0.77	0.8	0.84	0.44	0.66	0.86	0.89	1.13	0.79	0.68	1
FETUA	P29699	Alpha-2-HS-glycoprotein	1.42	1.39	4	0.83	1.27	0.78	0.46	0.42	0.65	2.46	5.22	1.77
FGF1	P61148	Heparin-binding growth factor 1	0.67	2.35	1.47	1.79	0.49	0.78	1.09	0.78	0.93	0.43	1.06	0.7
FHL1	P97447	Four and a half LIM domains protein 1	2.21	4.14	4.03	2.72	2.82	4.55	1.3	2.54	2.46	1.28	2.36	4.5
FHL2	O70433	Four and a half LIM domains protein 2	1.05	0.95	2.5	0.78	1.09	1.14	0.87	0.33	0.81	0.72	1.82	1.28
FHL3	Q9R059	Four and a half LIM domains protein 3	1.24	7.39	1.61	1.79	1.36	2.86	n.d.	n.d.	n.d.	n.d.	n.d.	n.d.
FHOD3	Q76LL6	FH1/FH2 domain-containing protein 3	3.1	0.37	0.69	1.36	0.23	0.63	n.d.	n.d.	n.d.	n.d.	n.d.	n.d.
FIBB	Q8K0E8	Fibrinogen beta chain	0.98	0.75	0.74	0.99	0.83	1.2	1.37	0.94	0.9	0.67	0.46	0.97
FINC	P11276	Fibronectin	1.89	1.86	1.89	2.13	0.79	2.31	2.24	1.4	2.61	1.15	0.81	0.67
FKBP8	O35465	Peptidyl-prolyl cis-trans isomerase FKBP8	0.76	1.62	0.78	0.44	0.83	0.92	n.d.	n.d.	n.d.	n.d.	n.d.	n.d.
FLNC	Q8VHX6	Filamin-C	1.73	2.13	2.1	2.11	1.95	2.78	1.28	1.65	1.72	1.89	1.4	2.44

Primary protein name*	Swiss Prot ID	Protein name	LV 4d	LV 14d	LV 21d	LV 28d	LV 42d	LV 56d	RV 4d	RV 14d	RV 21d	RV 28d	RV 42d	RV 56d
FRIH	P09528	Ferritin heavy chain	0.48	0.39	0.45	0.51	0.45	0.5	0.71	0.84	0.66	0.68	0.66	0.45
FRITZ	Q8C456	WD repeat-containing and planar cell polarity effector protein fritz homolog	1.42	0.75	0.69	2.37	0.63	1.31	n.d.	n.d.	n.d.	n.d.	n.d.	n.d.
FUS	P56959	RNA-binding protein FUS	0.65	1.14	1.36	0.39	1.59	0.95	1.12	1.17	1.04	0.83	0.95	1.02
G3BP1	P97855	Ras GTPase-activating protein-binding protein 1	1.04	1.13	1.32	1.06	0.69	1.03	1.24	0.77	0.8	2.14	1.03	1.03
G3PT	Q64467	Glyceraldehyde-3-phosphate dehydrogenase. testis-specific	2.25	0.53	0.22	1.99	0.16	0.37	n.d.	n.d.	n.d.	n.d.	n.d.	n.d.
GBB2	P62880	Guanine nucleotide-binding protein G(I)/G(S)/G(T) subunit beta-2	0.88	1.34	5.26	0.99	2.46	0.55	0.47	0.23	0.54	2.08	6.02	1.43
GBLP	P68040	Guanine nucleotide-binding protein subunit beta-2-like 1	0.75	2.11	4.27	1.26	1.52	0.82	1.09	0.99	1.24	1.68	0.97	1.49
GBP4	Q61107	Guanylate-binding protein 4	0.66	1.5	0.72	0.99	1.65	0.91	1.03	0.62	1.09	0.45	2.29	0.56
GBRR1	P56475	Gamma-aminobutyric acid receptor subunit rho-1	n.d.	n.d.	n.d.	n.d.	n.d.	n.d.	0.35	0.25	0.86	2.83	77.9	3.4
GCAB	P01864	Ig gamma-2A chain C region secreted form	0.77	0.88	1.48	3.27	0.49	0.96	0.36	0.7	2.25	4.07	0.45	0.87
GIMA4	Q99JY3	GTPase IMAP family member 4	0.92	1.25	1.01	1.48	0.96	1.5	0.8	1.22	1.24	3.86	0.65	0.73
GLCNE	Q91WG8	Bifunctional UDP-N-acetylglucosamine 2-epimerase/N-acetylmannosamine kinase	0.93	1.51	0.74	1.71	0.5	0.77	n.d.	n.d.	n.d.	n.d.	n.d.	n.d.
GLGB	Q9D6Y9	1.4-alpha-glucan-branching enzyme	1.17	0.88	0.82	2.09	0.71	1.54	n.d.	n.d.	n.d.	n.d.	n.d.	n.d.
GLNA	P15105	Glutamine synthetase	n.d.	n.d.	n.d.	n.d.	n.d.	n.d.	0.46	1.42	1.25	12.2	2.74	1.2
GLO2	Q99KB8	Hydroxyacylglutathione hydrolase. mitochondrial	0.85	2.12	1.22	1.24	1.24	1.14	1.07	1.05	1.19	0.84	1.13	1.28
GLOD4	Q9CPV4	Glyoxalase domain-containing protein 4	n.d.	n.d.	n.d.	n.d.	n.d.	n.d.	0.98	0.81	0.94	2.29	0.77	0.59
GLRX3	Q9CQM9	Glutaredoxin-3	2.53	2.25	2.87	0.6	0.84	0.46	n.d.	n.d.	n.d.	n.d.	n.d.	n.d.
GLRX5	Q80Y14	Glutaredoxin-related protein 5	0.98	1.17	10.4	1.28	1.07	0.77	0.31	0.08	0.48	1.51	7.09	4.96
GLSK	D3Z7P3	Glutaminase kidney isoform. mitochondrial	1.67	1.3	0.69	0.4	0.88	0.94	n.d.	n.d.	n.d.	n.d.	n.d.	n.d.
GLU2B	O08795	Glucosidase 2 subunit beta	0.55	9.58	3.14	0.76	1.8	1.7	n.d.	n.d.	n.d.	n.d.	n.d.	n.d.
GORS2	Q99JX3	Golgi reassembly-stacking protein 2	1.19	0.86	1.79	1.26	1.07	2.61	n.d.	n.d.	n.d.	n.d.	n.d.	n.d.
GPDM	Q64521	Glycerol-3-phosphate dehydrogenase. mitochondrial	0.89	0.49	0.47	2.15	0.45	0.64	0.87	1.1	1.34	0.62	0.8	0.31
GRB2	Q60631	Growth factor receptor-bound protein 2	1.14	0.67	1.67	1.6	2.8	1.54	0.95	1.14	1.65	1.7	0.62	0.75
GSTM7	Q80W21	Glutathione S-transferase Mu 7	0.78	0.65	0.89	0.47	0.44	0.39	0.89	0.99	0.86	1.08	0.61	0.58
H32	P84228	Histone H3.2	n.d.	n.d.	n.d.	n.d.	n.d.	n.d.	0.25	0.31	0.46	3.95	2.19	0.43
HAX1	O35387	HCLS1-associated protein X-1	n.d.	n.d.	n.d.	n.d.	n.d.	n.d.	1.21	2.29	1.27	1.15	1.14	1.03
HBA	P01942	Hemoglobin subunit alpha	1.12	0.99	0.69	2.02	0.91	0.49	1.03	1.04	1.31	0.7	0.73	1
HBB1	P02088	Hemoglobin subunit beta-1	1.13	1.04	0.71	2.29	0.88	0.59	1.11	0.86	2.02	0.71	0.53	0.94

Primary protein name*	Swiss Prot ID	Protein name	LV 4d	LV 14d	LV 21d	LV 28d	LV 42d	LV 56d	RV 4d	RV 14d	RV 21d	RV 28d	RV 42d	RV 56d
HDX	Q14B70	Highly divergent homeobox	0.4	1.29	1.23	0.44	1.31	2.72	n.d.	n.d.	n.d.	n.d.	n.d.	n.d.
HEM2	P10518	Delta-aminolevulinic acid dehydratase	1.16	0.62	0.61	0.59	0.82	0.49	1.13	0.98	0.92	0.5	0.63	0.5
HEMO	Q9n.d.72	Hemopexin	0.91	0.83	1.36	2.47	0.64	1.2	0.64	0.73	1.08	1.71	0.68	1.13
HEY2	Q9QUS4	Hairy/enhancer-of-split related with YRPW motif protein 2	n.d.	n.d.	n.d.	n.d.	n.d.	n.d.	1.27	1.57	1.14	7.64	0.12	0.23
HIG1A	Q9JLR9	HIG1 domain family member 1A	1.02	1.12	0.82	0.42	0.9	0.93	0.97	0.96	0.88	0.77	0.94	1.06
HNRPL	Q8R081	Heterogeneous nuclear ribonucleoprotein L	1.03	1.12	1.01	1.46	0.84	1.23	0.81	1.3	1.79	0.43	0.82	1.79
HOT	Q8R0N6	Hydroxyacid-oxoacid transhydrogenase. mitochondrial	1.01	0.54	0.43	1.02	0.85	0.64	1.31	0.88	1.16	0.7	0.78	0.71
HPT	Q61646	Haptoglobin	0.31	0.25	1.79	7.65	0.09	1.49	0.48	0.31	1.29	3.1	0.22	1.44
HRG	Q9ESB3	Histidine-rich glycoprotein	0.89	0.61	0.89	1.36	0.98	1.3	1.18	1.64	1.32	0.72	0.76	0.26
HSBP1	Q9CQZ1	Heat shock factor-binding protein 1	0.99	1.33	1.02	0.45	0.92	1.05	1.27	1.07	1.1	0.86	0.99	0.9
HSPB6	Q5EBG6	Heat shock protein beta-6	2.34	2.27	1.98	1.97	2.06	1.94	0.96	1.29	1.46	1.58	2.46	1.96
HSPB7	P35385	Heat shock protein beta-7	2.45	1.91	2.33	2.55	1.8	2.35	1.19	1.51	1.65	1.62	1.76	1.77
HVM51	P06330	Ig heavy chain V region AC38 205.12	n.d.	n.d.	n.d.	n.d.	n.d.	n.d.	0.58	0.46	2.36	3.38	0.61	0.84
IBP7	Q61581	Insulin-like growth factor-binding protein 7	1.02	0.5	0.56	2.14	0.46	1.01	n.d.	n.d.	n.d.	n.d.	n.d.	n.d.
ICAL	P51125	Calpastatin	0.76	2.47	1.43	1.72	0.74	0.86	n.d.	n.d.	n.d.	n.d.	n.d.	n.d.
IF4A2	P10630	Eukaryotic initiation factor 4A-II	0.83	2.53	0.62	0.54	0.98	1.04	n.d.	n.d.	n.d.	n.d.	n.d.	n.d.
IF5A1	P63242	Eukaryotic translation initiation factor 5A-1	1.19	1.48	9.32	0.56	0.6	0.74	0.72	1	1.43	2.08	0.53	0.35
IGG2B	P01867	Ig gamma-2B chain C region	0.63	1.31	1.46	2.41	0.34	1.26	0.5	0.73	1.77	2.1	0.47	1.15
IGH1M	P01869	Ig gamma-1 chain C region. membrane-bound form	0.63	0.86	4.3	3.87	0.44	1.55	0.55	0.59	1.76	2.47	0.49	1.31
IGKC	P01837	Ig kappa chain C region	0.77	1.19	3.12	1.82	0.63	1.59	0.36	0.65	2.1	3.5	0.5	1.13
ILK	O55222	Integrin-linked protein kinase	1.27	0.8	0.97	1.92	0.64	1.61	0.6	1.01	0.96	5.16	1.27	0.25
IMA4	O35343	Importin subunit alpha-4	1.66	1.19	0.38	0.99	0.65	1.54	1.39	1.24	1.14	0.91	1.02	1.9
IMB1	P70168	Importin subunit beta-1	1.02	1.18	1.31	1.24	0.89	1.42	0.97	1.05	2.48	0.7	0.92	0.51
ISC2A	P85094	Isochorismatase domain-containing protein 2A. mitochondrial	1.03	0.74	0.64	1.05	0.59	0.78	0.63	0.96	0.76	1.52	0.74	0.41
ISCU	Q9D7P6	Iron-sulfur cluster assembly enzyme ISCU. mitochondrial	2.53	1.21	4.02	0.53	1.49	0.81	n.d.	n.d.	n.d.	n.d.	n.d.	n.d.
ITA8	A2ARA8	Integrin alpha-8	n.d.	n.d.	n.d.	n.d.	n.d.	n.d.	0.81	1.02	1.2	1.27	0.81	0.42
K0753	Q6A000	Uncharacterized protein KIAA0753	2.77	0.31	1.7	0.95	0.46	0.59	n.d.	n.d.	n.d.	n.d.	n.d.	n.d.
K1324	A2AFS3	UPF0577 protein KIAA1324	1.25	0.11	0.78	1.51	0.85	0.76	n.d.	n.d.	n.d.	n.d.	n.d.	n.d.

Primary protein name*	Swiss Prot ID	Protein name	LV 4d	LV 14d	LV 21d	LV 28d	LV 42d	LV 56d	RV 4d	RV 14d	RV 21d	RV 28d	RV 42d	RV 56d
K1C13	P08730	Keratin. type I cytoskeletal 13	5.18	0.85	0.94	0.95	0.82	0.65	0.56	0.5	0.96	2.14	5.93	0.94
K1C13	P08730	Keratin. type I cytoskeletal 13	5.18	0.85	0.94	0.95	0.82	0.65	0.56	0.5	0.96	2.14	5.93	0.94
K2C1B	Q6IFZ6	Keratin. type II cytoskeletal 1b	n.d.	n.d.	n.d.	n.d.	n.d.	n.d.	14.8	1.11	1.34	1.67	0.67	1.08
K2C6A	P50446	Keratin. type II cytoskeletal 6A	1.79	1.44	23.5	0.95	1.16	1.68	n.d.	n.d.	n.d.	n.d.	n.d.	n.d.
K6PP	Q9WUA3	6-phosphofructokinase type C	1.54	1.49	1.66	1.86	2.12	2.46	n.d.	n.d.	n.d.	n.d.	n.d.	n.d.
KAD4	Q9WUR9	Adenylate kinase isoenzyme 4. mitochondrial	0.85	0.52	0.62	0.81	0.5	0.54	1.16	0.88	0.74	0.64	0.84	0.82
KAT1	Q8BTY1	Kynurenine--oxoglutarate transaminase 1	0.79	1	0.49	0.85	0.99	1.03	1.4	0.67	1.04	0.49	0.73	0.69
KCC1A	Q91YS8	Calcium/calmodulin-dependent protein kinase type 1	1.11	0.82	0.6	2.32	0.34	1.52	0.85	0.93	1.34	1.53	0.37	0.38
KCNQ4	Q9JK97	Potassium voltage-gated channel subfamily KQT member 4	n.d.	n.d.	n.d.	n.d.	n.d.	n.d.	1.29	1.04	1.25	0.35	1.05	2.54
KCY	Q9DBP5	UMP-CMP kinase	0.94	1.07	0.62	1.17	0.69	0.74	0.82	0.94	0.9	3.19	1.13	0.77
KINH	Q61768	Kinesin-1 heavy chain	1.23	1.84	1.71	1.41	2.61	1.41	n.d.	n.d.	n.d.	n.d.	n.d.	n.d.
KLD10	Q6PAR0	Kelch domain-containing protein 10	n.d.	n.d.	n.d.	n.d.	n.d.	n.d.	2.52	0.82	3.01	3.6	0.85	0.08
KLDC2	Q4G5Y1	Kelch domain-containing protein 2	0.87	0.11	1.17	1.3	0.55	0.83	n.d.	n.d.	n.d.	n.d.	n.d.	n.d.
KTN1	Q61595	Kinectin	n.d.	n.d.	n.d.	n.d.	n.d.	n.d.	1.46	1.38	1.62	1.17	0.44	0.51
KV5A3	P01635	Ig kappa chain V-V region K2 (Fragment)	1.05	1.92	2.7	3.41	1.17	2.22	0.84	2.01	2.65	2.73	0.64	1.69
L2HDH	Q91YP0	L-2-hydroxyglutarate dehydrogenase. mitochondrial	n.d.	n.d.	n.d.	n.d.	n.d.	n.d.	1.01	1.16	0.73	1.67	0.49	0.39
LAMA5	Q61001	Laminin subunit alpha-5	n.d.	n.d.	n.d.	n.d.	n.d.	n.d.	0.46	0.13	0.79	3.2	1000	10.7
LAMP1	P11438	Lysosome-associated membrane glycoprotein 1	n.d.	n.d.	n.d.	n.d.	n.d.	n.d.	1.13	0.91	0.99	0.42	1.27	0.96
LANC2	Q9JJK2	LanC-like protein 2	2.51	1.19	1.27	1.35	1.75	1.36	n.d.	n.d.	n.d.	n.d.	n.d.	n.d.
LAS1L	A2BE28	Protein LAS1 homolog	0.87	1.06	0.47	0.93	0.96	0.87	n.d.	n.d.	n.d.	n.d.	n.d.	n.d.
LDLR	P35951	Low-density lipoprotein receptor	n.d.	n.d.	n.d.	n.d.	n.d.	n.d.	1.46	1.8	1.35	1.27	0.97	2.14
LEG1	P16045	Galectin-1	1.25	1.37	2.62	1.25	1.31	1.11	1.08	0.53	1.02	0.84	1.55	1.53
LENEP	Q9WVB6	Lens epithelial cell protein LEP503	1.01	0.91	3.23	1000	0.1	0.79	n.d.	n.d.	n.d.	n.d.	n.d.	n.d.
LMNB2	P21619	Lamin-B2	n.d.	n.d.	n.d.	n.d.	n.d.	n.d.	0.96	0.77	1.16	0.12	0.83	0.46
LMOD2	Q3UHZ5	Leiomodin-2	1.34	2.2	2.54	1.34	1.77	1.25	n.d.	n.d.	n.d.	n.d.	n.d.	n.d.
LRP2	A2ARV4	Low-density lipoprotein receptor-related protein 2	n.d.	n.d.	n.d.	n.d.	n.d.	n.d.	0.76	1.02	1.19	4.23	0.91	0.72
LRRF1	Q3UZ39	Leucine-rich repeat flightless-interacting protein 2	n.d.	n.d.	n.d.	n.d.	n.d.	n.d.	6.85	0.18	0.99	0.63	1.36	1.15
LRRK1	Q3UHC2	Leucine-rich repeat serine/threonine-protein kinase 1	1.49	1.2	0.73	1.22	0.15	0.89	n.d.	n.d.	n.d.	n.d.	n.d.	n.d.

Primary protein name*	Swiss Prot ID	Protein name	LV 4d	LV 14d	LV 21d	LV 28d	LV 42d	LV 56d	RV 4d	RV 14d	RV 21d	RV 28d	RV 42d	RV 56d
MAGI3	Q9EQJ9	Membrane-associated guanylate kinase. WW and PDZ domain-containing protein 3	1.69	0.61	2.44	1.3	0.89	0.75	n.d.	n.d.	n.d.	n.d.	n.d.	n.d.
MARCS	P26645	Myristoylated alanine-rich C-kinase substrate	0.93	5.38	1.51	0.84	1.08	1.4	1.27	1.49	1.82	0.51	1.88	1.21
MAST4	Q811L6	Microtubule-associated serine/threonine-protein kinase 4	n.d.	n.d.	n.d.	n.d.	n.d.	n.d.	1.27	1.53	0.81	2.2	0.68	0.77
MCTS1	Q9DB27	Malignant T cell-amplified sequence 1	n.d.	n.d.	n.d.	n.d.	n.d.	n.d.	1.43	1.56	1.9	0.75	1.53	0.43
MEMO1	Q91VH6	Protein MEMO1	1.13	2.14	0.94	2.07	1.27	1.26	n.d.	n.d.	n.d.	n.d.	n.d.	n.d.
MFN2	Q80U63	Mitofusin-2	n.d.	n.d.	n.d.	n.d.	n.d.	n.d.	1.4	1.19	7.71	1	0.13	0.18
MIA40	Q8VEA4	Mitochondrial intermembrane space import and assembly protein 40	0.35	4.61	0.85	0.21	2.33	0.81	n.d.	n.d.	n.d.	n.d.	n.d.	n.d.
MIF	P34884	Macrophage migration inhibitory factor	n.d.	n.d.	n.d.	n.d.	n.d.	n.d.	0.55	0.27	0.66	3.07	6.84	3.71
MK14	P47811	Mitogen-activated protein kinase 14	1.02	1	0.87	1.25	1.04	0.68	0.84	0.94	0.71	0.39	1.1	0.87
MLRA	Q9QVP4	Myosin regulatory light chain 2. atrial isoform	0.16	0.52	0.3	0.75	0.9	1.1	0.32	1.42	0.24	0.95	0.27	3.84
MMAA	Q8C7H1	Methylmalonic aciduria type A homolog. mitochondrial	0.76	1.07	0.79	0.96	0.44	1.12	n.d.	n.d.	n.d.	n.d.	n.d.	n.d.
MMP21	Q8K3F2	Matrix metalloproteinase-21	1.21	1.03	0.49	0.86	0.84	0.98	n.d.	n.d.	n.d.	n.d.	n.d.	n.d.
MRCKG	Q80UW5	Serine/threonine-protein kinase MRCK gamma	n.d.	n.d.	n.d.	n.d.	n.d.	n.d.	0.82	0.89	1.69	1000	1.26	0.07
MRGA8	Q91ZC4	Mas-related G-protein coupled receptor member A8	0.56	0.73	1.4	8.6	0.17	1.06	n.d.	n.d.	n.d.	n.d.	n.d.	n.d.
MRP7	Q8R4P9	Multidrug resistance-associated protein 7	3.4	0.22	3.14	2.05	0	0.69	n.d.	n.d.	n.d.	n.d.	n.d.	n.d.
MSI2H	Q920Q6	RNA-binding protein Musashi homolog 2	1.35	0.84	1.27	1.29	0.77	1.24	0.91	0.83	0.92	0.97	0.35	0.76
MUC20	Q8BUE7	Mucin-20	n.d.	n.d.	n.d.	n.d.	n.d.	n.d.	0.68	0.41	0.3	0.77	1.77	0.94
MUCM	P01873	Ig mu chain C region membrane-bound form	1.03	1000	0.29	0.83	1.42	0.68	2.45	2	1.94	1.32	1.2	0.52
MVP	Q9EQK5	Major vault protein	1.34	1.38	1.23	0.9	1.8	2.33	1.61	1.21	1.05	0.83	1.06	1.52
MY18A	Q9JMH9	Myosin-XVIIIa	1.35	0.81	2.03	1.95	1.05	1.59	n.d.	n.d.	n.d.	n.d.	n.d.	n.d.
MYH3	P13541	Myosin-3	n.d.	n.d.	n.d.	n.d.	n.d.	n.d.	0.39	1.33	1.8	0.65	0.94	1.15
MYH7	Q91Z83	Myosin-7	1.59	5	5.21	7.11	5.27	17.1	2.3	1.4	1.76	2.38	1.72	9.5
MYL1	P05977	Myosin light chain 1/3. skeletal muscle isoform	2.01	5.36	4.07	2.64	2.91	2.85	1.43	1.49	2.13	1.35	1.37	1.71
MYL4	P09541	Myosin light chain 4	0.21	0.43	0.55	0.63	0.96	1.03	0.31	1.45	0.21	1.06	0.48	4.36
MYOTI	Q9JIF9	Myotilin	1.2	2.78	1.95	2.96	2.16	2.11	n.d.	n.d.	n.d.	n.d.	n.d.	n.d.
MYPT1	Q9DBR7	Protein phosphatase 1 regulatory subunit 12A	2.56	0.42	0.83	0.45	0.78	1.16	n.d.	n.d.	n.d.	n.d.	n.d.	n.d.
NAV1	Q8CH77	Neuron navigator 1	n.d.	n.d.	n.d.	n.d.	n.d.	n.d.	1.09	1.24	1.11	0.49	1.47	1.12

Primary protein name*	Swiss Prot ID	Protein name	LV 4d	LV 14d	LV 21d	LV 28d	LV 42d	LV 56d	RV 4d	RV 14d	RV 21d	RV 28d	RV 42d	RV 56d
NDUAB	Q9D8B4	NADH dehydrogenase [ubiquinone] 1 alpha subcomplex subunit 11	0.92	0.98	1.82	0.96	1.18	0.85	0.68	0.39	1.13	1.31	1.05	0.95
NDUB6	Q3UIU2	NADH dehydrogenase [ubiquinone] 1 beta subcomplex subunit 6	0.84	0.61	0.84	1.29	0.57	0.89	0.88	0.82	1.41	0.85	0.45	0.43
NDUF4	Q9D1H6	NADH dehydrogenase [ubiquinone] 1 alpha subcomplex assembly factor 4	0.82	0.52	0.91	1.55	0.37	0.97	0.72	0.72	0.67	1.84	0.37	0.3
NDUS5	Q99LY9	NADH dehydrogenase [ubiquinone] iron-sulfur protein 5	1.42	0.78	1.79	0.88	1.27	0.55	0.77	0.5	0.8	2.93	1.76	1.31
NDUV3	Q8BK30	NADH dehydrogenase [ubiquinone] flavoprotein 3. mitochondrial	0.52	2.28	1.14	0.59	1.04	0.69	1.05	0.82	1.23	0.47	1.22	0.85
NELL2	Q61220	Protein kinase C-binding protein NELL2	1.2	1.13	0.76	2.48	0.75	0.62	n.d.	n.d.	n.d.	n.d.	n.d.	n.d.
NENF	Q9CQ45	Neudesin	1.16	1.54	1.01	0.82	0.97	1.82	2.02	1.12	1.72	1.05	0.7	1.57
NFS1	Q9Z1J3	Cysteine desulfurase. mitochondrial	3.94	0.47	0.01	2.47	0.31	0.21	0.59	0.83	0.84	0.98	1.05	0.89
NIT1	Q8VDK1	Nitrilase homolog 1	n.d.	n.d.	n.d.	n.d.	n.d.	n.d.	0.8	0.89	1.25	2.22	0.73	0.46
NNRE	Q8K4Z3	NAD(P)H-hydrate epimerase	n.d.	n.d.	n.d.	n.d.	n.d.	n.d.	0.65	1.23	0.73	4.38	1.21	0.19
NOL3	Q9Dn.d.0	Nucleolar protein 3	0.96	2.69	2.93	0.85	1.53	0.95	n.d.	n.d.	n.d.	n.d.	n.d.	n.d.
NRAP	Q80XB4	Nebulin-related-anchoring protein	2.84	4.35	3.74	3.93	3.31	5.88	n.d.	n.d.	n.d.	n.d.	n.d.	n.d.
NS1BP	Q920Q8	Influenza virus NS1A-binding protein homolog	1.15	2.47	2.29	0.51	1.78	0.86	n.d.	n.d.	n.d.	n.d.	n.d.	n.d.
NU3M	P03899	NADH-ubiquinone oxidoreductase chain 3	4.03	1.21	5.35	0.73	1.18	0.59	0.36	0.15	0.47	2.76	18.2	5.52
NU4LM	P03903	NADH-ubiquinone oxidoreductase chain 4L	1.27	1.36	4.09	0.81	1.37	0.72	n.d.	n.d.	n.d.	n.d.	n.d.	n.d.
NU4M	P03911	NADH-ubiquinone oxidoreductase chain 4	0.97	0.95	0.68	0.47	0.81	0.7	1.01	1.03	0.83	1.19	0.93	0.84
NUCB1	Q02819	Nucleobindin-1	n.d.	n.d.	n.d.	n.d.	n.d.	n.d.	0.88	0.98	1.2	2.6	0.63	1.45
NUDT8	Q9CR24	Nucleoside diphosphate-linked moiety X motif 8. mitochondrial	0.83	0.88	0.47	0.89	0.83	0.93	1.53	1.18	1.13	0.84	0.82	1.09
NUSAP	Q9ERH4	Nucleolar and spindle-associated protein 1	n.d	1000	5.08	1000	n.d	0.18	n.d.	n.d.	n.d.	n.d.	n.d.	n.d.
OLA1	Q9CZ30	Obg-like ATPase 1	2.32	1.44	0.91	1.38	0.38	0.69	1.09	1.1	1.38	1.02	1.49	0.87
ONEC3	Q8K557	One cut domain family member 3	1.08	1.15	0.63	1.71	0.31	1.28	n.d.	n.d.	n.d.	n.d.	n.d.	n.d.
OSCAR	Q8VBT3	Osteoclast-associated immunoglobulin-like receptor	n.d.	n.d.	n.d.	n.d.	n.d.	n.d.	10.7	1.45	1.26	0.58	1.1	1.83
OTUB1	Q7TQI3	Ubiquitin thioesterase OTUB1	0.97	0.85	0.93	1.31	0.82	1.23	0.85	0.98	1.16	2.08	0.88	0.81
OXND1	Q8VE38	Oxidoreductase NAD-binding domain-containing protein 1	1.42	0.46	0.93	2.85	0.84	1.25	0.83	1.03	0.99	1.25	0.53	0.68
P20D1	Q8C165	Probable carboxypeptidase PM20D1	n.d.	n.d.	n.d.	n.d.	n.d.	n.d.	0.48	1.3	1.03	0.55	0.94	0.21
PAK3	Q61036	Serine/threonine-protein kinase PAK 3	0.91	0.83	0.79	1.35	3.12	2.02	n.d.	n.d.	n.d.	n.d.	n.d.	n.d.
PALLD	Q9ET54	Palladin	1.17	3.71	1.69	1.47	1.47	1.16	1.76	1.17	0.62	1.28	1.31	2.18
PCBP1	P60335	Poly(rC)-binding protein 1	1.19	2.16	3.75	1	1.25	0.73	n.d.	n.d.	n.d.	n.d.	n.d.	n.d.

Primary protein name*	Swiss Prot ID	Protein name	LV 4d	LV 14d	LV 21d	LV 28d	LV 42d	LV 56d	RV 4d	RV 14d	RV 21d	RV 28d	RV 42d	RV 56d
PCD19	Q80TF3	Protocadherin-19	n.d.	n.d.	n.d.	n.d.	n.d.	n.d.	0.79	1.14	0.7	0.3	3.72	1.61
PCSK5	Q04592	Proprotein convertase subtilisin/kexin type 5	n.d.	n.d.	n.d.	n.d.	n.d.	n.d.	1.24	0.99	1.49	1.38	0.36	1.65
PDIA1	P09103	Protein disulfide-isomerase	1.52	2.04	1.45	1.29	1.36	2.13	1.3	1.24	1.16	0.81	0.96	1.19
PDIA5	Q92n.d.9	Protein disulfide-isomerase A5	1.71	0.14	9.18	0.83	2.26	0.69	n.d.	n.d.	n.d.	n.d.	n.d.	n.d.
PDLI1	O70400	PDZ and LIM domain protein 1	1.01	2.32	1.59	1.7	1.87	1.65	1.23	1	1.73	1.37	1.63	2.41
PDP1	Q3UV70	[Pyruvate dehydrogenase [acetyl-transferring]]-phosphatase 1. mitochondrial	0.76	0.93	0.74	1.42	0.35	1.33	0.87	0.96	1.33	0.69	0.66	0.23
PDPR	Q7TSQ8	Pyruvate dehydrogenase phosphatase regulatory subunit. mitochondrial	1.13	0.88	0.35	1.23	1.53	0.81	n.d.	n.d.	n.d.	n.d.	n.d.	n.d.
PEA15	Q62048	Astrocytic phosphoprotein PEA-15	1.01	3.75	1.06	0.8	1.16	1.13	n.d.	n.d.	n.d.	n.d.	n.d.	n.d.
PEX19	Q8VCI5	Peroxisomal biogenesis factor 19	0.88	2.13	0.87	0.36	0.8	0.97	1.06	1.65	1.25	1.48	0.83	0.95
PGES2	Q8BWM0	Prostaglandin E synthase 2	1.11	0.63	0.94	1.07	0.95	0.92	1	1.38	1.12	3.59	0.82	0.29
PGFS	Q9DB60	Prostamide/prostaglandin F synthase	2.09	0.59	2.54	1.39	0.31	0.95	0.83	1.41	1.16	3.25	2.16	1.19
PGS1	P28653	Biglycan	1.49	1.68	2.05	2.16	1.64	2.51	1.22	1.32	1.45	1.59	0.75	1.12
PGS2	P28654	Decorin	0.9	0.7	0.45	0.69	0.24	0.93	0.99	0.9	0.72	0.78	0.78	0.7
PHLB1	Q6PDH0	Pleckstrin homology-like domain family B member 1	1.1	0.88	0.84	1.03	0.48	0.87	n.d.	n.d.	n.d.	n.d.	n.d.	n.d.
PHP14	Q9DAK9	14 kDa phosphohistidine phosphatase	1.1	0.43	1.15	0.87	0.99	0.67	0.7	1.16	0.75	1.19	1.36	0.9
PI3R5	Q5SW28	Phosphoinositide 3-kinase regulatory subunit 5	n.d.	n.d.	n.d.	n.d.	n.d.	n.d.	17.1	0.97	1.26	1.41	0.59	0.76
PIEZ2	Q8CD54	Piezo-type mechanosensitive ion channel component 2	n.d.	n.d.	n.d.	n.d.	n.d.	n.d.	2.08	2.36	1.35	1.04	0.2	0.51
PIN1	Q9QUR7	Peptidyl-prolyl cis-trans isomerase NIMA-interacting 1	0.79	1.63	1.49	1.57	1.09	1.84	1.33	0.7	1.95	2.44	0.68	0.9
PKHG3	Q4VAC9	Pleckstrin homology domain-containing family G member 3	n.d.	n.d.	n.d.	n.d.	n.d.	n.d.	0.52	0.23	0.57	1.41	6.07	2.22
PKHH2	Q8C115	Pleckstrin homology domain-containing family H member 2	0.99	1.93	1.23	1.46	1.05	0.72	1.04	1.1	1.09	0.41	1.1	0.77
PKHO1	Q9JIY0	Pleckstrin homology domain-containing family O member 1	0.85	1.18	0.5	0.65	0.76	0.88	0.89	0.79	0.89	2.05	0.66	0.76
PLIN4	O88492	Perilipin-4	0.41	1.26	0.66	1.73	1.05	0.9	0.91	1.92	1.67	0.48	0.54	0.38
PLSL	Q61233	Plastin-2	2.21	2.31	1.57	0.96	1.92	2.61	n.d.	n.d.	n.d.	n.d.	n.d.	n.d.
PLST	Q99K51	Plastin-3	0.7	2.13	4.4	1.1	1.51	1.34	1.24	1.25	1.64	1.51	0.65	1.83
PNPH	P23492	Purine nucleoside phosphorylase	n.d.	n.d.	n.d.	n.d.	n.d.	n.d.	0.87	1.53	0.98	0.46	1	1.29
POPD2	Q9ES82	Popeye domain-containing protein 2	0.82	0.37	1.09	1.8	0.67	1.23	n.d.	n.d.	n.d.	n.d.	n.d.	n.d.
POSTN	Q62009	Periostin	1.54	3.03	2.76	2.95	2.44	7.29	1.28	1.7	0.93	1.02	1.41	1.98
PP1B	P62141	Serine/threonine-protein phosphatase PP1-beta catalytic subunit	0.97	1.28	1.89	1.22	1.23	1.06	0.88	1.36	0.93	2.44	1.46	0.98

Primary protein name*	Swiss Prot ID	Protein name	LV 4d	LV 14d	LV 21d	LV 28d	LV 42d	LV 56d	RV 4d	RV 14d	RV 21d	RV 28d	RV 42d	RV 56d
PPA6	Q8BP40	Lysophosphatidic acid phosphatase type 6	1.06	1.13	0.48	0.65	0.65	0.56	n.d.	n.d.	n.d.	n.d.	n.d.	n.d.
PPT1	O88531	Palmitoyl-protein thioesterase 1	n.d.	n.d.	n.d.	n.d.	n.d.	n.d.	2.58	0.6	0.89	0.7	0.43	0.28
PREP	Q8K411	Presequence protease. mitochondrial	1.01	0.31	1.23	1.08	0.91	0.75	0.92	1.44	0.97	1.49	0.87	1.29
PROD	Q9WU79	Proline dehydrogenase. mitochondrial	0.94	0.84	1.17	0.65	0.66	0.62	0.67	1.23	0.75	2.24	0.59	0.46
PRP19	Q99KP6	Pre-mRNA-processing factor 19	n.d.	n.d.	n.d.	n.d.	n.d.	n.d.	1.4	0.9	1.84	1.53	0.64	0.47
PRPS1	Q9D7G0	Ribose-phosphate pyrophosphokinase 1	1.28	0.91	0.6	1.94	0.32	1.89	n.d.	n.d.	n.d.	n.d.	n.d.	n.d.
PRS8	P62196	26S protease regulatory subunit 8	1.33	0.41	0.61	0.91	0.95	1.29	n.d.	n.d.	n.d.	n.d.	n.d.	n.d.
PSB3	Q9R1P1	Proteasome subunit beta type-3	1.35	0.8	0.75	2.94	0.64	1.21	1.32	1.17	1.84	0.7	0.51	0.65
PSF3	Q9CY94	DNA replication complex GINS protein PSF3	n.d.	n.d.	n.d.	n.d.	n.d.	n.d.	0.59	0.96	1.27	0.26	1.39	0.92
PSMD1	Q3TXS7	26S proteasome non-ATPase regulatory subunit 1	0.89	1.03	0.92	1.79	1.17	2.54	n.d.	n.d.	n.d.	n.d.	n.d.	n.d.
PTCD3	Q14C51	Pentatricopeptide repeat-containing protein 3. mitochondrial	1.89	0.58	0.61	2.43	1.29	1.02	0.66	0.67	2.12	1.02	1.03	0.5
PTK7	Q8BKG3	Inactive tyrosine-protein kinase 7	1.01	0.43	0.91	1.69	0.86	0.94	n.d.	n.d.	n.d.	n.d.	n.d.	n.d.
PTN11	P35235	Tyrosine-protein phosphatase non-receptor type 11	0.79	3.18	1.31	0.65	1.41	0.92	1.17	0.79	1.17	0.86	1.11	1.14
PUR6	Q9DCL9	Multifunctional protein ADE2	1.03	0.91	1.06	1.55	0.95	1.14	0.95	0.72	1.6	1.42	0.4	0.74
PURB	O35295	Transcriptional activator protein Pur-beta	0.55	2.41	1.07	0.94	2.06	1.55	1.25	1.23	1.39	0.81	2.01	0.8
PUS7L	Q8CE46	Pseudouridylate synthase 7 homolog-like protein	n.d.	n.d.	n.d.	n.d.	n.d.	n.d.	1.12	1.29	0.9	0.47	0.97	1.15
PXMP2	P42925	Peroxisomal membrane protein 2	0.95	0.69	0.49	0.37	0.68	0.76	1.12	0.78	0.8	0.97	0.94	0.93
PYC	Q05920	Pyruvate carboxylase. mitochondrial	0.8	0.55	0.66	1.05	0.72	1.01	1	2.24	0.87	0.55	0.57	1.23
QCR10	Q9CPX8	Cytochrome b-c1 complex subunit 10	0.91	0.32	0.38	0.91	1.42	0.73	0.85	0.77	1.3	1.7	0.42	0.58
QCR6	P99028	Cytochrome b-c1 complex subunit 6. mitochondrial	6.5	1.12	5.53	1.03	1.36	0.54	0.4	0.15	0.79	2.7	37.9	2.87
QCR8	Q9CQ69	Cytochrome b-c1 complex subunit 8	1.62	0.89	0.44	1.01	0.5	0.61	0.87	0.78	0.86	1.86	1.28	0.93
QN1	Q6ZQ06	Protein QN1 homolog	1.14	0.97	0.4	1.14	0.65	0.64	n.d.	n.d.	n.d.	n.d.	n.d.	n.d.
RAB18	P35293	Ras-related protein Rab-18	3.36	1.66	0.62	1.26	0.75	1.14	1.28	1.22	1.4	1.11	0.38	1.95
RAB6A	P35279	Ras-related protein Rab-6A	1.32	0.85	1.27	1.48	1.01	2.21	0.81	0.51	3.1	2.97	0.67	1.24
RADI	P26043	Radixin	0.83	0.74	0.98	0.8	0.7	1.32	1	1.33	1.17	0.38	0.74	0.74
RALA	P63321	Ras-related protein Ral-B	0.95	0.46	1.37	0.94	0.85	1.45	n.d.	n.d.	n.d.	n.d.	n.d.	n.d.
RBM46	P86049	Probable RNA-binding protein 46	1.32	20.3	0.21	1.31	1.1	4.21	17.5	0.05	0.33	1.04	0.64	1.29
RBMX	Q9WV02	RNA-binding motif protein. X chromosome	n.d.	n.d.	n.d.	n.d.	n.d.	n.d.	1.42	0.78	1.47	0.25	1.16	1.06

Primary protein name*	Swiss Prot ID	Protein name	LV 4d	LV 14d	LV 21d	LV 28d	LV 42d	LV 56d	RV 4d	RV 14d	RV 21d	RV 28d	RV 42d	RV 56d
RCAN2	Q9JHG2	Calcipressin-2	0.54	0.74	0.1	0.66	0.66	1.43	n.d.	n.d.	n.d.	n.d.	n.d.	n.d.
RELN	Q60841	Reelin	0.85	0.67	0.76	1.2	0.72	0.86	1.06	0.99	0.96	0.5	0.62	0.79
RL23	P62830	60S ribosomal protein L23	1.33	1.31	2.49	0.83	1.03	1.3	n.d.	n.d.	n.d.	n.d.	n.d.	n.d.
RL39	P62892	60S ribosomal protein L39	0.84	0.55	1.01	3.7	0.48	1.39	n.d.	n.d.	n.d.	n.d.	n.d.	n.d.
RL5	P47962	60S ribosomal protein L5	1.27	1.32	0.96	0.81	1.18	0.94	0.89	1.26	0.99	2.33	1.12	1.14
RL6	P47911	60S ribosomal protein L6	1.39	1.27	0.83	1.61	0.67	0.5	1.04	0.78	1.67	0.99	0.72	0.55
RL7	P14148	60S ribosomal protein L7	n.d.	n.d.	n.d.	n.d.	n.d.	n.d.	0.91	0.86	0.84	2.06	0.73	0.9
RM23	O35972	39S ribosomal protein L23. mitochondrial	1.44	0.67	0.73	0.66	0.48	0.9	1.1	0.91	1.06	0.99	0.7	0.64
RM49	Q9CQ40	39S ribosomal protein L49. mitochondrial	3.38	1.21	0.6	2.29	1.69	1.1	n.d.	n.d.	n.d.	n.d.	n.d.	n.d.
ROA1	P49312	Heterogeneous nuclear ribonucleoprotein A1	0.55	8.8	1.17	1.18	1.72	1.4	0.93	1.24	1.47	1.24	0.92	0.62
RRAS2	P62071	Ras-related protein R-Ras2	1	1.36	1.39	1.57	0.99	1.84	0.93	1.22	1.63	2.36	1.3	1.35
RS10	P63325	40S ribosomal protein S10	1.42	0.67	0.85	2.05	0.63	1.39	0.92	0.9	1.45	0.79	0.88	0.53
RS15	P62843	40S ribosomal protein S15	4.6	0.78	0.9	0.91	0.41	0.98	1.27	1.35	0.99	3.1	1.73	0.6
RS20	P60867	40S ribosomal protein S20	2.12	0.9	0.45	0.84	0.7	0.81	n.d.	n.d.	n.d.	n.d.	n.d.	n.d.
RS21	Q9CQR2	40S ribosomal protein S21	2.31	1.3	5.05	0.76	1.36	0.83	n.d.	n.d.	n.d.	n.d.	n.d.	n.d.
RS3A	P97351	40S ribosomal protein S3a	1.48	1.13	2.06	1.24	0.87	0.86	0.77	0.42	0.82	1.11	2.56	2.08
RS3A	P97351	40S ribosomal protein S3a	1.48	1.13	2.06	1.24	0.87	0.86	0.77	0.42	0.82	1.11	2.56	2.08
RSH6A	Q8CDR2	Radial spoke head protein 6 homolog A	0.89	3.07	0.86	1.2	0.94	0.72	1.43	0.93	1.14	0.79	0.73	1.64
RT22	Q9CXW2	28S ribosomal protein S22. mitochondrial	n.d.	n.d.	n.d.	n.d.	n.d.	n.d.	0.96	2.88	0.91	0.47	0.85	0.72
RT23	Q8VE22	28S ribosomal protein S23. mitochondrial	0.56	2.01	1.31	0.65	0.97	0.92	0.79	1.02	0.77	0.61	1.34	1.03
RT27	Q8BK72	28S ribosomal protein S27. mitochondrial	0.53	0.57	0.94	0.81	0.44	1.24	n.d.	n.d.	n.d.	n.d.	n.d.	n.d.
RTN4	Q99P72	Reticulon-4	2.66	3	2.27	3.04	1.66	4.77	n.d.	n.d.	n.d.	n.d.	n.d.	n.d.
RYR1	E9PZQ0	Ryanodine receptor 1	2.17	1.46	0.56	1.55	0.71	1.56	n.d.	n.d.	n.d.	n.d.	n.d.	n.d.
S10AB	P50543	Protein S100-A11	1.91	2.35	1.78	1.96	1.12	2.08	1.11	1.61	1.58	1	1	1.39
S23A3	Q60850	Solute carrier family 23 member 3	n.d.	n.d.	n.d.	n.d.	n.d.	n.d.	1.16	2.75	0.97	1.65	0.98	0.98
S2542	Q8R0Y8	Solute carrier family 25 member 42	1.43	0.66	0.6	0.79	1.11	0.74	0.74	0.87	1.51	0.78	0.52	0.44
SAA1	P05366	Serum amyloid A-1 protein	0.5	1000	5.65	1000	0	0.93	0.63	1.28	2.45	0.47	1.32	2.13
SAP	Q61207	Sulfated glycoprotein 1	1.29	0.87	1.55	0.95	1.13	0.77	1	0.77	0.93	0.79	2.64	1.08

Primary protein name*	Swiss Prot ID	Protein name	LV 4d	LV 14d	LV 21d	LV 28d	LV 42d	LV 56d	RV 4d	RV 14d	RV 21d	RV 28d	RV 42d	RV 56d
SASH1	P59808	SAM and SH3 domain-containing protein 1	0.87	6.58	1.32	1.36	1.02	0.87	1.28	0.71	1.05	0.61	1.68	0.97
SBP1	P17563	Selenium-binding protein 1	0.67	0.76	0.76	0.69	0.8	0.62	0.93	0.46	0.81	0.52	1.12	0.81
SC23B	Q9D662	Protein transport protein Sec23B	n.d.	n.d.	n.d.	n.d.	n.d.	n.d.	0.35	0.63	2.11	1.13	0.28	2.65
SC31A	Q3UPL0	Protein transport protein Sec31A	0.85	7.66	1.22	1.96	1.07	1.66	n.d.	n.d.	n.d.	n.d.	n.d.	n.d.
SDK2	Q6V4S5	Protein sidekick-2	1.69	1.52	1.23	2.1	1.76	1.93	n.d.	n.d.	n.d.	n.d.	n.d.	n.d.
Sep 02	P42208	Septin-2	1.23	0.99	1.03	2.24	0.8	1.53	n.d.	n.d.	n.d.	n.d.	n.d.	n.d.
SERPH	P19324	Serpin H1	1.75	2.07	1.32	1.11	1.31	1.83	1.4	1.36	1.31	1.19	1.04	1.37
SIAT9	O88829	Lactosylceramide alpha-2.3-sialyltransferase	0.45	0.64	0.06	0.78	0.41	2.11	n.d.	n.d.	n.d.	n.d.	n.d.	n.d.
SIR3	Q8R104	NAD-dependent protein deacetylase sirtuin-3	1.98	0.96	0.43	0.42	0.12	0.9	1.09	2.16	1.09	2.7	0.69	0.55
SLIK5	Q810B7	SLIT and NTRK-like protein 5	0.98	1.43	0.43	1.25	0.71	2.23	n.d.	n.d.	n.d.	n.d.	n.d.	n.d.
SLIRP	Q9D8T7	SRA stem-loop-interacting RNA-binding protein. mitochondrial	0.92	1.03	0.83	0.8	0.91	0.49	n.d.	n.d.	n.d.	n.d.	n.d.	n.d.
SLMAP	Q3URD3	Sarcolemmal membrane-associated protein	1.08	1.73	2	1.51	1.54	2.05	1.3	1.71	2.23	1.64	1.77	2.5
SMD1	P62315	Small nuclear ribonucleoprotein Sm D1	1.22	2.55	0.69	0.45	0.84	1.14	n.d.	n.d.	n.d.	n.d.	n.d.	n.d.
SPRL1	P70663	SPARC-like protein 1	n.d.	n.d.	n.d.	n.d.	n.d.	n.d.	1.28	0.77	0.75	0.44	1.5	1.04
SPT6H	Q62383	Transcription elongation factor SPT6	0.53	1.68	0.37	0.61	0.78	1.12	0.71	2.62	0.98	0.57	0.56	1.28
SPTCS	Q3UHA3	Spatacsin	n.d.	n.d.	n.d.	n.d.	n.d.	n.d.	1.83	0.46	0.87	0.02	3.9	1.02
SQRD	Q9R112	Sulfide:quinone oxidoreductase. mitochondrial	0.94	1.48	1.26	0.73	0.81	0.37	1.14	1.1	0.61	0.73	0.96	0.73
SRBS2	Q3UTJ2	Sorbin and SH3 domain-containing protein 2	1.31	2.21	1.89	1.82	1.72	2.13	1.1	1.48	1.55	1.9	1.83	1.96
SRRM4	Q8BKA3	Serine/arginine repetitive matrix protein 4	0.64	1.49	2.24	1.04	1.17	0.84	n.d.	n.d.	n.d.	n.d.	n.d.	n.d.
SSPN	Q62147	Sarcospan	0.45	1	2.29	0.63	1.25	0.78	1.37	0.7	0.95	0.4	1.33	1.58
STAG3	O70576	Cohesin subunit SA-3	0.93	1.07	0.3	0.46	1.45	1.13	n.d.	n.d.	n.d.	n.d.	n.d.	n.d.
STAR8	Q8K031	StAR-related lipid transfer protein 8	1.23	0.49	1.91	1.46	2.06	0.89	n.d.	n.d.	n.d.	n.d.	n.d.	n.d.
STAT3	P42227	Signal transducer and activator of transcription 3	2.03	1.51	1.34	1.06	1.4	2.24	n.d.	n.d.	n.d.	n.d.	n.d.	n.d.
STRAP	Q9Z1Z2	Serine-threonine kinase receptor-associated protein	1.3	0.77	0.99	1.98	0.42	1.31	n.d.	n.d.	n.d.	n.d.	n.d.	n.d.
STT3A	P46978	Dolichyl-diphosphooligosaccharide--protein glycosyltransferase subunit STT3A	4.71	1.14	3.68	0.87	1.2	0.48	n.d.	n.d.	n.d.	n.d.	n.d.	n.d.
SYK	Q99MN1	Lysyl-tRNA synthetase	0.75	0.72	0.92	2.52	0.55	1.29	0.86	1.02	1.26	1.1	0.63	0.85
SYNC	Q8BP47	Asparaginyl-tRNA synthetase. cytoplasmic	1.76	1.2	1.05	1.85	1.32	2.29	n.d.	n.d.	n.d.	n.d.	n.d.	n.d.
SYNE1	Q6ZWR6	Nesprin-1	0.92	1.2	3.18	4.33	0.27	0.41	1.25	1.01	1.09	0.57	1.03	2.7

Primary protein name*	Swiss Prot ID	Protein name	LV 4d	LV 14d	LV 21d	LV 28d	LV 42d	LV 56d	RV 4d	RV 14d	RV 21d	RV 28d	RV 42d	RV 56d
SYNPO	Q8CC35	Synaptopodin	0.83	0.72	0.88	1.15	1.85	1.95	0.8	1.21	1.89	1.42	1.14	2.11
SYP2L	Q8BWB1	Synaptopodin 2-like protein	1.92	3.67	3.47	2.11	2.79	3.41	1.44	2.01	1.53	1.68	1.98	2.99
SYPL1	O09117	Synaptophysin-like protein 1	0.67	2.29	1.37	0.81	1.33	1.21	1.03	0.92	0.88	0.97	1.13	1.17
SYSM	Q9JJL8	Seryl-tRNA synthetase. mitochondrial	0.54	2.03	1.1	0.64	0.87	0.81	0.76	0.9	0.87	0.77	1.19	0.74
SYTM	Q3UQ84	Threonyl-tRNA synthetase. mitochondrial	n.d.	n.d.	n.d.	n.d.	n.d.	n.d.	0.84	0.86	0.36	1.89	0.64	0.43
SYYM	Q8BYL4	Tyrosine--tRNA ligase. mitochondrial	1.12	0.97	0.78	1.69	1.39	1.89	0.82	3.11	1.17	0.91	1.69	0.84
SZT2	A2A9C3	Protein SZT2	0.79	0.14	0.67	0.68	1.56	0.69	n.d.	n.d.	n.d.	n.d.	n.d.	n.d.
T126A	Q9D8Y1	Transmembrane protein 126A	1	0.64	0.28	0.34	0.73	0.61	0.88	1.15	0.73	1.08	0.75	0.82
TAB3	Q571K4	Mitogen-activated protein kinase kinase kinase 7-interacting protein 2	0.49	6.4	0.51	0.44	1.51	1.26	n.d.	n.d.	n.d.	n.d.	n.d.	n.d.
TACO1	Q8K0Z7	Translational activator of cytochrome c oxidase 1	0.71	0.83	3.42	1.09	1.21	0.88	0.73	0.93	1.12	1.5	0.99	0.64
TAU	P10637	Microtubule-associated protein tau	1.31	2.09	1.73	1.13	1.64	0.99	0.9	1.53	0.89	1.67	0.85	0.81
TB10C	Q8C9V1	Carabin	0.85	0.87	0.37	0.24	0.44	0.65	n.d.	n.d.	n.d.	n.d.	n.d.	n.d.
TBB2A	Q7TMM9	Tubulin beta-2A chain	1.45	2.77	2.54	1.92	1.68	3.45	1.35	1.65	1.47	0.92	1.64	2.29
TCAM1	Q80UF7	TIR domain-containing adapter molecule 1	n.d.	n.d.	n.d.	n.d.	n.d.	n.d.	0.69	2.01	1.43	1	1.12	0.98
TECR	Q9CY27	Trans-2.3-enoyl-CoA reductase	1.63	0.69	0.47	0.58	0.28	0.63	1.07	1.52	1.15	1	0.92	1.55
TENN	Q80Z71	Tenascin-N	1.32	0.73	2.18	2.61	0.62	0.59	n.d.	n.d.	n.d.	n.d.	n.d.	n.d.
TFR1	Q62351	Transferrin receptor protein 1	1.61	2.36	1.76	1.57	1.29	1.02	1.35	0.9	1.14	0.63	1.13	2.8
THIOM	P97493	Thioredoxin. mitochondrial	1.24	0.99	6.44	1.01	1.02	0.62	n.d.	n.d.	n.d.	n.d.	n.d.	n.d.
TIM	Q9Rn.d.4	Protein timeless homolog	0.2	1.42	4.91	0.25	2.68	0.92	n.d.	n.d.	n.d.	n.d.	n.d.	n.d.
TIM16	Q9CQV1	Mitochondrial import inner membrane translocase subunit Tim16	0.63	2.19	1.12	0.6	1.65	1.12	1.01	0.94	0.74	0.78	1.12	1.61
TM143	Q8VD26	Transmembrane protein 143	1.14	0.53	0.71	1.28	0.29	0.73	0.72	0.66	1.09	1.16	0.94	0.81
TM9S1	Q9DBU0	Transmembrane 9 superfamily member 1	n.d.	n.d.	n.d.	n.d.	n.d.	n.d.	1.29	1.58	1.68	0.82	0.08	1.77
TMCC2	Q80W04	Transmembrane and coiled-coil domains protein 2	0.63	0.2	0.3	0.74	1.28	0.67	n.d.	n.d.	n.d.	n.d.	n.d.	n.d.
TMEDA	Q9D1D4	Transmembrane emp24 domain-containing protein 10	1.99	0.34	2.24	3.63	0.32	0.75	1.02	1.16	1.26	0.83	1.38	0.68
TMM80	Q9D3H0	Transmembrane protein 80	1.49	2.38	4.08	0.68	0.86	0.85	n.d.	n.d.	n.d.	n.d.	n.d.	n.d.
TMTC3	Q8BRH0	Transmembrane and TPR repeat-containing protein 3	1.26	0.73	0.57	1.19	0.39	0.7	1	0.92	0.91	0.65	0.8	0.68
TNI3K	Q5GIG6	Serine/threonine-protein kinase TNNI3K	1.02	2.96	0.52	0.49	0.62	0.87	n.d.	n.d.	n.d.	n.d.	n.d.	n.d.
TOM70	Q9CZW5	Mitochondrial import receptor subunit TOM70	0.76	1.17	1.04	1.05	0.9	1.58	0.67	1.45	0.81	0.67	3.01	0.71

Primary protein name*	Swiss Prot ID	Protein name	LV 4d	LV 14d	LV 21d	LV 28d	LV 42d	LV 56d	RV 4d	RV 14d	RV 21d	RV 28d	RV 42d	RV 56d
TPD54	Q9CYZ2	Tumor protein D54	0.95	0.87	1.09	1.98	1.02	1.4	0.99	1.07	1.28	2.13	0.57	0.47
TPM2	P58774	Tropomyosin beta chain	0.72	2.19	1.57	0.95	1.43	1.64	1.1	1.26	1.23	0.76	1.65	1.08
TPP1	O89023	Tripeptidyl-peptidase 1	0.68	0.88	0.48	0.93	0.75	0.69	0.65	1.37	0.95	1.19	1.65	0.64
TR104	Q7M723	Taste receptor type 2 member 104	0.85	0.54	0.43	0.76	1.01	0.91	n.d.	n.d.	n.d.	n.d.	n.d.	n.d.
TRI60	Q8VI40	Tripartite motif-containing protein 60	0.6	2.31	1.42	0.88	1.23	1.9	n.d.	n.d.	n.d.	n.d.	n.d.	n.d.
TRUB1	Q8C0D0	Probable tRNA pseudouridine synthase 1	0.7	2.13	1.35	1.2	1.01	1.14	1.13	0.94	0.92	0.87	1.32	1.56
TRXR1	Q9JMH6	Thioredoxin reductase 1. cytoplasmic	0.64	4.43	2.09	1.75	0.51	1.83	n.d.	n.d.	n.d.	n.d.	n.d.	n.d.
TSP1	P35441	Thrombospondin-1	n.d.	n.d.	n.d.	n.d.	n.d.	n.d.	1.41	0.85	0.77	0.17	0.74	0.9
TXLNB	Q8VBT1	Beta-taxilin	1.15	1.24	1.11	0.94	1.3	1.33	0.31	0.43	0.47	1.56	32.3	1.97
TXNL1	Q8CDN6	Thioredoxin-like protein 1	0.73	2.13	1.45	0.93	1.25	1.16	0.95	1.09	0.98	0.71	0.96	1.25
UB2V1	Q9CZY3	Ubiquitin-conjugating enzyme E2 variant 1	0.48	1.04	1.23	0.65	1.61	1	0.78	1.03	1.05	1.25	1.38	1.14
UBC9	P63280	SUMO-conjugating enzyme UBC9	n.d.	n.d.	n.d.	n.d.	n.d.	n.d.	1.05	1.33	0.8	1.38	0.69	0.46
UBP2L	Q80X50	Ubiquitin-associated protein 2-like	1.74	0.54	1.35	1000	1.55	2.42	n.d.	n.d.	n.d.	n.d.	n.d.	n.d.
UBP5	P56399	Ubiquitin carboxyl-terminal hydrolase 5	0.94	0.88	0.82	1.2	0.94	1.12	0.83	1.21	1.03	2.1	0.97	0.5
UCHL1	Q9R0P9	Ubiquitin carboxyl-terminal hydrolase isozyme L1	1.39	2.43	1.78	1.18	1.51	1.7	0.98	0.9	1.28	0.83	1.27	1.5
UGGG1	Q6P5E4	UDP-glucose:glycoprotein glucosyltransferase 1	n.d.	n.d.	n.d.	n.d.	n.d.	n.d.	1.88	1.3	1.76	1.89	0.73	0.21
UN13B	Q9Z1N9	Protein unc-13 homolog B	n.d.	n.d.	n.d.	n.d.	n.d.	n.d.	0.45	0.11	0.34	1.8	1.89	0.61
VAMP3	P63024	Vesicle-associated membrane protein 3	1.07	2.4	0.68	0.97	0.97	1.14	n.d.	n.d.	n.d.	n.d.	n.d.	n.d.
VATA	P50516	V-type proton ATPase catalytic subunit A	1.1	1.04	0.8	3.84	0.58	2.13	n.d.	n.d.	n.d.	n.d.	n.d.	n.d.
VKIND	Q0KK55	Protein very KIND	1	0.86	0.33	0.94	0.37	1.13	n.d.	n.d.	n.d.	n.d.	n.d.	n.d.
VMA5A	Q99KC8	von Willebrand factor A domain-containing protein 5A	n.d.	n.d.	n.d.	n.d.	n.d.	n.d.	0.97	1.63	2.02	2.09	0.6	0.48
VP13C	Q8BX70	Vacuolar protein sorting-associated protein 13C	n.d.	n.d.	n.d.	n.d.	n.d.	n.d.	0.6	1.09	0.72	0.3	2.41	1.02
VSIG8	Q6P3A4	V-set and immunoglobulin domain-containing protein 8	n.d.	n.d.	n.d.	n.d.	n.d.	n.d.	1.46	1.01	1.09	0.34	1.17	0.88
VWC2	Q8C8N3	Brorin	n.d.	n.d.	n.d.	n.d.	n.d.	n.d.	1.55	2.4	1.64	0.67	0.43	0.68
WDR1	O88342	WD repeat-containing protein 1	1.2	1.61	2.2	1.33	1.37	1.1	0.92	1.33	0.99	0.61	1.46	1.31
WNK3	Q80XP9	Serine/threonine-protein kinase WNK3	9.13	0.18	2.48	0.75	0.92	0.46	n.d.	n.d.	n.d.	n.d.	n.d.	n.d.
X3CL1	O35188	Fractalkine	n.d.	n.d.	n.d.	n.d.	n.d.	n.d.	0.7	1.44	0.48	0.63	1.28	1.32
XDH	Q00519	Xanthine dehydrogenase/oxidase	1.21	0.7	0.39	0.61	0.41	1.53	1.07	1.15	1.11	0.77	0.69	0.8

Primary protein name*	Swiss Prot ID	Protein name	LV 4d	LV 14d	LV 21d	LV 28d	LV 42d	LV 56d	RV 4d	RV 14d	RV 21d	RV 28d	RV 42d	RV 56d
XIRP1	O70373	Xin actin-binding repeat-containing protein 1	1.94	3.17	2.99	2.13	3.22	3.81	1.27	1.71	1.61	1.1	1.32	2.45
XIRP2	Q4U4S6	Xin actin-binding repeat-containing protein 2	2.7	3.98	2.9	3.04	2.97	5.93	n.d.	n.d.	n.d.	n.d.	n.d.	n.d.
XPO7	Q9EPK7	Exportin-7	2.32	12.3	0.92	1.47	1.23	0.15	n.d.	n.d.	n.d.	n.d.	n.d.	n.d.
XPP1	Q6P1B1	Xaa-Pro aminopeptidase 1	0.6	2.11	1.16	0.53	1.62	1.11	n.d.	n.d.	n.d.	n.d.	n.d.	n.d.
