# Supplementary material for: Dynamic adaptation of myocardial proteome during heart failure development
Source: PLoS One. 2017 Oct 3;12(10):e0185915. doi: 10.1371/journal.pone.0185915 (PMC5626523; doi:10.1371/journal.pone.0185915)
Supplement: S5 Table — The table displays the ratios (TAC/sham) of proteins which showed altered abundance in both ventricles. * Labeling as exported from Rosetta Elucidator® package. (PDF) [file pone.0185915.s009.pdf]

S5 Table. Proteins altered in LV and RV after transverse aortic constriction (TAC).

Primary protein name*	Swiss Prot ID	Protein name	LV 4d	LV 14d	LV 21d	LV 28d	LV 42d	LV 56d	RV 4d	RV 14d	RV 21d	RV 28d	RV 42d	RV 56d
**6PGD**	Q9DCD0	6-phosphogluconate dehydrogenase,decarboxylating	2.78	5.80	2.93	0.61	2.31	1.10	2.78	0.90	0.61	1.07	0.82	2.61
**A1AG2**	P07361	Alpha-1-acid glycoprotein 2	0.30	1.82	1.95	10.6	0.00	3.43	0.46	0.16	2.36	5.82	0.11	0.68
**ACTN1**	Q7TPR4	Alpha-actinin-1	0.99	1.45	0.95	1.78	1.40	2.30	1.19	1.71	2.19	1.74	1.20	2.33
**ACTS**	P68134	Actin, alpha skeletal muscle	1.37	6.31	4.44	4.99	5.70	5.31	0.92	1.88	2.50	2.83	2.05	5.09
**ADA10**	O35598	Disintegrin and metalloproteinase domain-containing protein 10	1.02	0.85	0.47	1.01	1.38	1.78	0.57	1.66	1.46	0.42	1.81	4.00
**ANF**	P05125	Atrial natriuretic factor	1.84	4.91	6.26	5.33	4.37	32.8	0.11	0.53	0.17	1.44	0.58	15.2
**ANK1**	Q02357	Ankyrin-1	1.28	0.32	1.72	2.11	0.12	0.40	0.67	1.31	0.76	0.79	1.55	0.50
**ANK2**	Q8C8R3	Ankyrin-2	1.07	0.40	3.11	0.71	0.83	0.86	1.14	1.12	0.44	0.65	1.36	0.91
**ANXA1**	P10107	Annexin A1	1.87	2.01	1.12	1.28	0.95	1.98	1.25	1.58	0.47	1.24	2.23	1.44
**ANXA4**	P97429	Annexin A4	1.22	4.06	1.36	1.36	1.79	2.07	1.25	1.49	2.01	0.54	1.38	1.27
**BZW2**	Q91VK1	Basic leucine zipper and W2 domain-containing protein 2	0.55	0.93	0.69	0.45	1.29	1.09	0.70	0.90	0.59	1.70	0.78	0.31
**C1QBP**	O35658	Complement component 1 Q subcomponent-binding protein. mitochondrial	4.00	0.31	0.76	0.73	0.22	0.37	0.72	0.92	1.08	2.74	1.45	0.61
**CALM**	P62204	Calmodulin	2.30	0.62	0.94	1.08	0.64	0.84	0.89	1.69	0.77	2.83	1.40	0.27
**CAPZB**	P47757	F-actin-capping protein subunit beta	3.01	1.76	7.46	0.65	1.13	0.60	0.36	0.09	0.41	2.93	15.7	7.26
**CC138**	Q0VF22	Coiled-coil domain-containing protein 138	0.57	0.79	0.14	0.81	0.70	1.04	1.63	5.91	1.31	0.65	0.37	0.55
**CES1D**	Q8VCT4	Carboxylesterase 1D	0.75	0.36	0.30	0.55	0.47	0.30	0.93	0.92	0.92	0.65	0.76	0.47
**CHCH2**	Q9D1L0	Coiled-coil-helix-coiled-coil-helix domain-containing protein 2, mitochondrial	1.04	1.70	9.96	1.07	2.88	0.51	0.67	0.04	0.44	9.16	7.95	7.62
**CLN8**	Q9QUK3	Protein CLN8	0.55	1.64	0.51	0.45	0.65	1.49	1.11	2.73	1.08	0.56	1.10	0.82
**CN159**	Q8BH86	UPF0317 protein C14orf159 homolog,mitochondrial	0.96	0.57	0.60	0.79	0.54	0.41	0.72	0.84	0.71	0.77	0.65	0.49
**CND2**	Q8C156	Condensin complex subunit 2	0.84	1.03	2.42	1.39	0.72	0.71	0.83	0.95	1.45	2.99	1.15	0.85

Primary protein name*	Swiss Prot ID	Protein name	LV 4d	LV 14d	LV 21d	LV 28d	LV 42d	LV 56d	RV 4d	RV 14d	RV 21d	RV 28d	RV 42d	RV 56d
**CO1A2**	Q01149	Collagen alpha-2(I) chain	0.59	2.38	1.57	1.14	1.21	1.65	1.31	1.16	0.97	0.46	1.12	1.14
**CO4A1**	P02463	Collagen alpha-1(IV) chain	1.20	1.78	2.66	1.09	1.01	1.19	0.74	0.44	0.92	1.59	2.26	1.49
**COX1**	P00397	Cytochrome c oxidase subunit 1	1.19	0.38	2.82	1.26	0.73	0.73	0.73	0.81	0.78	2.82	1.33	1.06
**COX20**	Q9D7J4	Cytochrome c oxidase protein 20 homolog	1.09	0.67	0.88	0.45	0.73	0.81	0.92	1.28	0.89	2.04	0.76	0.47
**CP4AA**	O88833	Cytochrome P450 4A10	1.70	0.88	0.66	1.95	0.49	0.94	1.06	1.06	1.73	0.47	1.22	0.46
**CX6B1**	P56391	Cytochrome c oxidase subunit 6B1	5.29	0.94	5.63	1.09	1.21	0.61	0.32	0.11	0.57	4.29	28.9	4.23
**EIF3I**	Q9QZD9	Eukaryotic translation initiation factor 3 subunit I	0.96	2.05	0.97	0.91	1.21	1.34	1.66	0.88	1.25	0.38	0.97	0.94
**F210A**	Q8BGY7	Protein FAM210A	0.84	1.04	0.45	0.24	0.51	0.48	1.12	0.76	0.51	1.19	1.21	0.46
**FETUA**	P29699	Alpha-2-HS-glycoprotein	1.42	1.39	4.00	0.83	1.27	0.78	0.46	0.42	0.65	2.46	5.22	1.77
**FGF1**	P61148	Heparin-binding growth factor 1	0.67	2.35	1.47	1.79	0.49	0.78	1.09	0.78	0.93	0.43	1.06	0.70
**FHL1**	P97447	Four and a half LIM domains protein 1	2.21	4.14	4.03	2.72	2.82	4.55	1.30	2.54	2.46	1.28	2.36	4.50
**FHL2**	O70433	Four and a half LIM domains protein 2	1.05	0.95	2.50	0.78	1.09	1.14	0.87	0.33	0.81	0.72	1.82	1.28
**FINC**	P11276	Fibronectin	1.89	1.86	1.89	2.13	0.79	2.31	2.24	1.40	2.61	1.15	0.81	0.67
**FLNC**	Q8VHX6	Filamin-C	1.73	2.13	2.10	2.11	1.95	2.78	1.28	1.65	1.72	1.89	1.40	2.44
**FRIH**	P09528	Ferritin heavy chain	0.48	0.39	0.45	0.51	0.45	0.50	0.71	0.84	0.66	0.68	0.66	0.45
**GBB2**	P62880	Guanine nucleotide-binding protein G(I)/G(S)/G(T) subunit beta-2	0.88	1.34	5.26	0.99	2.46	0.55	0.47	0.23	0.54	2.08	6.02	1.43
**GCAB**	P01864	Ig gamma-2A chain C region secreted form	0.77	0.88	1.48	3.27	0.49	0.96	0.36	0.70	2.25	4.07	0.45	0.87
**GLRX5**	Q80Y14	Glutaredoxin-related protein 5	0.98	1.17	10.4	1.28	1.07	0.77	0.31	0.08	0.48	1.51	7.09	4.96
**GPDM**	Q64521	Glycerol-3-phosphate dehydrogenase, mitochondrial	0.89	0.49	0.47	2.15	0.45	0.64	0.87	1.10	1.34	0.62	0.80	0.31
**HBB1**	P02088	Hemoglobin subunit beta-1	1.13	1.04	0.71	2.29	0.88	0.59	1.11	0.86	2.02	0.71	0.53	0.94
**HPT**	Q61646	Haptoglobin	0.31	0.25	1.79	7.65	0.09	1.49	0.48	0.31	1.29	3.10	0.22	1.44
**HSPB6**	Q5EBG6	Heat shock protein beta-6	2.34	2.27	1.98	1.97	2.06	1.94	0.96	1.29	1.46	1.58	2.46	1.96
**IF5A1**	P63242	Eukaryotic translation initiation factor 5A-1	1.19	1.48	9.32	0.56	0.60	0.74	0.72	1.00	1.43	2.08	0.53	0.35
**IGG2B**	P01867	Ig gamma-2B chain C region	0.63	1.31	1.46	2.41	0.34	1.26	0.50	0.73	1.77	2.10	0.47	1.15

Primary protein name*	Swiss Prot ID	Protein name	LV 4d	LV 14d	LV 21d	LV 28d	LV 42d	LV 56d	RV 4d	RV 14d	RV 21d	RV 28d	RV 42d	RV 56d
**IGH1M**	P01869	Ig gamma-1 chain C region, membrane-bound form	0.63	0.86	4.30	3.87	0.44	1.55	0.55	0.59	1.76	2.47	0.49	1.31
**IGKC**	P01837	Ig kappa chain C region	0.77	1.19	3.12	1.82	0.63	1.59	0.36	0.65	2.10	3.50	0.50	1.13
**KAT1**	Q8BTY1	Kynurenine--oxoglutarate transaminase 1	0.79	1.00	0.49	0.85	0.99	1.03	1.40	0.67	1.04	0.49	0.73	0.69
**KCC1A**	Q91YS8	Calcium/calmodulin-dependent protein kinase type 1	1.11	0.82	0.60	2.32	0.34	1.52	0.85	0.93	1.34	1.53	0.37	0.38
**KV5A3**	P01635	Ig kappa chain V-V region K2 (Fragment)	1.05	1.92	2.70	3.41	1.17	2.22	0.84	2.01	2.65	2.73	0.64	1.69
**MLRA**	Q9QVP4	Myosin regulatory light chain 2, atrial isoform	0.16	0.52	0.30	0.75	0.90	1.10	0.32	1.42	0.24	0.95	0.27	3.84
**MUCM**	P01873	Ig mu chain C region membrane-bound form	1.03	1000	0.29	0.83	1.42	0.68	2.45	2.00	1.94	1.32	1.20	0.52
**MYH7**	Q91Z83	Myosin-7	1.59	5.00	5.21	7.11	5.27	17.2	2.30	1.40	1.76	2.38	1.72	9.50
**MYL1**	P05977	Myosin light chain 1/3, skeletal muscle isoform	2.01	5.36	4.07	2.64	2.91	2.85	1.43	1.49	2.13	1.35	1.37	1.71
**MYL4**	P09541	Myosin light chain 4	0.21	0.43	0.55	0.63	0.96	1.03	0.31	1.45	0.21	1.06	0.48	4.36
**NDUF4**	Q9D1H6	NADH dehydrogenase [ubiquinone] 1 alpha subcomplex assembly factor 4	0.82	0.52	0.91	1.55	0.37	0.97	0.72	0.72	0.67	1.84	0.37	0.30
**NDUV3**	Q8BK30	NADH dehydrogenase [ubiquinone] flavoprotein 3. mitochondrial	0.52	2.28	1.14	0.59	1.04	0.69	1.05	0.82	1.23	0.47	1.22	0.85
**NU3M**	P03899	NADH-ubiquinone oxidoreductase chain 3	4.03	1.21	5.35	0.73	1.18	0.59	0.36	0.15	0.47	2.76	18.3	5.52
**PALLD**	Q9ET54	Palladin	1.17	3.71	1.69	1.47	1.47	1.16	1.76	1.17	0.62	1.28	1.31	2.18
**PDLI1**	O70400	PDZ and LIM domain protein 1	1.01	2.32	1.59	1.70	1.87	1.65	1.23	1.00	1.73	1.37	1.63	2.41
**PDP1**	Q3UV70	[Pyruvate dehydrogenase [acetyl-transferring]]-phosphatase 1. mitochondrial	0.76	0.93	0.74	1.42	0.35	1.33	0.87	0.96	1.33	0.69	0.66	0.23
**PGFS**	Q9DB60	Prostamide/prostaglandin F synthase	2.09	0.59	2.54	1.39	0.31	0.95	0.83	1.41	1.16	3.25	2.16	1.19
**PKHO1**	Q9JIY0	Pleckstrin homology domain-containing family O member 1	0.85	1.18	0.50	0.65	0.76	0.88	0.89	0.79	0.89	2.05	0.66	0.76
**PLIN4**	O88492	Perilipin-4	0.41	1.26	0.66	1.73	1.05	0.90	0.91	1.92	1.67	0.48	0.54	0.38
**PTCD3**	Q14C51	Pentatricopeptide repeat-containing protein 3, mitochondrial	1.89	0.58	0.61	2.43	1.29	1.02	0.66	0.67	2.12	1.02	1.03	0.50
**PURB**	O35295	Transcriptional activator protein Pur-beta	0.55	2.41	1.07	0.94	2.06	1.55	1.25	1.23	1.39	0.81	2.01	0.80
**QCR10**	Q9CPX8	Cytochrome b-c1 complex subunit 10	0.91	0.32	0.38	0.91	1.42	0.73	0.85	0.77	1.30	1.70	0.42	0.58
**QCR6**	P99028	Cytochrome b-c1 complex subunit 6, mitochondrial	6.50	1.12	5.53	1.03	1.36	0.54	0.40	0.15	0.79	2.70	37.9	2.87
**RAB18**	P35293	Ras-related protein Rab-18	3.36	1.66	0.62	1.26	0.75	1.14	1.28	1.22	1.40	1.11	0.38	1.95

Primary protein name*	Swiss Prot ID	Protein name	LV 4d	LV 14d	LV 21d	LV 28d	LV 42d	LV 56d	RV 4d	RV 14d	RV 21d	RV 28d	RV 42d	RV 56d
**RAB6A**	P35279	Ras-related protein Rab-6A	1.32	0.85	1.27	1.48	1.01	2.21	0.81	0.51	3.10	2.97	0.67	1.24
**RBM46**	P86049	Probable RNA-binding protein 46	1.32	20.3	0.21	1.31	1.10	4.21	17.5	0.05	0.33	1.04	0.64	1.29
**RS15**	P62843	40S ribosomal protein S15	4.60	0.78	0.90	0.91	0.41	0.98	1.27	1.35	0.99	3.10	1.73	0.60
**RS3A**	P97351	40S ribosomal protein S3a	1.48	1.13	2.06	1.24	0.87	0.86	0.77	0.42	0.82	1.11	2.56	2.08
**SAA1**	P05366	Serum amyloid A-1 protein	0.50	1000	5.65	1000	0.00	0.93	0.63	1.28	2.45	0.47	1.32	2.13
**SIR3**	Q8R104	NAD-dependent protein deacetylase sirtuin-3	1.98	0.96	0.43	0.42	0.12	0.90	1.09	2.16	1.09	2.70	0.69	0.55
**SLMAP**	Q3URD3	Sarcolemmal membrane-associated protein	1.08	1.73	2.00	1.51	1.54	2.05	1.30	1.71	2.23	1.64	1.77	2.50
**SPT6H**	Q62383	Transcription elongation factor SPT6	0.53	1.68	0.37	0.61	0.78	1.12	0.71	2.62	0.98	0.57	0.56	1.28
**SSPN**	Q62147	Sarcospan	0.45	1.00	2.29	0.63	1.25	0.78	1.37	0.70	0.95	0.40	1.33	1.58
**SYNE1**	Q6ZWR6	Nesprin-1	0.92	1.20	3.18	4.33	0.27	0.41	1.25	1.01	1.09	0.57	1.03	2.70
**SYP2L**	Q8BWB1	Synaptopodin 2-like protein	1.92	3.67	3.47	2.11	2.79	3.41	1.44	2.01	1.53	1.68	1.98	2.99
**TBB2A**	Q7TMM9	Tubulin beta-2A chain	1.45	2.77	2.54	1.92	1.68	3.45	1.35	1.65	1.47	0.92	1.64	2.29
**TFR1**	Q62351	Transferrin receptor protein 1	1.61	2.36	1.76	1.57	1.29	1.02	1.35	0.90	1.14	0.63	1.13	2.80
**XIRP1**	O70373	Xin actin-binding repeat-containing protein 1	1.94	3.17	2.99	2.13	3.22	3.81	1.27	1.71	1.61	1.10	1.32	2.45
