# Supplementary material for: Dynamic adaptation of myocardial proteome during heart failure development
Source: PLoS One. 2017 Oct 3;12(10):e0185915. doi: 10.1371/journal.pone.0185915 (PMC5626523; doi:10.1371/journal.pone.0185915)
Supplement: S6 Table — The table shows the subset of proteins, which was assigned to the IPA z-Score categories proliferation of cells, necrosis, and apoptosis. * Labeling as exported from Rosetta Elucidator® package. (PDF) [file pone.0185915.s010.pdf]

S6 Table. Proteins of IPA activity z-score analysis.

Primary protein name*	Protein name	FC (TAC/ sham) 14d	FC (TAC/ sham) 21d	FC (TAC/ sham) 28d	FC (TAC/ sham) 42d	proliferation of cells	necrosis	apoptosis
**ALK**	anaplastic lymphoma receptor tyrosine kinase	2.24	6.44	-1.53	2.03	x	x	x
**ANXA1**	annexin A1	2.01	-2.33	1.32	-2.06	x	x	x
**ARF4**	ADP-ribosylation factor 4	2.22	2.20	-1.98	2.47			x
**ARIH2**	ariadne RBR E3 ubiquitin protein ligase 2	-6.58	-2.56	-1.63	-3.07	x		x
**BCAP31**	B-cell receptor-associated protein 31	2.72	-1.97	-2.22	2.80	x		x
**CAST**	calpastatin	2.47	1.43	1.72	-1.35	x	x	x
**CDKN2B**	cyclin-dependent kinase inhibitor 2B (p15, inhibits CDK4)	3.06	-1.84	-3.89	-6.54	x		
**COL1A2**	collagen. type I. alpha 2	2.38	-1.60	2.37	-3.27	x		
**COPS2**	COP9 (constitutive photomorphogenic) homolog. subunit 2	2.25	-1.44	-2.49	-2.10	x		
**DPYSL3**	dihydropyrimidinase-like 3	2.79	1.85	2.47	-2.36	x	x	
**EIF3B**	eukaryotic translation initiation factor 3, subunit B	8.59	-1.77	-2.21	-2.55	x	x	x
**EIF3I**	eukaryotic translation initiation factor 3, subunit I	2.05	1.46	2.41	-2.95	x	x	x
**FGF1**	fibroblast growth factor 1 (acidic)	2.35	1.47	2.48	-2.05	x	x	x
**GNB2L1**	guanine nucleotide binding protein (G protein). beta polypeptide 2-like 1	2.11	4.27	-2.14	1.52	x	x	x
**IGFBP7**	insulin-like growth factor binding protein 7	-2.02	-1.78	2.14	-2.19	x	x	x
**IVNS1ABP**	influenza virus NS1A binding protein	2.47	2.29	-1.95	1.78	x	x	x
**MAPT**	microtubule-associated protein tau	2.09	1.73	1.13	1.64	x	x	x
**MEMO1**	mediator of cell motility 1	2.14	-1.07	2.07	1.27	x		
**MT-ATP6**	ATP synthase F0 subunit 6	-3.69	2.41	2.77	-2.28	x		
**NDUFAB1**	NADH dehydrogenase (ubiquinone) 1. alpha/beta subcomplex. 1. 8kDa	-2.70	2.16	2.24	-3.58	x	x	
**NOL3**	nucleolar protein 3 (apoptosis repressor with CARD domain)	2.69	2.93	-1.18	1.53	x	x	x
**PALLD**	palladin. cytoskeletal associated protein	3.71	1.69	1.47	1.47	x	x	x
**PAM16**	presequence translocase-associated motor 16 homolog	2.19	1.12	-1.66	1.65			x
**PDIA5**	protein disulfide isomerase family A. member 5	-7.30	9.18	-1.21	2.26	x		
**PEA15**	phosphoprotein enriched in astrocytes 15	3.75	1.06	-1.25	1.16	x	x	x
**PLS3**	plastin 3	2.13	4.40	1.10	1.51	x		
**PRKCSH**	protein kinase C substrate 80K-H	9.58	3.14	2.43	1.80	x		
**PSMC5**	proteasome (prosome. macropain) 26S subunit. ATPase. 5	-2.45	-1.65	-1.10	-1.06	x		
**PTPN11**	protein tyrosine phosphatase. non-receptor type 11	3.18	1.31	-1.53	1.41	x	x	x

Primary protein name*	Protein name	FC (TAC/ sham) 14d	FC (TAC/ sham) 21d	FC (TAC/ sham) 28d	FC (TAC/ sham) 42d	proliferation of cells	necrosis	apoptosis
**RALA**	v-ral simian leukemia viral oncogene homolog A (ras related)	-2.17	1.37	-1.07	-1.18	x		
**SERPINH1**	serpin peptidase inhibitor. clade H (heat shock protein 47). member 1	2.07	1.32	1.11	1.31	x	x	x
**TFRC**	transferrin receptor	2.36	1.76	1.57	1.29	x	x	x
**TMED10**	transmembrane emp24-like trafficking protein 10	-2.99	2.24	3.63	-3.11		x	x
**Tpm2**	tropomyosin 2. beta	2.19	1.57	-1.06	1.43	x		
**TXNL1**	thioredoxin-like 1	2.13	1.45	-1.08	1.25	x		
**TXNRD1**	thioredoxin reductase 1	4.43	2.09	1.75	-1.95	x	x	x
**UCHL1**	ubiquitin carboxyl-terminal esterase L1 (ubiquitin thiolesterase)	2.43	1.78	1.18	1.51	x	x	x
**YWHAQ**	tyrosine 3-monooxygenase/tryptophan 5-monooxygenase activation protein. theta	2.40	-2.67	-4.18	-2.26	x	x	x
