# Supplementary material for: Dynamic adaptation of myocardial proteome during heart failure development
Source: PLoS One. 2017 Oct 3;12(10):e0185915. doi: 10.1371/journal.pone.0185915 (PMC5626523; doi:10.1371/journal.pone.0185915)
Supplement: S2 Table — (PDF) [file pone.0185915.s006.pdf]

S2 Table. Proteins identified in the left ventricle (A) and the right ventricle (B)

S2 Table A Details on all identified proteins in samples of the left ventricle

Swiss-Prot ID	Primary Protein Name	Protein Description	Peptide count	Sample count	ProteinTeller Probability	Intensity [base]	Intensity [SHAM 4d]	Intensity [SHAM 14d]
P58771	TPM1_MOUSE	Tropomyosin alpha-1 chain OS=Mus musculus GN=Tpm1 PE=1 SV=1 [MASS=32680]	5	13	1	4.77E+07	5.44E+07	5.18E+07
P58281	OPA1_MOUSE	Dynamin-like 120 kDa protein, mitochondrial OS=Mus musculus GN=Opa1 PE=1 SV=1 [MASS=111338]	10	13	1	9.92E+05	9.62E+05	1.07E+06
P63038	CH60_MOUSE	60 kDa heat shock protein, mitochondrial OS=Mus musculus GN=Hspd1 PE=1 SV=1 [MASS=60955]	19	13	1	2.25E+07	2.27E+07	1.95E+07
Q8VEM8	MPCP_MOUSE	Phosphate carrier protein, mitochondrial OS=Mus musculus GN=Slc25a3 PE=1 SV=1 [MASS=39632]	12	13	1	2.20E+07	1.94E+07	2.08E+07
O70468	MYPC3_MOUSE	Myosin-binding protein C, cardiac-type OS=Mus musculus GN=Mybpc3 PE=2 SV=1 [MASS=140631]	44	13	1	3.82E+07	4.00E+07	4.17E+07
Q9D855	QCR7_MOUSE	Cytochrome b-c1 complex subunit 7 OS=Mus musculus GN=Uqcrb PE=1 SV=3 [MASS=13527]	6	13	1	6.63E+06	7.79E+06	7.33E+06
Q9CQZ5	NDUA6_MOUSE	NADH dehydrogenase [ubiquinone] 1 alpha subcomplex subunit 6 OS=Mus musculus GN=Ndufa6 PE=1 SV=1 [MASS=15283]	5	13	1	2.64E+06	3.45E+06	3.93E+06
Q9Z0X1	AIFM1_MOUSE	Apoptosis-inducing factor 1, mitochondrial OS=Mus musculus GN=Aifm1 PE=1 SV=1 [MASS=66765]	12	13	1	2.93E+06	2.80E+06	2.07E+06
P14206	RSSA_MOUSE	40S ribosomal protein SA OS=Mus musculus GN=Rpsa PE=1 SV=4 [MASS=32838]	6	13	1	5.32E+05	5.72E+05	6.58E+05
Q99JY0	ECHB_MOUSE	Trifunctional enzyme subunit beta, mitochondrial OS=Mus musculus GN=Hadhb PE=1 SV=1 [MASS=51386]	16	13	1	3.41E+07	3.31E+07	3.62E+07
Q91VD9	NDUS1_MOUSE	NADH-ubiquinone oxidoreductase 75 kDa subunit, mitochondrial OS=Mus musculus GN=Ndufs1 PE=1 SV=1 [MASS=79748]	27	13	1	2.62E+07	2.35E+07	2.46E+07
P47738	ALDH2_MOUSE	Aldehyde dehydrogenase, mitochondrial OS=Mus musculus GN=Aldh2 PE=1 SV=1 [MASS=56537]	10	13	1	3.24E+06	3.38E+06	3.16E+06
P56480	ATPB_MOUSE	ATP synthase subunit beta, mitochondrial OS=Mus musculus GN=Atp5b PE=1 SV=2 [MASS=56300]	27	13	1	2.58E+08	3.04E+08	2.88E+08
P48962	ADT1_MOUSE	ADP/ATP translocase 1 OS=Mus musculus GN=Slc25a4 PE=1 SV=4 [MASS=32904]	7	13	1	3.24E+07	3.49E+07	5.63E+07
P63017	HSP7C_MOUSE	Heat shock cognate 71 kDa protein OS=Mus musculus GN=Hspa8 PE=1 SV=1 [MASS=70871]	12	13	1	8.82E+06	9.44E+06	8.40E+06
O35459	ECH1_MOUSE	Delta(3,5)-Delta(2,4)-dienoyl-CoA isomerase, mitochondrial OS=Mus musculus GN=Ech1 PE=2 SV=1 [MASS=36118]	12	13	1	8.61E+06	7.49E+06	6.20E+06
Q02566	MYH6_MOUSE	Myosin-6 OS=Mus musculus GN=Myh6 PE=1 SV=2 [MASS=223564]	25	13	1	2.41E+08	2.46E+08	2.13E+08
P54071	IDHP_MOUSE	Isocitrate dehydrogenase [NADP], mitochondrial OS=Mus musculus GN=Idh2 PE=1 SV=3 [MASS=50906]	19	13	1	4.69E+07	4.98E+07	6.28E+07
P62900	RL31_MOUSE	60S ribosomal protein L31 OS=Mus musculus GN=Rpl31 PE=2 SV=1 [MASS=14463]	1	12	0.99	1.06E+05	1.00E+05	1.16E+05
P16858	G3P_MOUSE	Glyceraldehyde-3-phosphate dehydrogenase OS=Mus musculus GN=Gapdh PE=1 SV=2 [MASS=35810]	9	13	1	2.83E+07	2.93E+07	3.04E+07
Q91YT0	NDUV1_MOUSE	NADH dehydrogenase [ubiquinone] flavoprotein 1, mitochondrial OS=Mus musculus GN=Ndufv1 PE=1 SV=1 [MASS=50834]	14	13	1	6.36E+06	6.04E+06	9.13E+06
P01887	B2MG_MOUSE	Beta-2-microglobulin OS=Mus musculus GN=B2m PE=1 SV=1 [MASS=13823]	1	7	0.97	7.74E+04	6.39E+04	5.44E+04
Q9CR61	NDUB7_MOUSE	NADH dehydrogenase [ubiquinone] 1 beta subcomplex subunit 7 OS=Mus musculus GN=Ndufb7 PE=1 SV=3 [MASS=16331]	4	13	1	1.33E+06	1.26E+06	9.90E+05
P47911	RL6_MOUSE	60S ribosomal protein L6 OS=Mus musculus GN=Rpl6 PE=1 SV=3 [MASS=33509]	1	2	1	2.75E+04	2.24E+04	1.17E+04
Q9D0K2	SCOT1_MOUSE	Succinyl-CoA:3-ketoacid-coenzyme A transferase 1, mitochondrial OS=Mus musculus GN=Oxct1 PE=1 SV=1 [MASS=55988]	13	13	1	1.75E+07	1.71E+07	1.31E+07
Q7TQ48	SRCA_MOUSE	Sarcalumenin OS=Mus musculus GN=Srl PE=1 SV=1 [MASS=99183]	27	13	1	2.57E+07	2.64E+07	2.65E+07
Q8CAQ8	IMMT_MOUSE	Mitochondrial inner membrane protein OS=Mus musculus GN=Immt PE=1 SV=1 [MASS=83899]	23	13	1	1.15E+07	1.15E+07	8.94E+06
Q01853	TERA_MOUSE	Transitional endoplasmic reticulum ATPase OS=Mus musculus GN=Vcp PE=1 SV=4 [MASS=89321]	15	13	1	1.83E+06	1.62E+06	2.02E+06
P17710	HXK1_MOUSE	Hexokinase-1 OS=Mus musculus GN=Hk1 PE=1 SV=2 [MASS=108302]	6	13	1	8.92E+05	6.99E+05	6.36E+05
Q99LC5	ETFA_MOUSE	Electron transfer flavoprotein subunit alpha, mitochondrial OS=Mus musculus GN=Etfa PE=1 SV=2 [MASS=35009]	14	13	1	3.63E+07	4.01E+07	3.64E+07
Q8K1Z0	COQ9_MOUSE	Ubiquinone biosynthesis protein COQ9, mitochondrial OS=Mus musculus GN=Coq9 PE=1 SV=1 [MASS=35082]	5	13	1	3.75E+06	3.82E+06	2.21E+06
P12787	COX5A_MOUSE	Cytochrome c oxidase subunit 5A, mitochondrial OS=Mus musculus GN=Cox5a PE=1 SV=2 [MASS=16101]	5	13	1	1.23E+07	1.82E+07	1.67E+07
Q9D1G3	HHATL_MOUSE	Protein-cysteine N-palmitoyltransferase HHAT-like protein OS=Mus musculus GN=Hhatl PE=1 SV=2 [MASS=56430]	5	12	1	7.18E+05	6.16E+05	7.56E+05
Q2TPA8	HSDL2_MOUSE	Hydroxysteroid dehydrogenase-like protein 2 OS=Mus musculus GN=Hsdl2 PE=2 SV=1 [MASS=54208]	6	13	1	1.10E+06	8.35E+05	8.61E+05
P99029	PRDX5_MOUSE	Peroxiredoxin-5, mitochondrial OS=Mus musculus GN=Prdx5 PE=1 SV=2 [MASS=21897]	8	13	1	5.41E+06	6.02E+06	8.65E+06
Q8VDN2	AT1A1_MOUSE	Sodium/potassium-transporting ATPase subunit alpha-1 OS=Mus musculus GN=Atp1a1 PE=1 SV=1 [MASS=112981]	13	13	1	3.46E+06	3.18E+06	3.22E+06
Q9CQA3	DHSB_MOUSE	Succinate dehydrogenase [ubiquinone] iron-sulfur subunit, mitochondrial OS=Mus musculus GN=Sdhb PE=1 SV=1 [MASS=31814]	11	13	1	1.13E+07	1.01E+07	1.08E+07
P97807	FUMH_MOUSE	Fumarate hydratase, mitochondrial OS=Mus musculus GN=Fh PE=1 SV=2 [MASS=54370]	14	13	1	2.33E+07	2.30E+07	2.17E+07
Q9DB20	ATPO_MOUSE	ATP synthase subunit O, mitochondrial OS=Mus musculus GN=Atp5o PE=1 SV=1 [MASS=23363]	8	13	1	2.56E+07	2.51E+07	2.81E+07
P26443	DHE3_MOUSE	Glutamate dehydrogenase 1, mitochondrial OS=Mus musculus GN=Glud1 PE=1 SV=1 [MASS=61336]	8	13	1	7.42E+05	6.18E+05	5.33E+05
P06151	LDHA_MOUSE	L-lactate dehydrogenase A chain OS=Mus musculus GN=Ldha PE=1 SV=3 [MASS=36498]	10	13	1	8.30E+06	7.04E+06	7.70E+06
Q8BH95	ECHM_MOUSE	Enoyl-CoA hydratase, mitochondrial OS=Mus musculus GN=Echs1 PE=1 SV=1 [MASS=31474]	8	13	1	4.80E+06	5.69E+06	6.76E+06
Q03265	ATPA_MOUSE	ATP synthase subunit alpha, mitochondrial OS=Mus musculus GN=Atp5a1 PE=1 SV=1 [MASS=59752]	23	13	1	1.48E+08	1.57E+08	1.55E+08
O35129	PHB2_MOUSE	Prohibitin-2 OS=Mus musculus GN=Phb2 PE=1 SV=1 [MASS=33296]	11	13	1	4.69E+06	4.29E+06	3.83E+06
Q921G7	ETFD_MOUSE	Electron transfer flavoprotein-ubiquinone oxidoreductase, mitochondrial OS=Mus musculus GN=Etfdh PE=1 SV=1 [MASS=68090]	18	13	1	1.09E+07	9.69E+06	1.19E+07
Q9CPU4	MGST3_MOUSE	Microsomal glutathione S-transferase 3 OS=Mus musculus GN=Mgst3 PE=1 SV=1 [MASS=16958]	2	13	1	5.27E+05	3.77E+05	3.32E+05
P03903	NU4LM_MOUSE	NADH-ubiquinone oxidoreductase chain 4L OS=Mus musculus GN=Mtnd4l PE=3 SV=1 [MASS=10493]	1	5	1	1.05E+04	1.70E+04	4.40E+04
Q99KI0	ACON_MOUSE	Aconitate hydratase, mitochondrial OS=Mus musculus GN=Aco2 PE=1 SV=1 [MASS=85463]	27	13	1	1.02E+08	9.36E+07	9.74E+07
P57776	EF1D_MOUSE	Elongation factor 1-delta OS=Mus musculus GN=Eef1d PE=1 SV=3 [MASS=31293]	3	7	1	3.79E+05	3.51E+05	2.57E+05
P20029	GRP78_MOUSE	78 kDa glucose-regulated protein OS=Mus musculus GN=Hspa5 PE=1 SV=3 [MASS=72422]	8	13	1	2.19E+06	2.24E+06	1.79E+06
P67984	RL22_MOUSE	60S ribosomal protein L22 OS=Mus musculus GN=Rpl22 PE=2 SV=2 [MASS=14759]	1	2	0.99	1.21E+05	8.37E+04	7.00E+04
P35486	ODPA_MOUSE	Pyruvate dehydrogenase E1 component subunit alpha, somatic form, mitochondrial OS=Mus musculus GN=Pdha1 PE=1 SV=1 [MASS=43231]	13	13	1	7.72E+06	8.38E+06	7.95E+06
Q01768	NDKB_MOUSE	Nucleoside diphosphate kinase B OS=Mus musculus GN=Nme2 PE=1 SV=1 [MASS=17363]	6	13	1	5.67E+06	4.91E+06	4.38E+06
O55143	AT2A2_MOUSE	Sarcoplasmic/endoplasmic reticulum calcium ATPase 2 OS=Mus musculus GN=Atp2a2 PE=1 SV=2 [MASS=114857]	23	13	1	4.37E+07	3.45E+07	3.58E+07
Q3UIU2	NDUB6_MOUSE	NADH dehydrogenase [ubiquinone] 1 beta subcomplex subunit 6 OS=Mus musculus GN=Ndufb6 PE=2 SV=3 [MASS=15515]	1	13	0.99	1.02E+05	8.16E+04	9.37E+04
P45952	ACADM_MOUSE	Medium-chain specific acyl-CoA dehydrogenase, mitochondrial OS=Mus musculus GN=Acadm PE=1 SV=1 [MASS=46481]	14	13	1	2.15E+07	2.35E+07	2.34E+07
Q60932	VDAC1_MOUSE	Voltage-dependent anion-selective channel protein 1 OS=Mus musculus GN=Vdac1 PE=1 SV=3 [MASS=32351]	13	13	1	2.03E+07	2.05E+07	1.91E+07
P03888	NU1M_MOUSE	NADH-ubiquinone oxidoreductase chain 1 OS=Mus musculus GN=Mtnd1 PE=1 SV=3 [MASS=36059]	4	13	1	1.70E+06	1.66E+06	1.97E+06
Q8BH59	CMC1_MOUSE	Calcium-binding mitochondrial carrier protein Aralar1 OS=Mus musculus GN=Slc25a12 PE=1 SV=1 [MASS=74569]	19	13	1	8.25E+06	7.78E+06	8.50E+06
Q60930	VDAC2_MOUSE	Voltage-dependent anion-selective channel protein 2 OS=Mus musculus GN=Vdac2 PE=1 SV=2 [MASS=31733]	9	13	1	5.75E+06	5.91E+06	5.97E+06
P50462	CSRP3_MOUSE	Cysteine and glycine-rich protein 3 OS=Mus musculus GN=Csrp3 PE=2 SV=1 [MASS=20895]	5	12	1	4.65E+06	5.89E+06	7.25E+06
P03921	NU5M_MOUSE	NADH-ubiquinone oxidoreductase chain 5 OS=Mus musculus GN=Mtnd5 PE=2 SV=2 [MASS=68440]	6	13	1	4.01E+06	3.23E+06	3.54E+06
Q9DCZ4	APOO_MOUSE	Apolipoprotein O OS=Mus musculus GN=Apoo PE=2 SV=1 [MASS=24136]	5	13	1	1.23E+06	1.20E+06	1.08E+06
Q8K2B3	DHSA_MOUSE	Succinate dehydrogenase [ubiquinone] flavoprotein subunit, mitochondrial OS=Mus musculus GN=Sdha PE=1 SV=1 [MASS=72585]	22	13	1	2.07E+07	1.77E+07	2.04E+07
Q9JHI5	IVD_MOUSE	Isovaleryl-CoA dehydrogenase, mitochondrial OS=Mus musculus GN=Ivd PE=1 SV=1 [MASS=46325]	13	13	1	6.32E+06	5.71E+06	5.06E+06
O08749	DLDH_MOUSE	Dihydrolipoyl dehydrogenase, mitochondrial OS=Mus musculus GN=Dld PE=1 SV=2 [MASS=54272]	9	13	1	6.32E+06	6.16E+06	7.10E+06
Q8CC88	K0564_MOUSE	Uncharacterized protein KIAA0564 homolog OS=Mus musculus GN=Kiaa0564 PE=2 SV=2 [MASS=213420]	19	13	1	1.43E+06	1.37E+06	1.34E+06
P11152	LIPL_MOUSE	Lipoprotein lipase OS=Mus musculus GN=Lpl PE=1 SV=2 [MASS=53126]	2	11	1	1.20E+05	1.20E+05	6.79E+04
P52825	CPT2_MOUSE	Carnitine O-palmitoyltransferase 2, mitochondrial OS=Mus musculus GN=Cpt2 PE=1 SV=1 [MASS=73927]	17	13	1	5.92E+06	5.63E+06	5.53E+06
P50544	ACADV_MOUSE	Very long-chain specific acyl-CoA dehydrogenase, mitochondrial OS=Mus musculus GN=Acadvl PE=1 SV=3 [MASS=70875]	27	13	1	4.49E+07	3.98E+07	3.55E+07
Q8BMS1	ECHA_MOUSE	Trifunctional enzyme subunit alpha, mitochondrial OS=Mus musculus GN=Hadha PE=1 SV=1 [MASS=82669]	29	13	1	8.48E+07	7.73E+07	8.77E+07
Q61554	FBN1_MOUSE	Fibrillin-1 OS=Mus musculus GN=Fbn1 PE=1 SV=1 [MASS=312266]	2	13	1	1.83E+04	3.18E+04	6.51E+04
Q8BMF4	ODP2_MOUSE	Dihydrolipoyllysine-residue acetyltransferase component of pyruvate dehydrogenase complex, mitochondrial OS=Mus musculus GN=Dlat PE=1 SV=2 [MASS=67941]	15	13	1	8.00E+06	8.80E+06	1.15E+07
O08756	HCD2_MOUSE	3-hydroxyacyl-CoA dehydrogenase type-2 OS=Mus musculus GN=Hsd17b10 PE=1 SV=4 [MASS=27418]	9	13	1	3.13E+06	2.89E+06	2.81E+06
Q8BK30	NDUV3_MOUSE	NADH dehydrogenase [ubiquinone] flavoprotein 3, mitochondrial OS=Mus musculus GN=Ndufv3 PE=2 SV=1 [MASS=11813]	1	4	0.99	8.53E+05	7.24E+05	1.70E+05
P50752	TNNT2_MOUSE	Troponin T, cardiac muscle OS=Mus musculus GN=Tnnt2 PE=2 SV=2 [MASS=35825]	6	13	1	2.89E+07	3.44E+07	3.70E+07
P56391	CX6B1_MOUSE	Cytochrome c oxidase subunit 6B1 OS=Mus musculus GN=Cox6b1 PE=1 SV=2 [MASS=10071]	3	11	1	2.23E+05	3.48E+05	1.04E+07
Q9D051	ODPB_MOUSE	Pyruvate dehydrogenase E1 component subunit beta, mitochondrial OS=Mus musculus GN=Pdhb PE=1 SV=1 [MASS=38937]	13	13	1	2.10E+07	2.23E+07	2.08E+07
P09542	MYL3_MOUSE	Myosin light chain 3 OS=Mus musculus GN=Myl3 PE=2 SV=4 [MASS=22421]	8	13	1	1.37E+08	1.38E+08	1.17E+08
Q60597	ODO1_MOUSE	2-oxoglutarate dehydrogenase E1 component, mitochondrial OS=Mus musculus GN=Ogdh PE=1 SV=3 [MASS=116448]	34	13	1	2.53E+07	2.60E+07	3.02E+07
P10605	CATB_MOUSE	Cathepsin B OS=Mus musculus GN=Ctsb PE=1 SV=2 [MASS=37280]	1	11	1	1.64E+05	1.47E+05	1.51E+05
Q9DB77	QCR2_MOUSE	Cytochrome b-c1 complex subunit 2, mitochondrial OS=Mus musculus GN=Uqcrc2 PE=1 SV=1 [MASS=48235]	13	13	1	3.57E+07	3.58E+07	3.42E+07
P35979	RL12_MOUSE	60S ribosomal protein L12 OS=Mus musculus GN=Rpl12 PE=1 SV=2 [MASS=17804]	2	7	1	8.54E+04	7.92E+04	9.07E+04
Q8BGH2	SAM50_MOUSE	Sorting and assembly machinery component 50 homolog OS=Mus musculus GN=Samm50 PE=1 SV=1 [MASS=51864]	10	13	1	1.74E+06	1.83E+06	1.91E+06
P62082	RS7_MOUSE	40S ribosomal protein S7 OS=Mus musculus GN=Rps7 PE=2 SV=1 [MASS=22127]	2	13	1	3.23E+05	2.24E+05	2.66E+05
P14824	ANXA6_MOUSE	Annexin A6 OS=Mus musculus GN=Anxa6 PE=1 SV=2 [MASS=75886]	20	13	1	3.13E+06	3.54E+06	3.69E+06
P27773	PDIA3_MOUSE	Protein disulfide-isomerase A3 OS=Mus musculus GN=Pdia3 PE=1 SV=2 [MASS=56678]	9	13	1	7.14E+05	7.40E+05	6.69E+05
P60867	RS20_MOUSE	40S ribosomal protein S20 OS=Mus musculus GN=Rps20 PE=1 SV=1 [MASS=13373]	1	5	0.99	6.26E+04	6.70E+04	1.08E+05
Q91WD5	NDUS2_MOUSE	NADH dehydrogenase [ubiquinone] iron-sulfur protein 2, mitochondrial OS=Mus musculus GN=Ndufs2 PE=1 SV=1 [MASS=52625]	13	13	1	7.71E+06	8.35E+06	8.03E+06
P41216	ACSL1_MOUSE	Long-chain-fatty-acid--CoA ligase 1 OS=Mus musculus GN=Acsl1 PE=1 SV=1 [MASS=77923]	22	13	1	9.08E+06	8.21E+06	1.08E+07
Q9CZ13	QCR1_MOUSE	Cytochrome b-c1 complex subunit 1, mitochondrial OS=Mus musculus GN=Uqcrc1 PE=1 SV=1 [MASS=52768]	14	13	1	1.91E+07	2.04E+07	2.70E+07
O35658	C1QBP_MOUSE	Complement component 1 Q subcomponent-binding protein, mitochondrial OS=Mus musculus GN=C1qbp PE=1 SV=1 [MASS=31013]	5	13	1	5.34E+05	5.72E+05	1.76E+06
P16125	LDHB_MOUSE	L-lactate dehydrogenase B chain OS=Mus musculus GN=Ldhb PE=1 SV=2 [MASS=36572]	13	13	1	1.94E+07	1.86E+07	2.18E+07
Q8JZQ2	AFG32_MOUSE	AFG3-like protein 2 OS=Mus musculus GN=Afg3l2 PE=1 SV=1 [MASS=89518]	4	7	1	1.51E+05	1.91E+05	1.81E+05
P01942	HBA_MOUSE	Hemoglobin subunit alpha OS=Mus musculus GN=Hba PE=1 SV=2 [MASS=15085]	4	13	1	1.07E+07	1.34E+07	1.56E+07
P07724	ALBU_MOUSE	Serum albumin OS=Mus musculus GN=Alb PE=1 SV=3 [MASS=68692]	22	13	1	2.43E+07	2.16E+07	3.38E+07
P04247	MYG_MOUSE	Myoglobin OS=Mus musculus GN=Mb PE=1 SV=3 [MASS=17070]	6	13	1	5.06E+07	5.06E+07	5.94E+07
P68134	ACTS_MOUSE	Actin, alpha skeletal muscle OS=Mus musculus GN=Acta1 PE=1 SV=1 [MASS=42051]	1	11	1	1.80E+06	2.12E+06	1.38E+06
Q9ERS2	NDUAD_MOUSE	NADH dehydrogenase [ubiquinone] 1 alpha subcomplex subunit 13 OS=Mus musculus GN=Ndufa13 PE=1 SV=3 [MASS=16859]	4	13	1	3.59E+06	3.57E+06	4.05E+06
P62897	CYC_MOUSE	Cytochrome c, somatic OS=Mus musculus GN=Cycs PE=1 SV=2 [MASS=11605]	5	13	1	2.24E+07	2.58E+07	2.44E+07
Q60936	ADCK3_MOUSE	Chaperone activity of bc1 complex-like, mitochondrial OS=Mus musculus GN=Cabc1 PE=2 SV=2 [MASS=71743]	7	13	1	1.02E+06	9.35E+05	9.28E+05
Q64475	H2B1P_MOUSE	Histone H2B type 1-B OS=Mus musculus GN=Hist1h2bb PE=1 SV=3 [MASS=13952]	1	13	1	7.03E+05	1.17E+06	9.25E+05
Q9Z2I9	SUCB1_MOUSE	Succinyl-CoA ligase [ADP-forming] subunit beta, mitochondrial OS=Mus musculus GN=Sucla2 PE=1 SV=2 [MASS=50114]	16	13	1	1.19E+07	1.13E+07	9.68E+06
P05202	AATM_MOUSE	Aspartate aminotransferase, mitochondrial OS=Mus musculus GN=Got2 PE=1 SV=1 [MASS=47411]	17	13	1	2.40E+07	2.29E+07	2.74E+07
Q8BWT1	THIM_MOUSE	3-ketoacyl-CoA thiolase, mitochondrial OS=Mus musculus GN=Acaa2 PE=1 SV=2 [MASS=41858]	18	13	1	6.00E+07	6.55E+07	5.71E+07
P61089	UBE2N_MOUSE	Ubiquitin-conjugating enzyme E2 N OS=Mus musculus GN=Ube2n PE=1 SV=1 [MASS=17138]	4	12	1	4.51E+05	5.52E+05	4.33E+05
O54724	PTRF_MOUSE	Polymerase I and transcript release factor OS=Mus musculus GN=Ptrf PE=1 SV=1 [MASS=43953]	5	13	1	2.26E+06	3.16E+06	2.59E+06
Q9CQQ7	AT5F1_MOUSE	ATP synthase subunit b, mitochondrial OS=Mus musculus GN=Atp5f1 PE=1 SV=1 [MASS=28949]	7	13	1	1.03E+07	9.94E+06	8.41E+06
P24270	CATA_MOUSE	Catalase OS=Mus musculus GN=Cat PE=1 SV=3 [MASS=59765]	7	10	1	7.44E+05	5.82E+05	4.53E+05
Q07417	ACADS_MOUSE	Short-chain specific acyl-CoA dehydrogenase, mitochondrial OS=Mus musculus GN=Acads PE=2 SV=1 [MASS=44946]	15	13	1	8.68E+06	8.43E+06	8.57E+06
Q9CR68	UCRI_MOUSE	Cytochrome b-c1 complex subunit Rieske, mitochondrial OS=Mus musculus GN=Uqcrfs1 PE=1 SV=1 [MASS=29367]	8	13	1	8.30E+06	8.59E+06	7.52E+06
Q8BFR5	EFTU_MOUSE	Elongation factor Tu, mitochondrial OS=Mus musculus GN=Tufm PE=1 SV=1 [MASS=49508]	13	13	1	6.76E+06	6.90E+06	7.28E+06
Q9D8B4	NDUAB_MOUSE	NADH dehydrogenase [ubiquinone] 1 alpha subcomplex subunit 11 OS=Mus musculus GN=Ndufa11 PE=2 SV=2 [MASS=14982]	3	13	1	8.11E+05	5.93E+05	1.13E+06
Q08857	CD36_MOUSE	Platelet glycoprotein 4 OS=Mus musculus GN=Cd36 PE=2 SV=2 [MASS=52698]	8	13	1	3.71E+06	3.19E+06	3.52E+06
Q9CR62	M2OM_MOUSE	Mitochondrial 2-oxoglutarate/malate carrier protein OS=Mus musculus GN=Slc25a11 PE=1 SV=3 [MASS=34155]	13	13	1	6.68E+06	6.96E+06	7.97E+06
Q8BFZ9	ERLN2_MOUSE	Erlin-2 OS=Mus musculus GN=Erlin2 PE=1 SV=1 [MASS=37872]	1	3	0.99	3.98E+04	3.49E+04	3.52E+04
P67778	PHB_MOUSE	Prohibitin OS=Mus musculus GN=Phb PE=1 SV=1 [MASS=29820]	9	13	1	2.51E+06	2.71E+06	2.28E+06
P51667	MLRV_MOUSE	Myosin regulatory light chain 2, ventricular/cardiac muscle isoform OS=Mus musculus GN=Myl2 PE=2 SV=3 [MASS=18864]	9	11	1	3.22E+07	3.29E+07	2.84E+07
Q6P8J7	KCRS_MOUSE	Creatine kinase S-type, mitochondrial OS=Mus musculus GN=Ckmt2 PE=1 SV=1 [MASS=47473]	13	13	1	3.27E+07	3.40E+07	4.69E+07
Q61941	NNTM_MOUSE	NAD(P) transhydrogenase, mitochondrial OS=Mus musculus GN=Nnt PE=1 SV=2 [MASS=113838]	25	13	1	2.01E+07	1.91E+07	2.23E+07

Swiss-Prot ID	Intensity [SHAM 21d]	Intensity [SHAM 28d]	Intensity [SHAM 42d]	Intensity [SHAM 56d]	Intensity [TAC 4d]	Intensity [TAC 14d]	Intensity [TAC 21d]	Intensity [TAC 28d]	Intensity [TAC 42d]	Intensity [TAC 56d]	P-Value [base]	P-Value [SHAM 4d]	P-Value [SHAM 14d]	P-Value [SHAM 21d]	P-Value [SHAM 28d]	P-Value [SHAM 42d]	P-Value [SHAM 56d]	P-Value [TAC 4d]	P-Value [TAC 14d]	P-Value [TAC 21d]	P-Value [TAC 28d]	P-Value [TAC 42d]	P-Value [TAC 56d]	Isotope Count	Peptide Count Score	Scan Total
P58771	5.33E+07	5.44E+07	5.09E+07	5.83E+07	5.30E+07	5.06E+07	5.32E+07	5.77E+07	5.32E+07	5.48E+07	0	0	0	0	0	0	0	0	0	0	0	0	0	3, 6, 5, 6, 6	11, 12, 13, 2, 9, 5, 11, 8, 12	10, 2, 11, 12, 11, 5, 12, 8, 13
P58281	9.87E+05	9.69E+05	1.19E+06	1.06E+06	1.04E+06	8.75E+05	8.32E+05	9.81E+05	9.23E+05	8.77E+05	2.42E-10	1.45E-12	3.79E-25	5.70E-17	6.19E-13	3.73E-16	2.55E-18	1.22E-16	1.74E-13	2.44E-16	1.79E-24	1.01E-09	4.25E-16	2, 3, 4, 5, 6, 7	1, 10, 13, 3, 4, 6	1, 10, 13, 14, 3, 4, 6
P63038	2.06E+07	2.14E+07	2.15E+07	2.05E+07	2.22E+07	1.68E+07	1.81E+07	1.80E+07	1.97E+07	1.60E+07	0	0	0	0	0	0	0	0	0	0	0	0	0	2, 5, 3, 3, 4, 3, 5, 4, 4, 5, 4, 7, 5, 6, 6, 7	11, 12, 13, 13, 2, 13, 3, 7, 3, 9, 5, 11, 5, 13, 8	11, 11, 5, 12, 13, 13, 13, 14, 13, 2, 13, 5, 3, 7, 3, 9, 8
Q8VEM8	2.05E+07	2.09E+07	2.00E+07	2.33E+07	1.80E+07	1.71E+07	2.23E+07	2.10E+07	1.67E+07	1.81E+07	0	0	0	0	0	0	0	0	0	0	0	0	0	3, 4, 4, 5, 4, 6, 4, 7, 5, 7	1, 13, 11, 11, 13, 12, 13, 5, 7	1, 13, 11, 11, 13, 12, 13, 5, 7
O70468	3.97E+07	4.05E+07	4.19E+07	4.51E+07	4.12E+07	4.08E+07	5.07E+07	4.42E+07	4.01E+07	4.58E+07	0	0	0	0	0	0	0	0	0	0	0	0	0	2, 2, 4, 2, 7, 3, 3, 5, 3, 6, 4, 4, 5, 4, 7, 5, 5, 6, 5, 8, 6, 7, 8	1, 11, 10, 11, 11, 13, 12, 12, 13, 13, 2, 2, 11, 2, 5, 3, 3, 10, 4, 12, 6, 7, 12, 8, 13, 9	1, 11, 10, 10, 3, 11, 11, 13, 11, 2, 12, 12, 13, 12, 15, 12, 4, 12, 7, 13, 13, 14, 13, 8, 15, [illegible]
Q9D855	6.75E+06	6.88E+06	6.20E+06	8.89E+06	6.73E+06	5.59E+06	5.43E+06	5.45E+06	6.12E+06	5.98E+06	0	0	0	0	0	0	0	0	0	0	0	0	0	4, 5, 6	13, 4, 12, 6	12, 4, 13, 6
Q9CQZ5	3.35E+06	3.10E+06	3.09E+06	3.55E+06	3.17E+06	2.22E+06	3.23E+06	3.10E+06	2.10E+06	3.11E+06	3.37E-24	3.72E-39	0	0	2.14E-36	6.31E-38	0	4.20E-45	8.58E-31	0	0	1.10E-19	0	2, 3, 3, 4, 4, 5, 5	1, 13, 1, 9, 11, 13	1, 13, 1, 9, 11, 13
Q9Z0X1	2.44E+06	2.40E+06	2.32E+06	2.10E+06	2.29E+06	1.76E+06	1.91E+06	2.05E+06	2.00E+06	1.86E+06	7.95E-27	9.62E-33	0	7.95E-39	4.17E-29	1.48E-29	3.52E-34	5.40E-34	3.65E-25	6.80E-35	0	6.27E-19	8.71E-32	2, 3, 4, 5, 6, 7	13, 2, 3, 5, 6, 9	13, 2, 3, 5, 6, 9
P14206	[illegible]	[illegible]	[illegible]	[illegible]	[illegible]	[illegible]	[illegible]	[illegible]	[illegible]	[illegible]	[illegible]	[illegible]	[illegible]	[illegible]	[illegible]	[illegible]	[illegible]	[illegible]	[illegible]	[illegible]	[illegible]	[illegible]	[illegible]	[illegible]	[illegible]	[illegible]
Q99JY0	[illegible]	[illegible]	[illegible]	[illegible]	[illegible]	[illegible]	[illegible]	[illegible]	[illegible]	[illegible]	[illegible]	[illegible]	[illegible]	[illegible]	[illegible]	[illegible]	[illegible]	[illegible]	[illegible]	[illegible]	[illegible]	[illegible]	[illegible]	[illegible]	[illegible]	[illegible]
Q91VD9	[illegible]	[illegible]	[illegible]	[illegible]	[illegible]	[illegible]	[illegible]	[illegible]	[illegible]	[illegible]	[illegible]	[illegible]	[illegible]	[illegible]	[illegible]	[illegible]	[illegible]	[illegible]	[illegible]	[illegible]	[illegible]	[illegible]	[illegible]	[illegible]	[illegible]	[illegible]
P47738	[illegible]	[illegible]	[illegible]	[illegible]	[illegible]	[illegible]	[illegible]	[illegible]	[illegible]	[illegible]	[illegible]	[illegible]	[illegible]	[illegible]	[illegible]	[illegible]	[illegible]	[illegible]	[illegible]	[illegible]	[illegible]	[illegible]	[illegible]	[illegible]	[illegible]	[illegible]
P56480	[illegible]	[illegible]	[illegible]	[illegible]	[illegible]	[illegible]	[illegible]	[illegible]	[illegible]	[illegible]	[illegible]	[illegible]	[illegible]	[illegible]	[illegible]	[illegible]	[illegible]	[illegible]	[illegible]	[illegible]	[illegible]	[illegible]	[illegible]	[illegible]	[illegible]	[illegible]
P48962	[illegible]	[illegible]	[illegible]	[illegible]	[illegible]	[illegible]	[illegible]	[illegible]	[illegible]	[illegible]	[illegible]	[illegible]	[illegible]	[illegible]	[illegible]	[illegible]	[illegible]	[illegible]	[illegible]	[illegible]	[illegible]	[illegible]	[illegible]	[illegible]	[illegible]	[illegible]
P63017	[illegible]	[illegible]	[illegible]	[illegible]	[illegible]	[illegible]	[illegible]	[illegible]	[illegible]	[illegible]	[illegible]	[illegible]	[illegible]	[illegible]	[illegible]	[illegible]	[illegible]	[illegible]	[illegible]	[illegible]	[illegible]	[illegible]	[illegible]	[illegible]	[illegible]	[illegible]
O35459	[illegible]	[illegible]	[illegible]	[illegible]	[illegible]	[illegible]	[illegible]	[illegible]	[illegible]	[illegible]	[illegible]	[illegible]	[illegible]	[illegible]	[illegible]	[illegible]	[illegible]	[illegible]	[illegible]	[illegible]	[illegible]	[illegible]	[illegible]	[illegible]	[illegible]	[illegible]
Q02566	[illegible]	[illegible]	[illegible]	[illegible]	[illegible]	[illegible]	[illegible]	[illegible]	[illegible]	[illegible]	[illegible]	[illegible]	[illegible]	[illegible]	[illegible]	[illegible]	[illegible]	[illegible]	[illegible]	[illegible]	[illegible]	[illegible]	[illegible]	[illegible]	[illegible]	[illegible]
P54071	[illegible]	[illegible]	[illegible]	[illegible]	[illegible]	[illegible]	[illegible]	[illegible]	[illegible]	[illegible]	[illegible]	[illegible]	[illegible]	[illegible]	[illegible]	[illegible]	[illegible]	[illegible]	[illegible]	[illegible]	[illegible]	[illegible]	[illegible]	[illegible]	[illegible]	[illegible]
P62900	[illegible]	[illegible]	[illegible]	[illegible]	[illegible]	[illegible]	[illegible]	[illegible]	[illegible]	[illegible]	[illegible]	[illegible]	[illegible]	[illegible]	[illegible]	[illegible]	[illegible]	[illegible]	[illegible]	[illegible]	[illegible]	[illegible]	[illegible]	[illegible]	[illegible]	[illegible]
P16858	[illegible]	[illegible]	[illegible]	[illegible]	[illegible]	[illegible]	[illegible]	[illegible]	[illegible]	[illegible]	[illegible]	[illegible]	[illegible]	[illegible]	[illegible]	[illegible]	[illegible]	[illegible]	[illegible]	[illegible]	[illegible]	[illegible]	[illegible]	[illegible]	[illegible]	[illegible]
Q91YT0	[illegible]	[illegible]	[illegible]	[illegible]	[illegible]	[illegible]	[illegible]	[illegible]	[illegible]	[illegible]	[illegible]	[illegible]	[illegible]	[illegible]	[illegible]	[illegible]	[illegible]	[illegible]	[illegible]	[illegible]	[illegible]	[illegible]	[illegible]	[illegible]	[illegible]	[illegible]
P01887	[illegible]	[illegible]	[illegible]	[illegible]	[illegible]	[illegible]	[illegible]	[illegible]	[illegible]	[illegible]	[illegible]	[illegible]	[illegible]	[illegible]	[illegible]	[illegible]	[illegible]	[illegible]	[illegible]	[illegible]	[illegible]	[illegible]	[illegible]	[illegible]	[illegible]	[illegible]
Q9CR61	[illegible]	[illegible]	[illegible]	[illegible]	[illegible]	[illegible]	[illegible]	[illegible]	[illegible]	[illegible]	[illegible]	[illegible]	[illegible]	[illegible]	[illegible]	[illegible]	[illegible]	[illegible]	[illegible]	[illegible]	[illegible]	[illegible]	[illegible]	[illegible]	[illegible]	[illegible]
P47911	[illegible]	[illegible]	[illegible]	[illegible]	[illegible]	[illegible]	[illegible]	[illegible]	[illegible]	[illegible]	[illegible]	[illegible]	[illegible]	[illegible]	[illegible]	[illegible]	[illegible]	[illegible]	[illegible]	[illegible]	[illegible]	[illegible]	[illegible]	[illegible]	[illegible]	[illegible]
Q9D0K2	[illegible]	[illegible]	[illegible]	[illegible]	[illegible]	[illegible]	[illegible]	[illegible]	[illegible]	[illegible]	[illegible]	[illegible]	[illegible]	[illegible]	[illegible]	[illegible]	[illegible]	[illegible]	[illegible]	[illegible]	[illegible]	[illegible]	[illegible]	[illegible]	[illegible]	[illegible]
Q7TQ48	[illegible]	[illegible]	[illegible]	[illegible]	[illegible]	[illegible]	[illegible]	[illegible]	[illegible]	[illegible]	[illegible]	[illegible]	[illegible]	[illegible]	[illegible]	[illegible]	[illegible]	[illegible]	[illegible]	[illegible]	[illegible]	[illegible]	[illegible]	[illegible]	[illegible]	[illegible]
Q8CAQ8	[illegible]	[illegible]	[illegible]	[illegible]	[illegible]	[illegible]	[illegible]	[illegible]	[illegible]	[illegible]	[illegible]	[illegible]	[illegible]	[illegible]	[illegible]	[illegible]	[illegible]	[illegible]	[illegible]	[illegible]	[illegible]	[illegible]	[illegible]	[illegible]	[illegible]	[illegible]
Q01853	[illegible]	[illegible]	[illegible]	[illegible]	[illegible]	[illegible]	[illegible]	[illegible]	[illegible]	[illegible]	[illegible]	[illegible]	[illegible]	[illegible]	[illegible]	[illegible]	[illegible]	[illegible]	[illegible]	[illegible]	[illegible]	[illegible]	[illegible]	[illegible]	[illegible]	[illegible]
P17710	[illegible]	[illegible]	[illegible]	[illegible]	[illegible]	[illegible]	[illegible]	[illegible]	[illegible]	[illegible]	[illegible]	[illegible]	[illegible]	[illegible]	[illegible]	[illegible]	[illegible]	[illegible]	[illegible]	[illegible]	[illegible]	[illegible]	[illegible]	[illegible]	[illegible]	[illegible]
Q99LC5	[illegible]	[illegible]	[illegible]	[illegible]	[illegible]	[illegible]	[illegible]	[illegible]	[illegible]	[illegible]	[illegible]	[illegible]	[illegible]	[illegible]	[illegible]	[illegible]	[illegible]	[illegible]	[illegible]	[illegible]	[illegible]	[illegible]	[illegible]	[illegible]	[illegible]	[illegible]
Q8K1Z0	[illegible]	[illegible]	[illegible]	[illegible]	[illegible]	[illegible]	[illegible]	[illegible]	[illegible]	[illegible]	[illegible]	[illegible]	[illegible]	[illegible]	[illegible]	[illegible]	[illegible]	[illegible]	[illegible]	[illegible]	[illegible]	[illegible]	[illegible]	[illegible]	[illegible]	[illegible]
P12787	[illegible]	[illegible]	[illegible]	[illegible]	[illegible]	[illegible]	[illegible]	[illegible]	[illegible]	[illegible]	[illegible]	[illegible]	[illegible]	[illegible]	[illegible]	[illegible]	[illegible]	[illegible]	[illegible]	[illegible]	[illegible]	[illegible]	[illegible]	[illegible]	[illegible]	[illegible]
Q9D1G3	[illegible]	[illegible]	[illegible]	[illegible]	[illegible]	[illegible]	[illegible]	[illegible]	[illegible]	[illegible]	[illegible]	[illegible]	[illegible]	[illegible]	[illegible]	[illegible]	[illegible]	[illegible]	[illegible]	[illegible]	[illegible]	[illegible]	[illegible]	[illegible]	[illegible]	[illegible]
Q2TPA8	[illegible]	[illegible]	[illegible]	[illegible]	[illegible]	[illegible]	[illegible]	[illegible]	[illegible]	[illegible]	[illegible]	[illegible]	[illegible]	[illegible]	[illegible]	[illegible]	[illegible]	[illegible]	[illegible]	[illegible]	[illegible]	[illegible]	[illegible]	[illegible]	[illegible]	[illegible]
P99029	[illegible]	[illegible]	[illegible]	[illegible]	[illegible]	[illegible]	[illegible]	[illegible]	[illegible]	[illegible]	[illegible]	[illegible]	[illegible]	[illegible]	[illegible]	[illegible]	[illegible]	[illegible]	[illegible]	[illegible]	[illegible]	[illegible]	[illegible]	[illegible]	[illegible]	[illegible]
Q8VDN2	[illegible]	[illegible]	[illegible]	[illegible]	[illegible]	[illegible]	[illegible]	[illegible]	[illegible]	[illegible]	[illegible]	[illegible]	[illegible]	[illegible]	[illegible]	[illegible]	[illegible]	[illegible]	[illegible]	[illegible]	[illegible]	[illegible]	[illegible]	[illegible]	[illegible]	[illegible]
Q9CQA3	[illegible]	[illegible]	[illegible]	[illegible]	[illegible]	[illegible]	[illegible]	[illegible]	[illegible]	[illegible]	[illegible]	[illegible]	[illegible]	[illegible]	[illegible]	[illegible]	[illegible]	[illegible]	[illegible]	[illegible]	[illegible]	[illegible]	[illegible]	[illegible]	[illegible]	[illegible]
P97807	[illegible]	[illegible]	[illegible]	[illegible]	[illegible]	[illegible]	[illegible]	[illegible]	[illegible]	[illegible]	[illegible]	[illegible]	[illegible]	[illegible]	[illegible]	[illegible]	[illegible]	[illegible]	[illegible]	[illegible]	[illegible]	[illegible]	[illegible]	[illegible]	[illegible]	[illegible]
Q9DB20	[illegible]	[illegible]	[illegible]	[illegible]	[illegible]	[illegible]	[illegible]	[illegible]	[illegible]	[illegible]	[illegible]	[illegible]	[illegible]	[illegible]	[illegible]	[illegible]	[illegible]	[illegible]	[illegible]	[illegible]	[illegible]	[illegible]	[illegible]	[illegible]	[illegible]	[illegible]
P26443	[illegible]	[illegible]	[illegible]	[illegible]	[illegible]	[illegible]	[illegible]	[illegible]	[illegible]	[illegible]	[illegible]	[illegible]	[illegible]	[illegible]	[illegible]	[illegible]	[illegible]	[illegible]	[illegible]	[illegible]	[illegible]	[illegible]	[illegible]	[illegible]	[illegible]	[illegible]
P06151	[illegible]	[illegible]	[illegible]	[illegible]	[illegible]	[illegible]	[illegible]	[illegible]	[illegible]	[illegible]	[illegible]	[illegible]	[illegible]	[illegible]	[illegible]	[illegible]	[illegible]	[illegible]	[illegible]	[illegible]	[illegible]	[illegible]	[illegible]	[illegible]	[illegible]	[illegible]
Q8BH95	[illegible]	[illegible]	[illegible]	[illegible]	[illegible]	[illegible]	[illegible]	[illegible]	[illegible]	[illegible]	[illegible]	[illegible]	[illegible]	[illegible]	[illegible]	[illegible]	[illegible]	[illegible]	[illegible]	[illegible]	[illegible]	[illegible]	[illegible]	[illegible]	[illegible]	[illegible]
Q03265	[illegible]	[illegible]	[illegible]	[illegible]	[illegible]	[illegible]	[illegible]	[illegible]	[illegible]	[illegible]	[illegible]	[illegible]	[illegible]	[illegible]	[illegible]	[illegible]	[illegible]	[illegible]	[illegible]	[illegible]	[illegible]	[illegible]	[illegible]	[illegible]	[illegible]	[illegible]
O35129	[illegible]	[illegible]	[illegible]	[illegible]	[illegible]	[illegible]	[illegible]	[illegible]	[illegible]	[illegible]	[illegible]	[illegible]	[illegible]	[illegible]	[illegible]	[illegible]	[illegible]	[illegible]	[illegible]	[illegible]	[illegible]	[illegible]	[illegible]	[illegible]	[illegible]	[illegible]
Q921G7	[illegible]	[illegible]	[illegible]	[illegible]	[illegible]	[illegible]	[illegible]	[illegible]	[illegible]	[illegible]	[illegible]	[illegible]	[illegible]	[illegible]	[illegible]	[illegible]	[illegible]	[illegible]	[illegible]	[illegible]	[illegible]	[illegible]	[illegible]	[illegible]	[illegible]	[illegible]
Q9CPU4	[illegible]	[illegible]	[illegible]	[illegible]	[illegible]	[illegible]	[illegible]	[illegible]	[illegible]	[illegible]	[illegible]	[illegible]	[illegible]	[illegible]	[illegible]	[illegible]	[illegible]	[illegible]	[illegible]	[illegible]	[illegible]	[illegible]	[illegible]	[illegible]	[illegible]	[illegible]
P03903	[illegible]	[illegible]	[illegible]	[illegible]	[illegible]	[illegible]	[illegible]	[illegible]	[illegible]	[illegible]	[illegible]	[illegible]	[illegible]	[illegible]	[illegible]	[illegible]	[illegible]	[illegible]	[illegible]	[illegible]	[illegible]	[illegible]	[illegible]	[illegible]	[illegible]	[illegible]
Q99KI0	[illegible]	[illegible]	[illegible]	[illegible]	[illegible]	[illegible]	[illegible]	[illegible]	[illegible]	[illegible]	[illegible]	[illegible]	[illegible]	[illegible]	[illegible]	[illegible]	[illegible]	[illegible]	[illegible]	[illegible]	[illegible]	[illegible]	[illegible]	[illegible]	[illegible]	[illegible]
P57776	[illegible]	[illegible]	[illegible]	[illegible]	[illegible]	[illegible]	[illegible]	[illegible]	[illegible]	[illegible]	[illegible]	[illegible]	[illegible]	[illegible]	[illegible]	[illegible]	[illegible]	[illegible]	[illegible]	[illegible]	[illegible]	[illegible]	[illegible]	[illegible]	[illegible]	[illegible]
P20029	[illegible]	[illegible]	[illegible]	[illegible]	[illegible]	[illegible]	[illegible]	[illegible]	[illegible]	[illegible]	[illegible]	[illegible]	[illegible]	[illegible]	[illegible]	[illegible]	[illegible]	[illegible]	[illegible]	[illegible]	[illegible]	[illegible]	[illegible]	[illegible]	[illegible]	[illegible]
P67984	[illegible]	[illegible]	[illegible]	[illegible]	[illegible]	[illegible]	[illegible]	[illegible]	[illegible]	[illegible]	[illegible]	[illegible]	[illegible]	[illegible]	[illegible]	[illegible]	[illegible]	[illegible]	[illegible]	[illegible]	[illegible]	[illegible]	[illegible]	[illegible]	[illegible]	[illegible]
P35486	[illegible]	[illegible]	[illegible]	[illegible]	[illegible]	[illegible]	[illegible]	[illegible]	[illegible]	[illegible]	[illegible]	[illegible]	[illegible]	[illegible]	[illegible]	[illegible]	[illegible]	[illegible]	[illegible]	[illegible]	[illegible]	[illegible]	[illegible]	[illegible]	[illegible]	[illegible]
Q01768	[illegible]	[illegible]	[illegible]	[illegible]	[illegible]	[illegible]	[illegible]	[illegible]	[illegible]	[illegible]	[illegible]	[illegible]	[illegible]	[illegible]	[illegible]	[illegible]	[illegible]	[illegible]	[illegible]	[illegible]	[illegible]	[illegible]	[illegible]	[illegible]	[illegible]	[illegible]
O55143	[illegible]	[illegible]	[illegible]	[illegible]	[illegible]	[illegible]	[illegible]	[illegible]	[illegible]	[illegible]	[illegible]	[illegible]	[illegible]	[illegible]	[illegible]	[illegible]	[illegible]	[illegible]	[illegible]	[illegible]	[illegible]	[illegible]	[illegible]	[illegible]	[illegible]	[illegible]
Q3UIU2	[illegible]	[illegible]	[illegible]	[illegible]	[illegible]	[illegible]	[illegible]	[illegible]	[illegible]	[illegible]	[illegible]	[illegible]	[illegible]	[illegible]	[illegible]	[illegible]	[illegible]	[illegible]	[illegible]	[illegible]	[illegible]	[illegible]	[illegible]	[illegible]	[illegible]	[illegible]
P45952	[illegible]	[illegible]	[illegible]	[illegible]	[illegible]	[illegible]	[illegible]	[illegible]	[illegible]	[illegible]	[illegible]	[illegible]	[illegible]	[illegible]	[illegible]	[illegible]	[illegible]	[illegible]	[illegible]	[illegible]	[illegible]	[illegible]	[illegible]	[illegible]	[illegible]	[illegible]
Q60932	[illegible]	[illegible]	[illegible]	[illegible]	[illegible]	[illegible]	[illegible]	[illegible]	[illegible]	[illegible]	[illegible]	[illegible]	[illegible]	[illegible]	[illegible]	[illegible]	[illegible]	[illegible]	[illegible]	[illegible]	[illegible]	[illegible]	[illegible]	[illegible]	[illegible]	[illegible]
P03888	[illegible]	[illegible]	[illegible]	[illegible]	[illegible]	[illegible]	[illegible]	[illegible]	[illegible]	[illegible]	[illegible]	[illegible]	[illegible]	[illegible]	[illegible]	[illegible]	[illegible]	[illegible]	[illegible]	[illegible]	[illegible]	[illegible]	[illegible]	[illegible]	[illegible]	[illegible]
Q8BH59	[illegible]	[illegible]	[illegible]	[illegible]	[illegible]	[illegible]	[illegible]	[illegible]	[illegible]	[illegible]	[illegible]	[illegible]	[illegible]	[illegible]	[illegible]	[illegible]	[illegible]	[illegible]	[illegible]	[illegible]	[illegible]	[illegible]	[illegible]	[illegible]	[illegible]	[illegible]
Q60930	[illegible]	[illegible]	[illegible]	[illegible]	[illegible]	[illegible]	[illegible]	[illegible]	[illegible]	[illegible]	[illegible]	[illegible]	[illegible]	[illegible]	[illegible]	[illegible]	[illegible]	[illegible]	[illegible]	[illegible]	[illegible]	[illegible]	[illegible]	[illegible]	[illegible]	[illegible]
P50462	[illegible]	[illegible]	[illegible]	[illegible]	[illegible]	[illegible]	[illegible]	[illegible]	[illegible]	[illegible]	[illegible]	[illegible]	[illegible]	[illegible]	[illegible]	[illegible]	[illegible]	[illegible]	[illegible]	[illegible]	[illegible]	[illegible]	[illegible]	[illegible]	[illegible]	[illegible]
P03921	[illegible]	[illegible]	[illegible]	[illegible]	[illegible]	[illegible]	[illegible]	[illegible]	[illegible]	[illegible]	[illegible]	[illegible]	[illegible]	[illegible]	[illegible]	[illegible]	[illegible]	[illegible]	[illegible]	[illegible]	[illegible]	[illegible]	[illegible]	[illegible]	[illegible]	[illegible]
Q9DCZ4	[illegible]	[illegible]	[illegible]	[illegible]	[illegible]	[illegible]	[illegible]	[illegible]	[illegible]	[illegible]	[illegible]	[illegible]	[illegible]	[illegible]	[illegible]	[illegible]	[illegible]	[illegible]	[illegible]	[illegible]	[illegible]	[illegible]	[illegible]	[illegible]	[illegible]	[illegible]
Q8K2B3	[illegible]	[illegible]	[illegible]	[illegible]	[illegible]	[illegible]	[illegible]	[illegible]	[illegible]	[illegible]	[illegible]	[illegible]	[illegible]	[illegible]	[illegible]	[illegible]	[illegible]	[illegible]	[illegible]	[illegible]	[illegible]	[illegible]	[illegible]	[illegible]	[illegible]	[illegible]
Q9JHI5	[illegible]	[illegible]	[illegible]	[illegible]	[illegible]	[illegible]	[illegible]	[illegible]	[illegible]	[illegible]	[illegible]	[illegible]	[illegible]	[illegible]	[illegible]	[illegible]	[illegible]	[illegible]	[illegible]	[illegible]	[illegible]	[illegible]	[illegible]	[illegible]	[illegible]	[illegible]
O08749	[illegible]	[illegible]	[illegible]	[illegible]	[illegible]	[illegible]	[illegible]	[illegible]	[illegible]	[illegible]	[illegible]	[illegible]	[illegible]	[illegible]	[illegible]	[illegible]	[illegible]	[illegible]	[illegible]	[illegible]	[illegible]	[illegible]	[illegible]	[illegible]	[illegible]	[illegible]
Q8CC88	[illegible]	[illegible]	[illegible]	[illegible]	[illegible]	[illegible]	[illegible]	[illegible]	[illegible]	[illegible]	[illegible]	[illegible]	[illegible]	[illegible]	[illegible]	[illegible]	[illegible]	[illegible]	[illegible]	[illegible]	[illegible]	[illegible]	[illegible]	[illegible]	[illegible]	[illegible]
P11152	[illegible]	[illegible]	[illegible]	[illegible]	[illegible]	[illegible]	[illegible]	[illegible]	[illegible]	[illegible]	[illegible]	[illegible]	[illegible]	[illegible]	[illegible]	[illegible]	[illegible]	[illegible]	[illegible]	[illegible]	[illegible]	[illegible]	[illegible]	[illegible]	[illegible]	[illegible]
P52825	[illegible]	[illegible]	[illegible]	[illegible]	[illegible]	[illegible]	[illegible]	[illegible]	[illegible]	[illegible]	[illegible]	[illegible]	[illegible]	[illegible]	[illegible]	[illegible]	[illegible]	[illegible]	[illegible]	[illegible]	[illegible]	[illegible]	[illegible]	[illegible]	[illegible]	[illegible]
P50544	[illegible]	[illegible]	[illegible]	[illegible]	[illegible]	[illegible]	[illegible]	[illegible]	[illegible]	[illegible]	[illegible]	[illegible]	[illegible]	[illegible]	[illegible]	[illegible]	[illegible]	[illegible]	[illegible]	[illegible]	[illegible]	[illegible]	[illegible]	[illegible]	[illegible]	[illegible]
Q8BMS1	[illegible]	[illegible]	[illegible]	[illegible]	[illegible]	[illegible]	[illegible]	[illegible]	[illegible]	[illegible]	[illegible]	[illegible]	[illegible]	[illegible]	[illegible]	[illegible]	[illegible]	[illegible]	[illegible]	[illegible]	[illegible]	[illegible]	[illegible]	[illegible]	[illegible]	[illegible]
Q61554	[illegible]	[illegible]	[illegible]	[illegible]	[illegible]	[illegible]	[illegible]	[illegible]	[illegible]	[illegible]	[illegible]	[illegible]	[illegible]	[illegible]	[illegible]	[illegible]	[illegible]	[illegible]	[illegible]	[illegible]	[illegible]	[illegible]	[illegible]	[illegible]	[illegible]	[illegible]
Q8BMF4	[illegible]	[illegible]	[illegible]	[illegible]	[illegible]	[illegible]	[illegible]	[illegible]	[illegible]	[illegible]	[illegible]	[illegible]	[illegible]	[illegible]	[illegible]	[illegible]	[illegible]	[illegible]	[illegible]	[illegible]	[illegible]	[illegible]	[illegible]	[illegible]	[illegible]	[illegible]
O08756	[illegible]	[illegible]	[illegible]	[illegible]	[illegible]	[illegible]	[illegible]	[illegible]	[illegible]	[illegible]	[illegible]	[illegible]	[illegible]	[illegible]	[illegible]	[illegible]	[illegible]	[illegible]	[illegible]	[illegible]	[illegible]	[illegible]	[illegible]	[illegible]	[illegible]	[illegible]
Q8BK30	[illegible]	[illegible]	[illegible]	[illegible]	[illegible]	[illegible]	[illegible]	[illegible]	[illegible]	[illegible]	[illegible]	[illegible]	[illegible]	[illegible]	[illegible]	[illegible]	[illegible]	[illegible]	[illegible]	[illegible]	[illegible]	[illegible]	[illegible]	[illegible]	[illegible]	[illegible]
P50752	[illegible]	[illegible]	[illegible]	[illegible]	[illegible]	[illegible]	[illegible]	[illegible]	[illegible]	[illegible]	[illegible]	[illegible]	[illegible]	[illegible]	[illegible]	[illegible]	[illegible]	[illegible]	[illegible]	[illegible]	[illegible]	[illegible]	[illegible]	[illegible]	[illegible]	[illegible]
P56391	[illegible]	[illegible]	[illegible]	[illegible]	[illegible]	[illegible]	[illegible]	[illegible]	[illegible]	[illegible]	[illegible]	[illegible]	[illegible]	[illegible]	[illegible]	[illegible]	[illegible]	[illegible]	[illegible]	[illegible]	[illegible]	[illegible]	[illegible]	[illegible]	[illegible]	[illegible]
Q9D051	[illegible]	[illegible]	[illegible]	[illegible]	[illegible]	[illegible]	[illegible]	[illegible]	[illegible]	[illegible]	[illegible]	[illegible]	[illegible]	[illegible]	[illegible]	[illegible]	[illegible]	[illegible]	[illegible]	[illegible]	[illegible]	[illegible]	[illegible]	[illegible]	[illegible]	[illegible]
P09542	[illegible]	[illegible]	[illegible]	[illegible]	[illegible]	[illegible]	[illegible]	[illegible]	[illegible]	[illegible]	[illegible]	[illegible]	[illegible]	[illegible]	[illegible]	[illegible]	[illegible]	[illegible]	[illegible]	[illegible]	[illegible]	[illegible]	[illegible]	[illegible]	[illegible]	[illegible]
Q60597	[illegible]	[illegible]	[illegible]	[illegible]	[illegible]	[illegible]	[illegible]	[illegible]	[illegible]	[illegible]	[illegible]	[illegible]	[illegible]	[illegible]	[illegible]	[illegible]	[illegible]	[illegible]	[illegible]	[illegible]	[illegible]	[illegible]	[illegible]	[illegible]	[illegible]	[illegible]
P10605	[illegible]	[illegible]	[illegible]	[illegible]	[illegible]	[illegible]	[illegible]	[illegible]	[illegible]	[illegible]	[illegible]	[illegible]	[illegible]	[illegible]	[illegible]	[illegible]	[illegible]	[illegible]	[illegible]	[illegible]	[illegible]	[illegible]	[illegible]	[illegible]	[illegible]	[illegible]
Q9DB77	[illegible]	[illegible]	[illegible]	[illegible]	[illegible]	[illegible]	[illegible]	[illegible]	[illegible]	[illegible]	[illegible]	[illegible]	[illegible]	[illegible]	[illegible]	[illegible]	[illegible]	[illegible]	[illegible]	[illegible]	[illegible]	[illegible]	[illegible]	[illegible]	[illegible]	[illegible]
P35979	[illegible]	[illegible]	[illegible]	[illegible]	[illegible]	[illegible]	[illegible]	[illegible]	[illegible]	[illegible]	[illegible]	[illegible]	[illegible]	[illegible]	[illegible]	[illegible]	[illegible]	[illegible]	[illegible]	[illegible]	[illegible]	[illegible]	[illegible]	[illegible]	[illegible]	[illegible]
Q8BGH2	[illegible]	[illegible]	[illegible]	[illegible]	[illegible]	[illegible]	[illegible]	[illegible]	[illegible]	[illegible]	[illegible]	[illegible]	[illegible]	[illegible]	[illegible]	[illegible]	[illegible]	[illegible]	[illegible]	[illegible]	[illegible]	[illegible]	[illegible]	[illegible]	[illegible]	[illegible]
P62082	[illegible]	[illegible]	[illegible]	[illegible]	[illegible]	[illegible]	[illegible]	[illegible]	[illegible]	[illegible]	[illegible]	[illegible]	[illegible]	[illegible]	[illegible]	[illegible]	[illegible]	[illegible]	[illegible]	[illegible]	[illegible]	[illegible]	[illegible]	[illegible]	[illegible]	[illegible]
P14824	[illegible]	[illegible]	[illegible]	[illegible]	[illegible]	[illegible]	[illegible]	[illegible]	[illegible]	[illegible]	[illegible]	[illegible]	[illegible]	[illegible]	[illegible]	[illegible]	[illegible]	[illegible]	[illegible]	[illegible]	[illegible]	[illegible]	[illegible]	[illegible]	[illegible]	[illegible]
P27773	[illegible]	[illegible]	[illegible]	[illegible]	[illegible]	[illegible]	[illegible]	[illegible]	[illegible]	[illegible]	[illegible]	[illegible]	[illegible]	[illegible]	[illegible]	[illegible]	[illegible]	[illegible]	[illegible]	[illegible]	[illegible]	[illegible]	[illegible]	[illegible]	[illegible]	[illegible]
P60867	[illegible]	[illegible]	[illegible]	[illegible]	[illegible]	[illegible]	[illegible]	[illegible]	[illegible]	[illegible]	[illegible]	[illegible]	[illegible]	[illegible]	[illegible]	[illegible]	[illegible]	[illegible]	[illegible]	[illegible]	[illegible]	[illegible]	[illegible]	[illegible]	[illegible]	[illegible]
Q91WD5	[illegible]	[illegible]	[illegible]	[illegible]	[illegible]	[illegible]	[illegible]	[illegible]	[illegible]	[illegible]	[illegible]	[illegible]	[illegible]	[illegible]	[illegible]	[illegible]	[illegible]	[illegible]	[illegible]	[illegible]	[illegible]	[illegible]	[illegible]	[illegible]	[illegible]	[illegible]
P41216	[illegible]	[illegible]	[illegible]	[illegible]	[illegible]	[illegible]	[illegible]	[illegible]	[illegible]	[illegible]	[illegible]	[illegible]	[illegible]	[illegible]	[illegible]	[illegible]	[illegible]	[illegible]	[illegible]	[illegible]	[illegible]	[illegible]	[illegible]	[illegible]	[illegible]	[illegible]
Q9CZ13	[illegible]	[illegible]	[illegible]	[illegible]	[illegible]	[illegible]	[illegible]	[illegible]	[illegible]	[illegible]	[illegible]	[illegible]	[illegible]	[illegible]	[illegible]	[illegible]	[illegible]	[illegible]	[illegible]	[illegible]	[illegible]	[illegible]	[illegible]	[illegible]	[illegible]	[illegible]
O35658	[illegible]	[illegible]	[illegible]	[illegible]	[illegible]	[illegible]	[illegible]	[illegible]	[illegible]	[illegible]	[illegible]	[illegible]	[illegible]	[illegible]	[illegible]	[illegible]	[illegible]	[illegible]	[illegible]	[illegible]	[illegible]	[illegible]	[illegible]	[illegible]	[illegible]	[illegible]
P16125	[illegible]	[illegible]	[illegible]	[illegible]	[illegible]	[illegible]	[illegible]	[illegible]	[illegible]	[illegible]	[illegible]	[illegible]	[illegible]	[illegible]	[illegible]	[illegible]	[illegible]	[illegible]	[illegible]	[illegible]	[illegible]	[illegible]	[illegible]	[illegible]	[illegible]	[illegible]
Q8JZQ2	[illegible]	[illegible]	[illegible]	[illegible]	[illegible]	[illegible]	[illegible]	[illegible]	[illegible]	[illegible]	[illegible]	[illegible]	[illegible]	[illegible]	[illegible]	[illegible]	[illegible]	[illegible]	[illegible]	[illegible]	[illegible]	[illegible]	[illegible]	[illegible]	[illegible]	[illegible]
P01942	[illegible]	[illegible]	[illegible]	[illegible]	[illegible]	[illegible]	[illegible]	[illegible]	[illegible]	[illegible]	[illegible]	[illegible]	[illegible]	[illegible]	[illegible]	[illegible]	[illegible]	[illegible]	[illegible]	[illegible]	[illegible]	[illegible]	[illegible]	[illegible]	[illegible]	[illegible]
P07724	[illegible]	[illegible]	[illegible]	[illegible]	[illegible]	[illegible]	[illegible]	[illegible]	[illegible]	[illegible]	[illegible]	[illegible]	[illegible]	[illegible]	[illegible]	[illegible]	[illegible]	[illegible]	[illegible]	[illegible]	[illegible]	[illegible]	[illegible]	[illegible]	[illegible]	[illegible]
P04247	[illegible]	[illegible]	[illegible]	[illegible]	[illegible]	[illegible]	[illegible]	[illegible]	[illegible]	[illegible]	[illegible]	[illegible]	[illegible]	[illegible]	[illegible]	[illegible]	[illegible]	[illegible]	[illegible]	[illegible]	[illegible]	[illegible]	[illegible]	[illegible]	[illegible]	[illegible]
P68134	[illegible]	[illegible]	[illegible]	[illegible]	[illegible]	[illegible]	[illegible]	[illegible]	[illegible]	[illegible]	[illegible]	[illegible]	[illegible]	[illegible]	[illegible]	[illegible]	[illegible]	[illegible]	[illegible]	[illegible]	[illegible]	[illegible]	[illegible]	[illegible]	[illegible]	[illegible]
Q9ERS2	[illegible]	[illegible]	[illegible]	[illegible]	[illegible]	[illegible]	[illegible]	[illegible]	[illegible]	[illegible]	[illegible]	[illegible]	[illegible]	[illegible]	[illegible]	[illegible]	[illegible]	[illegible]	[illegible]	[illegible]	[illegible]	[illegible]	[illegible]	[illegible]	[illegible]	[illegible]
P62897	[illegible]	[illegible]	[illegible]	[illegible]	[illegible]	[illegible]	[illegible]	[illegible]	[illegible]	[illegible]	[illegible]	[illegible]	[illegible]	[illegible]	[illegible]	[illegible]	[illegible]	[illegible]	[illegible]	[illegible]	[illegible]	[illegible]	[illegible]	[illegible]	[illegible]	[illegible]
Q60936	[illegible]	[illegible]	[illegible]	[illegible]	[illegible]	[illegible]	[illegible]	[illegible]	[illegible]	[illegible]	[illegible]	[illegible]	[illegible]	[illegible]	[illegible]	[illegible]	[illegible]	[illegible]	[illegible]	[illegible]	[illegible]	[illegible]	[illegible]	[illegible]	[illegible]	[illegible]
Q64475	[illegible]	[illegible]	[illegible]	[illegible]	[illegible]	[illegible]	[illegible]	[illegible]	[illegible]	[illegible]	[illegible]	[illegible]	[illegible]	[illegible]	[illegible]	[illegible]	[illegible]	[illegible]	[illegible]	[illegible]	[illegible]	[illegible]	[illegible]	[illegible]	[illegible]	[illegible]
Q9Z2I9	[illegible]	[illegible]	[illegible]	[illegible]	[illegible]	[illegible]	[illegible]	[illegible]	[illegible]	[illegible]	[illegible]	[illegible]	[illegible]	[illegible]	[illegible]	[illegible]	[illegible]	[illegible]	[illegible]	[illegible]	[illegible]	[illegible]	[illegible]	[illegible]	[illegible]	[illegible]
P05202	[illegible]	[illegible]	[illegible]	[illegible]	[illegible]	[illegible]	[illegible]	[illegible]	[illegible]	[illegible]	[illegible]	[illegible]	[illegible]	[illegible]	[illegible]	[illegible]	[illegible]	[illegible]	[illegible]	[illegible]	[illegible]	[illegible]	[illegible]	[illegible]	[illegible]	[illegible]
Q8BWT1	[illegible]	[illegible]	[illegible]	[illegible]	[illegible]	[illegible]	[illegible]	[illegible]	[illegible]	[illegible]	[illegible]	[illegible]	[illegible]	[illegible]	[illegible]	[illegible]	[illegible]	[illegible]	[illegible]	[illegible]	[illegible]	[illegible]	[illegible]	[illegible]	[illegible]	[illegible]
P61089	[illegible]	[illegible]	[illegible]	[illegible]	[illegible]	[illegible]	[illegible]	[illegible]	[illegible]	[illegible]	[illegible]	[illegible]	[illegible]	[illegible]	[illegible]	[illegible]	[illegible]	[illegible]	[illegible]	[illegible]	[illegible]	[illegible]	[illegible]	[illegible]	[illegible]	[illegible]
O54724	[illegible]	[illegible]	[illegible]	[illegible]	[illegible]	[illegible]	[illegible]	[illegible]	[illegible]	[illegible]	[illegible]	[illegible]	[illegible]	[illegible]	[illegible]	[illegible]	[illegible]	[illegible]	[illegible]	[illegible]	[illegible]	[illegible]	[illegible]	[illegible]	[illegible]	[illegible]
Q9CQQ7	[illegible]	[illegible]	[illegible]	[illegible]	[illegible]	[illegible]	[illegible]	[illegible]	[illegible]	[illegible]	[illegible]	[illegible]	[illegible]	[illegible]	[illegible]	[illegible]	[illegible]	[illegible]	[illegible]	[illegible]	[illegible]	[illegible]	[illegible]	[illegible]	[illegible]	[illegible]
P24270	[illegible]	[illegible]	[illegible]	[illegible]	[illegible]	[illegible]	[illegible]	[illegible]	[illegible]	[illegible]	[illegible]	[illegible]	[illegible]	[illegible]	[illegible]	[illegible]	[illegible]	[illegible]	[illegible]	[illegible]	[illegible]	[illegible]	[illegible]	[illegible]	[illegible]	[illegible]
Q07417	[illegible]	[illegible]	[illegible]	[illegible]	[illegible]	[illegible]	[illegible]	[illegible]	[illegible]	[illegible]	[illegible]	[illegible]	[illegible]	[illegible]	[illegible]	[illegible]	[illegible]	[illegible]	[illegible]	[illegible]	[illegible]	[illegible]	[illegible]	[illegible]	[illegible]	[illegible]
Q9CR68	[illegible]	[illegible]	[illegible]	[illegible]	[illegible]	[illegible]	[illegible]	[illegible]	[illegible]	[illegible]	[illegible]	[illegible]	[illegible]	[illegible]	[illegible]	[illegible]	[illegible]	[illegible]	[illegible]	[illegible]	[illegible]	[illegible]	[illegible]	[illegible]	[illegible]	[illegible]
Q8BFR5	[illegible]	[illegible]	[illegible]	[illegible]	[illegible]	[illegible]	[illegible]	[illegible]	[illegible]	[illegible]	[illegible]	[illegible]	[illegible]	[illegible]	[illegible]	[illegible]	[illegible]	[illegible]	[illegible]	[illegible]	[illegible]	[illegible]	[illegible]	[illegible]	[illegible]	[illegible]
Q9D8B4	[illegible]	[illegible]	[illegible]	[illegible]	[illegible]	[illegible]	[illegible]	[illegible]	[illegible]	[illegible]	[illegible]	[illegible]	[illegible]	[illegible]	[illegible]	[illegible]	[illegible]	[illegible]	[illegible]	[illegible]	[illegible]	[illegible]	[illegible]	[illegible]	[illegible]	[illegible]
Q08857	[illegible]	[illegible]	[illegible]	[illegible]	[illegible]	[illegible]	[illegible]	[illegible]	[illegible]	[illegible]	[illegible]	[illegible]	[illegible]	[illegible]	[illegible]	[illegible]	[illegible]	[illegible]	[illegible]	[illegible]	[illegible]	[illegible]	[illegible]	[illegible]	[illegible]	[illegible]
Q9CR62	[illegible]	[illegible]	[illegible]	[illegible]	[illegible]	[illegible]	[illegible]	[illegible]	[illegible]	[illegible]	[illegible]	[illegible]	[illegible]	[illegible]	[illegible]	[illegible]	[illegible]	[illegible]	[illegible]	[illegible]	[illegible]	[illegible]	[illegible]	[illegible]	[illegible]	[illegible]
Q8BFZ9	[illegible]	[illegible]	[illegible]	[illegible]	[illegible]	[illegible]	[illegible]	[illegible]	[illegible]	[illegible]	[illegible]	[illegible]	[illegible]	[illegible]	[illegible]	[illegible]	[illegible]	[illegible]	[illegible]	[illegible]	[illegible]	[illegible]	[illegible]	[illegible]	[illegible]	[illegible]
P67778	[illegible]	[illegible]	[illegible]	[illegible]	[illegible]	[illegible]	[illegible]	[illegible]	[illegible]	[illegible]	[illegible]	[illegible]	[illegible]	[illegible]	[illegible]	[illegible]	[illegible]	[illegible]	[illegible]	[illegible]	[illegible]	[illegible]	[illegible]	[illegible]	[illegible]	[illegible]
P51667	[illegible]	[illegible]	[illegible]	[illegible]	[illegible]	[illegible]	[illegible]	[illegible]	[illegible]	[illegible]	[illegible]	[illegible]	[illegible]	[illegible]	[illegible]	[illegible]	[illegible]	[illegible]	[illegible]	[illegible]	[illegible]	[illegible]	[illegible]	[illegible]	[illegible]	[illegible]
Q6P8J7	[illegible]	[illegible]	[illegible]	[illegible]	[illegible]	[illegible]	[illegible]	[illegible]	[illegible]	[illegible]	[illegible]	[illegible]	[illegible]	[illegible]	[illegible]	[illegible]	[illegible]	[illegible]	[illegible]	[illegible]	[illegible]	[illegible]	[illegible]	[illegible]	[illegible]	[illegible]
Q61941	[illegible]	[illegible]	[illegible]	[illegible]	[illegible]	[illegible]	[illegible]	[illegible]	[illegible]	[illegible]	[illegible]	[illegible]	[illegible]	[illegible]	[illegible]	[illegible]	[illegible]	[illegible]	[illegible]	[illegible]	[illegible]	[illegible]	[illegible]	[illegible]	[illegible]	[illegible]

Swiss-Prot ID	Protein Names	Protein Code
P58771	TPM1_MOUSE, SP:P58771	SP|P58771|TPM1_MOUSE
P58281	OPA1_MOUSE, SP:P58281	SP|P58281|OPA1_MOUSE
P63038	CH60_MOUSE, SP:P63038	SP|P63038|CH60_MOUSE
Q8VEM8	MPCP_MOUSE, SP:Q8VEM8	SP|Q8VEM8|MPCP_MOUSE
O70468	MYPC3_MOUSE, SP:O70468	SP|O70468|MYPC3_MOUSE
Q9D855	QCR7_MOUSE, SP:Q9D855	SP|Q9D855|QCR7_MOUSE
Q9CQZ5	NDUA6_MOUSE, SP:Q9CQZ5	SP|Q9CQZ5|NDUA6_MOUSE
Q9Z0X1	AIFM1_MOUSE, SP:Q9Z0X1	SP|Q9Z0X1|AIFM1_MOUSE
P14206	RSSA_MOUSE, SP:P14206	SP|P14206|RSSA_MOUSE
Q99JY0	ECHB_MOUSE, SP:Q99JY0	SP|Q99JY0|ECHB_MOUSE
Q91VD9	NDUS1_MOUSE, SP:Q91VD9	SP|Q91VD9|NDUS1_MOUSE
P47738	ALDH2_MOUSE, SP:P47738	SP|P47738|ALDH2_MOUSE
P56480	ATPB_MOUSE, SP:P56480	SP|P56480|ATPB_MOUSE
P48962	ADT1_MOUSE, SP:P48962	SP|P48962|ADT1_MOUSE
P63017	HSP7C_MOUSE, SP:P63017	SP|P63017|HSP7C_MOUSE
O35459	ECH1_MOUSE, SP:O35459	SP|O35459|ECH1_MOUSE
Q02566	MYH6_MOUSE, SP:Q02566	SP|Q02566|MYH6_MOUSE
P54071	IDHP_MOUSE, SP:P54071	SP|P54071|IDHP_MOUSE
P62900	RL31_MOUSE, SP:P62900	SP|P62900|RL31_MOUSE
P16858	G3P_MOUSE, SP:P16858	SP|P16858|G3P_MOUSE
Q91YT0	NDUV1_MOUSE, SP:Q91YT0	SP|Q91YT0|NDUV1_MOUSE
P01887	B2MG_MOUSE, SP:P01887	SP|P01887|B2MG_MOUSE
Q9CR61	NDUB7_MOUSE, SP:Q9CR61	SP|Q9CR61|NDUB7_MOUSE
P47911	RL6_MOUSE, SP:P47911	SP|P47911|RL6_MOUSE
Q9D0K2	SCOT1_MOUSE, SP:Q9D0K2	SP|Q9D0K2|SCOT1_MOUSE
Q7TQ48	SRCA_MOUSE, SP:Q7TQ48	SP|Q7TQ48|SRCA_MOUSE
Q8CAQ8	IMMT_MOUSE, SP:Q8CAQ8	SP|Q8CAQ8|IMMT_MOUSE
Q01853	TERA_MOUSE, SP:Q01853	SP|Q01853|TERA_MOUSE
P17710	HXK1_MOUSE, SP:P17710	SP|P17710|HXK1_MOUSE
Q99LC5	ETFA_MOUSE, SP:Q99LC5	SP|Q99LC5|ETFA_MOUSE
Q8K1Z0	COQ9_MOUSE, SP:Q8K1Z0	SP|Q8K1Z0|COQ9_MOUSE
P12787	COX5A_MOUSE, SP:P12787	SP|P12787|COX5A_MOUSE
Q9D1G3	HHATL_MOUSE, SP:Q9D1G3	SP|Q9D1G3|HHATL_MOUSE
Q2TPA8	HSDL2_MOUSE, SP:Q2TPA8	SP|Q2TPA8|HSDL2_MOUSE
P99029	PRDX5_MOUSE, SP:P99029	SP|P99029|PRDX5_MOUSE
Q8VDN2	AT1A1_MOUSE, SP:Q8VDN2	SP|Q8VDN2|AT1A1_MOUSE
Q9CQA3	DHSB_MOUSE, SP:Q9CQA3	SP|Q9CQA3|DHSB_MOUSE
P97807	FUMH_MOUSE, SP:P97807	SP|P97807|FUMH_MOUSE
Q9DB20	ATPO_MOUSE, SP:Q9DB20	SP|Q9DB20|ATPO_MOUSE
P26443	DHE3_MOUSE, SP:P26443	SP|P26443|DHE3_MOUSE
P06151	LDHA_MOUSE, SP:P06151	SP|P06151|LDHA_MOUSE
Q8BH95	ECHM_MOUSE, SP:Q8BH95	SP|Q8BH95|ECHM_MOUSE
Q03265	ATPA_MOUSE, SP:Q03265	SP|Q03265|ATPA_MOUSE
O35129	PHB2_MOUSE, SP:O35129	SP|O35129|PHB2_MOUSE
Q921G7	ETFD_MOUSE, SP:Q921G7	SP|Q921G7|ETFD_MOUSE
Q9CPU4	MGST3_MOUSE, SP:Q9CPU4	SP|Q9CPU4|MGST3_MOUSE
P03903	NU4LM_MOUSE, SP:P03903	SP|P03903|NU4LM_MOUSE
Q99KI0	ACON_MOUSE, SP:Q99KI0	SP|Q99KI0|ACON_MOUSE
P57776	EF1D_MOUSE, SP:P57776	SP|P57776|EF1D_MOUSE
P20029	GRP78_MOUSE, SP:P20029	SP|P20029|GRP78_MOUSE
P67984	RL22_MOUSE, SP:P67984	SP|P67984|RL22_MOUSE
P35486	ODPA_MOUSE, SP:P35486	SP|P35486|ODPA_MOUSE
Q01768	NDKB_MOUSE, SP:Q01768	SP|Q01768|NDKB_MOUSE
O55143	AT2A2_MOUSE, SP:O55143	SP|O55143|AT2A2_MOUSE
Q3UIU2	NDUB6_MOUSE, SP:Q3UIU2	SP|Q3UIU2|NDUB6_MOUSE
P45952	ACADM_MOUSE, SP:P45952	SP|P45952|ACADM_MOUSE
Q60932	VDAC1_MOUSE, SP:Q60932	SP|Q60932|VDAC1_MOUSE
P03888	NU1M_MOUSE, SP:P03888	SP|P03888|NU1M_MOUSE
Q8BH59	CMC1_MOUSE, SP:Q8BH59	SP|Q8BH59|CMC1_MOUSE
Q60930	VDAC2_MOUSE, SP:Q60930	SP|Q60930|VDAC2_MOUSE
P50462	CSRP3_MOUSE, SP:P50462	SP|P50462|CSRP3_MOUSE
P03921	NU5M_MOUSE, SP:P03921	SP|P03921|NU5M_MOUSE
Q9DCZ4	APOO_MOUSE, SP:Q9DCZ4	SP|Q9DCZ4|APOO_MOUSE
Q8K2B3	DHSA_MOUSE, SP:Q8K2B3	SP|Q8K2B3|DHSA_MOUSE
Q9JHI5	IVD_MOUSE, SP:Q9JHI5	SP|Q9JHI5|IVD_MOUSE
O08749	DLDH_MOUSE, SP:O08749	SP|O08749|DLDH_MOUSE
Q8CC88	K0564_MOUSE, SP:Q8CC88	SP|Q8CC88|K0564_MOUSE
P11152	LIPL_MOUSE, SP:P11152	SP|P11152|LIPL_MOUSE
P52825	CPT2_MOUSE, SP:P52825	SP|P52825|CPT2_MOUSE
P50544	ACADV_MOUSE, SP:P50544	SP|P50544|ACADV_MOUSE
Q8BMS1	ECHA_MOUSE, SP:Q8BMS1	SP|Q8BMS1|ECHA_MOUSE
Q61554	FBN1_MOUSE, SP:Q61554	SP|Q61554|FBN1_MOUSE
Q8BMF4	ODP2_MOUSE, SP:Q8BMF4	SP|Q8BMF4|ODP2_MOUSE
O08756	HCD2_MOUSE, SP:O08756	SP|O08756|HCD2_MOUSE
Q8BK30	NDUV3_MOUSE, SP:Q8BK30	SP|Q8BK30|NDUV3_MOUSE
P50752	TNNT2_MOUSE, SP:P50752	SP|P50752|TNNT2_MOUSE
P56391	CX6B1_MOUSE, SP:P56391	SP|P56391|CX6B1_MOUSE
Q9D051	ODPB_MOUSE, SP:Q9D051	SP|Q9D051|ODPB_MOUSE
P09542	MYL3_MOUSE, SP:P09542	SP|P09542|MYL3_MOUSE
Q60597	ODO1_MOUSE, SP:Q60597	SP|Q60597|ODO1_MOUSE
P10605	CATB_MOUSE, SP:P10605	SP|P10605|CATB_MOUSE
Q9DB77	QCR2_MOUSE, SP:Q9DB77	SP|Q9DB77|QCR2_MOUSE
P35979	RL12_MOUSE, SP:P35979	SP|P35979|RL12_MOUSE
Q8BGH2	SAM50_MOUSE, SP:Q8BGH2	SP|Q8BGH2|SAM50_MOUSE
P62082	RS7_MOUSE, SP:P62082	SP|P62082|RS7_MOUSE
P14824	ANXA6_MOUSE, SP:P14824	SP|P14824|ANXA6_MOUSE
P27773	PDIA3_MOUSE, SP:P27773	SP|P27773|PDIA3_MOUSE
P60867	RS20_MOUSE, SP:P60867	SP|P60867|RS20_MOUSE
Q91WD5	NDUS2_MOUSE, SP:Q91WD5	SP|Q91WD5|NDUS2_MOUSE
P41216	ACSL1_MOUSE, SP:P41216	SP|P41216|ACSL1_MOUSE
Q9CZ13	QCR1_MOUSE, SP:Q9CZ13	SP|Q9CZ13|QCR1_MOUSE
O35658	C1QBP_MOUSE, SP:O35658	SP|O35658|C1QBP_MOUSE
P16125	LDHB_MOUSE, SP:P16125	SP|P16125|LDHB_MOUSE
Q8JZQ2	AFG32_MOUSE, SP:Q8JZQ2	SP|Q8JZQ2|AFG32_MOUSE
P01942	HBA_MOUSE, SP:P01942	SP|P01942|HBA_MOUSE
P07724	ALBU_MOUSE, SP:P07724	SP|P07724|ALBU_MOUSE
P04247	MYG_MOUSE, SP:P04247	SP|P04247|MYG_MOUSE
P68134	ACTS_MOUSE, SP:P68134	SP|P68134|ACTS_MOUSE
Q9ERS2	NDUAD_MOUSE, SP:Q9ERS2	SP|Q9ERS2|NDUAD_MOUSE
P62897	CYC_MOUSE, SP:P62897	SP|P62897|CYC_MOUSE
Q60936	ADCK3_MOUSE, SP:Q60936	SP|Q60936|ADCK3_MOUSE
Q64475	H2B1P_MOUSE, SP:Q8CGP2	SP|Q8CGP2|H2B1P_MOUSE,SP|Q64475|H2B1B_MOUSE,SP|P10853|H2B1F_MOUSE,S[illegible]
Q9Z2I9	SUCB1_MOUSE, SP:Q9Z2I9	SP|Q9Z2I9|SUCB1_MOUSE
P05202	AATM_MOUSE, SP:P05202	SP|P05202|AATM_MOUSE
Q8BWT1	THIM_MOUSE, SP:Q8BWT1	SP|Q8BWT1|THIM_MOUSE
P61089	UBE2N_MOUSE, SP:P61089	SP|P61089|UBE2N_MOUSE
O54724	PTRF_MOUSE, SP:O54724	SP|O54724|PTRF_MOUSE
Q9CQQ7	AT5F1_MOUSE, SP:Q9CQQ7	SP|Q9CQQ7|AT5F1_MOUSE
P24270	CATA_MOUSE, SP:P24270	SP|P24270|CATA_MOUSE
Q07417	ACADS_MOUSE, SP:Q07417	SP|Q07417|ACADS_MOUSE
Q9CR68	UCRI_MOUSE, SP:Q9CR68	SP|Q9CR68|UCRI_MOUSE
Q8BFR5	EFTU_MOUSE, SP:Q8BFR5	SP|Q8BFR5|EFTU_MOUSE
Q9D8B4	NDUAB_MOUSE, SP:Q9D8B4	SP|Q9D8B4|NDUAB_MOUSE
Q08857	CD36_MOUSE, SP:Q08857	SP|Q08857|CD36_MOUSE
Q9CR62	M2OM_MOUSE, SP:Q9CR62	SP|Q9CR62|M2OM_MOUSE
Q8BFZ9	ERLN2_MOUSE, SP:Q8BFZ9	SP|Q8BFZ9|ERLN2_MOUSE
P67778	PHB_MOUSE, SP:P67778	SP|P67778|PHB_MOUSE
P51667	MLRV_MOUSE, SP:P51667	SP|P51667|MLRV_MOUSE
Q6P8J7	KCRS_MOUSE, SP:Q6P8J7	SP|Q6P8J7|KCRS_MOUSE
Q61941	NNTM_MOUSE, SP:Q61941	SP|Q61941|NNTM_MOUSE

Q1XH17	TRI72_MOUSE	Tripartite motif-containing protein 72 OS=Mus musculus GN=Trim72 PE=1 SV=1 [MASS=52816]	13	13	1	3.83E+06	[illegible]
P62242	RS8_MOUSE	40S ribosomal protein S8 OS=Mus musculus GN=Rps8 PE=1 SV=2 [MASS=24205]	3	8	1	1.04E+05	[illegible]
Q9Z2I0	LETM1_MOUSE	LETM1 and EF-hand domain-containing protein 1, mitochondrial OS=Mus musculus GN=Letm1 PE=2 SV=1 [MASS=82988]	7	13	1	8.50E+05	[illegible]
Q9CQH3	NDUB5_MOUSE	NADH dehydrogenase [ubiquinone] 1 beta subcomplex subunit 5, mitochondrial OS=Mus musculus GN=Ndufb5 PE=1 SV=1 [MASS=21710]	3	13	1	7.21E+05	[illegible]
Q9WTR5	CAD13_MOUSE	Cadherin-13 OS=Mus musculus GN=Cdh13 PE=2 SV=1 [MASS=78285]	4	13	1	1.01E+06	[illegible]
Q9CRB9	CHCH3_MOUSE	Coiled-coil-helix-coiled-coil-helix domain-containing protein 3, mitochondrial OS=Mus musculus GN=Chchd3 PE=1 SV=1 [MASS=26334]	3	13	1	1.58E+06	[illegible]
Q9CQ75	NDUA2_MOUSE	NADH dehydrogenase [ubiquinone] 1 alpha subcomplex subunit 2 OS=Mus musculus GN=Ndufa2 PE=1 SV=3 [MASS=10916]	3	13	1	2.74E+06	[illegible]
Q791V5	MTCH2_MOUSE	Mitochondrial carrier homolog 2 OS=Mus musculus GN=Mtch2 PE=1 SV=1 [MASS=33499]	8	13	1	1.29E+06	[illegible]
Q9QYG0	NDRG2_MOUSE	Protein NDRG2 OS=Mus musculus GN=Ndrg2 PE=1 SV=1 [MASS=40789]	11	13	1	5.19E+06	[illegible]
Q9D0M3	CY1_MOUSE	Cytochrome c1, heme protein, mitochondrial OS=Mus musculus GN=Cyc1 PE=1 SV=1 [MASS=35327]	10	13	1	1.29E+07	[illegible]
Q99LY9	NDUS5_MOUSE	NADH dehydrogenase [ubiquinone] iron-sulfur protein 5 OS=Mus musculus GN=Ndufs5 PE=1 SV=3 [MASS=12648]	3	13	1	2.56E+05	[illegible]
Q924X2	CPT1B_MOUSE	Carnitine O-palmitoyltransferase 1, muscle isoform OS=Mus musculus GN=Cpt1b PE=2 SV=1 [MASS=88217]	19	13	1	7.18E+06	[illegible]
Q8QZT1	THIL_MOUSE	Acetyl-CoA acetyltransferase, mitochondrial OS=Mus musculus GN=Acat1 PE=1 SV=1 [MASS=44816]	14	13	1	2.06E+07	[illegible]
Q80XN0	BDH_MOUSE	D-beta-hydroxybutyrate dehydrogenase, mitochondrial OS=Mus musculus GN=Bdh1 PE=1 SV=1 [MASS=38285]	8	12	1	5.14E+05	[illegible]
P47934	CACP_MOUSE	Carnitine O-acetyltransferase OS=Mus musculus GN=Crat PE=1 SV=2 [MASS=70924]	17	13	1	4.07E+06	[illegible]
P09671	SODM_MOUSE	Superoxide dismutase [Mn], mitochondrial OS=Mus musculus GN=Sod2 PE=1 SV=3 [MASS=24603]	5	13	1	7.90E+06	[illegible]
P14869	RLA0_MOUSE	60S acidic ribosomal protein P0 OS=Mus musculus GN=Rplp0 PE=1 SV=3 [MASS=34216]	5	13	1	4.43E+05	[illegible]
Q9CQJ8	NDUB9_MOUSE	NADH dehydrogenase [ubiquinone] 1 beta subcomplex subunit 9 OS=Mus musculus GN=Ndufb9 PE=1 SV=3 [MASS=21984]	6	13	1	2.43E+06	[illegible]
P62858	RS28_MOUSE	40S ribosomal protein S28 OS=Mus musculus GN=Rps28 PE=2 SV=1 [MASS=7841]	2	13	1	1.98E+05	[illegible]
Q9CZU6	CISY_MOUSE	Citrate synthase, mitochondrial OS=Mus musculus GN=Cs PE=1 SV=1 [MASS=51736]	15	13	1	2.70E+07	[illegible]
P04117	FABP4_MOUSE	Fatty acid-binding protein, adipocyte OS=Mus musculus GN=Fabp4 PE=1 SV=3 [MASS=14650]	4	13	1	6.74E+06	[illegible]
P19783	COX41_MOUSE	Cytochrome c oxidase subunit 4 isoform 1, mitochondrial OS=Mus musculus GN=Cox4i1 PE=1 SV=2 [MASS=19530]	8	13	1	2.93E+07	[illegible]
P19536	COX5B_MOUSE	Cytochrome c oxidase subunit 5B, mitochondrial OS=Mus musculus GN=Cox5b PE=1 SV=1 [MASS=13813]	4	13	1	5.11E+06	[illegible]
O70250	PGAM2_MOUSE	Phosphoglycerate mutase 2 OS=Mus musculus GN=Pgam2 PE=1 SV=3 [MASS=28827]	7	13	1	6.08E+06	[illegible]
Q99JB8	PACN3_MOUSE	Protein kinase C and casein kinase II substrate protein 3 OS=Mus musculus GN=Pacsin3 PE=1 SV=1 [MASS=48585]	3	3	1	5.80E+04	[illegible]
Q9DCT2	NDUS3_MOUSE	NADH dehydrogenase [ubiquinone] iron-sulfur protein 3, mitochondrial OS=Mus musculus GN=Ndufs3 PE=1 SV=2 [MASS=30149]	8	13	1	7.69E+06	[illegible]
P48771	CX7A2_MOUSE	Cytochrome c oxidase polypeptide 7A2, mitochondrial OS=Mus musculus GN=Cox7a2 PE=1 SV=2 [MASS=9291]	2	13	1	1.50E+06	[illegible]
Q91VR2	ATPG_MOUSE	ATP synthase subunit gamma, mitochondrial OS=Mus musculus GN=Atp5c1 PE=1 SV=1 [MASS=32886]	7	13	1	1.03E+07	[illegible]
Q9QXX4	CMC2_MOUSE	Calcium-binding mitochondrial carrier protein Aralar2 OS=Mus musculus GN=Slc25a13 PE=1 SV=1 [MASS=74466]	17	13	1	4.13E+06	[illegible]
P51881	ADT2_MOUSE	ADP/ATP translocase 2 OS=Mus musculus GN=Slc25a5 PE=1 SV=3 [MASS=32931]	6	13	1	1.29E+06	[illegible]
P58252	EF2_MOUSE	Elongation factor 2 OS=Mus musculus GN=Eef2 PE=1 SV=2 [MASS=95313]	14	13	1	2.25E+06	[illegible]
Q99LB2	DHRS4_MOUSE	Dehydrogenase/reductase SDR family member 4 OS=Mus musculus GN=Dhrs4 PE=2 SV=1 [MASS=27754]	4	13	1	4.01E+05	[illegible]
Q61387	COX7R_MOUSE	Cytochrome c oxidase subunit 7A-related protein, mitochondrial OS=Mus musculus GN=Cox7a2l PE=2 SV=1 [MASS=12399]	1	13	0.99	6.86E+04	[illegible]
P38647	GRP75_MOUSE	Stress-70 protein, mitochondrial OS=Mus musculus GN=Hspa9 PE=1 SV=2 [MASS=73528]	19	13	1	1.01E+07	[illegible]
Q7TMF3	NDUAC_MOUSE	NADH dehydrogenase [ubiquinone] 1 alpha subcomplex subunit 12 OS=Mus musculus GN=Ndufa12 PE=1 SV=2 [MASS=17086]	6	13	1	3.66E+06	[illegible]
Q9DCS9	NDUBA_MOUSE	NADH dehydrogenase [ubiquinone] 1 beta subcomplex subunit 10 OS=Mus musculus GN=Ndufb10 PE=1 SV=3 [MASS=21024]	6	13	1	6.89E+06	[illegible]
Q8VCW8	ACSF2_MOUSE	Acyl-CoA synthetase family member 2, mitochondrial OS=Mus musculus GN=Acsf2 PE=2 SV=1 [MASS=67950]	2	9	1	1.06E+05	[illegible]
P14094	AT1B1_MOUSE	Sodium/potassium-transporting ATPase subunit beta-1 OS=Mus musculus GN=Atp1b1 PE=1 SV=1 [MASS=35194]	5	13	1	2.30E+06	[illegible]
O09161	CASQ2_MOUSE	Calsequestrin-2 OS=Mus musculus GN=Casq2 PE=2 SV=2 [MASS=48197]	10	13	1	8.04E+06	[illegible]
Q61207	SAP_MOUSE	Sulfated glycoprotein 1 OS=Mus musculus GN=Psap PE=1 SV=2 [MASS=61422]	2	13	1	1.20E+05	[illegible]
Q8R404	QIL1_MOUSE	Protein QIL1 OS=Mus musculus GN=Qil1 PE=2 SV=1 [MASS=13373]	3	12	1	1.28E+06	[illegible]
P17751	TPIS_MOUSE	Triosephosphate isomerase OS=Mus musculus GN=Tpi1 PE=1 SV=3 [MASS=26712]	8	13	1	1.32E+07	[illegible]
Q8BKZ9	ODPX_MOUSE	Pyruvate dehydrogenase protein X component, mitochondrial OS=Mus musculus GN=Pdhx PE=2 SV=1 [MASS=53999]	8	13	1	1.48E+06	[illegible]
P80317	TCPZ_MOUSE	T-complex protein 1 subunit zeta OS=Mus musculus GN=Cct6a PE=1 SV=3 [MASS=58004]	2	10	1	8.06E+04	[illegible]
P62751	RL23A_MOUSE	60S ribosomal protein L23a OS=Mus musculus GN=Rpl23a PE=2 SV=1 [MASS=17695]	1	8	0.63	6.09E+05	[illegible]
Q9CPQ1	COX6C_MOUSE	Cytochrome c oxidase subunit 6C OS=Mus musculus GN=Cox6c PE=1 SV=3 [MASS=8469]	2	13	1	3.57E+06	[illegible]
P51174	ACADL_MOUSE	Long-chain specific acyl-CoA dehydrogenase, mitochondrial OS=Mus musculus GN=Acadl PE=2 SV=2 [MASS=47908]	10	13	1	3.11E+07	[illegible]
P08249	MDHM_MOUSE	Malate dehydrogenase, mitochondrial OS=Mus musculus GN=Mdh2 PE=1 SV=3 [MASS=35611]	14	13	1	4.64E+07	[illegible]
P26041	MOES_MOUSE	Moesin OS=Mus musculus GN=Msn PE=1 SV=3 [MASS=67767]	8	13	1	1.07E+06	[illegible]
P11499	HS90B_MOUSE	Heat shock protein HSP 90-beta OS=Mus musculus GN=Hsp90ab1 PE=1 SV=2 [MASS=83325]	12	13	1	3.36E+06	[illegible]
Q9Z2Z6	MCAT_MOUSE	Mitochondrial carnitine/acylcarnitine carrier protein OS=Mus musculus GN=Slc25a20 PE=1 SV=1 [MASS=33027]	7	13	1	1.75E+06	[illegible]
P11404	FABPH_MOUSE	Fatty acid-binding protein, heart OS=Mus musculus GN=Fabp3 PE=1 SV=5 [MASS=14819]	5	13	1	1.50E+07	[illegible]
Q9CQ62	DECR_MOUSE	2,4-dienoyl-CoA reductase, mitochondrial OS=Mus musculus GN=Decr1 PE=1 SV=1 [MASS=36214]	11	13	1	9.30E+06	[illegible]
Q78IK4	APOOL_MOUSE	Apolipoprotein O-like OS=Mus musculus GN=Apool PE=2 SV=1 [MASS=29261]	8	13	1	1.17E+06	[illegible]
P05064	ALDOA_MOUSE	Fructose-bisphosphate aldolase A OS=Mus musculus GN=Aldoa PE=1 SV=2 [MASS=39356]	11	13	1	1.83E+07	[illegible]
P49817	CAV1_MOUSE	Caveolin-1 OS=Mus musculus GN=Cav1 PE=1 SV=1 [MASS=20539]	4	13	1	1.45E+06	[illegible]
P52503	NDUS6_MOUSE	NADH dehydrogenase [ubiquinone] iron-sulfur protein 6, mitochondrial OS=Mus musculus GN=Ndufs6 PE=1 SV=2 [MASS=13020]	4	13	1	4.16E+06	[illegible]
Q99NB1	ACS2L_MOUSE	Acetyl-coenzyme A synthetase 2-like, mitochondrial OS=Mus musculus GN=Acss1 PE=1 SV=1 [MASS=74623]	12	13	1	3.65E+06	[illegible]
P62631	EF1A2_MOUSE	Elongation factor 1-alpha 2 OS=Mus musculus GN=Eef1a2 PE=1 SV=1 [MASS=50454]	5	13	1	6.96E+06	[illegible]
Q61425	HCDH_MOUSE	Hydroxyacyl-coenzyme A dehydrogenase, mitochondrial OS=Mus musculus GN=Hadh PE=1 SV=2 [MASS=34464]	8	13	1	1.28E+07	[illegible]
Q9CPP6	NDUA5_MOUSE	NADH dehydrogenase [ubiquinone] 1 alpha subcomplex subunit 5 OS=Mus musculus GN=Ndufa5 PE=1 SV=3 [MASS=13360]	4	13	1	1.20E+06	[illegible]
P18572	BASI_MOUSE	Basigin OS=Mus musculus GN=Bsg PE=1 SV=2 [MASS=42444]	4	13	1	1.31E+06	[illegible]
P14131	RS16_MOUSE	40S ribosomal protein S16 OS=Mus musculus GN=Rps16 PE=2 SV=4 [MASS=16445]	1	6	0.89	4.83E+04	[illegible]
P97450	ATP5J_MOUSE	ATP synthase-coupling factor 6, mitochondrial OS=Mus musculus GN=Atp5j PE=1 SV=1 [MASS=12496]	3	13	1	3.30E+06	[illegible]
Q922B1	MACD1_MOUSE	MACRO domain-containing protein 1 OS=Mus musculus GN=Macrod1 PE=2 SV=2 [MASS=35294]	5	13	1	1.59E+06	[illegible]
P00405	COX2_MOUSE	Cytochrome c oxidase subunit 2 OS=Mus musculus GN=Mtco2 PE=1 SV=1 [MASS=25976]	5	13	1	3.76E+07	[illegible]
Q9DCJ5	NDUA8_MOUSE	NADH dehydrogenase [ubiquinone] 1 alpha subcomplex subunit 8 OS=Mus musculus GN=Ndufa8 PE=1 SV=3 [MASS=19992]	5	13	1	8.44E+05	[illegible]
P62702	RS4X_MOUSE	40S ribosomal protein S4, X isoform OS=Mus musculus GN=Rps4x PE=2 SV=2 [MASS=29598]	2	12	1	1.37E+05	[illegible]
Q62425	NDUA4_MOUSE	NADH dehydrogenase [ubiquinone] 1 alpha subcomplex subunit 4 OS=Mus musculus GN=Ndufa4 PE=1 SV=2 [MASS=9327]	4	13	1	1.48E+07	[illegible]
P07310	KCRM_MOUSE	Creatine kinase M-type OS=Mus musculus GN=Ckm PE=1 SV=1 [MASS=43045]	12	13	1	3.45E+07	[illegible]
Q9CZW5	TOM70_MOUSE	Mitochondrial import receptor subunit TOM70 OS=Mus musculus GN=Tomm70a PE=1 SV=1 [MASS=67521]	3	7	1	4.50E+04	[illegible]
Q9D6U8	F162A_MOUSE	UPF0389 protein FAM162A OS=Mus musculus GN=Fam162a PE=2 SV=1 [MASS=17725]	2	13	1	3.01E+05	[illegible]
Q9CQF9	PCYOX_MOUSE	Prenylcysteine oxidase OS=Mus musculus GN=Pcyox1 PE=1 SV=1 [MASS=56494]	1	2	0.97	4.35E+04	[illegible]
Q9CQ69	QCR8_MOUSE	Cytochrome b-c1 complex subunit 8 OS=Mus musculus GN=Uqcrq PE=1 SV=3 [MASS=9768]	2	13	1	4.58E+05	[illegible]
P19123	TNNC1_MOUSE	Troponin C, slow skeletal and cardiac muscles OS=Mus musculus GN=Tnnc1 PE=2 SV=1 [MASS=18420]	4	13	1	1.88E+06	[illegible]
P68368	TBA4A_MOUSE	Tubulin alpha-4A chain OS=Mus musculus GN=Tuba4a PE=1 SV=1 [MASS=49924]	4	13	1	1.22E+06	[illegible]
Q9QYR9	ACOT2_MOUSE	Acyl-coenzyme A thioesterase 2, mitochondrial OS=Mus musculus GN=Acot2 PE=1 SV=1 [MASS=49652]	4	12	1	2.08E+06	[illegible]
O88441	MTX2_MOUSE	Metaxin-2 OS=Mus musculus GN=Mtx2 PE=1 SV=1 [MASS=29758]	2	13	1	5.14E+05	[illegible]
Q9CXV1	DHSD_MOUSE	Succinate dehydrogenase [ubiquinone] cytochrome b small subunit, mitochondrial OS=Mus musculus GN=Sdhd PE=2 SV=2 [MASS=17014]	2	13	1	4.59E+05	[illegible]
Q64433	CH10_MOUSE	10 kDa heat shock protein, mitochondrial OS=Mus musculus GN=Hspe1 PE=1 SV=2 [MASS=10963]	3	13	1	1.01E+06	[illegible]
Q06185	ATP5I_MOUSE	ATP synthase subunit e, mitochondrial OS=Mus musculus GN=Atp5i PE=1 SV=2 [MASS=8236]	2	8	1	7.09E+06	[illegible]
Q9D6J6	NDUV2_MOUSE	NADH dehydrogenase [ubiquinone] flavoprotein 2, mitochondrial OS=Mus musculus GN=Ndufv2 PE=1 SV=2 [MASS=27285]	8	13	1	3.94E+06	[illegible]
Q9CQ91	NDUA3_MOUSE	NADH dehydrogenase [ubiquinone] 1 alpha subcomplex subunit 3 OS=Mus musculus GN=Ndufa3 PE=1 SV=1 [MASS=9331]	2	13	1	7.67E+05	[illegible]
O08528	HXK2_MOUSE	Hexokinase-2 OS=Mus musculus GN=Hk2 PE=1 SV=1 [MASS=102535]	13	13	1	3.52E+06	[illegible]
Q9D023	BR44_MOUSE	Brain protein 44 OS=Mus musculus GN=Brp44 PE=1 SV=1 [MASS=14286]	5	13	1	1.19E+06	[illegible]
Q9D6J5	NDUB8_MOUSE	NADH dehydrogenase [ubiquinone] 1 beta subcomplex subunit 8, mitochondrial OS=Mus musculus GN=Ndufb8 PE=1 SV=1 [MASS=21876]	6	13	1	4.40E+06	[illegible]
Q4VAE3	TMM65_MOUSE	Transmembrane protein 65 OS=Mus musculus GN=Tmem65 PE=2 SV=1 [MASS=24918]	2	9	1	9.18E+04	[illegible]
Q9CPQ8	ATP5L_MOUSE	ATP synthase subunit g, mitochondrial OS=Mus musculus GN=Atp5l PE=1 SV=1 [MASS=11424]	4	13	1	3.59E+06	[illegible]
Q9CQR4	ACO13_MOUSE	Acyl-coenzyme A thioesterase 13 OS=Mus musculus GN=Acot13 PE=1 SV=1 [MASS=15183]	3	13	1	1.78E+06	[illegible]
Q91WS0	CISD1_MOUSE	CDGSH iron sulfur domain-containing protein 1 OS=Mus musculus GN=Cisd1 PE=1 SV=1 [MASS=12097]	3	13	1	1.22E+06	[illegible]
P56135	ATPK_MOUSE	ATP synthase subunit f, mitochondrial OS=Mus musculus GN=Atp5j2 PE=1 SV=3 [MASS=10344]	2	11	1	9.15E+06	[illegible]
Q9D2G2	ODO2_MOUSE	Dihydrolipoyllysine-residue succinyltransferase component of 2-oxoglutarate dehydrogenase complex, mitochondrial OS=Mus musculus GN=Dlst PE=1 SV=1 [MASS=48994]	7	13	1	6.74E+06	[illegible]
P48787	TNNI3_MOUSE	Troponin I, cardiac muscle OS=Mus musculus GN=Tnni3 PE=1 SV=2 [MASS=24259]	6	13	1	1.99E+07	[illegible]
Q9Z239	PLM_MOUSE	Phospholemman OS=Mus musculus GN=Fxyd1 PE=1 SV=1 [MASS=10323]	1	12	0.99	3.24E+05	[illegible]
Q9CQC7	NDUB4_MOUSE	NADH dehydrogenase [ubiquinone] 1 beta subcomplex subunit 4 OS=Mus musculus GN=Ndufb4 PE=1 SV=3 [MASS=15081]	4	13	1	4.12E+06	[illegible]
Q64310	SURF4_MOUSE	Surfeit locus protein 4 OS=Mus musculus GN=Surf4 PE=2 SV=1 [MASS=30381]	2	9	1	4.90E+04	[illegible]
P35980	RL18_MOUSE	60S ribosomal protein L18 OS=Mus musculus GN=Rpl18 PE=2 SV=3 [MASS=21644]	3	8	1	1.12E+05	[illegible]
P63101	1433Z_MOUSE	14-3-3 protein zeta/delta OS=Mus musculus GN=Ywhaz PE=1 SV=1 [MASS=27771]	3	13	1	1.32E+06	[illegible]
Q99LC3	NDUAA_MOUSE	NADH dehydrogenase [ubiquinone] 1 alpha subcomplex subunit 10, mitochondrial OS=Mus musculus GN=Ndufa10 PE=1 SV=1 [MASS=40603]	16	13	1	1.38E+07	[illegible]
P56379	68MP_MOUSE	6.8 kDa mitochondrial proteolipid OS=Mus musculus GN=Mp68 PE=2 SV=1 [MASS=6698]	1	11	0.99	1.46E+06	[illegible]
P35293	RAB18_MOUSE	Ras-related protein Rab-18 OS=Mus musculus GN=Rab18 PE=2 SV=2 [MASS=23035]	1	1	1	3.67E+03	[illegible]
Q9CXZ1	NDUS4_MOUSE	NADH dehydrogenase [ubiquinone] iron-sulfur protein 4, mitochondrial OS=Mus musculus GN=Ndufs4 PE=1 SV=3 [MASS=19785]	8	13	1	2.60E+06	[illegible]
P99027	RLA2_MOUSE	60S acidic ribosomal protein P2 OS=Mus musculus GN=Rplp2 PE=1 SV=3 [MASS=11651]	3	13	1	9.16E+05	[illegible]
P21550	ENOB_MOUSE	Beta-enolase OS=Mus musculus GN=Eno3 PE=1 SV=3 [MASS=47025]	9	13	1	2.42E+07	[illegible]
O09111	NDUBB_MOUSE	NADH dehydrogenase [ubiquinone] 1 beta subcomplex subunit 11, mitochondrial OS=Mus musculus GN=Ndufb11 PE=1 SV=2 [MASS=17444]	5	13	1	2.66E+06	[illegible]
Q9CZB0	C560_MOUSE	Succinate dehydrogenase cytochrome b560 subunit, mitochondrial OS=Mus musculus GN=Sdhc PE=2 SV=1 [MASS=18382]	4	13	1	6.01E+05	[illegible]
P53986	MOT1_MOUSE	Monocarboxylate transporter 1 OS=Mus musculus GN=Slc16a1 PE=1 SV=1 [MASS=53267]	5	13	1	1.19E+06	[illegible]
P52480	KPYM_MOUSE	Pyruvate kinase isozymes M1/M2 OS=Mus musculus GN=Pkm2 PE=1 SV=4 [MASS=57845]	17	13	1	1.32E+07	[illegible]
Q9DC70	NDUS7_MOUSE	NADH dehydrogenase [ubiquinone] iron-sulfur protein 7, mitochondrial OS=Mus musculus GN=Ndufs7 PE=1 SV=1 [MASS=24683]	3	13	1	1.95E+06	[illegible]
P02089	HBB2_MOUSE	Hemoglobin subunit beta-2 OS=Mus musculus GN=Hbb-b2 PE=1 SV=2 [MASS=15878]	1	1	1	8.89E+03	[illegible]
O55126	NIPS2_MOUSE	Protein NipSnap homolog 2 OS=Mus musculus GN=Gbas PE=2 SV=1 [MASS=32932]	4	13	1	1.93E+06	[illegible]
P32020	NLTP_MOUSE	Non-specific lipid-transfer protein OS=Mus musculus GN=Scp2 PE=1 SV=3 [MASS=59125]	2	13	1	2.39E+05	[illegible]
Q9CQ54	NDUC2_MOUSE	NADH dehydrogenase [ubiquinone] 1 subunit C2 OS=Mus musculus GN=Ndufc2 PE=1 SV=1 [MASS=14164]	5	13	1	1.23E+06	[illegible]
Q9DC69	NDUA9_MOUSE	NADH dehydrogenase [ubiquinone] 1 alpha subcomplex subunit 9, mitochondrial OS=Mus musculus GN=Ndufa9 PE=1 SV=1 [MASS=42509]	18	13	1	1.55E+07	[illegible]
P62204	CALM_MOUSE	Calmodulin OS=Mus musculus GN=Calm1 PE=1 SV=2 [MASS=16838]	3	6	1	1.48E+05	[illegible]
P99028	QCR6_MOUSE	Cytochrome b-c1 complex subunit 6, mitochondrial OS=Mus musculus GN=Uqcrh PE=1 SV=2 [MASS=10435]	2	11	1	9.02E+04	[illegible]
Q8R0Y8	S2542_MOUSE	Solute carrier family 25 member 42 OS=Mus musculus GN=Slc25a42 PE=2 SV=1 [MASS=35241]	1	8	0.99	1.12E+05	[illegible]
P12970	RL7A_MOUSE	60S ribosomal protein L7a OS=Mus musculus GN=Rpl7a PE=2 SV=2 [MASS=29976]	1	1	0.75	1.94E+04	[illegible]
P14115	RL27A_MOUSE	60S ribosomal protein L27a OS=Mus musculus GN=Rpl27a PE=1 SV=4 [MASS=16589]	2	13	1	6.29E+04	[illegible]
Q9EQ20	MMSA_MOUSE	Methylmalonate-semialdehyde dehydrogenase [acylating], mitochondrial OS=Mus musculus GN=Aldh6a1 PE=1 SV=1 [MASS=57915]	14	13	1	4.24E+06	[illegible]
P62259	1433E_MOUSE	14-3-3 protein epsilon OS=Mus musculus GN=Ywhae PE=1 SV=1 [MASS=29174]	8	13	1	2.79E+06	[illegible]
Q60931	VDAC3_MOUSE	Voltage-dependent anion-selective channel protein 3 OS=Mus musculus GN=Vdac3 PE=1 SV=1 [MASS=30753]	5	13	1	3.25E+06	[illegible]
Q9D6R2	IDH3A_MOUSE	Isocitrate dehydrogenase [NAD] subunit alpha, mitochondrial OS=Mus musculus GN=Idh3a PE=1 SV=1 [MASS=39638]	13	13	1	2.20E+07	[illegible]
P03911	NU4M_MOUSE	NADH-ubiquinone oxidoreductase chain 4 OS=Mus musculus GN=Mtnd4 PE=1 SV=1 [MASS=51882]	4	13	1	4.54E+06	[illegible]
Q06890	CLUS_MOUSE	Clusterin OS=Mus musculus GN=Clu PE=1 SV=1 [MASS=51655]	4	12	1	1.07E+05	[illegible]

P62806	H4_MOUSE	Histone H4 OS=Mus musculus GN=Hist1h4a PE=1 SV=2 [MASS=11367]	5	13	1	8.46E+06	7.72E+06	8.51E+06	6.97E+06	7.38E+06	[illegible]
Q9D172	ES1_MOUSE	ES1 protein homolog, mitochondrial OS=Mus musculus GN=D10Jhu81e PE=1 SV=1 [MASS=28090]	4	13	1	3.40E+06	4.10E+06	3.76E+06	3.95E+06	4.00E+06	[illegible]
Q9DCX2	ATP5H_MOUSE	ATP synthase subunit d, mitochondrial OS=Mus musculus GN=Atp5h PE=1 SV=3 [MASS=18749]	9	13	1	3.22E+07	3.01E+07	3.24E+07	3.43E+07	3.25E+07	[illegible]
P20152	VIME_MOUSE	Vimentin OS=Mus musculus GN=Vim PE=1 SV=3 [MASS=53687]	15	13	1	3.39E+06	4.09E+06	3.87E+06	3.36E+06	3.20E+06	[illegible]
Q9DCW4	ETFB_MOUSE	Electron transfer flavoprotein subunit beta OS=Mus musculus GN=Etfb PE=1 SV=3 [MASS=27623]	12	13	1	1.56E+07	1.70E+07	1.95E+07	1.79E+07	2.07E+07	[illegible]
Q64523	H2A1F_MOUSE	Histone H2A type 2-C OS=Mus musculus GN=Hist2h2ac PE=1 SV=3 [MASS=13988]	1	12	1	6.06E+06	4.77E+06	5.13E+06	5.04E+06	4.19E+06	[illegible]
Q9Z1P6	NDUA7_MOUSE	NADH dehydrogenase [ubiquinone] 1 alpha subcomplex subunit 7 OS=Mus musculus GN=Ndufa7 PE=1 SV=3 [MASS=12576]	3	13	1	1.37E+06	1.11E+06	8.50E+05	9.88E+05	1.48E+06	[illegible]
P14152	MDHC_MOUSE	Malate dehydrogenase, cytoplasmic OS=Mus musculus GN=Mdh1 PE=1 SV=3 [MASS=36511]	11	13	1	2.37E+07	2.47E+07	2.64E+07	2.37E+07	2.40E+07	[illegible]
P20108	PRDX3_MOUSE	Thioredoxin-dependent peroxide reductase, mitochondrial OS=Mus musculus GN=Prdx3 PE=1 SV=1 [MASS=28127]	6	13	1	2.18E+06	2.84E+06	5.18E+06	4.31E+06	4.90E+06	[illegible]
P03899	NU3M_MOUSE	NADH-ubiquinone oxidoreductase chain 3 OS=Mus musculus GN=Mtnd3 PE=3 SV=3 [MASS=13219]	1	11	0.99	3.05E+04	1.83E+04	2.26E+05	7.14E+04	4.08E+05	[illegible]
P02088	HBB1_MOUSE	Hemoglobin subunit beta-1 OS=Mus musculus GN=Hbb-b1 PE=1 SV=2 [MASS=15840]	4	13	1	1.18E+07	1.66E+07	1.71E+07	2.39E+07	1.38E+07	[illegible]
Q9CR21	ACPM_MOUSE	Acyl carrier protein, mitochondrial OS=Mus musculus GN=Ndufab1 PE=1 SV=1 [MASS=17370]	3	13	1	1.06E+06	1.10E+06	2.95E+06	1.84E+06	1.19E+06	[illegible]
P70670	NACAM_MOUSE	Nascent polypeptide-associated complex subunit alpha, muscle-specific form OS=Mus musculus GN=Naca PE=1 SV=1 [MASS=220600]	22	13	1	1.68E+06	1.50E+06	1.17E+06	1.32E+06	1.58E+06	[illegible]
Q9ESD7	DYSF_MOUSE	Dysferlin OS=Mus musculus GN=Dysf PE=1 SV=3 [MASS=237909]	3	3	1	1.04E+05	8.51E+04	9.32E+04	1.07E+05	1.00E+05	[illegible]
P23242	CXA1_MOUSE	Gap junction alpha-1 protein OS=Mus musculus GN=Gja1 PE=1 SV=2 [MASS=43004]	4	10	1	7.86E+05	6.49E+05	3.96E+05	4.17E+05	4.54E+05	[illegible]
Q9D3D9	ATPD_MOUSE	ATP synthase subunit delta, mitochondrial OS=Mus musculus GN=Atp5d PE=1 SV=1 [MASS=17600]	2	13	1	1.07E+07	9.98E+06	4.44E+06	6.54E+06	6.66E+06	[illegible]
P47955	RLA1_MOUSE	60S acidic ribosomal protein P1 OS=Mus musculus GN=Rplp1 PE=1 SV=1 [MASS=11475]	1	13	1	4.90E+05	3.72E+05	4.00E+05	4.16E+05	3.38E+05	[illegible]
Q9CQZ6	NDUB3_MOUSE	NADH dehydrogenase [ubiquinone] 1 beta subcomplex subunit 3 OS=Mus musculus GN=Ndufb3 PE=1 SV=1 [MASS=11692]	3	13	1	2.39E+06	1.85E+06	1.73E+06	1.80E+06	1.94E+06	[illegible]
A2AMM0	MURC_MOUSE	Muscle-related coiled-coil protein OS=Mus musculus GN=Murc PE=2 SV=1 [MASS=41008]	4	13	1	3.42E+05	3.66E+05	4.77E+05	3.63E+05	4.65E+05	[illegible]
Q9D0F9	PGM1_MOUSE	Phosphoglucomutase-1 OS=Mus musculus GN=Pgm1 PE=1 SV=3 [MASS=61517]	16	13	1	5.73E+06	4.53E+06	4.15E+06	4.34E+06	4.14E+06	[illegible]
Q8K3J1	NDUS8_MOUSE	NADH dehydrogenase [ubiquinone] iron-sulfur protein 8, mitochondrial OS=Mus musculus GN=Ndufs8 PE=1 SV=1 [MASS=24038]	4	13	1	3.58E+06	3.76E+06	4.53E+06	4.46E+06	3.57E+06	[illegible]
Q8BSY0	ASPH_MOUSE	Aspartyl/asparaginyl beta-hydroxylase OS=Mus musculus GN=Asph PE=2 SV=1 [MASS=83042]	1	1	0.98	1.11E+05	1.09E+05	8.20E+04	1.07E+05	9.82E+04	[illegible]
P51660	DHB4_MOUSE	Peroxisomal multifunctional enzyme type 2 OS=Mus musculus GN=Hsd17b4 PE=1 SV=3 [MASS=79481]	3	10	1	3.01E+05	2.04E+05	1.21E+05	1.68E+05	1.56E+05	[illegible]
Q6PB66	LPPRC_MOUSE	Leucine-rich PPR motif-containing protein, mitochondrial OS=Mus musculus GN=Lrpprc PE=1 SV=2 [MASS=156614]	23	13	1	3.00E+06	2.56E+06	2.88E+06	2.71E+06	2.71E+06	[illegible]
P68040	GBLP_MOUSE	Guanine nucleotide-binding protein subunit beta-2-like 1 OS=Mus musculus GN=Gnb2l1 PE=1 SV=3 [MASS=35076]	2	4	1	1.90E+04	2.58E+04	5.13E+04	2.58E+04	6.13E+04	[illegible]
Q9WUM5	SUCA_MOUSE	Succinyl-CoA ligase [GDP-forming] subunit alpha, mitochondrial OS=Mus musculus GN=Suclg1 PE=1 SV=4 [MASS=36155]	8	13	1	6.26E+06	5.84E+06	4.90E+06	5.68E+06	5.88E+06	[illegible]
Q8C129	LCAP_MOUSE	Leucyl-cystinyl aminopeptidase OS=Mus musculus GN=Lnpep PE=1 SV=1 [MASS=117303]	1	6	1	5.97E+04	4.40E+04	5.26E+04	5.34E+04	5.11E+04	[illegible]
P09103	PDIA1_MOUSE	Protein disulfide-isomerase OS=Mus musculus GN=P4hb PE=1 SV=1 [MASS=57143]	8	13	1	8.04E+05	7.46E+05	5.58E+05	6.77E+05	6.17E+05	[illegible]
P08752	GNAI2_MOUSE	Guanine nucleotide-binding protein G(i), alpha-2 subunit OS=Mus musculus GN=Gnai2 PE=1 SV=4 [MASS=40471]	5	12	1	2.38E+05	2.24E+05	2.61E+05	2.54E+05	3.52E+05	[illegible]
Q9DCM2	GSTK1_MOUSE	Glutathione S-transferase kappa 1 OS=Mus musculus GN=Gstk1 PE=1 SV=3 [MASS=25704]	8	13	1	1.24E+06	1.28E+06	1.31E+06	1.41E+06	1.36E+06	[illegible]
Q8BMS4	COQ3_MOUSE	Hexaprenyldihydroxybenzoate methyltransferase, mitochondrial OS=Mus musculus GN=Coq3 PE=2 SV=1 [MASS=40956]	2	12	1	4.45E+05	3.49E+05	2.16E+05	2.81E+05	2.54E+05	[illegible]
P08228	SODC_MOUSE	Superoxide dismutase [Cu-Zn] OS=Mus musculus GN=Sod1 PE=1 SV=2 [MASS=15943]	4	13	1	3.84E+06	4.31E+06	4.27E+06	3.83E+06	3.88E+06	[illegible]
Q8CHT0	AL4A1_MOUSE	Delta-1-pyrroline-5-carboxylate dehydrogenase, mitochondrial OS=Mus musculus GN=Aldh4a1 PE=1 SV=2 [MASS=61810]	7	13	1	1.72E+06	1.58E+06	1.28E+06	1.40E+06	1.66E+06	[illegible]
Q9DCV4	RMD1_MOUSE	Regulator of microtubule dynamics protein 1 OS=Mus musculus GN=Fam82b PE=2 SV=2 [MASS=35000]	6	13	1	4.77E+05	5.18E+05	4.87E+05	5.29E+05	4.56E+05	[illegible]
Q8K370	ACD10_MOUSE	Acyl-CoA dehydrogenase family member 10 OS=Mus musculus GN=Acad10 PE=2 SV=1 [MASS=118978]	6	13	1	7.41E+05	8.08E+05	8.08E+05	9.77E+05	8.05E+05	[illegible]
P47802	MTX1_MOUSE	Metaxin-1 OS=Mus musculus GN=Mtx1 PE=1 SV=1 [MASS=35624]	2	6	1	9.12E+04	6.54E+04	4.14E+04	3.91E+04	5.54E+04	[illegible]
Q925I1	ATAD3_MOUSE	ATPase family AAA domain-containing protein 3 OS=Mus musculus GN=Atad3 PE=1 SV=1 [MASS=66742]	2	6	1	1.21E+05	8.35E+04	7.33E+04	7.84E+04	8.62E+04	[illegible]
Q9DCN2	NB5R3_MOUSE	NADH-cytochrome b5 reductase 3 OS=Mus musculus GN=Cyb5r3 PE=1 SV=3 [MASS=34127]	2	7	1	9.68E+04	8.12E+04	6.02E+04	7.57E+04	8.53E+04	[illegible]
P62264	RS14_MOUSE	40S ribosomal protein S14 OS=Mus musculus GN=Rps14 PE=2 SV=3 [MASS=16273]	3	13	1	1.98E+05	1.65E+05	9.25E+04	1.51E+05	1.23E+05	[illegible]
Q62167	DDX3X_MOUSE	ATP-dependent RNA helicase DDX3X OS=Mus musculus GN=Ddx3x PE=1 SV=3 [MASS=73101]	1	5	1	3.90E+04	2.94E+04	2.95E+04	2.85E+04	2.91E+04	[illegible]
P10649	GSTM1_MOUSE	Glutathione S-transferase Mu 1 OS=Mus musculus GN=Gstm1 PE=1 SV=2 [MASS=25970]	5	13	1	1.11E+06	1.24E+06	1.32E+06	1.18E+06	1.45E+06	[illegible]
Q9D8E6	RL4_MOUSE	60S ribosomal protein L4 OS=Mus musculus GN=Rpl4 PE=1 SV=3 [MASS=47153]	5	11	1	2.16E+05	3.07E+05	2.66E+05	2.58E+05	2.66E+05	[illegible]
Q9CPR4	RL17_MOUSE	60S ribosomal protein L17 OS=Mus musculus GN=Rpl17 PE=2 SV=3 [MASS=21423]	1	10	0.99	0.00E+00	1.97E+04	5.04E+04	2.40E+04	9.08E+03	[illegible]
P25444	RS2_MOUSE	40S ribosomal protein S2 OS=Mus musculus GN=Rps2 PE=1 SV=3 [MASS=31231]	3	11	1	2.47E+05	2.18E+05	2.49E+05	2.57E+05	1.71E+05	[illegible]
P47963	RL13_MOUSE	60S ribosomal protein L13 OS=Mus musculus GN=Rpl13 PE=2 SV=3 [MASS=24305]	2	12	1	1.68E+05	1.53E+05	9.15E+04	1.10E+05	1.36E+05	[illegible]
P63260	ACTG_MOUSE	Actin, cytoplasmic 2 OS=Mus musculus GN=Actg1 PE=1 SV=1 [MASS=41793]	5	13	1	4.98E+06	4.80E+06	3.59E+06	3.56E+06	4.33E+06	[illegible]
P28654	PGS2_MOUSE	Decorin OS=Mus musculus GN=Dcn PE=2 SV=1 [MASS=39809]	1	3	0.98	2.58E+04	4.03E+04	7.42E+04	4.48E+04	4.06E+04	[illegible]
P07356	ANXA2_MOUSE	Annexin A2 OS=Mus musculus GN=Anxa2 PE=1 SV=2 [MASS=38676]	7	13	1	9.06E+05	8.22E+05	7.55E+05	8.58E+05	7.02E+05	[illegible]
Q9D8Y1	T126A_MOUSE	Transmembrane protein 126A OS=Mus musculus GN=Tmem126a PE=2 SV=1 [MASS=21539]	1	1	0.99	2.76E+04	3.18E+04	3.93E+04	2.59E+04	4.01E+04	[illegible]
P63325	RS10_MOUSE	40S ribosomal protein S10 OS=Mus musculus GN=Rps10 PE=1 SV=1 [MASS=18916]	1	13	0.99	8.38E+04	6.11E+04	1.12E+05	1.20E+05	5.84E+04	[illegible]
Q64521	GPDM_MOUSE	Glycerol-3-phosphate dehydrogenase, mitochondrial OS=Mus musculus GN=Gpd2 PE=1 SV=2 [MASS=80953]	1	4	0.99	1.39E+04	1.38E+04	2.25E+04	2.21E+04	8.13E+03	[illegible]
P62270	RS18_MOUSE	40S ribosomal protein S18 OS=Mus musculus GN=Rps18 PE=2 SV=3 [MASS=17719]	4	5	1	1.23E+05	1.25E+05	6.95E+04	8.27E+04	9.60E+04	[illegible]
P18242	CATD_MOUSE	Cathepsin D OS=Mus musculus GN=Ctsd PE=1 SV=1 [MASS=44954]	3	13	1	4.41E+05	7.97E+05	7.76E+05	7.27E+05	5.45E+05	[illegible]
P62141	PP1B_MOUSE	Serine/threonine-protein phosphatase PP1-beta catalytic subunit OS=Mus musculus GN=Ppp1cb PE=1 SV=3 [MASS=37187]	2	12	1	1.91E+05	1.89E+05	2.05E+05	1.59E+05	2.04E+05	[illegible]
P84099	RL19_MOUSE	60S ribosomal protein L19 OS=Mus musculus GN=Rpl19 PE=1 SV=1 [MASS=23466]	1	2	0.99	4.09E+04	3.66E+04	3.19E+04	2.34E+04	3.01E+04	[illegible]
P08113	ENPL_MOUSE	Endoplasmin OS=Mus musculus GN=Hsp90b1 PE=1 SV=2 [MASS=92475]	6	13	1	2.92E+05	2.54E+05	3.22E+05	3.18E+05	2.63E+05	[illegible]
P51150	RAB7A_MOUSE	Ras-related protein Rab-7a OS=Mus musculus GN=Rab7a PE=1 SV=2 [MASS=23490]	3	3	1	7.62E+04	7.29E+04	7.86E+04	6.53E+04	6.12E+04	[illegible]
Q8BW75	AOFB_MOUSE	Amine oxidase [flavin-containing] B OS=Mus musculus GN=Maob PE=1 SV=3 [MASS=58543]	7	12	1	7.20E+05	6.90E+05	6.32E+05	6.89E+05	7.58E+05	[illegible]
P62908	RS3_MOUSE	40S ribosomal protein S3 OS=Mus musculus GN=Rps3 PE=1 SV=1 [MASS=26674]	7	13	1	6.14E+05	6.04E+05	5.82E+05	6.02E+05	5.72E+05	[illegible]
Q9CQN6	TM14C_MOUSE	Transmembrane protein 14C OS=Mus musculus GN=Tmem14c PE=1 SV=1 [MASS=11642]	2	11	1	2.17E+05	1.90E+05	1.57E+05	1.86E+05	1.79E+05	[illegible]
P62754	RS6_MOUSE	40S ribosomal protein S6 OS=Mus musculus GN=Rps6 PE=1 SV=1 [MASS=28680]	1	1	0.99	3.88E+04	6.31E+04	6.26E+04	7.44E+04	6.46E+04	[illegible]
Q9CPQ3	TOM22_MOUSE	Mitochondrial import receptor subunit TOM22 homolog OS=Mus musculus GN=Tomm22 PE=2 SV=3 [MASS=15537]	1	13	0.99	1.76E+05	1.38E+05	6.61E+04	8.96E+04	1.33E+05	[illegible]
P63276	RS17_MOUSE	40S ribosomal protein S17 OS=Mus musculus GN=Rps17 PE=1 SV=2 [MASS=15524]	1	13	0.99	4.09E+05	2.87E+05	2.13E+05	2.78E+05	3.16E+05	[illegible]
P01837	IGKC_MOUSE	Ig kappa chain C region OS=Mus musculus PE=1 SV=1 [MASS=11778]	1	8	1	2.81E+04	2.28E+04	1.72E+04	5.07E+04	3.98E+04	[illegible]
Q99JI6	RAP1B_MOUSE	Ras-related protein Rap-1b OS=Mus musculus GN=Rap1b PE=2 SV=2 [MASS=20825]	1	1	1	2.08E+04	2.12E+04	2.33E+04	1.88E+04	2.62E+04	[illegible]
P41105	RL28_MOUSE	60S ribosomal protein L28 OS=Mus musculus GN=Rpl28 PE=1 SV=2 [MASS=15733]	1	7	0.99	4.43E+04	3.69E+04	7.99E+03	2.02E+04	2.69E+04	[illegible]
P05201	AATC_MOUSE	Aspartate aminotransferase, cytoplasmic OS=Mus musculus GN=Got1 PE=1 SV=2 [MASS=46231]	18	13	1	2.66E+07	2.71E+07	2.27E+07	2.42E+07	2.24E+07	[illegible]
Q9DCT8	CRIP2_MOUSE	Cysteine-rich protein 2 OS=Mus musculus GN=Crip2 PE=1 SV=1 [MASS=22727]	2	13	1	3.30E+06	3.36E+06	2.51E+06	2.77E+06	2.88E+06	[illegible]
Q9QUI0	RHOA_MOUSE	Transforming protein RhoA OS=Mus musculus GN=Rhoa PE=1 SV=1 [MASS=21782]	1	12	1	2.17E+05	1.64E+05	1.24E+05	1.40E+05	1.74E+05	[illegible]
P62075	TIM13_MOUSE	Mitochondrial import inner membrane translocase subunit Tim13 OS=Mus musculus GN=Timm13 PE=1 SV=1 [MASS=10458]	2	13	1	7.46E+04	9.73E+04	9.69E+04	8.64E+04	1.02E+05	[illegible]
Q9ET78	JPH2_MOUSE	Junctophilin-2 OS=Mus musculus GN=Jph2 PE=1 SV=2 [MASS=74692]	4	12	1	3.97E+05	4.47E+05	2.84E+05	3.66E+05	4.02E+05	[illegible]
P48036	ANXA5_MOUSE	Annexin A5 OS=Mus musculus GN=Anxa5 PE=1 SV=1 [MASS=35752]	5	12	1	8.11E+05	6.45E+05	6.69E+05	6.96E+05	4.93E+05	[illegible]
Q9D2R6	CCD56_MOUSE	Coiled-coil domain-containing protein 56 OS=Mus musculus GN=Ccdc56 PE=2 SV=1 [MASS=11987]	1	11	1	5.82E+04	4.10E+04	3.62E+04	3.15E+04	4.72E+04	[illegible]
P24369	PPIB_MOUSE	Peptidyl-prolyl cis-trans isomerase B OS=Mus musculus GN=Ppib PE=2 SV=2 [MASS=23713]	2	9	1	4.21E+04	7.71E+04	7.68E+04	5.30E+04	4.89E+04	[illegible]
Q8QZS1	HIBCH_MOUSE	3-hydroxyisobutyryl-CoA hydrolase, mitochondrial OS=Mus musculus GN=Hibch PE=1 SV=1 [MASS=43037]	12	13	1	2.87E+06	2.81E+06	2.78E+06	2.80E+06	2.98E+06	[illegible]
P35564	CALX_MOUSE	Calnexin OS=Mus musculus GN=Canx PE=1 SV=1 [MASS=67277]	3	13	1	3.03E+05	3.03E+05	3.02E+05	3.02E+05	2.80E+05	[illegible]
P62830	RL23_MOUSE	60S ribosomal protein L23 OS=Mus musculus GN=Rpl23 PE=2 SV=1 [MASS=14865]	1	1	0.99	1.91E+04	1.29E+04	2.12E+04	1.38E+04	2.60E+04	[illegible]
Q61171	PRDX2_MOUSE	Peroxiredoxin-2 OS=Mus musculus GN=Prdx2 PE=1 SV=3 [MASS=21778]	6	13	1	2.18E+06	2.15E+06	1.91E+06	1.81E+06	2.01E+06	[illegible]
P16546	SPTA2_MOUSE	Spectrin alpha chain, brain OS=Mus musculus GN=Sptan1 PE=1 SV=4 [MASS=284595]	41	13	1	6.56E+06	5.74E+06	4.55E+06	4.97E+06	4.91E+06	[illegible]
P62880	GBB2_MOUSE	Guanine nucleotide-binding protein G(I)/G(S)/G(T) subunit beta-2 OS=Mus musculus GN=Gnb2 PE=1 SV=3 [MASS=37331]	1	1	1	1.20E+04	1.31E+04	3.13E+04	1.20E+04	4.24E+04	[illegible]
P47857	K6PF_MOUSE	6-phosphofructokinase, muscle type OS=Mus musculus GN=Pfkm PE=1 SV=3 [MASS=85268]	15	13	1	4.03E+06	3.86E+06	3.86E+06	4.11E+06	4.08E+06	[illegible]
P63323	RS12_MOUSE	40S ribosomal protein S12 OS=Mus musculus GN=Rps12 PE=1 SV=2 [MASS=14525]	2	5	0.99	3.28E+04	5.26E+04	4.68E+04	5.00E+04	4.88E+04	[illegible]
Q9WUB3	PYGM_MOUSE	Glycogen phosphorylase, muscle form OS=Mus musculus GN=Pygm PE=1 SV=3 [MASS=97286]	24	13	1	9.27E+06	8.63E+06	8.81E+06	9.54E+06	8.89E+06	[illegible]
Q9D1G1	RAB1B_MOUSE	Ras-related protein Rab-1B OS=Mus musculus GN=Rab1b PE=1 SV=1 [MASS=22187]	3	13	1	3.29E+05	2.38E+05	2.46E+05	2.76E+05	2.29E+05	[illegible]
Q91ZA3	PCCA_MOUSE	Propionyl-CoA carboxylase alpha chain, mitochondrial OS=Mus musculus GN=Pcca PE=2 SV=2 [MASS=79921]	11	13	1	1.11E+06	1.04E+06	8.92E+05	1.03E+06	1.13E+06	[illegible]
O35683	NDUA1_MOUSE	NADH dehydrogenase [ubiquinone] 1 alpha subcomplex subunit 1 OS=Mus musculus GN=Ndufa1 PE=2 SV=1 [MASS=8138]	1	13	1	8.50E+05	1.44E+06	9.81E+05	1.38E+06	1.10E+06	[illegible]
P31001	DESM_MOUSE	Desmin OS=Mus musculus GN=Des PE=1 SV=3 [MASS=53497]	20	13	1	3.30E+07	3.05E+07	2.40E+07	2.70E+07	2.67E+07	[illegible]
P63028	TCTP_MOUSE	Translationally-controlled tumor protein OS=Mus musculus GN=Tpt1 PE=1 SV=1 [MASS=19462]	2	13	1	2.13E+05	4.26E+05	4.87E+05	3.54E+05	2.69E+05	[illegible]
Q9QY76	VAPB_MOUSE	Vesicle-associated membrane protein-associated protein B OS=Mus musculus GN=Vapb PE=2 SV=3 [MASS=26946]	2	13	1	4.02E+05	3.24E+05	4.08E+05	3.67E+05	3.81E+05	[illegible]
P35700	PRDX1_MOUSE	Peroxiredoxin-1 OS=Mus musculus GN=Prdx1 PE=1 SV=1 [MASS=22176]	4	13	1	1.85E+06	1.72E+06	1.81E+06	1.64E+06	1.62E+06	[illegible]
Q9DB15	RM12_MOUSE	39S ribosomal protein L12, mitochondrial OS=Mus musculus GN=Mrpl12 PE=1 SV=2 [MASS=21708]	3	13	1	7.69E+05	7.02E+05	4.96E+05	4.86E+05	5.77E+05	[illegible]
Q8BH64	EHD2_MOUSE	EH domain-containing protein 2 OS=Mus musculus GN=Ehd2 PE=1 SV=1 [MASS=61174]	10	13	1	1.04E+06	8.68E+05	1.27E+06	1.16E+06	9.43E+05	[illegible]
Q6PIE5	AT1A2_MOUSE	Sodium/potassium-transporting ATPase subunit alpha-2 OS=Mus musculus GN=Atp1a2 PE=1 SV=1 [MASS=112217]	1	8	1	2.17E+04	1.65E+04	2.07E+04	1.73E+04	1.09E+04	[illegible]
P51410	RL9_MOUSE	60S ribosomal protein L9 OS=Mus musculus GN=Rpl9 PE=2 SV=2 [MASS=21881]	3	12	1	2.82E+05	2.28E+05	1.92E+05	2.03E+05	2.54E+05	[illegible]
P59017	B2L13_MOUSE	Bcl-2-like protein 13 OS=Mus musculus GN=Bcl2l13 PE=1 SV=1 [MASS=46669]	3	13	1	5.36E+05	4.68E+05	3.18E+05	4.14E+05	4.28E+05	[illegible]
Q9Z2I8	SUCB2_MOUSE	Succinyl-CoA ligase [GDP-forming] subunit beta, mitochondrial OS=Mus musculus GN=Suclg2 PE=2 SV=3 [MASS=46840]	10	13	1	9.06E+05	9.65E+05	8.59E+05	8.85E+05	1.11E+06	[illegible]
Q8BMK4	CKAP4_MOUSE	Cytoskeleton-associated protein 4 OS=Mus musculus GN=Ckap4 PE=2 SV=2 [MASS=63692]	2	10	1	5.72E+04	3.64E+04	1.88E+04	3.67E+04	2.51E+04	[illegible]
Q99JB2	STML2_MOUSE	Stomatin-like protein 2 OS=Mus musculus GN=Stoml2 PE=1 SV=1 [MASS=38385]	1	5	1	9.85E+04	6.15E+04	3.55E+04	5.63E+04	7.30E+04	[illegible]
P29699	FETUA_MOUSE	Alpha-2-HS-glycoprotein OS=Mus musculus GN=Ahsg PE=1 SV=1 [MASS=37325]	4	13	1	1.08E+05	1.11E+05	5.30E+05	1.41E+05	4.27E+05	[illegible]
P09411	PGK1_MOUSE	Phosphoglycerate kinase 1 OS=Mus musculus GN=Pgk1 PE=1 SV=4 [MASS=44550]	12	13	1	5.64E+06	6.44E+06	7.37E+06	6.69E+06	6.65E+06	[illegible]
Q8VE95	CH082_MOUSE	UPF0598 protein C8orf82 homolog OS=Mus musculus PE=2 SV=1 [MASS=24330]	3	12	1	1.68E+05	1.43E+05	8.45E+04	1.20E+05	1.39E+05	[illegible]
Q8VD26	TM143_MOUSE	Transmembrane protein 143 OS=Mus musculus GN=Tmem143 PE=2 SV=1 [MASS=51595]	3	10	1	4.10E+04	8.42E+04	1.21E+05	1.13E+05	8.08E+04	[illegible]
P47962	RL5_MOUSE	60S ribosomal protein L5 OS=Mus musculus GN=Rpl5 PE=1 SV=3 [MASS=34400]	1	6	1	7.81E+04	6.78E+04	5.86E+04	5.31E+04	6.37E+04	[illegible]
P61014	PPLA_MOUSE	Cardiac phospholamban OS=Mus musculus GN=Pln PE=2 SV=1 [MASS=6094]	1	13	1	2.75E+06	1.55E+06	1.28E+06	9.49E+05	1.09E+06	[illegible]
Q8CIB5	FERM2_MOUSE	Fermitin family homolog 2 OS=Mus musculus GN=Fermt2 PE=1 SV=1 [MASS=77800]	6	12	1	2.68E+05	2.41E+05	2.48E+05	2.49E+05	2.39E+05	[illegible]
P97351	RS3A_MOUSE	40S ribosomal protein S3a OS=Mus musculus GN=Rps3a PE=1 SV=3 [MASS=29885]	3	10	1	7.85E+04	7.17E+04	1.50E+05	9.40E+04	1.10E+05	[illegible]
P62821	RAB1A_MOUSE	Ras-related protein Rab-1A OS=Mus musculus GN=Rab1A PE=1 SV=3 [MASS=22678]	1	13	1	1.60E+05	1.05E+05	9.49E+04	1.13E+05	9.31E+04	[illegible]
Q8CG76	ARK72_MOUSE	Aflatoxin B1 aldehyde reductase member 2 OS=Mus musculus GN=Akr7a2 PE=1 SV=2 [MASS=40598]	3	13	1	3.34E+05	3.15E+05	2.25E+05	2.76E+05	2.55E+05	[illegible]
Q9CZX8	RS19_MOUSE	40S ribosomal protein S19 OS=Mus musculus GN=Rps19 PE=1 SV=3 [MASS=16085]	1	10	0.99	8.18E+04	7.70E+04	6.03E+04	8.03E+04	7.07E+04	[illegible]
Q9CQ92	FIS1_MOUSE	Mitochondrial fission 1 protein OS=Mus musculus GN=Fis1 PE=1 SV=1 [MASS=17009]	3	13	1	3.07E+05	2.96E+05	1.84E+05	2.51E+05	2.45E+05	[illegible]
Q8K0E8	FIBB_MOUSE	Fibrinogen beta chain OS=Mus musculus GN=Fgb PE=2 SV=1 [MASS=54752]	6	13	1	4.97E+05	1.04E+06	5.80E+05	8.70E+05	5.95E+05	[illegible]
O35855	BCAT2_MOUSE	Branched-chain-amino-acid aminotransferase, mitochondrial OS=Mus musculus GN=Bcat2 PE=2 SV=2 [MASS=44127]	7	13	1	9.42E+05	1.57E+06	1.51E+06	1.56E+06	1.55E+06	[illegible]
Q9R0Y5	KAD1_MOUSE	Adenylate kinase isoenzyme 1 OS=Mus musculus GN=Ak1 PE=1 SV=1 [MASS=21539]	7	13	1	4.21E+06	5.75E+06	1.04E+07	6.82E+06	8.57E+06	[illegible]
P14142	GTR4_MOUSE	Solute carrier family 2, facilitated glucose transporter member 4 OS=Mus musculus GN=Slc2a4 PE=1 SV=2 [MASS=54783]	2	11	1	9.47E+04	1.10E+05	1.29E+05	1.32E+05	9.89E+04	[illegible]
Q61735	CD47_MOUSE	Leukocyte surface antigen CD47 OS=Mus musculus GN=Cd47 PE=1 SV=2 [MASS=33097]	1	2	0.99	6.29E+04	2.61E+04	4.27E+04	4.68E+04	4.26E+04	[illegible]
P63030	BR44L_MOUSE	Brain protein 44-like protein OS=Mus musculus GN=Brp44l PE=1 SV=1 [MASS=12455]	2	13	1	2.06E+06	2.66E+06	3.15E+06	2.36E+06	2.29E+06	[illegible]
O88322	NID2_MOUSE	Nidogen-2 OS=Mus musculus GN=Nid2 PE=1 SV=1 [MASS=154248]	2	9	1	1.18E+05	1.34E+05	1.08E+05	1.08E+05	8.60E+04	[illegible]
P14602	HSPB1_MOUSE	Heat shock protein beta-1 OS=Mus musculus GN=Hspb1 PE=1 SV=3 [MASS=23014]	5	13	1	1.93E+06	2.38E+06	2.10E+06	2.05E+06	1.80E+06	[illegible]
P62862	RS30_MOUSE	40S ribosomal protein S30 OS=Mus musculus GN=Fau PE=3 SV=1 [MASS=6648]	1	12	0.99	3.27E+04	3.81E+04	5.48E+04	4.86E+04	5.52E+04	[illegible]
Q9D880	TIM50_MOUSE	Mitochondrial import inner membrane translocase subunit TIM50 OS=Mus musculus GN=Timm50 PE=1 SV=1 [MASS=39776]	2	10	1	1.46E+05	1.03E+05	3.89E+04	5.84E+04	6.71E+04	[illegible]
Q9JKL4	NDUF3_MOUSE	NADH dehydrogenase [ubiquinone] 1 alpha subcomplex assembly factor 3 OS=Mus musculus GN=Ndufaf3 PE=2 SV=1 [MASS=20734]	1	2	1	1.33E+05	1.29E+05	9.69E+04	1.31E+05	1.09E+05	[illegible]
Q91VT4	CBR4_MOUSE	Carbonyl reductase family member 4 OS=Mus musculus GN=Cbr4 PE=2 SV=2 [MASS=25414]	2	13	1	1.64E+05	1.33E+05	1.38E+05	2.00E+05	1.48E+05	[illegible]
Q9CY58	PAIRB_MOUSE	Plasminogen activator inhibitor 1 RNA-binding protein OS=Mus musculus GN=Serbp1 PE=1 SV=2 [MASS=44714]	1	13	0.99	1.13E+05	7.44E+04	6.57E+04	8.47E+04	8.46E+04	[illegible]
Q6ZWY3	RS27L_MOUSE	40S ribosomal protein S27-like protein OS=Mus musculus GN=Rps27l PE=2 SV=3 [MASS=9477]	1	11	1	1.66E+05	1.17E+05	1.14E+05	1.23E+05	1.34E+05	[illegible]
P16332	MUTA_MOUSE	Methylmalonyl-CoA mutase, mitochondrial OS=Mus musculus GN=Mut PE=2 SV=1 [MASS=82965]	8	11	1	6.12E+05	6.03E+05	5.64E+05	6.04E+05	6.36E+05	[illegible]

P09541	MYL4_MOUSE	Myosin light chain 4 OS=Mus musculus GN=Myl4 PE=2 SV=3 [MASS=21159]	2	9	1	1.15E+05	6.03E+05	1.84E+05	1.34E+05	1.10E+05	1.70E+05	1.29E+05	1.27E+05	8.01E+04	7.34E+04	6.98E+04	1.62E+05	1.33E+05	0.017	1.77E-08	9.35E-06	0.001	0.008	0.001	0.001	0.002	0.014	0.008	0.004	0.006	8.53E-04	5	8, 9	8, 9	MYL4_MOUSE, SP:P09541	SP|P09541|MYL4_MOUSE
Q9CQN1	TRAP1_MOUSE	Heat shock protein 75 kDa, mitochondrial OS=Mus musculus GN=Trap1 PE=1 SV=1 [MASS=80208]	9	13	1	7.32E+05	6.70E+05	5.44E+05	6.73E+05	5.52E+05	6.00E+05	5.11E+05	6.23E+05	5.41E+05	4.58E+05	5.24E+05	4.54E+05	4.21E+05	4.31E-08	2.69E-09	1.06E-13	3.53E-12	3.96E-08	5.16E-09	9.67E-10	1.16E-10	5.03E-09	8.14E-10	6.06E-14	1.28E-05	1.20E-08	2, 3, 5, 6	1, 13, 3, 4, 5, 6, 7, 8	1, 13, 3, 4, 5, 6, 7, 8	TRAP1_MOUSE, SP:Q9CQN1	SP|Q9CQN1|TRAP1_MOUSE
P70296	PEBP1_MOUSE	Phosphatidylethanolamine-binding protein 1 OS=Mus musculus GN=Pebp1 PE=1 SV=3 [MASS=20830]	7	13	1	5.12E+06	5.28E+06	5.26E+06	5.42E+06	6.55E+06	6.47E+06	6.28E+06	5.31E+06	6.79E+06	6.46E+06	7.22E+06	7.59E+06	6.65E+06	1.96E-44	0	0	0	0	0	0	0	0	0	0	0	0	4, 4, 8, 5, 7	10, 12, 13, 5, 11, 8, 9	10, 11, 5, 12, 13, 8, 9	PEBP1_MOUSE, SP:P70296	SP|P70296|PEBP1_MOUSE
Q8C3X2	CC90B_MOUSE	Coiled-coil domain-containing protein 90B, mitochondrial OS=Mus musculus GN=Ccdc90b PE=2 SV=1 [MASS=29597]	1	11	0.99	1.97E+05	1.48E+05	1.29E+05	1.35E+05	1.65E+05	1.56E+05	1.09E+05	1.39E+05	1.04E+05	1.03E+05	1.09E+05	1.48E+05	1.08E+05	0.003	0.003	1.76E-04	0.001	0.002	0.002	0.003	0.001	0.006	0.002	3.45E-04	0.008	0.002	5	11	11	CC90B_MOUSE, SP:Q8C3X2	SP|Q8C3X2|CC90B_MOUSE
Q99L13	3HIDH_MOUSE	3-hydroxyisobutyrate dehydrogenase, mitochondrial OS=Mus musculus GN=Hibadh PE=2 SV=1 [MASS=35440]	8	13	1	1.51E+06	1.70E+06	1.31E+06	1.44E+06	1.43E+06	1.28E+06	1.30E+06	1.42E+06	1.14E+06	1.23E+06	1.26E+06	1.13E+06	1.00E+06	9.09E-15	9.10E-21	3.52E-30	8.03E-24	3.56E-18	3.40E-17	6.03E-22	7.93E-22	5.63E-17	3.77E-23	1.53E-30	1.74E-11	4.69E-18	2, 3, 3, 4, 4, 4, 5, 4, 6, 6	1, 13, 1, 8, 13, 2, 5, 7, 7, 13	1, 13, 1, 8, 13, 13, 7, 2, 5, 7	3HIDH_MOUSE, SP:Q99L13	SP|Q99L13|3HIDH_MOUSE
Q99M87	DNJA3_MOUSE	DnaJ homolog subfamily A member 3, mitochondrial OS=Mus musculus GN=Dnaja3 PE=1 SV=1 [MASS=52443]	5	13	1	3.39E+05	3.34E+05	3.08E+05	3.42E+05	3.29E+05	3.08E+05	3.07E+05	3.39E+05	2.78E+05	2.50E+05	2.41E+05	2.96E+05	2.92E+05	1.36E-04	1.95E-05	1.69E-08	5.14E-07	1.71E-05	2.07E-05	1.61E-06	1.48E-06	2.02E-05	4.20E-06	2.42E-07	3.34E-04	1.64E-06	2, 3, 5	13, 2, 3, 4	10, 13, 2, 3, 4	DNJA3_MOUSE, SP:Q99M87	SP|Q99M87|DNJA3_MOUSE
Q99MN9	PCCB_MOUSE	Propionyl-CoA carboxylase beta chain, mitochondrial OS=Mus musculus GN=Pccb PE=1 SV=1 [MASS=58394]	12	13	1	2.32E+06	2.26E+06	1.97E+06	2.49E+06	2.10E+06	2.27E+06	2.22E+06	2.10E+06	1.63E+06	1.81E+06	2.00E+06	1.70E+06	1.89E+06	9.71E-22	5.72E-27	3.64E-44	1.32E-39	6.56E-26	5.61E-29	4.54E-36	1.80E-31	1.45E-23	3.76E-33	0	1.93E-16	2.03E-32	2, 3, 3, 4, 3, 5, 4, 5, 6	1, 11, 1, 2, 13, 2, 4, 4, 8, 5, 6, 7, 8	1, 11, 1, 2, 13, 2, 4, 4, 8, 5, 6, 7, 8	PCCB_MOUSE, SP:Q99MN9	SP|Q99MN9|PCCB_MOUSE
Q9JI91	ACTN2_MOUSE	Alpha-actinin-2 OS=Mus musculus GN=Actn2 PE=1 SV=1 [MASS=103653]	29	13	1	4.58E+07	4.87E+07	4.33E+07	4.64E+07	4.57E+07	4.72E+07	4.74E+07	4.87E+07	5.26E+07	5.46E+07	5.42E+07	4.96E+07	5.77E+07	0	0	0	0	0	0	0	0	0	0	0	0	0	2, 2, 3, 2, 7, 3, 3, 4, 3, 5, 3, 6, 4, 4, 5, 4, 6, 5, 5, 6, 5, 8, 6, 8, 6, 9	1, 1, 12, 1, 9, 10, 11, 11, 11, 13, 12, 12, 13, 13, 2, 12, 2, 4, 4, 5, 6, 6, 13, 7, 8	1, 1, 12, 10, 11, 11, 12, 12, 13, 12, 2, 13, 13, 14, 13, 15, 13, 6, 19, 6, 7, 8, 9	ACTN2_MOUSE, SP:Q9JI91	SP|Q9JI91|ACTN2_MOUSE
Q8BIJ6	SYIM_MOUSE	Isoleucyl-tRNA synthetase, mitochondrial OS=Mus musculus GN=Iars2 PE=2 SV=1 [MASS=112803]	3	13	1	4.71E+05	4.39E+05	3.91E+05	4.41E+05	4.49E+05	4.57E+05	4.44E+05	3.56E+05	3.40E+05	3.24E+05	4.00E+05	4.23E+05	4.24E+05	8.82E-06	1.18E-06	2.38E-10	1.48E-08	6.47E-07	2.90E-07	1.09E-08	8.34E-07	2.78E-06	1.99E-07	4.15E-11	2.42E-05	1.08E-08	4, 4, 5, 6	3, 5, 6, 13	13, 6, 3, 5	SYIM_MOUSE, SP:Q8BIJ6	SP|Q8BIJ6|SYIM_MOUSE
P53702	CCHL_MOUSE	Cytochrome c-type heme lyase OS=Mus musculus GN=Hccs PE=2 SV=1 [MASS=31007]	2	10	1	1.65E+05	1.93E+05	1.51E+05	1.54E+05	1.39E+05	1.44E+05	1.05E+05	2.01E+05	1.30E+05	1.18E+05	1.18E+05	1.28E+05	1.14E+05	0.006	8.80E-04	5.28E-05	5.36E-04	0.004	0.003	0.003	1.65E-04	0.002	0.001	2.09E-04	0.013	0.002	3, 5	10, 2	10, 2	CCHL_MOUSE, SP:P53702	SP|P53702|CCHL_MOUSE
P45376	ALDR_MOUSE	Aldose reductase OS=Mus musculus GN=Akr1b1 PE=1 SV=3 [MASS=35732]	10	13	1	4.07E+06	4.14E+06	3.73E+06	4.19E+06	3.71E+06	3.76E+06	3.77E+06	3.58E+06	3.28E+06	3.70E+06	3.64E+06	3.53E+06	3.75E+06	3.54E-36	0	0	0	2.13E-43	0	0	0	1.12E-44	0	0	1.35E-31	0	2, 3, 3, 5, 4, 4, 6, 6	1, 12, 13, 2, 2, 5, 3, 12, 3, 13, 8	1, 12, 12, 3, 13, 13, 3, 2, 2, 5, 8	ALDR_MOUSE, SP:P45376	SP|P45376|ALDR_MOUSE
Q9D1L0	CHCH2_MOUSE	Coiled-coil-helix-coiled-coil-helix domain-containing protein 2, mitochondrial OS=Mus musculus GN=Chchd2 PE=1 SV=1 [MASS=15661]	1	6	0.99	1.63E+04	1.40E+04	2.97E+04	1.00E+04	5.62E+04	2.58E+04	3.25E+04	1.45E+04	5.03E+04	9.97E+04	6.04E+04	7.43E+04	1.66E+04	0.212	0.199	0.043	0.201	0.043	0.117	0.064	0.166	0.04	0.002	0.006	0.044	0.133	4	6	6	CHCH2_MOUSE, SP:Q9D1L0	SP|Q9D1L0|CHCH2_MOUSE
Q9WVK4	EHD1_MOUSE	EH domain-containing protein 1 OS=Mus musculus GN=Ehd1 PE=1 SV=1 [MASS=60602]	6	13	1	5.81E+05	5.00E+05	4.89E+05	4.66E+05	5.60E+05	4.60E+05	4.25E+05	4.28E+05	5.01E+05	5.31E+05	4.87E+05	5.49E+05	4.02E+05	9.66E-07	2.48E-07	2.07E-12	6.75E-09	3.44E-08	2.77E-07	2.44E-08	8.08E-08	1.87E-08	4.58E-11	4.70E-13	1.97E-06	2.78E-08	2, 3, 4, 5, 6	13, 2, 4, 6	13, 2, 4, 6, 7	EHD1_MOUSE, SP:Q9WVK4	SP|Q9WVK4|EHD1_MOUSE
P53994	RAB2A_MOUSE	Ras-related protein Rab-2A OS=Mus musculus GN=Rab2a PE=1 SV=1 [MASS=23547]	1	11	0.99	6.33E+04	6.29E+04	6.50E+04	6.26E+04	6.51E+04	4.70E+04	6.17E+04	6.11E+04	5.42E+04	4.03E+04	7.99E+04	5.86E+04	6.50E+04	0.058	0.037	0.006	0.018	0.033	0.054	0.018	0.023	0.035	0.037	0.002	0.065	0.014	2	11	11	RAB2A_MOUSE, SP:P53994	SP|P53994|RAB2A_MOUSE
Q9EQP2	EHD4_MOUSE	EH domain-containing protein 4 OS=Mus musculus GN=Ehd4 PE=1 SV=1 [MASS=61480]	11	13	1	1.53E+06	1.38E+06	9.66E+05	1.09E+06	1.20E+06	9.89E+05	1.05E+06	1.59E+06	1.53E+06	1.26E+06	1.20E+06	1.34E+06	1.51E+06	7.28E-15	5.58E-17	1.59E-22	2.38E-18	2.07E-15	1.36E-13	7.44E-18	4.58E-24	5.48E-22	1.94E-23	6.57E-29	3.20E-13	4.99E-26	2, 3, 4, 5, 6, 7	1, 11, 13, 2, 4, 5, 9	1, 11, 13, 2, 4, 5, 9	EHD4_MOUSE, SP:Q9EQP2	SP|Q9EQP2|EHD4_MOUSE
Q99KR7	PPIF_MOUSE	Peptidyl-prolyl cis-trans isomerase, mitochondrial OS=Mus musculus GN=Ppif PE=2 SV=1 [MASS=21737]	4	13	1	3.77E+05	4.64E+05	6.06E+05	4.52E+05	6.89E+05	5.25E+05	6.65E+05	5.07E+05	6.49E+05	8.31E+05	7.32E+05	6.15E+05	5.35E+05	6.35E-05	6.64E-07	6.71E-15	1.18E-08	1.16E-09	4.65E-08	4.52E-12	6.28E-09	2.08E-10	3.75E-16	1.64E-18	5.13E-07	1.85E-10	3, 3, 4, 5	1, 1, 13, 11, 6	1, 13, 11, 17, 6	PPIF_MOUSE, SP:Q99KR7	SP|Q99KR7|PPIF_MOUSE
Q91VN4	CHCH6_MOUSE	Coiled-coil-helix-coiled-coil-helix domain-containing protein 6 OS=Mus musculus GN=Chchd6 PE=2 SV=1 [MASS=29799]	1	2	0.99	7.97E+03	6.68E+03	0.00E+00	5.23E+03	7.06E+03	5.68E+03	3.64E+03	2.46E+03	6.27E+03	5.76E+03	5.73E+03	6.29E+03	6.16E+03	0.288	0.28	0.5	0.272	0.271	0.288	0.305	0.345	0.268	0.249	0.217	0.31	0.249	2	1	2	CHCH6_MOUSE, SP:Q91VN4	SP|Q91VN4|CHCH6_MOUSE
Q9CRD0	OCAD1_MOUSE	OCIA domain-containing protein 1 OS=Mus musculus GN=Ociad1 PE=1 SV=1 [MASS=27610]	1	9	0.99	9.34E+04	9.76E+04	6.05E+04	6.88E+04	9.22E+04	1.08E+05	7.80E+04	1.00E+05	7.38E+04	9.01E+04	7.88E+04	9.31E+04	7.87E+04	0.029	0.013	0.008	0.015	0.015	0.008	0.01	0.006	0.018	0.004	0.002	0.029	0.008	5	9	9	OCAD1_MOUSE, SP:Q9CRD0	SP|Q9CRD0|OCAD1_MOUSE
Q9DBL1	ACDSB_MOUSE	Short/branched chain specific acyl-CoA dehydrogenase, mitochondrial OS=Mus musculus GN=Acadsb PE=1 SV=1 [MASS=47874]	6	13	1	7.35E+05	5.79E+05	6.24E+05	6.08E+05	7.39E+05	7.53E+05	7.36E+05	5.67E+05	5.18E+05	6.03E+05	4.94E+05	5.60E+05	4.70E+05	4.44E-08	3.29E-08	2.43E-15	4.31E-11	3.15E-10	8.32E-11	3.34E-13	9.06E-10	1.25E-08	3.63E-12	3.65E-13	1.55E-06	2.05E-09	2, 3, 4, 5	13, 3, 6, 7, 8	13, 3, 6, 7, 8	ACDSB_MOUSE, SP:Q9DBL1	SP|Q9DBL1|ACDSB_MOUSE
P61982	1433G_MOUSE	14-3-3 protein gamma OS=Mus musculus GN=Ywhag PE=1 SV=2 [MASS=28302]	4	13	1	1.69E+06	1.43E+06	1.42E+06	1.49E+06	1.77E+06	1.56E+06	1.51E+06	1.64E+06	1.93E+06	1.88E+06	1.81E+06	1.91E+06	1.80E+06	3.08E-16	1.34E-17	2.85E-32	2.46E-24	6.28E-22	2.10E-20	6.36E-25	9.11E-25	3.35E-27	6.48E-34	1.36E-42	4.44E-18	1.25E-30	3, 4, 5, 5, 6	11, 11, 13, 8, 13	11, 11, 13, 13, 8	1433G_MOUSE, SP:P61982	SP|P61982|1433G_MOUSE
Q9D5T0	ATAD1_MOUSE	ATPase family AAA domain-containing protein 1 OS=Mus musculus GN=Atad1 PE=1 SV=1 [MASS=40744]	3	13	1	3.02E+05	2.56E+05	2.49E+05	2.76E+05	2.27E+05	2.18E+05	2.17E+05	2.52E+05	2.33E+05	2.09E+05	1.70E+05	2.03E+05	2.08E+05	3.03E-04	1.68E-04	4.11E-07	6.12E-06	3.11E-04	2.91E-04	4.86E-05	3.01E-05	9.05E-05	2.53E-05	1.38E-05	0.003	4.75E-05	2, 3, 4	1, 1, 10, 13	1, 1, 10, 13	ATAD1_MOUSE, SP:Q9D5T0	SP|Q9D5T0|ATAD1_MOUSE
Q9JLZ3	AUHM_MOUSE	Methylglutaconyl-CoA hydratase, mitochondrial OS=Mus musculus GN=Auh PE=2 SV=1 [MASS=33395]	3	11	1	2.75E+05	3.06E+05	3.50E+05	3.57E+05	3.60E+05	3.50E+05	4.94E+05	2.87E+05	2.94E+05	2.99E+05	3.13E+05	3.38E+05	2.76E+05	5.21E-04	4.16E-05	2.12E-09	3.23E-07	7.51E-06	6.20E-06	1.97E-09	8.98E-06	1.27E-05	5.85E-07	5.03E-09	1.42E-04	3.23E-06	3, 4	1, 11, 2	1, 11, 2	AUHM_MOUSE, SP:Q9JLZ3	SP|Q9JLZ3|AUHM_MOUSE
Q9CQR2	RS21_MOUSE	40S ribosomal protein S21 OS=Mus musculus GN=Rps21 PE=2 SV=1 [MASS=9141]	1	7	0.98	1.55E+04	1.16E+04	6.70E+04	1.86E+04	7.72E+04	5.61E+04	6.22E+04	2.69E+04	8.72E+04	9.38E+04	5.87E+04	7.65E+04	5.16E+04	0.22	0.223	0.005	0.128	0.022	0.04	0.018	0.094	0.011	0.003	0.006	0.042	0.026	3	7	7	RS21_MOUSE, SP:Q9CQR2	SP|Q9CQR2|RS21_MOUSE
Q3ULD5	MCCB_MOUSE	Methylcrotonoyl-CoA carboxylase beta chain, mitochondrial OS=Mus musculus GN=Mccc2 PE=2 SV=1 [MASS=61379]	9	13	1	9.78E+05	8.13E+05	7.50E+05	7.58E+05	1.03E+06	8.59E+05	9.04E+05	6.90E+05	6.10E+05	6.88E+05	6.44E+05	6.96E+05	5.52E+05	3.23E-10	6.84E-11	3.34E-18	1.79E-13	1.34E-13	3.75E-12	7.18E-16	1.33E-11	5.96E-10	8.01E-14	8.78E-17	9.41E-08	8.42E-11	2, 3, 4, 4, 6, 5	12, 12, 13, 2, 3, 7, 7, 13, 9	12, 12, 13, 13, 7, 2, 3, 7, 9	MCCB_MOUSE, SP:Q3ULD5	SP|Q3ULD5|MCCB_MOUSE
P35762	CD81_MOUSE	CD81 antigen OS=Mus musculus GN=Cd81 PE=1 SV=1 [MASS=25828]	1	13	0.99	2.02E+05	2.47E+05	1.28E+05	1.71E+05	1.82E+05	1.78E+05	1.88E+05	2.28E+05	1.61E+05	1.84E+05	1.97E+05	1.88E+05	2.35E+05	0.003	2.04E-04	1.85E-04	2.81E-04	0.001	9.28E-04	1.41E-04	6.59E-05	8.93E-04	6.77E-05	2.81E-06	0.003	1.62E-05	4	13	13	CD81_MOUSE, SP:P35762	SP|P35762|CD81_MOUSE
Q99LP6	GRPE1_MOUSE	GrpE protein homolog 1, mitochondrial OS=Mus musculus GN=Grpel1 PE=2 SV=1 [MASS=24307]	2	13	1	1.70E+05	1.51E+05	2.55E+05	2.04E+05	1.27E+05	1.31E+05	1.96E+05	1.71E+05	1.44E+05	2.56E+05	1.86E+05	1.22E+05	1.99E+05	0.005	0.003	2.59E-07	8.03E-05	0.005	0.004	9.96E-05	4.36E-04	0.002	3.34E-06	4.63E-06	0.015	6.18E-05	3, 4	10, 13	10, 13	GRPE1_MOUSE, SP:Q99LP6	SP|Q99LP6|GRPE1_MOUSE
Q8CGK3	LONM_MOUSE	Lon protease homolog, mitochondrial OS=Mus musculus GN=Lonp1 PE=1 SV=2 [MASS=105842]	13	13	1	1.76E+06	1.77E+06	1.64E+06	1.69E+06	1.57E+06	1.59E+06	1.56E+06	1.62E+06	1.60E+06	1.57E+06	1.63E+06	1.69E+06	1.87E+06	6.05E-17	1.27E-21	3.45E-37	1.25E-27	8.61E-20	5.72E-21	4.77E-26	7.96E-25	4.40E-23	4.38E-29	2.76E-39	2.46E-16	4.31E-32	2, 4, 3, 4, 5, 6, 7	1, 13, 10, 11, 13, 3, 5	10, 11, 13, 14, 3, 5	LONM_MOUSE, SP:Q8CGK3	SP|Q8CGK3|LONM_MOUSE
Q6P3A8	ODBB_MOUSE	2-oxoisovalerate dehydrogenase subunit beta, mitochondrial OS=Mus musculus GN=Bckdhb PE=2 SV=2 [MASS=42880]	1	12	1	3.36E+05	3.03E+05	3.90E+05	2.62E+05	2.79E+05	3.12E+05	2.86E+05	3.18E+05	2.33E+05	1.79E+05	2.36E+05	1.81E+05	1.66E+05	1.54E-04	4.77E-05	3.76E-10	1.14E-05	7.06E-05	2.01E-05	3.85E-06	4.22E-06	9.36E-05	8.66E-05	3.89E-07	0.004	2.40E-04	3, 6	1, 12	1, 12	ODBB_MOUSE, SP:Q6P3A8	SP|Q6P3A8|ODBB_MOUSE
P18760	COF1_MOUSE	Cofilin-1 OS=Mus musculus GN=Cfl1 PE=1 SV=3 [MASS=18559]	3	12	1	2.53E+05	2.45E+05	2.10E+05	1.95E+05	2.65E+05	2.31E+05	2.11E+05	2.58E+05	3.49E+05	3.26E+05	2.91E+05	3.22E+05	2.77E+05	8.26E-04	2.08E-04	2.49E-06	1.12E-04	9.96E-05	1.86E-04	5.54E-05	2.22E-05	2.06E-06	1.76E-07	1.45E-08	1.97E-04	3.01E-06	2, 4, 3, 4	1, 3, 12, 7, 8	1, 3, 12, 8, 9	COF1_MOUSE, SP:P18760	SP|P18760|COF1_MOUSE
P45591	COF2_MOUSE	Cofilin-2 OS=Mus musculus GN=Cfl2 PE=1 SV=1 [MASS=18709]	3	13	1	6.91E+05	6.31E+05	5.88E+05	6.47E+05	6.03E+05	6.88E+05	6.39E+05	6.90E+05	7.97E+05	8.36E+05	1.02E+06	8.03E+05	9.94E+05	1.08E-07	8.30E-09	1.54E-14	1.15E-11	1.10E-08	5.13E-10	1.10E-11	1.54E-11	2.28E-12	3.15E-16	9.39E-25	1.20E-08	9.42E-18	2, 3, 5	11, 13, 12, 3	11, 13, 12, 3	COF2_MOUSE, SP:P45591	SP|P45591|COF2_MOUSE
P50171	DHB8_MOUSE	Estradiol 17-beta-dehydrogenase 8 OS=Mus musculus GN=Hsd17b8 PE=1 SV=1 [MASS=26645]	5	13	1	3.81E+05	4.03E+05	2.95E+05	3.31E+05	3.84E+05	3.92E+05	3.54E+05	2.78E+05	2.72E+05	3.07E+05	3.04E+05	3.09E+05	3.06E+05	5.71E-05	3.10E-06	3.16E-08	7.68E-07	3.81E-06	1.86E-06	2.83E-07	1.14E-05	2.42E-05	4.08E-07	7.74E-09	2.57E-04	9.97E-07	2, 3	12, 13, 3, 6, 9	12, 13, 3, 6, 9	DHB8_MOUSE, SP:P50171	SP|P50171|DHB8_MOUSE
Q62446	FKBP3_MOUSE	FK506-binding protein 3 OS=Mus musculus GN=Fkbp3 PE=1 SV=2 [MASS=25148]	1	2	1	7.30E+04	5.31E+04	2.11E+04	4.24E+04	4.21E+04	4.66E+04	3.56E+04	3.56E+04	4.04E+04	3.67E+04	2.88E+04	5.95E+04	3.64E+04	0.046	0.05	0.073	0.043	0.069	0.055	0.056	0.065	0.059	0.044	0.04	0.064	0.05	2	2	2	FKBP3_MOUSE, SP:Q62446	SP|Q62446|FKBP3_MOUSE
P19324	SERPH_MOUSE	Serpin H1 OS=Mus musculus GN=Serpinh1 PE=1 SV=2 [MASS=46589]	4	13	1	1.16E+06	1.03E+06	8.35E+05	8.42E+05	8.40E+05	7.39E+05	5.74E+05	1.80E+06	1.73E+06	1.11E+06	9.32E+05	9.71E+05	1.05E+06	9.78E-12	3.32E-13	6.72E-20	1.04E-14	2.07E-11	1.18E-10	1.01E-10	8.98E-27	2.02E-24	4.48E-21	3.09E-23	4.32E-10	9.48E-19	3, 4, 4, 7, 5	11, 13, 2, 13, 4	11, 13, 13, 2, 4	SERPH_MOUSE, SP:P19324	SP|P19324|SERPH_MOUSE
Q8VDD5	MYH9_MOUSE	Myosin-9 OS=Mus musculus GN=Myh9 PE=1 SV=4 [MASS=226371]	8	13	1	5.88E+05	5.60E+05	4.81E+05	5.80E+05	5.48E+05	5.05E+05	5.47E+05	7.31E+05	7.23E+05	6.74E+05	7.29E+05	5.99E+05	7.77E+05	8.01E-07	4.86E-08	2.67E-12	1.06E-10	4.45E-08	7.35E-08	2.63E-10	3.33E-12	1.78E-11	1.33E-13	9.74E-19	6.57E-07	1.87E-14	2, 3, 3, 3, 4, 4, 5	1, 2, 1, 4, 13, 2, 3, 5, 4, 5, 8	1, 2, 13, 2, 3, 5, 4, 5, 6, 8	MYH9_MOUSE, SP:Q8VDD5	SP|Q8VDD5|MYH9_MOUSE
Q9D1I5	MCEE_MOUSE	Methylmalonyl-CoA epimerase, mitochondrial OS=Mus musculus GN=Mcee PE=2 SV=1 [MASS=19017]	2	11	1	6.10E+05	5.47E+05	5.38E+05	6.30E+05	4.34E+05	4.65E+05	4.62E+05	4.73E+05	3.79E+05	3.80E+05	4.98E+05	4.98E+05	4.23E+05	5.52E-07	7.38E-08	2.05E-13	2.15E-11	1.01E-06	2.44E-07	6.21E-09	1.82E-08	8.37E-07	2.13E-08	2.87E-13	5.36E-06	1.23E-08	6	11, 8	11, 8	MCEE_MOUSE, SP:Q9D1I5	SP|Q9D1I5|MCEE_MOUSE
Q9CXJ4	ABCB8_MOUSE	ATP-binding cassette sub-family B member 8, mitochondrial OS=Mus musculus GN=Abcb8 PE=2 SV=1 [MASS=77999]	5	11	1	4.51E+05	4.10E+05	3.05E+05	3.51E+05	3.93E+05	4.39E+05	3.51E+05	3.02E+05	2.68E+05	2.83E+05	2.64E+05	3.23E+05	2.93E+05	1.38E-05	2.65E-06	1.94E-08	4.00E-07	3.10E-06	5.10E-07	3.36E-07	5.38E-06	2.86E-05	1.27E-06	7.32E-08	1.95E-04	1.70E-06	2, 3, 4, 6	11, 2, 3, 9	11, 2, 4, 9	ABCB8_MOUSE, SP:Q9CXJ4	SP|Q9CXJ4|ABCB8_MOUSE
P61205	ARF2_MOUSE	ADP-ribosylation factor 3 OS=Mus musculus GN=Arf3 PE=2 SV=2 [MASS=20601]	2	13	1	4.32E+05	3.91E+05	3.06E+05	3.41E+05	3.77E+05	3.53E+05	3.17E+05	3.98E+05	3.60E+05	3.22E+05	3.62E+05	3.97E+05	3.71E+05	2.06E-05	4.40E-06	2.00E-08	5.72E-07	4.79E-06	5.82E-06	1.16E-06	2.20E-07	1.56E-06	2.25E-07	4.09E-10	4.32E-05	8.89E-08	4	13	13	ARF2_MOUSE, SP:Q8BSL7	SP|Q8BSL7|ARF2_MOUSE,SP|P84078|ARF1_MOUSE,SP|P61205|ARF3_MOUSE
O35857	TIM44_MOUSE	Mitochondrial import inner membrane translocase subunit TIM44 OS=Mus musculus GN=Timm44 PE=2 SV=1 [MASS=51176]	4	13	1	2.56E+05	2.28E+05	2.71E+05	2.98E+05	1.83E+05	1.70E+05	2.21E+05	2.04E+05	1.87E+05	2.27E+05	2.49E+05	1.69E+05	2.35E+05	7.67E-04	3.25E-04	1.07E-07	2.49E-06	9.75E-04	0.001	3.86E-05	1.40E-04	3.67E-04	1.08E-05	1.42E-07	0.005	1.51E-05	2, 3, 3, 4	13, 3, 5, 6	13, 3, 5, 6	TIM44_MOUSE, SP:O35857	SP|O35857|TIM44_MOUSE
P11087	CO1A1_MOUSE	Collagen alpha-1(I) chain OS=Mus musculus GN=Col1a1 PE=1 SV=4 [MASS=138031]	2	13	1	2.60E+05	2.38E+05	1.46E+05	1.32E+05	1.83E+05	1.53E+05	1.54E+05	1.95E+05	2.21E+05	2.06E+05	1.85E+05	1.84E+05	2.48E+05	7.24E-04	2.59E-04	7.18E-05	0.001	0.001	0.002	4.94E-04	1.99E-04	1.28E-04	2.71E-05	5.37E-06	0.004	9.82E-06	3, 4	13, 3	16	CO1A1_MOUSE, SP:P11087	SP|P11087|CO1A1_MOUSE
Q99J39	DCMC_MOUSE	Malonyl-CoA decarboxylase, mitochondrial OS=Mus musculus GN=Mlycd PE=2 SV=1 [MASS=54736]	7	13	1	6.39E+05	6.11E+05	5.40E+05	5.72E+05	6.01E+05	5.87E+05	5.59E+05	5.02E+05	4.36E+05	4.49E+05	5.09E+05	5.60E+05	4.17E+05	2.95E-07	1.27E-08	1.20E-13	1.34E-10	1.06E-08	7.44E-09	1.64E-10	6.37E-09	1.37E-07	1.22E-09	1.26E-13	1.50E-06	1.42E-08	3, 4, 5	11, 13, 2, 4, 3, 4, 8	11, 13, 2, 4, 3, 4, 8	DCMC_MOUSE, SP:Q99J39	SP|Q99J39|DCMC_MOUSE
Q9CQW2	ARL8B_MOUSE	ADP-ribosylation factor-like protein 8B OS=Mus musculus GN=Arl8b PE=2 SV=1 [MASS=21539]	1	1	0.89	4.45E+04	2.84E+04	3.51E+04	3.04E+04	2.70E+04	2.55E+04	3.56E+04	2.99E+04	3.51E+04	4.16E+04	3.38E+04	3.71E+04	2.95E+04	0.094	0.116	0.031	0.073	0.118	0.119	0.057	0.083	0.072	0.035	0.03	0.115	0.07	3	1	1	ARL8B_MOUSE, SP:Q9CQW2	SP|Q9CQW2|ARL8B_MOUSE,SP|Q8VEH3|ARL8A_MOUSE
P99024	TBB5_MOUSE	Tubulin beta-5 chain OS=Mus musculus GN=Tubb5 PE=1 SV=1 [MASS=49671]	3	13	1	4.27E+05	3.10E+05	3.13E+05	3.20E+05	2.94E+05	2.84E+05	2.69E+05	5.63E+05	5.11E+05	4.95E+05	4.50E+05	3.47E+05	4.77E+05	2.24E-05	3.62E-05	1.27E-08	1.16E-06	4.50E-05	4.10E-05	6.48E-06	9.41E-10	1.38E-08	2.07E-10	3.15E-12	1.17E-04	1.52E-09	2, 3, 4	13, 5, 12, 7	12, 6, 13, 7	TBB5_MOUSE, SP:P99024	SP|P99024|TBB5_MOUSE
P17742	PPIA_MOUSE	Peptidyl-prolyl cis-trans isomerase A OS=Mus musculus GN=Ppia PE=1 SV=2 [MASS=17971]	5	13	1	1.05E+06	1.34E+06	1.69E+06	1.39E+06	1.62E+06	1.43E+06	1.85E+06	1.58E+06	1.97E+06	1.64E+06	1.69E+06	1.57E+06	2.01E+06	8.94E-11	1.59E-16	1.10E-37	9.88E-23	3.44E-20	7.93E-19	5.47E-30	9.68E-24	1.19E-27	7.49E-30	8.09E-40	3.57E-15	2.22E-33	4, 4, 5, 5	13, 2, 5, 11	11, 5, 13, 2	PPIA_MOUSE, SP:P17742	SP|P17742|PPIA_MOUSE
P27546	MAP4_MOUSE	Microtubule-associated protein 4 OS=Mus musculus GN=Map4 PE=1 SV=3 [MASS=117428]	6	12	1	3.66E+05	3.09E+05	2.78E+05	3.20E+05	3.39E+05	3.24E+05	3.29E+05	3.84E+05	4.25E+05	3.72E+05	4.48E+05	4.23E+05	4.44E+05	7.96E-05	3.92E-05	8.00E-08	1.25E-06	1.35E-05	1.32E-05	7.50E-07	3.34E-07	1.96E-07	2.90E-08	3.74E-12	2.50E-05	5.15E-09	3, 4, 5	12, 2, 4, 5, 7	12, 2, 4, 5, 7	MAP4_MOUSE, SP:P27546	SP|P27546|MAP4_MOUSE
P00416	COX3_MOUSE	Cytochrome c oxidase subunit 3 OS=Mus musculus GN=Mtco3 PE=3 SV=1 [MASS=29937]	1	13	1	3.08E+05	3.95E+05	1.93E+05	4.87E+05	1.62E+05	3.45E+05	2.92E+05	2.47E+05	3.24E+05	5.02E+05	3.02E+05	3.00E+05	2.84E+05	2.80E-04	4.30E-06	6.43E-06	3.67E-09	0.002	7.81E-06	3.22E-06	3.58E-05	5.10E-06	2.22E-10	1.05E-08	3.35E-04	2.61E-06	3, 5	13	13	COX3_MOUSE, SP:P00416	SP|P00416|COX3_MOUSE
Q68FD5	CLH_MOUSE	Clathrin heavy chain 1 OS=Mus musculus GN=Cltc PE=1 SV=3 [MASS=191555]	20	13	1	1.73E+06	1.60E+06	1.48E+06	1.55E+06	1.50E+06	1.48E+06	1.33E+06	1.93E+06	2.08E+06	1.84E+06	1.79E+06	1.70E+06	1.98E+06	1.02E-16	1.12E-19	4.70E-34	1.49E-25	5.72E-19	1.41E-19	1.76E-22	3.86E-29	1.88E-29	1.21E-33	6.17E-43	2.19E-16	5.37E-34	2, 3, 4, 5, 5, 6, 6	1, 10, 11, 12, 13, 3, 4, 7, 9	1, 10, 11, 12, 13, 3, 4, 7, 9	CLH_MOUSE, SP:Q68FD5	SP|Q68FD5|CLH_MOUSE
Q60605	MYL6_MOUSE	Myosin light polypeptide 6 OS=Mus musculus GN=Myl6 PE=1 SV=3 [MASS=16930]	3	13	1	3.32E+05	4.20E+05	4.43E+05	3.92E+05	2.96E+05	3.61E+05	3.80E+05	6.15E+05	5.58E+05	4.70E+05	5.24E+05	3.02E+05	4.94E+05	1.58E-04	2.00E-06	2.20E-11	9.25E-08	4.31E-05	4.71E-06	1.20E-07	1.96E-10	3.47E-09	6.00E-10	8.43E-14	3.01E-04	8.84E-10	2, 3, 5	13, 8	13, 8	MYL6_MOUSE, SP:Q60605	SP|Q60605|MYL6_MOUSE
Q80Y14	GLRX5_MOUSE	Glutaredoxin-related protein 5 OS=Mus musculus GN=Glrx5 PE=2 SV=2 [MASS=16292]	1	9	1	2.09E+04	4.50E+04	2.15E+05	2.85E+04	2.18E+05	1.75E+05	1.79E+05	4.39E+04	2.52E+05	2.96E+05	2.79E+05	1.87E+05	1.37E+05	0.185	0.067	1.97E-06	0.082	3.75E-04	0.001	1.91E-04	0.047	4.72E-05	6.92E-07	3.34E-08	0.003	7.58E-04	4, 6	2, 9	2, 9	GLRX5_MOUSE, SP:Q80Y14	SP|Q80Y14|GLRX5_MOUSE
Q91YQ5	RPN1_MOUSE	Dolichyl-diphosphooligosaccharide--protein glycosyltransferase subunit 1 OS=Mus musculus GN=Rpn1 PE=2 SV=1 [MASS=68528]	1	2	1	2.22E+04	2.44E+04	2.19E+04	2.80E+04	3.33E+04	2.79E+04	3.65E+04	3.62E+04	2.06E+04	3.06E+04	3.82E+04	2.39E+04	2.51E+04	0.177	0.134	0.07	0.082	0.094	0.109	0.054	0.063	0.132	0.06	0.022	0.167	0.087	3	2	2	RPN1_MOUSE, SP:Q91YQ5	SP|Q91YQ5|RPN1_MOUSE
P34914	HYES_MOUSE	Epoxide hydrolase 2 OS=Mus musculus GN=Ephx2 PE=1 SV=2 [MASS=62515]	17	13	1	4.70E+06	3.96E+06	4.21E+06	4.18E+06	3.84E+06	3.74E+06	3.73E+06	3.89E+06	3.02E+06	3.39E+06	3.68E+06	3.21E+06	3.27E+06	7.51E-42	1.40E-45	0	0	9.90E-42	1.68E-44	0	0	6.26E-32	1.40E-45	0	1.38E-23	8.41E-45	2, 3, 3, 5, 4, 4, 5, 4, 8, 5, 5, 6, 7	1, 1, 11, 1, 13, 12, 12, 13, 13, 3, 3, 13, 4, 8, 8, 9	1, 1, 11, 1, 13, 12, 12, 13, 13, 13, 4, 4, 5, 8, 8, 9	HYES_MOUSE, SP:P34914	SP|P34914|HYES_MOUSE
Q9JK42	PDK2_MOUSE	[Pyruvate dehydrogenase [lipoamide]] kinase isozyme 2, mitochondrial OS=Mus musculus GN=Pdk2 PE=1 SV=1 [MASS=46068]	8	13	1	8.34E+05	7.56E+05	7.67E+05	8.41E+05	9.31E+05	9.99E+05	9.60E+05	6.15E+05	6.27E+05	5.86E+05	8.15E+05	8.66E+05	7.23E+05	5.95E-09	3.10E-10	1.64E-18	1.08E-14	1.71E-12	8.33E-14	1.05E-16	1.61E-10	3.73E-10	4.88E-12	1.38E-20	3.16E-09	1.49E-13	2, 3, 4, 5, 7	10, 13, 2, 6, 8, 9	10, 13, 2, 6, 8, 9	PDK2_MOUSE, SP:Q9JK42	SP|Q9JK42|PDK2_MOUSE
P55096	ABCD3_MOUSE	ATP-binding cassette sub-family D member 3 OS=Mus musculus GN=Abcd3 PE=1 SV=2 [MASS=75482]	2	2	1	1.06E+05	7.10E+04	5.60E+04	7.40E+04	7.90E+04	8.01E+04	6.32E+04	6.64E+04	5.79E+04	4.74E+04	6.52E+04	7.06E+04	8.06E+04	0.021	0.029	0.009	0.012	0.021	0.018	0.017	0.019	0.031	0.027	0.004	0.049	0.007	3, 5	2	2	ABCD3_MOUSE, SP:P55096	SP|P55096|ABCD3_MOUSE
Q9R0H0	ACOX1_MOUSE	Peroxisomal acyl-coenzyme A oxidase 1 OS=Mus musculus GN=Acox1 PE=1 SV=4 [MASS=74634]	2	1	1	6.81E+04	4.39E+04	3.72E+04	5.16E+04	4.63E+04	4.78E+04	4.46E+04	4.47E+04	3.40E+04	3.95E+04	4.72E+04	3.20E+04	3.51E+04	0.052	0.069	0.028	0.03	0.062	0.054	0.04	0.046	0.079	0.04	0.015	0.136	0.056	2, 3	1	1	ACOX1_MOUSE, SP:Q9R0H0	SP|Q9R0H0|ACOX1_MOUSE
P21981	TGM2_MOUSE	Protein-glutamine gamma-glutamyltransferase 2 OS=Mus musculus GN=Tgm2 PE=1 SV=4 [MASS=77061]	10	13	1	1.07E+06	1.05E+06	1.14E+06	9.60E+05	9.96E+05	9.71E+05	9.05E+05	1.16E+06	1.45E+06	1.52E+06	1.44E+06	1.45E+06	1.80E+06	5.67E-11	1.58E-13	2.94E-26	2.00E-16	3.28E-13	1.88E-13	8.07E-16	3.62E-18	4.62E-21	3.84E-28	9.95E-35	2.90E-14	1.12E-30	2, 3, 4, 5	1, 12, 13, 3, 5, 6, 7, 8, 9	1, 12, 13, 3, 5, 6, 7, 8, 9	TGM2_MOUSE, SP:P21981	SP|P21981|TGM2_MOUSE
Q9CXI0	COQ5_MOUSE	Ubiquinone biosynthesis methyltransferase COQ5, mitochondrial OS=Mus musculus GN=Coq5 PE=2 SV=2 [MASS=37335]	4	13	1	3.69E+05	4.50E+05	3.79E+05	3.95E+05	4.01E+05	4.20E+05	3.17E+05	3.93E+05	4.07E+05	3.83E+05	2.99E+05	2.89E+05	2.59E+05	7.31E-05	9.02E-07	4.29E-10	7.62E-08	2.40E-06	8.44E-07	1.10E-06	2.43E-07	3.41E-07	1.74E-08	9.84E-09	3.89E-04	5.98E-06	2, 4, 4, 6, 5	1, 13, 2, 4	1, 13, 2, 4	COQ5_MOUSE, SP:Q9CXI0	SP|Q9CXI0|COQ5_MOUSE
Q99MR8	MCCA_MOUSE	Methylcrotonoyl-CoA carboxylase subunit alpha, mitochondrial OS=Mus musculus GN=Mccc1 PE=2 SV=2 [MASS=79343]	17	13	1	2.19E+06	2.29E+06	1.94E+06	2.17E+06	2.40E+06	2.35E+06	2.35E+06	1.84E+06	1.51E+06	1.57E+06	1.82E+06	1.85E+06	1.42E+06	1.31E-20	2.43E-27	1.68E-43	6.62E-35	2.84E-29	5.73E-30	6.15E-38	8.70E-28	7.12E-22	5.34E-29	1.37E-43	1.12E-17	8.34E-25	2, 3, 4, 4, 6, 6, 7	1, 1, 13, 10, 11, 12, 13, 2, 6, 9	1, 1, 13, 10, 11, 12, 13, 2, 6, 9	MCCA_MOUSE, SP:Q99MR8	SP|Q99MR8|MCCA_MOUSE
Q9D0L4	ADCK1_MOUSE	Uncharacterized aarF domain-containing protein kinase 1 OS=Mus musculus GN=Adck1 PE=2 SV=1 [MASS=59736]	1	4	0.97	3.04E+04	1.43E+04	1.95E+04	3.08E+04	1.95E+04	1.27E+04	1.39E+04	1.37E+04	2.00E+04	2.46E+04	1.39E+04	1.45E+04	1.77E+04	0.138	0.198	0.083	0.072	0.156	0.205	0.163	0.176	0.136	0.082	0.119	0.226	0.128	5	4	4	ADCK1_MOUSE, SP:Q9D0L4	SP|Q9D0L4|ADCK1_MOUSE
Q9D6K8	FUND2_MOUSE	FUN14 domain-containing protein 2 OS=Mus musculus GN=Fundc2 PE=2 SV=1 [MASS=16564]	1	1	0.97	2.59E+04	1.43E+04	3.98E+04	2.58E+04	2.46E+04	2.32E+04	2.70E+04	2.31E+04	2.68E+04	2.41E+04	2.84E+04	3.10E+04	2.32E+04	0.157	0.197	0.024	0.09	0.128	0.13	0.084	0.111	0.101	0.084	0.042	0.136	0.096	2	1	1	FUND2_MOUSE, SP:Q9D6K8	SP|Q9D6K8|FUND2_MOUSE
O35381	AN32A_MOUSE	Acidic leucine-rich nuclear phosphoprotein 32 family member A OS=Mus musculus GN=Anp32a PE=1 SV=1 [MASS=28537]	1	5	1	6.15E+03	7.67E+03	3.97E+04	1.61E+04	5.11E+04	3.52E+04	4.05E+04	1.53E+04	5.70E+04	6.55E+04	7.00E+04	3.75E+04	2.75E+04	0.312	0.266	0.024	0.145	0.051	0.082	0.045	0.16	0.031	0.011	0.003	0.113	0.077	3	5	5	AN32A_MOUSE, SP:O35381	SP|O35381|AN32A_MOUSE
Q8R2G4	NAR3_MOUSE	Ecto-ADP-ribosyltransferase 3 OS=Mus musculus GN=Art3 PE=2 SV=2 [MASS=42015]	4	4	1	3.76E+05	3.04E+05	2.25E+05	2.19E+05	2.93E+05	2.63E+05	2.31E+05	2.15E+05	1.48E+05	1.43E+05	1.58E+05	2.07E+05	1.69E+05	6.67E-05	4.81E-05	1.26E-06	5.35E-05	5.08E-05	8.18E-05	2.93E-05	1.07E-04	0.001	4.19E-04	2.99E-05	0.002	2.24E-04	2, 3, 5, 6	2, 4, 3, 4	2, 4, 3, 4	NAR3_MOUSE, SP:Q8R2G4	SP|Q8R2G4|NAR3_MOUSE
P49813	TMOD1_MOUSE	Tropomodulin-1 OS=Mus musculus GN=Tmod1 PE=2 SV=1 [MASS=40484]	8	13	1	1.29E+06	1.29E+06	1.07E+06	1.15E+06	1.13E+06	1.04E+06	1.16E+06	1.21E+06	1.15E+06	1.10E+06	1.13E+06	1.13E+06	1.13E+06	6.63E-13	4.00E-16	6.90E-25	2.45E-19	1.10E-14	2.61E-14	1.26E-19	9.00E-19	4.97E-17	7.16E-21	1.47E-27	1.68E-11	5.55E-20	2, 5, 3, 4, 4, 5, 5	11, 11, 12, 13, 2, 2, 6, 3	11, 11, 12, 13, 2, 2, 6, 3	TMOD1_MOUSE, SP:P49813	SP|P49813|TMOD1_MOUSE
Q9WVA2	TIM8A_MOUSE	Mitochondrial import inner membrane translocase subunit Tim8 A OS=Mus musculus GN=Timm8a1 PE=1 SV=1 [MASS=11042]	1	2	1	1.00E+05	9.97E+04	8.71E+04	8.81E+04	7.98E+04	6.99E+04	9.40E+04	8.50E+04	5.43E+04	1.05E+05	8.34E+04	8.41E+04	8.04E+04	0.024	0.012	0.002	0.007	0.021	0.025	0.005	0.01	0.035	0.002	0.002	0.035	0.007	2	2	2	TIM8A_MOUSE, SP:Q9WVA2	SP|Q9WVA2|TIM8A_MOUSE
P19157	GSTP1_MOUSE	Glutathione S-transferase P 1 OS=Mus musculus GN=Gstp1 PE=1 SV=2 [MASS=23609]	2	13	1	5.86E+05	4.50E+05	4.30E+05	4.57E+05	4.86E+05	4.05E+05	4.82E+05	3.79E+05	3.48E+05	2.88E+05	3.42E+05	4.56E+05	3.32E+05	1.07E-06	1.15E-06	5.99E-11	1.42E-08	3.14E-07	1.63E-06	4.28E-09	5.17E-07	2.74E-06	1.08E-06	2.10E-09	1.39E-05	4.66E-07	3, 7	1, 13	1, 13	GSTP1_MOUSE, SP:P19157	SP|P19157|GSTP1_MOUSE
Q8VE22	RT23_MOUSE	28S ribosomal protein S23, mitochondrial OS=Mus musculus GN=Mrps23 PE=2 SV=1 [MASS=20348]	1	7	0.99	9.11E+04	6.40E+04	2.39E+04	3.28E+04	4.42E+04	4.65E+04	3.10E+04	3.55E+04	4.81E+04	4.29E+04	2.89E+04	4.53E+04	2.84E+04	0.029	0.035	0.061	0.065	0.064	0.055	0.069	0.065	0.043	0.032	0.04	0.091	0.073	4	7	7	RT23_MOUSE, SP:Q8VE22	SP|Q8VE22|RT23_MOUSE
Q3URD3	SLMAP_MOUSE	Sarcolemmal membrane-associated protein OS=Mus musculus GN=Slmap PE=1 SV=2 [MASS=96933]	5	9	1	4.54E+05	3.93E+05	3.21E+05	2.90E+05	3.86E+05	3.42E+05	3.06E+05	4.22E+05	5.57E+05	5.79E+05	5.83E+05	5.27E+05	6.28E+05	1.28E-05	4.16E-06	9.19E-09	3.52E-06	3.72E-06	7.87E-06	1.73E-06	9.50E-08	3.36E-09	7.35E-12	3.02E-15	3.01E-06	5.48E-12	2, 3, 4	2, 3, 7, 8, 9	2, 3, 7, 8, 9	SLMAP_MOUSE, SP:Q3URD3	SP|Q3URD3|SLMAP_MOUSE
O35206	COFA1_MOUSE	Collagen alpha-1(XV) chain OS=Mus musculus GN=Col15a1 PE=1 SV=2 [MASS=140471]	5	9	1	1.96E+05	1.90E+05	2.04E+05	1.89E+05	1.43E+05	1.58E+05	1.66E+05	2.44E+05	2.73E+05	2.01E+05	2.31E+05	1.83E+05	3.25E+05	0.003	9.42E-04	3.69E-06	1.40E-04	0.003	0.002	3.13E-04	3.67E-05	2.33E-05	3.23E-05	4.00E-07	0.004	4.86E-07	2, 3	2, 4, 6, 9	2, 5, 6, 9	COFA1_MOUSE, SP:O35206	SP|O35206|COFA1_MOUSE
Q8VCF0	MAVS_MOUSE	Mitochondrial antiviral-signaling protein OS=Mus musculus GN=Mavs PE=1 SV=1 [MASS=53399]	3	4	1	6.12E+04	5.16E+04	1.54E+04	3.24E+04	4.28E+04	4.18E+04	3.51E+04	5.17E+04	2.85E+04	4.12E+04	3.91E+04	4.63E+04	4.80E+04	0.061	0.052	0.11	0.066	0.067	0.065	0.057	0.034	0.094	0.035	0.021	0.089	0.029	2, 3, 4	1, 4	1, 4	MAVS_MOUSE, SP:Q8VCF0	SP|Q8VCF0|MAVS_MOUSE
Q8BFP9	PDK1_MOUSE	[Pyruvate dehydrogenase [lipoamide]] kinase isozyme 1, mitochondrial OS=Mus musculus GN=Pdk1 PE=2 SV=1 [MASS=48926]	2	4	1	8.30E+04	9.95E+04	1.28E+05	1.35E+05	1.02E+05	1.16E+05	1.53E+05	7.82E+04	8.60E+04	1.07E+05	1.41E+05	7.38E+04	1.65E+05	0.036	0.012	1.83E-04	0.001	0.01	0.006	4.98E-04	0.012	0.011	0.002	5.71E-05	0.045	2.37E-04	3	1, 4	1, 4	PDK1_MOUSE, SP:Q8BFP9	SP|Q8BFP9|PDK1_MOUSE
P53395	ODB2_MOUSE	Lipoamide acyltransferase component of branched-chain alpha-keto acid dehydrogenase complex, mitochondrial OS=Mus musculus GN=Dbt PE=2 SV=1 [MASS=53160]	5	13	1	6.01E+05	4.39E+05	6.77E+05	6.95E+05	6.43E+05	6.06E+05	6.53E+05	4.71E+05	4.42E+05	4.99E+05	6.25E+05	5.03E+05	4.67E+05	6.58E-07	1.23E-06	1.59E-16	2.01E-12	3.60E-09	4.52E-09	5.91E-12	1.90E-08	1.13E-07	1.57E-10	2.91E-16	4.83E-06	2.21E-09	2, 3, 4, 5, 6	11, 12, 5, 6, 11	11, 6, 12, 13, 5	ODB2_MOUSE, SP:P53395	SP|P53395|ODB2_MOUSE
Q61598	GDIB_MOUSE	Rab GDP dissociation inhibitor beta OS=Mus musculus GN=Gdi2 PE=1 SV=1 [MASS=50537]	6	13	1	9.12E+05	9.11E+05	8.37E+05	8.89E+05	7.06E+05	6.91E+05	6.74E+05	1.04E+06	8.46E+05	8.46E+05	8.33E+05	7.54E+05	8.85E+05	1.18E-09	5.29E-12	4.64E-20	1.83E-15	6.46E-10	4.00E-10	2.68E-12	1.44E-16	4.22E-13	1.39E-16	4.44E-21	2.94E-08	3.21E-16	3, 4, 4, 5, 7	1, 12, 13, 3, 13, 4, 8	1, 12, 13, 13, 3, 4, 8	GDIB_MOUSE, SP:Q61598	SP|Q61598|GDIB_MOUSE
Q9WTP7	KAD3_MOUSE	GTP:AMP phosphotransferase mitochondrial OS=Mus musculus GN=Ak3 PE=1 SV=3 [MASS=25426]	7	13	1	7.50E+05	8.13E+05	7.59E+05	8.43E+05	7.50E+05	6.91E+05	7.61E+05	7.82E+05	6.92E+05	7.10E+05	7.51E+05	6.82E+05	7.12E+05	3.06E-08	6.79E-11	2.16E-18	9.38E-15	2.03E-10	4.07E-10	1.15E-13	6.53E-13	4.69E-11	3.10E-14	3.32E-19	1.25E-07	2.06E-13	2, 3, 3, 4, 3, 5, 4	12, 13, 3, 12, 5, 6, 9	12, 12, 3, 13, 5, 6, 9	KAD3_MOUSE, SP:Q9WTP7	SP|Q9WTP7|KAD3_MOUSE
P70414	NAC1_MOUSE	Sodium/calcium exchanger 1 OS=Mus musculus GN=Slc8a1 PE=1 SV=1 [MASS=108035]	2	10	1	1.94E+05	1.18E+05	8.16E+04	1.03E+05	1.13E+05	1.05E+05	1.07E+05	1.10E+05	1.10E+05	1.12E+05	1.27E+05	1.21E+05	1.23E+05	0.003	0.007	0.002	0.004	0.008	0.008	0.003	0.004	0.005	0.001	1.23E-04	0.015	0.001	3, 4	10, 6	10, 6	NAC1_MOUSE, SP:P70414	SP|P70414|NAC1_MOUSE
Q149B8	CA170_MOUSE	Uncharacterized protein C1orf170 homolog OS=Mus musculus PE=2 SV=1 [MASS=88451]	3	13	1	3.07E+05	2.55E+05	1.74E+05	2.05E+05	2.05E+05	2.51E+05	1.95E+05	1.99E+05	2.29E+05	1.55E+05	1.77E+05	1.92E+05	1.57E+05	2.67E-04	1.58E-04	1.59E-05	7.71E-05	5.28E-04	1.06E-04	1.01E-04	1.71E-04	9.77E-05	2.22E-04	7.81E-06	0.003	3.28E-04	2, 3, 5	13, 2, 5	13, 2, 5	CA170_MOUSE, SP:Q149B8	SP|Q149B8|CA170_MOUSE
P82347	SGCD_MOUSE	Delta-sarcoglycan OS=Mus musculus GN=Sgcd PE=1 SV=1 [MASS=32133]	1	13	1	6.87E+04	6.17E+04	5.15E+04	5.82E+04	6.02E+04	5.20E+04	6.12E+04	4.63E+04	4.42E+04	4.39E+04	5.25E+04	5.80E+04	6.14E+04	0.05	0.038	0.012	0.022	0.038	0.046	0.019	0.042	0.051	0.031	0.009	0.066	0.016	3	13	13	SGCD_MOUSE, SP:P82347	SP|P82347|SGCD_MOUSE
O70622	RTN2_MOUSE	Reticulon-2 OS=Mus musculus GN=Rtn2 PE=1 SV=1 [MASS=51346]	3	13	1	4.51E+05	4.69E+05	4.47E+05	4.13E+05	4.58E+05	4.81E+05	4.29E+05	3.85E+05	2.68E+05	2.53E+05	2.98E+05	3.00E+05	2.37E+05	1.39E-05	5.76E-07	2.08E-11	4.36E-08	6.02E-07	1.72E-07	2.25E-08	3.38E-07	3.20E-05	5.19E-06	1.56E-08	3.32E-04	1.46E-05	2, 4	1, 13, 8	1, 13, 8	RTN2_MOUSE, SP:O70622	SP|O70622|RTN2_MOUSE
Q922Q1	MOSC2_MOUSE	MOSC domain-containing protein 2, mitochondrial OS=Mus musculus GN=Mosc2 PE=1 SV=1 [MASS=38194]	1	1	0.95	8.46E+03	1.26E+04	1.81E+04	1.99E+04	1.16E+04	1.36E+04	2.74E+04	2.08E+04	2.27E+04	1.71E+04	1.48E+04	9.47E+03	2.13E+04	0.282	0.211	0.089	0.119	0.217	0.194	0.082	0.123	0.12	0.121	0.105	0.271	0.104	3	1	1	MOSC2_MOUSE, SP:Q922Q1	SP|Q922Q1|MOSC2_MOUSE
P02468	LAMC1_MOUSE	Laminin subunit gamma-1 OS=Mus musculus GN=Lamc1 PE=1 SV=2 [MASS=177298]	12	13	1	1.71E+06	1.84E+06	1.41E+06	1.61E+06	1.71E+06	1.64E+06	1.66E+06	1.50E+06	1.56E+06	1.48E+06	1.32E+06	1.55E+06	1.67E+06	1.73E-16	3.91E-22	3.23E-32	3.06E-26	2.06E-21	2.01E-21	2.09E-27	6.04E-23	1.44E-22	2.77E-27	6.73E-32	3.97E-15	9.77E-29	2, 3, 4, 5, 6	10, 13, 2, 4	10, 12, 13, 4	LAMC1_MOUSE, SP:P02468	SP|P02468|LAMC1_MOUSE
P47757	CAPZB_MOUSE	F-actin-capping protein subunit beta OS=Mus musculus GN=Capzb PE=1 SV=3 [MASS=31345]	2	9	1	4.56E+04	1.51E+04	2.98E+05	6.21E+04	3.76E+05	2.80E+05	2.55E+05	4.55E+04	5.23E+05	4.63E+05	2.43E+05	3.16E+05	1.53E+05	0.092	0.203	2.88E-08	0.019	4.96E-06	4.76E-05	1.14E-05	0.045	1.02E-08	7.74E-10	2.14E-07	2.27E-04	3.89E-04	2, 8	2, 9	2, 9	CAPZB_MOUSE, SP:P47757	SP|P47757|CAPZB_MOUSE
P06728	APOA4_MOUSE	Apolipoprotein A-IV OS=Mus musculus GN=Apoa4 PE=2 SV=2 [MASS=45029]	1	1	0.99	2.67E+04	1.62E+04	9.97E+03	1.03E+04	9.26E+03	2.31E+04	1.67E+04	1.07E+04	2.06E+04	1.45E+04	9.47E+03	1.76E+04	1.17E+04	0.153	0.182	0.171	0.197	0.243	0.13	0.138	0.203	0.131	0.141	0.158	0.203	0.175	2	1	1	APOA4_MOUSE, SP:P06728	SP|P06728|APOA4_MOUSE
Q00623	APOA1_MOUSE	Apolipoprotein A-I OS=Mus musculus GN=Apoa1 PE=1 SV=1 [MASS=30587]	4	13	1	5.38E+05	4.44E+05	6.04E+05	5.59E+05	4.90E+05	6.76E+05	7.76E+05	5.36E+05	5.99E+05	5.02E+05	6.55E+05	8.55E+05	5.62E+05	2.49E-06	1.14E-06	7.55E-15	2.55E-10	2.38E-07	7.34E-10	1.06E-13	2.55E-09	1.01E-09	1.85E-10	9.39E-17	4.52E-09	7.15E-11	2, 5, 3, 4	13, 4, 11, 8	11, 4, 13, 8	APOA1_MOUSE, SP:Q00623	SP|Q00623|APOA1_MOUSE
Q9JKS4	LDB3_MOUSE	LIM domain-binding protein 3 OS=Mus musculus GN=Ldb3 PE=1 SV=1 [MASS=76431]	16	13	1	1.15E+07	1.33E+07	1.09E+07	1.22E+07	1.22E+07	1.20E+07	1.30E+07	1.22E+07	1.34E+07	1.33E+07	1.48E+07	1.38E+07	1.51E+07	0	0	0	0	0	0	0	0	0	0	0	0	0	2, 2, 4, 2, 5, 3, 5, 3, 6, 4, 4, 5, 5, 5, 6, 6, 7, 8	1, 1, 13, 10, 10, 12, 13, 2, 2, 3, 3, 8, 4, 10, 4, 7, 8, 13	1, 1, 13, 10, 10, 12, 10, 4, 13, 19, 8, 2, 2, 3, 3, 8, 4, 7	LDB3_MOUSE, SP:Q9JKS4	SP|Q9JKS4|LDB3_MOUSE
Q9R112	SQRD_MOUSE	Sulfide:quinone oxidoreductase, mitochondrial OS=Mus musculus GN=Sqrdl PE=2 SV=2 [MASS=50340]	3	8	1	4.77E+04	4.30E+04	5.21E+04	4.62E+04	8.41E+04	6.67E+04	1.08E+05	4.03E+04	7.70E+04	5.81E+04	6.14E+04	5.42E+04	3.98E+04	0.086	0.07	0.011	0.036	0.018	0.028	0.003	0.053	0.015	0.016	0.005	0.073	0.043	2, 3	2, 8	2, 8	SQRD_MOUSE, SP:Q9R112	SP|Q9R112|SQRD_MOUSE
P10107	ANXA1_MOUSE	Annexin A1 OS=Mus musculus GN=Anxa1 PE=1 SV=2 [MASS=38734]	2	5	1	3.11E+04	2.29E+04	2.35E+04	2.49E+04	1.91E+04	2.18E+04	2.23E+04	4.27E+04	4.72E+04	2.77E+04	2.44E+04	2.08E+04	4.42E+04	0.134	0.14	0.064	0.093	0.159	0.137	0.104	0.048	0.045	0.068	0.054	0.184	0.035	2	2, 5	2, 5	ANXA1_MOUSE, SP:P10107	SP|P10107|ANXA1_MOUSE
Q6PHZ2	KCC2D_MOUSE	Calcium/calmodulin-dependent protein kinase type II delta chain OS=Mus musculus GN=Camk2d PE=1 SV=1 [MASS=56369]	3	12	1	2.28E+05	1.56E+05	1.38E+05	1.48E+05	1.58E+05	1.57E+05	1.54E+05	1.88E+05	1.86E+05	1.94E+05	1.92E+05	1.86E+05	1.90E+05	0.001	0.002	1.08E-04	6.63E-04	0.002	0.002	4.99E-04	2.52E-04	3.93E-04	4.40E-05	3.46E-06	0.004	8.88E-05	2, 3, 4	12, 6	12, 6	KCC2D_MOUSE, SP:Q6PHZ2	SP|Q6PHZ2|KCC2D_MOUSE
Q9D7B6	ACAD8_MOUSE	Isobutyryl-CoA dehydrogenase, mitochondrial OS=Mus musculus GN=Acad8 PE=2 SV=2 [MASS=45020]	4	13	1	2.37E+05	2.57E+05	1.96E+05	2.26E+05	2.03E+05	1.93E+05	2.29E+05	2.11E+05	1.43E+05	1.22E+05	1.79E+05	1.74E+05	1.82E+05	0.001	1.56E-04	5.44E-06	3.67E-05	5.78E-04	5.96E-04	3.01E-05	1.17E-04	0.002	9.52E-04	7.58E-06	0.005	1.24E-04	2, 3, 4	10, 13, 4	10, 13, 4	ACAD8_MOUSE, SP:Q9D7B6	SP|Q9D7B6|ACAD8_MOUSE
Q9QYJ0	DNJA2_MOUSE	DnaJ homolog subfamily A member 2 OS=Mus musculus GN=Dnaja2 PE=1 SV=1 [MASS=45745]	3	13	1	4.37E+05	4.24E+05	4.31E+05	4.87E+05	4.13E+05	4.00E+05	3.19E+05	4.98E+05	4.42E+05	3.99E+05	4.36E+05	5.02E+05	4.27E+05	1.83E-05	1.78E-06	3.37E-11	2.98E-09	1.75E-06	1.48E-06	1.04E-06	7.45E-09	1.16E-07	9.19E-09	6.81E-12	4.88E-06	1.00E-08	3, 4, 5, 5, 6	1, 2, 8, 13, 7	13, 3, 9, 7	DNJA2_MOUSE, SP:Q9QYJ0	SP|Q9QYJ0|DNJA2_MOUSE
Q91V41	RAB14_MOUSE	Ras-related protein Rab-14 OS=Mus musculus GN=Rab14 PE=1 SV=3 [MASS=23897]	2	12	1	1.09E+05	1.13E+05	9.40E+04	1.20E+05	9.33E+04	9.36E+04	1.06E+05	1.35E+05	1.17E+05	1.19E+05	1.33E+05	1.02E+05	1.12E+05	0.02	0.008	0.001	0.002	0.014	0.012	0.003	0.002	0.004	0.001	9.15E-05	0.023	0.002	3	1, 12	1, 12	RAB14_MOUSE, SP:Q91V41	SP|Q91V41|RAB14_MOUSE
Q5M8N4	D39U1_MOUSE	Epimerase family protein SDR39U1 OS=Mus musculus GN=Sdr39u1 PE=1 SV=1 [MASS=32995]	2	2	1	1.09E+05	1.36E+05	1.09E+05	1.17E+05	9.27E+04	1.09E+05	1.23E+05	1.09E+05	6.54E+04	9.28E+04	1.04E+05	1.03E+05	9.91E+04	0.019	0.004	4.98E-04	0.002	0.014	0.007	0.002	0.004	0.023	0.003	4.50E-04	0.022	0.003	2, 4	1, 2	1, 2	D39U1_MOUSE, SP:Q5M8N4	SP|Q5M8N4|D39U1_MOUSE
P35278	RAB5B_MOUSE	Ras-related protein Rab-5C OS=Mus musculus GN=Rab5c PE=1 SV=2 [MASS=23412]	1	5	0.97	5.09E+04	2.94E+04	2.86E+04	2.51E+04	3.54E+04	2.71E+04	3.19E+04	4.00E+04	2.93E+04	3.11E+04	3.33E+04	4.21E+04	3.87E+04	0.079	0.111	0.046	0.093	0.087	0.112	0.067	0.054	0.091	0.058	0.031	0.1	0.045	2	5	5	RAB5B_MOUSE, SP:P61021	SP|P61021|RAB5B_MOUSE,SP|Q9CQD1|RAB5A_MOUSE,SP|P35278|RAB5C_MOUSE
Q8VEK3	HNRPU_MOUSE	Heterogeneous nuclear ribonucleoprotein U OS=Mus musculus GN=Hnrnpu PE=1 SV=1 [MASS=87917]	4	13	1	3.42E+05	3.16E+05	2.76E+05	2.97E+05	2.90E+05	2.51E+05	2.51E+05	3.73E+05	3.17E+05	2.31E+05	2.58E+05	2.59E+05	2.62E+05	1.30E-04	3.19E-05	8.88E-08	2.78E-06	5.16E-05	1.08E-04	1.31E-05	4.81E-07	5.91E-06	9.42E-06	1.02E-07	7.40E-04	5.54E-06	2, 4, 5	1, 13, 3	1, 13, 3	HNRPU_MOUSE, SP:Q8VEK3	SP|Q8VEK3|HNRPU_MOUSE
P38060	HMGCL_MOUSE	Hydroxymethylglutaryl-CoA lyase, mitochondrial OS=Mus musculus GN=Hmgcl PE=1 SV=2 [MASS=34161]	3	13	1	4.68E+05	3.21E+05	2.98E+05	3.17E+05	3.26E+05	3.65E+05	3.09E+05	3.76E+05	2.81E+05	3.02E+05	3.46E+05	4.11E+05	3.00E+05	9.59E-06	2.74E-05	2.70E-08	1.32E-06	1.84E-05	4.07E-06	1.48E-06	4.41E-07	1.81E-05	4.90E-07	7.94E-10	3.13E-05	1.24E-06	3, 6	1, 6, 13, 6	1, 6, 13, 6	HMGCL_MOUSE, SP:P38060	SP|P38060|HMGCL_MOUSE
P09405	NUCL_MOUSE	Nucleolin OS=Mus musculus GN=Ncl PE=1 SV=2 [MASS=76723]	4	11	1	1.83E+05	1.81E+05	1.47E+05	1.31E+05	1.47E+05	1.39E+05	1.23E+05	1.86E+05	1.45E+05	1.32E+05	1.38E+05	1.06E+05	1.59E+05	0.004	0.001	6.49E-05	0.001	0.003	0.003	0.002	2.62E-04	0.001	6.03E-04	6.61E-05	0.021	3.02E-04	3, 4	1, 11, 6	1, 11, 6	NUCL_MOUSE, SP:P09405	SP|P09405|NUCL_MOUSE
O08600	NUCG_MOUSE	Endonuclease G, mitochondrial OS=Mus musculus GN=Endog PE=2 SV=1 [MASS=32190]	2	1	1	1.68E+05	1.64E+05	1.52E+05	1.76E+05	1.43E+05	1.10E+05	1.35E+05	1.64E+05	1.33E+05	1.44E+05	1.72E+05	1.25E+05	1.29E+05	0.005	0.002	5.06E-05	2.24E-04	0.003	0.007	9.94E-04	5.78E-04	0.002	3.58E-04	1.06E-05	0.014	0.001	3, 5	1	1	NUCG_MOUSE, SP:O08600	SP|O08600|NUCG_MOUSE
P48678	LMNA_MOUSE	Lamin-A/C OS=Mus musculus GN=Lmna PE=1 SV=2 [MASS=74237]	10	13	1	1.31E+06	1.40E+06	1.07E+06	1.04E+06	1.23E+06	1.24E+06	1.16E+06	1.27E+06	1.18E+06	1.44E+06	1.19E+06	1.24E+06	1.42E+06	4.55E-13	1.89E-17	5.25E-25	7.48E-18	7.06E-16	9.73E-17	9.07E-20	8.02E-20	1.39E-17	8.16E-27	2.12E-29	1.71E-12	6.23E-25	2, 3, 3, 5, 4, 5	1, 1, 12, 13, 2, 2, 12, 3, 6, 5	1, 1, 12, 12, 2, 13, 2, 3, 6, 5	LMNA_MOUSE, SP:P48678	SP|P48678|LMNA_MOUSE
P28650	PURA1_MOUSE	Adenylosuccinate synthetase isozyme 1 OS=Mus musculus GN=Adssl1 PE=1 SV=2 [MASS=50254]	4	13	1	2.85E+05	2.45E+05	2.54E+05	2.50E+05	2.66E+05	2.53E+05	2.64E+05	2.11E+05	1.66E+05	1.97E+05	2.14E+05	2.07E+05	2.60E+05	4.24E-04	2.10E-04	2.76E-07	1.49E-05	9.83E-05	1.00E-04	7.77E-06	1.12E-04	7.64E-04	3.89E-05	1.06E-06	0.002	5.80E-06	3, 5	1, 13, 3, 9	1, 13, 3, 9	PURA1_MOUSE, SP:P28650	SP|P28650|PURA1_MOUSE
P63242	IF5A1_MOUSE	Eukaryotic translation initiation factor 5A-1 OS=Mus musculus GN=Eif5a PE=1 SV=2 [MASS=16832]	1	4	1	8.43E+03	1.63E+04	8.46E+04	1.35E+04	8.02E+04	7.75E+04	3.97E+04	1.94E+04	1.25E+05	1.26E+05	4.49E+04	4.67E+04	2.93E+04	0.283	0.181	0.002	0.165	0.02	0.02	0.047	0.132	0.003	7.69E-04	0.015	0.088	0.07	5	4	4	IF5A1_MOUSE, SP:P63242	SP|P63242|IF5A1_MOUSE
Q924D0	RT4I1_MOUSE	Reticulon-4-interacting protein 1, mitochondrial OS=Mus musculus GN=Rtn4ip1 PE=1 SV=2 [MASS=43371]	4	13	1	5.25E+05	5.30E+05	4.55E+05	6.22E+05	4.85E+05	5.01E+05	4.50E+05	4.51E+05	3.78E+05	4.09E+05	5.49E+05	3.75E+05	3.97E+05	2.95E-06	1.11E-07	1.14E-11	2.51E-11	2.53E-07	8.46E-08	8.96E-09	3.60E-08	8.56E-07	6.29E-09	1.82E-14	6.53E-05	3.09E-08	2, 5	13, 4, 8	13, 4, 8	RT4I1_MOUSE, SP:Q924D0	SP|Q924D0|RT4I1_MOUSE
P60335	PCBP1_MOUSE	Poly(rC)-binding protein 1 OS=Mus musculus GN=Pcbp1 PE=1 SV=1 [MASS=37498]	3	3	1	6.19E+04	5.59E+04	8.70E+04	5.04E+04	1.40E+05	1.05E+05	1.05E+05	6.66E+04	1.88E+05	1.89E+05	1.40E+05	1.31E+05	7.60E+04	0.06	0.047	0.002	0.031	0.003	0.008	0.003	0.019	3.69E-04	5.34E-05	6.52E-05	0.012	0.009	3, 4, 5	1, 2, 3	1, 2, 3	PCBP1_MOUSE, SP:P60335	SP|P60335|PCBP1_MOUSE
P62071	RRAS2_MOUSE	Ras-related protein R-Ras2 OS=Mus musculus GN=Rras2 PE=1 SV=1 [MASS=23399]	2	13	1	9.08E+04	9.10E+04	7.70E+04	8.00E+04	7.44E+04	8.78E+04	6.61E+04	9.10E+04	1.05E+05	1.11E+05	1.17E+05	8.72E+04	1.22E+05	0.03	0.016	0.003	0.009	0.024	0.014	0.015	0.008	0.006	0.001	2.14E-04	0.032	0.001	3, 4	13, 2	13, 2	RRAS2_MOUSE, SP:P62071	SP|P62071|RRAS2_MOUSE
Q3V384	LACE1_MOUSE	Lactation elevated protein 1 OS=Mus musculus GN=Lace1 PE=2 SV=1 [MASS=54313]	3	12	1	9.37E+04	9.94E+04	1.10E+05	1.06E+05	7.79E+04	8.79E+04	9.73E+04	9.67E+04	7.97E+04	7.54E+04	1.02E+05	8.35E+04	7.98E+04	0.028	0.012	4.75E-04	0.003	0.022	0.014	0.004	0.006	0.014	0.007	5.45E-04	0.036	0.008	2, 3	12, 4, 8	12, 4, 8	LACE1_MOUSE, SP:Q3V384	SP|Q3V384|LACE1_MOUSE
Q921H8	THIKA_MOUSE	3-ketoacyl-CoA thiolase A, peroxisomal OS=Mus musculus GN=Acaa1a PE=2 SV=1 [MASS=43953]	2	12	1	3.25E+05	3.26E+05	2.54E+05	2.24E+05	2.30E+05	2.34E+05	1.76E+05	2.66E+05	2.28E+05	1.65E+05	1.45E+05	2.17E+05	1.79E+05	1.80E-04	2.38E-05	2.61E-07	3.79E-05	2.66E-04	1.78E-04	2.07E-04	1.81E-05	9.99E-05	1.50E-04	4.89E-05	0.002	1.34E-04	5, 6	12, 5	12, 5	THIKA_MOUSE, SP:Q921H8	SP|Q921H8|THIKA_MOUSE
Q9D6S7	RRFM_MOUSE	Ribosome-recycling factor, mitochondrial OS=Mus musculus GN=Mrrf PE=1 SV=1 [MASS=29050]	2	4	1	2.70E+05	2.51E+05	1.66E+05	1.96E+05	1.98E+05	1.92E+05	1.95E+05	1.79E+05	1.63E+05	1.64E+05	1.67E+05	1.58E+05	1.96E+05	5.84E-04	1.87E-04	2.50E-05	1.15E-04	6.56E-04	6.07E-04	1.05E-04	3.60E-04	8.35E-04	1.55E-04	1.43E-05	0.007	7.92E-05	3, 5	2, 3, 4	2, 3, 4	RRFM_MOUSE, SP:Q9D6S7	SP|Q9D6S7|RRFM_MOUSE
Q9WVA4	TAGL2_MOUSE	Transgelin-2 OS=Mus musculus GN=Tagln2 PE=1 SV=4 [MASS=22395]	3	13	1	4.27E+05	4.89E+05	3.91E+05	4.57E+05	4.09E+05	3.20E+05	3.70E+05	7.59E+05	6.31E+05	5.91E+05	5.37E+05	5.13E+05	6.34E+05	2.18E-05	3.24E-07	8.85E-10	9.85E-09	1.96E-06	1.47E-05	2.26E-07	1.54E-12	3.35E-10	4.68E-12	3.32E-14	3.91E-06	5.10E-12	4	10, 12, 13, 6	10, 12, 13, 6	TAGL2_MOUSE, SP:Q9WVA4	SP|Q9WVA4|TAGL2_MOUSE
Q8BXV2	BRI3B_MOUSE	BRI3-binding protein OS=Mus musculus GN=Bri3bp PE=2 SV=1 [MASS=28263]	2	10	1	9.75E+04	9.34E+04	7.33E+04	8.76E+04	7.58E+04	9.80E+04	1.02E+05	8.51E+04	8.14E+04	1.08E+05	8.79E+04	8.77E+04	1.12E+05	0.025	0.015	0.004	0.007	0.023	0.01	0.004	0.01	0.013	0.002	0.001	0.032	0.002	3, 3, 4	1, 2, 10	1, 2, 10	BRI3B_MOUSE, SP:Q8BXV2	SP|Q8BXV2|BRI3B_MOUSE
P56395	CYB5_MOUSE	Cytochrome b5 OS=Mus musculus GN=Cyb5a PE=1 SV=2 [MASS=15241]	2	10	1	1.85E+05	1.87E+05	1.01E+05	1.08E+05	1.51E+05	1.43E+05	1.15E+05	1.35E+05	1.29E+05	1.44E+05	1.52E+05	1.64E+05	1.58E+05	0.004	0.001	7.46E-04	0.003	0.002	0.003	0.002	0.002	0.003	3.64E-04	3.19E-05	0.006	3.16E-04	3, 4	1, 10	1, 10	CYB5_MOUSE, SP:P56395	SP|P56395|CYB5_MOUSE
O09117	SYPL1_MOUSE	Synaptophysin-like protein 1 OS=Mus musculus GN=Sypl1 PE=1 SV=2 [MASS=28899]	1	13	0.99	1.09E+05	1.03E+05	3.72E+04	5.56E+04	8.07E+04	7.02E+04	5.68E+04	6.91E+04	8.51E+04	7.60E+04	6.52E+04	9.32E+04	6.86E+04	0.019	0.011	0.027	0.024	0.02	0.025	0.022	0.017	0.011	0.007	0.004	0.028	0.012	3	13	13	SYPL1_MOUSE, SP:O09117	SP|O09117|SYPL1_MOUSE
Q9WTP6	KAD2_MOUSE	Adenylate kinase 2, mitochondrial OS=Mus musculus GN=Ak2 PE=1 SV=5 [MASS=26469]	2	13	1	2.17E+05	2.12E+05	1.36E+05	1.65E+05	1.73E+05	1.74E+05	1.82E+05	1.76E+05	1.51E+05	1.62E+05	1.58E+05	1.57E+05	1.40E+05	0.002	5.24E-04	1.20E-04	3.45E-04	0.001	0.001	1.69E-04	3.80E-04	0.001	1.70E-04	2.30E-05	0.007	6.48E-04	2, 3	13, 2	13, 2	KAD2_MOUSE, SP:Q9WTP6	SP|Q9WTP6|KAD2_MOUSE
Q02788	CO6A2_MOUSE	Collagen alpha-2(VI) chain OS=Mus musculus GN=Col6a2 PE=2 SV=3 [MASS=110334]	6	13	1	6.74E+05	8.05E+05	7.47E+05	8.02E+05	7.91E+05	7.89E+05	8.98E+05	7.30E+05	8.10E+05	7.32E+05	9.17E+05	8.08E+05	1.15E+06	1.45E-07	8.65E-11	5.02E-18	4.22E-14	7.22E-11	2.84E-11	1.14E-15	3.87E-12	1.32E-12	1.52E-14	6.35E-23	1.01E-08	2.09E-20	3, 4, 5	10, 11, 12, 13, 3	10, 11, 12, 13, 3	CO6A2_MOUSE, SP:Q02788	SP|Q02788|CO6A2_MOUSE
Q8VCM7	FIBG_MOUSE	Fibrinogen gamma chain OS=Mus musculus GN=Fgg PE=2 SV=1 [MASS=49391]	7	13	1	5.00E+05	1.14E+06	7.39E+05	9.00E+05	6.84E+05	8.21E+05	8.02E+05	1.11E+06	4.69E+05	7.45E+05	5.63E+05	5.38E+05	8.32E+05	5.11E-06	1.67E-14	9.13E-18	1.46E-15	1.40E-09	1.36E-11	3.48E-14	2.33E-17	5.71E-08	1.37E-14	1.23E-14	2.45E-06	3.07E-15	2, 3, 4	1, 13, 2, 5, 7	1, 13, 2, 5, 7	FIBG_MOUSE, SP:Q8VCM7	SP|Q8VCM7|FIBG_MOUSE
P97823	LYPA1_MOUSE	Acyl-protein thioesterase 1 OS=Mus musculus GN=Lypla1 PE=1 SV=1 [MASS=24688]	4	13	1	3.76E+05	3.07E+05	3.32E+05	2.97E+05	3.99E+05	3.69E+05	3.78E+05	3.01E+05	3.44E+05	3.79E+05	3.59E+05	3.85E+05	2.98E+05	6.55E-05	4.10E-05	5.42E-09	2.85E-06	2.58E-06	3.62E-06	1.24E-07	5.48E-06	2.54E-06	2.14E-08	4.00E-10	5.38E-05	1.38E-06	3, 4, 5	1, 10, 13, 6	1, 10, 13, 6	LYPA1_MOUSE, SP:P97823	SP|P97823|LYPA1_MOUSE
Q62351	TFR1_MOUSE	Transferrin receptor protein 1 OS=Mus musculus GN=Tfrc PE=1 SV=1 [MASS=85731]	5	8	1	1.28E+05	8.94E+04	5.15E+04	6.89E+04	4.98E+04	4.74E+04	5.16E+04	1.44E+05	1.22E+05	1.21E+05	7.84E+04	6.12E+04	5.24E+04	0.013	0.017	0.012	0.014	0.054	0.054	0.028	0.001	0.003	9.52E-04	0.002	0.061	0.025	2, 3	1, 5, 6, 8	1, 5, 6, 8	TFR1_MOUSE, SP:Q62351	SP|Q62351|TFR1_MOUSE
Q59J78	MIMIT_MOUSE	Mimitin, mitochondrial OS=Mus musculus GN=Ndufaf2 PE=2 SV=1 [MASS=19628]	1	8	0.99	4.39E+04	3.41E+04	2.08E+04	2.64E+04	3.54E+04	2.97E+04	3.30E+04	3.13E+04	2.65E+04	2.91E+04	3.76E+04	4.06E+04	2.92E+04	0.095	0.094	0.075	0.086	0.087	0.101	0.063	0.081	0.102	0.064	0.023	0.104	0.07	3	8	8	MIMIT_MOUSE, SP:Q59J78	SP|Q59J78|MIMIT_MOUSE
Q8R1I1	QCR9_MOUSE	Cytochrome b-c1 complex subunit 9 OS=Mus musculus GN=Uqcr10 PE=1 SV=1 [MASS=7445]	2	13	1	4.04E+06	4.50E+06	4.28E+06	4.15E+06	4.27E+06	3.91E+06	4.31E+06	3.98E+06	3.41E+06	3.16E+06	3.04E+06	3.18E+06	3.35E+06	7.68E-34	0	0	0	0	4.20E-44	0	0	4.06E-43	0	0	1.60E-27	0	4, 4, 5	12, 8, 13	12, 13, 8	QCR9_MOUSE, SP:Q8R1I1	SP|Q8R1I1|QCR9_MOUSE
P17665	COX7C_MOUSE	Cytochrome c oxidase subunit 7C, mitochondrial OS=Mus musculus GN=Cox7c PE=1 SV=1 [MASS=7333]	1	13	0.99	8.52E+05	1.10E+06	1.39E+06	1.09E+06	1.03E+06	1.17E+06	1.48E+06	1.03E+06	8.75E+05	9.35E+05	8.88E+05	7.07E+05	8.80E+05	6.76E-09	1.68E-13	2.17E-28	2.09E-17	5.07E-13	4.87E-15	1.32E-22	1.53E-15	6.07E-13	5.08E-17	1.19E-20	1.08E-07	2.55E-15	4	13	13	COX7C_MOUSE, SP:P17665	SP|P17665|COX7C_MOUSE
P03930	ATP8_MOUSE	ATP synthase protein 8 OS=Mus musculus GN=Mtatp8 PE=1 SV=1 [MASS=7766]	3	13	1	5.97E+06	5.70E+06	6.99E+06	5.82E+06	6.22E+06	6.02E+06	7.69E+06	5.57E+06	4.45E+06	4.78E+06	4.70E+06	5.06E+06	4.81E+06	0	0	0	0	0	0	0	0	0	0	0	1.72E-40	0	3, 3, 5, 4	11, 4, 7, 11, 9	11, 15, 7, 9	ATP8_MOUSE, SP:P03930	SP|P03930|ATP8_MOUSE
P56392	CX7A1_MOUSE	Cytochrome c oxidase polypeptide 7A1, mitochondrial OS=Mus musculus GN=Cox7a1 PE=2 SV=1 [MASS=8986]	2	13	1	6.74E+06	7.85E+06	9.11E+06	8.94E+06	6.15E+06	7.21E+06	9.71E+06	8.79E+06	6.48E+06	6.57E+06	6.95E+06	6.03E+06	5.89E+06	0	0	0	0	0	0	0	0	0	0	0	0	0	4, 7, 5	13	13	CX7A1_MOUSE, SP:P56392	SP|P56392|CX7A1_MOUSE
P15116	CADH2_MOUSE	Cadherin-2 OS=Mus musculus GN=Cdh2 PE=1 SV=1 [MASS=99761]	7	13	1	9.87E+05	8.90E+05	7.65E+05	9.06E+05	8.27E+05	9.14E+05	8.58E+05	8.74E+05	9.99E+05	9.41E+05	1.12E+06	1.06E+06	1.20E+06	2.67E-10	9.51E-12	2.06E-18	1.10E-15	2.67E-11	8.49E-13	4.02E-15	3.42E-14	3.92E-15	3.31E-18	1.31E-27	6.71E-11	3.12E-21	2, 3, 4, 5, 6	1, 11, 13, 6, 8	1, 11, 13, 6, 8	CADH2_MOUSE, SP:P15116	SP|P15116|CADH2_MOUSE
P00158	CYB_MOUSE	Cytochrome b OS=Mus musculus GN=Mt-Cyb PE=2 SV=1 [MASS=43209]	1	13	0.99	1.76E+05	4.65E+05	5.96E+05	4.45E+05	5.13E+05	5.28E+05	6.97E+05	4.88E+05	4.44E+05	6.69E+05	6.97E+05	2.82E+05	7.54E+05	0.004	7.51E-07	3.35E-14	1.85E-08	1.52E-07	5.52E-08	3.25E-12	1.57E-08	1.47E-07	5.37E-13	5.82E-17	4.75E-04	1.55E-13	4	13	13	CYB_MOUSE, SP:P00158	SP|P00158|CYB_MOUSE
Q8R0N6	HOT_MOUSE	Hydroxyacid-oxoacid transhydrogenase, mitochondrial OS=Mus musculus GN=Adhfe1 PE=2 SV=2 [MASS=49937]	2	2	1	8.83E+04	8.31E+04	9.08E+04	1.04E+05	6.89E+04	5.82E+04	7.03E+04	8.38E+04	4.92E+04	4.53E+04	7.01E+04	4.94E+04	4.49E+04	0.034	0.021	0.002	0.004	0.032	0.04	0.014	0.011	0.045	0.032	0.004	0.085	0.037	3, 4	1, 2	1, 2	HOT_MOUSE, SP:Q8R0N6	SP|Q8R0N6|HOT_MOUSE
P35279	RAB6A_MOUSE	Ras-related protein Rab-6A OS=Mus musculus GN=Rab6a PE=1 SV=4 [MASS=23590]	1	1	0.93	1.39E+04	1.22E+04	1.71E+04	1.70E+04	1.35E+04	1.56E+04	1.14E+04	1.61E+04	1.45E+04	2.16E+04	2.00E+04	1.57E+04	2.52E+04	0.231	0.217	0.097	0.139	0.202	0.179	0.187	0.155	0.175	0.096	0.075	0.218	0.087	3	1	1	RAB6A_MOUSE, SP:P35279	SP|P35279|RAB6A_MOUSE
Q9CQ40	RM49_MOUSE	39S ribosomal protein L49, mitochondrial OS=Mus musculus GN=Mrpl49 PE=2 SV=1 [MASS=19133]	1	1	0.99	1.65E+04	6.10E+03	1.29E+04	2.02E+04	1.20E+04	8.23E+03	1.34E+04	2.06E+04	1.56E+04	1.21E+04	2.74E+04	1.39E+04	1.47E+04	0.211	0.291	0.13	0.118	0.215	0.253	0.165	0.125	0.166	0.164	0.045	0.23	0.148	2	1	1	RM49_MOUSE, SP:Q9CQ40	SP|Q9CQ40|RM49_MOUSE
P27659	RL3_MOUSE	60S ribosomal protein L3 OS=Mus musculus GN=Rpl3 PE=2 SV=2 [MASS=46124]	2	2	1	8.97E+04	6.26E+04	5.23E+04	6.39E+04	4.77E+04	5.88E+04	4.94E+04	1.16E+05	9.52E+04	8.02E+04	9.30E+04	8.57E+04	7.03E+04	0.03	0.038	0.012	0.018	0.058	0.037	0.031	0.003	0.008	0.006	9.03E-04	0.034	0.011	2, 5	1, 2	1, 2	RL3_MOUSE, SP:P27659	SP|P27659|RL3_MOUSE
Q9CPX8	QCR10_MOUSE	Cytochrome b-c1 complex subunit 10 OS=Mus musculus GN=Uqcr PE=2 SV=1 [MASS=6539]	1	13	0.99	2.57E+05	1.27E+05	2.46E+05	2.35E+05	1.44E+05	7.96E+04	1.33E+05	1.16E+05	7.96E+04	8.80E+04	1.30E+05	1.13E+05	9.68E+04	8.02E-04	0.006	4.95E-07	3.07E-05	0.003	0.019	0.001	0.003	0.014	0.004	1.19E-04	0.018	0.004	4	13	13	QCR10_MOUSE, SP:Q9CPX8	SP|Q9CPX8|QCR10_MOUSE
P61979	HNRPK_MOUSE	Heterogeneous nuclear ribonucleoprotein K OS=Mus musculus GN=Hnrnpk PE=1 SV=1 [MASS=50976]	8	13	1	1.10E+06	9.74E+05	9.60E+05	9.10E+05	7.90E+05	7.58E+05	6.96E+05	9.31E+05	8.69E+05	8.72E+05	8.70E+05	8.21E+05	8.37E+05	3.08E-11	1.07E-12	1.29E-22	8.83E-16	6.91E-11	6.22E-11	1.21E-12	4.99E-15	2.03E-13	4.85E-17	6.31E-22	7.64E-09	2.05E-15	3, 4, 5, 7	1, 12, 13, 4	1, 12, 13, 4	HNRPK_MOUSE, SP:P61979	SP|P61979|HNRPK_MOUSE
P62245	RS15A_MOUSE	40S ribosomal protein S15a OS=Mus musculus GN=Rps15a PE=2 SV=2 [MASS=14839]	2	4	1	5.28E+04	6.79E+04	1.17E+05	8.32E+04	6.31E+04	6.48E+04	1.07E+05	1.28E+05	9.90E+04	1.16E+05	7.38E+04	5.71E+04	9.01E+04	0.075	0.032	3.37E-04	0.008	0.035	0.03	0.003	0.002	0.007	0.001	0.003	0.068	0.005	2, 3	1, 4	1, 4	RS15A_MOUSE, SP:P62245	SP|P62245|RS15A_MOUSE

Q9D1X0	NOL3_MOUSE	Nucleolar protein 3 OS=Mus musculus GN=Nol3 PE=1 SV=1 [MASS=24567]	2	3	1	[illegible]
Q9CQF4	CF203_MOUSE	Uncharacterized protein C6orf203 homolog OS=Mus musculus PE=1 SV=1 [MASS=27847]	1	4	1	[illegible]
P10630	IF4A2_MOUSE	Eukaryotic initiation factor 4A-II OS=Mus musculus GN=Eif4a2 PE=2 SV=2 [MASS=46402]	1	7	0.99	[illegible]
P14211	CALR_MOUSE	Calreticulin OS=Mus musculus GN=Calr PE=1 SV=1 [MASS=47994]	5	11	1	[illegible]
Q61102	ABCB7_MOUSE	ATP-binding cassette sub-family B member 7, mitochondrial OS=Mus musculus GN=Abcb7 PE=1 SV=2 [MASS=82594]	1	4	0.99	[illegible]
P10493	NID1_MOUSE	Nidogen-1 OS=Mus musculus GN=Nid1 PE=1 SV=1 [MASS=136622]	10	13	1	[illegible]
Q9D517	PLCC_MOUSE	1-acyl-sn-glycerol-3-phosphate acyltransferase gamma OS=Mus musculus GN=Agpat3 PE=1 SV=2 [MASS=43296]	1	8	0.99	[illegible]
Q921I1	TRFE_MOUSE	Serotransferrin OS=Mus musculus GN=Tf PE=1 SV=1 [MASS=76723]	14	13	1	[illegible]
Q61292	LAMB2_MOUSE	Laminin subunit beta-2 OS=Mus musculus GN=Lamb2 PE=2 SV=1 [MASS=196352]	7	13	1	[illegible]
Q9DB73	NB5R1_MOUSE	NADH-cytochrome b5 reductase 1 OS=Mus musculus GN=Cyb5r1 PE=2 SV=1 [MASS=34135]	1	1	0.99	[illegible]
Q9CZ44	NSF1C_MOUSE	NSFL1 cofactor p47 OS=Mus musculus GN=Nsfl1c PE=1 SV=1 [MASS=40710]	2	6	1	[illegible]
Q9CQV1	TIM16_MOUSE	Mitochondrial import inner membrane translocase subunit Tim16 OS=Mus musculus GN=Timm16 PE=2 SV=1 [MASS=13785]	1	8	0.99	[illegible]
Q9JIW9	RALA_MOUSE	Ras-related protein Ral-B OS=Mus musculus GN=Ralb PE=2 SV=1 [MASS=23349]	1	1	0.84	[illegible]
O88338	CAD16_MOUSE	Cadherin-16 OS=Mus musculus GN=Cdh16 PE=2 SV=1 [MASS=89860]	1	11	0.69	[illegible]
Q9EQU5	SET_MOUSE	Protein SET OS=Mus musculus GN=Set PE=1 SV=1 [MASS=33378]	1	4	0.99	[illegible]
P59999	ARPC4_MOUSE	Actin-related protein 2/3 complex subunit 4 OS=Mus musculus GN=Arpc4 PE=1 SV=3 [MASS=19667]	2	5	1	[illegible]
Q80W04	TMCC2_MOUSE	Transmembrane and coiled-coil domains protein 2 OS=Mus musculus GN=Tmcc2 PE=1 SV=1 [MASS=77054]	1	3	0.9	[illegible]
Q63918	SDPR_MOUSE	Serum deprivation-response protein OS=Mus musculus GN=Sdpr PE=1 SV=3 [MASS=46764]	6	13	1	[illegible]
Q9D0S9	HINT2_MOUSE	Histidine triad nucleotide-binding protein 2 OS=Mus musculus GN=Hint2 PE=2 SV=1 [MASS=17320]	3	13	1	[illegible]
Q9JJI8	RL38_MOUSE	60S ribosomal protein L38 OS=Mus musculus GN=Rpl38 PE=2 SV=3 [MASS=8204]	2	6	0.99	[illegible]
P62889	RL30_MOUSE	60S ribosomal protein L30 OS=Mus musculus GN=Rpl30 PE=2 SV=2 [MASS=12784]	2	3	1	[illegible]
P29758	OAT_MOUSE	Ornithine aminotransferase, mitochondrial OS=Mus musculus GN=Oat PE=1 SV=1 [MASS=48354]	5	13	1	[illegible]
Q9DC61	MPPA_MOUSE	Mitochondrial-processing peptidase subunit alpha OS=Mus musculus GN=Pmpca PE=1 SV=1 [MASS=58279]	2	1	1	[illegible]
P01867	IGG2B_MOUSE	Ig gamma-2B chain C region OS=Mus musculus GN=Igh-3 PE=1 SV=3 [MASS=44259]	1	3	1	[illegible]
P23927	CRYAB_MOUSE	Alpha-crystallin B chain OS=Mus musculus GN=Cryab PE=1 SV=2 [MASS=20069]	7	13	1	[illegible]
Q05793	PGBM_MOUSE	Basement membrane-specific heparan sulfate proteoglycan core protein OS=Mus musculus GN=Hspg2 PE=1 SV=1 [MASS=398292]	25	13	1	[illegible]
Q9JJH7	TRPM5_MOUSE	Transient receptor potential cation channel subfamily M member 5 OS=Mus musculus GN=Trpm5 PE=1 SV=1 [MASS=130843]	1	11	0.83	[illegible]
P09055	ITB1_MOUSE	Integrin beta-1 OS=Mus musculus GN=Itgb1 PE=1 SV=1 [MASS=88231]	5	10	1	[illegible]
Q9EQH3	VPS35_MOUSE	Vacuolar protein sorting-associated protein 35 OS=Mus musculus GN=Vps35 PE=1 SV=1 [MASS=91712]	3	9	1	[illegible]
Q61335	BAP31_MOUSE	B-cell receptor-associated protein 31 OS=Mus musculus GN=Bcap31 PE=1 SV=3 [MASS=27922]	1	7	0.99	[illegible]
P10126	EF1A1_MOUSE	Elongation factor 1-alpha 1 OS=Mus musculus GN=Eef1a1 PE=1 SV=3 [MASS=50113]	4	13	1	[illegible]
P50580	PA2G4_MOUSE	Proliferation-associated protein 2G4 OS=Mus musculus GN=Pa2g4 PE=1 SV=3 [MASS=43699]	1	9	0.99	[illegible]
O88569	ROA2_MOUSE	Heterogeneous nuclear ribonucleoproteins A2/B1 OS=Mus musculus GN=Hnrnpa2b1 PE=1 SV=2 [MASS=37402]	6	13	1	[illegible]
P47754	CAZA2_MOUSE	F-actin-capping protein subunit alpha-2 OS=Mus musculus GN=Capza2 PE=1 SV=3 [MASS=32967]	3	13	1	[illegible]
Q8BWF0	SSDH_MOUSE	Succinate-semialdehyde dehydrogenase, mitochondrial OS=Mus musculus GN=Aldh5a1 PE=1 SV=1 [MASS=55968]	6	11	1	[illegible]
Q9JMA1	UBP14_MOUSE	Ubiquitin carboxyl-terminal hydrolase 14 OS=Mus musculus GN=Usp14 PE=1 SV=3 [MASS=56002]	1	2	0.99	[illegible]
Q8CI94	PYGB_MOUSE	Glycogen phosphorylase, brain form OS=Mus musculus GN=Pygb PE=1 SV=3 [MASS=96729]	14	13	1	[illegible]
Q8R0F8	FAHD1_MOUSE	Fumarylacetoacetate hydrolase domain-containing protein 1 OS=Mus musculus GN=Fahd1 PE=2 SV=1 [MASS=25158]	4	7	1	[illegible]
O88587	COMT_MOUSE	Catechol O-methyltransferase OS=Mus musculus GN=Comt PE=1 SV=1 [MASS=29496]	5	11	1	[illegible]
Q3TC72	FAHD2_MOUSE	Fumarylacetoacetate hydrolase domain-containing protein 2A OS=Mus musculus GN=Fahd2 PE=1 SV=1 [MASS=34690]	1	2	0.99	[illegible]
Q99LX0	PARK7_MOUSE	Protein DJ-1 OS=Mus musculus GN=Park7 PE=1 SV=1 [MASS=20021]	8	13	1	[illegible]
Q64516	GLPK_MOUSE	Glycerol kinase OS=Mus musculus GN=Gk PE=2 SV=2 [MASS=61227]	1	4	1	[illegible]
A2ASS6	TITIN_MOUSE	Titin OS=Mus musculus GN=Ttn PE=1 SV=1	719	13		[illegible]
P57780	ACTN4_MOUSE	Alpha-actinin-4 OS=Mus musculus GN=Actn4 PE=1 SV=1	4	9	1	[illegible]
Q60668	HNRPD_MOUSE	Heterogeneous nuclear ribonucleoprotein D0 OS=Mus musculus GN=Hnrnpd PE=1 SV=2	1	3	1	[illegible]
Q3UTJ2	SRBS2_MOUSE	Sorbin and SH3 domain-containing protein 2 OS=Mus musculus GN=Sorbs2 PE=1 SV=2	7	13	1	[illegible]
Q91Z83	MYH7_MOUSE	Myosin-7 OS=Mus musculus GN=Myh7 PE=1 SV=1	20	13	1	[illegible]
Q8R081	HNRPL_MOUSE	Heterogeneous nuclear ribonucleoprotein L OS=Mus musculus GN=Hnrnpl PE=1 SV=2	2	3	1	[illegible]
Q9JJW5	MYOZ2_MOUSE	Myozenin-2 OS=Mus musculus GN=Myoz2 PE=1 SV=1	10	13	1	[illegible]
P46638	RB11B_MOUSE	Ras-related protein Rab-11B OS=Mus musculus GN=Rab11b PE=1 SV=3	1	1	1	[illegible]
Q93092	TALDO_MOUSE	Transaldolase OS=Mus musculus GN=Taldo1 PE=1 SV=2	3	12	1	[illegible]
Q8BGK2	ARHL1_MOUSE	[Protein ADP-ribosylarginine] hydrolase-like protein 1 OS=Mus musculus GN=Adprhl1 PE=2 SV=1	5	10	1	[illegible]
P26043	RADI_MOUSE	Radixin OS=Mus musculus GN=Rdx PE=1 SV=2	3	6	1	[illegible]
Q9DBC7	KAP0_MOUSE	cAMP-dependent protein kinase type I-alpha regulatory subunit OS=Mus musculus GN=Prkar1a PE=1 SV=3	4	8	1	[illegible]
Q99020	ROAA_MOUSE	Heterogeneous nuclear ribonucleoprotein A/B OS=Mus musculus GN=Hnrnpab PE=1 SV=1	4	13	1	[illegible]
P26883	FKB1A_MOUSE	Peptidyl-prolyl cis-trans isomerase FKBP1A OS=Mus musculus GN=Fkbp1a PE=2 SV=2	1	13	1	[illegible]
O08553	DPYL2_MOUSE	Dihydropyrimidinase-related protein 2 OS=Mus musculus GN=Dpysl2 PE=1 SV=2	5	13	1	[illegible]
O88844	IDHC_MOUSE	Isocitrate dehydrogenase [NADP] cytoplasmic OS=Mus musculus GN=Idh1 PE=1 SV=1	4	6	1	[illegible]
Q8BG05	ROA3_MOUSE	Heterogeneous nuclear ribonucleoprotein A3 OS=Mus musculus GN=Hnrnpa3 PE=1 SV=1	2	13	1	[illegible]
O08638	MYH11_MOUSE	Myosin-11 OS=Mus musculus GN=Myh11 PE=1 SV=1	4	6	1	[illegible]
O08709	PRDX6_MOUSE	Peroxiredoxin-6 OS=Mus musculus GN=Prdx6 PE=1 SV=3	8	13	1	[illegible]
Q9D0E1	HNRPM_MOUSE	Heterogeneous nuclear ribonucleoprotein M OS=Mus musculus GN=Hnrnpm PE=1 SV=3	5	12	1	[illegible]
P68254	1433T_MOUSE	14-3-3 protein theta OS=Mus musculus GN=Ywhaq PE=1 SV=1	1	13	1	[illegible]
Q60675	LAMA2_MOUSE	Laminin subunit alpha-2 OS=Mus musculus GN=Lama2 PE=1 SV=1	20	13	1	[illegible]
Q9CPU0	LGUL_MOUSE	Lactoylglutathione lyase OS=Mus musculus GN=Glo1 PE=1 SV=3	4	13	1	[illegible]
Q6IRU2	TPM4_MOUSE	Tropomyosin alpha-4 chain OS=Mus musculus GN=Tpm4 PE=2 SV=3	4	13	1	[illegible]
Q99PT1	GDIR1_MOUSE	Rho GDP-dissociation inhibitor 1 OS=Mus musculus GN=Arhgdia PE=1 SV=3	3	13	1	[illegible]
Q8VHX6	FLNC_MOUSE	Filamin-C OS=Mus musculus GN=Flnc PE=1 SV=3	35	13	1	[illegible]
Q8BG32	PSD11_MOUSE	26S proteasome non-ATPase regulatory subunit 11 OS=Mus musculus GN=Psmd11 PE=1 SV=3	2	6	1	[illegible]
P40142	TKT_MOUSE	Transketolase OS=Mus musculus GN=Tkt PE=1 SV=1	1	4	0.99	[illegible]
Q64727	VINC_MOUSE	Vinculin OS=Mus musculus GN=Vcl PE=1 SV=4	20	13	1	[illegible]
O08795	GLU2B_MOUSE	Glucosidase 2 subunit beta OS=Mus musculus GN=Prkcsh PE=1 SV=1	1	3	0.97	[illegible]
P17182	ENOA_MOUSE	Alpha-enolase OS=Mus musculus GN=Eno1 PE=1 SV=3	8	13	1	[illegible]
Q8BZF8	PGM5_MOUSE	Phosphoglucomutase-like protein 5 OS=Mus musculus GN=Pgm5 PE=1 SV=2	7	13	1	[illegible]
Q91X72	HEMO_MOUSE	Hemopexin OS=Mus musculus GN=Hpx PE=1 SV=1	7	13	1	[illegible]
P14733	LMNB1_MOUSE	Lamin-B1 OS=Mus musculus GN=Lmnb1 PE=1 SV=3	4	8	1	[illegible]
P50396	GDIA_MOUSE	Rab GDP dissociation inhibitor alpha OS=Mus musculus GN=Gdi1 PE=1 SV=3	2	5	1	[illegible]
Q7TPR4	ACTN1_MOUSE	Alpha-actinin-1 OS=Mus musculus GN=Actn1 PE=2 SV=1	2	3	1	[illegible]
P26231	CTNA1_MOUSE	Catenin alpha-1 OS=Mus musculus GN=Ctnna1 PE=1 SV=1	11	12	1	[illegible]
P28665	MUG1_MOUSE	Murinoglobulin-1 OS=Mus musculus GN=Mug1 PE=1 SV=3	5	12	1	[illegible]
O55131	SEPT7_MOUSE	Septin-7 OS=Mus musculus GN=Sept7 PE=1 SV=1	3	5	1	[illegible]
Q8CI51	PDLI5_MOUSE	PDZ and LIM domain protein 5 OS=Mus musculus GN=Pdlim5 PE=1 SV=3	9	13	1	[illegible]
Q7TMK9	HNRPQ_MOUSE	Heterogeneous nuclear ribonucleoprotein Q OS=Mus musculus GN=Syncrip PE=1 SV=2	3	4	1	[illegible]
Q9R0P5	DEST_MOUSE	Destrin OS=Mus musculus GN=Dstn PE=1 SV=3	2	1	1	[illegible]
P50446	K2C6A_MOUSE	Keratin, type II cytoskeletal 6A OS=Mus musculus GN=Krt6a PE=2 SV=3	1	1	1	[illegible]
Q6ZQ06	QN1_MOUSE	Protein QN1 homolog OS=Mus musculus GN=Kiaa1009 PE=1 SV=2	1	8	0.82	[illegible]
A2AAJ9	OBSCN_MOUSE	Obscurin OS=Mus musculus GN=Obscn PE=2 SV=2	22	13	1	[illegible]
P08556	RASN_MOUSE	GTPase NRas OS=Mus musculus GN=Nras PE=2 SV=1	1	1	0.88	[illegible]
O08529	CAN2_MOUSE	Calpain-2 catalytic subunit OS=Mus musculus GN=Capn2 PE=2 SV=4	3	9	1	[illegible]
Q8BTM8	FLNA_MOUSE	Filamin-A OS=Mus musculus GN=Flna PE=1 SV=4	8	10	1	[illegible]
P10518	HEM2_MOUSE	Delta-aminolevulinic acid dehydratase OS=Mus musculus GN=Alad PE=1 SV=1	1	4	0.98	[illegible]
Q99K51	PLST_MOUSE	Plastin-3 OS=Mus musculus GN=Pls3 PE=1 SV=3	1	1	1	[illegible]
P07309	TTHY_MOUSE	Transthyretin OS=Mus musculus GN=Ttr PE=1 SV=1	3	11	1	[illegible]
Q80XB4	NRAP_MOUSE	Nebulin-related-anchoring protein OS=Mus musculus GN=Nrap PE=1 SV=2	3	6	1	[illegible]
P16045	LEG1_MOUSE	Galectin-1 OS=Mus musculus GN=Lgals1 PE=1 SV=3	5	13	1	[illegible]
Q62234	MYOM1_MOUSE	Myomesin-1 OS=Mus musculus GN=Myom1 PE=1 SV=2	46	13	1	[illegible]
Q9DBP5	KCY_MOUSE	UMP-CMP kinase OS=Mus musculus GN=Cmpk1 PE=1 SV=1	3	9	1	[illegible]
Q62261	SPTB2_MOUSE	Spectrin beta chain, brain 1 OS=Mus musculus GN=Sptbn1 PE=1 SV=2	39	13	1	[illegible]
Q9WTI7	MYO1C_MOUSE	Myosin-Ic OS=Mus musculus GN=Myo1c PE=1 SV=2	1	5	0.99	[illegible]
P99026	PSB4_MOUSE	Proteasome subunit beta type-4 OS=Mus musculus GN=Psmb4 PE=1 SV=1	1	10	1	[illegible]
P42208	SEPT2_MOUSE	Septin-2 OS=Mus musculus GN=Sept2 PE=1 SV=2	3	4	1	[illegible]
Q61838	A2M_MOUSE	Alpha-2-macroglobulin OS=Mus musculus GN=A2m PE=1 SV=2	6	5	1	[illegible]
Q80X90	FLNB_MOUSE	Filamin-B OS=Mus musculus GN=Flnb PE=2 SV=2	1	2	1	[illegible]
Q6P069	SORCN_MOUSE	Sorcin OS=Mus musculus GN=Sri PE=2 SV=1	2	4	0.99	[illegible]
Q99JY9	ARP3_MOUSE	Actin-related protein 3 OS=Mus musculus GN=Actr3 PE=1 SV=3	2	3	1	[illegible]
Q9JLV1	BAG3_MOUSE	BAG family molecular chaperone regulator 3 OS=Mus musculus GN=Bag3 PE=1 SV=1	8	13	1	[illegible]
Q9D358	PPAC_MOUSE	Low molecular weight phosphotyrosine protein phosphatase OS=Mus musculus GN=Acp1 PE=2 SV=3	1	3	1	[illegible]
Q9JM76	ARPC3_MOUSE	Actin-related protein 2/3 complex subunit 3 OS=Mus musculus GN=Arpc3 PE=1 SV=3	1	10	0.99	[illegible]
Q9R062	GLYG_MOUSE	Glycogenin-1 OS=Mus musculus GN=Gyg1 PE=2 SV=3	2	11	1	[illegible]
P26039	TLN1_MOUSE	Talin-1 OS=Mus musculus GN=Tln1 PE=1 SV=1	12	13	1	[illegible]
P62962	PROF1_MOUSE	Profilin-1 OS=Mus musculus GN=Pfn1 PE=1 SV=2	6	13	1	[illegible]
P32921	SYWC_MOUSE	Tryptophanyl-tRNA synthetase, cytoplasmic OS=Mus musculus GN=Wars PE=1 SV=2	2	10	1	[illegible]
Q9D1D4	TMEDA_MOUSE	Transmembrane emp24 domain-containing protein 10 OS=Mus musculus GN=Tmed10 PE=2 SV=1	1	5	0.99	[illegible]
Q9EQK5	MVP_MOUSE	Major vault protein OS=Mus musculus GN=Mvp PE=2 SV=3	2	8	1	[illegible]
O55222	ILK_MOUSE	Integrin-linked protein kinase OS=Mus musculus GN=Ilk PE=1 SV=2	2	13	1	[illegible]
Q76MZ3	2AAA_MOUSE	Serine/threonine-protein phosphatase 2A 65 kDa regulatory subunit A alpha isoform OS=Mus musculus GN=Ppp2r1a PE=1 SV=3	7	12	1	[illegible]
Q9WVJ2	PSD13_MOUSE	26S proteasome non-ATPase regulatory subunit 13 OS=Mus musculus GN=Psmd13 PE=1 SV=1	2	5	1	[illegible]
Q8K4G5	ABLM1_MOUSE	Actin-binding LIM protein 1 OS=Mus musculus GN=Ablim1 PE=1 SV=1	5	13	1	[illegible]
P37804	TAGL_MOUSE	Transgelin OS=Mus musculus GN=Tagln PE=1 SV=3	6	13	1	[illegible]
P13020	GELS_MOUSE	Gelsolin OS=Mus musculus GN=Gsn PE=1 SV=3	2	4	1	[illegible]
P17563	SBP1_MOUSE	Selenium-binding protein 1 OS=Mus musculus GN=Selenbp1 PE=1 SV=2	4	13	1	[illegible]
Q01149	CO1A2_MOUSE	Collagen alpha-2(I) chain OS=Mus musculus GN=Col1a2 PE=2 SV=2	2	13	1	[illegible]
Q9R0P9	UCHL1_MOUSE	Ubiquitin carboxyl-terminal hydrolase isozyme L1 OS=Mus musculus GN=Uchl1 PE=1 SV=1	2	8	1	[illegible]
O70373	XIRP1_MOUSE	Xin actin-binding repeat-containing protein 1 OS=Mus musculus GN=Xirp1 PE=1 SV=2	10	13	1	[illegible]
P63024	VAMP3_MOUSE	Vesicle-associated membrane protein 3 OS=Mus musculus GN=Vamp3 PE=2 SV=1	1	2	1	[illegible]

P61971	NTF2_MOUSE	Nuclear transport factor 2 OS=Mus musculus GN=Nutf2 PE=2 SV=1	2	12	1	3.47E+05
Q99PR8	HSPB2_MOUSE	Heat shock protein beta-2 OS=Mus musculus GN=Hspb2 PE=2 SV=1	1	5	0.99	6.20E+04
Q9JHU2	PALMD_MOUSE	Palmdelphin OS=Mus musculus GN=Palmd PE=1 SV=1	2	5	1	1.21E+05
P08122	CO4A2_MOUSE	Collagen alpha-2(IV) chain OS=Mus musculus GN=Col4a2 PE=2 SV=4	4	13	1	5.05E+05
Q9JJU8	SH3L1_MOUSE	SH3 domain-binding glutamic acid-rich-like protein OS=Mus musculus GN=Sh3bgrl PE=2 SV=1	1	4	0.99	4.41E+04
P42932	TCPQ_MOUSE	T-complex protein 1 subunit theta OS=Mus musculus GN=Cct8 PE=1 SV=3	2	5	1	6.89E+04
P35385	HSPB7_MOUSE	Heat shock protein beta-7 OS=Mus musculus GN=Hspb7 PE=1 SV=2	3	13	1	4.42E+05
P40124	CAP1_MOUSE	Adenylyl cyclase-associated protein 1 OS=Mus musculus GN=Cap1 PE=1 SV=3	4	12	1	3.95E+05
Q8R1S0	COQ6_MOUSE	Ubiquinone biosynthesis monooxygenase COQ6 OS=Mus musculus GN=Coq6 PE=2 SV=2	5	9	1	2.97E+05
Q9DBJ1	PGAM1_MOUSE	Phosphoglycerate mutase 1 OS=Mus musculus GN=Pgam1 PE=1 SV=3	5	13	1	5.48E+05
P97447	FHL1_MOUSE	Four and a half LIM domains protein 1 OS=Mus musculus GN=Fhl1 PE=2 SV=3	6	12	1	1.91E+05
Q9JII6	AK1A1_MOUSE	Alcohol dehydrogenase [NADP+] OS=Mus musculus GN=Akr1a1 PE=1 SV=3	1	3	0.99	5.67E+04
Q9CQV8	1433B_MOUSE	14-3-3 protein beta/alpha OS=Mus musculus GN=Ywhab PE=1 SV=3	5	13	1	7.76E+05
Q60994	ADIPO_MOUSE	Adiponectin OS=Mus musculus GN=Adipoq PE=1 SV=1	3	12	1	9.02E+05
P06745	G6PI_MOUSE	Glucose-6-phosphate isomerase OS=Mus musculus GN=Gpi PE=1 SV=4	13	13	1	8.83E+06
Q8CHP8	PGP_MOUSE	Phosphoglycolate phosphatase OS=Mus musculus GN=Pgp PE=2 SV=1	1	6	0.99	3.12E+04
Q04857	CO6A1_MOUSE	Collagen alpha-1(VI) chain OS=Mus musculus GN=Col6a1 PE=2 SV=1	6	13	1	8.97E+05
Q61316	HSP74_MOUSE	Heat shock 70 kDa protein 4 OS=Mus musculus GN=Hspa4 PE=1 SV=1	9	13	1	7.20E+05
Q62188	DPYL3_MOUSE	Dihydropyrimidinase-related protein 3 OS=Mus musculus GN=Dpysl3 PE=1 SV=1	3	7	1	5.73E+04
Q9Z1Q5	CLIC1_MOUSE	Chloride intracellular channel protein 1 OS=Mus musculus GN=Clic1 PE=1 SV=3	1	4	1	3.75E+04
Q9ES97	RTN3_MOUSE	Reticulon-3 OS=Mus musculus GN=Rtn3 PE=1 SV=2	1	13	0.99	1.42E+05
O70400	PDLI1_MOUSE	PDZ and LIM domain protein 1 OS=Mus musculus GN=Pdlim1 PE=2 SV=4	2	11	1	3.87E+05
Q02013	AQP1_MOUSE	Aquaporin-1 OS=Mus musculus GN=Aqp1 PE=1 SV=3	3	12	1	5.33E+05
P60843	IF4A1_MOUSE	Eukaryotic initiation factor 4A-I OS=Mus musculus GN=Eif4a1 PE=2 SV=1	3	12	1	4.12E+05
Q02257	PLAK_MOUSE	Junction plakoglobin OS=Mus musculus GN=Jup PE=2 SV=3	16	13	1	1.59E+06
Q9CXW4	RL11_MOUSE	60S ribosomal protein L11 OS=Mus musculus GN=Rpl11 PE=1 SV=4	2	13	1	1.45E+05
P62774	MTPN_MOUSE	Myotrophin OS=Mus musculus GN=Mtpn PE=2 SV=2	1	7	0.99	2.76E+04
P68510	1433F_MOUSE	14-3-3 protein eta OS=Mus musculus GN=Ywhah PE=1 SV=2	1	7	1	4.88E+04
P07759	SPA3K_MOUSE	Serine protease inhibitor A3K OS=Mus musculus GN=Serpina3k PE=1 SV=2	4	13	1	4.70E+05
P29341	PABP1_MOUSE	Polyadenylate-binding protein 1 OS=Mus musculus GN=Pabpc1 PE=1 SV=1	1	3	1	7.16E+04
P24527	LKHA4_MOUSE	Leukotriene A-4 hydrolase OS=Mus musculus GN=Lta4h PE=1 SV=3	1	7	0.99	6.11E+04
P09528	FRIH_MOUSE	Ferritin heavy chain OS=Mus musculus GN=Fth1 PE=1 SV=2	5	13	1	8.44E+05
Q60864	STIP1_MOUSE	Stress-induced-phosphoprotein 1 OS=Mus musculus GN=Stip1 PE=1 SV=1	5	11	1	4.21E+05
P83940	ELOC_MOUSE	Transcription elongation factor B polypeptide 1 OS=Mus musculus GN=Tceb1 PE=1 SV=1	1	1	1	2.67E+04
P51885	LUM_MOUSE	Lumican OS=Mus musculus GN=Lum PE=1 SV=2	3	8	1	3.48E+05
P61164	ACTZ_MOUSE	Alpha-centractin OS=Mus musculus GN=Actr1a PE=2 SV=1	1	10	1	1.54E+05
Q8R5C5	ACTY_MOUSE	Beta-centractin OS=Mus musculus GN=Actr1b PE=1 SV=1	1	1	1	7.99E+04
Q04447	KCRB_MOUSE	Creatine kinase B-type OS=Mus musculus GN=Ckb PE=1 SV=1	2	13	1	1.92E+05
P40630	TFAM_MOUSE	Transcription factor A, mitochondrial OS=Mus musculus GN=Tfam PE=2 SV=2	2	3	1	6.95E+04
Q9WVE8	PACN2_MOUSE	Protein kinase C and casein kinase substrate in neurons protein 2 OS=Mus musculus GN=Pacsin2 PE=2 SV=1	1	3	0.96	5.03E+04
O55023	IMPA1_MOUSE	Inositol monophosphatase OS=Mus musculus GN=Impa1 PE=2 SV=1	1	12	0.58	1.70E+06
O35887	CALU_MOUSE	Calumenin OS=Mus musculus GN=Calu PE=1 SV=1	3	10	1	1.29E+05
Q9D020	5NT3_MOUSE	Cytosolic 5'-nucleotidase 3 OS=Mus musculus GN=Nt5c3 PE=1 SV=4	4	9	1	1.05E+05
Q9CVB6	ARPC2_MOUSE	Actin-related protein 2/3 complex subunit 2 OS=Mus musculus GN=Arpc2 PE=1 SV=3	2	4	1	1.98E+05
P00920	CAH2_MOUSE	Carbonic anhydrase 2 OS=Mus musculus GN=Ca2 PE=1 SV=4	4	13	1	3.35E+05
Q8VDQ1	PTGR2_MOUSE	Prostaglandin reductase 2 OS=Mus musculus GN=Ptgr2 PE=1 SV=2	4	13	1	5.57E+05
P80314	TCPB_MOUSE	T-complex protein 1 subunit beta OS=Mus musculus GN=Cct2 PE=1 SV=4	5	5	1	3.51E+05
Q9CPY7	AMPL_MOUSE	Cytosol aminopeptidase OS=Mus musculus GN=Lap3 PE=1 SV=3	2	8	1	1.75E+05
P29391	FRIL1_MOUSE	Ferritin light chain 1 OS=Mus musculus GN=Ftl1 PE=1 SV=2	1	11	0.99	1.29E+05
P97443	SMYD1_MOUSE	SET and MYND domain-containing protein 1 OS=Mus musculus GN=Smyd1 PE=1 SV=2	7	13	1	1.62E+06
P82350	SGCA_MOUSE	Alpha-sarcoglycan OS=Mus musculus GN=Sgca PE=1 SV=1	3	10	1	1.02E+05
O88342	WDR1_MOUSE	WD repeat-containing protein 1 OS=Mus musculus GN=Wdr1 PE=1 SV=3	6	11	1	3.44E+05
Q02053	UBA1_MOUSE	Ubiquitin-like modifier-activating enzyme 1 OS=Mus musculus GN=Uba1 PE=1 SV=1	13	13	1	1.73E+06
Q8R4N0	CLYBL_MOUSE	Citrate lyase subunit beta-like protein, mitochondrial OS=Mus musculus GN=Clybl PE=2 SV=2	4	13	1	4.97E+05
Q8K310	MATR3_MOUSE	Matrin-3 OS=Mus musculus GN=Matr3 PE=1 SV=1	2	5	1	1.21E+05
Q62417	SRBS1_MOUSE	Sorbin and SH3 domain-containing protein 1 OS=Mus musculus GN=Sorbs1 PE=1 SV=2	6	13	1	4.86E+05
P97371	PSME1_MOUSE	Proteasome activator complex subunit 1 OS=Mus musculus GN=Psme1 PE=2 SV=2	5	13	1	4.37E+05
P07901	HS90A_MOUSE	Heat shock protein HSP 90-alpha OS=Mus musculus GN=Hsp90aa1 PE=1 SV=4	3	9	1	1.05E+05
Q9Z2U1	PSA5_MOUSE	Proteasome subunit alpha type-5 OS=Mus musculus GN=Psma5 PE=1 SV=1	3	13	1	3.08E+05
Q7TMM9	TBB2A_MOUSE	Tubulin beta-2A chain OS=Mus musculus GN=Tubb2a PE=1 SV=1	1	13	1	5.78E+04
P05125	ANF_MOUSE	Atrial natriuretic factor OS=Mus musculus GN=Nppa PE=2 SV=1	2	2	1	8.83E+03
P02469	LAMB1_MOUSE	Laminin subunit beta-1 OS=Mus musculus GN=Lamb1-1 PE=1 SV=2	14	13	1	1.81E+06
Q9D7X3	DUS3_MOUSE	Dual specificity protein phosphatase 3 OS=Mus musculus GN=Dusp3 PE=1 SV=1	1	1	0.99	1.89E+04
Q9QYB1	CLIC4_MOUSE	Chloride intracellular channel protein 4 OS=Mus musculus GN=Clic4 PE=1 SV=3	3	11	1	2.02E+05
Q99JY3	GIMA4_MOUSE	GTPase IMAP family member 4 OS=Mus musculus GN=Gimap4 PE=2 SV=1	1	11	0.99	8.38E+04
Q01730	RSU1_MOUSE	Ras suppressor protein 1 OS=Mus musculus GN=Rsu1 PE=2 SV=3	3	11	1	3.07E+05
Q9Z2X1	HNRPF_MOUSE	Heterogeneous nuclear ribonucleoprotein F OS=Mus musculus GN=Hnrnpf PE=1 SV=3	2	3	1	3.72E+04
Q60854	SPB6_MOUSE	Serpin B6 OS=Mus musculus GN=Serpinb6 PE=2 SV=1	4	13	1	3.61E+05
P54728	RD23B_MOUSE	UV excision repair protein RAD23 homolog B OS=Mus musculus GN=Rad23b PE=1 SV=1	1	1	0.59	3.49E+04
P63330	PP2AA_MOUSE	Serine/threonine-protein phosphatase 2A catalytic subunit alpha isoform OS=Mus musculus GN=Ppp2ca PE=1 SV=1	2	13	1	2.54E+05
Q64105	SPRE_MOUSE	Sepiapterin reductase OS=Mus musculus GN=Spr PE=1 SV=1	4	13	1	3.84E+05
Q9JHW2	NIT2_MOUSE	Nitrilase homolog 2 OS=Mus musculus GN=Nit2 PE=1 SV=1	4	10	1	1.58E+05
P01869	IGH1M_MOUSE	Ig gamma-1 chain C region, membrane-bound form OS=Mus musculus GN=Ighg1 PE=1 SV=2	3	7	1	1.72E+05
P56565	S10A1_MOUSE	Protein S100-A1 OS=Mus musculus GN=S100a1 PE=1 SV=2	2	13	1	4.65E+05
Q91ZJ5	UGPA_MOUSE	UTP--glucose-1-phosphate uridylyltransferase OS=Mus musculus GN=Ugp2 PE=2 SV=3	9	13	1	1.61E+06
P97429	ANXA4_MOUSE	Annexin A4 OS=Mus musculus GN=Anxa4 PE=2 SV=3	1	6	0.99	2.77E+04
Q9D8N0	EF1G_MOUSE	Elongation factor 1-gamma OS=Mus musculus GN=Eef1g PE=1 SV=3	7	13	1	6.75E+05
O70433	FHL2_MOUSE	Four and a half LIM domains protein 2 OS=Mus musculus GN=Fhl2 PE=1 SV=1	10	13	1	1.06E+06
P56375	ACYP2_MOUSE	Acylphosphatase-2 OS=Mus musculus GN=Acyp2 PE=2 SV=1	2	13	1	2.89E+05
P50247	SAHH_MOUSE	Adenosylhomocysteinase OS=Mus musculus GN=Ahcy PE=1 SV=3	5	13	1	4.35E+05
Q8CDN6	TXNL1_MOUSE	Thioredoxin-like protein 1 OS=Mus musculus GN=Txnl1 PE=2 SV=3	1	3	0.96	9.52E+04
O55234	PSB5_MOUSE	Proteasome subunit beta type-5 OS=Mus musculus GN=Psmb5 PE=1 SV=3	2	6	1	7.55E+04
Q8BWB1	SYP2L_MOUSE	Synaptopodin 2-like protein OS=Mus musculus GN=Synpo2l PE=2 SV=1	6	10	1	3.06E+05
P70349	HINT1_MOUSE	Histidine triad nucleotide-binding protein 1 OS=Mus musculus GN=Hint1 PE=1 SV=3	1	13	1	5.10E+05
Q8VIJ6	SFPQ_MOUSE	Splicing factor, proline- and glutamine-rich OS=Mus musculus GN=Sfpq PE=1 SV=1	4	12	1	1.98E+05
P80315	TCPD_MOUSE	T-complex protein 1 subunit delta OS=Mus musculus GN=Cct4 PE=1 SV=3	5	8	1	3.25E+05
P62827	RAN_MOUSE	GTP-binding nuclear protein Ran OS=Mus musculus GN=Ran PE=1 SV=3	1	7	1	9.37E+04
P36536	SAR1A_MOUSE	GTP-binding protein SAR1a OS=Mus musculus GN=Sar1a PE=2 SV=1	1	5	0.99	9.41E+04
Q9CQC9	SAR1B_MOUSE	GTP-binding protein SAR1b OS=Mus musculus GN=Sar1b PE=1 SV=1	2	5	1	9.92E+04
Q9Z2W0	DNPEP_MOUSE	Aspartyl aminopeptidase OS=Mus musculus GN=Dnpep PE=2 SV=1	4	10	1	3.37E+05
P15864	H13_MOUSE	Histone H1.2 OS=Mus musculus GN=Hist1h1c PE=1 SV=2	1	8	0.99	1.17E+05
Q62048	PEA15_MOUSE	Astrocytic phosphoprotein PEA-15 OS=Mus musculus GN=Pea15 PE=1 SV=1	1	1	0.99	3.22E+04
P80318	TCPG_MOUSE	T-complex protein 1 subunit gamma OS=Mus musculus GN=Cct3 PE=1 SV=1	6	9	1	3.68E+05
P10639	THIO_MOUSE	Thioredoxin OS=Mus musculus GN=Txn PE=1 SV=3	1	13	1	3.79E+05
P31786	ACBP_MOUSE	Acyl-CoA-binding protein OS=Mus musculus GN=Dbi PE=1 SV=2	2	13	1	2.49E+06
P00397	COX1_MOUSE	Cytochrome c oxidase subunit 1 OS=Mus musculus GN=Mtco1 PE=3 SV=1	3	13	1	3.79E+05
Q5EBG6	HSPB6_MOUSE	Heat shock protein beta-6 OS=Mus musculus GN=Hspb6 PE=2 SV=1	4	13	1	1.31E+06
Q7TQI3	OTUB1_MOUSE	Ubiquitin thioesterase OTUB1 OS=Mus musculus GN=Otub1 PE=1 SV=2	2	7	1	5.19E+04
Q61166	MARE1_MOUSE	Microtubule-associated protein RP/EB family member 1 OS=Mus musculus GN=Mapre1 PE=1 SV=3	1	1	0.99	3.83E+04
O08997	ATOX1_MOUSE	Copper transport protein ATOX1 OS=Mus musculus GN=Atox1 PE=2 SV=1	2	3	1	2.02E+05
Q9R0P3	ESTD_MOUSE	S-formylglutathione hydrolase OS=Mus musculus GN=Esd PE=2 SV=1	2	13	1	1.21E+06
P46471	PRS7_MOUSE	26S protease regulatory subunit 7 OS=Mus musculus GN=Psmc2 PE=1 SV=5	1	6	1	4.44E+04
P22599	A1AT2_MOUSE	Alpha-1-antitrypsin 1-2 OS=Mus musculus GN=Serpina1b PE=1 SV=2	1	13	1	1.62E+05
Q00897	A1AT4_MOUSE	Alpha-1-antitrypsin 1-4 OS=Mus musculus GN=Serpina1d PE=2 SV=1	4	13	1	5.46E+05
Q00898	A1AT5_MOUSE	Alpha-1-antitrypsin 1-5 OS=Mus musculus GN=Serpina1e PE=1 SV=1	3	13	1	7.64E+05
Q8BG95	MYPT2_MOUSE	Protein phosphatase 1 regulatory subunit 12B OS=Mus musculus GN=Ppp1r12b PE=1 SV=2	4	12	1	2.93E+05
Q9CQX2	CYB5B_MOUSE	Cytochrome b5 type B OS=Mus musculus GN=Cyb5b PE=1 SV=1 [MASS=16318]	1	13	0.99	4.31E+04
Q9R0Q7	TEBP_MOUSE	Prostaglandin E synthase 3 OS=Mus musculus GN=Ptges3 PE=1 SV=1 [MASS=18721]	2	9	1	9.95E+04
Q8JZN5	ACAD9_MOUSE	Acyl-CoA dehydrogenase family member 9, mitochondrial OS=Mus musculus GN=Acad9 PE=2 SV=1 [MASS=68707]	8	13	1	9.13E+05
P85094	ISC2A_MOUSE	Isochorismatase domain-containing protein 2A, mitochondrial OS=Mus musculus GN=Isoc2a PE=2 SV=1 [MASS=22417]	3	11	1	2.73E+05
Q9D1H6	NDUF4_MOUSE	NADH dehydrogenase [ubiquinone] 1 alpha subcomplex assembly factor 4 OS=Mus musculus GN=Ndufaf4 PE=2 SV=1 [MASS=20082]	1	5	0.97	2.32E+04
Q9QYA2	TOM40_MOUSE	Mitochondrial import receptor subunit TOM40 homolog OS=Mus musculus GN=Tomm40 PE=1 SV=3 [MASS=37895]	3	9	1	2.27E+05
Q8R164	BPHL_MOUSE	Valacyclovir hydrolase OS=Mus musculus GN=Bphl PE=2 SV=1 [MASS=32851]	2	13	1	1.35E+05
Q8BWM0	PGES2_MOUSE	Prostaglandin E synthase 2 OS=Mus musculus GN=Ptges2 PE=1 SV=2 [MASS=43307]	3	10	1	1.81E+05
Q99JI4	PSMD6_MOUSE	26S proteasome non-ATPase regulatory subunit 6 OS=Mus musculus GN=Psmd6 PE=2 SV=1	2	2	1	5.75E+04
P49722	PSA2_MOUSE	Proteasome subunit alpha type-2 OS=Mus musculus GN=Psma2 PE=1 SV=2	2	3	1	1.75E+05
O09061	PSB1_MOUSE	Proteasome subunit beta type-1 OS=Mus musculus GN=Psmb1 PE=1 SV=1	4	12	1	3.72E+05
Q9R1P3	PSB2_MOUSE	Proteasome subunit beta type-2 OS=Mus musculus GN=Psmb2 PE=1 SV=1	2	4	1	1.33E+05
Q9R1P4	PSA1_MOUSE	Proteasome subunit alpha type-1 OS=Mus musculus GN=Psma1 PE=1 SV=1	2	13	1	1.33E+05
P50136	ODBA_MOUSE	2-oxoisovalerate dehydrogenase subunit alpha, mitochondrial OS=Mus musculus GN=Bckdha PE=1 SV=1	6	11	1	7.56E+05
Q9R1P1	PSB3_MOUSE	Proteasome subunit beta type-3 OS=Mus musculus GN=Psmb3 PE=1 SV=1	1	4	0.99	3.53E+04
Q8VDM4	PSMD2_MOUSE	26S proteasome non-ATPase regulatory subunit 2 OS=Mus musculus GN=Psmd2 PE=1 SV=1	6	10	1	4.93E+05
P54775	PRS6B_MOUSE	26S protease regulatory subunit 6B OS=Mus musculus GN=Psmc4 PE=1 SV=1	2	2	1	1.12E+05
O35295	PURB_MOUSE	Transcriptional activator protein Pur-beta OS=Mus musculus GN=Purb PE=1 SV=3	1	1	0.93	1.92E+04
Q9Z1E4	GYS1_MOUSE	Glycogen [starch] synthase, muscle OS=Mus musculus GN=Gys1 PE=1 SV=2	2	12	1	1.23E+05

P14685	PSMD3_MOUSE	26S proteasome non-ATPase regulatory subunit 3 OS=Mus musculus GN=Psmd3 PE=2 SV=2	1	6	0.99	7.21E+04	4.75E+04	4.41E+04
Q3TXS7	PSMD1_MOUSE	26S proteasome non-ATPase regulatory subunit 1 OS=Mus musculus GN=Psmd1 PE=1 SV=1	1	3	1	1.85E+04	1.69E+04	1.65E+04
Q64481	CP3AG_MOUSE	Cytochrome P450 3A16 OS=Mus musculus GN=Cyp3a16 PE=2 SV=1	1	10	0.89	1.01E+05	1.18E+05	1.18E+05
O88685	PRS6A_MOUSE	26S protease regulatory subunit 6A OS=Mus musculus GN=Psmc3 PE=2 SV=1	4	11	1	1.95E+05	1.69E+05	1.42E+05
Q9JJL8	SYSM_MOUSE	Seryl-tRNA synthetase, mitochondrial OS=Mus musculus GN=Sars2 PE=2 SV=1	1	10	0.99	6.74E+04	5.44E+04	1.68E+04
Q99KJ8	DCTN2_MOUSE	Dynactin subunit 2 OS=Mus musculus GN=Dctn2 PE=1 SV=3	3	4	1	5.50E+04	7.85E+04	8.09E+04
P62334	PRS10_MOUSE	26S protease regulatory subunit S10B OS=Mus musculus GN=Psmc6 PE=1 SV=1	2	5	1	1.35E+05	1.10E+05	1.22E+05
P62196	PRS8_MOUSE	26S protease regulatory subunit 8 OS=Mus musculus GN=Psmc5 PE=1 SV=1	1	13	1	1.27E+05	1.30E+05	2.87E+05
P62192	PRS4_MOUSE	26S protease regulatory subunit 4 OS=Mus musculus GN=Psmc1 PE=1 SV=1	1	1	0.99	5.91E+04	4.36E+04	3.92E+04
Q8JZQ9	EIF3B_MOUSE	Eukaryotic translation initiation factor 3 subunit B OS=Mus musculus GN=Eif3b PE=1 SV=1	1	1	0.99	3.45E+04	1.87E+04	2.81E+03
Q8R001	MARE2_MOUSE	Microtubule-associated protein RP/EB family member 2 OS=Mus musculus GN=Mapre2 PE=1 SV=1	1	5	0.99	1.22E+05	1.11E+05	9.38E+04
Q9QZD9	EIF3I_MOUSE	Eukaryotic translation initiation factor 3 subunit I OS=Mus musculus GN=Eif3i PE=1 SV=1	1	2	0.99	1.59E+04	1.50E+04	5.49E+03
Q9D8W5	PSD12_MOUSE	26S proteasome non-ATPase regulatory subunit 12 OS=Mus musculus GN=Psmd12 PE=2 SV=3	2	1	1	7.02E+04	5.83E+04	6.18E+04
Q6NWW9	FND3B_MOUSE	Fibronectin type III domain-containing protein 3B OS=Mus musculus GN=Fndc3b PE=1 SV=1	1	10	0.97	1.95E+04	1.98E+04	2.77E+04
O70251	EF1B_MOUSE	Elongation factor 1-beta OS=Mus musculus GN=Eef1b PE=1 SV=5	2	13	1	1.14E+05	1.15E+05	1.84E+05
Q9DCH4	EIF3F_MOUSE	Eukaryotic translation initiation factor 3 subunit F OS=Mus musculus GN=Eif3f PE=1 SV=1	1	1	1	8.82E+04	9.01E+04	6.52E+04
P35908	K22E_HUMAN	Keratin, type II cytoskeletal 2 epidermal OS=Homo sapiens GN=KRT2 PE=1 SV=2	12	7	1	3.02E+05	1.66E+06	3.53E+05
P13645	K1C10_HUMAN	Keratin, type I cytoskeletal 10 OS=Homo sapiens GN=KRT10 PE=1 SV=6	14	13	1	1.40E+06	8.30E+06	1.61E+06
P35527	K1C9_HUMAN	Keratin, type I cytoskeletal 9 OS=Homo sapiens GN=KRT9 PE=1 SV=3	13	12	1	3.19E+06	1.28E+07	3.98E+06
P04264	K2C1_HUMAN	Keratin, type II cytoskeletal 1 OS=Homo sapiens GN=KRT1 PE=1 SV=6	14	13	1	3.43E+06	1.40E+07	3.98E+06
P02533	K1C14_HUMAN	Keratin, type I cytoskeletal 14 OS=Homo sapiens GN=KRT14 PE=1 SV=4	1	5	1	4.44E+04	1.37E+05	5.37E+04
P13647	K2C5_HUMAN	Keratin, type II cytoskeletal 5 OS=Homo sapiens GN=KRT5 PE=1 SV=3	4	5	1	5.36E+04	1.22E+05	6.63E+04
P02538	K2C6A_HUMAN	Keratin, type II cytoskeletal 6A OS=Homo sapiens GN=KRT6A PE=1 SV=3	1	2	1	4.18E+03	1.32E+04	1.18E+04
P08779	K1C16_HUMAN	Keratin, type I cytoskeletal 16 OS=Homo sapiens GN=KRT16 PE=1 SV=4	2	4	1	4.92E+04	1.35E+05	2.10E+05
Q9CY64	BIEA_MOUSE	Biliverdin reductase A OS=Mus musculus GN=Blvra PE=2 SV=1	1	2	0.73	1.12E+04	1.67E+04	1.38E+04
Q9D967	MGDP1_MOUSE	Magnesium-dependent phosphatase 1 OS=Mus musculus GN=Mdp1 PE=1 SV=1	1	5	1	7.32E+04	9.21E+04	7.37E+04
P97855	G3BP1_MOUSE	Ras GTPase-activating protein-binding protein 1 OS=Mus musculus GN=G3bp1 PE=1 SV=1	3	2	1	7.32E+04	1.25E+05	1.00E+05
Q6P1B1	XPP1_MOUSE	Xaa-Pro aminopeptidase 1 OS=Mus musculus GN=Xpnpep1 PE=2 SV=1	1	1	0.95	5.87E+05	5.55E+05	1.97E+05
Q9WUA3	K6PP_MOUSE	6-phosphofructokinase type C OS=Mus musculus GN=Pfkp PE=1 SV=1	2	2	1	5.29E+04	2.92E+04	3.56E+04
Q9DCD0	6PGD_MOUSE	6-phosphogluconate dehydrogenase, decarboxylating OS=Mus musculus GN=Pgd PE=2 SV=3	1	1	0.76	9.35E+01	1.08E+03	2.54E+03
Q9WU28	PFD5_MOUSE	Prefoldin subunit 5 OS=Mus musculus GN=Pfdn5 PE=2 SV=1	1	1	0.82	5.49E+04	5.09E+04	2.37E+04
Q9CZM2	RL15_MOUSE	60S ribosomal protein L15 OS=Mus musculus GN=Rpl15 PE=2 SV=4	1	7	1	4.86E+04	3.54E+04	1.87E+04
P48758	CBR1_MOUSE	Carbonyl reductase [NADPH] 1 OS=Mus musculus GN=Cbr1 PE=1 SV=3	2	7	1	6.21E+04	5.09E+04	4.72E+04
P56399	UBP5_MOUSE	Ubiquitin carboxyl-terminal hydrolase 5 OS=Mus musculus GN=Usp5 PE=1 SV=1	6	13	1	1.95E+05	2.37E+05	2.22E+05
Q9ERK4	XPO2_MOUSE	Exportin-2 OS=Mus musculus GN=Cse1l PE=2 SV=1	2	3	1	1.00E+05	9.33E+04	7.46E+04
Q64737	PUR2_MOUSE	Trifunctional purine biosynthetic protein adenosine-3 OS=Mus musculus GN=Gart PE=2 SV=2	2	9	1	1.49E+05	1.21E+05	1.28E+05
Q9CQW1	YKT6_MOUSE	Synaptobrevin homolog YKT6 OS=Mus musculus GN=Ykt6 PE=2 SV=1	1	1	0.97	4.27E+04	3.60E+04	3.94E+04
Q61768	KINH_MOUSE	Kinesin-1 heavy chain OS=Mus musculus GN=Kif5b PE=1 SV=2	1	4	1	2.08E+04	1.99E+04	1.06E+04
Q8BKC5	IPO5_MOUSE	Importin-5 OS=Mus musculus GN=Ipo5 PE=1 SV=3	1	7	1	6.54E+04	4.36E+04	4.18E+04
P12367	KAP2_MOUSE	cAMP-dependent protein kinase type II-alpha regulatory subunit OS=Mus musculus GN=Prkar2a PE=1 SV=2	1	11	0.99	1.20E+05	1.15E+05	1.09E+05
P54116	STOM_MOUSE	Erythrocyte band 7 integral membrane protein OS=Mus musculus GN=Stom PE=1 SV=3	2	7	1	1.71E+05	1.24E+05	1.11E+05
P80316	TCPE_MOUSE	T-complex protein 1 subunit epsilon OS=Mus musculus GN=Cct5 PE=1 SV=1	4	11	1	4.26E+05	2.89E+05	2.47E+05
Q6P5F9	XPO1_MOUSE	Exportin-1 OS=Mus musculus GN=Xpo1 PE=1 SV=1	3	1	1	1.25E+05	7.88E+04	7.33E+04
P56376	ACYP1_MOUSE	Acylphosphatase-1 OS=Mus musculus GN=Acyp1 PE=2 SV=2	2	11	1	9.17E+04	1.11E+05	5.27E+04
Q9QZ23	NFU1_MOUSE	NFU1 iron-sulfur cluster scaffold homolog, mitochondrial OS=Mus musculus GN=Nfu1 PE=1 SV=2	2	12	1	1.22E+05	1.02E+05	1.31E+05
Q8K1M6	DNM1L_MOUSE	Dynamin-1-like protein OS=Mus musculus GN=Dnm1l PE=1 SV=2	9	13	1	9.24E+05	7.84E+05	7.42E+05
Q8CGC7	SYEP_MOUSE	Bifunctional aminoacyl-tRNA synthetase OS=Mus musculus GN=Eprs PE=2 SV=3	3	13	1	2.79E+05	2.44E+05	1.45E+05
Q9QUR6	PPCE_MOUSE	Prolyl endopeptidase OS=Mus musculus GN=Prep PE=2 SV=1	1	2	1	3.73E+04	2.20E+04	2.69E+04
Q9Z1Z2	STRAP_MOUSE	Serine-threonine kinase receptor-associated protein OS=Mus musculus GN=Strap PE=1 SV=2	1	1	0.59	9.49E+03	1.17E+04	1.95E+04
P58774	TPM2_MOUSE	Tropomyosin beta chain OS=Mus musculus GN=Tpm2 PE=1 SV=1	1	11	1	4.51E+04	4.68E+04	2.09E+04
Q9D6Y9	GLGB_MOUSE	1,4-alpha-glucan-branching enzyme OS=Mus musculus GN=Gbe1 PE=2 SV=1	1	5	0.98	4.19E+04	4.28E+04	5.31E+04
Q8VDJ3	VIGLN_MOUSE	Vigilin OS=Mus musculus GN=Hdlbp PE=1 SV=1	2	10	1	6.65E+04	5.80E+04	4.49E+04
Q8K0D5	EFGM_MOUSE	Elongation factor G, mitochondrial OS=Mus musculus GN=Gfm1 PE=2 SV=1	2	2	1	3.28E+04	3.85E+04	6.29E+04
Q9DC77	SMPX_MOUSE	Small muscular protein OS=Mus musculus GN=Smpx PE=2 SV=2	3	13	1	4.33E+05	4.19E+05	2.53E+05
P11276	FINC_MOUSE	Fibronectin OS=Mus musculus GN=Fn1 PE=1 SV=3	2	1	1	3.50E+04	3.60E+04	3.13E+04
Q9CR16	PPID_MOUSE	Peptidyl-prolyl cis-trans isomerase D OS=Mus musculus GN=Ppid PE=1 SV=3	1	1	0.88	2.93E+03	1.50E+04	1.98E+04
Q9CZY3	UB2V1_MOUSE	Ubiquitin-conjugating enzyme E2 variant 1 OS=Mus musculus GN=Ube2v1 PE=1 SV=1	1	7	1	5.51E+04	4.66E+04	2.07E+04
P84089	ERH_MOUSE	Enhancer of rudimentary homolog OS=Mus musculus GN=Erh PE=1 SV=1	1	10	0.99	3.63E+04	3.41E+04	5.67E+03
P51859	HDGF_MOUSE	Hepatoma-derived growth factor OS=Mus musculus GN=Hdgf PE=1 SV=2	1	2	0.98	3.79E+04	3.43E+04	3.31E+04
Q8CGY6	UN45B_MOUSE	Protein unc-45 homolog B OS=Mus musculus GN=Unc45b PE=2 SV=1	3	9	1	1.77E+05	1.26E+05	8.08E+04
Q9CQ45	NENF_MOUSE	Neudesin OS=Mus musculus GN=Nenf PE=1 SV=1	1	1	0.94	3.97E+04	3.57E+04	2.78E+04
Q91VI7	RINI_MOUSE	Ribonuclease inhibitor OS=Mus musculus GN=Rnh1 PE=2 SV=1	2	7	1	7.81E+04	5.95E+04	1.13E+05
Q9JMH6	TRXR1_MOUSE	Thioredoxin reductase 1, cytoplasmic OS=Mus musculus GN=Txnrd1 PE=1 SV=3	1	2	0.78	6.03E+03	4.90E+03	1.97E+03
P54822	PUR8_MOUSE	Adenylosuccinate lyase OS=Mus musculus GN=Adsl PE=2 SV=1	2	9	1	3.82E+04	3.79E+04	6.47E+04
P50516	VATA_MOUSE	V-type proton ATPase catalytic subunit A OS=Mus musculus GN=Atp6v1a PE=1 SV=2	1	1	0.99	9.79E+03	1.47E+04	1.81E+04
P80313	TCPH_MOUSE	T-complex protein 1 subunit eta OS=Mus musculus GN=Cct7 PE=1 SV=1	3	13	1	2.03E+05	1.74E+05	1.36E+05
Q99KB8	GLO2_MOUSE	Hydroxyacylglutathione hydrolase, mitochondrial OS=Mus musculus GN=Hagh PE=2 SV=2	2	11	1	2.11E+05	1.68E+05	9.14E+04
Q8BP47	SYNC_MOUSE	Asparaginyl-tRNA synthetase, cytoplasmic OS=Mus musculus GN=Nars PE=1 SV=2	2	2	1	3.51E+04	2.67E+04	3.40E+04
P62315	SMD1_MOUSE	Small nuclear ribonucleoprotein Sm D1 OS=Mus musculus GN=Snrpd1 PE=2 SV=1	1	3	0.99	7.94E+04	5.51E+04	2.17E+04
P70168	IMB1_MOUSE	Importin subunit beta-1 OS=Mus musculus GN=Kpnb1 PE=1 SV=1	6	3	1	1.43E+05	1.32E+05	1.69E+05
Q91VR5	DDX1_MOUSE	ATP-dependent RNA helicase DDX1 OS=Mus musculus GN=Ddx1 PE=2 SV=1	4	12	1	2.41E+05	2.05E+05	2.10E+05
P62869	ELOB_MOUSE	Transcription elongation factor B polypeptide 2 OS=Mus musculus GN=Tceb2 PE=1 SV=1	1	1	0.94	1.27E+05	1.00E+05	6.94E+04
Q6V4S5	SDK2_MOUSE	Protein sidekick-2 OS=Mus musculus GN=Sdk2 PE=2 SV=1	1	5	0.59	3.76E+04	5.51E+04	4.63E+04
B2RUP2	UN13D_MOUSE	Protein unc-13 homolog D OS=Mus musculus GN=Unc13d PE=1 SV=2	1	10	0.97	7.70E+05	8.62E+05	7.29E+05
P17426	AP2A1_MOUSE	AP-2 complex subunit alpha-1 OS=Mus musculus GN=Ap2a1 PE=1 SV=1	1	1	1	2.51E+04	2.17E+04	1.24E+04
Q61584	FXR1_MOUSE	Fragile X mental retardation syndrome-related protein 1 OS=Mus musculus GN=Fxr1 PE=1 SV=2	2	4	1	5.80E+04	6.81E+04	5.48E+04
Q9CYR0	SSBP_MOUSE	Single-stranded DNA-binding protein, mitochondrial OS=Mus musculus GN=Ssbp1 PE=2 SV=1	2	10	1	1.91E+05	1.66E+05	1.08E+05
Q61937	NPM_MOUSE	Nucleophosmin OS=Mus musculus GN=Npm1 PE=1 SV=1	2	13	1	1.63E+05	1.67E+05	1.62E+05
Q9WTX5	SKP1_MOUSE	S-phase kinase-associated protein 1 OS=Mus musculus GN=Skp1 PE=1 SV=3	3	5	1	1.79E+05	1.36E+05	9.53E+04
Q05920	PYC_MOUSE	Pyruvate carboxylase, mitochondrial OS=Mus musculus GN=Pc PE=1 SV=1	1	1	0.99	2.45E+04	1.80E+04	2.11E+04
Q9D071	MMS19_MOUSE	MMS19 nucleotide excision repair protein homolog OS=Mus musculus GN=Mms19 PE=1 SV=1	1	3	0.71	5.80E+04	1.33E+05	9.12E+04
Q9JHU4	DYHC1_MOUSE	Cytoplasmic dynein 1 heavy chain 1 OS=Mus musculus GN=Dync1h1 PE=1 SV=1	22	13	1	1.18E+06	1.02E+06	1.01E+06
P26516	PSD7_MOUSE	26S proteasome non-ATPase regulatory subunit 7 OS=Mus musculus GN=Psmd7 PE=1 SV=2	1	5	0.99	1.34E+04	1.10E+04	1.62E+04
Q78ZA7	NP1L4_MOUSE	Nucleosome assembly protein 1-like 4 OS=Mus musculus GN=Nap1l4 PE=1 SV=1	2	11	1	1.06E+05	1.26E+05	1.18E+05
Q3UPL0	SC31A_MOUSE	Protein transport protein Sec31A OS=Mus musculus GN=Sec31a PE=1 SV=2	1	9	1	4.06E+04	2.51E+04	4.49E+03
Q9CQF3	CPSF5_MOUSE	Cleavage and polyadenylation specificity factor subunit 5 OS=Mus musculus GN=Nudt21 PE=2 SV=1	1	2	0.95	2.11E+05	1.75E+05	1.05E+05
Q9CYZ2	TPD54_MOUSE	Tumor protein D54 OS=Mus musculus GN=Tpd52l2 PE=1 SV=1	1	6	0.99	6.86E+04	6.08E+04	7.19E+04
Q7TPW1	NEXN_MOUSE	Nexilin OS=Mus musculus GN=Nexn PE=1 SV=2	1	8	0.99	4.14E+04	2.87E+04	3.95E+04
O35465	FKBP8_MOUSE	Peptidyl-prolyl cis-trans isomerase FKBP8 OS=Mus musculus GN=Fkbp8 PE=1 SV=2	1	1	0.98	5.13E+04	4.44E+04	1.80E+04
Q11011	PSA_MOUSE	Puromycin-sensitive aminopeptidase OS=Mus musculus GN=Npepps PE=1 SV=1	4	10	1	2.05E+05	1.84E+05	1.99E+05
Q60634	FLOT2_MOUSE	Flotillin-2 OS=Mus musculus GN=Flot2 PE=1 SV=2	1	3	1	3.13E+04	3.14E+04	1.34E+04
Q923D2	BLVRB_MOUSE	Flavin reductase OS=Mus musculus GN=Blvrb PE=2 SV=3	3	3	1	7.71E+04	7.12E+04	3.70E+04
P46412	GPX3_MOUSE	Glutathione peroxidase 3 OS=Mus musculus GN=Gpx3 PE=2 SV=2	3	12	1	1.04E+05	1.63E+05	1.92E+05
P62960	YBOX1_MOUSE	Nuclease-sensitive element-binding protein 1 OS=Mus musculus GN=Ybx1 PE=1 SV=3	3	13	1	1.59E+05	1.17E+05	5.35E+04
Q6NZJ6	IF4G1_MOUSE	Eukaryotic translation initiation factor 4 gamma 1 OS=Mus musculus GN=Eif4g1 PE=1 SV=1	1	4	0.8	1.20E+04	9.84E+03	1.35E+04
Q99KQ4	NAMPT_MOUSE	Nicotinamide phosphoribosyltransferase OS=Mus musculus GN=Nampt PE=1 SV=1	4	13	1	1.08E+06	9.27E+05	9.63E+05
Q8BVI4	DHPR_MOUSE	Dihydropteridine reductase OS=Mus musculus GN=Qdpr PE=1 SV=2	1	10	1	1.45E+05	1.51E+05	1.02E+05
Q9WV98	TIM9_MOUSE	Mitochondrial import inner membrane translocase subunit Tim9 OS=Mus musculus GN=Timm9 PE=1 SV=1	1	13	1	3.29E+05	3.30E+05	2.12E+05
P12382	K6PL_MOUSE	6-phosphofructokinase, liver type OS=Mus musculus GN=Pfkl PE=1 SV=3	1	2	1	4.86E+04	5.29E+04	3.81E+04
Q9CQ65	MTAP_MOUSE	S-methyl-5'-thioadenosine phosphorylase OS=Mus musculus GN=Mtap PE=2 SV=1	1	4	0.97	3.08E+04	4.25E+04	4.74E+04
Q99MN1	SYK_MOUSE	Lysyl-tRNA synthetase OS=Mus musculus GN=Kars PE=1 SV=1	1	5	0.94	9.51E+03	2.02E+04	2.16E+04
Q9DCM0	ETHE1_MOUSE	Protein ETHE1, mitochondrial OS=Mus musculus GN=Ethe1 PE=1 SV=2	3	7	1	1.15E+05	1.32E+05	1.34E+05
Q9CQZ1	HSBP1_MOUSE	Heat shock factor-binding protein 1 OS=Mus musculus GN=Hsbp1 PE=2 SV=1	1	1	0.88	5.01E+04	4.09E+04	3.46E+04
Q61234	SNTA1_MOUSE	Alpha-1-syntrophin OS=Mus musculus GN=Snta1 PE=1 SV=1	2	13	1	3.62E+05	3.18E+05	2.17E+05
Q9DCL9	PUR6_MOUSE	Multifunctional protein ADE2 OS=Mus musculus GN=Paics PE=1 SV=3	2	12	1	1.15E+05	9.49E+04	1.04E+05
Q99JW4	LIMS1_MOUSE	LIM and senescent cell antigen-like-containing domain protein 1 OS=Mus musculus GN=Lims1 PE=1 SV=3	1	9	0.93	2.73E+04	2.68E+04	3.78E+04
Q3UM45	PP1R7_MOUSE	Protein phosphatase 1 regulatory subunit 7 OS=Mus musculus GN=Ppp1r7 PE=1 SV=2	2	5	1	7.55E+04	5.70E+04	3.85E+04
Q91V64	ISOC1_MOUSE	Isochorismatase domain-containing protein 1 OS=Mus musculus GN=Isoc1 PE=2 SV=1	2	13	1	1.70E+05	1.07E+05	1.43E+05
P49312	ROA1_MOUSE	Heterogeneous nuclear ribonucleoprotein A1 OS=Mus musculus GN=Hnrnpa1 PE=1 SV=2	1	11	0.99	3.63E+04	2.49E+04	2.11E+03
Q9JKB1	UCHL3_MOUSE	Ubiquitin carboxyl-terminal hydrolase isozyme L3 OS=Mus musculus GN=Uchl3 PE=1 SV=2	2	11	1	4.30E+04	4.17E+04	7.41E+04
P23506	PIMT_MOUSE	Protein-L-isoaspartate(D-aspartate) O-methyltransferase OS=Mus musculus GN=Pcmt1 PE=1 SV=3	3	13	1	8.87E+04	9.64E+04	1.18E+05
Q9WVB0	RBPMS_MOUSE	RNA-binding protein with multiple splicing OS=Mus musculus GN=Rbpms PE=2 SV=1	1	3	0.99	2.78E+04	2.10E+04	6.77E+03
Q9D0M5	DYL2_MOUSE	Dynein light chain 2, cytoplasmic OS=Mus musculus GN=Dynll2 PE=1 SV=1	2	13	1	6.53E+04	8.36E+04	3.60E+05
P61161	ARP2_MOUSE	Actin-related protein 2 OS=Mus musculus GN=Actr2 PE=1 SV=1	1	9	0.99	5.25E+04	5.25E+04	6.15E+04
P56959	FUS_MOUSE	RNA-binding protein FUS OS=Mus musculus GN=Fus PE=2 SV=1	1	4	0.97	2.65E+04	2.36E+04	1.33E+04
P28271	ACOC_MOUSE	Cytoplasmic aconitate hydratase OS=Mus musculus GN=Aco1 PE=1 SV=2	3	4	1	2.24E+05	2.03E+05	1.42E+05
P27612	PLAP_MOUSE	Phospholipase A-2-activating protein OS=Mus musculus GN=Plaa PE=2 SV=3	1	1	0.98	2.78E+05	2.58E+05	1.84E+05
Q9QXM0	ABHD2_MOUSE	Abhydrolase domain-containing protein 2 OS=Mus musculus GN=Abhd2 PE=2 SV=1	1	2	0.96	2.55E+04	2.60E+04	7.12E+03
Q9DAK9	PHP14_MOUSE	14 kDa phosphohistidine phosphatase OS=Mus musculus GN=Phpt1 PE=1 SV=1	2	13	1	8.78E+04	1.42E+05	1.95E+05
Q9ESL4	MLTK_MOUSE	Mitogen-activated protein kinase kinase kinase MLT OS=Mus musculus GN=Mltk PE=1 SV=1	1	12	0.99	7.73E+04	6.11E+04	5.14E+04
Q9CQM9	GLRX3_MOUSE	Glutaredoxin-3 OS=Mus musculus GN=Glrx3 PE=1 SV=1	1	2	0.99	8.31E+03	4.82E+03	1.14E+04
P06801	MAOX_MOUSE	NADP-dependent malic enzyme OS=Mus musculus GN=Me1 PE=1 SV=1	2	2	1	1.10E+05	9.60E+04	9.01E+04
Q9WUZ7	SH3BG_MOUSE	SH3 domain-binding glutamic acid-rich protein OS=Mus musculus GN=Sh3bgr PE=2 SV=1	1	13	1	3.60E+05	2.87E+05	2.81E+05
P62627	DLRB1_MOUSE	Dynein light chain roadblock-type 1 OS=Mus musculus GN=Dynlrb1 PE=1 SV=3	2	2	1	2.81E+05	2.73E+05	1.87E+05
P31230	AIMP1_MOUSE	Aminoacyl tRNA synthetase complex-interacting multifunctional protein 1 OS=Mus musculus GN=Aimp1 PE=1 SV=2	1	3	0.98	9.25E+04	7.73E+04	5.52E+04

Q99JX3	GORS2_MOUSE	Golgi reassembly-stacking protein 2 OS=Mus musculus GN=Gorasp2 PE=1 SV=3	1	2	0.98	1.50E+04	1.97E+04	1.82E+04	1.12E+04	1.99E+04	1.50E+04	1.18E+04	2.35E+04	1.57E+04	2.00E+04	2.50E+04	1.60E+04	3.08E+04	0.222	0.158	0.091	0.189	0.154	0.183	0.181	0.109	0.164	0.103	0.052	0.215	0.065	3	2	2	GORS2_MOUSE, SP:Q99JX3	SP|Q99JX3|GORS2_MOUSE
Q9D7N9	APMAP_MOUSE	Adipocyte plasma membrane-associated protein OS=Mus musculus GN=Apmap PE=1 SV=1	1	1	0.99	8.72E+04	6.68E+04	4.33E+04	5.06E+04	5.25E+04	5.00E+04	4.24E+04	7.98E+04	6.40E+04	5.36E+04	4.23E+04	8.18E+04	5.72E+04	0.032	0.033	0.019	0.03	0.049	0.049	0.042	0.012	0.024	0.02	0.017	0.037	0.02	4	1	1	APMAP_MOUSE, SP:Q9D7N9	SP|Q9D7N9|APMAP_MOUSE
O88487	DC1I2_MOUSE	Cytoplasmic dynein 1 intermediate chain 2 OS=Mus musculus GN=Dync1i2 PE=2 SV=1	1	3	0.99	4.53E+04	3.28E+04	1.07E+04	1.70E+04	2.08E+04	2.23E+04	2.08E+04	2.75E+04	3.11E+04	2.85E+04	2.02E+04	2.62E+04	1.67E+04	0.092	0.099	0.15	0.138	0.149	0.136	0.113	0.092	0.085	0.067	0.073	0.156	0.133	3	3	3	DC1I2_MOUSE, SP:O88487	SP|O88487|DC1I2_MOUSE
P55264	ADK_MOUSE	Adenosine kinase OS=Mus musculus GN=Adk PE=1 SV=2	7	13	1	8.67E+05	7.40E+05	7.58E+05	6.94E+05	7.24E+05	7.47E+05	5.82E+05	7.73E+05	7.29E+05	8.20E+05	6.15E+05	6.74E+05	6.96E+05	2.95E-09	4.53E-10	2.34E-18	1.77E-12	4.04E-10	8.63E-11	7.05E-11	8.87E-13	1.49E-11	3.98E-16	4.21E-16	1.47E-07	3.60E-13	3, 4, 4, 5, 6, 7	1, 11, 13, 3, 4, 5, 9	1, 11, 13, 3, 4, 5, 9	ADK_MOUSE, SP:P55264	SP|P55264|ADK_MOUSE
P28474	ADHX_MOUSE	Alcohol dehydrogenase class-3 OS=Mus musculus GN=Adh5 PE=1 SV=3	2	8	1	2.64E+05	2.58E+05	2.95E+05	2.76E+05	2.98E+05	2.46E+05	2.20E+05	2.53E+05	2.91E+05	2.78E+05	2.97E+05	2.88E+05	2.37E+05	6.49E-04	1.46E-04	3.25E-08	5.74E-06	3.93E-05	1.22E-04	3.98E-05	2.68E-05	1.31E-05	1.30E-06	1.14E-08	3.99E-04	1.37E-05	4, 6	8	8	ADHX_MOUSE, SP:P28474	SP|P28474|ADHX_MOUSE
O89023	TPP1_MOUSE	Tripeptidyl-peptidase 1 OS=Mus musculus GN=Tpp1 PE=1 SV=2	1	5	0.99	6.15E+04	7.61E+04	5.26E+04	8.39E+04	6.24E+04	6.49E+04	6.73E+04	5.18E+04	4.62E+04	4.06E+04	5.79E+04	4.87E+04	4.63E+04	0.064	0.026	0.012	0.009	0.038	0.031	0.016	0.037	0.051	0.04	0.009	0.087	0.035	4	5	5	TPP1_MOUSE, SP:O89023	SP|O89023|TPP1_MOUSE
P49443	PPM1B_MOUSE	Protein phosphatase 1A OS=Mus musculus GN=Ppm1a PE=1 SV=1	1	9	0.99	8.57E+04	7.27E+04	3.48E+04	4.60E+04	4.82E+04	4.78E+04	3.34E+04	6.95E+04	5.37E+04	5.81E+04	4.70E+04	4.62E+04	4.99E+04	0.033	0.027	0.031	0.036	0.056	0.053	0.062	0.017	0.035	0.016	0.013	0.089	0.027	3	9	9	PPM1B_MOUSE, SP:P36993	SP|P36993|PPM1B_MOUSE,SP|P49443|PPM1A_MOUSE
Q9CQ60	6PGL_MOUSE	6-phosphogluconolactonase OS=Mus musculus GN=Pgls PE=2 SV=1	5	13	1	5.18E+05	4.16E+05	3.41E+05	4.03E+05	4.45E+05	4.43E+05	3.56E+05	4.35E+05	4.55E+05	3.52E+05	4.09E+05	5.36E+05	3.65E+05	3.51E-06	2.30E-06	3.60E-09	6.14E-08	7.83E-07	4.57E-07	2.80E-07	6.46E-08	7.99E-08	6.81E-08	2.89E-11	2.45E-06	1.08E-07	2, 3, 5	1, 13, 5, 7	1, 13, 5, 7	6PGL_MOUSE, SP:Q9CQ60	SP|Q9CQ60|6PGL_MOUSE
Q9CZ30	OLA1_MOUSE	Obg-like ATPase 1 OS=Mus musculus GN=Ola1 PE=1 SV=1	1	4	1	2.08E+04	1.61E+04	2.34E+04	2.98E+04	2.12E+04	2.81E+04	3.26E+04	3.74E+04	3.38E+04	2.70E+04	2.94E+04	1.07E+04	2.25E+04	0.183	0.182	0.063	0.074	0.145	0.107	0.064	0.06	0.076	0.071	0.039	0.258	0.098	4	4	4	OLA1_MOUSE, SP:Q9CZ30	SP|Q9CZ30|OLA1_MOUSE
Q9WUK2	IF4H_MOUSE	Eukaryotic translation initiation factor 4H OS=Mus musculus GN=Eif4h PE=1 SV=3	3	12	1	3.67E+05	3.11E+05	2.52E+05	2.34E+05	2.76E+05	2.60E+05	2.11E+05	3.21E+05	2.86E+05	2.50E+05	2.26E+05	2.68E+05	2.31E+05	7.71E-05	3.64E-05	3.00E-07	2.71E-05	7.39E-05	8.40E-05	5.70E-05	2.96E-06	1.58E-05	4.29E-06	5.71E-07	6.07E-04	1.78E-05	2, 3, 6	11, 12, 8	11, 12, 8	IF4H_MOUSE, SP:Q9WUK2	SP|Q9WUK2|IF4H_MOUSE
Q9R0Q6	ARC1A_MOUSE	Actin-related protein 2/3 complex subunit 1A OS=Mus musculus GN=Arpc1a PE=1 SV=1	1	2	0.99	3.02E+03	1.25E+04	1.95E+04	1.54E+04	1.07E+04	1.50E+04	1.27E+04	1.50E+04	1.22E+04	2.64E+04	1.91E+04	8.41E+03	2.50E+04	0.365	0.212	0.081	0.149	0.227	0.182	0.171	0.162	0.194	0.073	0.077	0.283	0.086	3	2	2	ARC1A_MOUSE, SP:Q9R0Q6	SP|Q9R0Q6|ARC1A_MOUSE
Q9QUR7	PIN1_MOUSE	Peptidyl-prolyl cis-trans isomerase NIMA-interacting 1 OS=Mus musculus GN=Pin1 PE=1 SV=1	1	3	0.99	2.90E+04	6.55E+04	4.33E+04	4.70E+04	4.65E+04	5.26E+04	3.96E+04	5.16E+04	7.05E+04	7.01E+04	7.28E+04	5.70E+04	7.28E+04	0.144	0.034	0.021	0.035	0.06	0.045	0.049	0.035	0.019	0.009	0.003	0.068	0.01	3	3	3	PIN1_MOUSE, SP:Q9QUR7	SP|Q9QUR7|PIN1_MOUSE
O70310	NMT1_MOUSE	Glycylpeptide N-tetradecanoyltransferase 1 OS=Mus musculus GN=Nmt1 PE=1 SV=1	1	1	0.99	1.91E+04	7.88E+03	1.49E+04	1.51E+04	1.45E+04	9.81E+03	1.26E+04	1.38E+04	1.10E+04	1.13E+04	1.20E+04	1.68E+04	1.74E+04	0.194	0.264	0.112	0.153	0.191	0.232	0.172	0.173	0.206	0.172	0.13	0.209	0.128	2	1	1	NMT1_MOUSE, SP:O70310	SP|O70310|NMT1_MOUSE
Q922B2	SYDC_MOUSE	Aspartyl-tRNA synthetase, cytoplasmic OS=Mus musculus GN=Dars PE=2 SV=1	2	4	1	6.53E+04	6.42E+04	6.78E+04	5.80E+04	5.29E+04	6.42E+04	6.57E+04	7.33E+04	7.58E+04	7.74E+04	6.32E+04	4.71E+04	8.21E+04	0.055	0.035	0.005	0.022	0.048	0.03	0.015	0.015	0.016	0.007	0.005	0.087	0.007	3	3, 4	3, 4	SYDC_MOUSE, SP:Q922B2	SP|Q922B2|SYDC_MOUSE
Q9JLR9	HIG1A_MOUSE	HIG1 domain family member 1A OS=Mus musculus GN=Higd1a PE=2 SV=1	1	12	0.99	2.72E+05	1.84E+05	1.37E+05	1.81E+05	2.38E+05	1.69E+05	1.27E+05	1.88E+05	1.53E+05	1.49E+05	1.01E+05	1.51E+05	1.18E+05	5.62E-04	0.001	1.15E-04	1.93E-04	2.18E-04	0.001	0.001	2.55E-04	0.001	2.94E-04	5.47E-04	0.008	0.002	5	12	12	HIG1A_MOUSE, SP:Q9JLR9	SP|Q9JLR9|HIG1A_MOUSE
Q9D819	IPYR_MOUSE	Inorganic pyrophosphatase OS=Mus musculus GN=Ppa1 PE=1 SV=1	3	5	1	2.56E+05	2.17E+05	2.57E+05	2.04E+05	2.57E+05	2.43E+05	2.26E+05	2.37E+05	2.79E+05	3.09E+05	2.75E+05	3.46E+05	2.72E+05	7.73E-04	4.45E-04	2.26E-07	7.90E-05	1.26E-04	1.32E-04	3.20E-05	4.67E-05	1.95E-05	3.81E-07	3.77E-08	1.21E-04	3.70E-06	3, 4, 6	2, 5	2, 5	IPYR_MOUSE, SP:Q9D819	SP|Q9D819|IPYR_MOUSE
Q8BK64	AHSA1_MOUSE	Activator of 90 kDa heat shock protein ATPase homolog 1 OS=Mus musculus GN=Ahsa1 PE=2 SV=2	1	1	0.99	1.52E+04	2.80E+04	5.19E+04	3.52E+04	3.08E+04	3.80E+04	3.32E+04	3.75E+04	2.67E+04	2.61E+04	2.94E+04	2.43E+04	2.90E+04	0.22	0.116	0.011	0.058	0.102	0.074	0.062	0.06	0.101	0.074	0.038	0.165	0.071	3	1	1	AHSA1_MOUSE, SP:Q8BK64	SP|Q8BK64|AHSA1_MOUSE
O54734	OST48_MOUSE	Dolichyl-diphosphooligosaccharide--protein glycosyltransferase 48 kDa subunit OS=Mus musculus GN=Ddost PE=1 SV=1	1	2	0.99	1.58E+04	2.55E+04	1.78E+04	1.77E+04	1.52E+04	1.69E+04	1.92E+04	2.86E+04	1.86E+04	2.61E+04	3.01E+04	1.83E+04	3.22E+04	0.215	0.127	0.091	0.133	0.186	0.167	0.121	0.087	0.144	0.074	0.037	0.199	0.061	2	2	2	OST48_MOUSE, SP:O54734	SP|O54734|OST48_MOUSE
Q99K48	NONO_MOUSE	Non-POU domain-containing octamer-binding protein OS=Mus musculus GN=Nono PE=1 SV=3	2	7	1	1.18E+05	9.44E+04	5.93E+04	8.10E+04	9.81E+04	5.25E+04	6.26E+04	9.62E+04	7.69E+04	7.69E+04	6.75E+04	6.20E+04	6.31E+04	0.016	0.014	0.007	0.009	0.012	0.045	0.017	0.006	0.015	0.007	0.004	0.06	0.015	3, 4	1, 7	1, 7	NONO_MOUSE, SP:Q99K48	SP|Q99K48|NONO_MOUSE
P68037	UB2L3_MOUSE	Ubiquitin-conjugating enzyme E2 L3 OS=Mus musculus GN=Ube2l3 PE=2 SV=1	4	12	1	4.54E+05	3.66E+05	3.32E+05	4.28E+05	3.14E+05	3.72E+05	3.30E+05	3.75E+05	4.45E+05	3.93E+05	3.60E+05	4.70E+05	4.02E+05	1.25E-05	8.12E-06	4.93E-09	2.37E-08	2.54E-05	3.23E-06	6.84E-07	4.37E-07	1.02E-07	1.16E-08	3.64E-10	9.26E-06	2.46E-08	2, 3, 5, 6	10, 12, 5, 8	10, 12, 5, 8	UB2L3_MOUSE, SP:P68037	SP|P68037|UB2L3_MOUSE
P97372	PSME2_MOUSE	Proteasome activator complex subunit 2 OS=Mus musculus GN=Psme2 PE=2 SV=4	2	1	1	8.45E+04	5.49E+04	7.11E+04	6.53E+04	8.79E+04	6.34E+04	5.90E+04	6.73E+04	9.00E+04	7.91E+04	6.03E+04	8.69E+04	5.32E+04	0.035	0.048	0.004	0.017	0.016	0.032	0.021	0.019	0.01	0.006	0.006	0.033	0.024	3, 4	1	1	PSME2_MOUSE, SP:P97372	SP|P97372|PSME2_MOUSE
Q9CQ89	CUTA_MOUSE	Protein CutA OS=Mus musculus GN=Cuta PE=2 SV=3	1	1	0.98	5.88E+04	6.01E+04	4.19E+04	5.33E+04	5.18E+04	5.50E+04	4.03E+04	3.89E+04	4.65E+04	3.73E+04	5.76E+04	5.09E+04	4.57E+04	0.065	0.041	0.021	0.027	0.05	0.042	0.046	0.057	0.047	0.043	0.007	0.08	0.033	2	1	1	CUTA_MOUSE, SP:Q9CQ89	SP|Q9CQ89|CUTA_MOUSE
P42227	STAT3_MOUSE	Signal transducer and activator of transcription 3 OS=Mus musculus GN=Stat3 PE=1 SV=2	1	3	1	3.68E+04	2.04E+04	1.55E+04	1.92E+04	3.19E+04	2.08E+04	1.52E+04	4.14E+04	2.33E+04	2.57E+04	3.38E+04	2.92E+04	3.40E+04	0.115	0.154	0.113	0.123	0.098	0.142	0.149	0.051	0.117	0.076	0.029	0.142	0.056	3	3	3	STAT3_MOUSE, SP:P42227	SP|P42227|STAT3_MOUSE
P17047	LAMP2_MOUSE	Lysosome-associated membrane glycoprotein 2 OS=Mus musculus GN=Lamp2 PE=2 SV=1	2	2	1	3.57E+04	3.91E+04	5.11E+04	3.46E+04	3.68E+04	4.08E+04	5.13E+04	4.71E+04	5.06E+04	5.31E+04	4.38E+04	2.26E+04	6.55E+04	0.118	0.079	0.012	0.06	0.082	0.067	0.028	0.04	0.04	0.02	0.016	0.174	0.014	2	1, 2	1, 2	LAMP2_MOUSE, SP:P17047	SP|P17047|LAMP2_MOUSE
P05977	MYL1_MOUSE	Myosin light chain 1/3, skeletal muscle isoform OS=Mus musculus GN=Myl1 PE=1 SV=2	4	10	1	1.11E+05	1.02E+05	4.88E+04	5.95E+04	6.34E+04	6.96E+04	6.35E+04	2.04E+05	2.62E+05	2.42E+05	1.67E+05	2.02E+05	1.81E+05	0.019	0.011	0.014	0.021	0.034	0.026	0.017	1.40E-04	3.39E-05	5.84E-06	1.31E-05	0.002	1.25E-04	2, 3, 4	10, 5, 6, 8	10, 5, 6, 8	MYL1_MOUSE, SP:P05977	SP|P05977|MYL1_MOUSE
Q99MR9	PPR3A_MOUSE	Protein phosphatase 1 regulatory subunit 3A OS=Mus musculus GN=Ppp1r3a PE=1 SV=1	1	4	1	2.99E+04	1.74E+04	7.02E+03	1.54E+04	1.53E+04	2.20E+04	1.07E+04	1.59E+04	1.37E+04	1.48E+04	1.15E+04	1.83E+04	1.31E+04	0.139	0.173	0.206	0.149	0.186	0.136	0.192	0.155	0.181	0.139	0.135	0.199	0.162	2	4	4	PPR3A_MOUSE, SP:Q99MR9	SP|Q99MR9|PPR3A_MOUSE
P61750	ARF4_MOUSE	ADP-ribosylation factor 4 OS=Mus musculus GN=Arf4 PE=2 SV=2	1	5	1	2.78E+04	3.46E+04	2.95E+04	2.04E+04	3.35E+04	3.46E+04	1.19E+04	6.21E+04	6.54E+04	2.24E+04	1.69E+04	1.66E+04	2.04E+04	0.148	0.092	0.043	0.115	0.093	0.084	0.179	0.023	0.023	0.09	0.09	0.21	0.109	4	5	5	ARF4_MOUSE, SP:P61750	SP|P61750|ARF4_MOUSE
Q91V12	BACH_MOUSE	Cytosolic acyl coenzyme A thioester hydrolase OS=Mus musculus GN=Acot7 PE=1 SV=2	2	6	1	4.24E+04	5.99E+04	8.39E+04	6.90E+04	6.51E+04	5.50E+04	5.37E+04	4.75E+04	4.49E+04	6.09E+04	6.33E+04	3.94E+04	5.69E+04	0.098	0.04	0.002	0.014	0.032	0.041	0.026	0.04	0.049	0.014	0.005	0.107	0.02	2	3, 6	3, 6	BACH_MOUSE, SP:Q91V12	SP|Q91V12|BACH_MOUSE
P58242	ASM3B_MOUSE	Acid sphingomyelinase-like phosphodiesterase 3b OS=Mus musculus GN=Smpdl3b PE=1 SV=1	1	13	0.85	1.94E+06	2.00E+06	1.49E+06	2.10E+06	1.75E+06	2.30E+06	2.18E+06	2.14E+06	1.89E+06	1.90E+06	2.42E+06	2.13E+06	2.37E+06	5.19E-18	4.16E-23	1.52E-32	3.10E-32	3.56E-21	2.44E-28	7.41E-34	1.17E-30	6.17E-26	4.78E-33	0	1.40E-19	6.84E-38	6	4, 8	13	ASM3B_MOUSE, SP:P58242	SP|P58242|ASM3B_MOUSE
Q99L47	F10A1_MOUSE	Hsc70-interacting protein OS=Mus musculus GN=St13 PE=2 SV=1	2	10	1	9.34E+04	8.54E+04	7.49E+04	7.67E+04	1.04E+05	8.51E+04	8.66E+04	9.65E+04	8.27E+04	8.43E+04	9.83E+04	1.12E+05	1.09E+05	0.028	0.019	0.003	0.01	0.01	0.015	0.007	0.006	0.012	0.005	6.30E-04	0.018	0.002	2, 3	1, 10	1, 10	F10A1_MOUSE, SP:Q99L47	SP|Q99L47|F10A1_MOUSE
Q4VAA2	CDV3_MOUSE	Protein CDV3 OS=Mus musculus GN=Cdv3 PE=1 SV=2	3	6	1	1.21E+05	1.14E+05	4.59E+04	8.90E+04	1.07E+05	1.10E+05	8.54E+04	1.06E+05	1.30E+05	1.19E+05	1.21E+05	1.05E+05	1.44E+05	0.015	0.008	0.016	0.006	0.009	0.007	0.007	0.005	0.002	0.001	1.77E-04	0.021	5.52E-04	2, 4, 5	1, 6	1, 6	CDV3_MOUSE, SP:Q4VAA2	SP|Q4VAA2|CDV3_MOUSE
O35343	IMA4_MOUSE	Importin subunit alpha-4 OS=Mus musculus GN=Kpna4 PE=2 SV=1	1	8	1	3.93E+04	3.30E+04	5.17E+04	3.33E+04	3.08E+04	3.61E+04	2.17E+04	5.47E+04	6.14E+04	1.25E+04	3.04E+04	2.35E+04	3.34E+04	0.107	0.098	0.012	0.063	0.102	0.08	0.107	0.03	0.027	0.159	0.036	0.168	0.058	4	8	8	IMA4_MOUSE, SP:O35343	SP|O35343|IMA4_MOUSE
P60766	CDC42_MOUSE	Cell division control protein 42 homolog OS=Mus musculus GN=Cdc42 PE=1 SV=1	2	7	1	7.01E+04	4.76E+04	8.83E+04	7.15E+04	8.63E+04	6.91E+04	8.67E+04	1.17E+05	1.11E+05	1.04E+05	1.12E+05	6.43E+04	1.18E+05	0.049	0.06	0.002	0.013	0.017	0.026	0.007	0.003	0.005	0.002	2.95E-04	0.056	0.002	2, 4	4, 7	4, 7	CDC42_MOUSE, SP:P60766	SP|P60766|CDC42_MOUSE
P62843	RS15_MOUSE	40S ribosomal protein S15 OS=Mus musculus GN=Rps15 PE=2 SV=2	1	8	0.99	8.62E+04	6.28E+04	1.18E+05	7.19E+04	6.52E+04	1.23E+05	3.87E+04	2.89E+05	9.17E+04	6.49E+04	5.90E+04	5.04E+04	3.80E+04	0.033	0.037	3.11E-04	0.012	0.032	0.005	0.049	8.30E-06	0.009	0.011	0.006	0.08	0.046	6	8	8	RS15_MOUSE, SP:P62843	SP|P62843|RS15_MOUSE
P15626	GSTM2_MOUSE	Glutathione S-transferase Mu 2 OS=Mus musculus GN=Gstm2 PE=1 SV=2	3	13	1	2.79E+05	3.77E+05	4.68E+05	4.90E+05	3.53E+05	4.09E+05	4.45E+05	3.51E+05	2.77E+05	3.47E+05	3.56E+05	2.58E+05	3.24E+05	4.84E-04	6.44E-06	5.92E-12	2.96E-09	8.96E-06	1.19E-06	1.13E-08	1.04E-06	2.08E-05	7.86E-08	4.90E-10	7.57E-04	5.10E-07	2, 3, 5	13, 6, 8	13, 6, 8	GSTM2_MOUSE, SP:P15626	SP|P15626|GSTM2_MOUSE
Q9CQE8	CN166_MOUSE	UPF0568 protein C14orf166 homolog OS=Mus musculus PE=2 SV=1	1	2	0.99	3.08E+04	1.81E+04	2.05E+04	2.87E+04	2.02E+04	1.60E+04	1.69E+04	2.53E+04	1.93E+04	2.13E+04	2.50E+04	1.97E+04	2.82E+04	0.136	0.169	0.076	0.078	0.152	0.175	0.137	0.1	0.14	0.097	0.053	0.19	0.074	3	2	2	CN166_MOUSE, SP:Q9CQE8	SP|Q9CQE8|CN166_MOUSE
P63073	IF4E_MOUSE	Eukaryotic translation initiation factor 4E OS=Mus musculus GN=Eif4e PE=1 SV=1	2	1	1	3.47E+04	3.17E+04	4.94E+04	4.04E+04	2.90E+04	3.09E+04	3.84E+04	4.81E+04	3.54E+04	4.15E+04	4.55E+04	2.90E+04	4.94E+04	0.122	0.102	0.014	0.046	0.109	0.097	0.049	0.039	0.071	0.035	0.014	0.144	0.028	2, 3	1	1	IF4E_MOUSE, SP:P63073	SP|P63073|IF4E_MOUSE
P42669	PURA_MOUSE	Transcriptional activator protein Pur-alpha OS=Mus musculus GN=Pura PE=1 SV=1	2	12	1	2.00E+05	2.16E+05	1.74E+05	2.00E+05	1.99E+05	1.89E+05	1.71E+05	1.58E+05	1.55E+05	1.69E+05	2.10E+05	1.95E+05	2.54E+05	0.003	4.68E-04	2.09E-05	9.52E-05	6.47E-04	6.73E-04	2.58E-04	7.20E-04	0.001	1.33E-04	1.52E-06	0.003	7.73E-06	3, 4	12, 5	12, 5	PURA_MOUSE, SP:P42669	SP|P42669|PURA_MOUSE
Q9QZH6	ECSIT_MOUSE	Evolutionarily conserved signaling intermediate in Toll pathway, mitochondrial OS=Mus musculus GN=Ecsit PE=1 SV=2	1	2	0.99	4.85E+04	2.99E+04	4.31E+04	3.70E+04	4.14E+04	2.61E+04	2.01E+04	3.75E+04	3.17E+04	2.27E+04	2.37E+04	3.51E+04	2.65E+04	0.084	0.109	0.02	0.054	0.071	0.117	0.117	0.06	0.083	0.09	0.057	0.121	0.08	4	2	2	ECSIT_MOUSE, SP:Q9QZH6	SP|Q9QZH6|ECSIT_MOUSE
Q9QVP4	MLRA_MOUSE	Myosin regulatory light chain 2, atrial isoform OS=Mus musculus GN=Myl7 PE=2 SV=1	2	2	1	1.25E+05	4.67E+05	1.28E+05	1.17E+05	7.77E+04	1.53E+05	8.82E+04	7.57E+04	6.71E+04	3.46E+04	5.83E+04	1.38E+05	9.72E+04	0.013	5.86E-07	1.76E-04	0.002	0.022	0.002	0.006	0.014	0.022	0.049	0.007	0.01	0.004	3, 5	1, 2	1, 2	MLRA_MOUSE, SP:Q9QVP4	SP|Q9QVP4|MLRA_MOUSE
Q9DCB8	ISCA2_MOUSE	Iron-sulfur cluster assembly 2 homolog, mitochondrial OS=Mus musculus GN=Isca2 PE=2 SV=2	1	7	0.99	3.98E+04	4.57E+04	3.84E+04	4.02E+04	4.49E+04	4.21E+04	4.64E+04	3.89E+04	3.80E+04	2.96E+04	3.84E+04	4.85E+04	3.99E+04	0.107	0.065	0.026	0.048	0.064	0.065	0.036	0.058	0.065	0.064	0.023	0.085	0.044	2	7	7	ISCA2_MOUSE, SP:Q9DCB8	SP|Q9DCB8|ISCA2_MOUSE
Q922J3	CLIP1_MOUSE	CAP-Gly domain-containing linker protein 1 OS=Mus musculus GN=Clip1 PE=1 SV=1	1	1	0.99	9.68E+03	1.03E+04	1.39E+04	1.72E+04	1.54E+04	1.77E+04	1.25E+04	1.75E+04	2.03E+04	1.30E+04	2.44E+04	9.11E+03	2.02E+04	0.269	0.235	0.121	0.136	0.185	0.162	0.174	0.144	0.133	0.155	0.054	0.275	0.111	3	1	1	CLIP1_MOUSE, SP:Q922J3	SP|Q922J3|CLIP1_MOUSE
Q64010	CRK_MOUSE	Adapter molecule crk OS=Mus musculus GN=Crk PE=1 SV=1	1	3	0.99	1.25E+05	1.49E+05	1.44E+05	1.45E+05	1.03E+05	8.42E+04	8.05E+04	1.37E+05	1.12E+05	8.35E+04	7.11E+04	8.54E+04	6.37E+04	0.013	0.003	8.12E-05	7.41E-04	0.01	0.016	0.008	0.001	0.005	0.005	0.003	0.034	0.015	3	3	3	CRK_MOUSE, SP:Q64010	SP|Q64010|CRK_MOUSE
Q9JI75	NQO2_MOUSE	Ribosyldihydronicotinamide dehydrogenase [quinone] OS=Mus musculus GN=Nqo2 PE=2 SV=3	2	9	1	2.08E+05	1.70E+05	1.19E+05	1.87E+05	1.67E+05	1.66E+05	1.40E+05	1.65E+05	1.05E+05	1.14E+05	1.25E+05	1.14E+05	1.13E+05	0.002	0.002	2.93E-04	1.47E-04	0.002	0.001	8.21E-04	5.44E-04	0.006	0.001	1.35E-04	0.018	0.002	3, 5	9	9	NQO2_MOUSE, SP:Q9JI75	SP|Q9JI75|NQO2_MOUSE
P43024	CX6A1_MOUSE	Cytochrome c oxidase subunit 6A1, mitochondrial OS=Mus musculus GN=Cox6a1 PE=1 SV=2	1	13	1	3.18E+05	4.43E+05	2.90E+05	3.19E+05	2.96E+05	3.42E+05	1.49E+05	4.42E+05	3.42E+05	2.74E+05	1.58E+05	2.24E+05	1.94E+05	2.12E-04	1.10E-06	4.06E-08	1.20E-06	4.22E-05	7.73E-06	5.83E-04	4.99E-08	2.73E-06	1.56E-06	2.20E-05	0.002	7.59E-05	5	11, 13	11, 13	CX6A1_MOUSE, SP:P43024	SP|P43024|CX6A1_MOUSE
P05132	KAPCA_MOUSE	cAMP-dependent protein kinase catalytic subunit alpha OS=Mus musculus GN=Prkaca PE=1 SV=3	1	2	1	4.35E+04	3.02E+04	2.48E+04	3.47E+04	3.13E+04	2.59E+04	2.40E+04	3.18E+04	2.24E+04	3.23E+04	2.69E+04	2.80E+04	2.75E+04	0.168	0.175	0.203	0.182	0.133	0.176	0.191	0.149	0.209	0.13	0.145	0.199	0.138	4	2	2	KAPCA_MOUSE, SP:P05132	SP|P05132|KAPCA_MOUSE
Q91Z53	GRHPR_MOUSE	Glyoxylate reductase/hydroxypyruvate reductase OS=Mus musculus GN=Grhpr PE=1 SV=1	4	11	1	3.07E+05	2.80E+05	2.88E+05	2.99E+05	3.00E+05	2.39E+05	2.90E+05	2.58E+05	2.66E+05	2.41E+05	3.00E+05	2.87E+05	3.30E+05	2.64E-04	8.16E-05	5.22E-08	2.55E-06	3.94E-05	1.53E-04	3.12E-06	2.34E-05	3.03E-05	6.16E-06	1.02E-08	4.12E-04	3.97E-07	2, 2, 5, 3	1, 10, 6, 11, 8	1, 10, 11, 6, 8	GRHPR_MOUSE, SP:Q91Z53	SP|Q91Z53|GRHPR_MOUSE
Q8R1G2	CMBL_MOUSE	Carboxymethylenebutenolidase homolog OS=Mus musculus GN=Cmbl PE=2 SV=1	3	7	1	2.43E+05	2.38E+05	1.83E+05	2.51E+05	1.83E+05	2.27E+05	1.79E+05	2.92E+05	1.42E+05	1.94E+05	1.63E+05	1.49E+05	1.53E+05	0.001	2.61E-04	1.09E-05	1.51E-05	9.98E-04	2.18E-04	1.91E-04	7.73E-06	0.002	4.46E-05	1.79E-05	0.008	3.97E-04	2, 4, 5	1, 5, 7	1, 5, 7	CMBL_MOUSE, SP:Q8R1G2	SP|Q8R1G2|CMBL_MOUSE
Q8VEA4	MIA40_MOUSE	Mitochondrial intermembrane space import and assembly protein 40 OS=Mus musculus GN=Chchd4 PE=1 SV=1	1	1	0.99	4.45E+04	4.24E+04	7.71E+03	3.72E+04	3.58E+04	1.74E+04	4.01E+04	1.47E+04	3.55E+04	3.15E+04	7.46E+03	4.06E+04	3.23E+04	0.093	0.071	0.19	0.053	0.085	0.164	0.046	0.164	0.071	0.056	0.186	0.104	0.061	4	1	1	MIA40_MOUSE, SP:Q8VEA4	SP|Q8VEA4|MIA40_MOUSE
Q8R010	AIMP2_MOUSE	Aminoacyl tRNA synthetase complex-interacting multifunctional protein 2 OS=Mus musculus GN=Aimp2 PE=1 SV=1	1	1	0.99	3.92E+04	3.10E+04	2.32E+04	2.63E+04	3.40E+04	3.53E+04	2.39E+04	3.72E+04	3.55E+04	2.82E+04	2.80E+04	2.72E+04	3.16E+04	0.107	0.104	0.064	0.087	0.091	0.082	0.096	0.06	0.071	0.067	0.042	0.151	0.062	3	1	1	AIMP2_MOUSE, SP:Q8R010	SP|Q8R010|AIMP2_MOUSE
P56812	PDCD5_MOUSE	Programmed cell death protein 5 OS=Mus musculus GN=Pdcd5 PE=1 SV=3	1	7	0.99	3.38E+04	2.89E+04	2.42E+04	2.17E+04	1.79E+04	2.27E+04	2.03E+04	2.90E+04	2.29E+04	2.20E+04	2.30E+04	2.98E+04	3.88E+04	0.126	0.114	0.062	0.111	0.168	0.133	0.116	0.087	0.12	0.094	0.061	0.141	0.046	2	6	7	PDCD5_MOUSE, SP:P56812	SP|P56812|PDCD5_MOUSE
O88746	TOM1_MOUSE	Target of Myb protein 1 OS=Mus musculus GN=Tom1 PE=1 SV=1	4	2	1	1.98E+05	1.61E+05	1.50E+05	1.49E+05	1.32E+05	1.55E+05	1.44E+05	1.92E+05	2.02E+05	1.79E+05	1.74E+05	1.78E+05	1.66E+05	0.003	0.002	5.52E-05	6.10E-04	0.004	0.002	6.95E-04	2.16E-04	2.27E-04	8.04E-05	9.05E-06	0.004	2.24E-04	3, 5	1, 2	1, 2	TOM1_MOUSE, SP:O88746	SP|O88746|TOM1_MOUSE
P28656	NP1L1_MOUSE	Nucleosome assembly protein 1-like 1 OS=Mus musculus GN=Nap1l1 PE=1 SV=2	1	6	0.98	7.00E+04	5.99E+04	6.50E+04	5.92E+04	5.36E+04	6.10E+04	5.10E+04	6.71E+04	5.86E+04	5.73E+04	5.99E+04	6.27E+04	8.01E+04	0.049	0.041	0.006	0.021	0.047	0.034	0.029	0.019	0.03	0.017	0.006	0.059	0.008	3	6	6	NP1L1_MOUSE, SP:P28656	SP|P28656|NP1L1_MOUSE
O55028	BCKD_MOUSE	[3-methyl-2-oxobutanoate dehydrogenase [lipoamide]] kinase, mitochondrial OS=Mus musculus GN=Bckdk PE=1 SV=1	2	12	1	1.18E+05	1.22E+05	1.10E+05	8.86E+04	9.52E+04	1.02E+05	9.52E+04	1.02E+05	8.70E+04	9.64E+04	7.38E+04	9.37E+04	7.03E+04	0.016	0.007	4.63E-04	0.006	0.013	0.009	0.005	0.005	0.011	0.003	0.003	0.028	0.011	4, 6	12, 5	12, 5	BCKD_MOUSE, SP:O55028	SP|O55028|BCKD_MOUSE
O35215	DOPD_MOUSE	D-dopachrome decarboxylase OS=Mus musculus GN=Ddt PE=1 SV=3	3	12	1	2.43E+05	2.36E+05	2.66E+05	2.40E+05	3.01E+05	2.72E+05	2.90E+05	1.82E+05	2.32E+05	2.29E+05	3.06E+05	2.61E+05	2.24E+05	0.001	2.72E-04	1.40E-07	2.14E-05	3.72E-05	5.79E-05	3.02E-06	3.10E-04	8.90E-05	9.87E-06	7.05E-09	7.13E-04	2.39E-05	2, 3, 4	11, 12, 7	12, 7	DOPD_MOUSE, SP:O35215	SP|O35215|DOPD_MOUSE
P62849	RS24_MOUSE	40S ribosomal protein S24 OS=Mus musculus GN=Rps24 PE=1 SV=1	2	12	1	9.88E+04	7.55E+04	9.00E+04	9.42E+04	8.46E+04	9.49E+04	6.82E+04	1.34E+05	9.96E+04	8.64E+04	1.33E+05	1.04E+05	8.47E+04	0.025	0.025	0.002	0.005	0.018	0.011	0.014	0.002	0.007	0.004	9.40E-05	0.022	0.006	3, 4	12, 6	12, 6	RS24_MOUSE, SP:P62849	SP|P62849|RS24_MOUSE
P62267	RS23_MOUSE	40S ribosomal protein S23 OS=Mus musculus GN=Rps23 PE=2 SV=3	1	7	0.99	3.16E+04	6.03E+04	5.96E+04	4.84E+04	5.28E+04	4.93E+04	6.11E+04	7.66E+04	5.00E+04	4.57E+04	5.37E+04	3.38E+04	5.84E+04	0.133	0.04	0.008	0.033	0.048	0.05	0.019	0.013	0.041	0.028	0.009	0.125	0.019	3	7	7	RS23_MOUSE, SP:P62267	SP|P62267|RS23_MOUSE
Q02357	ANK1_MOUSE	Ankyrin-1 OS=Mus musculus GN=Ank1 PE=1 SV=2	1	10	0.98	1.34E+05	1.15E+05	3.87E+05	3.07E+05	4.89E+04	2.00E+05	3.71E+05	1.46E+05	1.24E+05	5.27E+05	1.03E+05	2.36E+04	1.48E+05	0.011	0.008	5.67E-10	2.19E-06	0.055	4.90E-04	2.02E-07	0.001	0.003	1.12E-10	4.84E-04	0.168	4.71E-04	4	10	10	ANK1_MOUSE, SP:Q02357	SP|Q02357|ANK1_MOUSE
Q8R1Q8	DC1L1_MOUSE	Cytoplasmic dynein 1 light intermediate chain 1 OS=Mus musculus GN=Dync1li1 PE=1 SV=1	1	1	1	3.32E+04	3.97E+04	2.01E+04	2.70E+04	2.73E+04	2.19E+04	1.36E+04	4.58E+04	3.98E+04	2.44E+04	3.20E+04	3.65E+04	3.89E+04	0.127	0.077	0.078	0.084	0.116	0.136	0.163	0.043	0.06	0.081	0.033	0.116	0.045	3	1	1	DC1L1_MOUSE, SP:Q8R1Q8	SP|Q8R1Q8|DC1L1_MOUSE
Q91VK1	BZW2_MOUSE	Basic leucine zipper and W2 domain-containing protein 2 OS=Mus musculus GN=Bzw2 PE=1 SV=1	1	1	0.68	2.57E+04	2.51E+04	1.58E+04	1.84E+04	2.39E+04	1.84E+04	1.66E+04	1.39E+04	1.47E+04	1.27E+04	1.07E+04	2.38E+04	1.81E+04	0.162	0.133	0.111	0.135	0.135	0.163	0.144	0.176	0.175	0.164	0.155	0.17	0.128	2	1	1	BZW2_MOUSE, SP:Q91VK1	SP|Q91VK1|BZW2_MOUSE
Q99LT0	DPY30_MOUSE	Protein dpy-30 homolog OS=Mus musculus GN=Dpy30 PE=2 SV=1	1	3	0.99	4.63E+04	4.73E+04	3.25E+04	2.80E+04	3.62E+04	3.50E+04	1.88E+04	4.49E+04	4.45E+04	1.70E+04	1.83E+04	3.20E+04	2.29E+04	0.089	0.061	0.037	0.081	0.084	0.084	0.124	0.045	0.051	0.122	0.082	0.132	0.096	3	3	3	DPY30_MOUSE, SP:Q99LT0	SP|Q99LT0|DPY30_MOUSE
Q02248	CTNB1_MOUSE	Catenin beta-1 OS=Mus musculus GN=Ctnnb1 PE=1 SV=1	4	12	1	2.09E+05	1.36E+05	9.67E+04	1.37E+05	1.32E+05	1.41E+05	1.26E+05	1.70E+05	1.54E+05	1.58E+05	1.21E+05	1.76E+05	1.34E+05	0.002	0.004	1.00E-03	9.88E-04	0.004	0.003	0.001	4.62E-04	0.001	1.97E-04	1.79E-04	0.004	8.20E-04	2, 3, 5	1, 12, 3, 4	1, 12, 3, 4	CTNB1_MOUSE, SP:Q02248	SP|Q02248|CTNB1_MOUSE
Q9CZ04	CSN7A_MOUSE	COP9 signalosome complex subunit 7a OS=Mus musculus GN=Cops7a PE=1 SV=2	1	2	0.58	1.94E+04	1.57E+04	3.29E+04	2.93E+04	2.05E+04	3.21E+04	2.32E+04	4.74E+04	2.50E+04	2.01E+04	2.27E+04	1.78E+04	2.04E+04	0.191	0.186	0.035	0.076	0.15	0.092	0.1	0.04	0.109	0.103	0.061	0.202	0.109	4	1	2	CSN7A_MOUSE, SP:Q9CZ04	SP|Q9CZ04|CSN7A_MOUSE
O54950	AAKG1_MOUSE	5'-AMP-activated protein kinase subunit gamma-1 OS=Mus musculus GN=Prkag1 PE=2 SV=2	1	2	1	4.99E+04	4.49E+04	3.81E+04	4.20E+04	3.64E+04	5.39E+04	4.15E+04	5.18E+04	4.54E+04	1.56E+04	2.69E+04	3.23E+04	3.61E+04	0.084	0.068	0.029	0.045	0.088	0.045	0.045	0.035	0.05	0.138	0.049	0.133	0.053	3	2	2	AAKG1_MOUSE, SP:O54950	SP|O54950|AAKG1_MOUSE
Q9CQX8	RT36_MOUSE	28S ribosomal protein S36, mitochondrial OS=Mus musculus GN=Mrps36 PE=1 SV=1	2	13	1	1.16E+06	1.20E+06	8.65E+05	9.86E+05	1.10E+06	9.44E+05	9.80E+05	1.08E+06	8.16E+05	7.49E+05	8.92E+05	9.95E+05	7.24E+05	1.29E-11	7.12E-15	5.88E-20	1.77E-16	3.90E-14	6.54E-13	1.32E-16	1.34E-16	1.80E-12	1.42E-14	1.28E-21	3.37E-10	2.40E-13	3, 6	13, 7	13, 7	RT36_MOUSE, SP:Q9CQX8	SP|Q9CQX8|RT36_MOUSE
P98197	AT11A_MOUSE	Probable phospholipid-transporting ATPase IH OS=Mus musculus GN=Atp11a PE=2 SV=1	1	5	0.81	5.01E+04	4.03E+04	1.71E+04	2.48E+04	3.39E+04	3.08E+04	2.54E+04	2.78E+04	3.54E+04	4.50E+04	2.39E+04	4.60E+04	2.33E+04	0.08	0.076	0.095	0.094	0.091	0.097	0.09	0.09	0.071	0.029	0.055	0.09	0.094	3	5	5	AT11A_MOUSE, SP:P98197	SP|P98197|AT11A_MOUSE
Q9DCS3	MECR_MOUSE	Trans-2-enoyl-CoA reductase, mitochondrial OS=Mus musculus GN=Mecr PE=2 SV=2	1	6	0.99	1.76E+05	1.25E+05	1.04E+05	1.22E+05	1.22E+05	1.07E+05	1.24E+05	1.21E+05	9.06E+04	1.02E+05	1.35E+05	1.29E+05	1.05E+05	0.004	0.006	6.48E-04	0.002	0.006	0.008	0.002	0.003	0.01	0.002	8.42E-05	0.012	0.003	5	6	6	MECR_MOUSE, SP:Q9DCS3	SP|Q9DCS3|MECR_MOUSE
P61211	ARL1_MOUSE	ADP-ribosylation factor-like protein 1 OS=Mus musculus GN=Arl1 PE=2 SV=1	1	4	1	2.00E+04	2.34E+04	2.67E+04	2.01E+04	1.99E+04	1.19E+04	1.59E+04	2.48E+04	2.34E+04	1.81E+04	1.30E+04	2.42E+04	2.49E+04	0.188	0.138	0.051	0.117	0.154	0.21	0.144	0.103	0.116	0.115	0.12	0.165	0.087	3	4	4	ARL1_MOUSE, SP:P61211	SP|P61211|ARL1_MOUSE
Q91YS8	KCC1A_MOUSE	Calcium/calmodulin-dependent protein kinase type 1 OS=Mus musculus GN=Camk1 PE=1 SV=1	1	1	0.98	7.09E+03	1.21E+04	1.62E+04	1.82E+04	8.94E+03	9.58E+03	1.03E+04	1.35E+04	1.32E+04	1.09E+04	2.08E+04	3.28E+03	1.57E+04	0.299	0.216	0.102	0.129	0.247	0.234	0.196	0.175	0.184	0.175	0.069	0.36	0.14	2	1	1	KCC1A_MOUSE, SP:Q91YS8	SP|Q91YS8|KCC1A_MOUSE
Q6P1F6	2ABA_MOUSE	Serine/threonine-protein phosphatase 2A 55 kDa regulatory subunit B alpha isoform OS=Mus musculus GN=Ppp2r2a PE=1 SV=1	1	1	0.99	3.24E+04	2.93E+04	2.14E+04	1.80E+04	2.46E+04	2.11E+04	2.22E+04	2.47E+04	1.82E+04	2.64E+04	1.98E+04	2.31E+04	1.44E+04	0.13	0.112	0.072	0.131	0.129	0.142	0.105	0.104	0.147	0.074	0.075	0.171	0.151	2	1	1	2ABA_MOUSE, SP:Q6P1F6	SP|Q6P1F6|2ABA_MOUSE
Q9DBR7	MYPT1_MOUSE	Protein phosphatase 1 regulatory subunit 12A OS=Mus musculus GN=Ppp1r12a PE=1 SV=2	1	2	0.82	0.00E+00	9.38E+03	7.51E+03	1.07E+04	1.31E+04	1.26E+04	2.03E+04	2.40E+04	3.17E+03	8.86E+03	5.90E+03	9.79E+03	2.36E+04	0.5	0.244	0.193	0.193	0.203	0.202	0.115	0.106	0.33	0.2	0.214	0.268	0.092	3	2	2	MYPT1_MOUSE, SP:Q9DBR7	SP|Q9DBR7|MYPT1_MOUSE
Q99LD4	CSN1_MOUSE	COP9 signalosome complex subunit 1 OS=Mus musculus GN=Gps1 PE=1 SV=1	2	5	1	4.50E+04	4.83E+04	4.04E+04	4.42E+04	4.58E+04	4.17E+04	4.22E+04	5.21E+04	4.22E+04	5.13E+04	5.29E+04	3.47E+04	5.70E+04	0.103	0.073	0.045	0.052	0.075	0.082	0.057	0.043	0.074	0.037	0.021	0.139	0.038	2, 4	2, 4	2, 5	CSN1_MOUSE, SP:Q99LD4	SP|Q99LD4|CSN1_MOUSE
Q8VBW6	ULA1_MOUSE	NEDD8-activating enzyme E1 regulatory subunit OS=Mus musculus GN=Nae1 PE=2 SV=1	1	1	0.78	2.52E+04	1.66E+04	1.93E+04	1.65E+04	2.54E+04	1.60E+04	2.23E+04	1.87E+04	1.32E+04	1.72E+04	1.66E+04	1.69E+04	2.01E+04	0.161	0.182	0.083	0.142	0.126	0.175	0.105	0.138	0.187	0.124	0.094	0.209	0.114	2	1	1	ULA1_MOUSE, SP:Q8VBW6	SP|Q8VBW6|ULA1_MOUSE
Q5F285	CQ061_MOUSE	UPF0451 protein C17orf61 homolog OS=Mus musculus PE=2 SV=1	1	11	0.99	8.21E+04	1.02E+05	8.56E+04	1.17E+05	1.06E+05	1.04E+05	9.58E+04	7.77E+04	5.86E+04	7.01E+04	8.93E+04	9.27E+04	9.67E+04	0.037	0.012	0.002	0.002	0.009	0.009	0.005	0.013	0.03	0.009	0.001	0.029	0.004	4	11	11	CQ061_MOUSE, SP:Q5F285	SP|Q5F285|CQ061_MOUSE
Q8VCI5	PEX19_MOUSE	Peroxisomal biogenesis factor 19 OS=Mus musculus GN=Pex19 PE=1 SV=1	1	8	0.99	6.67E+04	7.21E+04	3.86E+04	5.26E+04	6.09E+04	4.82E+04	3.25E+04	6.36E+04	8.23E+04	4.59E+04	2.20E+04	3.86E+04	3.17E+04	0.053	0.028	0.025	0.027	0.037	0.052	0.064	0.021	0.013	0.028	0.063	0.109	0.062	6	8	8	PEX19_MOUSE, SP:Q8VCI5	SP|Q8VCI5|PEX19_MOUSE
O88783	FA5_MOUSE	Coagulation factor V OS=Mus musculus GN=F5 PE=1 SV=1	1	2	1	2.11E+04	2.71E+04	3.85E+04	2.05E+04	2.84E+04	2.66E+04	1.98E+04	3.93E+04	3.10E+04	1.11E+04	7.29E+03	1.30E+04	1.30E+04	0.181	0.12	0.025	0.115	0.111	0.113	0.118	0.055	0.085	0.172	0.189	0.238	0.162	4	2	2	FA5_MOUSE, SP:O88783	SP|O88783|FA5_MOUSE
Q8C878	UBA3_MOUSE	NEDD8-activating enzyme E1 catalytic subunit OS=Mus musculus GN=Uba3 PE=2 SV=1	1	1	0.83	3.59E+04	2.35E+04	3.57E+04	3.05E+04	3.11E+04	2.31E+04	2.10E+04	3.50E+04	2.24E+04	1.96E+04	3.15E+04	2.25E+04	2.92E+04	0.118	0.138	0.032	0.073	0.103	0.135	0.112	0.067	0.127	0.113	0.038	0.176	0.071	3	1	1	UBA3_MOUSE, SP:Q8C878	SP|Q8C878|UBA3_MOUSE
Q6ZQ73	CAND2_MOUSE	Cullin-associated NEDD8-dissociated protein 2 OS=Mus musculus GN=Cand2 PE=1 SV=2	5	10	1	2.99E+05	2.10E+05	2.31E+05	2.42E+05	2.13E+05	2.23E+05	2.02E+05	3.08E+05	2.79E+05	1.92E+05	1.66E+05	2.32E+05	2.11E+05	3.09E-04	5.41E-04	8.16E-07	1.91E-05	4.34E-04	2.39E-04	7.78E-05	4.01E-06	1.93E-05	4.89E-05	1.46E-05	0.001	3.97E-05	3, 3, 4	1, 2, 9, 10	1, 10, 9, 2	CAND2_MOUSE, SP:Q6ZQ73	SP|Q6ZQ73|CAND2_MOUSE
P48774	GSTM5_MOUSE	Glutathione S-transferase Mu 5 OS=Mus musculus GN=Gstm5 PE=1 SV=1	4	13	1	9.56E+04	9.17E+04	1.53E+05	1.31E+05	1.31E+05	1.36E+05	1.33E+05	1.04E+05	1.05E+05	1.15E+05	1.15E+05	7.37E+04	1.37E+05	0.026	0.015	4.64E-05	0.001	0.004	0.003	0.001	0.005	0.006	0.001	2.36E-04	0.045	7.11E-04	2, 3	13, 6, 7, 8	13, 6, 7, 8	GSTM5_MOUSE, SP:P48774	SP|P48774|GSTM5_MOUSE
Q921X9	PDIA5_MOUSE	Protein disulfide-isomerase A5 OS=Mus musculus GN=Pdia5 PE=2 SV=1	1	1	0.97	4.53E+03	2.90E+03	1.82E+05	4.61E+03	4.93E+04	2.22E+04	3.06E+04	4.97E+03	2.49E+04	4.23E+04	4.10E+04	5.01E+04	2.09E+04	0.338	0.352	1.11E-05	0.286	0.054	0.135	0.07	0.286	0.109	0.033	0.019	0.081	0.106	4	1	1	PDIA5_MOUSE, SP:Q921X9	SP|Q921X9|PDIA5_MOUSE
Q80X50	UBP2L_MOUSE	Ubiquitin-associated protein 2-like OS=Mus musculus GN=Ubap2l PE=1 SV=1	1	2	0.97	3.76E+03	4.52E+03	1.24E+04	6.65E+03	0.00E+00	1.36E+03	6.05E+03	7.88E+03	6.71E+03	9.01E+03	1.12E+04	2.12E+03	1.46E+04	0.35	0.315	0.132	0.247	0.5	0.392	0.256	0.237	0.261	0.198	0.138	0.387	0.148	2	2	2	UBP2L_MOUSE, SP:Q80X50	SP|Q80X50|UBP2L_MOUSE
Q3ULJ0	GPD1L_MOUSE	Glycerol-3-phosphate dehydrogenase 1-like protein OS=Mus musculus GN=Gpd1l PE=1 SV=2	1	12	1	9.96E+04	7.36E+04	5.34E+04	6.06E+04	6.09E+04	5.01E+04	5.75E+04	5.27E+04	4.83E+04	5.32E+04	4.75E+04	6.27E+04	3.99E+04	0.024	0.026	0.011	0.02	0.037	0.049	0.022	0.032	0.043	0.02	0.012	0.059	0.043	4	12	12	GPD1L_MOUSE, SP:Q3ULJ0	SP|Q3ULJ0|GPD1L_MOUSE
Q9WV35	ABEC2_MOUSE	Probable C->U-editing enzyme APOBEC-2 OS=Mus musculus GN=Apobec2 PE=1 SV=1	5	12	1	6.77E+05	7.10E+05	6.87E+05	8.33E+05	5.72E+05	5.79E+05	7.17E+05	8.71E+05	6.16E+05	6.10E+05	6.22E+05	5.41E+05	7.56E+05	1.36E-07	9.81E-10	9.15E-17	1.39E-14	2.36E-08	9.55E-09	5.81E-13	3.70E-14	5.04E-10	1.81E-12	3.23E-16	2.17E-06	4.25E-14	2, 3, 4, 4, 5	1, 11, 6, 12, 8	1, 11, 12, 6, 8	ABEC2_MOUSE, SP:Q9WV35	SP|Q9WV35|ABEC2_MOUSE
Q99P72	RTN4_MOUSE	Reticulon-4 OS=Mus musculus GN=Rtn4 PE=1 SV=2	1	1	1	6.63E+03	3.42E+03	6.21E+03	6.96E+03	4.95E+03	4.68E+03	7.52E+03	9.11E+03	1.86E+04	1.58E+04	1.51E+04	7.76E+03	3.58E+04	0.305	0.338	0.216	0.243	0.305	0.307	0.233	0.221	0.143	0.131	0.104	0.291	0.052	2	1	1	RTN4_MOUSE, SP:Q99P72	SP|Q99P72|RTN4_MOUSE
Q9DB05	SNAB_MOUSE	Alpha-soluble NSF attachment protein OS=Mus musculus GN=Napa PE=1 SV=1	1	2	0.98	2.81E+04	2.36E+04	2.23E+04	2.98E+04	2.14E+04	1.71E+04	2.43E+04	2.27E+04	1.91E+04	1.95E+04	2.79E+04	2.44E+04	2.97E+04	0.147	0.136	0.068	0.074	0.145	0.166	0.095	0.112	0.14	0.106	0.043	0.164	0.068	3	2	2	SNAB_MOUSE, SP:P28663	SP|P28663|SNAB_MOUSE,SP|Q9DB05|SNAA_MOUSE
O35643	AP1B1_MOUSE	AP-1 complex subunit beta-1 OS=Mus musculus GN=Ap1b1 PE=1 SV=1	1	2	1	6.45E+04	4.42E+04	5.35E+04	4.59E+04	5.24E+04	4.58E+04	3.78E+04	5.60E+04	5.10E+04	4.15E+04	4.89E+04	5.97E+04	5.30E+04	0.057	0.068	0.011	0.038	0.05	0.058	0.052	0.03	0.04	0.036	0.012	0.064	0.025	4	2	2	AP1B1_MOUSE, SP:O35643	SP|O35643|AP1B1_MOUSE
P97927	LAMA4_MOUSE	Laminin subunit alpha-4 OS=Mus musculus GN=Lama4 PE=1 SV=2	3	12	1	1.20E+05	1.07E+05	1.47E+05	1.12E+05	7.90E+04	8.73E+04	7.54E+04	1.09E+05	1.40E+05	1.15E+05	1.08E+05	1.02E+05	1.37E+05	0.015	0.01	8.00E-05	0.003	0.021	0.015	0.011	0.004	0.002	0.001	3.90E-04	0.023	7.72E-04	2, 3, 4	12, 2	12, 2	LAMA4_MOUSE, SP:P97927	SP|P97927|LAMA4_MOUSE
Q9JMH9	MY18A_MOUSE	Myosin-XVIIIa OS=Mus musculus GN=Myo18a PE=1 SV=2	1	1	0.99	1.47E+04	1.75E+04	2.18E+04	8.63E+03	1.35E+04	1.62E+04	5.75E+03	2.36E+04	1.76E+04	1.76E+04	2.62E+04	1.69E+04	9.12E+03	0.226	0.174	0.077	0.223	0.203	0.175	0.268	0.11	0.152	0.121	0.049	0.21	0.212	3	1	1	MY18A_MOUSE, SP:Q9JMH9	SP|Q9JMH9|MY18A_MOUSE
Q9CQM5	TXD17_MOUSE	Thioredoxin domain-containing protein 17 OS=Mus musculus GN=Txndc17 PE=1 SV=1	1	7	0.96	1.79E+04	3.02E+04	4.82E+04	3.09E+04	3.37E+04	3.81E+04	5.33E+04	3.95E+04	4.09E+04	2.05E+04	2.96E+04	2.92E+04	4.70E+04	0.201	0.107	0.014	0.07	0.092	0.074	0.026	0.055	0.057	0.1	0.038	0.142	0.031	3	7	7	TXD17_MOUSE, SP:Q9CQM5	SP|Q9CQM5|TXD17_MOUSE
P51125	ICAL_MOUSE	Calpastatin OS=Mus musculus GN=Cast PE=1 SV=2	1	2	0.99	3.22E+06	1.89E+06	7.91E+05	1.46E+06	9.06E+05	2.40E+06	1.92E+06	1.43E+06	1.96E+06	2.08E+06	1.56E+06	1.79E+06	1.64E+06	4.96E-27	1.52E-21	3.12E-18	1.18E-22	6.66E-12	4.40E-28	6.45E-29	6.74E-21	8.72E-26	5.65E-34	6.21E-34	2.04E-16	2.41E-26	6	2	2	ICAL_MOUSE, SP:P51125	SP|P51125|ICAL_MOUSE
Q9Z2Y8	PROSC_MOUSE	Proline synthetase co-transcribed bacterial homolog protein OS=Mus musculus GN=Prosc PE=1 SV=1	3	13	1	1.77E+05	1.84E+05	1.79E+05	2.05E+05	1.66E+05	1.64E+05	1.81E+05	1.58E+05	1.45E+05	1.65E+05	1.94E+05	1.63E+05	2.01E+05	0.004	0.001	1.22E-05	7.50E-05	0.002	0.001	1.73E-04	6.92E-04	0.002	1.47E-04	3.09E-06	0.006	5.63E-05	2, 3, 3, 4	13, 4, 5, 6	13, 4, 5, 6	PROSC_MOUSE, SP:Q9Z2Y8	SP|Q9Z2Y8|PROSC_MOUSE
Q64152	BTF3_MOUSE	Transcription factor BTF3 OS=Mus musculus GN=Btf3 PE=2 SV=3	1	13	0.99	1.97E+05	1.83E+05	1.69E+05	1.50E+05	1.30E+05	1.39E+05	1.02E+05	2.48E+05	1.81E+05	1.33E+05	1.25E+05	2.09E+05	1.17E+05	0.003	0.001	2.29E-05	6.33E-04	0.005	0.003	0.004	3.45E-05	4.75E-04	6.11E-04	1.60E-04	0.002	0.002	7	13	13	BTF3_MOUSE, SP:Q64152	SP|Q64152|BTF3_MOUSE
P61202	CSN2_MOUSE	COP9 signalosome complex subunit 2 OS=Mus musculus GN=Cops2 PE=1 SV=1	1	1	0.99	3.58E+04	4.05E+04	1.65E+04	2.43E+04	3.67E+04	2.37E+04	2.11E+04	3.94E+04	3.72E+04	2.00E+04	1.78E+04	2.43E+04	3.03E+04	0.118	0.075	0.099	0.096	0.083	0.127	0.11	0.055	0.066	0.103	0.085	0.164	0.067	4	1	1	CSN2_MOUSE, SP:P61202	SP|P61202|CSN2_MOUSE
Q921L3	TMCO1_MOUSE	Transmembrane and coiled-coil domain-containing protein 1 OS=Mus musculus GN=Tmco1 PE=2 SV=1	1	1	0.99	2.79E+04	1.84E+04	2.76E+04	2.59E+04	1.80E+04	1.58E+04	2.20E+04	2.32E+04	2.65E+04	3.30E+04	2.89E+04	1.31E+04	3.16E+04	0.148	0.167	0.05	0.09	0.166	0.176	0.106	0.11	0.102	0.053	0.04	0.237	0.063	3	1	1	TMCO1_MOUSE, SP:Q921L3	SP|Q921L3|TMCO1_MOUSE
Q91VM9	IPYR2_MOUSE	Inorganic pyrophosphatase 2, mitochondrial OS=Mus musculus GN=Ppa2 PE=2 SV=1	5	11	1	6.08E+05	7.27E+05	7.26E+05	6.88E+05	6.72E+05	6.89E+05	6.84E+05	6.19E+05	4.88E+05	5.10E+05	6.05E+05	5.90E+05	5.23E+05	5.46E-07	6.76E-10	1.47E-17	2.45E-12	1.72E-09	4.53E-10	2.02E-12	1.43E-10	2.71E-08	1.07E-10	9.42E-16	8.25E-07	2.66E-10	2, 3, 4	11, 4, 5, 9	11, 4, 5, 9	IPYR2_MOUSE, SP:Q91VM9	SP|Q91VM9|IPYR2_MOUSE
[illegible]	[illegible]	[illegible]	[illegible]	[illegible]	[illegible]	[illegible]	[illegible]	[illegible]	[illegible]	[illegible]	[illegible]	[illegible]	[illegible]	[illegible]	[illegible]	[illegible]	[illegible]	[illegible]	[illegible]	[illegible]	[illegible]	[illegible]	[illegible]	[illegible]	[illegible]	[illegible]	[illegible]	[illegible]	[illegible]	[illegible]	[illegible]	[illegible]	[illegible]	[illegible]	[illegible]	[illegible]

Q920Q8	NS1BP_MOUSE	Influenza virus NS1A-binding protein homolog OS=Mus musculus GN=Ivns1abp PE=1 SV=2	1	1	0.77	2.09E+04	1.78E+04	2.19E+04	1.78E+04	7.11E+04	3.82E+04	2.76E+04	2.05E+04	5.41E+04	4.07E+04	3.66E+04	6.81E+04	2.38E+04	0.183	0.17	0.069	0.132	0.027	0.074	0.081	0.125	0.035	0.036	0.024	0.051	0.092	2	1	1	NS1BP_MOUSE, SP:Q920Q8	SP|Q920Q8|NS1BP_MOUSE
P11352	GPX1_MOUSE	Glutathione peroxidase 1 OS=Mus musculus GN=Gpx1 PE=1 SV=2	2	5	1	5.74E+04	9.29E+04	6.60E+04	7.59E+04	6.74E+04	8.26E+04	6.12E+04	9.47E+04	7.15E+04	8.04E+04	9.80E+04	8.29E+04	1.03E+05	0.067	0.015	0.005	0.011	0.03	0.017	0.019	0.007	0.019	0.006	6.46E-04	0.036	0.003	4, 5	2, 5	3, 5	GPX1_MOUSE, SP:P11352	SP|P11352|GPX1_MOUSE
Q8CGB6	TENC1_MOUSE	Tensin-like C1 domain-containing phosphatase OS=Mus musculus GN=Tenc1 PE=1 SV=1	1	12	0.95	2.97E+05	4.58E+05	4.33E+05	4.19E+05	4.43E+05	3.24E+05	5.43E+05	3.71E+05	3.85E+05	3.29E+05	4.01E+05	3.94E+05	3.60E+05	3.44E-04	8.51E-07	5.35E-11	4.02E-08	9.17E-07	1.43E-05	4.84E-10	5.92E-07	8.03E-07	1.97E-07	7.41E-11	4.81E-05	1.54E-07	5	12	12	TENC1_MOUSE, SP:Q8CGB6	SP|Q8CGB6|TENC1_MOUSE
Q9D0F3	LMAN1_MOUSE	Protein ERGIC-53 OS=Mus musculus GN=Lman1 PE=2 SV=1	1	8	0.99	2.20E+04	2.23E+04	1.80E+04	2.29E+04	2.03E+04	1.94E+04	2.62E+04	3.06E+04	2.67E+04	2.58E+04	1.52E+04	2.70E+04	2.88E+04	0.177	0.144	0.09	0.103	0.152	0.151	0.087	0.08	0.101	0.076	0.102	0.152	0.072	2	8	8	LMAN1_MOUSE, SP:Q9D0F3	SP|Q9D0F3|LMAN1_MOUSE
Q63844	MK03_MOUSE	Mitogen-activated protein kinase 3 OS=Mus musculus GN=Mapk3 PE=1 SV=5	1	1	0.99	3.22E+04	2.98E+04	3.02E+04	3.11E+04	2.63E+04	3.21E+04	2.59E+04	4.08E+04	4.12E+04	3.89E+04	4.92E+04	3.33E+04	2.74E+04	0.131	0.109	0.042	0.07	0.121	0.093	0.088	0.052	0.057	0.039	0.011	0.127	0.077	4	1	1	MK03_MOUSE, SP:Q63844	SP|Q63844|MK03_MOUSE
P53810	PIPNA_MOUSE	Phosphatidylinositol transfer protein alpha isoform OS=Mus musculus GN=Pitpna PE=1 SV=2	1	3	0.99	8.56E+04	5.90E+04	7.13E+04	7.56E+04	5.11E+04	5.55E+04	5.69E+04	6.11E+04	6.83E+04	5.63E+04	7.56E+04	6.97E+04	8.40E+04	0.034	0.042	0.004	0.011	0.052	0.041	0.023	0.024	0.021	0.017	0.003	0.05	0.006	4	3	3	PIPNA_MOUSE, SP:P53810	SP|P53810|PIPNA_MOUSE
P63328	PP2BB_MOUSE	Serine/threonine-protein phosphatase 2B catalytic subunit alpha isoform OS=Mus musculus GN=Ppp3ca PE=1 SV=1	1	5	0.99	2.07E+04	1.69E+04	1.21E+04	1.47E+04	2.25E+04	9.66E+03	1.87E+04	1.74E+04	1.32E+04	2.18E+04	1.69E+04	4.94E+03	9.73E+03	0.184	0.177	0.136	0.155	0.139	0.234	0.125	0.144	0.185	0.093	0.09	0.331	0.198	2	5	5	PP2BB_MOUSE, SP:P48453	SP|P48453|PP2BB_MOUSE,SP|P63328|PP2BA_MOUSE
P26645	MARCS_MOUSE	Myristoylated alanine-rich C-kinase substrate OS=Mus musculus GN=Marcks PE=1 SV=2	3	9	1	5.02E+04	4.87E+04	1.08E+04	3.22E+04	4.07E+04	3.94E+04	3.30E+04	4.54E+04	5.83E+04	4.88E+04	3.42E+04	4.24E+04	4.63E+04	0.08	0.057	0.149	0.066	0.072	0.071	0.063	0.043	0.03	0.024	0.028	0.099	0.032	3	1, 9	1, 9	MARCS_MOUSE, SP:P26645	SP|P26645|MARCS_MOUSE
Q9JJK2	LANC2_MOUSE	LanC-like protein 2 OS=Mus musculus GN=Lancl2 PE=1 SV=1	1	1	0.84	5.59E+03	4.79E+03	3.38E+04	1.35E+04	2.67E+04	3.34E+04	3.23E+04	1.20E+04	4.03E+04	1.72E+04	3.60E+04	5.86E+04	4.39E+04	0.324	0.317	0.034	0.167	0.12	0.089	0.066	0.191	0.059	0.122	0.026	0.065	0.036	3	1	1	LANC2_MOUSE, SP:Q9JJK2	SP|Q9JJK2|LANC2_MOUSE
Q3UH68	LIMC1_MOUSE	LIM and calponin homology domains-containing protein 1 OS=Mus musculus GN=Limch1 PE=1 SV=2	2	1	1	3.62E+04	3.84E+04	2.02E+04	1.98E+04	2.75E+04	2.32E+04	1.27E+04	2.72E+04	3.12E+04	2.75E+04	2.85E+04	3.06E+04	2.54E+04	0.118	0.082	0.079	0.12	0.116	0.13	0.172	0.093	0.084	0.07	0.041	0.137	0.085	2, 3	1	1	LIMC1_MOUSE, SP:Q3UH68	SP|Q3UH68|LIMC1_MOUSE
Q6R0H7	GNAS1_MOUSE	Guanine nucleotide-binding protein G(s) subunit alpha isoforms XLas OS=Mus musculus GN=Gnas PE=2 SV=1	3	7	1	1.40E+05	1.27E+05	1.06E+05	1.21E+05	1.53E+05	1.29E+05	1.13E+05	1.14E+05	1.11E+05	8.82E+04	9.28E+04	1.44E+05	1.16E+05	0.01	0.006	5.82E-04	0.002	0.002	0.004	0.002	0.003	0.005	0.004	8.47E-04	0.009	0.002	2, 5	2, 3, 7	2, 3, 7	GNAS1_MOUSE, SP:Q6R0H7	SP|Q6R0H7|GNAS1_MOUSE,SP|P63094|GNAS2_MOUSE
Q61233	PLSL_MOUSE	Plastin-2 OS=Mus musculus GN=Lcp1 PE=1 SV=4	2	5	1	8.11E+04	7.48E+04	5.26E+04	7.64E+04	7.06E+04	5.40E+04	5.88E+04	1.65E+05	1.21E+05	1.20E+05	6.76E+04	1.04E+05	1.53E+05	0.038	0.027	0.014	0.011	0.029	0.046	0.021	5.85E-04	0.003	0.001	0.004	0.022	3.87E-04	3, 5	1, 5	1, 5	PLSL_MOUSE, SP:Q61233	SP|Q61233|PLSL_MOUSE
Q9Z1J3	NFS1_MOUSE	Cysteine desulfurase, mitochondrial OS=Mus musculus GN=Nfs1 PE=2 SV=2	1	1	0.91	0.00E+00	2.75E+03	1.88E+04	1.00E+04	3.19E+03	1.77E+04	3.12E+04	1.08E+04	8.90E+03	0.00E+00	7.89E+03	5.41E+03	6.67E+03	0.5	0.354	0.085	0.201	0.341	0.162	0.068	0.201	0.231	0.5	0.18	0.323	0.24	3	1	1	NFS1_MOUSE, SP:Q9Z1J3	SP|Q9Z1J3|NFS1_MOUSE
Q91WU5	AS3MT_MOUSE	Arsenite methyltransferase OS=Mus musculus GN=As3mt PE=2 SV=1	1	2	0.99	3.53E+03	7.29E+03	2.06E+04	2.30E+04	6.54E+03	1.22E+04	1.94E+04	1.58E+04	1.05E+04	1.15E+04	6.18E+03	7.16E+03	1.82E+04	0.355	0.271	0.076	0.102	0.279	0.207	0.12	0.156	0.212	0.169	0.209	0.298	0.122	3	2	2	AS3MT_MOUSE, SP:Q91WU5	SP|Q91WU5|AS3MT_MOUSE
Q8BP40	PPA6_MOUSE	Lysophosphatidic acid phosphatase type 6 OS=Mus musculus GN=Acp6 PE=2 SV=1	1	1	0.99	3.29E+04	3.16E+04	2.51E+04	3.58E+04	3.16E+04	3.14E+04	3.24E+04	3.36E+04	2.85E+04	1.73E+04	2.06E+04	2.05E+04	1.82E+04	0.128	0.103	0.057	0.057	0.099	0.095	0.065	0.07	0.094	0.12	0.07	0.185	0.123	2	1	1	PPA6_MOUSE, SP:Q8BP40	SP|Q8BP40|PPA6_MOUSE
P35505	FAAA_MOUSE	Fumarylacetoacetase OS=Mus musculus GN=Fah PE=1 SV=1	1	3	0.99	1.03E+05	7.90E+04	3.85E+04	8.13E+04	1.16E+05	9.59E+04	7.42E+04	6.64E+04	5.53E+04	4.63E+04	3.79E+04	6.74E+04	3.89E+04	0.022	0.022	0.025	0.008	0.007	0.011	0.011	0.019	0.033	0.027	0.022	0.052	0.044	5	3	3	FAAA_MOUSE, SP:P35505	SP|P35505|FAAA_MOUSE
Q924M7	MPI_MOUSE	Mannose-6-phosphate isomerase OS=Mus musculus GN=Mpi PE=2 SV=1	7	13	1	9.12E+05	6.59E+05	6.91E+05	6.89E+05	7.72E+05	6.61E+05	7.12E+05	7.91E+05	8.01E+05	7.39E+05	8.85E+05	8.84E+05	7.54E+05	1.30E-09	4.07E-09	7.25E-17	2.40E-12	1.22E-10	1.02E-09	7.49E-13	5.75E-13	1.86E-12	1.09E-14	4.13E-22	2.27E-09	4.90E-14	2, 3, 4, 7	1, 11, 12, 13, 2, 2, 12, 3	1, 11, 12, 12, 2, 13, 2, 3	MPI_MOUSE, SP:Q924M7	SP|Q924M7|MPI_MOUSE
Q60759	GCDH_MOUSE	Glutaryl-CoA dehydrogenase, mitochondrial OS=Mus musculus GN=Gcdh PE=2 SV=1	4	9	1	2.51E+05	2.82E+05	3.54E+05	3.12E+05	4.35E+05	3.58E+05	3.44E+05	2.99E+05	2.49E+05	2.99E+05	3.33E+05	2.77E+05	2.65E+05	8.74E-04	7.86E-05	1.58E-09	1.54E-06	9.77E-07	5.03E-06	4.26E-07	6.08E-06	5.18E-05	5.57E-07	1.70E-09	5.11E-04	4.71E-06	2, 3, 4, 5	2, 4, 3, 4, 9	2, 4, 3, 4, 9	GCDH_MOUSE, SP:Q60759	SP|Q60759|GCDH_MOUSE
Q71RI9	KAT3_MOUSE	Kynurenine--oxoglutarate transaminase 3 OS=Mus musculus GN=Ccbl2 PE=2 SV=1	3	6	1	1.87E+05	2.32E+05	1.96E+05	2.11E+05	2.17E+05	2.16E+05	2.50E+05	1.57E+05	1.44E+05	1.55E+05	1.84E+05	1.76E+05	1.78E+05	0.003	3.09E-04	5.35E-06	6.35E-05	3.90E-04	3.01E-04	1.36E-05	7.59E-04	0.002	2.36E-04	5.79E-06	0.004	1.44E-04	2, 3, 4	3, 6	3, 6	KAT3_MOUSE, SP:Q71RI9	SP|Q71RI9|KAT3_MOUSE
Q9JMC3	DNJA4_MOUSE	DnaJ homolog subfamily A member 4 OS=Mus musculus GN=Dnaja4 PE=2 SV=1	2	9	1	1.32E+05	1.13E+05	8.58E+04	1.28E+05	1.01E+05	9.49E+04	8.76E+04	1.12E+05	1.66E+05	1.31E+05	1.34E+05	1.32E+05	1.39E+05	0.011	0.008	0.002	0.001	0.011	0.011	0.006	0.003	7.39E-04	6.30E-04	8.37E-05	0.012	6.49E-04	5	6, 9	6, 9	DNJA4_MOUSE, SP:Q9JMC3	SP|Q9JMC3|DNJA4_MOUSE
O70571	PDK4_MOUSE	[Pyruvate dehydrogenase [lipoamide]] kinase isozyme 4, mitochondrial OS=Mus musculus GN=Pdk4 PE=2 SV=1	3	5	1	1.31E+05	8.69E+04	1.04E+05	9.95E+04	1.15E+05	8.71E+04	9.11E+04	9.54E+04	8.39E+04	7.26E+04	7.98E+04	7.74E+04	8.45E+04	0.012	0.019	8.62E-04	0.005	0.007	0.016	0.006	0.007	0.013	0.009	0.002	0.046	0.008	2, 3, 4	2, 5	2, 5	PDK4_MOUSE, SP:O70571	SP|O70571|PDK4_MOUSE
Q9DBG5	PLIN3_MOUSE	Perilipin-3 OS=Mus musculus GN=Plin3 PE=1 SV=1	7	13	1	1.21E+06	1.05E+06	8.33E+05	9.92E+05	8.96E+05	9.27E+05	8.65E+05	9.25E+05	8.18E+05	7.40E+05	9.73E+05	9.51E+05	8.60E+05	2.87E-12	1.66E-13	5.70E-20	5.03E-17	4.22E-12	5.82E-13	3.01E-15	6.36E-15	1.01E-12	9.62E-15	3.04E-24	5.77E-10	8.31E-16	3, 4, 6, 7	1, 11, 12, 13, 2, 8	1, 11, 12, 13, 2, 8	PLIN3_MOUSE, SP:Q9DBG5	SP|Q9DBG5|PLIN3_MOUSE
Q9QZQ8	H2AY_MOUSE	Core histone macro-H2A.1 OS=Mus musculus GN=H2afy PE=1 SV=3	1	10	0.99	6.30E+04	8.50E+04	8.96E+04	9.11E+04	8.14E+04	8.19E+04	9.25E+04	8.88E+04	7.55E+04	8.10E+04	8.66E+04	5.16E+04	9.82E+04	0.058	0.019	0.001	0.006	0.02	0.017	0.005	0.008	0.016	0.006	0.001	0.078	0.003	4	10	10	H2AY_MOUSE, SP:Q9QZQ8	SP|Q9QZQ8|H2AY_MOUSE
Q61646	HPT_MOUSE	Haptoglobin OS=Mus musculus GN=Hp PE=1 SV=1	1	2	1	2.47E+04	7.08E+04	1.54E+04	3.85E+04	2.34E+03	3.24E+04	2.42E+04	2.22E+04	3.84E+03	6.89E+04	1.79E+04	2.80E+03	3.60E+04	0.163	0.029	0.108	0.051	0.367	0.092	0.096	0.116	0.317	0.01	0.085	0.372	0.051	2	2	2	HPT_MOUSE, SP:Q61646	SP|Q61646|HPT_MOUSE
Q8BK72	RT27_MOUSE	28S ribosomal protein S27, mitochondrial OS=Mus musculus GN=Mrps27 PE=2 SV=1	1	3	0.83	8.62E+03	1.52E+04	1.42E+04	1.33E+04	1.30E+04	9.90E+03	1.11E+04	8.10E+03	8.13E+03	1.25E+04	1.06E+04	4.39E+03	1.38E+04	0.28	0.189	0.117	0.167	0.204	0.23	0.187	0.234	0.241	0.159	0.145	0.339	0.155	3	2	3	RT27_MOUSE, SP:Q8BK72	SP|Q8BK72|RT27_MOUSE
P22315	HEMH_MOUSE	Ferrochelatase, mitochondrial OS=Mus musculus GN=Fech PE=1 SV=2	2	4	1	1.87E+05	2.29E+05	1.73E+05	2.36E+05	2.08E+05	2.58E+05	2.10E+05	2.39E+05	1.89E+05	1.67E+05	2.08E+05	2.40E+05	2.43E+05	0.004	3.32E-04	1.97E-05	2.61E-05	5.09E-04	9.01E-05	6.22E-05	4.66E-05	3.73E-04	1.41E-04	1.59E-06	0.001	1.16E-05	4, 5	4	4	HEMH_MOUSE, SP:P22315	SP|P22315|HEMH_MOUSE
Q9ESW4	AGK_MOUSE	Acylglycerol kinase, mitochondrial OS=Mus musculus GN=Agk PE=1 SV=1	2	8	1	1.55E+05	1.13E+05	1.03E+05	1.16E+05	1.04E+05	1.15E+05	9.11E+04	9.31E+04	9.11E+04	7.85E+04	9.07E+04	1.04E+05	7.89E+04	0.007	0.008	6.97E-04	0.002	0.01	0.006	0.006	0.007	0.009	0.006	0.001	0.022	0.008	2, 5	2, 8	2, 8	AGK_MOUSE, SP:Q9ESW4	SP|Q9ESW4|AGK_MOUSE
Q9D1B9	RM28_MOUSE	39S ribosomal protein L28, mitochondrial OS=Mus musculus GN=Mrpl28 PE=2 SV=3	1	6	0.99	9.03E+04	6.60E+04	8.01E+04	5.69E+04	6.08E+04	4.94E+04	4.95E+04	8.47E+04	6.24E+04	5.14E+04	4.45E+04	4.45E+04	3.21E+04	0.03	0.033	0.002	0.023	0.037	0.05	0.03	0.01	0.026	0.021	0.015	0.094	0.061	5	6	6	RM28_MOUSE, SP:Q9D1B9	SP|Q9D1B9|RM28_MOUSE
Q8BTS4	NUP54_MOUSE	Nuclear pore complex protein Nup54 OS=Mus musculus GN=Nup54 PE=1 SV=1	1	6	0.58	9.20E+04	1.28E+05	9.81E+04	1.62E+05	1.17E+05	1.10E+05	1.17E+05	8.99E+04	9.87E+04	1.23E+05	1.33E+05	9.38E+04	1.39E+05	0.029	0.005	9.19E-04	3.96E-04	0.007	0.007	0.002	0.008	0.007	9.09E-04	9.32E-05	0.028	6.91E-04	2	6	6	NUP54_MOUSE, SP:Q8BTS4	SP|Q8BTS4|NUP54_MOUSE
P00330	ADH1_YEAST	Alcohol dehydrogenase I (EC 1.1.1.1) OS=Saccharomyces cerevisiae GN= ADH1 PE=1 SV=5	16	13	1	6.59E+07	7.60E+07	7.60E+07	7.74E+07	5.96E+07	8.83E+07	1.07E+08	1.00E+08	5.76E+07	7.36E+07	8.62E+07	7.44E+07	9.09E+07	0	0	0	0	0	0	0	0	0	0	0	0	0	3, 3, 4, 6, 4, 4, 5, 5, 5, 6, 6, 7	1, 8, 13, 11, 13, 12, 13, 4, 5, 13, 7, 10, 8, 12, 9	1, 21, 10, 20, 12, 12, 8, 13, 17, 20, 22, 6, 26, 28, 4, 9	ADH1_YEAST, SP:P00330	SP|P00330|ADH1_YEAST
P54923	ADPRH_MOUSE	[Protein ADP-ribosylarginine] hydrolase OS=Mus musculus GN=Adprh PE=2 SV=1	1	1	0.97	4.60E+04	2.63E+04	3.77E+04	5.15E+04	3.45E+04	4.83E+04	2.93E+04	3.95E+04	3.14E+04	3.21E+04	4.06E+04	3.01E+04	4.57E+04	0.09	0.124	0.026	0.029	0.09	0.052	0.075	0.055	0.084	0.055	0.02	0.139	0.033	4	1	1	ADPRH_MOUSE, SP:P54923	SP|P54923|ADPRH_MOUSE
O88696	CLPP_MOUSE	Putative ATP-dependent Clp protease proteolytic subunit, mitochondrial OS=Mus musculus GN=Clpp PE=2 SV=1	1	13	0.99	1.19E+05	1.15E+05	1.00E+05	8.57E+04	1.12E+05	9.43E+04	9.58E+04	9.15E+04	6.83E+04	9.31E+04	7.10E+04	1.11E+05	6.98E+04	0.016	0.008	8.23E-04	0.007	0.008	0.012	0.005	0.008	0.021	0.003	0.003	0.019	0.012	4	13	13	CLPP_MOUSE, SP:O88696	SP|O88696|CLPP_MOUSE
O35972	RM23_MOUSE	39S ribosomal protein L23, mitochondrial OS=Mus musculus GN=Mrpl23 PE=2 SV=1	1	2	0.99	2.11E+04	1.41E+04	1.45E+04	1.71E+04	2.02E+04	1.64E+04	9.78E+03	2.04E+04	9.69E+03	1.24E+04	1.33E+04	7.80E+03	8.79E+03	0.182	0.2	0.116	0.138	0.152	0.173	0.204	0.126	0.222	0.161	0.12	0.291	0.212	2	2	2	RM23_MOUSE, SP:O35972	SP|O35972|RM23_MOUSE
Q9WV60	GSK3B_MOUSE	Glycogen synthase kinase-3 beta OS=Mus musculus GN=Gsk3b PE=1 SV=2	1	2	0.99	6.45E+04	4.23E+04	4.76E+04	4.50E+04	4.78E+04	4.81E+04	5.14E+04	5.10E+04	5.75E+04	5.98E+04	6.00E+04	5.41E+04	5.52E+04	0.056	0.071	0.015	0.038	0.057	0.052	0.028	0.034	0.031	0.015	0.006	0.073	0.021	3	2	2	GSK3B_MOUSE, SP:Q9WV60	SP|Q9WV60|GSK3B_MOUSE
Q8C156	CND2_MOUSE	Condensin complex subunit 2 OS=Mus musculus GN=Ncaph PE=2 SV=1	1	12	0.97	1.78E+05	1.84E+05	2.39E+05	2.18E+05	8.70E+04	1.63E+05	2.46E+05	1.55E+05	2.46E+05	5.29E+05	1.21E+05	1.17E+05	1.75E+05	0.004	0.001	6.83E-07	5.10E-05	0.016	0.001	1.68E-05	8.05E-04	5.98E-05	9.64E-11	1.78E-04	0.016	1.63E-04	4	12	12	CND2_MOUSE, SP:Q8C156	SP|Q8C156|CND2_MOUSE
Q99J45	NRBP_MOUSE	Nuclear receptor-binding protein OS=Mus musculus GN=Nrbp1 PE=1 SV=1	1	1	0.66	4.88E+04	4.41E+04	3.10E+04	4.12E+04	3.99E+04	3.84E+04	3.85E+04	5.21E+04	5.29E+04	3.45E+04	4.71E+04	6.03E+04	6.87E+04	0.083	0.067	0.04	0.045	0.074	0.074	0.049	0.033	0.036	0.049	0.013	0.062	0.012	3	1	1	NRBP_MOUSE, SP:Q99J45	SP|Q99J45|NRBP_MOUSE
P31750	AKT1_MOUSE	RAC-alpha serine/threonine-protein kinase OS=Mus musculus GN=Akt1 PE=1 SV=1	1	1	0.92	9.42E+03	1.08E+04	1.12E+04	1.38E+04	9.31E+03	1.01E+04	7.36E+03	9.95E+03	1.19E+04	8.61E+03	1.51E+04	7.06E+03	1.14E+04	0.272	0.229	0.145	0.162	0.242	0.228	0.235	0.211	0.197	0.204	0.103	0.299	0.179	2	1	1	AKT1_MOUSE, SP:P31750	SP|P31750|AKT1_MOUSE
Q8BGC4	ZADH2_MOUSE	Zinc-binding alcohol dehydrogenase domain-containing protein 2 OS=Mus musculus GN=Zadh2 PE=2 SV=1	4	11	1	2.81E+05	2.87E+05	2.60E+05	3.08E+05	3.18E+05	3.28E+05	3.11E+05	2.48E+05	2.13E+05	1.98E+05	2.25E+05	2.49E+05	2.28E+05	4.49E-04	6.70E-05	1.87E-07	1.70E-06	2.26E-05	1.14E-05	1.34E-06	3.11E-05	1.59E-04	3.54E-05	5.47E-07	9.01E-04	1.96E-05	3, 4, 4, 4, 5	1, 11, 2, 4, 4, 10	1, 10, 4, 11, 2, 4	ZADH2_MOUSE, SP:Q8BGC4	SP|Q8BGC4|ZADH2_MOUSE
O35943	FRDA_MOUSE	Frataxin, mitochondrial OS=Mus musculus GN=Fxn PE=2 SV=1	1	3	1	1.43E+04	2.85E+04	2.37E+04	2.63E+04	1.78E+04	1.43E+04	2.59E+04	2.22E+04	2.04E+04	1.69E+04	1.64E+04	1.42E+04	1.36E+04	0.227	0.114	0.063	0.087	0.167	0.188	0.088	0.115	0.133	0.123	0.093	0.229	0.158	2	3	3	FRDA_MOUSE, SP:O35943	SP|O35943|FRDA_MOUSE
Q7M6Y3	AP180_MOUSE	Phosphatidylinositol-binding clathrin assembly protein OS=Mus musculus GN=Picalm PE=1 SV=1	1	6	0.99	5.70E+04	4.36E+04	3.87E+04	4.41E+04	3.47E+04	4.12E+04	2.85E+04	4.56E+04	4.66E+04	4.87E+04	5.43E+04	5.31E+04	6.22E+04	0.067	0.068	0.025	0.039	0.089	0.085	0.078	0.043	0.046	0.024	0.008	0.075	0.016	3	6	6	AP180_MOUSE, SP:Q61548	SP|Q61548|AP180_MOUSE,SP|Q7M6Y3|PICA_MOUSE
Q9EPK7	XPO7_MOUSE	Exportin-7 OS=Mus musculus GN=Xpo7 PE=2 SV=3	1	1	0.65	8.21E+04	7.94E+03	8.64E+03	3.24E+04	1.31E+04	1.25E+04	8.08E+04	1.84E+04	1.07E+05	2.98E+04	1.92E+04	1.54E+04	1.24E+04	0.036	0.264	0.185	0.066	0.204	0.205	0.008	0.138	0.005	0.062	0.078	0.219	0.172	4	1	1	XPO7_MOUSE, SP:Q9EPK7	SP|Q9EPK7|XPO7_MOUSE
O70548	TELT_MOUSE	Telethonin OS=Mus musculus GN=Tcap PE=2 SV=1	2	13	1	1.63E+05	1.70E+05	2.33E+05	1.69E+05	1.64E+05	1.64E+05	1.99E+05	1.87E+05	2.41E+05	1.93E+05	2.74E+05	2.14E+05	2.81E+05	0.006	0.002	9.54E-07	3.06E-04	0.002	0.001	8.95E-05	2.54E-04	6.72E-05	4.65E-05	4.24E-08	0.002	2.77E-06	3, 4	11, 13	11, 13	TELT_MOUSE, SP:O70548	SP|O70548|TELT_MOUSE
Q9CRA7	ATP5S_MOUSE	ATP synthase subunit s, mitochondrial OS=Mus musculus GN=Atp5s PE=2 SV=1	1	1	0.95	2.57E+04	2.05E+04	2.77E+04	2.28E+04	2.49E+04	2.03E+04	2.05E+04	2.09E+04	2.01E+04	2.02E+04	2.52E+04	2.13E+04	2.30E+04	0.158	0.154	0.049	0.103	0.127	0.147	0.114	0.123	0.135	0.103	0.052	0.181	0.096	2	1	1	ATP5S_MOUSE, SP:Q9CRA7	SP|Q9CRA7|ATP5S_MOUSE
Q9CXJ1	SYEM_MOUSE	Probable glutamyl-tRNA synthetase, mitochondrial OS=Mus musculus GN=Ears2 PE=2 SV=1	1	12	0.78	5.58E+05	6.65E+05	6.64E+05	4.80E+05	6.12E+05	3.86E+05	6.04E+05	5.88E+05	4.76E+05	7.55E+05	6.10E+05	5.52E+05	6.09E+05	1.91E-06	5.34E-09	2.15E-15	5.91E-09	1.34E-08	2.92E-06	8.95E-11	7.68E-10	6.26E-08	3.58E-14	3.68E-15	2.21E-06	2.79E-11	4	12	12	SYEM_MOUSE, SP:Q9CXJ1	SP|Q9CXJ1|SYEM_MOUSE
P82348	SGCG_MOUSE	Gamma-sarcoglycan OS=Mus musculus GN=Sgcg PE=1 SV=2	2	8	1	2.00E+05	2.27E+05	1.77E+05	1.94E+05	1.93E+05	1.97E+05	1.96E+05	1.73E+05	1.68E+05	1.69E+05	2.04E+05	1.71E+05	2.00E+05	0.003	3.42E-04	1.43E-05	1.15E-04	7.46E-04	5.24E-04	9.90E-05	4.15E-04	7.07E-04	1.22E-04	1.76E-06	0.005	6.14E-05	3, 4	3, 8	3, 8	SGCG_MOUSE, SP:P82348	SP|P82348|SGCG_MOUSE
P24472	GSTA4_MOUSE	Glutathione S-transferase A4 OS=Mus musculus GN=Gsta4 PE=1 SV=3	2	4	1	4.09E+05	3.78E+05	3.15E+05	5.03E+05	2.61E+05	3.13E+05	3.42E+05	3.35E+05	2.78E+05	2.72E+05	2.89E+05	2.64E+05	2.95E+05	5.09E-05	1.11E-05	2.03E-08	4.67E-09	1.84E-04	3.65E-05	7.56E-07	5.79E-06	3.19E-05	3.78E-06	1.67E-07	9.40E-04	5.61E-06	4, 5	1, 4	1, 4	GSTA4_MOUSE, SP:P24472	SP|P24472|GSTA4_MOUSE
Q9QZZ6	DERM_MOUSE	Dermatopontin OS=Mus musculus GN=Dpt PE=2 SV=1	1	4	0.93	3.10E+04	3.44E+04	1.92E+04	1.59E+04	2.36E+04	2.09E+04	2.58E+04	2.15E+04	1.80E+04	2.80E+04	1.78E+04	2.24E+04	2.24E+04	0.135	0.093	0.084	0.146	0.133	0.143	0.088	0.12	0.148	0.068	0.085	0.175	0.099	2	4	4	DERM_MOUSE, SP:Q9QZZ6	SP|Q9QZZ6|DERM_MOUSE
P11531	DMD_MOUSE	Dystrophin OS=Mus musculus GN=Dmd PE=1 SV=2	22	13	1	1.42E+06	1.38E+06	1.19E+06	1.26E+06	1.24E+06	1.21E+06	1.19E+06	1.38E+06	1.22E+06	1.30E+06	1.22E+06	1.31E+06	1.37E+06	4.66E-14	3.40E-17	1.75E-27	5.21E-21	5.19E-16	2.26E-16	3.24E-20	2.14E-21	4.85E-18	2.16E-24	9.08E-30	5.05E-13	5.41E-24	2, 3, 3, 5, 4, 5	1, 1, 13, 11, 12, 2, 2, 12, 3, 4, 5, 6, 8, 9	1, 1, 13, 11, 12, 2, 2, 3, 4, 5, 6, 8, 9	DMD_MOUSE, SP:P11531	SP|P11531|DMD_MOUSE
Q62009	POSTN_MOUSE	Periostin OS=Mus musculus GN=Postn PE=1 SV=2	6	5	1	1.14E+05	1.07E+05	1.09E+05	7.95E+04	8.73E+04	9.11E+04	8.59E+04	1.64E+05	3.32E+05	2.19E+05	2.57E+05	2.22E+05	6.26E+05	0.019	0.01	6.09E-04	0.011	0.017	0.013	0.007	6.09E-04	3.83E-06	1.74E-05	1.19E-07	0.002	5.95E-12	2, 3, 4	1, 2	1, 2, 5	POSTN_MOUSE, SP:Q62009	SP|Q62009|POSTN_MOUSE
Q62407	SPEG_MOUSE	Striated muscle-specific serine/threonine-protein kinase OS=Mus musculus GN=Speg PE=1 SV=2	3	9	1	8.78E+04	8.04E+04	8.21E+04	7.69E+04	8.52E+04	7.22E+04	6.33E+04	8.00E+04	7.36E+04	6.92E+04	4.74E+04	8.15E+04	6.03E+04	0.032	0.022	0.002	0.01	0.017	0.024	0.017	0.012	0.017	0.009	0.013	0.037	0.017	2, 5	2, 3, 9	3, 4, 9	SPEG_MOUSE, SP:Q62407	SP|Q62407|SPEG_MOUSE
P01027	CO3_MOUSE	Complement C3 OS=Mus musculus GN=C3 PE=1 SV=2	8	9	1	3.82E+06	3.49E+06	4.53E+06	4.87E+06	3.03E+06	3.70E+06	3.07E+06	4.30E+06	3.44E+06	3.69E+06	4.02E+06	4.59E+06	2.97E+06	1.39E-32	5.13E-38	0	0	3.16E-34	1.49E-42	4.20E-45	0	1.12E-43	0	0	5.71E-38	0	2, 3, 4, 5, 8	1, 2, 4, 9	1, 2, 4, 9	CO3_MOUSE, SP:P01027	SP|P01027|CO3_MOUSE
Q9WUR9	KAD4_MOUSE	Adenylate kinase isoenzyme 4, mitochondrial OS=Mus musculus GN=Ak3l1 PE=2 SV=1	2	4	1	4.54E+04	5.50E+04	8.40E+04	5.76E+04	5.06E+04	7.58E+04	8.97E+04	4.65E+04	4.36E+04	3.56E+04	4.10E+04	3.79E+04	4.84E+04	0.091	0.047	0.002	0.022	0.052	0.021	0.006	0.041	0.052	0.046	0.018	0.112	0.029	3	2, 4	2, 4	KAD4_MOUSE, SP:Q9WUR9	SP|Q9WUR9|KAD4_MOUSE
Q9Z2M7	PMM2_MOUSE	Phosphomannomutase 2 OS=Mus musculus GN=Pmm2 PE=2 SV=1	1	2	0.99	1.50E+04	1.60E+04	1.76E+04	1.98E+04	1.14E+04	1.60E+04	2.40E+04	1.62E+04	1.55E+04	2.57E+04	1.44E+04	2.04E+04	2.30E+04	0.221	0.183	0.092	0.119	0.22	0.175	0.096	0.153	0.166	0.076	0.108	0.186	0.096	2	2	2	PMM2_MOUSE, SP:Q9Z2M7	SP|Q9Z2M7|PMM2_MOUSE
Q8K4F5	ABHDB_MOUSE	Abhydrolase domain-containing protein 11 OS=Mus musculus GN=Abhd11 PE=2 SV=1	3	13	1	2.46E+05	3.06E+05	2.67E+05	3.07E+05	2.42E+05	2.77E+05	2.78E+05	2.65E+05	2.20E+05	2.36E+05	2.86E+05	2.00E+05	2.71E+05	0.001	4.26E-05	1.75E-07	2.15E-06	2.00E-04	5.10E-05	5.08E-06	1.90E-05	1.33E-04	8.54E-06	2.75E-08	0.003	4.31E-06	3, 4, 5	10, 12, 13	10, 12, 13	ABHDB_MOUSE, SP:Q8K4F5	SP|Q8K4F5|ABHDB_MOUSE
Q64669	NQO1_MOUSE	NAD(P)H dehydrogenase [quinone] 1 OS=Mus musculus GN=Nqo1 PE=1 SV=3	2	8	1	1.40E+05	1.44E+05	1.05E+05	1.55E+05	1.75E+05	1.54E+05	1.50E+05	1.26E+05	1.27E+05	1.35E+05	1.60E+05	2.25E+05	2.20E+05	0.01	0.004	6.69E-04	5.23E-04	0.001	0.002	5.93E-04	0.002	0.003	5.52E-04	2.17E-05	0.002	2.79E-05	4, 5	4, 8	4, 8	NQO1_MOUSE, SP:Q64669	SP|Q64669|NQO1_MOUSE
Q9CR24	NUDT8_MOUSE	Nucleoside diphosphate-linked moiety X motif 8, mitochondrial OS=Mus musculus GN=Nudt8 PE=1 SV=1	1	4	0.99	4.35E+04	3.08E+04	3.43E+04	3.74E+04	3.88E+04	4.29E+04	3.43E+04	2.56E+04	3.01E+04	1.77E+04	3.46E+04	3.55E+04	3.18E+04	0.096	0.105	0.033	0.053	0.077	0.063	0.06	0.099	0.088	0.118	0.028	0.12	0.063	2	4	4	NUDT8_MOUSE, SP:Q9CR24	SP|Q9CR24|NUDT8_MOUSE
Q9D7J9	ECHD3_MOUSE	Enoyl-CoA hydratase domain-containing protein 3, mitochondrial OS=Mus musculus GN=Echdc3 PE=2 SV=1	3	12	1	1.08E+05	1.41E+05	1.35E+05	1.34E+05	1.06E+05	1.44E+05	1.36E+05	1.38E+05	1.07E+05	9.81E+04	1.08E+05	8.05E+04	1.08E+05	0.02	0.004	1.33E-04	0.001	0.01	0.003	0.001	0.001	0.006	0.003	4.13E-04	0.039	0.002	2, 3, 4	12, 3	12, 3	ECHD3_MOUSE, SP:Q9D7J9	SP|Q9D7J9|ECHD3_MOUSE
Q8BZA9	TIGAR_MOUSE	Probable fructose-2,6-bisphosphatase TIGAR OS=Mus musculus GN=Tigar PE=2 SV=1	1	5	0.97	3.75E+03	1.21E+04	2.41E+04	1.72E+04	1.07E+04	9.78E+03	2.21E+04	1.72E+04	1.83E+04	1.13E+04	1.65E+04	7.29E+03	1.79E+04	0.351	0.216	0.06	0.136	0.227	0.232	0.105	0.145	0.146	0.171	0.093	0.297	0.124	2	5	5	TIGAR_MOUSE, SP:Q8BZA9	SP|Q8BZA9|TIGAR_MOUSE
Q8VE38	OXND1_MOUSE	Oxidoreductase NAD-binding domain-containing protein 1 OS=Mus musculus GN=Oxnad1 PE=2 SV=2	1	3	0.96	7.09E+03	5.18E+03	1.33E+04	9.32E+03	3.97E+03	7.12E+03	7.45E+03	7.36E+03	6.06E+03	8.64E+03	1.13E+04	5.95E+03	9.30E+03	0.299	0.303	0.124	0.209	0.324	0.266	0.233	0.245	0.272	0.203	0.136	0.315	0.203	3	3	3	OXND1_MOUSE, SP:Q8VE38	SP|Q8VE38|OXND1_MOUSE
Q61160	FADD_MOUSE	Protein FADD OS=Mus musculus GN=Fadd PE=1 SV=1	1	7	0.99	7.32E+04	4.52E+04	3.69E+04	5.47E+04	5.66E+04	6.72E+04	4.91E+04	6.14E+04	6.12E+04	5.13E+04	4.84E+04	6.82E+04	5.79E+04	0.046	0.065	0.029	0.026	0.043	0.028	0.032	0.024	0.027	0.022	0.012	0.051	0.019	4	7	7	FADD_MOUSE, SP:Q61160	SP|Q61160|FADD_MOUSE
Q8K3A0	HSC20_MOUSE	Iron-sulfur cluster co-chaperone protein HscB, mitochondrial OS=Mus musculus GN=Hscb PE=2 SV=1	1	3	0.57	5.08E+05	3.61E+05	1.62E+05	2.08E+05	2.96E+05	2.43E+05	1.58E+05	2.06E+05	1.84E+05	1.75E+05	1.66E+05	2.99E+05	1.12E+05	4.94E-06	1.11E-05	3.36E-05	7.72E-05	4.76E-05	1.45E-04	4.36E-04	1.43E-04	4.51E-04	1.10E-04	1.67E-05	3.38E-04	0.002	3	3	3	HSC20_MOUSE, SP:Q8K3A0	SP|Q8K3A0|HSC20_MOUSE
P61922	GABT_MOUSE	4-aminobutyrate aminotransferase, mitochondrial OS=Mus musculus GN=Abat PE=1 SV=1	2	1	1	2.79E+04	2.60E+04	2.57E+04	3.09E+04	3.36E+04	5.18E+04	6.00E+04	1.64E+04	3.13E+04	3.48E+04	5.16E+04	7.11E+04	1.07E+05	0.148	0.126	0.056	0.072	0.093	0.046	0.02	0.154	0.084	0.049	0.01	0.048	0.002	3, 4	1	1	GABT_MOUSE, SP:P61922	SP|P61922|GABT_MOUSE
Q8C8R3	ANK2_MOUSE	Ankyrin-2 OS=Mus musculus GN=Ank2 PE=1 SV=2	1	9	1	5.11E+04	3.77E+04	6.22E+04	3.57E+04	3.83E+04	3.24E+04	3.11E+04	4.04E+04	2.52E+04	1.11E+05	2.71E+04	2.71E+04	2.67E+04	0.078	0.083	0.006	0.057	0.078	0.091	0.069	0.053	0.108	0.001	0.045	0.152	0.08	3	9	9	ANK2_MOUSE, SP:Q8C8R3	SP|Q8C8R3|ANK2_MOUSE
Q9D6Y7	MSRA_MOUSE	Peptide methionine sulfoxide reductase OS=Mus musculus GN=Msra PE=1 SV=1	3	4	1	1.43E+05	1.86E+05	1.51E+05	1.63E+05	1.62E+05	1.34E+05	1.82E+05	1.71E+05	1.76E+05	1.48E+05	1.75E+05	2.14E+05	1.56E+05	0.009	0.001	5.51E-05	3.95E-04	0.002	0.003	1.78E-04	4.70E-04	5.49E-04	3.09E-04	9.30E-06	0.002	3.61E-04	2, 4, 6	4	4	MSRA_MOUSE, SP:Q9D6Y7	SP|Q9D6Y7|MSRA_MOUSE
Q8BRH0	TMTC3_MOUSE	Transmembrane and TPR repeat-containing protein 3 OS=Mus musculus GN=Tmtc3 PE=2 SV=1	1	8	0.92	9.70E+05	7.94E+05	9.72E+05	1.17E+06	5.67E+05	7.39E+05	7.24E+05	1.00E+06	7.09E+05	6.65E+05	6.74E+05	2.90E+05	5.06E+05	5.39E-10	1.82E-10	8.15E-22	5.70E-19	3.41E-08	1.68E-10	9.26E-13	1.52E-15	4.87E-11	4.32E-13	6.66E-17	3.91E-04	6.86E-10	6	8	8	TMTC3_MOUSE, SP:Q8BRH0	SP|Q8BRH0|TMTC3_MOUSE
P54797	T10_MOUSE	Ser/Thr-rich protein T10 in DGCR region OS=Mus musculus GN=T10 PE=2 SV=1	1	4	0.98	1.35E+05	1.12E+05	1.01E+05	1.26E+05	1.09E+05	1.18E+05	8.17E+04	9.63E+04	7.20E+04	7.85E+04	1.10E+05	1.03E+05	8.99E+04	0.011	0.008	7.88E-04	0.002	0.009	0.006	0.008	0.006	0.018	0.006	3.33E-04	0.022	0.005	5	4	4	T10_MOUSE, SP:P54797	SP|P54797|T10_MOUSE
Q9CZR8	EFTS_MOUSE	Elongation factor Ts, mitochondrial OS=Mus musculus GN=Tsfm PE=2 SV=1	5	13	1	6.08E+05	5.81E+05	4.79E+05	5.53E+05	5.86E+05	5.80E+05	4.91E+05	5.37E+05	5.90E+05	6.38E+05	6.55E+05	6.46E+05	4.48E+05	5.33E-07	2.82E-08	2.61E-12	2.66E-10	1.58E-08	8.97E-09	1.92E-09	1.99E-09	1.10E-09	5.50E-13	5.17E-17	2.56E-07	4.30E-09	3, 5, 6	1, 13, 3, 7, 9	1, 13, 3, 7, 9	EFTS_MOUSE, SP:Q9CZR8	SP|Q9CZR8|EFTS_MOUSE
O09131	GSTO1_MOUSE	Glutathione S-transferase omega-1 OS=Mus musculus GN=Gsto1 PE=2 SV=2	5	13	1	5.78E+05	1.01E+06	1.02E+06	9.81E+05	9.94E+05	9.95E+05	9.83E+05	1.06E+06	1.08E+06	8.67E+05	1.06E+06	8.85E+05	1.12E+06	9.94E-07	5.06E-13	7.88E-24	8.33E-17	3.33E-13	9.68E-14	5.27E-17	9.03E-17	4.20E-16	6.13E-17	3.72E-26	2.15E-09	5.81E-20	2, 4, 4, 5, 5, 6	12, 2, 13, 6, 9, 13	12, 13, 2, 13, 9, 6, 8	GSTO1_MOUSE, SP:O09131	SP|O09131|GSTO1_MOUSE
Q8K0Z7	TACO1_MOUSE	Translational activator of cytochrome c oxidase 1 OS=Mus musculus GN=Taco1 PE=2 SV=1	2	3	1	1.75E+04	4.96E+04	7.86E+04	3.37E+04	6.22E+04	5.48E+04	7.15E+04	3.53E+04	6.52E+04	1.15E+05	6.75E+04	6.62E+04	6.26E+04	0.204	0.056	0.003	0.062	0.036	0.041	0.012	0.065	0.023	0.001	0.004	0.054	0.016	3, 4	1, 3	1, 3	TACO1_MOUSE, SP:Q8K0Z7	SP|Q8K0Z7|TACO1_MOUSE
Q811I0	ATPF1_MOUSE	ATP synthase mitochondrial F1 complex assembly factor 1 OS=Mus musculus GN=Atpaf1 PE=2 SV=1	1	7	0.99	3.90E+04	4.23E+04	4.03E+04	3.97E+04	4.12E+04	4.01E+04	3.24E+04	3.71E+04	2.32E+04	3.44E+04	4.11E+04	3.70E+04	2.73E+04	0.111	0.074	0.025	0.052	0.073	0.071	0.068	0.063	0.123	0.053	0.022	0.116	0.084	2	7	7	ATPF1_MOUSE, SP:Q811I0	SP|Q811I0|ATPF1_MOUSE
Q9EPB5	SERHL_MOUSE	Serine hydrolase-like protein OS=Mus musculus GN=Serhl PE=2 SV=1	2	9	1	1.08E+05	9.56E+04	7.71E+04	7.54E+04	7.11E+04	7.40E+04	6.07E+04	5.79E+04	8.65E+04	7.46E+04	5.95E+04	7.52E+04	9.42E+04	0.02	0.014	0.003	0.011	0.027	0.022	0.019	0.026	0.011	0.007	0.006	0.043	0.004	2, 6	1, 9	1, 9	SERHL_MOUSE, SP:Q9EPB5	SP|Q9EPB5|SERHL_MOUSE
Q9WVL0	MAAI_MOUSE	Maleylacetoacetate isomerase OS=Mus musculus GN=Gstz1 PE=1 SV=1	5	13	1	3.75E+05	4.87E+05	3.62E+05	4.11E+05	4.26E+05	4.28E+05	3.65E+05	4.44E+05	3.38E+05	4.02E+05	4.52E+05	3.15E+05	3.15E+05	6.41E-05	3.38E-07	1.06E-09	4.28E-08	1.20E-06	6.72E-07	1.89E-07	4.57E-08	2.93E-06	8.12E-09	2.61E-12	2.26E-04	6.80E-07	3, 4, 5	13, 2, 5, 7	13, 2, 5, 7	MAAI_MOUSE, SP:Q9WVL0	SP|Q9WVL0|MAAI_MOUSE
P13707	GPDA_MOUSE	Glycerol-3-phosphate dehydrogenase [NAD+], cytoplasmic OS=Mus musculus GN=Gpd1 PE=1 SV=3	2	10	1	6.13E+04	1.12E+05	9.56E+04	8.36E+04	8.14E+04	7.66E+04	8.04E+04	9.32E+04	9.46E+04	1.08E+05	8.93E+04	6.70E+04	1.08E+05	0.061	0.009	0.001	0.008	0.02	0.02	0.009	0.007	0.008	0.002	0.001	0.053	0.002	3	1, 10	1, 10	GPDA_MOUSE, SP:P13707	SP|P13707|GPDA_MOUSE
Q9D1H9	MFAP4_MOUSE	Microfibril-associated glycoprotein 4 OS=Mus musculus GN=Mfap4 PE=1 SV=1	2	13	1	1.46E+05	1.67E+05	1.85E+05	1.98E+05	1.17E+05	1.42E+05	1.20E+05	1.88E+05	1.00E+05	1.28E+05	1.34E+05	1.10E+05	1.26E+05	0.008	0.002	9.54E-06	1.03E-04	0.007	0.003	0.002	2.46E-04	0.007	7.01E-04	8.56E-05	0.019	0.001	2, 4	13, 6	13, 6	MFAP4_MOUSE, SP:Q9D1H9	SP|Q9D1H9|MFAP4_MOUSE
P70695	F16P2_MOUSE	Fructose-1,6-bisphosphatase isozyme 2 OS=Mus musculus GN=Fbp2 PE=2 SV=2	1	6	1	3.71E+04	2.80E+04	3.85E+04	3.37E+04	3.61E+04	3.54E+04	2.34E+04	3.31E+04	1.58E+04	1.60E+04	1.19E+04	1.28E+04	9.39E+03	0.114	0.116	0.025	0.062	0.084	0.082	0.098	0.071	0.163	0.129	0.13	0.239	0.201	4	4	6	F16P2_MOUSE, SP:P70695	SP|P70695|F16P2_MOUSE
Q60841	RELN_MOUSE	Reelin OS=Mus musculus GN=Reln PE=1 SV=2	1	1	1	2.49E+06	2.37E+06	2.50E+06	2.51E+06	2.01E+06	2.37E+06	2.20E+06	2.02E+06	1.67E+06	1.92E+06	2.41E+06	1.70E+06	1.88E+06	5.10E-22	1.55E-26	0	1.55E-36	1.50E-23	3.12E-28	4.38E-33	1.86E-28	7.58E-23	1.86E-32	0	7.16E-16	5.08E-30	4, 7	1	1	RELN_MOUSE, SP:Q60841	SP|Q60841|RELN_MOUSE
Q8C7H1	MMAA_MOUSE	Methylmalonic aciduria type A homolog, mitochondrial OS=Mus musculus GN=Mmaa PE=2 SV=1	1	2	0.99	1.45E+04	9.68E+03	5.26E+03	8.19E+03	3.88E+03	6.39E+03	5.23E+03	7.34E+03	5.65E+03	6.44E+03	3.74E+03	2.82E+03	5.84E+03	0.225	0.241	0.234	0.224	0.326	0.277	0.271	0.245	0.279	0.237	0.264	0.37	0.255	2	2	2	MMAA_MOUSE, SP:Q8C7H1	SP|Q8C7H1|MMAA_MOUSE
Q91VH6	MEMO1_MOUSE	Protein MEMO1 OS=Mus musculus GN=Memo1 PE=1 SV=1	1	1	1	5.69E+04	5.84E+04	3.33E+04	6.57E+04	4.35E+04	5.36E+04	6.50E+04	6.58E+04	7.12E+04	6.14E+04	9.00E+04	6.82E+04	8.20E+04	0.068	0.042	0.035	0.016	0.066	0.043	0.016	0.02	0.019	0.013	0.001	0.051	0.007	5	1	1	MEMO1_MOUSE, SP:Q91VH6	SP|Q91VH6|MEMO1_MOUSE
Q9CY73	RM44_MOUSE	39S ribosomal protein L44, mitochondrial OS=Mus musculus GN=Mrpl44 PE=1 SV=2	1	1	0.98	2.70E+04	2.79E+04	2.90E+04	1.80E+04	2.28E+04	2.09E+04	1.58E+04	2.89E+04	2.53E+04	1.82E+04	1.68E+04	1.26E+04	1.51E+04	0.152	0.117	0.044	0.131	0.137	0.142	0.145	0.086	0.107	0.114	0.091	0.241	0.145	4	1	1	RM44_MOUSE, SP:Q9CY73	SP|Q9CY73|RM44_MOUSE
Q9D404	OXSM_MOUSE	3-oxoacyl-[acyl-carrier-protein] synthase, mitochondrial OS=Mus musculus GN=Oxsm PE=2 SV=1	2	4	1	1.41E+05	1.28E+05	8.37E+04	1.31E+05	1.25E+05	1.26E+05	1.17E+05	9.48E+04	1.02E+05	1.01E+05	9.65E+04	1.01E+05	1.02E+05	0.009	0.005	0.002	0.001	0.005	0.004	0.002	0.007	0.006	0.002	6.98E-04	0.024	0.003	4, 6	1, 4	1, 4	OXSM_MOUSE, SP:Q9D404	SP|Q9D404|OXSM_MOUSE
Q9JIF9	MYOTI_MOUSE	Myotilin OS=Mus musculus GN=Myot PE=2 SV=1	2	3	1	6.26E+04	5.07E+04	4.05E+04	5.44E+04	4.76E+04	6.97E+04	8.78E+04	6.09E+04	1.13E+05	1.06E+05	1.41E+05	1.50E+05	1.85E+05	0.059	0.055	0.025	0.026	0.059	0.026	0.007	0.024	0.005	0.002	6.17E-05	0.008	1.11E-04	3	2, 3	2, 3	MYOTI_MOUSE, SP:Q9JIF9	SP|Q9JIF9|MYOTI_MOUSE
Q9JLT4	TRXR2_MOUSE	Thioredoxin reductase 2, mitochondrial OS=Mus musculus GN=Txnrd2 PE=1 SV=4	2	8	1	6.28E+04	6.87E+04	6.31E+04	4.04E+04	4.95E+04	5.90E+04	6.82E+04	4.69E+04	4.40E+04	7.09E+04	4.64E+04	4.17E+04	5.02E+04	0.058	0.031	0.006	0.046	0.054	0.036	0.014	0.041	0.051	0.009	0.013	0.101	0.027	2, 3	7, 8	7, 8	TRXR2_MOUSE, SP:Q9JLT4	SP|Q9JLT4|TRXR2_MOUSE
Q9ERP3	TRI54_MOUSE	Tripartite motif-containing protein 54 OS=Mus musculus GN=Trim54 PE=1 SV=1	1	5	1	6.15E+04	5.24E+04	5.60E+04	5.17E+04	5.33E+04	5.45E+04	5.50E+04	7.37E+04	5.39E+04	6.67E+04	8.40E+04	7.14E+04	7.07E+04	0.061	0.053	0.01	0.03	0.049	0.044	0.025	0.015	0.037	0.012	0.002	0.049	0.012	4	5	5	TRI54_MOUSE, SP:Q9ERP3	SP|Q9ERP3|TRI54_MOUSE
P47811	MK14_MOUSE	Mitogen-activated protein kinase 14 OS=Mus musculus GN=Mapk14 PE=1 SV=3	1	4	0.96	3.76E+04	3.79E+04	3.21E+04	3.67E+04	2.68E+04	3.77E+04	3.85E+04	3.89E+04	3.22E+04	3.19E+04	3.35E+04	3.93E+04	2.63E+04	0.113	0.083	0.037	0.055	0.119	0.076	0.05	0.057	0.081	0.056	0.03	0.108	0.082	5	4	4	MK14_MOUSE, SP:P47811	SP|P47811|MK14_MOUSE
Q3URS9	CCD51_MOUSE	Coiled-coil domain-containing protein 51 OS=Mus musculus GN=Ccdc51 PE=2 SV=1	1	7	0.99	5.95E+04	3.65E+04	1.29E+04	2.82E+04	1.99E+04	3.27E+04	2.73E+04	2.18E+04	3.29E+04	2.67E+04	2.65E+04	2.99E+04	2.58E+04	0.063	0.086	0.128	0.08	0.153	0.09	0.082	0.117	0.078	0.072	0.047	0.14	0.083	4	7	7	CCD51_MOUSE, SP:Q3URS9	SP|Q3URS9|CCD51_MOUSE
Q8QZR5	ALAT1_MOUSE	Alanine aminotransferase 1 OS=Mus musculus GN=Gpt PE=2 SV=3	2	1	1	8.51E+04	7.28E+04	8.89E+04	9.55E+04	6.71E+04	7.75E+04	9.68E+04	6.86E+04	6.18E+04	7.05E+04	7.53E+04	4.70E+04	1.23E+05	0.034	0.027	0.001	0.005	0.031	0.02	0.004	0.018	0.026	0.009	0.002	0.088	0.001	2, 5	1	1	ALAT1_MOUSE, SP:Q8QZR5	SP|Q8QZR5|ALAT1_MOUSE
P21614	VTDB_MOUSE	Vitamin D-binding protein OS=Mus musculus GN=Gc PE=1 SV=2	3	8	1	2.89E+05	2.33E+05	1.77E+05	2.02E+05	1.74E+05	2.71E+05	2.11E+05	2.31E+05	2.45E+05	1.89E+05	1.78E+05	3.06E+05	2.22E+05	3.84E-04	2.90E-04	1.34E-05	8.39E-05	0.001	5.86E-05	5.65E-05	5.68E-05	5.70E-05	5.30E-05	6.96E-06	2.75E-04	2.53E-05	3, 5	1, 7, 8	1, 7, 8	VTDB_MOUSE, SP:P21614	SP|P21614|VTDB_MOUSE
P31428	DPEP1_MOUSE	Dipeptidase 1 OS=Mus musculus GN=Dpep1 PE=1 SV=1	1	2	0.99	5.77E+04	4.83E+04	6.74E+04	6.03E+04	5.24E+04	6.94E+04	6.58E+04	3.83E+04	4.37E+04	6.25E+04	7.31E+04	6.20E+04	7.03E+04	0.067	0.059	0.005	0.02	0.049	0.026	0.016	0.058	0.052	0.013	0.003	0.06	0.011	5	2	2	DPEP1_MOUSE, SP:P31428	SP|P31428|DPEP1_MOUSE
Q9DBI2	BBS10_MOUSE	Bardet-Biedl syndrome 10 protein homolog OS=Mus musculus GN=Bbs10 PE=2 SV=2	1	2	0.53	1.18E+05	1.03E+05	2.37E+05	1.51E+05	4.53E+05	3.91E+05	2.63E+05	1.24E+05	4.92E+05	4.25E+05	4.11E+05	5.58E+05	2.75E+05	0.016	0.011	6.33E-07	5.93E-04	6.31E-07	2.04E-06	8.40E-06	0.002	2.66E-08	3.75E-09	3.08E-11	1.67E-06	3.37E-06	8	2	2	BBS10_MOUSE, SP:Q9DBI2	SP|Q9DBI2|BBS10_MOUSE
Q3UHZ5	LMOD2_MOUSE	Leiomodin-2 OS=Mus musculus GN=Lmod2 PE=1 SV=1	2	3	1	5.06E+04	3.99E+04	6.79E+04	4.60E+04	1.10E+05	8.01E+04	7.77E+04	5.35E+04	1.49E+05	1.17E+05	1.47E+05	1.42E+05	9.71E+04	0.08	0.078	0.005	0.037	0.008	0.018	0.009	0.031	0.001	0.001	4.02E-05	0.009	0.004	2, 4	1, 3	1, 3	LMOD2_MOUSE, SP:Q3UHZ5	SP|Q3UHZ5|LMOD2_MOUSE
P40336	VP26A_MOUSE	Vacuolar protein sorting-associated protein 26A OS=Mus musculus GN=Vps26a PE=2 SV=1	1	8	1	3.66E+04	3.50E+04	3.23E+04	2.82E+04	3.69E+04	1.71E+04	3.58E+04	2.50E+04	2.98E+04	2.40E+04	2.94E+04	2.99E+04	3.41E+04	0.117	0.092	0.037	0.082	0.083	0.168	0.056	0.104	0.09	0.085	0.04	0.141	0.057	2	8	8	VP26A_MOUSE, SP:P40336	SP|P40336|VP26A_MOUSE
P01873	MUCM_MOUSE	Ig mu chain C region membrane-bound form OS=Mus musculus GN=Igh-6 PE=2 SV=3	1	1	0.99	3.53E+02	8.31E+03	0.00E+00	1.04E+04	7.51E+03	6.28E+03	1.46E+04	8.53E+03	5.04E+03	3.03E+03	6.22E+03	8.94E+03	9.86E+03	0.453	0.257	0.5	0.197	0.265	0.278	0.154	0.228	0.29	0.311	0.208	0.277	0.196	2	1	1	MUCM_MOUSE, SP:P01873	SP|P01873|MUCM_MOUSE,SP|P01872|IGHM_MOUSE
Q14C51	PTCD3_MOUSE	Pentatricopeptide repeat-containing protein 3, mitochondrial OS=Mus musculus GN=Ptcd3 PE=2 SV=2	1	6	1	1.92E+04	1.17E+04	2.03E+04	1.98E+04	6.21E+03	1.02E+04	1.44E+04	2.20E+04	1.18E+04	1.21E+04	1.51E+04	1.32E+04	1.46E+04	0.193	0.22	0.077	0.119	0.284	0.227	0.156	0.116	0.198	0.163	0.102	0.236	0.148	2	6	6	PTCD3_MOUSE, SP:Q14C51	SP|Q14C51|PTCD3_MOUSE
P32261	ANT3_MOUSE	Antithrombin-III OS=Mus musculus GN=Serpinc1 PE=1 SV=1	2	2	1	3.39E+04	3.01E+04	4.22E+04	3.80E+04	5.02E+04	4.82E+04	5.45E+04	2.95E+04	3.92E+04	4.05E+04	5.43E+04	3.54E+04	5.17E+04	0.125	0.109	0.021	0.052	0.053	0.052	0.025	0.084	0.062	0.037	0.009	0.12	0.025	2	1, 2	1, 2	ANT3_MOUSE, SP:P32261	SP|P32261|ANT3_MOUSE
O08677	KNG1_MOUSE	Kininogen-1 OS=Mus musculus GN=Kng1 PE=1 SV=1	3	5	1	2.93E+04	4.37E+04	7.07E+04	5.46E+04	4.25E+04	6.03E+04	8.11E+04	4.64E+04	4.74E+04	8.59E+04	6.09E+04	6.08E+04	6.52E+04	0.142	0.068	0.004	0.025	0.068	0.035	0.008	0.042	0.045	0.004	0.006	0.062	0.014	2	1, 5	1, 5	KNG1_MOUSE, SP:O08677	SP|O08677|KNG1_MOUSE
Q8BH86	CN159_MOUSE	UPF0317 protein C14orf159 homolog, mitochondrial OS=Mus musculus PE=2 SV=1	3	12	1	1.56E+05	1.44E+05	1.62E+05	1.68E+05	1.37E+05	1.51E+05	1.69E+05	1.38E+05	9.13E+04	9.98E+04	1.08E+05	8.17E+04	6.89E+04	0.007	0.003	3.09E-05	3.11E-04	0.004	0.002	2.69E-04	0.001	0.009	0.002	3.66E-04	0.037	0.012	3, 4	1, 10, 12	1, 10, 12	CN159_MOUSE, SP:Q8BH86	SP|Q8BH86|CN159_MOUSE
Q9D2N4	DTNA_MOUSE	Dystrobrevin alpha OS=Mus musculus GN=Dtna PE=1 SV=1	1	2	0.99	1.13E+04	1.08E+04	6.19E+03	1.29E+04	1.20E+04	9.89E+03	1.31E+04	6.84E+03	8.60E+03	6.97E+03	8.69E+03	8.55E+03	9.27E+03	0.253	0.229	0.216	0.171	0.214	0.231	0.167	0.253	0.234	0.228	0.168	0.281	0.203	3	2	2	DTNA_MOUSE, SP:Q9D2N4	SP|Q9D2N4|DTNA_MOUSE
Q8BMF3	MAON_MOUSE	NADP-dependent malic enzyme, mitochondrial OS=Mus musculus GN=Me3 PE=1 SV=1	4	9	1	6.77E+05	6.99E+05	5.16E+05	6.67E+05	6.12E+05	6.56E+05	6.05E+05	6.02E+05	4.46E+05	4.48E+05	5.63E+05	5.73E+05	5.56E+05	1.37E-07	1.41E-09	5.59E-13	5.34E-12	9.28E-09	1.22E-09	3.94E-11	2.72E-10	1.14E-07	1.55E-09	1.12E-14	1.24E-06	8.48E-11	2, 3, 5	1, 4, 9, 5, 7	1, 4, 9, 5, 7	MAON_MOUSE, SP:Q8BMF3	SP|Q8BMF3|MAON_MOUSE
Q3UV70	PDP1_MOUSE	[Pyruvate dehydrogenase [acetyl-transferring]]-phosphatase 1, mitochondrial OS=Mus musculus GN=Pdp1 PE=2 SV=1	1	8	1	7.80E+03	4.96E+03	9.01E+03	1.48E+04	6.09E+03	7.27E+03	1.23E+04	3.78E+03	8.41E+03	1.09E+04	8.64E+03	2.54E+03	1.64E+04	0.29	0.307	0.171	0.154	0.286	0.264	0.175	0.31	0.237	0.175	0.169	0.376	0.135	2	8	8	PDP1_MOUSE, SP:Q3UV70	SP|Q3UV70|PDP1_MOUSE
Q9DBF1	AL7A1_MOUSE	Alpha-aminoadipic semialdehyde dehydrogenase OS=Mus musculus GN=Aldh7a1 PE=2 SV=3	1	5	0.99	4.36E+04	3.51E+04	3.18E+04	2.56E+04	3.19E+04	3.60E+04	3.04E+04	2.74E+04	2.66E+04	3.01E+04	4.21E+04	3.27E+04	3.87E+04	0.095	0.091	0.038	0.091	0.099	0.081	0.072	0.092	0.103	0.063	0.019	0.129	0.045	4	5	5	AL7A1_MOUSE, SP:Q9DBF1	SP|Q9DBF1|AL7A1_MOUSE
Q03734	SPA3M_MOUSE	Serine protease inhibitor A3M OS=Mus musculus GN=Serpina3m PE=1 SV=1	1	13	1	4.66E+05	3.31E+05	3.78E+05	4.73E+05	3.18E+05	5.05E+05	3.55E+05	5.25E+05	5.00E+05	2.45E+05	2.42E+05	3.96E+05	3.51E+05	1.00E-05	2.05E-05	4.79E-10	5.10E-09	2.33E-05	7.94E-08	2.82E-07	3.38E-09	1.95E-08	5.06E-06	2.17E-07	4.29E-05	1.78E-07	5, 6	13	13	SPA3M_MOUSE, SP:Q03734	SP|Q03734|SPA3M_MOUSE
Q8VBT1	TXLNB_MOUSE	Beta-taxilin OS=Mus musculus GN=Txlnb PE=2 SV=2	2	8	1	8.39E+04	8.32E+04	8.73E+04	1.04E+05	1.25E+05	1.15E+05	9.48E+04	9.56E+04	1.08E+05	1.16E+05	1.17E+05	1.50E+05	1.26E+05	0.035	0.02	0.002	0.003	0.005	0.006	0.005	0.006	0.005	0.001	2.27E-04	0.008	0.001	4, 5	3, 8	3, 8	TXLNB_MOUSE, SP:Q8VBT1	SP|Q8VBT1|TXLNB_MOUSE
P10637	TAU_MOUSE	Microtubule-associated protein tau OS=Mus musculus GN=Mapt PE=1 SV=3	1	2	0.99	1.60E+04	1.85E+04	1.21E+04	1.26E+04	1.75E+04	1.56E+04	1.92E+04	2.42E+04	2.53E+04	2.17E+04	1.97E+04	2.55E+04	1.89E+04	0.214	0.166	0.136	0.174	0.169	0.178	0.122	0.105	0.107	0.094	0.074	0.159	0.118	3	2	2	TAU_MOUSE, SP:P10637	SP|P10637|TAU_MOUSE
Q8K411	PREP_MOUSE	Presequence protease, mitochondrial OS=Mus musculus GN=Pitrm1 PE=2 SV=1	2	2	1	5.20E+04	3.84E+04	1.61E+05	4.91E+04	5.42E+04	5.78E+04	6.09E+04	3.88E+04	5.02E+04	6.05E+04	5.85E+04	5.28E+04	4.56E+04	0.077	0.081	3.26E-05	0.032	0.046	0.038	0.019	0.057	0.04	0.014	0.006	0.075	0.033	2, 4	1, 2	1, 2	PREP_MOUSE, SP:Q8K411	SP|Q8K411|PREP_MOUSE
Q8CC35	SYNPO_MOUSE	Synaptopodin OS=Mus musculus GN=Synpo PE=1 SV=2	2	5	1	4.54E+04	4.06E+04	5.77E+04	5.57E+04	5.20E+04	3.58E+04	3.96E+04	3.36E+04	4.12E+04	4.87E+04	5.95E+04	6.62E+04	7.73E+04	0.092	0.077	0.008	0.025	0.05	0.082	0.048	0.072	0.058	0.025	0.007	0.055	0.009	3, 4	2, 5	2, 5	SYNPO_MOUSE, SP:Q8CC35	SP|Q8CC35|SYNPO_MOUSE
O88492	PLIN4_MOUSE	Perilipin-4 OS=Mus musculus GN=Plin4 PE=1 SV=1	5	11	1	3.18E+06	2.49E+06	7.68E+05	1.81E+06	7.63E+05	9.76E+05	1.22E+06	1.02E+06	9.67E+05	1.19E+06	1.32E+06	1.03E+06	1.10E+06	1.10E-27	3.80E-28	4.45E-18	4.36E-28	1.97E-10	2.62E-13	3.86E-20	6.64E-16	1.90E-14	6.46E-22	1.68E-30	1.69E-10	3.57E-19	2, 3, 8	1, 11, 3, 6, 9	1, 10, 11, 3, 6	PLIN4_MOUSE, SP:O88492	SP|O88492|PLIN4_MOUSE
Q7TSQ8	PDPR_MOUSE	Pyruvate dehydrogenase phosphatase regulatory subunit, mitochondrial OS=Mus musculus GN=Pdpr PE=2 SV=1	1	1	0.59	1.92E+04	1.58E+04	3.09E+04	5.02E+04	2.08E+04	2.06E+04	3.87E+04	1.77E+04	2.73E+04	1.76E+04	2.56E+04	3.15E+04	3.13E+04	0.193	0.185	0.039	0.03	0.148	0.144	0.049	0.142	0.098	0.118	0.049	0.134	0.064	3	1	1	PDPR_MOUSE, SP:Q7TSQ8	SP|Q7TSQ8|PDPR_MOUSE
Q65CL1	CTNA3_MOUSE	Catenin alpha-3 OS=Mus musculus GN=Ctnna3 PE=1 SV=2	3	6	1	2.43E+05	2.52E+05	1.93E+05	2.05E+05	2.34E+05	1.98E+05	1.61E+05	1.89E+05	2.39E+05	1.81E+05	1.41E+05	1.83E+05	1.34E+05	0.001	1.74E-04	6.40E-06	7.85E-05	2.37E-04	5.17E-04	3.64E-04	2.41E-04	6.93E-05	7.39E-05	5.81E-05	0.004	8.10E-04	4, 5, 6	2, 5, 6	2, 5, 6	CTNA3_MOUSE, SP:Q65CL1	SP|Q65CL1|CTNA3_MOUSE
Q8VDC0	SYLM_MOUSE	Probable leucyl-tRNA synthetase, mitochondrial OS=Mus musculus GN=Lars2 PE=2 SV=1	2	2	1	4.31E+04	3.31E+04	3.85E+04	3.68E+04	4.66E+04	2.49E+04	2.78E+04	4.85E+04	2.37E+04	3.83E+04	3.60E+04	4.15E+04	3.25E+04	0.097	0.097	0.026	0.055	0.06	0.122	0.081	0.039	0.116	0.041	0.026	0.102	0.061	2	1, 2	1, 2	SYLM_MOUSE, SP:Q8VDC0	SP|Q8VDC0|SYLM_MOUSE
Q9ET54	PALLD_MOUSE	Palladin OS=Mus musculus GN=Palld PE=1 SV=2	1	2	0.67	1.74E+04	1.22E+04	4.65E+03	8.79E+03	8.70E+03	7.60E+03	1.20E+04	1.43E+04	1.72E+04	1.49E+04	1.27E+04	1.12E+04	1.39E+04	0.205	0.217	0.249	0.218	0.251	0.26	0.179	0.169	0.154	0.139	0.123	0.255	0.155	3	2	2	PALLD_MOUSE, SP:Q9ET54	SP|Q9ET54|PALLD_MOUSE
Q8R2Q4	RRF2M_MOUSE	Ribosome-releasing factor 2, mitochondrial OS=Mus musculus GN=Gfm2 PE=2 SV=2	1	4	1	4.24E+04	4.21E+04	3.99E+04	3.57E+04	4.30E+04	3.69E+04	2.73E+04	3.38E+04	3.86E+04	3.28E+04	3.14E+04	3.71E+04	4.03E+04	0.098	0.072	0.024	0.057	0.067	0.077	0.082	0.07	0.063	0.053	0.034	0.114	0.042	4	4	4	RRF2M_MOUSE, SP:Q8R2Q4	SP|Q8R2Q4|RRF2M_MOUSE
O55111	DSG2_MOUSE	Desmoglein-2 OS=Mus musculus GN=Dsg2 PE=1 SV=2	1	2	1	1.61E+04	2.19E+04	1.87E+04	1.58E+04	1.94E+04	1.64E+04	2.03E+04	1.58E+04	1.82E+04	1.38E+04	1.95E+04	1.59E+04	1.48E+04	0.213	0.145	0.086	0.146	0.157	0.172	0.115	0.156	0.147	0.147	0.075	0.215	0.147	2	2	2	DSG2_MOUSE, SP:O55111	SP|O55111|DSG2_MOUSE
Q9WU79	PROD_MOUSE	Proline dehydrogenase, mitochondrial OS=Mus musculus GN=Prodh PE=2 SV=1	1	3	0.99	4.22E+04	3.57E+04	2.55E+04	2.73E+04	3.18E+04	2.55E+04	2.79E+04	3.35E+04	2.13E+04	3.21E+04	2.05E+04	1.69E+04	1.72E+04	0.099	0.088	0.055	0.083	0.098	0.118	0.079	0.07	0.127	0.055	0.07	0.208	0.129	3	3	3	PROD_MOUSE, SP:Q9WU79	SP|Q9WU79|PROD_MOUSE
Q9CQL5	RM18_MOUSE	39S ribosomal protein L18, mitochondrial OS=Mus musculus GN=Mrpl18 PE=2 SV=1	1	1	0.95	8.29E+03	1.15E+04	1.52E+04	1.40E+04	1.16E+04	1.01E+04	1.45E+04	2.26E+04	1.57E+04	9.90E+03	9.89E+03	5.20E+03	1.20E+04	0.284	0.222	0.109	0.161	0.217	0.228	0.155	0.113	0.164	0.187	0.153	0.326	0.173	3	1	1	RM18_MOUSE, SP:Q9CQL5	SP|Q9CQL5|RM18_MOUSE
Q9QZ57	HSPB3_MOUSE	Heat shock protein beta-3 OS=Mus musculus GN=Hspb3 PE=2 SV=1	1	2	0.96	7.58E+03	1.43E+04	1.78E+04	1.78E+04	1.76E+04	1.68E+04	1.73E+04	2.56E+04	1.94E+04	2.92E+04	2.26E+04	2.85E+04	2.24E+04	0.293	0.197	0.092	0.132	0.169	0.168	0.134	0.099	0.139	0.063	0.061	0.145	0.099	2	2	2	HSPB3_MOUSE, SP:Q9QZ57	SP|Q9QZ57|HSPB3_MOUSE
Q9D7P6	ISCU_MOUSE	Iron-sulfur cluster assembly enzyme ISCU, mitochondrial OS=Mus musculus GN=Iscu PE=1 SV=1	1	1	0.99	8.75E+03	3.64E+03	2.14E+04	7.31E+03	4.24E+04	1.99E+04	2.52E+04	9.19E+03	2.60E+04	2.94E+04	2.25E+04	2.97E+04	2.04E+04	0.279	0.334	0.072	0.237	0.068	0.148	0.09	0.22	0.104	0.063	0.061	0.14	0.109	5	1	1	ISCU_MOUSE, SP:Q9D7P6	SP|Q9D7P6|ISCU_MOUSE
Q920Q6	MSI2H_MOUSE	RNA-binding protein Musashi homolog 2 OS=Mus musculus GN=Msi2 PE=1 SV=1	1	2	0.97	2.51E+04	1.97E+04	2.71E+04	1.92E+04	2.34E+04	2.87E+04	2.03E+04	2.66E+04	2.28E+04	2.44E+04	3.02E+04	2.20E+04	2.51E+04	0.161	0.159	0.05	0.124	0.135	0.105	0.116	0.096	0.12	0.082	0.038	0.177	0.086	5	2	2	MSI2H_MOUSE, SP:Q920Q6	SP|Q920Q6|MSI2H_MOUSE
Q8C0M9	ASGL1_MOUSE	L-asparaginase OS=Mus musculus GN=Asrgl1 PE=1 SV=1	1	3	1	1.39E+04	1.48E+04	2.03E+04	2.24E+04	1.64E+04	2.29E+04	2.49E+04	2.10E+04	2.03E+04	1.61E+04	2.40E+04	1.58E+04	2.44E+04	0.23	0.193	0.077	0.105	0.177	0.131	0.092	0.122	0.133	0.129	0.055	0.216	0.089	2	3	3	ASGL1_MOUSE, SP:Q8C0M9	SP|Q8C0M9|ASGL1_MOUSE
P60605	UB2G2_MOUSE	Ubiquitin-conjugating enzyme E2 G2 OS=Mus musculus GN=Ube2g2 PE=1 SV=1	1	12	0.64	5.76E+04	4.44E+04	5.59E+04	6.23E+04	3.00E+04	3.96E+04	6.21E+04	4.05E+04	6.22E+04	8.79E+04	3.30E+04	2.69E+04	4.74E+04	0.066	0.066	0.009	0.018	0.105	0.07	0.018	0.053	0.026	0.004	0.031	0.152	0.03	3	12	12	UB2G2_MOUSE, SP:P60605	SP|P60605|UB2G2_MOUSE
Q3TBW2	RM10_MOUSE	39S ribosomal protein L10, mitochondrial OS=Mus musculus GN=Mrpl10 PE=2 SV=2	1	3	0.98	3.18E+04	3.50E+04	2.94E+04	3.35E+04	2.50E+04	3.21E+04	2.05E+04	2.99E+04	2.69E+04	2.31E+04	2.93E+04	3.20E+04	2.29E+04	0.145	0.101	0.053	0.081	0.142	0.104	0.127	0.095	0.115	0.111	0.066	0.142	0.127	2	3	3	RM10_MOUSE, SP:Q3TBW2	SP|Q3TBW2|RM10_MOUSE
Q61036	PAK3_MOUSE	Serine/threonine-protein kinase PAK 3 OS=Mus musculus GN=Pak3 PE=1 SV=2	1	2	0.62	2.66E+04	3.81E+04	3.27E+04	2.45E+04	1.98E+04	6.00E+04	2.35E+04	3.46E+04	2.70E+04	1.93E+04	2.67E+04	1.87E+04	4.75E+04	0.155	0.083	0.036	0.097	0.157	0.035	0.1	0.068	0.101	0.11	0.05	0.003	0.031	5	2	2	PAK3_MOUSE, SP:Q61036	SP|Q61036|PAK3_MOUSE
P97493	THIOM_MOUSE	Thioredoxin, mitochondrial OS=Mus musculus GN=Txn2 PE=2 SV=1	1	5	0.99	1.55E+04	1.66E+04	9.09E+04	1.47E+04	1.13E+05	1.01E+05	1.10E+05	2.05E+04	8.99E+04	9.43E+04	1.14E+05	1.02E+05	6.88E+04	0.219	0.18	0.001	0.157	0.008	0.009	0.003	0.125	0.01	0.003	2.71E-04	0.023	0.012	3	5	5	THIOM_MOUSE, SP:P97493	SP|P97493|THIOM_MOUSE
P03893	NU2M_MOUSE	NADH-ubiquinone oxidoreductase chain 2 OS=Mus musculus GN=Mtnd2 PE=1 SV=1	3	12	1	1.00E+06	7.54E+05	5.78E+05	8.01E+05	7.76E+05	6.34E+05	6.25E+05	6.37E+05	6.64E+05	6.80E+05	4.96E+05	5.95E+05	5.00E+05	2.11E-10	3.17E-10	1.89E-14	4.49E-14	1.01E-10	2.02E-09	1.53E-11	7.57E-11	1.16E-10	1.06E-13	2.39E-13	7.22E-07	5.82E-10	3, 4, 4, 7	10, 12	10, 12	NU2M_MOUSE, SP:P03893	SP|P03893|NU2M_MOUSE
Q9D9R9	F186A_MOUSE	Protein FAM186A OS=Mus musculus GN=FAM186A PE=2 SV=2	1	11	1	7.00E+04	1.84E+05	2.42E+05	1.79E+05	1.36E+05	1.63E+05	1.65E+05	1.89E+05	2.13E+05	2.33E+05	2.16E+05	1.03E+05	2.72E+05	0.05	0.001	5.80E-07	2.08E-04	0.004	0.001	3.46E-04	2.46E-04	1.69E-04	9.55E-06	1.18E-06	0.023	4.38E-06	4	11	11	F186A_MOUSE, SP:Q9D9R9	SP|Q9D9R9|F186A_MOUSE
Q91WG8	GLCNE_MOUSE	Bifunctional UDP-N-acetylglucosamine 2-epimerase/N-acetylmannosamine kinase OS=Mus musculus GN=Gne PE=1 SV=1	1	5	0.97	0.00E+00	3.06E+04	1.70E+04	2.19E+04	1.24E+04	1.53E+04	2.63E+04	2.84E+04	2.57E+04	1.61E+04	2.12E+04	7.63E+03	2.02E+04	0.5	0.106	0.096	0.107	0.21	0.179	0.086	0.087	0.105	0.128	0.066	0.292	0.11	3	5	5	GLCNE_MOUSE, SP:Q91WG8	SP|Q91WG8|GLCNE_MOUSE

Q6PD03	2A5A_MOUSE	Serine/threonine-protein phosphatase 2A 56 kDa regulatory subunit alpha isoform OS=Mus musculus GN=Ppp2r5a PE=1 SV=1	1	3	0.99	2.01E+04	2.68E+04	2.46E+04	3.13E+04	2.56E+04	2.17E+04	3.02E+04	3.25E+04	2.15E+04	2.06E+04	2.43E+04	1.94E+04	1.65E+04	0.188	0.122	0.059	0.07	0.125	0.138	0.072	0.074	0.128	0.101	0.055	0.192	0.135	2	3	3	2A5A_MOUSE, SP:Q6PD03	SP|Q6PD03|2A5A_MOUSE
Q9DBH5	LMAN2_MOUSE	Vesicular integral-membrane protein VIP36 OS=Mus musculus GN=Lman2 PE=2 SV=1	1	4	0.91	8.78E+04	9.94E+04	7.80E+04	7.10E+04	8.37E+04	9.77E+04	8.41E+04	9.12E+04	9.23E+04	8.89E+04	7.88E+04	9.26E+04	7.26E+04	0.032	0.012	0.003	0.013	0.018	0.01	0.007	0.008	0.009	0.004	0.002	0.029	0.01	3	4	4	LMAN2_MOUSE, SP:Q9DBH5	SP|Q9DBH5|LMAN2_MOUSE
Q6PDH0	PHLB1_MOUSE	Pleckstrin homology-like domain family B member 1 OS=Mus musculus GN=Phldb1 PE=1 SV=1	1	7	0.64	1.10E+05	1.18E+05	1.05E+05	1.39E+05	8.31E+04	1.32E+05	9.92E+04	1.30E+05	9.26E+04	1.17E+05	8.53E+04	6.29E+04	8.59E+04	0.02	0.007	6.93E-04	9.66E-04	0.019	0.004	0.004	0.002	0.009	0.001	0.001	0.06	0.006	5	7	7	PHLB1_MOUSE, SP:Q6PDH0	SP|Q6PDH0|PHLB1_MOUSE
P46978	STT3A_MOUSE	Dolichyl-diphosphooligosaccharide--protein glycosyltransferase subunit STT3A OS=Mus musculus GN=Stt3a PE=1 SV=1	1	5	0.87	1.16E+04	5.19E+03	1.21E+05	3.55E+04	9.38E+04	5.94E+04	1.33E+05	2.44E+04	1.38E+05	1.31E+05	8.17E+04	7.10E+04	6.34E+04	0.252	0.316	3.06E-04	0.063	0.014	0.038	0.001	0.111	0.002	6.75E-04	0.002	0.048	0.016	3	5	5	STT3A_MOUSE, SP:P46978	SP|P46978|STT3A_MOUSE
Q9ES83	POPD1_MOUSE	Blood vessel epicardial substance OS=Mus musculus GN=Bves PE=2 SV=1	2	8	1	2.42E+05	1.96E+05	1.08E+05	1.56E+05	1.66E+05	1.50E+05	1.44E+05	1.46E+05	1.47E+05	1.45E+05	1.42E+05	1.56E+05	1.78E+05	0.001	7.99E-04	5.21E-04	4.81E-04	0.002	0.002	7.02E-04	0.001	0.001	3.35E-04	5.22E-05	0.007	1.39E-04	3, 5	6, 8	6, 8	POPD1_MOUSE, SP:Q9ES83	SP|Q9ES83|POPD1_MOUSE
Q9CQB5	CISD2_MOUSE	CDGSH iron sulfur domain-containing protein 2 OS=Mus musculus GN=Cisd2 PE=1 SV=1	1	5	0.69	8.12E+03	8.65E+03	1.62E+04	1.47E+04	1.63E+04	1.36E+04	2.00E+04	2.09E+04	1.62E+04	8.56E+03	1.17E+04	1.76E+04	1.95E+04	0.286	0.253	0.105	0.155	0.178	0.194	0.117	0.122	0.16	0.204	0.132	0.204	0.114	2	5	5	CISD2_MOUSE, SP:Q9CQB5	SP|Q9CQB5|CISD2_MOUSE
Q00896	A1AT1_MOUSE	Alpha-1-antitrypsin 1-3 OS=Mus musculus GN=Serpina1c PE=1 SV=2	1	13	1	3.80E+05	2.62E+05	1.91E+05	2.71E+05	1.24E+05	3.45E+05	2.88E+05	2.21E+05	3.41E+05	3.07E+05	2.12E+05	2.73E+05	2.42E+05	6.03E-05	1.37E-04	6.81E-06	7.09E-06	0.005	7.44E-06	3.43E-06	8.01E-05	2.96E-06	4.53E-07	1.19E-06	5.60E-04	1.23E-05	3, 5	5, 13	13, 5	A1AT1_MOUSE, SP:P07758	SP|P07758|A1AT1_MOUSE,SP|Q00896|A1AT3_MOUSE
Q9CXU9	EIF1B_MOUSE	Eukaryotic translation initiation factor 1b OS=Mus musculus GN=Eif1b PE=2 SV=2	1	12	0.99	5.60E+04	6.21E+04	4.24E+04	4.34E+04	6.70E+04	4.93E+04	4.18E+04	5.94E+04	4.46E+04	3.87E+04	4.77E+04	4.56E+04	5.00E+04	0.07	0.038	0.02	0.041	0.031	0.05	0.043	0.025	0.05	0.04	0.012	0.091	0.027	3	12	12	EIF1B_MOUSE, SP:Q9CXU9	SP|Q9CXU9|EIF1B_MOUSE,SP|P48024|EIF1_MOUSE
P28653	PGS1_MOUSE	Biglycan OS=Mus musculus GN=Bgn PE=2 SV=1	2	6	1	6.62E+04	5.74E+04	7.82E+04	7.20E+04	6.58E+04	7.52E+04	7.37E+04	8.53E+04	1.31E+05	1.48E+05	1.42E+05	1.23E+05	1.85E+05	0.054	0.044	0.003	0.013	0.032	0.021	0.011	0.01	0.002	3.03E-04	5.76E-05	0.014	1.09E-04	4	1, 6	1, 6	PGS1_MOUSE, SP:P28653	SP|P28653|PGS1_MOUSE
Q61581	IBP7_MOUSE	Insulin-like growth factor-binding protein 7 OS=Mus musculus GN=Igfbp7 PE=2 SV=3	1	2	0.56	4.16E+04	4.12E+04	7.68E+04	9.32E+04	2.54E+04	5.10E+04	4.52E+04	4.18E+04	3.80E+04	5.25E+04	5.42E+04	2.33E+04	4.58E+04	0.101	0.074	0.003	0.005	0.124	0.047	0.037	0.05	0.064	0.02	0.008	0.17	0.032	3	2	2	IBP7_MOUSE, SP:Q61581	SP|Q61581|IBP7_MOUSE
Q9QUH0	GLRX1_MOUSE	Glutaredoxin-1 OS=Mus musculus GN=Glrx PE=1 SV=3	2	12	1	1.42E+05	1.78E+05	1.70E+05	1.59E+05	1.68E+05	1.45E+05	1.20E+05	1.55E+05	1.63E+05	1.60E+05	1.32E+05	1.11E+05	1.15E+05	0.009	0.001	2.01E-05	4.35E-04	0.002	0.002	0.002	8.00E-04	8.33E-04	1.89E-04	1.00E-04	0.018	0.002	5	1, 12	1, 12	GLRX1_MOUSE, SP:Q9QUH0	SP|Q9QUH0|GLRX1_MOUSE
Q62383	SPT6H_MOUSE	Transcription elongation factor SPT6 OS=Mus musculus GN=Supt6h PE=1 SV=2	1	6	0.58	1.05E+05	1.49E+05	1.47E+04	7.02E+04	4.58E+04	4.64E+04	4.33E+04	7.93E+04	2.48E+04	2.56E+04	2.80E+04	3.62E+04	4.84E+04	0.021	0.003	0.124	0.014	0.061	0.056	0.043	0.012	0.11	0.079	0.044	0.118	0.032	2	6	6	SPT6H_MOUSE, SP:Q62383	SP|Q62383|SPT6H_MOUSE
Q7TST5	LAMP3_MOUSE	Lysosome-associated membrane glycoprotein 3 OS=Mus musculus GN=Lamp3 PE=2 SV=1	1	6	0.59	5.55E+05	4.68E+05	2.02E+05	3.55E+05	3.91E+05	3.38E+05	2.88E+05	3.77E+05	4.03E+05	2.89E+05	2.70E+05	4.41E+05	3.08E+05	1.71E-06	5.89E-07	3.84E-06	3.46E-07	3.29E-06	8.91E-06	3.33E-06	4.42E-07	4.14E-07	8.62E-07	4.99E-08	1.76E-05	9.70E-07	6	6	6	LAMP3_MOUSE, SP:Q7TST5	SP|Q7TST5|LAMP3_MOUSE
Q9CY27	TECR_MOUSE	Trans-2,3-enoyl-CoA reductase OS=Mus musculus GN=Tecr PE=1 SV=1	2	11	1	1.83E+04	1.94E+04	4.09E+04	2.68E+04	2.89E+04	2.04E+04	3.90E+04	3.16E+04	2.84E+04	1.25E+04	1.69E+04	5.71E+03	2.44E+04	0.198	0.16	0.022	0.085	0.109	0.145	0.048	0.076	0.094	0.158	0.09	0.318	0.089	2	11, 4	11, 4	TECR_MOUSE, SP:Q9CY27	SP|Q9CY27|TECR_MOUSE
P01864	GCAB_MOUSE	Ig gamma-2A chain C region secreted form OS=Mus musculus PE=1 SV=1	1	7	1	1.87E+04	2.53E+04	1.88E+04	4.40E+04	1.59E+04	7.38E+04	7.36E+04	1.95E+04	1.65E+04	6.53E+04	5.19E+04	3.59E+04	7.09E+04	0.196	0.128	0.085	0.039	0.181	0.022	0.011	0.13	0.158	0.011	0.009	0.118	0.011	5	7	7	GCAB_MOUSE, SP:P01864	SP|P01864|GCAB_MOUSE
O35598	ADA10_MOUSE	Disintegrin and metalloproteinase domain-containing protein 10 OS=Mus musculus GN=Adam10 PE=1 SV=1	1	11	0.71	5.71E+06	5.32E+06	2.57E+06	5.64E+06	4.69E+06	5.90E+06	4.18E+06	5.43E+06	2.18E+06	2.66E+06	4.73E+06	8.12E+06	7.44E+06	0	0	0	0	0	0	0	0	1.79E-29	1.96E-44	0	0	0	10	10	11	ADA10_MOUSE, SP:O35598	SP|O35598|ADA10_MOUSE
Q61410	KGP2_MOUSE	cGMP-dependent protein kinase 2 OS=Mus musculus GN=Prkg2 PE=2 SV=1	1	2	0.56	6.31E+05	4.70E+05	1.66E+05	2.57E+05	2.88E+05	3.38E+05	1.99E+05	3.18E+05	3.05E+05	1.89E+05	2.20E+05	3.84E+05	1.55E+05	4.74E-07	6.92E-07	2.84E-05	1.36E-05	5.86E-05	1.02E-05	9.61E-05	3.61E-06	9.83E-06	6.05E-05	9.41E-07	6.12E-05	3.72E-04	3	2	2	KGP2_MOUSE, SP:Q61410	SP|Q61410|KGP2_MOUSE
Q99L02	PA1_MOUSE	PAXIP1-associated protein 1 OS=Mus musculus GN=Pa1 PE=1 SV=1	1	2	0.97	4.89E+05	4.29E+05	1.79E+05	1.73E+05	1.42E+05	2.23E+05	1.96E+05	2.30E+05	3.21E+05	2.48E+05	1.86E+05	2.81E+05	1.66E+05	6.99E-06	1.81E-06	1.35E-05	2.58E-04	0.003	2.53E-04	1.02E-04	6.13E-05	5.78E-06	5.21E-06	5.26E-06	4.81E-04	2.38E-04	3	2	2	PA1_MOUSE, SP:Q99L02	SP|Q99L02|PA1_MOUSE
Q99K90	TAB3_MOUSE	Mitogen-activated protein kinase kinase kinase 7-interacting protein 2 OS=Mus musculus GN=Map3k7ip2 PE=1 SV=1	1	9	0.93	1.67E+05	2.10E+05	3.64E+04	2.47E+05	1.23E+05	1.52E+05	1.06E+05	1.03E+05	2.33E+05	1.27E+05	5.41E+04	2.29E+05	1.34E+05	0.005	5.57E-04	0.036	1.81E-05	0.006	0.002	0.003	0.005	9.02E-05	7.70E-04	0.008	0.001	8.37E-04	4	9	9	TAB3_MOUSE, SP:Q571K4	SP|Q571K4|TAB3_MOUSE,SP|Q99K90|TAB2_MOUSE
P61148	FGF1_MOUSE	Heparin-binding growth factor 1 OS=Mus musculus GN=Fgf1 PE=2 SV=1	1	3	0.99	3.77E+04	3.14E+04	1.01E+04	2.22E+04	9.99E+03	3.62E+04	3.11E+04	2.11E+04	2.37E+04	3.25E+04	1.79E+04	1.76E+04	2.44E+04	0.112	0.103	0.158	0.106	0.235	0.079	0.069	0.121	0.115	0.054	0.084	0.203	0.089	4	3	3	FGF1_MOUSE, SP:P61148	SP|P61148|FGF1_MOUSE
Q91XQ0	DYH8_MOUSE	Dynein heavy chain 8, axonemal OS=Mus musculus GN=Dnahc8 PE=2 SV=1	1	5	0.89	2.56E+04	1.85E+04	3.63E+04	3.18E+04	6.43E+03	1.92E+04	2.81E+04	2.44E+04	3.01E+04	9.40E+04	1.45E+04	7.99E+03	3.01E+04	0.158	0.165	0.036	0.068	0.281	0.152	0.079	0.104	0.088	0.003	0.107	0.288	0.067	3	5	5	DYH8_MOUSE, SP:Q91XQ0	SP|Q91XQ0|DYH8_MOUSE
O88829	SIAT9_MOUSE	Lactosylceramide alpha-2,3-sialyltransferase OS=Mus musculus GN=St3gal5 PE=1 SV=2	1	4	0.68	1.27E+06	1.07E+06	1.22E+05	5.67E+05	6.91E+04	1.27E+05	9.20E+04	4.85E+05	7.73E+05	3.15E+04	5.36E+04	5.14E+04	1.94E+05	1.59E-12	1.48E-13	2.49E-04	2.02E-10	0.028	0.004	0.005	1.29E-08	0.015	0.056	0.008	0.078	7.38E-05	8	4	4	SIAT9_MOUSE, SP:O88829	SP|O88829|SIAT9_MOUSE
Q8C115	PKHH2_MOUSE	Pleckstrin homology domain-containing family H member 2 OS=Mus musculus GN=Plekhh2 PE=2 SV=2	1	13	0.97	2.09E+05	1.19E+05	8.16E+04	1.31E+05	8.37E+04	1.73E+05	1.72E+05	1.18E+05	1.58E+05	1.60E+05	1.23E+05	1.82E+05	1.24E+05	0.002	0.007	0.002	0.001	0.018	0.001	2.54E-04	0.003	0.001	1.87E-04	1.65E-04	0.004	0.001	4	13	13	PKHH2_MOUSE, SP:Q8C115	SP|Q8C115|PKHH2_MOUSE
Q91ZT5	FGD4_MOUSE	FYVE, RhoGEF and PH domain-containing protein 4 OS=Mus musculus GN=Fgd4 PE=1 SV=1	1	9	0.84	4.07E+05	3.24E+05	1.72E+05	2.30E+05	2.23E+05	2.90E+05	1.77E+05	2.22E+05	2.34E+05	1.90E+05	1.59E+05	3.57E+05	1.52E+05	3.56E-05	2.69E-05	1.88E-05	3.28E-05	3.31E-04	3.65E-05	2.10E-04	8.09E-05	8.68E-05	5.40E-05	2.21E-05	1.01E-04	4.07E-04	4	9	9	FGD4_MOUSE, SP:Q91ZT5	SP|Q91ZT5|FGD4_MOUSE
Q80XP9	WNK3_MOUSE	Serine/threonine-protein kinase WNK3 OS=Mus musculus GN=Wnk3 PE=2 SV=2	1	3	0.53	1.34E+03	1.73E+03	1.83E+05	1.25E+04	2.28E+04	2.22E+04	3.84E+04	1.58E+04	3.24E+04	3.11E+04	1.72E+04	2.04E+04	1.76E+04	0.409	0.383	1.13E-05	0.174	0.137	0.135	0.049	0.155	0.08	0.058	0.088	0.186	0.126	3	3	3	WNK3_MOUSE, SP:Q80XP9	SP|Q80XP9|WNK3_MOUSE
Q9Z0N2	IF2H_MOUSE	Eukaryotic translation initiation factor 2 subunit 3, Y-linked OS=Mus musculus GN=Eif2s3y PE=2 SV=2	1	1	0.99	4.26E+04	5.02E+04	3.77E+04	4.74E+04	3.91E+04	3.43E+04	3.85E+04	4.70E+04	4.78E+04	4.80E+04	5.45E+04	3.83E+04	4.12E+04	0.098	0.055	0.026	0.034	0.076	0.085	0.049	0.041	0.044	0.025	0.008	0.111	0.04	5	1	1	IF2H_MOUSE, SP:Q9Z0N2	SP|Q9Z0N2|IF2H_MOUSE,SP|Q9Z0N1|IF2G_MOUSE
Q4U4S6	XIRP2_MOUSE	Xin actin-binding repeat-containing protein 2 OS=Mus musculus GN=Xirp2 PE=1 SV=1	6	4	1	7.00E+04	5.60E+04	6.10E+04	5.65E+04	6.11E+04	7.03E+04	5.25E+04	1.51E+05	2.43E+05	1.64E+05	1.86E+05	2.09E+05	3.11E+05	0.049	0.046	0.007	0.024	0.037	0.025	0.027	8.87E-04	6.13E-05	1.52E-04	4.85E-06	0.002	7.88E-07	2, 3, 4, 5	1, 2, 4	1, 2, 4	XIRP2_MOUSE, SP:Q4U4S6	SP|Q4U4S6|XIRP2_MOUSE
P86049	RBM46_MOUSE	Probable RNA-binding protein 46 OS=Mus musculus GN=Rbm46 PE=4 SV=1	1	6	0.9	4.32E+04	2.61E+05	2.60E+04	3.53E+05	3.21E+04	2.68E+04	4.83E+04	3.45E+05	5.27E+05	7.43E+04	4.21E+04	2.95E+04	2.03E+05	0.097	1.43E-04	0.056	3.96E-07	0.099	0.116	0.033	1.35E-06	1.01E-08	0.008	0.019	0.143	5.58E-05	6	6	6	RBM46_MOUSE, SP:P86049	SP|P86049|RBM46_MOUSE
Q8BKA3	SRRM4_MOUSE	Serine/arginine repetitive matrix protein 4 OS=Mus musculus GN=Srrm4 PE=1 SV=1	1	10	0.83	2.81E+05	1.22E+05	8.65E+04	7.91E+04	7.60E+04	1.15E+05	1.10E+05	7.86E+04	1.29E+05	1.77E+05	7.90E+04	1.34E+05	9.19E+04	4.90E-04	0.006	0.002	0.009	0.023	0.006	0.003	0.012	0.003	9.62E-05	0.002	0.011	0.005	2	10	10	SRRM4_MOUSE, SP:Q8BKA3	SP|Q8BKA3|SRRM4_MOUSE
Q9BDB7	IF44L_MOUSE	Interferon-induced protein 44-like OS=Mus musculus GN=IFI44L PE=2 SV=2	1	8	0.59	2.98E+05	3.82E+05	3.54E+05	3.73E+05	2.93E+05	3.06E+05	3.64E+05	5.52E+05	3.64E+05	2.81E+05	3.24E+05	2.22E+05	3.08E+05	3.35E-04	5.98E-06	2.33E-09	2.07E-07	4.97E-05	2.32E-05	2.45E-07	1.81E-09	1.54E-06	1.33E-06	3.61E-09	0.002	1.05E-06	4	8	8	IF44L_MOUSE, SP:Q9BDB7	SP|Q9BDB7|IF44L_MOUSE
P09813	APOA2_MOUSE	Apolipoprotein A-II OS=Mus musculus GN=Apoa2 PE=1 SV=1	1	9	1	5.50E+04	3.80E+04	3.62E+04	4.65E+04	3.78E+04	4.81E+04	5.83E+04	3.68E+04	4.20E+04	3.98E+04	4.34E+04	5.33E+04	3.44E+04	0.084	0.105	0.036	0.046	0.097	0.064	0.026	0.079	0.072	0.07	0.027	0.083	0.068	2	9	9	APOA2_MOUSE, SP:P09813	SP|P09813|APOA2_MOUSE
Q4G5Y1	KLDC2_MOUSE	Kelch domain-containing protein 2 OS=Mus musculus GN=Klhdc2 PE=2 SV=2	1	1	0.95	3.75E+04	6.20E+04	5.45E+05	7.53E+04	4.90E+04	7.18E+04	1.01E+05	5.38E+04	5.85E+04	8.80E+04	6.35E+04	3.94E+04	8.39E+04	0.112	0.038	1.95E-13	0.011	0.054	0.024	0.004	0.031	0.029	0.004	0.005	0.107	0.006	6	1	1	KLDC2_MOUSE, SP:Q4G5Y1	SP|Q4G5Y1|KLDC2_MOUSE
Q99K41	EMIL1_MOUSE	EMILIN-1 OS=Mus musculus GN=Emilin1 PE=1 SV=1	1	3	1	1.96E+04	1.64E+04	7.03E+03	1.06E+04	1.36E+04	1.76E+04	1.21E+04	2.15E+04	4.36E+04	2.55E+04	1.72E+04	1.99E+04	3.33E+04	0.191	0.181	0.204	0.195	0.2	0.163	0.179	0.119	0.052	0.078	0.089	0.19	0.058	3	3	3	EMIL1_MOUSE, SP:Q99K41	SP|Q99K41|EMIL1_MOUSE
P82349	SGCB_MOUSE	Beta-sarcoglycan OS=Mus musculus GN=Sgcb PE=1 SV=1	2	10	1	5.71E+04	6.29E+04	4.96E+04	4.42E+04	4.96E+04	5.86E+04	5.69E+04	5.55E+04	5.15E+04	4.34E+04	4.96E+04	4.49E+04	4.81E+04	0.067	0.037	0.013	0.039	0.053	0.036	0.022	0.029	0.038	0.031	0.011	0.092	0.029	2, 4	10, 7	10, 7	SGCB_MOUSE, SP:P82349	SP|P82349|SGCB_MOUSE
Q9R0X4	ACOT9_MOUSE	Acyl-coenzyme A thioesterase 9, mitochondrial OS=Mus musculus GN=Acot9 PE=1 SV=1	1	7	0.93	3.07E+03	1.45E+04	1.61E+04	1.22E+04	1.07E+04	5.01E+03	1.40E+04	1.34E+04	1.19E+04	1.42E+04	1.57E+04	6.72E+03	2.98E+04	0.364	0.195	0.102	0.178	0.227	0.3	0.159	0.176	0.197	0.143	0.098	0.304	0.068	3	7	7	ACOT9_MOUSE, SP:Q9R0X4	SP|Q9R0X4|ACOT9_MOUSE,SP|Q32MW3|ACO10_MOUSE
O88833	CP4AA_MOUSE	Cytochrome P450 4A10 OS=Mus musculus GN=Cyp4a10 PE=2 SV=1	1	13	0.79	2.54E+05	2.55E+05	3.55E+05	4.55E+05	2.31E+05	4.08E+05	3.65E+05	4.34E+05	3.12E+05	2.99E+05	4.50E+05	1.99E+05	3.42E+05	8.15E-04	1.61E-04	1.92E-09	1.12E-08	2.64E-04	1.33E-06	2.21E-07	7.28E-08	7.29E-06	6.30E-07	4.88E-12	0.003	2.75E-07	5	13	13	CP4AA_MOUSE, SP:O88833	SP|O88833|CP4AA_MOUSE
O89103	C1QR1_MOUSE	Complement component C1q receptor OS=Mus musculus GN=Cd93 PE=1 SV=1	1	13	1	1.01E+06	2.01E+06	2.55E+06	1.81E+06	2.16E+06	1.72E+06	2.69E+06	1.52E+06	1.60E+06	2.50E+06	2.06E+06	1.46E+06	2.56E+06	2.24E-10	3.72E-23	0	6.28E-28	2.55E-25	1.38E-21	1.69E-39	2.17E-22	3.84E-22	3.40E-41	5.61E-45	4.20E-14	3.51E-39	5, 6	13	13	C1QR1_MOUSE, SP:O89103	SP|O89103|C1QR1_MOUSE
Q8BZ05	ARAP2_MOUSE	Arf-GAP with Rho-GAP domain, ANK repeat and PH domain-containing protein 2 OS=Mus musculus GN=Arap2 PE=1 SV=2	1	9	0.96	6.24E+05	8.14E+05	9.17E+05	9.42E+05	5.86E+05	7.16E+05	1.30E+06	1.25E+06	5.47E+05	1.26E+06	9.90E+05	6.85E+05	9.65E+05	5.21E-07	1.42E-10	6.91E-20	1.52E-15	2.56E-08	4.39E-10	4.32E-20	2.99E-18	6.74E-09	1.62E-21	1.42E-22	1.78E-07	2.72E-16	3	9	9	ARAP2_MOUSE, SP:Q8BZ05	SP|Q8BZ05|ARAP2_MOUSE
P02463	CO4A1_MOUSE	Collagen alpha-1(IV) chain OS=Mus musculus GN=Col4a1 PE=2 SV=4	2	5	1	5.08E+04	7.08E+04	1.07E+05	8.75E+04	1.09E+05	1.49E+05	1.23E+05	8.49E+04	1.90E+05	2.33E+05	1.18E+05	1.50E+05	1.47E+05	0.079	0.029	5.70E-04	0.007	0.009	0.002	0.002	0.01	3.39E-04	8.74E-06	1.97E-04	0.008	4.85E-04	2, 4, 3	2, 5, 4	2, 5, 4	CO4A1_MOUSE, SP:P02463	SP|P02463|CO4A1_MOUSE
Q76LL6	FHOD3_MOUSE	FH1/FH2 domain-containing protein 3 OS=Mus musculus GN=Fhod3 PE=2 SV=1	1	7	0.54	0.00E+00	6.76E+03	7.45E+04	2.92E+04	2.14E+04	3.91E+04	7.74E+04	2.10E+04	2.73E+04	2.01E+04	2.91E+04	8.96E+03	4.87E+04	0.5	0.278	0.003	0.076	0.145	0.072	0.01	0.122	0.099	0.102	0.039	0.277	0.029	3	7	7	FHOD3_MOUSE, SP:Q76LL6	SP|Q76LL6|FHOD3_MOUSE
Q3TQQ9	CA112_MOUSE	Uncharacterized protein C1orf112 homolog OS=Mus musculus PE=2 SV=2	1	8	0.65	3.11E+05	3.55E+05	1.20E+05	3.40E+05	2.85E+05	4.14E+05	3.12E+05	3.99E+05	1.75E+05	2.64E+05	5.35E+05	4.42E+05	7.70E+05	2.54E-04	1.22E-05	2.85E-04	6.68E-07	6.07E-05	1.17E-06	1.53E-06	2.38E-07	5.79E-04	2.63E-06	7.90E-14	1.79E-05	6.63E-14	4	8	8	CA112_MOUSE, SP:Q3TQQ9	SP|Q3TQQ9|CA112_MOUSE
P12979	MYOG_MOUSE	Myogenin OS=Mus musculus GN=Myog PE=2 SV=2	1	7	0.97	1.59E+06	1.74E+06	1.34E+06	1.32E+06	1.67E+06	1.09E+06	1.17E+06	1.14E+06	1.22E+06	8.16E+05	1.23E+06	1.20E+06	8.91E+05	8.73E-15	9.21E-20	1.11E-27	1.72E-20	1.78E-19	2.64E-14	1.32E-18	5.33E-17	4.32E-17	2.81E-15	4.01E-27	1.14E-11	1.77E-15	5	7	7	MYOG_MOUSE, SP:P12979	SP|P12979|MYOG_MOUSE
Q8BGC1	CE022_MOUSE	UPF0489 protein C5orf22 homolog OS=Mus musculus PE=2 SV=1	1	8	0.99	7.04E+04	3.63E+04	6.71E+04	8.77E+04	3.47E+04	3.76E+04	5.18E+04	4.83E+04	5.90E+04	6.88E+04	6.36E+04	3.23E+04	7.79E+04	0.049	0.088	0.005	0.007	0.09	0.077	0.028	0.039	0.03	0.01	0.005	0.131	0.008	4	8	8	CE022_MOUSE, SP:Q8BGC1	SP|Q8BGC1|CE022_MOUSE
Q9CR42	ANKR1_MOUSE	Ankyrin repeat domain-containing protein 1 OS=Mus musculus GN=Ankrd1 PE=2 SV=1	1	2	0.99	3.07E+03	8.66E+03	7.85E+03	5.50E+03	6.35E+03	4.40E+03	7.37E+03	9.20E+03	2.02E+04	1.70E+04	1.42E+04	2.82E+04	1.92E+04	0.364	0.253	0.188	0.267	0.282	0.311	0.234	0.22	0.134	0.122	0.11	0.147	0.116	2	2	2	ANKR1_MOUSE, SP:Q9CR42	SP|Q9CR42|ANKR1_MOUSE
Q9JIY0	PKHO1_MOUSE	Pleckstrin homology domain-containing family O member 1 OS=Mus musculus GN=Plekho1 PE=1 SV=1	1	4	0.76	2.73E+05	2.56E+05	1.95E+05	3.44E+05	2.33E+05	2.50E+05	2.11E+05	2.18E+05	2.31E+05	1.72E+05	1.52E+05	1.90E+05	1.85E+05	5.40E-04	1.58E-04	5.34E-06	5.14E-07	2.47E-04	1.11E-04	5.71E-05	8.73E-05	9.12E-05	1.11E-04	3.08E-05	0.003	1.08E-04	6	4	4	PKHO1_MOUSE, SP:Q9JIY0	SP|Q9JIY0|PKHO1_MOUSE
Q9DCC8	TOM20_MOUSE	Mitochondrial import receptor subunit TOM20 homolog OS=Mus musculus GN=Tomm20 PE=1 SV=1	1	11	1	7.64E+04	7.46E+04	8.74E+04	7.45E+04	8.29E+04	7.55E+04	5.66E+04	9.05E+04	6.13E+04	6.99E+04	5.25E+04	6.16E+04	4.96E+04	0.042	0.026	0.002	0.011	0.019	0.021	0.023	0.008	0.027	0.009	0.009	0.06	0.028	4	11	11	TOM20_MOUSE, SP:Q9DCC8	SP|Q9DCC8|TOM20_MOUSE
A2BE28	LAS1L_MOUSE	Protein LAS1 homolog OS=Mus musculus GN=Las1l PE=2 SV=1	1	1	0.99	1.01E+05	1.33E+05	1.18E+05	1.39E+05	1.38E+05	1.28E+05	1.22E+05	1.15E+05	1.24E+05	6.51E+04	1.28E+05	1.23E+05	1.06E+05	0.024	0.005	3.21E-04	9.38E-04	0.004	0.004	0.002	0.003	0.003	0.011	1.19E-04	0.014	0.003	4	1	1	LAS1L_MOUSE, SP:A2BE28	SP|A2BE28|LAS1L_MOUSE
Q8BR07	BICD1_MOUSE	Protein bicaudal D homolog 1 OS=Mus musculus GN=Bicd1 PE=1 SV=2	1	7	1	1.47E+04	2.43E+04	2.43E+04	2.34E+04	1.71E+04	2.85E+04	3.29E+04	3.35E+04	4.44E+04	1.29E+04	2.83E+04	3.18E+04	3.21E+04	0.224	0.133	0.06	0.1	0.173	0.106	0.064	0.071	0.05	0.156	0.042	0.133	0.062	3	7	7	BICD1_MOUSE, SP:Q8BR07	SP|Q8BR07|BICD1_MOUSE
Q80Z71	TENN_MOUSE	Tenascin-N OS=Mus musculus GN=Tnn PE=1 SV=1	1	11	0.98	5.83E+05	5.27E+05	6.56E+05	6.75E+05	2.13E+05	4.35E+05	1.09E+06	6.98E+05	4.79E+05	1.47E+06	5.56E+05	2.70E+05	6.40E+05	1.21E-06	1.74E-07	4.43E-15	1.29E-11	4.49E-04	7.56E-07	2.66E-17	3.29E-11	5.80E-08	3.82E-24	7.15E-14	6.18E-04	1.18E-11	4	11	11	TENN_MOUSE, SP:Q80Z71	SP|Q80Z71|TENN_MOUSE
Q99MS7	EH1L1_MOUSE	EH domain-binding protein 1-like protein 1 OS=Mus musculus GN=Ehbp1l1 PE=2 SV=1	1	7	0.63	1.01E+05	5.84E+05	3.18E+05	3.68E+05	2.60E+05	4.32E+05	2.75E+05	6.51E+05	6.57E+05	5.01E+05	5.55E+05	9.88E+04	2.03E+05	0.023	3.40E-08	1.32E-08	2.46E-07	1.18E-04	6.92E-07	6.04E-06	8.28E-11	2.38E-10	2.39E-10	2.96E-14	0.025	5.59E-05	5	7	7	EH1L1_MOUSE, SP:Q99MS7	SP|Q99MS7|EH1L1_MOUSE
Q8BIZ1	ANS1B_MOUSE	Ankyrin repeat and sterile alpha motif domain-containing protein 1B OS=Mus musculus GN=Anks1b PE=1 SV=2	1	13	1	5.11E+05	4.70E+05	5.13E+05	4.76E+05	3.95E+05	4.21E+05	4.86E+05	4.71E+05	2.64E+05	5.10E+05	3.07E+05	3.51E+05	2.89E+05	4.81E-06	7.00E-07	2.09E-12	7.68E-09	3.49E-06	1.07E-06	4.40E-09	2.93E-08	3.51E-05	2.69E-10	1.03E-08	1.19E-04	2.36E-06	4	13	13	ANS1B_MOUSE, SP:Q8BIZ1	SP|Q8BIZ1|ANS1B_MOUSE
Q8BTY1	KAT1_MOUSE	Kynurenine--oxoglutarate transaminase 1 OS=Mus musculus GN=Ccbl1 PE=2 SV=1	1	2	0.99	7.17E+04	6.80E+04	4.93E+04	7.41E+04	5.68E+04	4.91E+04	4.20E+04	5.36E+04	4.90E+04	3.60E+04	4.84E+04	4.87E+04	4.30E+04	0.047	0.032	0.013	0.011	0.042	0.051	0.042	0.031	0.042	0.046	0.012	0.084	0.037	5	2	2	KAT1_MOUSE, SP:Q8BTY1	SP|Q8BTY1|KAT1_MOUSE
Q9CXR1	DHRS7_MOUSE	Dehydrogenase/reductase SDR family member 7 OS=Mus musculus GN=Dhrs7 PE=2 SV=2	1	1	0.99	1.28E+04	1.19E+04	4.48E+03	8.64E+03	9.41E+03	9.07E+03	6.49E+03	7.97E+03	5.89E+03	5.67E+03	9.81E+03	2.96E+03	4.89E+03	0.239	0.218	0.254	0.219	0.243	0.241	0.25	0.237	0.276	0.254	0.156	0.368	0.274	2	1	1	DHRS7_MOUSE, SP:Q9CXR1	SP|Q9CXR1|DHRS7_MOUSE
Q91UZ5	IMPA2_MOUSE	Inositol monophosphatase 2 OS=Mus musculus GN=Impa2 PE=1 SV=1	1	5	0.58	1.83E+05	2.14E+05	1.71E+05	1.49E+05	2.17E+05	1.32E+05	1.03E+05	1.22E+05	1.20E+05	1.38E+05	1.47E+05	1.35E+05	1.18E+05	0.004	5.08E-04	2.08E-05	6.48E-04	4.01E-04	0.004	0.004	0.003	0.004	4.94E-04	4.73E-05	0.011	0.002	5	5	5	IMPA2_MOUSE, SP:Q91UZ5	SP|Q91UZ5|IMPA2_MOUSE
P20782	ACHE_MOUSE	Acetylcholine receptor subunit epsilon OS=Mus musculus GN=Chrne PE=2 SV=1	1	12	0.62	4.26E+04	5.09E+04	2.39E+05	1.57E+05	4.16E+04	5.21E+04	2.06E+05	1.10E+05	5.22E+04	3.15E+05	7.32E+04	2.08E+04	1.43E+05	0.098	0.054	7.16E-07	4.86E-04	0.07	0.045	7.44E-05	0.004	0.037	3.85E-07	0.003	0.184	5.90E-04	3	12	12	ACHE_MOUSE, SP:P20782	SP|P20782|ACHE_MOUSE
Q3UN02	LCLT1_MOUSE	Lysocardiolipin acyltransferase 1 OS=Mus musculus GN=Lclat1 PE=2 SV=2	1	2	0.98	4.43E+04	6.11E+04	4.67E+04	4.28E+04	4.55E+04	4.12E+04	7.89E+04	5.67E+04	6.79E+04	5.80E+04	7.12E+04	4.88E+04	6.49E+04	0.094	0.039	0.015	0.042	0.061	0.067	0.009	0.028	0.021	0.016	0.003	0.084	0.014	4	2	2	LCLT1_MOUSE, SP:Q3UN02	SP|Q3UN02|LCLT1_MOUSE
Q8BRH4	MLL3_MOUSE	Histone-lysine N-methyltransferase MLL3 OS=Mus musculus GN=Mll3 PE=2 SV=2	1	4	0.92	9.05E+05	7.87E+05	7.34E+05	1.04E+06	7.40E+05	9.86E+05	8.18E+05	7.74E+05	8.22E+05	8.49E+05	1.13E+06	9.23E+05	9.28E+05	1.77E-09	1.85E-10	2.55E-17	3.15E-17	3.68E-10	2.17E-13	3.18E-14	1.38E-12	1.44E-12	3.36E-16	1.73E-26	1.35E-09	1.86E-16	5	4	4	MLL3_MOUSE, SP:Q8BRH4	SP|Q8BRH4|MLL3_MOUSE
Q9D710	TMX2_MOUSE	Thioredoxin-related transmembrane protein 2 OS=Mus musculus GN=Tmx2 PE=2 SV=1	1	1	0.96	4.25E+04	4.11E+04	4.88E+04	4.46E+04	3.30E+04	2.36E+04	3.24E+04	4.11E+04	3.93E+04	4.00E+04	5.84E+04	3.31E+04	4.21E+04	0.1	0.076	0.014	0.041	0.097	0.133	0.067	0.054	0.063	0.04	0.008	0.13	0.04	3	1	1	TMX2_MOUSE, SP:Q9D710	SP|Q9D710|TMX2_MOUSE
P28661	SEPT4_MOUSE	Septin-4 OS=Mus musculus GN=Sept4 PE=1 SV=1	1	1	0.57	8.48E+03	7.50E+03	4.92E+03	1.15E+04	8.56E+03	7.99E+03	9.13E+03	6.10E+03	8.44E+03	5.97E+03	9.72E+03	1.26E+04	1.03E+04	0.282	0.269	0.242	0.185	0.252	0.254	0.211	0.265	0.237	0.246	0.156	0.241	0.191	2	1	1	SEPT4_MOUSE, SP:P28661	SP|P28661|SEPT4_MOUSE
Q9D7G0	PRPS1_MOUSE	Ribose-phosphate pyrophosphokinase 1 OS=Mus musculus GN=Prps1 PE=1 SV=3	1	2	0.95	9.17E+03	2.65E+04	2.59E+04	3.34E+04	1.67E+04	4.09E+04	2.27E+04	3.39E+04	2.37E+04	1.99E+04	3.24E+04	1.31E+04	4.28E+04	0.275	0.123	0.054	0.063	0.176	0.068	0.104	0.07	0.115	0.105	0.033	0.238	0.038	3	2	2	PRPS1_MOUSE, SP:Q9D7G0	SP|Q9D7G0|PRPS1_MOUSE,SP|Q9CS42|PRPS2_MOUSE
Q9R059	FHL3_MOUSE	Four and a half LIM domains protein 3 OS=Mus musculus GN=Fhl3 PE=2 SV=1	1	1	0.98	6.20E+03	7.49E+03	2.01E+03	7.09E+03	3.63E+03	6.81E+03	4.42E+03	9.25E+03	1.48E+04	1.14E+04	6.50E+03	9.27E+03	1.26E+04	0.311	0.268	0.327	0.24	0.331	0.27	0.288	0.219	0.171	0.17	0.203	0.273	0.166	2	1	1	FHL3_MOUSE, SP:Q9R059	SP|Q9R059|FHL3_MOUSE
P97303	BACH2_MOUSE	Transcription regulator protein BACH2 OS=Mus musculus GN=Bach2 PE=2 SV=1	1	3	0.54	2.96E+04	4.24E+04	2.15E+04	4.17E+04	2.69E+04	3.18E+04	4.54E+04	5.69E+04	1.87E+04	2.25E+04	3.48E+04	4.13E+04	4.72E+04	0.141	0.071	0.073	0.044	0.118	0.094	0.037	0.028	0.144	0.091	0.028	0.102	0.031	3	3	3	BACH2_MOUSE, SP:P97303	SP|P97303|BACH2_MOUSE
E9Q557	DESP_MOUSE	Desmoplakin OS=Mus musculus GN=Dsp PE=3 SV=1	33	13	1	2.99E+06	2.65E+06	2.30E+06	2.73E+06	2.51E+06	2.53E+06	2.62E+06	2.71E+06	2.73E+06	2.98E+06	3.01E+06	2.82E+06	3.42E+06	1.45E-27	2.18E-31	0	1.35E-43	1.27E-30	3.70E-32	2.15E-42	4.44E-40	4.49E-38	0	0	4.99E-26	0	2, 3, 4, 4, 5, 5, 6	1, 10, 12, 13, 2, 3, 4, 4, 13, 5, 7, 8, 9	1, 10, 12, 13, 13, 4, 2, 3, 4, 5, 7, 8, 9	DESP_MOUSE, SP:E9Q557	SP|E9Q557|DESP_MOUSE
Q9WVJ3	CBPQ_MOUSE	Carboxypeptidase Q OS=Mus musculus GN=Cpq PE=2 SV=1	1	1	0.99	3.88E+04	2.88E+04	2.65E+04	4.42E+04	3.51E+04	4.09E+04	2.27E+04	3.48E+04	3.44E+04	3.26E+04	4.92E+04	4.89E+04	3.79E+04	0.109	0.113	0.052	0.039	0.088	0.067	0.102	0.067	0.074	0.053	0.011	0.083	0.047	4	1	1	CBPQ_MOUSE, SP:Q9WVJ3	SP|Q9WVJ3|CBPQ_MOUSE
Q9ERH4	NUSAP_MOUSE	Nucleolar and spindle-associated protein 1 OS=Mus musculus GN=Nusap1 PE=1 SV=1	1	1	0.66	9.57E+03	0.00E+00	0.00E+00	2.81E+03	0.00E+00	0.00E+00	2.16E+04	0.00E+00	1.14E+04	1.43E+04	1.89E+03	0.00E+00	3.96E+03	0.27	0.5	0.5	0.328	0.5	0.5	0.108	0.5	0.202	0.143	0.327	0.5	0.293	2	1	1	NUSAP_MOUSE, SP:Q9ERH4	SP|Q9ERH4|NUSAP_MOUSE
Q9DC07	LNEBL_MOUSE	LIM zinc-binding domain-containing Nebulette OS=Mus musculus GN=Nebl PE=2 SV=1	6	13	1	1.48E+06	1.36E+06	9.85E+05	1.22E+06	1.10E+06	1.19E+06	1.15E+06	1.09E+06	1.08E+06	1.09E+06	1.18E+06	1.16E+06	1.14E+06	1.61E-14	6.11E-17	7.39E-23	2.17E-20	3.77E-14	6.46E-16	2.48E-19	3.01E-17	7.97E-16	8.13E-20	1.32E-27	1.34E-11	4.24E-20	2, 3, 4, 4, 5	1, 13, 2, 4, 9	1, 13, 2, 4, 9	LNEBL_MOUSE, SP:Q9DC07	SP|Q9DC07|LNEBL_MOUSE
Q9R1X4	TIM_MOUSE	Protein timeless homolog OS=Mus musculus GN=Timeless PE=1 SV=3	1	2	0.94	1.12E+05	1.24E+05	2.01E+04	9.55E+03	1.19E+05	2.08E+04	1.38E+04	2.42E+04	2.85E+04	4.69E+04	2.96E+04	5.58E+04	1.26E+04	0.018	0.006	0.079	0.209	0.006	0.143	0.163	0.106	0.095	0.027	0.039	0.07	0.168	4	2	2	TIM_MOUSE, SP:Q9R1X4	SP|Q9R1X4|TIM_MOUSE
Q61597	CRGC_MOUSE	Gamma-crystallin C OS=Mus musculus GN=Crygc PE=1 SV=3	1	1	0.63	7.25E+03	2.06E+04	6.68E+04	4.74E+04	2.17E+04	2.48E+04	2.00E+04	4.50E+04	5.29E+04	4.22E+04	3.46E+04	1.84E+04	2.50E+04	0.297	0.152	0.005	0.034	0.143	0.121	0.116	0.044	0.036	0.033	0.028	0.198	0.086	6	1	1	CRGC_MOUSE, SP:Q61597	SP|Q61597|CRGC_MOUSE
Q9QXS1	PLEC_MOUSE	Plectin OS=Mus musculus GN=Plec PE=1 SV=2	33	13	1	2.08E+06	1.90E+06	1.72E+06	1.95E+06	1.65E+06	1.86E+06	1.91E+06	2.08E+06	1.90E+06	1.89E+06	2.06E+06	1.86E+06	1.94E+06	1.05E-19	4.44E-23	2.87E-39	1.22E-31	7.81E-21	3.63E-24	1.95E-31	2.44E-31	3.63E-27	7.98E-35	0	8.07E-18	2.49E-33	2, 3, 3, 4, 4, 5, 6	1, 10, 11, 12, 13, 2, 2, 11, 3, 4, 5, 6, 7, 8, 9	1, 10, 11, 11, 2, 12, 13, 2, 3, 4, 5, 6, 7, 8, 9	PLEC_MOUSE, SP:Q9QXS1	SP|Q9QXS1|PLEC_MOUSE
Q8R104	SIR3_MOUSE	NAD-dependent protein deacetylase sirtuin-3 OS=Mus musculus GN=Sirt3 PE=2 SV=1	1	4	0.99	6.97E+03	3.80E+04	3.56E+04	1.89E+04	2.83E+04	5.46E+04	8.99E+03	7.53E+04	3.42E+04	8.05E+03	1.20E+04	6.37E+03	8.05E+03	0.3	0.082	0.03	0.124	0.111	0.042	0.212	0.014	0.074	0.211	0.129	0.309	0.219	4	4	4	SIR3_MOUSE, SP:Q8R104	SP|Q8R104|SIR3_MOUSE
Q02284	5HT1F_MOUSE	5-hydroxytryptamine receptor 1F OS=Mus musculus GN=Htr1f PE=2 SV=1	1	1	0.61	6.92E+03	1.41E+04	3.59E+04	9.69E+03	1.12E+04	9.94E+03	2.85E+03	9.78E+03	5.61E+03	1.13E+04	1.10E+04	8.03E+03	1.32E+04	0.301	0.198	0.029	0.205	0.222	0.23	0.077	0.213	0.279	0.171	0.14	0.287	0.161	3	1	1	5HT1F_MOUSE, SP:Q02284	SP|Q02284|5HT1F_MOUSE
E9Q401	RYR2_MOUSE	Ryanodine receptor 2 OS=Mus musculus GN=Ryr2 PE=1 SV=1	41	13	1	3.32E+06	2.99E+06	2.98E+06	3.08E+06	2.96E+06	2.91E+06	2.97E+06	2.90E+06	2.48E+06	2.45E+06	2.50E+06	2.45E+06	2.40E+06	2.79E-30	4.41E-35	0	0	1.45E-35	1.34E-36	0	1.15E-42	9.09E-35	2.24E-44	0	8.44E-23	1.09E-40	2, 3, 4, 5, 6	1, 10, 11, 12, 13, 2, 3, 4, 5, 6, 7, 8, 9	1, 10, 11, 12, 13, 2, 3, 4, 5, 6, 7, 8, 9	RYR2_MOUSE, SP:E9Q401	SP|E9Q401|RYR2_MOUSE
Q8VCT4	CES1D_MOUSE	Carboxylesterase 1D OS=Mus musculus GN=Ces1d PE=1 SV=1	6	10	1	3.19E+05	3.31E+05	3.55E+05	3.55E+05	2.84E+05	2.88E+05	3.19E+05	2.48E+05	1.27E+05	1.07E+05	1.56E+05	1.34E+05	9.68E+04	2.05E-04	2.09E-05	1.87E-09	3.73E-07	6.00E-05	3.79E-05	1.15E-06	3.41E-05	0.003	0.002	2.87E-05	0.011	0.004	2, 3, 4	1, 10, 4, 8	1, 10, 4, 8	CES1D_MOUSE, SP:Q8VCT4	SP|Q8VCT4|CES1D_MOUSE
Q8K2C6	SIR5_MOUSE	NAD-dependent protein deacylase sirtuin-5, mitochondrial OS=Mus musculus GN=Sirt5 PE=1 SV=1	2	12	1	1.27E+05	1.19E+05	1.02E+05	8.81E+04	1.20E+05	9.92E+04	1.16E+05	8.70E+04	1.19E+05	1.18E+05	9.56E+04	1.02E+05	9.62E+04	0.013	0.007	6.98E-04	0.007	0.006	0.01	0.002	0.009	0.004	0.001	7.33E-04	0.023	0.004	2, 4	12, 5	12, 5	SIR5_MOUSE, SP:Q8K2C6	SP|Q8K2C6|SIR5_MOUSE
P23953	EST1C_MOUSE	Carboxylesterase 1C OS=Mus musculus GN=Ces1c PE=1 SV=4	2	11	1	7.21E+04	1.05E+05	3.40E+04	5.52E+04	3.70E+04	6.69E+04	5.78E+04	6.65E+04	5.25E+04	4.77E+04	4.70E+04	6.14E+04	4.73E+04	0.047	0.01	0.033	0.025	0.083	0.028	0.022	0.019	0.037	0.026	0.013	0.061	0.031	2, 3	11, 4	11, 4	EST1C_MOUSE, SP:P23953	SP|P23953|EST1C_MOUSE
P11983	TCPA_MOUSE	T-complex protein 1 subunit alpha OS=Mus musculus GN=Tcp1 PE=1 SV=3	3	11	1	1.70E+05	1.47E+05	1.33E+05	1.59E+05	1.37E+05	1.65E+05	1.28E+05	2.15E+05	1.51E+05	1.59E+05	1.80E+05	1.40E+05	1.64E+05	0.005	0.003	1.41E-04	4.20E-04	0.004	0.001	0.001	9.69E-05	0.001	1.91E-04	6.60E-06	0.01	2.44E-04	2, 3, 4, 5	1, 11, 2, 6	1, 11, 2, 6	TCPA_MOUSE, SP:P11983	SP|P11983|TCPA_MOUSE
Q9ESB3	HRG_MOUSE	Histidine-rich glycoprotein OS=Mus musculus GN=Hrg PE=1 SV=2	2	3	1	4.35E+04	4.18E+04	2.82E+04	3.17E+04	1.88E+04	2.70E+04	3.01E+04	3.73E+04	1.73E+04	2.83E+04	2.56E+04	2.63E+04	3.91E+04	0.096	0.072	0.047	0.068	0.16	0.112	0.072	0.06	0.153	0.067	0.05	0.155	0.044	2	2, 3	2, 3	HRG_MOUSE, SP:Q9ESB3	SP|Q9ESB3|HRG_MOUSE
Q9DB60	PGFS_MOUSE	Prostamide/prostaglandin F synthase OS=Mus musculus GN=Fam213b PE=1 SV=1	2	9	1	1.43E+04	2.72E+04	6.53E+04	4.55E+04	3.24E+04	5.62E+04	7.15E+04	5.67E+04	3.85E+04	1.15E+05	4.51E+04	1.73E+04	6.79E+04	0.227	0.12	0.005	0.037	0.096	0.04	0.012	0.028	0.063	0.001	0.014	0.206	0.013	2, 4	4, 9	4, 9	PGFS_MOUSE, SP:Q9DB60	SP|Q9DB60|PGFS_MOUSE
Q14B70	HDX_MOUSE	Highly divergent homeobox OS=Mus musculus GN=Hdx PE=2 SV=1	1	1	0.87	8.27E+03	1.39E+04	4.45E+03	4.94E+03	9.34E+03	7.20E+03	1.86E+03	5.54E+03	5.75E+03	6.06E+03	4.08E+03	9.44E+03	5.06E+03	0.284	0.2	0.252	0.278	0.242	0.265	0.358	0.274	0.277	0.243	0.255	0.271	0.269	2	1	1	HDX_MOUSE, SP:Q14B70	SP|Q14B70|HDX_MOUSE
D3Z7P3	GLSK_MOUSE	Glutaminase kidney isoform, mitochondrial OS=Mus musculus GN=Gls PE=1 SV=1	1	1	0.98	1.27E+04	1.85E+04	1.44E+04	1.77E+04	1.46E+04	1.22E+04	1.19E+04	3.08E+04	1.87E+04	1.23E+04	5.89E+03	1.08E+04	1.12E+04	0.242	0.168	0.119	0.134	0.193	0.208	0.18	0.08	0.144	0.162	0.219	0.259	0.182	2	1	1	GLSK_MOUSE, SP:D3Z7P3	SP|D3Z7P3|GLSK_MOUSE
Q3UN90	CQ108_MOUSE	UPF0631 protein C17orf108 homolog OS=Mus musculus PE=2 SV=1	1	3	0.94	4.51E+04	3.43E+04	7.67E+03	2.03E+04	1.26E+04	2.54E+04	2.01E+04	1.77E+04	2.09E+04	1.97E+04	1.47E+04	1.90E+04	1.92E+04	0.092	0.093	0.191	0.116	0.208	0.119	0.116	0.142	0.13	0.105	0.106	0.194	0.116	3	3	3	CQ108_MOUSE, SP:Q3UN90	SP|Q3UN90|CQ108_MOUSE
Q8VHI4	AL2S8_MOUSE	Amyotrophic lateral sclerosis 2 chromosomal region candidate gene 8 protein homolog OS=Mus musculus GN=Als2cr8 PE=1 SV=1	1	6	0.53	1.86E+04	1.76E+04	7.45E+03	7.61E+03	1.40E+04	1.52E+04	8.02E+03	1.29E+04	1.78E+04	1.43E+04	1.26E+04	1.88E+04	1.12E+04	0.196	0.172	0.194	0.232	0.196	0.181	0.225	0.18	0.148	0.142	0.123	0.195	0.181	2	6	6	AL2S8_MOUSE, SP:Q8VHI4	SP|Q8VHI4|AL2S8_MOUSE
Q0II04	NEBL_MOUSE	Nebulette OS=Mus musculus GN=Nebl PE=2 SV=1	9	13	1	1.90E+06	1.98E+06	1.73E+06	1.73E+06	1.80E+06	1.70E+06	1.76E+06	1.68E+06	1.57E+06	1.71E+06	1.78E+06	1.73E+06	1.78E+06	5.17E-18	1.07E-23	5.40E-39	4.49E-28	2.62E-22	4.16E-22	8.20E-29	2.43E-25	1.26E-22	3.12E-31	3.69E-42	1.42E-16	1.82E-30	2, 3, 5, 4, 5	1, 10, 11, 13, 7, 13, 8	1, 10, 11, 13, 13, 7, 8	NEBL_MOUSE, SP:Q0II04	SP|Q0II04|NEBL_MOUSE
P70404	IDHG1_MOUSE	Isocitrate dehydrogenase [NAD] subunit gamma 1, mitochondrial OS=Mus musculus GN=Idh3g PE=1 SV=1	9	13	1	4.93E+06	5.54E+06	5.27E+06	5.66E+06	6.09E+06	5.91E+06	5.68E+06	5.00E+06	4.79E+06	5.50E+06	5.49E+06	5.03E+06	5.28E+06	3.13E-42	0	0	0	0	0	0	0	0	0	0	1.74E-43	0	2, 3, 3, 5, 4, 5, 6, 9	11, 12, 13, 2, 3, 8, 4, 7	11, 12, 13, 2, 3, 8, 4, 7	IDHG1_MOUSE, SP:P70404	SP|P70404|IDHG1_MOUSE
P0CG49	UBC_MOUSE	Polyubiquitin-B OS=Mus musculus GN=Ubb PE=1 SV=1	3	13	1	1.90E+06	1.91E+06	1.65E+06	1.71E+06	1.94E+06	1.58E+06	2.02E+06	1.99E+06	1.67E+06	2.32E+06	1.89E+06	1.85E+06	2.05E+06	1.10E-17	2.23E-22	1.76E-36	4.18E-27	3.09E-23	2.78E-20	7.27E-32	8.18E-29	2.58E-23	1.49E-40	6.05E-43	2.84E-17	1.12E-33	2, 4, 4, 5, 6	1, 1, 13, 13	1, 1, 13, 13	UBC_MOUSE, SP:P0CG50	SP|P0CG50|UBC_MOUSE,SP|P0CG49|UBB_MOUSE,SP|P62983|RS27A_MOUSE,SP|P62984|RL40_MOUSE
Q9CRB8	MTFP1_MOUSE	Mitochondrial fission process protein 1 OS=Mus musculus GN=Mtfp1 PE=1 SV=1	3	13	1	8.38E+05	7.09E+05	6.78E+05	7.40E+05	6.85E+05	7.38E+05	6.19E+05	7.01E+05	4.68E+05	6.12E+05	6.03E+05	5.97E+05	5.02E+05	6.58E-09	1.32E-09	4.99E-14	6.45E-13	1.51E-09	1.42E-10	5.46E-11	1.50E-11	5.86E-08	4.84E-12	1.77E-15	8.10E-07	1.07E-09	4, 5	13, 4, 6	13, 4, 6	MTFP1_MOUSE, SP:Q9CRB8	SP|Q9CRB8|MTFP1_MOUSE
Q6DIC0	SMCA2_MOUSE	Probable global transcription activator SNF2L2 OS=Mus musculus GN=Smarca2 PE=1 SV=1	1	1	0.52	4.71E+05	6.04E+05	3.89E+05	5.60E+05	5.48E+05	5.56E+05	5.55E+05	5.86E+05	4.50E+05	5.36E+05	5.28E+05	4.26E+05	5.13E+05	2.16E-05	4.88E-08	1.82E-06	9.19E-10	1.77E-07	1.86E-07	3.36E-09	1.79E-09	1.58E-06	1.92E-10	7.46E-11	0.002	2.61E-08	6	1	1	SMCA2_MOUSE, SP:Q6DIC0	SP|Q6DIC0|SMCA2_MOUSE
Q8K031	STAR8_MOUSE	StAR-related lipid transfer protein 8 OS=Mus musculus GN=Stard8 PE=1 SV=2	1	13	0.78	1.48E+05	1.71E+05	2.64E+05	1.82E+05	1.27E+05	1.30E+05	2.79E+05	2.10E+05	1.29E+05	3.47E+05	1.85E+05	2.67E+05	2.49E+05	0.008	0.002	2.17E-07	1.91E-04	0.005	0.004	5.48E-06	1.29E-04	0.003	1.11E-07	5.65E-06	6.57E-04	1.04E-05	4	13	13	STAR8_MOUSE, SP:Q8K031	SP|Q8K031|STAR8_MOUSE
Q9Z0H7	BCL10_MOUSE	B-cell lymphoma/leukemia 10 OS=Mus musculus GN=Bcl10 PE=2 SV=1	1	11	0.59	2.53E+05	1.81E+05	2.90E+05	2.09E+05	3.94E+04	1.74E+05	4.68E+05	2.02E+05	1.72E+05	5.34E+05	1.25E+05	6.41E+04	2.40E+05	8.60E-04	0.001	5.62E-08	7.25E-05	0.075	0.001	7.28E-09	1.63E-04	6.36E-04	8.73E-11	1.48E-04	0.057	1.40E-05	4	11	11	BCL10_MOUSE, SP:Q9Z0H7	SP|Q9Z0H7|BCL10_MOUSE
P42125	ECI1_MOUSE	Enoyl-CoA delta isomerase 1, mitochondrial OS=Mus musculus GN=Eci1 PE=2 SV=1	10	13	1	7.34E+06	9.00E+06	1.17E+07	9.18E+06	1.09E+07	1.05E+07	1.03E+07	8.86E+06	7.00E+06	9.04E+06	7.01E+06	6.86E+06	6.07E+06	0	0	0	0	0	0	0	0	0	0	0	0	0	3, 3, 5, 4, 4, 5, 4, 6, 5, 6, 6, 7	1, 1, 10, 12, 13, 9, 9, 11	1, 1, 10, 11, 9, 12, 13, 9	ECI1_MOUSE, SP:P42125	SP|P42125|ECI1_MOUSE
Q8BLF1	NCEH1_MOUSE	Neutral cholesterol ester hydrolase 1 OS=Mus musculus GN=Nceh1 PE=1 SV=1	2	12	1	6.15E+05	4.40E+05	3.99E+05	4.49E+05	5.12E+05	5.01E+05	4.07E+05	4.01E+05	3.87E+05	3.45E+05	3.46E+05	4.80E+05	3.82E+05	5.04E-07	1.23E-06	2.19E-10	1.24E-08	1.31E-07	9.15E-08	4.35E-08	2.10E-07	6.96E-07	9.20E-08	8.90E-10	7.82E-06	5.80E-08	5, 6	12, 5	12, 5	NCEH1_MOUSE, SP:Q8BLF1	SP|Q8BLF1|NCEH1_MOUSE
P13864	DNMT1_MOUSE	DNA (cytosine-5)-methyltransferase 1 OS=Mus musculus GN=Dnmt1 PE=1 SV=5	1	10	0.86	3.69E+05	3.36E+05	3.96E+05	3.71E+05	3.35E+05	3.82E+05	4.06E+05	4.08E+05	3.81E+05	2.58E+05	2.40E+05	2.00E+05	2.74E+05	7.79E-05	2.01E-05	4.53E-10	2.42E-07	1.67E-05	3.09E-06	8.41E-08	2.05E-07	9.21E-07	4.01E-06	3.18E-07	0.003	5.15E-06	5	10	10	DNMT1_MOUSE, SP:P13864	SP|P13864|DNMT1_MOUSE
Q6QR59	F115E_MOUSE	Protein FAM115E OS=Mus musculus GN=Fam115e PE=2 SV=1	1	9	0.94	5.12E+05	4.81E+05	3.97E+05	4.05E+05	5.00E+05	4.45E+05	3.26E+05	4.26E+05	3.46E+05	2.76E+05	3.97E+05	4.32E+05	3.30E+05	4.08E-06	4.24E-07	2.24E-10	5.89E-08	1.85E-07	4.59E-07	8.55E-07	8.96E-08	2.47E-06	1.47E-06	6.50E-11	2.12E-05	4.21E-07	6	9	9	F115E_MOUSE, SP:Q6QR59	SP|Q6QR59|F115E_MOUSE
A2AFS3	K1324_MOUSE	UPF0577 protein KIAA1324 OS=Mus musculus GN=Kiaa1324 PE=2 SV=1	1	2	0.63	1.00E+05	1.54E+05	8.49E+05	1.73E+05	1.07E+05	1.46E+05	2.96E+05	1.92E+05	9.50E+04	1.34E+05	1.62E+05	1.24E+05	2.24E+05	0.024	0.003	4.43E-19	2.63E-04	0.009	0.002	2.74E-06	2.23E-04	0.008	5.52E-04	1.82E-05	0.014	2.49E-05	3	2	2	K1324_MOUSE, SP:A2AFS3	SP|A2AFS3|K1324_MOUSE
O88398	AVIL_MOUSE	Advillin OS=Mus musculus GN=Avil PE=1 SV=2	1	1	0.9	6.52E+05	1.05E+06	5.68E+05	8.63E+05	3.50E+05	5.53E+05	4.66E+05	8.78E+05	2.77E+05	1.37E+05	1.90E+05	1.42E+05	5.14E+05	2.49E-07	2.48E-13	5.45E-14	8.17E-15	1.01E-05	2.23E-08	5.69E-09	4.88E-14	2.19E-05	5.06E-04	4.26E-06	0.009	4.46E-10	7	1	1	AVIL_MOUSE, SP:O88398	SP|O88398|AVIL_MOUSE
Q9D3H0	TMM80_MOUSE	Transmembrane protein 80 OS=Mus musculus GN=Tmem80 PE=2 SV=1	1	4	0.71	7.21E+04	9.19E+04	3.90E+05	1.62E+05	5.67E+05	5.58E+05	3.85E+05	1.36E+05	9.27E+05	6.62E+05	3.84E+05	4.81E+05	3.26E+05	0.047	0.016	3.05E-10	3.85E-04	3.16E-08	2.04E-08	1.02E-07	0.002	6.37E-14	3.55E-13	1.27E-10	7.74E-06	4.84E-07	7	4	4	TMM80_MOUSE, SP:Q9D3H0	SP|Q9D3H0|TMM80_MOUSE
Q3ULF4	SPG7_MOUSE	Paraplegin OS=Mus musculus GN=Spg7 PE=1 SV=1	1	1	0.94	5.10E+04	2.55E+04	1.60E+04	3.99E+04	3.08E+04	2.40E+04	2.90E+04	2.51E+04	2.10E+04	2.21E+04	4.43E+04	3.79E+04	2.90E+04	0.079	0.128	0.106	0.048	0.102	0.126	0.076	0.102	0.13	0.092	0.015	0.112	0.071	4	1	1	SPG7_MOUSE, SP:Q3ULF4	SP|Q3ULF4|SPG7_MOUSE
Q8C0D0	TRUB1_MOUSE	Probable tRNA pseudouridine synthase 1 OS=Mus musculus GN=Trub1 PE=2 SV=1	1	13	0.99	4.92E+05	3.90E+05	1.77E+05	2.66E+05	2.05E+05	3.26E+05	2.68E+05	2.75E+05	3.77E+05	3.60E+05	2.45E+05	3.27E+05	3.05E+05	7.32E-06	5.29E-06	1.65E-05	9.79E-06	6.06E-04	1.54E-05	9.49E-06	1.55E-05	1.12E-06	7.68E-08	2.25E-07	2.03E-04	1.35E-06	4	10, 13	10, 13	TRUB1_MOUSE, SP:Q8C0D0	SP|Q8C0D0|TRUB1_MOUSE
Q3LAC4	PREX2_MOUSE	Phosphatidylinositol 3,4,5-trisphosphate-dependent Rac exchanger 2 protein OS=Mus musculus GN=Prex2 PE=2 SV=2	1	5	0.57	1.47E+05	2.38E+05	2.06E+05	1.65E+05	2.50E+05	2.53E+05	2.46E+05	2.06E+05	2.26E+05	2.27E+05	2.38E+05	3.13E+05	2.93E+05	0.008	2.62E-04	3.46E-06	3.45E-04	1.58E-04	1.06E-04	1.68E-05	1.37E-04	1.12E-04	1.17E-05	3.26E-07	2.48E-04	1.89E-06	5	5	5	PREX2_MOUSE, SP:Q3LAC4	SP|Q3LAC4|PREX2_MOUSE
A6H6E2	MMRN2_MOUSE	Multimerin-2 OS=Mus musculus GN=Mmrn2 PE=2 SV=1	1	1	0.99	4.25E+05	4.68E+05	2.62E+05	3.42E+05	3.80E+05	4.20E+05	2.84E+05	4.03E+05	4.71E+05	4.45E+05	4.37E+05	4.32E+05	4.26E+05	2.32E-05	5.85E-07	1.78E-07	5.33E-07	4.38E-06	8.74E-07	3.80E-06	1.80E-07	4.91E-08	1.57E-09	7.37E-12	2.07E-05	1.09E-08	7	1	1	MMRN2_MOUSE, SP:A6H6E2	SP|A6H6E2|MMRN2_MOUSE
P02301	H31_MOUSE	Histone H3.3C OS=Mus musculus GN=H3f3c PE=1 SV=3	3	13	1	1.73E+06	1.59E+06	1.87E+06	1.79E+06	1.22E+06	1.36E+06	1.88E+06	1.62E+06	1.62E+06	2.94E+06	1.40E+06	9.46E+05	1.42E+06	1.71E-16	3.21E-19	1.26E-39	2.74E-28	1.61E-15	8.23E-18	9.63E-30	4.53E-24	7.12E-23	0	8.83E-33	7.90E-10	3.59E-24	3, 4, 4, 5	10, 12, 12, 13	10, 12, 12, 13	H31_MOUSE, SP:P68433	SP|P68433|H31_MOUSE,SP|P84228|H32_MOUSE,SP|P84244|H33_MOUSE,SP|P02301|H3C_MOUSE
Q9WUR2	ECI2_MOUSE	Enoyl-CoA delta isomerase 2, mitochondrial OS=Mus musculus GN=Eci2 PE=1 SV=2	6	13	1	1.02E+06	1.19E+06	9.72E+05	1.11E+06	9.94E+05	1.00E+06	1.01E+06	9.88E+05	8.67E+05	9.10E+05	9.44E+05	8.02E+05	7.81E+05	1.38E-10	4.36E-15	6.73E-23	9.21E-19	3.24E-13	8.08E-14	1.81E-17	8.99E-16	2.27E-13	1.18E-17	1.48E-23	1.14E-08	1.85E-14	2, 4, 4, 5, 6	12, 13, 2, 6, 9	12, 13, 2, 6, 9	ECI2_MOUSE, SP:Q9WUR2	SP|Q9WUR2|ECI2_MOUSE
P97793	ALK_MOUSE	ALK tyrosine kinase receptor OS=Mus musculus GN=Alk PE=1 SV=1	1	2	0.95	2.33E+05	3.69E+05	1.45E+05	3.45E+05	3.05E+05	3.47E+05	2.29E+05	3.56E+05	3.24E+05	2.62E+05	3.61E+05	2.39E+05	3.07E+05	0.001	7.96E-06	7.45E-05	5.18E-07	3.39E-05	7.06E-06	2.94E-05	8.93E-07	4.89E-06	2.69E-06	4.34E-10	0.001	1.03E-06	4	2	2	ALK_MOUSE, SP:P97793	SP|P97793|ALK_MOUSE
Q3TLH4	PRC2C_MOUSE	Protein PRRC2C OS=Mus musculus GN=Prrc2c PE=1 SV=2	1	1	0.98	3.42E+05	2.74E+05	2.75E+05	2.23E+05	3.44E+05	2.30E+05	2.53E+05	2.02E+05	1.66E+05	3.04E+05	2.36E+05	3.49E+05	2.38E+05	1.33E-04	9.98E-05	1.06E-07	4.25E-05	1.24E-05	2.06E-04	1.28E-05	1.61E-04	7.80E-04	5.24E-07	3.45E-07	1.18E-04	1.49E-05	5	1	1	PRC2C_MOUSE, SP:Q3TLH4	SP|Q3TLH4|PRC2C_MOUSE
Q5S006	LRRK2_MOUSE	Leucine-rich repeat serine/threonine-protein kinase 2 OS=Mus musculus GN=Lrrk2 PE=1 SV=2	1	8	0.69	1.52E+05	1.16E+05	1.05E+05	1.41E+05	1.05E+05	1.02E+05	1.24E+05	1.08E+05	1.06E+05	9.92E+04	1.26E+05	1.09E+05	1.77E+05	0.007	0.008	6.38E-04	9.01E-04	0.01	0.009	0.002	0.004	0.006	0.003	1.40E-04	0.02	1.60E-04	2	8	8	LRRK2_MOUSE, SP:Q5S006	SP|Q5S006|LRRK2_MOUSE
Q7TN08	DACT2_MOUSE	Dapper homolog 2 OS=Mus musculus GN=Dact2 PE=2 SV=2	1	4	0.8	2.67E+05	3.29E+05	1.90E+05	1.78E+05	2.48E+05	3.41E+05	1.79E+05	2.21E+05	2.62E+05	3.18E+05	2.63E+05	2.57E+05	3.01E+05	6.17E-04	2.29E-05	7.10E-06	2.10E-04	1.63E-04	8.24E-06	1.89E-04	8.16E-05	3.42E-05	2.79E-07	7.43E-08	7.75E-04	1.25E-06	6	4	4	DACT2_MOUSE, SP:Q7TN08	SP|Q7TN08|DACT2_MOUSE
A2A5N8	LMBL1_MOUSE	Lethal(3)malignant brain tumor-like protein 1 OS=Mus musculus GN=L3mbtl1 PE=2 SV=1	1	5	0.83	1.39E+05	1.56E+05	1.62E+05	1.39E+05	1.55E+05	1.92E+05	1.67E+05	2.44E+05	1.86E+05	1.11E+05	1.46E+05	1.60E+05	1.68E+05	0.01	0.003	3.54E-05	9.50E-04	0.002	6.23E-04	3.15E-04	4.11E-05	4.07E-04	0.002	4.89E-05	0.006	2.22E-04	5	5	5	LMBL1_MOUSE, SP:A2A5N8	SP|A2A5N8|LMBL1_MOUSE
Q61315	APC_MOUSE	Adenomatous polyposis coli protein OS=Mus musculus GN=Apc PE=1 SV=1	1	4	0.94	3.12E+06	4.82E+06	3.68E+06	4.53E+06	4.92E+06	4.93E+06	5.44E+06	5.52E+06	4.42E+06	4.50E+06	3.93E+06	5.88E+06	5.49E+06	3.35E-26	0	0	0	0	0	0	0	0	0	0	8.55E-44	0	7	4	4	APC_MOUSE, SP:Q61315	SP|Q61315|APC_MOUSE
P00761	TRYP_PIG	Trypsin OS=Sus scrofa PE=1 SV=1	5	13	1	1.13E+08	1.04E+08	1.59E+08	8.92E+07	1.09E+08	1.22E+08	1.30E+08	9.32E+07	1.21E+08	9.54E+07	1.08E+08	1.82E+08	1.19E+08	0	0	0	0	0	0	0	0	0	0	0	0	0	5, 7, 6, 6, 8, 7, 8	11, 12, 13, 2, 12	11, 13, 14, 25, 16	TRYP_PIG, SP:P00761	SP|P00761|TRYP_PIG
Q6RUT8	CC154_MOUSE	Coiled-coil domain-containing protein 154 OS=Mus musculus GN=Ccdc154 PE=2 SV=2	1	11	0.81	1.08E+06	2.35E+06	1.67E+06	2.61E+06	1.68E+06	1.80E+06	2.54E+06	2.68E+06	2.14E+06	1.47E+06	1.92E+06	4.99E+05	1.64E+06	7.77E-11	6.42E-26	3.58E-34	6.45E-37	5.76E-20	5.02E-22	2.08E-36	2.69E-35	7.65E-28	1.84E-25	2.48E-40	5.74E-06	2.33E-26	6	11	11	CC154_MOUSE, SP:Q6RUT8	SP|Q6RUT8|CC154_MOUSE
Q99KU6	CF089_MOUSE	Bombesin receptor-activated protein C6orf89 homolog OS=Mus musculus PE=2 SV=2	1	8	0.77	9.67E+05	7.59E+05	5.44E+05	6.43E+05	4.53E+05	7.00E+05	8.02E+05	7.75E+05	5.32E+05	8.15E+05	5.67E+05	6.36E+05	6.07E+05	8.06E-10	5.62E-10	3.92E-13	2.77E-11	7.70E-07	6.42E-10	1.23E-13	2.51E-12	1.11E-08	3.36E-15	2.79E-14	4.29E-07	2.62E-11	5	8	8	CF089_MOUSE, SP:Q99KU6	SP|Q99KU6|CF089_MOUSE
A2A8L5	PTPRF_MOUSE	Receptor-type tyrosine-protein phosphatase F OS=Mus musculus GN=Ptprf PE=1 SV=1	1	9	0.86	9.66E+05	1.12E+06	8.64E+05	1.12E+06	8.91E+05	1.04E+06	1.34E+06	1.47E+06	7.84E+05	8.32E+05	1.22E+06	1.19E+06	1.04E+06	6.85E-10	7.83E-14	1.74E-19	5.00E-18	1.07E-11	9.38E-14	4.14E-21	3.27E-21	6.35E-12	1.17E-15	1.82E-27	1.12E-11	9.92E-18	4	9	9	PTPRF_MOUSE, SP:A2A8L5	SP|A2A8L5|PTPRF_MOUSE
Q8K557	ONEC3_MOUSE	One cut domain family member 3 OS=Mus musculus GN=Onecut3 PE=1 SV=2	1	8	0.88	1.86E+06	2.01E+06	1.30E+06	2.56E+06	9.05E+05	1.44E+06	1.04E+06	2.17E+06	1.50E+06	1.60E+06	1.55E+06	4.40E+05	1.33E+06	2.43E-17	3.30E-23	1.43E-28	9.89E-38	5.07E-12	1.60E-18	1.89E-17	1.55E-30	4.81E-21	3.54E-28	5.13E-35	1.79E-05	1.84E-22	8	8	8	ONEC3_MOUSE, SP:Q8K557	SP|Q8K557|ONEC3_MOUSE
Q9QUK3	CLN8_MOUSE	Protein CLN8 OS=Mus musculus GN=Cln8 PE=2 SV=1	1	9	0.9	1.87E+06	2.33E+06	6.40E+05	1.37E+06	1.06E+06	1.35E+06	7.90E+05	1.29E+06	1.05E+06	6.96E+05	4.76E+05	8.81E+05	1.18E+06	2.08E-17	2.46E-26	1.80E-15	5.92E-22	1.15E-13	1.64E-17	7.90E-14	2.02E-19	1.89E-15	1.01E-13	1.04E-12	2.98E-09	3.28E-20	6	9	9	CLN8_MOUSE, SP:Q9QUK3	SP|Q9QUK3|CLN8_MOUSE
Q3V2J0	FA92B_MOUSE	Protein FAM92B OS=Mus musculus GN=Fam92b PE=2 SV=1	1	9	0.59	1.36E+06	1.29E+06	2.88E+05	7.11E+05	5.98E+05	1.14E+06	4.40E+05	9.67E+05	9.20E+05	3.60E+05	4.54E+05	1.37E+06	5.90E+05	4.62E-13	1.59E-15	5.71E-08	2.44E-12	1.66E-08	6.49E-15	1.78E-08	6.54E-15	1.39E-13	5.84E-08	4.83E-12	4.21E-13	3.95E-11	5	9	9	FA92B_MOUSE, SP:Q3V2J0	SP|Q3V2J0|FA92B_MOUSE
Q8BGY7	F210A_MOUSE	Protein FAM210A OS=Mus musculus GN=Fam210a PE=2 SV=1	1	2	1	1.27E+05	9.92E+04	5.62E+04	7.53E+04	8.73E+04	7.07E+04	5.13E+04	8.29E+04	5.86E+04	3.41E+04	2.13E+04	3.62E+04	2.46E+04	0.013	0.012	0.009	0.011	0.017	0.025	0.028	0.01	0.03	0.05	0.068	0.118	0.089	4	2	2	F210A_MOUSE, SP:Q8BGY7	SP|Q8BGY7|F210A_MOUSE
Q9JI33	NET4_MOUSE	Netrin-4 OS=Mus musculus GN=Ntn4 PE=2 SV=2	1	3	0.63	5.30E+05	4.32E+05	8.22E+05	6.40E+05	4.41E+05	5.40E+05	6.54E+05	6.49E+05	4.24E+05	8.83E+05	4.79E+05	3.20E+05	5.02E+05	3.04E-06	1.65E-06	1.04E-18	2.34E-11	9.73E-07	3.80E-08	1.15E-11	8.99E-11	2.46E-07	1.79E-16	1.40E-12	2.14E-04	8.96E-10	5	3	3	NET4_MOUSE, SP:Q9JI33	SP|Q9JI33|NET4_MOUSE
Q5YD48	A1CF_MOUSE	APOBEC1 complementation factor OS=Mus musculus GN=A1cf PE=2 SV=2	1	12	0.82	9.27E+05	1.05E+06	6.78E+05	1.03E+06	8.14E+05	6.59E+05	8.43E+05	6.85E+05	5.11E+05	7.43E+05	8.97E+05	5.36E+05	7.15E+05	1.35E-09	4.18E-13	5.28E-16	6.97E-17	6.65E-11	1.46E-09	1.97E-14	2.92E-11	1.72E-08	2.57E-14	2.47E-21	2.79E-06	4.46E-13	5	12	12	A1CF_MOUSE, SP:Q5YD48	SP|Q5YD48|A1CF_MOUSE
Q7TSK7	ATL2_MOUSE	ADAMTS-like protein 2 OS=Mus musculus GN=Adamtsl2 PE=2 SV=1	1	6	0.81	1.01E+06	1.03E+06	4.79E+05	7.18E+05	1.04E+06	1.24E+06	9.90E+05	1.00E+06	5.94E+05	7.76E+05	9.45E+05	7.54E+05	9.15E+05	2.36E-10	4.89E-13	4.25E-12	1.29E-12	1.70E-13	2.97E-16	9.67E-17	1.28E-15	1.27E-09	4.72E-15	8.37E-23	3.50E-08	2.72E-16	7	6	6	ATL2_MOUSE, SP:Q7TSK7	SP|Q7TSK7|ATL2_MOUSE
O54834	RHG06_MOUSE	Rho GTPase-activating protein 6 OS=Mus musculus GN=Arhgap6 PE=1 SV=3	1	1	0.55	6.92E+05	1.05E+06	6.96E+05	1.28E+06	7.73E+05	7.64E+05	7.52E+05	9.45E+05	7.39E+05	7.86E+05	6.87E+05	6.33E+05	7.29E+05	1.25E-07	4.07E-13	2.36E-16	2.33E-20	1.90E-10	9.27E-11	4.07E-13	9.95E-15	2.13E-11	5.12E-15	4.79E-17	4.14E-07	2.84E-13	4	1	1	RHG06_MOUSE, SP:O54834	SP|O54834|RHG06_MOUSE
Q3UIJ9	MYZAP_MOUSE	Myocardial zonula adherens protein OS=Mus musculus GN=Myzap PE=1 SV=1	2	2	1	3.87E+04	4.45E+04	3.84E+04	4.40E+04	3.10E+04	4.20E+04	3.31E+04	5.40E+04	4.43E+04	3.78E+04	4.42E+04	2.95E+04	4.58E+04	0.109	0.066	0.025	0.04	0.102	0.065	0.063	0.031	0.05	0.041	0.015	0.141	0.033	2	1, 2	1, 2	MYZAP_MOUSE, SP:Q3UIJ9	SP|Q3UIJ9|MYZAP_MOUSE
P07361	A1AG2_MOUSE	Alpha-1-acid glycoprotein 2 OS=Mus musculus GN=Orm2 PE=1 SV=1	1	3	0.99	4.51E+03	1.83E+04	1.09E+03	2.31E+04	1.00E+03	6.83E+03	5.34E+03	5.49E+03	1.98E+03	4.50E+04	1.06E+04	0.00E+00	1.83E+04	0.337	0.167	0.373	0.101	0.409	0.27	0.269	0.275	0.364	0.029	0.144	0.5	0.122	2	3	3	A1AG2_MOUSE, SP:P07361	SP|P07361|A1AG2_MOUSE
Q9CZ42	NNRD_MOUSE	ATP-dependent (S)-NAD(P)H-hydrate dehydratase OS=Mus musculus GN=Carkd PE=1 SV=1	2	12	1	1.88E+05	2.15E+05	1.53E+05	1.57E+05	1.83E+05	1.78E+05	1.85E+05	1.60E+05	1.34E+05	1.52E+05	1.52E+05	1.50E+05	1.85E+05	0.003	4.78E-04	4.95E-05	4.59E-04	0.001	9.09E-04	1.49E-04	6.60E-04	0.002	2.55E-04	3.15E-05	0.008	1.09E-04	2, 5	1, 12	1, 12	NNRD_MOUSE, SP:Q9CZ42	SP|Q9CZ42|NNRD_MOUSE
Q61644	PACN1_MOUSE	Protein kinase C and casein kinase substrate in neurons protein 1 OS=Mus musculus GN=Pacsin1 PE=1 SV=1	1	4	0.66	1.08E+05	1.07E+05	8.42E+04	9.91E+04	1.14E+05	1.27E+05	1.46E+05	1.37E+05	8.90E+04	1.86E+05	1.19E+05	7.85E+04	1.18E+05	0.02	0.01	0.002	0.004	0.007	0.004	6.81E-04	0.002	0.01	6.31E-05	2.00E-04	0.04	0.002	4	4	4	PACN1_MOUSE, SP:Q61644	SP|Q61644|PACN1_MOUSE
P00848	ATP6_MOUSE	ATP synthase subunit a OS=Mus musculus GN=Mtatp6 PE=2 SV=1	1	12	0.95	6.41E+04	3.91E+04	1.38E+05	9.36E+04	5.30E+04	7.65E+04	1.37E+05	7.45E+04	3.73E+04	2.25E+05	9.21E+04	3.56E+04	8.94E+04	0.056	0.079	1.10E-04	0.005	0.048	0.02	9.60E-04	0.014	0.066	1.27E-05	9.03E-04	0.119	0.005	5	12	12	ATP6_MOUSE, SP:P00848	SP|P00848|ATP6_MOUSE

Q8R127	SCPDL_MOUSE	Saccharopine dehydrogenase-like oxidoreductase OS=Mus musculus GN=Sccpdh PE=2 SV=1	2	4	1	2.65E+05	2.35E+05
Q14B46	RTKN2_MOUSE	Rhotekin-2 OS=Mus musculus GN=Rtkn2 PE=2 SV=2	1	2	0.69	1.25E+05	8.82E+04
Q8BLR5	PSD4_MOUSE	PH and SEC7 domain-containing protein 4 OS=Mus musculus GN=Psd4 PE=2 SV=1	1	6	0.52	1.23E+05	1.43E+05
Q63880	EST3A_MOUSE	Carboxylesterase 3A OS=Mus musculus GN=Ces3a PE=1 SV=2	1	8	0.52	1.62E+05	1.34E+05
Q9D8T7	SLIRP_MOUSE	SRA stem-loop-interacting RNA-binding protein, mitochondrial OS=Mus musculus GN=Slirp PE=1 SV=2	1	11	0.99	7.33E+04	8.60E+04
A2A9C3	SZT2_MOUSE	Protein SZT2 OS=Mus musculus GN=Szt2 PE=1 SV=1	1	2	0.69	8.67E+04	5.60E+04
Q8CDR2	RSH6A_MOUSE	Radial spoke head protein 6 homolog A OS=Mus musculus GN=Rsph6a PE=2 SV=1	1	4	0.54	1.56E+05	1.19E+05
Q8C456	FRITZ_MOUSE	WD repeat-containing and planar cell polarity effector protein fritz homolog OS=Mus musculus GN=Wdpcp PE=2 SV=1	1	1	0.76	5.43E+04	7.90E+04
Q810B7	SLIK5_MOUSE	SLIT and NTRK-like protein 5 OS=Mus musculus GN=Slitrk5 PE=2 SV=1	1	1	0.94	1.94E+05	2.04E+05
Q9D1C3	PREY_MOUSE	Protein preY, mitochondrial OS=Mus musculus GN=Prey PE=2 SV=1	1	6	0.99	3.05E+05	2.07E+05
Q7M723	TR104_MOUSE	Taste receptor type 2 member 104 OS=Mus musculus GN=Tas2r104 PE=2 SV=1	1	8	0.75	1.05E+05	9.81E+04
Q8C9V1	TB10C_MOUSE	Carabin OS=Mus musculus GN=Tbc1d10c PE=2 SV=1	1	7	0.72	2.42E+05	2.62E+05
Q5NCY0	KDM6B_MOUSE	Lysine-specific demethylase 6B OS=Mus musculus GN=Kdm6b PE=2 SV=1	1	3	0.94	1.76E+05	1.74E+05
Q8QZR1	ATTY_MOUSE	Tyrosine aminotransferase OS=Mus musculus GN=Tat PE=1 SV=1	1	3	0.98	1.11E+05	1.26E+05
D3YZF7	VS10L_MOUSE	V-set and immunoglobulin domain-containing protein 10-like OS=Mus musculus GN=Vsig10l PE=4 SV=1	1	10	0.96	1.41E+04	3.37E+04
Q9D7J4	COX20_MOUSE	Cytochrome c oxidase protein 20 homolog OS=Mus musculus GN=Cox20 PE=2 SV=1	1	5	0.99	5.62E+04	1.03E+05
Q0VF22	CC138_MOUSE	Coiled-coil domain-containing protein 138 OS=Mus musculus GN=Ccdc138 PE=2 SV=1	1	1	0.93	5.57E+05	3.52E+05
Q8BU51	BPIB6_MOUSE	BPI fold-containing family B member 6 OS=Mus musculus GN=Bpifb6 PE=2 SV=3	1	1	0.62	2.44E+04	2.84E+04
Q80U40	RIMB2_MOUSE	RIMS-binding protein 2 OS=Mus musculus GN=Rimbp2 PE=1 SV=3	1	5	0.63	8.62E+04	1.12E+05
Q62147	SSPN_MOUSE	Sarcospan OS=Mus musculus GN=Sspn PE=2 SV=1	1	5	0.99	2.12E+04	1.18E+04
Q8VI40	TRI60_MOUSE	Tripartite motif-containing protein 60 OS=Mus musculus GN=Trim60 PE=2 SV=1	1	1	0.94	1.31E+05	1.26E+05
Q9EQJ9	MAGI3_MOUSE	Membrane-associated guanylate kinase, WW and PDZ domain-containing protein 3 OS=Mus musculus GN=Magi3 PE=1 SV=2	1	6	0.95	1.01E+04	1.73E+04
Q9D1B8	DCD2C_MOUSE	Doublecortin domain-containing protein 2C OS=Mus musculus GN=Dcdc2c PE=2 SV=2	1	1	0.69	8.40E+04	6.60E+04
O70576	STAG3_MOUSE	Cohesin subunit SA-3 OS=Mus musculus GN=Stag3 PE=1 SV=2	1	1	0.99	1.42E+05	9.33E+04
Q61220	NELL2_MOUSE	Protein kinase C-binding protein NELL2 OS=Mus musculus GN=Nell2 PE=2 SV=2	1	2	0.77	6.28E+04	7.69E+04
E9PZQ0	RYR1_MOUSE	Ryanodine receptor 1 OS=Mus musculus GN=Ryr1 PE=1 SV=1	1	4	0.67	6.83E+04	5.22E+04
P55271	CDN2B_MOUSE	Cyclin-dependent kinase 4 inhibitor B OS=Mus musculus GN=Cdkn2b PE=1 SV=1	1	11	0.98	1.10E+05	7.04E+04
Q9WVB6	LENEP_MOUSE	Lens epithelial cell protein LEP503 OS=Mus musculus GN=Lenep PE=4 SV=1	1	6	0.53	2.60E+04	2.57E+04
Q6NXW6	RAD17_MOUSE	Cell cycle checkpoint protein RAD17 OS=Mus musculus GN=Rad17 PE=1 SV=2	1	5	0.62	4.49E+04	3.13E+04
Q8CAK1	CAF17_MOUSE	Putative transferase CAF17 homolog, mitochondrial OS=Mus musculus GN=Iba57 PE=2 SV=1	2	4	1	6.07E+04	6.52E+04
Q8CDJ3	BAKOR_MOUSE	Beclin 1-associated autophagy-related key regulator OS=Mus musculus GN=Atg14 PE=1 SV=1	1	1	0.83	2.83E+04	1.92E+04
Q9JLG8	CAN15_MOUSE	Calpain-15 OS=Mus musculus GN=Solh PE=1 SV=1	1	2	0.59	1.48E+04	1.64E+04
Q91YM4	TBRG4_MOUSE	Protein TBRG4 OS=Mus musculus GN=Tbrg4 PE=2 SV=1	1	9	0.99	1.05E+05	8.89E+04
P42925	PXMP2_MOUSE	Peroxisomal membrane protein 2 OS=Mus musculus GN=Pxmp2 PE=2 SV=2	1	12	0.99	9.37E+04	7.98E+04
Q5FW52	MLIP_MOUSE	Muscular LMNA-interacting protein OS=Mus musculus GN=Mlip PE=1 SV=1	1	2	0.54	7.42E+04	6.54E+04
Q9JHG2	RCAN2_MOUSE	Calcipressin-2 OS=Mus musculus GN=Rcan2 PE=2 SV=1	1	2	0.7	4.32E+05	3.56E+05
Q8R3P6	CO044_MOUSE	UPF0464 protein C15orf44 homolog OS=Mus musculus PE=2 SV=1	1	7	0.58	3.95E+04	3.03E+04
P59808	SASH1_MOUSE	SAM and SH3 domain-containing protein 1 OS=Mus musculus GN=Sash1 PE=1 SV=1	1	8	1	6.67E+04	4.22E+04
Q7TN75	PEG10_MOUSE	Retrotransposon-derived protein PEG10 OS=Mus musculus GN=Peg10 PE=1 SV=2	1	2	0.72	7.09E+04	7.42E+04
Q60590	A1AG1_MOUSE	Alpha-1-acid glycoprotein 1 OS=Mus musculus GN=Orm1 PE=1 SV=1	1	3	0.98	2.10E+04	4.01E+04
Q6A000	K0753_MOUSE	Uncharacterized protein KIAA0753 OS=Mus musculus GN=Kiaa0753 PE=2 SV=2	1	4	0.96	2.21E+04	2.22E+04
Q9WVQ5	MTNB_MOUSE	Probable methylthioribulose-1-phosphate dehydratase OS=Mus musculus GN=Apip PE=1 SV=1	1	4	1	5.11E+04	4.28E+04
Q9QZM0	UBQL2_MOUSE	Ubiquilin-2 OS=Mus musculus GN=Ubqln2 PE=1 SV=2	1	1	0.92	1.03E+04	3.04E+04
Q8BUY5	TIDC1_MOUSE	Translocase of inner mitochondrial membrane domain-containing protein 1 OS=Mus musculus GN=Timmdc1 PE=2 SV=1	2	12	1	1.44E+05	8.84E+04
Q8BMG7	RBGPR_MOUSE	Rab3 GTPase-activating protein non-catalytic subunit OS=Mus musculus GN=Rab3gap2 PE=1 SV=2	1	1	0.81	4.47E+04	4.23E+04
Q5GIG6	TNI3K_MOUSE	Serine/threonine-protein kinase TNNI3K OS=Mus musculus GN=Tnni3k PE=2 SV=4	1	1	0.99	1.69E+04	1.11E+04
Q3UHU5	CC165_MOUSE	Coiled-coil domain-containing protein 165 OS=Mus musculus GN=Ccdc165 PE=1 SV=1	1	6	0.96	3.99E+04	5.10E+04
Q8R4P9	MRP7_MOUSE	Multidrug resistance-associated protein 7 OS=Mus musculus GN=Abcc10 PE=2 SV=1	1	5	0.98	2.02E+03	7.24E+03
Q0KK55	VKIND_MOUSE	Protein very KIND OS=Mus musculus GN=Kndc1 PE=1 SV=2	1	1	0.55	2.71E+05	1.95E+05
Q3UHC2	LRRK1_MOUSE	Leucine-rich repeat serine/threonine-protein kinase 1 OS=Mus musculus GN=Lrrk1 PE=2 SV=1	1	2	0.92	1.47E+04	5.00E+04
P05366	SAA1_MOUSE	Serum amyloid A-1 protein OS=Mus musculus GN=Saa1 PE=2 SV=2	1	1	0.98	1.29E+03	1.24E+04
Q6URW6	MYH14_MOUSE	Myosin-14 OS=Mus musculus GN=Myh14 PE=1 SV=1	1	2	0.6	4.80E+04	4.94E+04
Q99LF4	RTCB_MOUSE	tRNA-splicing ligase RtcB homolog OS=Mus musculus GN=D10Wsu52e PE=2 SV=1	1	2	1	4.35E+04	2.89E+04
Q6ZWR6	SYNE1_MOUSE	Nesprin-1 OS=Mus musculus GN=Syne1 PE=1 SV=2	1	7	0.98	2.01E+04	2.06E+04
Q8K3F2	MMP21_MOUSE	Matrix metalloproteinase-21 OS=Mus musculus GN=Mmp21 PE=2 SV=1	1	2	0.69	5.63E+04	4.66E+04
Q8BYL4	SYYM_MOUSE	Tyrosine--tRNA ligase, mitochondrial OS=Mus musculus GN=Yars2 PE=2 SV=2	1	1	0.99	2.52E+04	2.25E+04
Q8BKG3	PTK7_MOUSE	Inactive tyrosine-protein kinase 7 OS=Mus musculus GN=Ptk7 PE=1 SV=1	1	9	0.69	1.76E+04	3.02E+04
Q9ES82	POPD2_MOUSE	Popeye domain-containing protein 2 OS=Mus musculus GN=Popdc2 PE=2 SV=1	1	4	0.99	1.01E+04	2.18E+04
Q91ZC4	MRGA8_MOUSE	Mas-related G-protein coupled receptor member A8 OS=Mus musculus GN=Mrgpra8 PE=2 SV=1	1	1	0.98	0.00E+00	8.43E+03
Q6PIU9	YJ005_MOUSE	Uncharacterized protein FLJ45252 homolog OS=Mus musculus PE=1 SV=2	1	1	0.81	3.36E+04	2.90E+04
Q9ESN5	CENPK_MOUSE	Centromere protein K OS=Mus musculus GN=Cenpk PE=1 SV=2	1	1	1	1.93E+04	5.60E+04
Q63810	CANB1_MOUSE	Calcineurin subunit B type 1 OS=Mus musculus GN=Ppp3r1 PE=1 SV=3	1	1	0.95	6.40E+04	7.24E+04

Q91ZC4	1.23E+04	6.85E+03	1.70E+03	5.25E+03	1.26E+04	4.72E+03	8.97E+03	9.60E+03	1.46E+04	9.09E+02	1.32E+04	0.5	0.256	0.134	0.244	0.382	0.295	0.172	0.29	0.23	0.19	0.106	0.425	0.16	3	1	1	MRGA8_MOUSE, SP:Q91ZC4	SP|Q91ZC4|MRGA8_MOUSE
Q6PIU9	1.31E+04	1.69E+04	3.06E+04	2.78E+04	2.12E+04	1.99E+04	2.07E+04	2.45E+04	1.93E+04	2.36E+04	1.93E+04	0.126	0.112	0.126	0.138	0.103	0.108	0.11	0.128	0.131	0.081	0.076	0.168	0.115	2	1	1	YJ005_MOUSE, SP:Q6PIU9	SP|Q6PIU9|YJ005_MOUSE
Q9ESN5	4.29E+04	5.63E+04	3.94E+04	2.55E+04	3.46E+04	5.35E+04	6.69E+04	4.25E+04	4.90E+04	3.49E+04	4.30E+04	0.192	0.046	0.02	0.024	0.076	0.119	0.059	0.032	0.022	0.033	0.011	0.122	0.037	4	1	1	CENPK_MOUSE, SP:Q9ESN5	SP|Q9ESN5|CENPK_MOUSE
Q63810	1.87E+04	3.10E+04	4.19E+04	3.68E+04	3.39E+04	3.65E+04	2.25E+04	2.95E+04	3.21E+04	5.49E+04	9.50E+04	0.057	0.028	0.093	0.074	0.07	0.079	0.062	0.064	0.123	0.065	0.035	0.072	0.004	4	1	1	CANB1_MOUSE, SP:Q63810	SP|Q63810|CANB1_MOUSE

S2 Table B Details on all identified proteins in samples of the right ventricle

Swiss-Prot ID	Primary Protein Name	Protein Description	Squeeze Count	Sample count	ProteinTeller Probability	Intensity [base]	Intensity [SHAM 4d]
P58771	TPM1_MOUSE	Tropomyosin alpha-1 chain OS=Mus musculus GN=Tpm1 PE=1 SV=1 [MASS=32680]	6	13	1.0	1.25E+11	1.35E+11
P58281	OPA1_MOUSE	Dynamin-like 120 kDa protein, mitochondrial OS=Mus musculus GN=Opa1 PE=1 SV=1 [MASS=111338]	13	13	1.0	2.50E+09	2.69E+09
P63038	CH60_MOUSE	60 kDa heat shock protein, mitochondrial OS=Mus musculus GN=Hspd1 PE=1 SV=1 [MASS=60955]	23	13	1.0	4.43E+10	4.70E+10
Q8VEM8	MPCP_MOUSE	Phosphate carrier protein, mitochondrial OS=Mus musculus GN=Slc25a3 PE=1 SV=1 [MASS=39632]	12	13	1.0	2.46E+10	3.65E+10
O70468	MYPC3_MOUSE	Myosin-binding protein C, cardiac-type OS=Mus musculus GN=Mybpc3 PE=2 SV=1 [MASS=140631]	38	13	1.0	7.36E+10	8.60E+10
Q9D855	QCR7_MOUSE	Cytochrome b-c1 complex subunit 7 OS=Mus musculus GN=Uqcrb PE=1 SV=3 [MASS=13527]	6	13	1.0	2.27E+10	2.30E+10
Q9CQZ5	NDUA6_MOUSE	NADH dehydrogenase [ubiquinone] 1 alpha subcomplex subunit 6 OS=Mus musculus GN=Ndufa6 PE=1 SV=1 [MASS=15283]	7	13	1.0	9.93E+09	8.75E+09
Q9Z0X1	AIFM1_MOUSE	Apoptosis-inducing factor 1, mitochondrial OS=Mus musculus GN=Aifm1 PE=1 SV=1 [MASS=66765]	10	13	1.0	5.07E+09	5.81E+09
P14206	RSSA_MOUSE	40S ribosomal protein SA OS=Mus musculus GN=Rpsa PE=1 SV=4 [MASS=32838]	5	13	1.0	1.34E+09	1.68E+09
Q99JY0	ECHB_MOUSE	Trifunctional enzyme subunit beta, mitochondrial OS=Mus musculus GN=Hadhb PE=1 SV=1 [MASS=51386]	18	13	1.0	8.27E+10	9.30E+10
Q91VD9	NDUS1_MOUSE	NADH-ubiquinone oxidoreductase 75 kDa subunit, mitochondrial OS=Mus musculus GN=Ndufs1 PE=1 SV=1 [MASS=79748]	24	13	1.0	4.79E+10	5.20E+10
P47738	ALDH2_MOUSE	Aldehyde dehydrogenase, mitochondrial OS=Mus musculus GN=Aldh2 PE=1 SV=1 [MASS=56537]	12	13	1.0	7.78E+09	7.95E+09
P56480	ATPB_MOUSE	ATP synthase subunit beta, mitochondrial OS=Mus musculus GN=Atp5b PE=1 SV=2 [MASS=56300]	32	13	1.0	6.54E+11	7.07E+11
P48962	ADT1_MOUSE	ADP/ATP translocase 1 OS=Mus musculus GN=Slc25a4 PE=1 SV=4 [MASS=32904]	8	13	1.0	7.83E+10	1.07E+11
P63017	HSP7C_MOUSE	Heat shock cognate 71 kDa protein OS=Mus musculus GN=Hspa8 PE=1 SV=1 [MASS=70871]	12	13	1.0	1.85E+10	1.98E+10
O35459	ECH1_MOUSE	Delta(3,5)-Delta(2,4)-dienoyl-CoA isomerase, mitochondrial OS=Mus musculus GN=Ech1 PE=2 SV=1 [MASS=36118]	10	13	1.0	1.37E+10	1.26E+10
Q02566	MYH6_MOUSE	Myosin-6 OS=Mus musculus GN=Myh6 PE=1 SV=2 [MASS=223564]	27	13	1.0	3.65E+11	4.60E+11
P54071	IDHP_MOUSE	Isocitrate dehydrogenase [NADP], mitochondrial OS=Mus musculus GN=Idh2 PE=1 SV=3 [MASS=50906]	22	13	1.0	1.16E+11	1.42E+11
P62900	RL31_MOUSE	60S ribosomal protein L31 OS=Mus musculus GN=Rpl31 PE=2 SV=1 [MASS=14463]	1	13	0.99	1.28E+08	2.20E+08
P16858	G3P_MOUSE	Glyceraldehyde-3-phosphate dehydrogenase OS=Mus musculus GN=Gapdh PE=1 SV=2 [MASS=35810]	13	13	1.0	5.78E+10	7.68E+10
Q91YT0	NDUV1_MOUSE	NADH dehydrogenase [ubiquinone] flavoprotein 1, mitochondrial OS=Mus musculus GN=Ndufv1 PE=1 SV=1 [MASS=50834]	15	13	1.0	1.50E+10	1.73E+10
P01887	B2MG_MOUSE	Beta-2-microglobulin OS=Mus musculus GN=B2m PE=1 SV=1 [MASS=13823]	1	10	0.99	2.01E+08	1.37E+08
Q9CR61	NDUB7_MOUSE	NADH dehydrogenase [ubiquinone] 1 beta subcomplex subunit 7 OS=Mus musculus GN=Ndufb7 PE=1 SV=3 [MASS=16331]	2	13	1.0	1.79E+09	1.98E+09
P47911	RL6_MOUSE	60S ribosomal protein L6 OS=Mus musculus GN=Rpl6 PE=1 SV=3 [MASS=33509]	2	12	1.0	8.18E+07	1.56E+08
Q9D0K2	SCOT1_MOUSE	Succinyl-CoA:3-ketoacid-coenzyme A transferase 1, mitochondrial OS=Mus musculus GN=Oxct1 PE=1 SV=1 [MASS=55988]	13	13	1.0	2.57E+10	3.07E+10
Q7TQ48	SRCA_MOUSE	Sarcalumenin OS=Mus musculus GN=Srl PE=1 SV=1 [MASS=99183]	25	13	1.0	4.81E+10	6.30E+10
Q8CAQ8	IMMT_MOUSE	Mitochondrial inner membrane protein OS=Mus musculus GN=Immt PE=1 SV=1 [MASS=83899]	24	13	1.0	2.39E+10	2.47E+10
Q01853	TERA_MOUSE	Transitional endoplasmic reticulum ATPase OS=Mus musculus GN=Vcp PE=1 SV=4 [MASS=89321]	13	13	1.0	3.48E+09	4.16E+09
P17710	HXK1_MOUSE	Hexokinase-1 OS=Mus musculus GN=Hk1 PE=1 SV=2 [MASS=108302]	9	13	1.0	1.64E+09	1.85E+09
Q99LC5	ETFA_MOUSE	Electron transfer flavoprotein subunit alpha, mitochondrial OS=Mus musculus GN=Etfa PE=1 SV=2 [MASS=35009]	16	13	1.0	7.37E+10	8.26E+10
Q8K1Z0	COQ9_MOUSE	Ubiquinone biosynthesis protein COQ9, mitochondrial OS=Mus musculus GN=Coq9 PE=1 SV=1 [MASS=35082]	4	13	1.0	7.29E+09	7.25E+09
P12787	COX5A_MOUSE	Cytochrome c oxidase subunit 5A, mitochondrial OS=Mus musculus GN=Cox5a PE=1 SV=2 [MASS=16101]	5	13	1.0	2.15E+10	2.93E+10
Q9D1G3	HHATL_MOUSE	Protein-cysteine N-palmitoyltransferase HHAT-like protein OS=Mus musculus GN=Hhatl PE=1 SV=2 [MASS=56430]	4	13	1.0	9.94E+08	1.24E+09
Q2TPA8	HSDL2_MOUSE	Hydroxysteroid dehydrogenase-like protein 2 OS=Mus musculus GN=Hsdl2 PE=2 SV=1 [MASS=54208]	6	13	1.0	2.07E+09	1.74E+09
P99029	PRDX5_MOUSE	Peroxiredoxin-5, mitochondrial OS=Mus musculus GN=Prdx5 PE=1 SV=2 [MASS=21897]	8	13	1.0	1.58E+10	1.70E+10
Q8VDN2	AT1A1_MOUSE	Sodium/potassium-transporting ATPase subunit alpha-1 OS=Mus musculus GN=Atp1a1 PE=1 SV=1 [MASS=112981]	12	13	1.0	6.65E+09	7.55E+09
Q9CQA3	DHSB_MOUSE	Succinate dehydrogenase [ubiquinone] iron-sulfur subunit, mitochondrial OS=Mus musculus GN=Sdhb PE=1 SV=1 [MASS=31814]	10	13	1.0	2.19E+10	2.50E+10
P97807	FUMH_MOUSE	Fumarate hydratase, mitochondrial OS=Mus musculus GN=Fh PE=1 SV=2 [MASS=54370]	15	13	1.0	4.91E+10	6.02E+10
Q9DB20	ATPO_MOUSE	ATP synthase subunit O, mitochondrial OS=Mus musculus GN=Atp5o PE=1 SV=1 [MASS=23363]	10	13	1.0	5.58E+10	6.74E+10
P26443	DHE3_MOUSE	Glutamate dehydrogenase 1, mitochondrial OS=Mus musculus GN=Glud1 PE=1 SV=1 [MASS=61336]	10	13	1.0	1.58E+09	1.71E+09
P06151	LDHA_MOUSE	L-lactate dehydrogenase A chain OS=Mus musculus GN=Ldha PE=1 SV=3 [MASS=36498]	12	13	1.0	1.65E+10	2.00E+10
Q8BH95	ECHM_MOUSE	Enoyl-CoA hydratase, mitochondrial OS=Mus musculus GN=Echs1 PE=1 SV=1 [MASS=31474]	8	13	1.0	1.19E+10	1.32E+10
Q03265	ATPA_MOUSE	ATP synthase subunit alpha, mitochondrial OS=Mus musculus GN=Atp5a1 PE=1 SV=1 [MASS=59752]	24	13	1.0	3.11E+11	3.41E+11
O35129	PHB2_MOUSE	Prohibitin-2 OS=Mus musculus GN=Phb2 PE=1 SV=1 [MASS=33296]	9	13	1.0	6.75E+09	9.90E+09
Q921G7	ETFD_MOUSE	Electron transfer flavoprotein-ubiquinone oxidoreductase, mitochondrial OS=Mus musculus GN=Etfdh PE=1 SV=1 [MASS=68090]	16	13	1.0	2.65E+10	2.49E+10
Q9CPU4	MGST3_MOUSE	Microsomal glutathione S-transferase 3 OS=Mus musculus GN=Mgst3 PE=1 SV=1 [MASS=16958]	1	13	1.0	2.54E+08	4.91E+08
Q99KI0	ACON_MOUSE	Aconitate hydratase, mitochondrial OS=Mus musculus GN=Aco2 PE=1 SV=1 [MASS=85463]	28	13	1.0	1.91E+11	2.07E+11
P57776	EF1D_MOUSE	Elongation factor 1-delta OS=Mus musculus GN=Eef1d PE=1 SV=3 [MASS=31293]	2	12	1.0	7.07E+08	5.15E+08
P20029	GRP78_MOUSE	78 kDa glucose-regulated protein OS=Mus musculus GN=Hspa5 PE=1 SV=3 [MASS=72422]	10	13	1.0	6.85E+09	6.32E+09
P67984	RL22_MOUSE	60S ribosomal protein L22 OS=Mus musculus GN=Rpl22 PE=2 SV=2 [MASS=14759]	1	2	0.91	1.96E+08	2.34E+08
P35486	ODPA_MOUSE	Pyruvate dehydrogenase E1 component subunit alpha, somatic form, mitochondrial OS=Mus musculus GN=Pdha1 PE=1 SV=1 [MASS=43231]	12	13	1.0	1.87E+10	1.89E+10
Q01768	NDKB_MOUSE	Nucleoside diphosphate kinase B OS=Mus musculus GN=Nme2 PE=1 SV=1 [MASS=17363]	5	13	1.0	1.11E+10	1.10E+10
O55143	AT2A2_MOUSE	Sarcoplasmic/endoplasmic reticulum calcium ATPase 2 OS=Mus musculus GN=Atp2a2 PE=1 SV=2 [MASS=114857]	21	13	1.0	6.45E+10	7.53E+10
Q3UIU2	NDUB6_MOUSE	NADH dehydrogenase [ubiquinone] 1 beta subcomplex subunit 6 OS=Mus musculus GN=Ndufb6 PE=2 SV=3 [MASS=15515]	1	9	0.99	4.54E+07	1.63E+08
P45952	ACADM_MOUSE	Medium-chain specific acyl-CoA dehydrogenase, mitochondrial OS=Mus musculus GN=Acadm PE=1 SV=1 [MASS=46481]	15	13	1.0	6.39E+10	6.45E+10
Q60932	VDAC1_MOUSE	Voltage-dependent anion-selective channel protein 1 OS=Mus musculus GN=Vdac1 PE=1 SV=3 [MASS=32351]	12	13	1.0	4.46E+10	4.02E+10
P03888	NU1M_MOUSE	NADH-ubiquinone oxidoreductase chain 1 OS=Mus musculus GN=Mtnd1 PE=1 SV=3 [MASS=36059]	4	13	1.0	3.43E+09	4.36E+09
Q8BH59	CMC1_MOUSE	Calcium-binding mitochondrial carrier protein Aralar1 OS=Mus musculus GN=Slc25a12 PE=1 SV=1 [MASS=74569]	17	13	1.0	1.17E+10	1.67E+10

Swiss-Prot ID	Intensity [SHAM 14d]	Intensity [SHAM 21d]	Intensity [SHAM 28d]	Intensity [SHAM 42d]	Intensity [SHAM 56d]	Intensity [TAC 4d]	Intensity [TAC 14d]	Intensity [TAC 21d]	Intensity [TAC 28d]	Intensity [TAC 42d]	Intensity [TAC 56d]	P-Value [base]	P-Value [SHAM 14d]	P-Value [SHAM 21d]	P-Value [SHAM 28d]	P-Value [SHAM 42d]	P-Value [SHAM 4d]	P-Value [SHAM 56d]	P-Value [TAC 14d]	P-Value [TAC 21d]	P-Value [TAC 28d]	P-Value [TAC 42d]	P-Value [TAC 4d]	P-Value [TAC 56d]	Isotope Count	Peptide Count Score	Scan Total	Protein Names	Protein Code
P58771	1.39E+11	1.16E+11	1.36E+11	1.31E+11	1.25E+11	1.19E+11	1.23E+11	1.34E+11	1.29E+11	1.36E+11	1.39E+11	0	0	0	0	0	0	0	0	0	0	0	0	0	3, 5, 4, 6, 5, 6, 6	11, 13, 6, 13, 8, 11, 12, 8, 12	11, 12, 24, 17, 22, 23, 25, 35	TPM1_MOUSE, SP:P58771	SP|P58771|TPM1_MOUSE
P58281	2.51E+09	2.81E+09	2.80E+09	2.70E+09	2.81E+09	2.46E+09	2.43E+09	2.57E+09	2.51E+09	2.29E+09	2.20E+09	1.42E-40	0	0	8.47E-30	4.96E-27	0	1.85E-38	1.07E-35	1.35E-27	0	1.87E-26	4.66E-32	7.35E-14	2, 3, 4, 5, 6	1, 10, 13, 3, 4, 5, 6, 7, 8, 9	1, 10, 13, 3, 4, 5, 6, 7, 8, 9	OPA1_MOUSE, SP:P58281	SP|P58281|OPA1_MOUSE
P63038	4.75E+10	4.82E+10	5.57E+10	4.97E+10	5.01E+10	4.53E+10	4.36E+10	4.67E+10	4.32E+10	4.58E+10	4.36E+10	0	0	0	0	0	0	0	0	0	0	0	0	0	2, 2, 5, 3, 3, 4, 3, 5, 4, 4, 6, 5, 5, 7, 5, 8, 7	1, 1, 11, 10, 12, 12, 13, 13, 2, 13, 2, 3, 3, 4, 4, 13, 7, 9	1, 1, 11, 10, 12, 13, 13, 13, 14, 13, 2, 13, 4, 13, 54, 15, 2, 3, 24, 3, 54, 7, 9	CH60_MOUSE, SP:P63038	SP|P63038|CH60_MOUSE
Q8VEM8	3.23E+10	2.67E+10	2.51E+10	3.00E+10	2.80E+10	3.03E+10	2.24E+10	2.80E+10	2.69E+10	2.80E+10	2.58E+10	0	0	0	0	0	0	0	0	0	0	0	0	0	2, 3, 3, 6, 4, 5, 6, 7	11, 12, 13, 3, 4, 6, 11, 9	11, 11, 6, 12, 13, 15, 3, 4, 9	MPCP_MOUSE, SP:Q8VEM8	SP|Q8VEM8|MPCP_MOUSE
O70468	8.39E+10	7.86E+10	7.45E+10	7.84E+10	7.09E+10	7.76E+10	7.05E+10	8.09E+10	7.52E+10	8.53E+10	8.92E+10	0	0	0	0	0	0	0	0	0	0	0	0	0	2, 2, 5, 3, 3, 4, 3, 6, 3, 7, 4, 4, 5, 4, 6, 5, 5, 7, 6, 6, 7, 7	1, 1, 10, 1, 7, 10, 13, 10, 10, 11, 10, 13, 11, 12, 13, 3, 4, 5, 6, 13, 7, 8, 9, 12	1, 1, 10, 10, 10, 11, 10, 13, 10, 22, 11, 12, 12, 9, 13, 13, 17, 13, 19, 14, 17, 17, 6, 4, 5, 7, 8	MYPC3_MOUSE, SP:O70468	SP|O70468|MYPC3_MOUSE
Q9D855	2.33E+10	2.40E+10	2.44E+10	2.30E+10	2.39E+10	2.11E+10	1.94E+10	2.22E+10	2.17E+10	2.09E+10	2.11E+10	0	0	0	0	0	0	0	0	0	0	0	0	0	2, 4, 4, 5, 6	1, 10, 12, 13, 6, 13, 8	1, 12, 13, 13, 6, 14, 17	QCR7_MOUSE, SP:Q9D855	SP|Q9D855|QCR7_MOUSE
Q9CQZ5	8.78E+09	1.06E+10	9.03E+09	6.98E+09	9.02E+09	7.35E+09	7.15E+09	7.89E+09	6.79E+09	8.34E+09	6.79E+09	0	0	0	0	0	0	0	0	0	0	0	0	0	2, 2, 3, 4, 4, 6	11, 13, 2, 3, 8, 9	11, 13, 3, 8, 9	NDUA6_MOUSE, SP:Q9CQZ5	SP|Q9CQZ5|NDUA6_MOUSE
Q9Z0X1	5.35E+09	4.92E+09	5.12E+09	5.44E+09	4.78E+09	5.48E+09	4.34E+09	5.00E+09	4.87E+09	4.62E+09	5.71E+09	0	0	0	0	0	0	0	0	0	0	0	0	1.43E-37	2, 4, 3, 4, 5, 6	12, 13, 2, 13, 6, 7	12, 13, 13, 2, 6, 7	AIFM1_MOUSE, SP:Q9Z0X1	SP|Q9Z0X1|AIFM1_MOUSE

Q60930	VDAC2_MOUSE	Voltage-dependent anion-selective channel protein 2 OS=Mus musculus GN=Vdac2 PE=1 SV=2 [MASS=31733]	9	13	1.0	1.64E+10	1.50E+10	1.53E+10	1.57E+10	1.48E+10	1.46E+10	1.38E+10	1.47E+10	1.34E+10	1.54E+10	1.25E+10	1.48E+10	1.44E+10	0	0	[illegible]
P50462	CSRP3_MOUSE	Cysteine and glycine-rich protein 3 OS=Mus musculus GN=Csrp3 PE=2 SV=1 [MASS=20895]	4	13	1.0	1.08E+10	1.61E+10	1.73E+10	1.21E+10	1.15E+10	1.11E+10	1.15E+10	1.18E+10	1.56E+10	1.72E+10	1.75E+10	2.13E+10	2.23E+10	0	0	[illegible]
P43274	H14_MOUSE	Histone H1.4 OS=Mus musculus GN=Hist1h1e PE=1 SV=2 [MASS=21977]	1	2	1.0	2.23E+08	1.82E+08	4.50E+08	3.04E+08	4.16E+08	2.52E+08	2.39E+08	2.63E+08	2.33E+08	2.66E+08	2.53E+08	2.65E+08	2.26E+08	2.55E-02	1.07E-06	[illegible]
P03921	NU5M_MOUSE	NADH-ubiquinone oxidoreductase chain 5 OS=Mus musculus GN=Mtnd5 PE=2 SV=2 [MASS=68440]	4	13	1.0	6.29E+09	6.69E+09	6.34E+09	7.11E+09	5.05E+09	5.49E+09	6.38E+09	6.43E+09	6.07E+09	5.45E+09	5.90E+09	6.06E+09	4.63E+09	0	0	[illegible]
Q9DCZ4	APOO_MOUSE	Apolipoprotein O OS=Mus musculus GN=Apoo PE=2 SV=1 [MASS=24136]	5	13	1.0	2.03E+09	3.27E+09	2.61E+09	2.22E+09	2.14E+09	2.53E+09	2.65E+09	2.87E+09	2.29E+09	2.36E+09	2.61E+09	1.95E+09	1.76E+09	3.60E-32	0	[illegible]
Q8K2B3	DHSA_MOUSE	Succinate dehydrogenase [ubiquinone] flavoprotein subunit, mitochondrial OS=Mus musculus GN=Sdha PE=1 SV=1 [MASS=72585]	21	13	1.0	4.61E+10	4.40E+10	5.02E+10	4.86E+10	4.54E+10	4.30E+10	4.77E+10	3.89E+10	4.24E+10	4.24E+10	3.89E+10	5.05E+10	4.37E+10	0	0	[illegible]
Q9JHI5	IVD_MOUSE	Isovaleryl-CoA dehydrogenase, mitochondrial OS=Mus musculus GN=Ivd PE=1 SV=1 [MASS=46325]	12	13	1.0	1.24E+10	1.17E+10	1.13E+10	1.24E+10	1.43E+10	1.32E+10	1.39E+10	1.07E+10	1.03E+10	1.09E+10	1.10E+10	1.15E+10	1.04E+10	0	0	[illegible]
O08749	DLDH_MOUSE	Dihydrolipoyl dehydrogenase, mitochondrial OS=Mus musculus GN=Dld PE=1 SV=2 [MASS=54272]	11	13	1.0	1.52E+10	1.86E+10	2.00E+10	1.52E+10	1.56E+10	1.78E+10	1.82E+10	1.57E+10	1.54E+10	1.61E+10	1.70E+10	1.50E+10	1.28E+10	0	0	[illegible]
Q8CC88	K0564_MOUSE	Uncharacterized protein KIAA0564 homolog OS=Mus musculus GN=Kiaa0564 PE=2 SV=2 [MASS=213420]	16	13	1.0	2.50E+09	2.34E+09	2.49E+09	2.63E+09	2.59E+09	2.47E+09	2.52E+09	2.15E+09	2.09E+09	2.13E+09	2.10E+09	2.20E+09	1.86E+09	1.02E-40	0	[illegible]
P11152	LIPL_MOUSE	Lipoprotein lipase OS=Mus musculus GN=Lpl PE=1 SV=2 [MASS=53126]	2	13	1.0	3.13E+08	3.76E+08	3.92E+08	3.98E+08	4.00E+08	3.85E+08	3.19E+08	4.76E+08	3.30E+08	3.76E+08	3.11E+08	3.44E+08	4.01E+08	4.68E-04	7.74E-06	[illegible]
P52825	CPT2_MOUSE	Carnitine O-palmitoyltransferase 2, mitochondrial OS=Mus musculus GN=Cpt2 PE=1 SV=1 [MASS=73927]	16	13	1.0	1.08E+10	1.19E+10	1.21E+10	1.14E+10	1.30E+10	1.28E+10	1.22E+10	1.18E+10	1.05E+10	1.06E+10	1.07E+10	1.15E+10	9.65E+09	0	0	[illegible]
P50544	ACADV_MOUSE	Very long-chain specific acyl-CoA dehydrogenase, mitochondrial OS=Mus musculus GN=Acadvl PE=1 SV=3 [MASS=70875]	29	13	1.0	7.85E+10	8.32E+10	8.20E+10	8.17E+10	9.27E+10	8.57E+10	8.78E+10	7.72E+10	7.31E+10	7.94E+10	7.49E+10	7.96E+10	6.76E+10	0	0	[illegible]
Q8BMS1	ECHA_MOUSE	Trifunctional enzyme subunit alpha, mitochondrial OS=Mus musculus GN=Hadha PE=1 SV=1 [MASS=82669]	35	13	1.0	1.47E+11	1.75E+11	1.86E+11	1.73E+11	2.03E+11	2.00E+11	1.92E+11	1.68E+11	1.57E+11	1.61E+11	1.59E+11	1.71E+11	1.53E+11	0	0	[illegible]
Q61554	FBN1_MOUSE	Fibrillin-1 OS=Mus musculus GN=Fbn1 PE=1 SV=1 [MASS=312266]	2	13	1.0	1.73E+08	2.05E+08	1.97E+08	3.33E+08	2.38E+08	2.41E+08	2.41E+08	2.00E+08	1.96E+08	2.00E+08	2.09E+08	1.88E+08	3.96E+08	1.30E-01	2.72E-02	[illegible]
Q8BMF4	ODP2_MOUSE	Dihydrolipoyllysine-residue acetyltransferase component of pyruvate dehydrogenase complex, mitochondrial OS=Mus musculus GN=Dlat PE=1 SV=2 [MASS=67941]	16	13	1.0	2.24E+10	2.68E+10	2.74E+10	2.30E+10	2.67E+10	2.55E+10	2.52E+10	2.29E+10	2.15E+10	2.36E+10	2.28E+10	2.39E+10	2.02E+10	0	0	[illegible]
O08756	HCD2_MOUSE	3-hydroxyacyl-CoA dehydrogenase type-2 OS=Mus musculus GN=Hsd17b10 PE=1 SV=4 [MASS=27418]	10	13	1.0	8.30E+09	9.58E+09	9.35E+09	8.60E+09	9.58E+09	9.65E+09	9.97E+09	8.55E+09	8.43E+09	7.48E+09	8.61E+09	7.79E+09	6.46E+09	0	0	[illegible]
Q8BK30	NDUV3_MOUSE	NADH dehydrogenase [ubiquinone] flavoprotein 3, mitochondrial OS=Mus musculus GN=Ndufv3 PE=2 SV=1 [MASS=11813]	1	5	0.99	7.25E+08	6.37E+08	6.16E+08	6.39E+08	9.60E+08	7.29E+08	8.68E+08	6.68E+08	5.01E+08	7.83E+08	4.46E+08	8.87E+08	7.37E+08	8.15E-11	8.17E-10	[illegible]
P50752	TNNT2_MOUSE	Troponin T, cardiac muscle OS=Mus musculus GN=Tnnt2 PE=2 SV=2 [MASS=35825]	6	13	1.0	5.67E+10	6.33E+10	6.56E+10	6.98E+10	6.11E+10	6.11E+10	6.30E+10	5.68E+10	5.76E+10	6.85E+10	6.38E+10	5.79E+10	6.19E+10	0	0	[illegible]
P56391	CX6B1_MOUSE	Cytochrome c oxidase subunit 6B1 OS=Mus musculus GN=Cox6b1 PE=1 SV=2 [MASS=10071]	3	12	1.0	4.04E+09	5.09E+09	2.88E+10	5.48E+09	1.13E+09	4.71E+08	2.43E+09	1.64E+09	3.08E+09	3.15E+09	4.86E+09	1.36E+10	1.03E+10	0	0	[illegible]
Q9D051	ODPB_MOUSE	Pyruvate dehydrogenase E1 component subunit beta, mitochondrial OS=Mus musculus GN=Pdhb PE=1 SV=1 [MASS=38937]	16	13	1.0	3.92E+10	4.63E+10	4.43E+10	4.08E+10	4.75E+10	4.94E+10	4.85E+10	4.25E+10	4.09E+10	4.32E+10	4.29E+10	4.10E+10	3.92E+10	0	0	[illegible]
P09542	MYL3_MOUSE	Myosin light chain 3 OS=Mus musculus GN=Myl3 PE=2 SV=4 [MASS=22421]	9	13	1.0	2.43E+11	2.45E+11	2.38E+11	2.04E+11	2.31E+11	2.24E+11	2.36E+11	2.24E+11	2.36E+11	2.42E+11	2.33E+11	2.49E+11	2.08E+11	0	0	[illegible]
Q60597	ODO1_MOUSE	2-oxoglutarate dehydrogenase E1 component, mitochondrial OS=Mus musculus GN=Ogdh PE=1 SV=3 [MASS=116448]	34	13	1.0	6.00E+10	6.71E+10	7.01E+10	5.98E+10	5.46E+10	6.52E+10	6.47E+10	6.15E+10	6.23E+10	6.39E+10	6.70E+10	6.71E+10	6.15E+10	0	0	[illegible]
P10605	CATB_MOUSE	Cathepsin B OS=Mus musculus GN=Ctsb PE=1 SV=2 [MASS=37280]	2	12	1.0	3.83E+08	4.53E+08	3.98E+08	5.03E+08	4.16E+08	5.04E+08	5.26E+08	4.74E+08	4.70E+08	5.16E+08	5.48E+08	4.92E+08	4.97E+08	3.47E-05	5.71E-06	[illegible]
Q9DB77	QCR2_MOUSE	Cytochrome b-c1 complex subunit 2, mitochondrial OS=Mus musculus GN=Uqcrc2 PE=1 SV=1 [MASS=48235]	15	13	1.0	8.65E+10	9.66E+10	9.05E+10	9.59E+10	9.04E+10	8.63E+10	9.08E+10	8.26E+10	7.64E+10	8.59E+10	8.18E+10	8.61E+10	7.56E+10	0	0	[illegible]
Q8BGH2	SAM50_MOUSE	Sorting and assembly machinery component 50 homolog OS=Mus musculus GN=Samm50 PE=1 SV=1 [MASS=51864]	12	13	1.0	4.54E+09	5.20E+09	4.70E+09	4.90E+09	4.25E+09	4.69E+09	4.53E+09	4.62E+09	4.01E+09	4.55E+09	4.26E+09	3.90E+09	3.97E+09	0	0	[illegible]
P62082	RS7_MOUSE	40S ribosomal protein S7 OS=Mus musculus GN=Rps7 PE=2 SV=1 [MASS=22127]	2	13	1.0	3.93E+08	5.89E+08	5.15E+08	4.19E+08	6.37E+08	6.23E+08	5.11E+08	7.61E+08	6.02E+08	6.04E+08	5.76E+08	5.16E+08	4.86E+08	1.63E-05	2.90E-08	[illegible]
P14824	ANXA6_MOUSE	Annexin A6 OS=Mus musculus GN=Anxa6 PE=1 SV=2 [MASS=75886]	20	13	1.0	7.93E+09	9.68E+09	9.03E+09	7.92E+09	7.86E+09	8.20E+09	7.39E+09	8.60E+09	7.81E+09	7.83E+09	7.84E+09	6.91E+09	6.43E+09	0	0	[illegible]
P27773	PDIA3_MOUSE	Protein disulfide-isomerase A3 OS=Mus musculus GN=Pdia3 PE=1 SV=2 [MASS=56678]	7	13	1.0	1.46E+09	1.54E+09	1.50E+09	1.51E+09	1.70E+09	1.55E+09	1.48E+09	1.80E+09	1.82E+09	1.76E+09	1.63E+09	1.41E+09	1.44E+09	1.19E-23	3.83E-26	[illegible]
Q91WD5	NDUS2_MOUSE	NADH dehydrogenase [ubiquinone] iron-sulfur protein 2, mitochondrial OS=Mus musculus GN=Ndufs2 PE=1 SV=1 [MASS=52625]	15	13	1.0	1.97E+10	2.17E+10	2.23E+10	2.06E+10	1.94E+10	2.07E+10	1.95E+10	1.92E+10	1.84E+10	2.03E+10	1.84E+10	1.87E+10	1.83E+10	0	0	[illegible]
P41216	ACSL1_MOUSE	Long-chain-fatty-acid--CoA ligase 1 OS=Mus musculus GN=Acsl1 PE=1 SV=1 [MASS=77923]	24	13	1.0	2.43E+10	3.09E+10	3.21E+10	2.63E+10	2.62E+10	2.86E+10	2.53E+10	2.76E+10	2.39E+10	2.54E+10	2.66E+10	2.61E+10	2.11E+10	0	0	[illegible]
Q9CZ13	QCR1_MOUSE	Cytochrome b-c1 complex subunit 1, mitochondrial OS=Mus musculus GN=Uqcrc1 PE=1 SV=2 [MASS=52768]	16	13	1.0	4.71E+10	5.47E+10	7.08E+10	5.33E+10	5.30E+10	5.17E+10	5.13E+10	4.57E+10	4.85E+10	5.07E+10	4.96E+10	5.86E+10	5.36E+10	0	0	[illegible]
O35658	C1QBP_MOUSE	Complement component 1 Q subcomponent-binding protein, mitochondrial OS=Mus musculus GN=C1qbp PE=1 SV=1 [MASS=31013]	5	12	1.0	1.15E+09	1.37E+09	1.33E+09	8.66E+08	6.81E+08	7.86E+08	1.46E+09	9.83E+08	1.22E+09	9.31E+08	1.87E+09	1.14E+09	8.93E+08	2.12E-18	9.05E-23	[illegible]
P16125	LDHB_MOUSE	L-lactate dehydrogenase B chain OS=Mus musculus GN=Ldhb PE=1 SV=2 [MASS=36572]	14	13	1.0	4.53E+10	5.16E+10	5.17E+10	4.61E+10	5.04E+10	4.96E+10	4.63E+10	4.43E+10	4.17E+10	4.23E+10	3.99E+10	4.77E+10	3.68E+10	0	0	[illegible]
Q8JZQ2	AFG32_MOUSE	AFG3-like protein 2 OS=Mus musculus GN=Afg3l2 PE=1 SV=1 [MASS=89518]	1	9	1.0	1.15E+08	1.07E+08	1.55E+08	1.01E+08	1.32E+08	1.29E+08	1.16E+08	8.89E+07	9.45E+07	1.03E+08	1.09E+08	1.07E+08	1.02E+08	0.002	2.08E-01	[illegible]
P01942	HBA_MOUSE	Hemoglobin subunit alpha OS=Mus musculus GN=Hba PE=1 SV=2 [MASS=15085]	5	13	1.0	9.01E+10	4.37E+10	5.56E+10	4.97E+10	6.31E+10	4.82E+10	6.68E+10	4.50E+10	5.80E+10	6.51E+10	4.39E+10	3.50E+10	6.67E+10	0	0	[illegible]
P07724	ALBU_MOUSE	Serum albumin OS=Mus musculus GN=Alb PE=1 SV=3 [MASS=68692]	21	13	1.0	8.06E+10	6.25E+10	1.13E+11	8.68E+10	9.92E+10	9.68E+10	9.38E+10	5.88E+10	1.03E+11	7.52E+10	7.91E+10	1.07E+11	9.31E+10	0	0	[illegible]
P04247	MYG_MOUSE	Myoglobin OS=Mus musculus GN=Mb PE=1 SV=3 [MASS=17070]	6	13	1.0	1.32E+11	1.39E+11	1.50E+11	1.36E+11	1.30E+11	1.56E+11	1.58E+11	1.21E+11	1.39E+11	1.43E+11	1.35E+11	1.46E+11	1.52E+11	0	0	[illegible]
P68134	ACTS_MOUSE	Actin, alpha skeletal muscle OS=Mus musculus GN=Acta1 PE=1 SV=1 [MASS=42051]	1	13	1.0	1.47E+09	2.06E+09	1.84E+09	1.37E+09	1.50E+09	2.13E+09	1.91E+09	1.89E+09	3.46E+09	3.41E+09	4.24E+09	4.36E+09	9.73E+09	1.06E-22	1.64E-30	[illegible]
Q9ERS2	NDUAD_MOUSE	NADH dehydrogenase [ubiquinone] 1 alpha subcomplex subunit 13 OS=Mus musculus GN=Ndufa13 PE=1 SV=3 [MASS=16859]	4	13	1.0	7.92E+09	1.02E+10	9.16E+09	7.85E+09	9.14E+09	9.89E+09	9.93E+09	9.19E+09	8.05E+09	8.82E+09	8.23E+09	8.22E+09	7.41E+09	0	0	[illegible]
P62897	CYC_MOUSE	Cytochrome c, somatic OS=Mus musculus GN=Cycs PE=1 SV=2 [MASS=11605]	5	13	1.0	6.48E+10	5.88E+10	7.09E+10	7.20E+10	6.66E+10	6.03E+10	7.36E+10	6.10E+10	5.98E+10	6.03E+10	6.08E+10	5.84E+10	5.66E+10	0	0	[illegible]
Q60936	ADCK3_MOUSE	Chaperone activity of bc1 complex-like, mitochondrial OS=Mus musculus GN=Cabc1 PE=2 SV=2 [MASS=71743]	6	13	1.0	2.04E+09	2.14E+09	2.09E+09	2.32E+09	2.32E+09	2.58E+09	2.77E+09	1.79E+09	1.91E+09	2.11E+09	2.19E+09	2.28E+09	1.73E+09	8.64E-33	3.11E-36	[illegible]
Q64475	H2B1P_MOUSE	Histone H2B type 1-B OS=Mus musculus GN=Hist1h2bb PE=1 SV=3 [MASS=13952]	1	12	1.0	4.30E+09	3.61E+09	3.49E+09	4.01E+09	3.56E+09	2.40E+09	3.25E+09	2.97E+09	3.80E+09	3.39E+09	2.86E+09	3.25E+09	2.51E+09	0	0	[illegible]
Q9Z2I9	SUCB1_MOUSE	Succinyl-CoA ligase [ADP-forming] subunit beta, mitochondrial OS=Mus musculus GN=Sucla2 PE=1 SV=2 [MASS=50114]	17	13	1.0	2.76E+10	2.84E+10	2.80E+10	2.76E+10	2.71E+10	2.68E+10	2.78E+10	2.47E+10	2.20E+10	2.54E+10	2.26E+10	2.70E+10	2.23E+10	0	0	[illegible]
P05202	AATM_MOUSE	Aspartate aminotransferase, mitochondrial OS=Mus musculus GN=Got2 PE=1 SV=1 [MASS=47411]	18	13	1.0	6.70E+10	6.88E+10	7.95E+10	7.23E+10	6.36E+10	6.93E+10	7.02E+10	5.96E+10	6.48E+10	6.86E+10	6.19E+10	7.96E+10	7.13E+10	0	0	[illegible]
Q8BWT1	THIM_MOUSE	3-ketoacyl-CoA thiolase, mitochondrial OS=Mus musculus GN=Acaa2 PE=1 SV=2 [MASS=41858]	21	13	1.0	1.44E+11	1.50E+11	1.58E+11	1.55E+11	1.72E+11	1.66E+11	1.57E+11	1.40E+11	1.36E+11	1.36E+11	1.40E+11	1.34E+11	1.16E+11	0	0	[illegible]
P61089	UBE2N_MOUSE	Ubiquitin-conjugating enzyme E2 N OS=Mus musculus GN=Ube2n PE=1 SV=1 [MASS=17138]	3	13	1.0	7.35E+08	8.75E+08	7.58E+08	6.19E+08	5.19E+08	9.26E+08	7.01E+08	8.23E+08	8.20E+08	7.85E+08	7.60E+08	6.98E+08	6.17E+08	3.38E-11	1.72E-12	[illegible]
O54724	PTRF_MOUSE	Polymerase I and transcript release factor OS=Mus musculus GN=Ptrf PE=1 SV=1 [MASS=43953]	7	13	1.0	8.97E+09	8.36E+09	7.71E+09	8.20E+09	7.72E+09	7.41E+09	7.69E+09	7.41E+09	7.74E+09	8.26E+09	8.01E+09	8.29E+09	6.83E+09	0	0	[illegible]
Q9CQQ7	AT5F1_MOUSE	ATP synthase subunit b, mitochondrial OS=Mus musculus GN=Atp5f1 PE=1 SV=1 [MASS=28949]	8	13	1.0	3.36E+10	3.74E+10	3.27E+10	3.66E+10	3.32E+10	3.49E+10	3.63E+10	3.84E+10	3.35E+10	3.41E+10	3.42E+10	3.51E+10	2.85E+10	0	0	[illegible]
P24270	CATA_MOUSE	Catalase OS=Mus musculus GN=Cat PE=1 SV=3 [MASS=59765]	9	13	1.0	1.80E+09	1.32E+09	1.17E+09	1.23E+09	1.40E+09	1.28E+09	1.59E+09	1.38E+09	1.19E+09	1.42E+09	1.24E+09	1.29E+09	1.15E+09	3.01E-29	9.79E-20	[illegible]
Q07417	ACADS_MOUSE	Short-chain specific acyl-CoA dehydrogenase, mitochondrial OS=Mus musculus GN=Acads PE=2 SV=1 [MASS=44946]	13	13	1.0	1.36E+10	1.49E+10	1.57E+10	1.53E+10	1.61E+10	1.45E+10	1.58E+10	1.29E+10	1.24E+10	1.33E+10	1.33E+10	1.28E+10	1.22E+10	0	0	[illegible]
Q9CR68	UCRI_MOUSE	Cytochrome b-c1 complex subunit Rieske, mitochondrial OS=Mus musculus GN=Uqcrfs1 PE=1 SV=1 [MASS=29367]	8	13	1.0	1.56E+10	1.65E+10	1.66E+10	1.55E+10	1.78E+10	1.79E+10	1.77E+10	1.56E+10	1.54E+10	1.70E+10	1.59E+10	1.71E+10	1.59E+10	0	0	[illegible]
Q8BFR5	EFTU_MOUSE	Elongation factor Tu, mitochondrial OS=Mus musculus GN=Tufm PE=1 SV=1 [MASS=49508]	12	13	1.0	1.34E+10	1.58E+10	1.51E+10	1.58E+10	1.60E+10	1.49E+10	1.53E+10	1.44E+10	1.35E+10	1.37E+10	1.44E+10	1.33E+10	1.36E+10	0	0	[illegible]
Q9D8B4	NDUAB_MOUSE	NADH dehydrogenase [ubiquinone] 1 alpha subcomplex subunit 11 OS=Mus musculus GN=Ndufa11 PE=2 SV=2 [MASS=14982]	3	13	1.0	1.07E+09	2.22E+09	2.66E+09	1.15E+09	1.06E+09	1.28E+09	1.20E+09	1.50E+09	1.04E+09	1.31E+09	1.39E+09	1.35E+09	1.13E+09	3.90E-16	0	[illegible]
Q08857	CD36_MOUSE	Platelet glycoprotein 4 OS=Mus musculus GN=Cd36 PE=2 SV=2 [MASS=52698]	8	13	1.0	7.01E+09	8.25E+09	7.83E+09	6.96E+09	7.62E+09	7.16E+09	7.42E+09	7.37E+09	6.17E+09	6.96E+09	6.55E+09	6.85E+09	7.45E+09	0	0	[illegible]
Q9CR62	M2OM_MOUSE	Mitochondrial 2-oxoglutarate/malate carrier protein OS=Mus musculus GN=Slc25a11 PE=1 SV=3 [MASS=34155]	12	13	1.0	1.23E+10	1.78E+10	1.60E+10	1.54E+10	1.39E+10	1.72E+10	1.54E+10	1.58E+10	1.20E+10	1.43E+10	1.45E+10	1.43E+10	1.47E+10	0	0	[illegible]
Q91X78	ERLN1_MOUSE	Erlin-1 OS=Mus musculus GN=Erlin1 PE=2 SV=1 [MASS=38936]	1	4	0.99	3.78E+07	3.09E+07	3.68E+07	4.25E+07	3.36E+07	3.55E+07	3.99E+07	3.61E+07	2.54E+07	5.30E+07	2.75E+07	4.14E+07	4.61E+07	0.042	0.039	[illegible]
P67778	PHB_MOUSE	Prohibitin OS=Mus musculus GN=Phb PE=1 SV=1 [MASS=29820]	9	13	1.0	6.36E+09	7.03E+09	6.94E+09	5.84E+09	7.27E+09	6.96E+09	6.52E+09	6.75E+09	6.43E+09	6.62E+09	5.99E+09	6.35E+09	5.80E+09	0	0	[illegible]
P51667	MLRV_MOUSE	Myosin regulatory light chain 2, ventricular/cardiac muscle isoform OS=Mus musculus GN=Myl2 PE=2 SV=3 [MASS=18864]	8	10	1.0	6.68E+10	8.44E+10	7.24E+10	7.00E+10	9.92E+10	8.45E+10	8.61E+10	7.62E+10	7.35E+10	8.83E+10	7.27E+10	8.28E+10	7.98E+10	0	0	[illegible]
Q6P8J7	KCRS_MOUSE	Creatine kinase S-type, mitochondrial OS=Mus musculus GN=Ckmt2 PE=1 SV=1 [MASS=47473]	15	13	1.0	5.35E+10	7.58E+10	9.71E+10	6.14E+10	6.12E+10	7.39E+10	6.80E+10	6.09E+10	5.90E+10	6.90E+10	6.25E+10	8.12E+10	7.92E+10	0	0	[illegible]
Q61941	NNTM_MOUSE	NAD(P) transhydrogenase, mitochondrial OS=Mus musculus GN=Nnt PE=1 SV=2 [MASS=113838]	26	13	1.0	4.37E+10	5.25E+10	4.91E+10	5.06E+10	5.14E+10	5.40E+10	5.55E+10	4.41E+10	4.13E+10	4.42E+10	4.61E+10	4.99E+10	4.86E+10	0	0	[illegible]
Q1XH17	TRI72_MOUSE	Tripartite motif-containing protein 72 OS=Mus musculus GN=Trim72 PE=1 SV=1 [MASS=52816]	11	13	1.0	5.02E+09	6.98E+09	5.78E+09	4.57E+09	4.60E+09	5.56E+09	5.10E+09	6.92E+09	6.22E+09	6.08E+09	7.05E+09	5.11E+09	5.40E+09	0	0	[illegible]
P62242	RS8_MOUSE	40S ribosomal protein S8 OS=Mus musculus GN=Rps8 PE=1 SV=2 [MASS=24205]	2	8	1.0	1.71E+08	1.38E+08	1.99E+08	1.97E+08	2.03E+08	1.50E+08	1.80E+08	1.74E+08	2.04E+08	1.85E+08	1.98E+08	1.79E+08	1.93E+08	1.43E-01	2.95E-02	[illegible]
Q9Z2I0	LETM1_MOUSE	LETM1 and EF-hand domain-containing protein 1, mitochondrial OS=Mus musculus GN=Letm1 PE=2 SV=1 [MASS=82988]	7	12	1.0	8.63E+08	1.04E+09	1.07E+09	8.20E+08	9.12E+08	1.02E+09	9.23E+08	1.07E+09	8.25E+08	9.46E+08	9.24E+08	9.79E+08	8.58E+08	2.13E-13	3.09E-18	[illegible]
Q9CQH3	NDUB5_MOUSE	NADH dehydrogenase [ubiquinone] 1 beta subcomplex subunit 5, mitochondrial OS=Mus musculus GN=Ndufb5 PE=1 SV=1 [MASS=21710]	3	13	1.0	1.55E+09	2.71E+09	1.67E+09	1.68E+09	1.31E+09	1.73E+09	1.40E+09	2.69E+09	1.29E+09	1.82E+09	1.48E+09	1.63E+09	1.21E+09	1.46E-24	1.88E-28	[illegible]
Q9WTR5	CAD13_MOUSE	Cadherin-13 OS=Mus musculus GN=Cdh13 PE=2 SV=1 [MASS=78285]	2	13	1.0	7.39E+08	6.91E+08	6.31E+08	6.23E+08	8.27E+08	7.07E+08	6.71E+08	6.58E+08	5.74E+08	6.26E+08	5.90E+08	6.44E+08	5.22E+08	4.50E-11	4.78E-10	[illegible]
Q9CRB9	CHCH3_MOUSE	Coiled-coil-helix-coiled-coil-helix domain-containing protein 3, mitochondrial OS=Mus musculus GN=Chchd3 PE=1 SV=1 [MASS=26334]	2	13	1.0	2.51E+09	2.53E+09	2.23E+09	2.85E+09	3.08E+09	2.12E+09	2.75E+09	2.14E+09	2.42E+09	2.45E+09	2.21E+09	2.64E+09	2.13E+09	5.80E-37	5.23E-35	[illegible]
Q9CQ75	NDUA2_MOUSE	NADH dehydrogenase [ubiquinone] 1 alpha subcomplex subunit 2 OS=Mus musculus GN=Ndufa2 PE=1 SV=3 [MASS=10916]	3	13	1.0	5.91E+09	5.35E+09	6.28E+09	7.41E+09	7.30E+09	5.75E+09	6.63E+09	5.47E+09	5.47E+09	5.88E+09	5.39E+09	5.34E+09	5.98E+09	0	0	[illegible]
Q791V5	MTCH2_MOUSE	Mitochondrial carrier homolog 2 OS=Mus musculus GN=Mtch2 PE=1 SV=1 [MASS=33499]	5	13	1.0	2.28E+09	2.19E+09	2.31E+09	2.36E+09	2.16E+09	1.97E+09	2.07E+09	2.16E+09	1.81E+09	1.91E+09	2.16E+09	1.87E+09	2.15E+09	2.59E-36	1.72E-39	[illegible]
Q9QYG0	NDRG2_MOUSE	Protein NDRG2 OS=Mus musculus GN=Ndrg2 PE=1 SV=1 [MASS=40789]	11	13	1.0	7.78E+09	9.17E+09	1.19E+10	8.46E+09	9.36E+09	9.81E+09	9.00E+09	9.15E+09	9.23E+09	9.04E+09	8.37E+09	9.72E+09	8.01E+09	0	0	[illegible]
Q9D0M3	CY1_MOUSE	Cytochrome c1, heme protein, mitochondrial OS=Mus musculus GN=Cyc1 PE=1 SV=1 [MASS=35327]	10	13	1.0	2.47E+10	2.46E+10	2.78E+10	2.71E+10	2.39E+10	2.46E+10	2.47E+10	2.43E+10	2.28E+10	2.44E+10	2.20E+10	2.30E+10	2.37E+10	0	0	[illegible]
Q99LY9	NDUS5_MOUSE	NADH dehydrogenase [ubiquinone] iron-sulfur protein 5 OS=Mus musculus GN=Ndufs5 PE=1 SV=3 [MASS=12648]	3	12	1.0	1.40E+09	1.41E+09	2.76E+09	1.45E+09	4.81E+08	1.04E+09	1.17E+09	1.08E+09	1.37E+09	1.16E+09	1.41E+09	1.83E+09	1.53E+09	1.03E-20	0	[illegible]
Q924X2	CPT1B_MOUSE	Carnitine O-palmitoyltransferase 1, muscle isoform OS=Mus musculus GN=Cpt1b PE=2 SV=1 [MASS=88217]	14	13	1.0	1.15E+10	1.37E+10	1.36E+10	1.21E+10	9.75E+09	1.23E+10	1.14E+10	1.24E+10	1.14E+10	1.12E+10	1.24E+10	1.29E+10	1.11E+10	0	0	[illegible]
Q8QZT1	THIL_MOUSE	Acetyl-CoA acetyltransferase, mitochondrial OS=Mus musculus GN=Acat1 PE=1 SV=1 [MASS=44816]	13	13	1.0	4.28E+10	3.95E+10	4.18E+10	4.14E+10	4.88E+10	4.58E+10	4.47E+10	3.89E+10	3.85E+10	3.90E+10	3.89E+10	4.09E+10	3.91E+10	0	0	[illegible]
Q80XN0	BDH_MOUSE	D-beta-hydroxybutyrate dehydrogenase, mitochondrial OS=Mus musculus GN=Bdh1 PE=1 SV=1 [MASS=38285]	7	13	1.0	4.59E+08	1.51E+09	1.61E+09	8.96E+08	1.10E+09	1.87E+09	2.41E+09	1.08E+09	1.13E+09	1.54E+09	1.97E+09	2.58E+09	4.81E+09	1.82E-06	9.02E-28	[illegible]
P47934	CACP_MOUSE	Carnitine O-acetyltransferase OS=Mus musculus GN=Crat PE=1 SV=2 [MASS=70924]	16	13	1.0	1.19E+10	1.20E+10	1.28E+10	1.32E+10	1.31E+10	1.29E+10	1.23E+10	1.10E+10	9.78E+09	1.12E+10	1.14E+10	1.04E+10	9.19E+09	0	0	[illegible]
P09671	SODM_MOUSE	Superoxide dismutase [Mn], mitochondrial OS=Mus musculus GN=Sod2 PE=1 SV=3 [MASS=24603]	4	13	1.0	1.87E+10	2.26E+10	2.08E+10	2.24E+10	2.56E+10	2.35E+10	2.46E+10	2.21E+10	1.97E+10	2.04E+10	2.08E+10	1.73E+10	1.66E+10	0	0	[illegible]
P14869	RLA0_MOUSE	60S acidic ribosomal protein P0 OS=Mus musculus GN=Rplp0 PE=1 SV=3 [MASS=34216]	4	13	1.0	7.96E+08	1.21E+09	9.47E+08	8.80E+08	9.70E+08	9.58E+08	8.30E+08	1.21E+09	9.58E+08	1.04E+09	9.87E+08	7.37E+08	7.69E+08	3.01E-12	9.51E-16	[illegible]
Q9CQJ8	NDUB9_MOUSE	NADH dehydrogenase [ubiquinone] 1 beta subcomplex subunit 9 OS=Mus musculus GN=Ndufb9 PE=1 SV=3 [MASS=21984]	4	13	1.0	5.87E+09	5.91E+09	6.32E+09	6.33E+09	5.77E+09	6.10E+09	5.82E+09	5.58E+09	5.52E+09	5.75E+09	5.72E+09	4.61E+09	4.57E+09	0	0	[illegible]
P62858	RS28_MOUSE	40S ribosomal protein S28 OS=Mus musculus GN=Rps28 PE=2 SV=1 [MASS=7841]	2	13	1.0	4.15E+08	4.03E+08	3.69E+08	3.39E+08	4.43E+08	3.86E+08	3.77E+08	4.59E+08	4.09E+08	3.97E+08	3.84E+08	3.47E+08	3.25E+08	7.39E-06	1.57E-05	[illegible]
Q9CZU6	CISY_MOUSE	Citrate synthase, mitochondrial OS=Mus musculus GN=Cs PE=1 SV=1 [MASS=51736]	18	13	1.0	7.06E+10	8.18E+10	7.49E+10	6.56E+10	6.63E+10	7.42E+10	7.40E+10	7.75E+10	6.95E+10	6.28E+10	6.84E+10	6.53E+10	4.81E+10	0	0	[illegible]
P04117	FABP4_MOUSE	Fatty acid-binding protein, adipocyte OS=Mus musculus GN=Fabp4 PE=1 SV=3 [MASS=14650]	6	13	1.0	1.55E+10	1.45E+10	1.56E+10	1.81E+10	1.64E+10	1.47E+10	1.47E+10	1.41E+10	1.52E+10	1.61E+10	1.23E+10	1.41E+10	1.57E+10	0	0	[illegible]
P19783	COX41_MOUSE	Cytochrome c oxidase subunit 4 isoform 1, mitochondrial OS=Mus musculus GN=Cox4i1 PE=1 SV=2 [MASS=19530]	11	13	1.0	7.25E+10	8.76E+10	8.07E+10	7.65E+10	8.62E+10	8.62E+10	8.34E+10	7.35E+10	7.31E+10	8.34E+10	7.63E+10	7.64E+10	7.51E+10	0	0	[illegible]
P19536	COX5B_MOUSE	Cytochrome c oxidase subunit 5B, mitochondrial OS=Mus musculus GN=Cox5b PE=1 SV=1 [MASS=13813]	4	13	1.0	1.21E+10	1.31E+10	1.16E+10	1.35E+10	1.44E+10	1.07E+10	1.31E+10	1.10E+10	9.73E+09	1.24E+10	9.81E+09	1.38E+10	1.22E+10	0	0	[illegible]
O70250	PGAM2_MOUSE	Phosphoglycerate mutase 2 OS=Mus musculus GN=Pgam2 PE=1 SV=3 [MASS=28827]	7	13	1.0	1.07E+10	1.18E+10	1.05E+10	1.01E+10	9.59E+09	1.12E+10	1.03E+10	1.15E+10	1.18E+10	1.15E+10	1.16E+10	1.13E+10	1.02E+10	0	0	[illegible]
Q99JB8	PACN3_MOUSE	Protein kinase C and casein kinase II substrate protein 3 OS=Mus musculus GN=Pacsin3 PE=1 SV=1 [MASS=48585]	3	2	1.0	1.17E+08	1.59E+08	1.55E+08	1.58E+08	1.35E+08	1.39E+08	1.48E+08	1.45E+08	1.72E+08	1.39E+08	2.41E+08	1.61E+08	1.59E+08	0.001	1.77E-01	[illegible]
Q9DCT2	NDUS3_MOUSE	NADH dehydrogenase [ubiquinone] iron-sulfur protein 3, mitochondrial OS=Mus musculus GN=Ndufs3 PE=1 SV=2 [MASS=30149]	8	13	1.0	1.51E+10	2.00E+10	1.78E+10	1.44E+10	1.68E+10	1.82E+10	1.77E+10	1.66E+10	1.47E+10	1.66E+10	1.61E+10	1.44E+10	1.22E+10	0	0	[illegible]
P48771	CX7A2_MOUSE	Cytochrome c oxidase polypeptide 7A2, mitochondrial OS=Mus musculus GN=Cox7a2 PE=1 SV=2 [MASS=9291]	2	13	1.0	5.19E+09	4.89E+09	4.46E+09	4.45E+09	3.71E+09	3.06E+09	3.96E+09	3.99E+09	4.33E+09	4.05E+09	4.84E+09	3.45E+09	4.17E+09	0	0	[illegible]
Q91VR2	ATPG_MOUSE	ATP synthase subunit gamma, mitochondrial OS=Mus musculus GN=Atp5c1 PE=1 SV=1 [MASS=32886]	7	13	1.0	3.08E+10	3.18E+10	3.37E+10	3.87E+10	3.83E+10	2.90E+10	3.67E+10	2.93E+10	2.74E+10	3.19E+10	2.75E+10	3.42E+10	3.47E+10	0	0	[illegible]
Q9QXX4	CMC2_MOUSE	Calcium-binding mitochondrial carrier protein Aralar2 OS=Mus musculus GN=Slc25a13 PE=1 SV=1 [MASS=74466]	14	13	1.0	6.97E+09	8.64E+09	8.62E+09	7.91E+09	9.48E+09	1.00E+10	9.40E+09	8.51E+09	7.75E+09	8.01E+09	7.57E+09	7.95E+09	6.40E+09	0	0	[illegible]
P51881	ADT2_MOUSE	ADP/ATP translocase 2 OS=Mus musculus GN=Slc25a5 PE=1 SV=3 [MASS=32931]	3	12	1.0	2.98E+09	5.06E+09	3.62E+09	2.46E+09	2.88E+09	3.91E+09	3.01E+09	3.89E+09	3.21E+09	3.49E+09	3.16E+09	2.64E+09	2.69E+09	2.80E-42	0	[illegible]
P58252	EF2_MOUSE	Elongation factor 2 OS=Mus musculus GN=Eef2 PE=1 SV=2 [MASS=95313]	14	13	1.0	4.20E+09	5.72E+09	6.98E+09	4.95E+09	5.05E+09	4.80E+09	4.53E+09	5.76E+09	5.30E+09	4.86E+09	4.89E+09	5.76E+09	5.60E+09	0	0	[illegible]
Q99LB2	DHRS4_MOUSE	Dehydrogenase/reductase SDR family member 4 OS=Mus musculus GN=Dhrs4 PE=2 SV=1 [MASS=27754]	4	13	1.0	1.34E+09	1.43E+09	1.73E+09	1.52E+09	1.59E+09	1.47E+09	1.40E+09	1.17E+09	1.22E+09	1.37E+09	1.22E+09	1.72E+09	1.53E+09	4.96E-21	2.27E-29	[illegible]
Q61387	COX7R_MOUSE	Cytochrome c oxidase subunit 7A-related protein, mitochondrial OS=Mus musculus GN=Cox7a2l PE=2 SV=1 [MASS=12399]	1	3	0.99	1.18E+08	9.24E+07	9.31E+07	1.22E+08	1.11E+08	1.69E+08	1.08E+08	8.48E+07	1.11E+08	1.08E+08	9.20E+07	1.23E+08	1.19E+08	0.011	0.010	[illegible]
P38647	GRP75_MOUSE	Stress-70 protein, mitochondrial OS=Mus musculus GN=Hspa9 PE=1 SV=2 [MASS=73528]	20	13	1.0	2.08E+10	2.24E+10	2.17E+10	2.00E+10	2.17E+10	2.14E+10	1.99E+10	2.06E+10	1.88E+10	2.10E+10	2.08E+10	1.96E+10	1.96E+10	0	0	[illegible]
Q7TMF3	NDUAC_MOUSE	NADH dehydrogenase [ubiquinone] 1 alpha subcomplex subunit 12 OS=Mus musculus GN=Ndufa12 PE=1 SV=2 [MASS=17086]	6	13	1.0	6.39E+09	7.46E+09	7.77E+09	6.14E+09	5.23E+09	6.71E+09	6.53E+09	6.53E+09	5.84E+09	6.15E+09	6.43E+09	6.50E+09	6.25E+09	0	0	[illegible]
Q9DCS9	NDUBA_MOUSE	NADH dehydrogenase [ubiquinone] 1 beta subcomplex subunit 10 OS=Mus musculus GN=Ndufb10 PE=1 SV=3 [MASS=21024]	5	13	1.0	1.03E+10	1.20E+10	1.22E+10	1.04E+10	1.25E+10	1.16E+10	1.11E+10	1.09E+10	1.07E+10	1.12E+10	1.08E+10	9.63E+09	8.71E+09	0	0	[illegible]
Q8VCW8	ACSF2_MOUSE	Acyl-CoA synthetase family member 2, mitochondrial OS=Mus musculus GN=Acsf2 PE=2 SV=1 [MASS=67950]	3	10	1.0	2.35E+08	1.97E+08	2.91E+08	2.69E+08	3.73E+08	3.79E+08	2.80E+08	3.03E+08	3.29E+08	2.90E+08	3.34E+08	3.59E+08	4.02E+08	8.90E-03	4.42E-04	[illegible]
P14094	AT1B1_MOUSE	Sodium/potassium-transporting ATPase subunit beta-1 OS=Mus musculus GN=Atp1b1 PE=1 SV=1 [MASS=35194]	6	13	1.0	4.68E+09	5.14E+09	5.00E+09	4.50E+09	4.39E+09	4.45E+09	5.00E+09	4.80E+09	4.59E+09	4.78E+09	4.77E+09	5.30E+09	4.27E+09	0	0	[illegible]
O09161	CASQ2_MOUSE	Calsequestrin-2 OS=Mus musculus GN=Casq2 PE=2 SV=2 [MASS=48197]	8	13	1.0	2.16E+10	2.46E+10	2.54E+10	2.37E+10	2.45E+10	2.52E+10	2.57E+10	2.34E+10	2.32E+10	2.40E+10	2.52E+10	2.14E+10	1.85E+10	0	0	[illegible]
Q61207	SAP_MOUSE	Sulfated glycoprotein 1 OS=Mus musculus GN=Psap PE=1 SV=2 [MASS=61422]	1	13	1.0	2.41E+08	2.11E+08	4.86E+08	2.86E+08	3.98E+08	1.74E+08	3.14E+08	2.12E+08	3.73E+08	2.65E+08	3.15E+08	4.60E+08	3.39E+08	8.02E-03	1.08E-07	[illegible]
Q8R404	QIL1_MOUSE	Protein QIL1 OS=Mus musculus GN=Qil1 PE=2 SV=1 [MASS=13373]	2	10	1.0	8.69E+08	1.28E+09	1.36E+09	1.25E+09	1.59E+09	1.40E+09	1.40E+09	1.24E+09	1.25E+09	1.35E+09	9.27E+08	1.25E+09	1.16E+09	1.83E-13	7.15E-23	[illegible]
P17751	TPIS_MOUSE	Triosephosphate isomerase OS=Mus musculus GN=Tpi1 PE=1 SV=3 [MASS=26712]	9	13	1.0	1.92E+10	2.46E+10	2.64E+10	2.15E+10	2.47E+10	2.50E+10	2.36E+10	2.14E+10	2.30E+10	2.33E+10	2.32E+10	2.44E+10	2.26E+10	0	0	[illegible]
Q8BKZ9	ODPX_MOUSE	Pyruvate dehydrogenase protein X component, mitochondrial OS=Mus musculus GN=Pdhx PE=2 SV=1 [MASS=53999]	8	13	1.0	3.48E+09	3.67E+09	3.58E+09	3.48E+09	3.79E+09	3.91E+09	3.35E+09	3.71E+09	3.36E+09	3.58E+09	3.75E+09	3.63E+09	3.35E+09	0	0	[illegible]
P80317	TCPZ_MOUSE	T-complex protein 1 subunit zeta OS=Mus musculus GN=Cct6a PE=1 SV=3 [MASS=58004]	2	4	1.0	1.38E+08	1.82E+08	1.82E+08	1.54E+08	2.40E+08	1.82E+08	1.87E+08	2.09E+08	1.78E+08	2.16E+08	1.44E+08	1.57E+08	1.76E+08	5.53E-01	6.33E-02	[illegible]
P62751	RL23A_MOUSE	60S ribosomal protein L23a OS=Mus musculus GN=Rpl23a PE=2 SV=1 [MASS=17695]	1	11	0.71	1.32E+09	1.17E+09	1.55E+09	1.35E+09	1.88E+09	1.83E+09	1.52E+09	1.06E+09	1.85E+09	1.31E+09	1.36E+09	1.06E+09	1.02E+09	4.88E-20	4.08E-25	[illegible]
Q9CPQ1	COX6C_MOUSE	Cytochrome c oxidase subunit 6C OS=Mus musculus GN=Cox6c PE=1 SV=3 [MASS=8469]	2	13	1.0	8.41E+09	7.13E+09	5.85E+09	7.31E+09	6.50E+09	6.19E+09	8.16E+09	6.37E+09	5.71E+09	6.74E+09	4.81E+09	8.36E+09	8.16E+09	0	0	[illegible]
P51174	ACADL_MOUSE	Long-chain specific acyl-CoA dehydrogenase, mitochondrial OS=Mus musculus GN=Acadl PE=2 SV=2 [MASS=47908]	13	13	1.0	5.62E+10	6.66E+10	7.05E+10	6.69E+10	6.17E+10	6.67E+10	6.35E+10	5.83E+10	5.45E+10	6.12E+10	6.59E+10	6.33E+10	5.86E+10	0	0	[illegible]
P08249	MDHM_MOUSE	Malate dehydrogenase, mitochondrial OS=Mus musculus GN=Mdh2 PE=1 SV=3 [MASS=35611]	17	13	1.0	1.09E+11	1.40E+11	1.58E+11	1.06E+11	1.20E+11	1.24E+11	1.26E+11	1.16E+11	1.10E+11	1.14E+11	1.15E+11	1.33E+11	1.18E+11	0	0	[illegible]
P26041	MOES_MOUSE	Moesin OS=Mus musculus GN=Msn PE=1 SV=3 [MASS=67767]	10	13	1.0	2.32E+09	2.28E+09	2.39E+09	2.06E+09	2.51E+09	2.22E+09	2.02E+09	2.59E+09	2.76E+09	2.54E+09	2.16E+09	2.58E+09	2.59E+09	6.23E-38	7.01E-42	[illegible]
P11499	HS90B_MOUSE	Heat shock protein HSP 90-beta OS=Mus musculus GN=Hsp90ab1 PE=1 SV=2 [MASS=83325]	11	13	1.0	9.12E+09	9.11E+09	1.09E+10	9.74E+09	8.48E+09	8.33E+09	8.43E+09	9.33E+09	8.98E+09	9.78E+09	8.81E+09	9.45E+09	9.06E+09	0	0	[illegible]
Q9Z2Z6	MCAT_MOUSE	Mitochondrial carnitine/acylcarnitine carrier protein OS=Mus musculus GN=Slc25a20 PE=1 SV=1 [MASS=33027]	4	13	1.0	1.39E+09	2.13E+09	2.51E+09	1.59E+09	2.18E+09	2.14E+09	2.08E+09	2.04E+09	1.79E+09	1.83E+09	1.87E+09	2.22E+09	1.77E+09	1.97E-22	0	[illegible]
P11404	FABPH_MOUSE	Fatty acid-binding protein, heart OS=Mus musculus GN=Fabp3 PE=1 SV=5 [MASS=14819]	6	13	1.0	5.43E+10	4.59E+10	4.95E+10	6.38E+10	6.07E+10	4.54E+10	6.24E+10	3.43E+10	4.82E+10	5.69E+10	4.29E+10	5.89E+10	6.70E+10	0	0	[illegible]
Q9CQ62	DECR_MOUSE	2,4-dienoyl-CoA reductase, mitochondrial OS=Mus musculus GN=Decr1 PE=1 SV=1 [MASS=36214]	8	13	1.0	2.09E+10	2.23E+10	2.39E+10	2.19E+10	2.62E+10	2.34E+10	2.44E+10	1.97E+10	1.96E+10	2.13E+10	2.09E+10	2.15E+10	2.00E+10	0	0	[illegible]
Q78IK4	APOOL_MOUSE	Apolipoprotein O-like OS=Mus musculus GN=Apool PE=2 SV=1 [MASS=29261]	6	13	1.0	1.55E+09	2.02E+09	1.84E+09	1.66E+09	2.09E+09	1.78E+09	1.88E+09	1.73E+09	1.54E+09	1.72E+09	1.59E+09	1.72E+09	1.61E+09	3.37E-25	3.78E-32	[illegible]
P05064	ALDOA_MOUSE	Fructose-bisphosphate aldolase A OS=Mus musculus GN=Aldoa PE=1 SV=2 [MASS=39356]	12	13	1.0	3.88E+10	4.52E+10	4.19E+10	4.07E+10	4.39E+10	4.45E+10	4.16E+10	3.81E+10	4.01E+10	4.40E+10	4.11E+10	4.20E+10	4.20E+10	0	0	[illegible]
P49817	CAV1_MOUSE	Caveolin-1 OS=Mus musculus GN=Cav1 PE=1 SV=1 [MASS=20539]	3	13	1.0	2.60E+09	3.35E+09	2.90E+09	3.12E+09	2.46E+09	2.64E+09	3.17E+09	2.81E+09	3.03E+09	3.25E+09	3.41E+09	3.44E+09	2.68E+09	2.51E-39	0	[illegible]
P52503	NDUS6_MOUSE	NADH dehydrogenase [ubiquinone] iron-sulfur protein 6, mitochondrial OS=Mus musculus GN=Ndufs6 PE=1 SV=2 [MASS=13020]	4	13	1.0	7.96E+09	9.13E+09	8.27E+09	7.13E+09	6.91E+09	8.16E+09	8.25E+09	7.85E+09	6.35E+09	8.01E+09	7.53E+09	7.41E+09	6.88E+09	0	0	[illegible]
Q99NB1	ACS2L_MOUSE	Acetyl-coenzyme A synthetase 2-like, mitochondrial OS=Mus musculus GN=Acss1 PE=1 SV=1 [MASS=74623]	11	13	1.0	7.20E+09	7.55E+09	7.37E+09	7.62E+09	7.88E+09	7.29E+09	7.11E+09	6.81E+09	6.03E+09	6.39E+09	5.93E+09	6.05E+09	5.81E+09	0	0	[illegible]
P62631	EF1A2_MOUSE	Elongation factor 1-alpha 2 OS=Mus musculus GN=Eef1a2 PE=1 SV=1 [MASS=50454]	8	13	1.0	9.95E+09	1.28E+10	1.31E+10	8.20E+09	6.69E+09	1.11E+10	1.06E+10	1.18E+10	1.11E+10	9.67E+09	1.15E+10	9.30E+09	7.83E+09	0	0	[illegible]
Q61425	HCDH_MOUSE	Hydroxyacyl-coenzyme A dehydrogenase, mitochondrial OS=Mus musculus GN=Hadh PE=1 SV=2 [MASS=34464]	12	13	1.0	2.28E+10	2.70E+10	3.52E+10	2.65E+10	2.94E+10	2.93E+10	3.02E+10	2.66E+10	2.67E+10	2.42E+10	3.04E+10	2.63E+10	2.16E+10	0	0	[illegible]
Q9CPP6	NDUA5_MOUSE	NADH dehydrogenase [ubiquinone] 1 alpha subcomplex subunit 5 OS=Mus musculus GN=Ndufa5 PE=1 SV=3 [MASS=13360]	2	13	1.0	1.71E+09	1.77E+09	1.70E+09	2.35E+09	2.59E+09	1.92E+09	2.23E+09	1.65E+09	1.55E+09	2.06E+09	1.55E+09	2.41E+09	2.44E+09	1.56E-24	2.41E-26	[illegible]
P18572	BASI_MOUSE	Basigin OS=Mus musculus GN=Bsg PE=1 SV=2 [MASS=42444]	4	13	1.0	2.33E+09	2.32E+09	2.41E+09	2.72E+09	3.12E+09	2.34E+09	2.72E+09	2.29E+09	2.55E+09	2.85E+09	2.22E+09	2.99E+09	2.66E+09	1.12E-24	1.09E-39	[illegible]
P14131	RS16_MOUSE	40S ribosomal protein S16 OS=Mus musculus GN=Rps16 PE=2 SV=4 [MASS=16445]	1	6	0.75	2.72E+08	1.68E+08	1.57E+08	2.17E+08	1.98E+08	1.38E+08	1.99E+08	1.69E+08	2.14E+08	1.97E+08	1.52E+08	2.30E+08	2.14E+08	2.44E-03	1.49E-01	[illegible]
P97450	ATP5J_MOUSE	ATP synthase-coupling factor 6, mitochondrial OS=Mus musculus GN=Atp5j PE=1 SV=1 [MASS=12496]	4	13	1.0	1.43E+10	1.42E+10	1.41E+10	1.34E+10	1.59E+10	1.32E+10	1.47E+10	1.19E+10	1.25E+10	1.31E+10	1.23E+10	1.25E+10	1.11E+10	0	0	[illegible]
Q922B1	MACD1_MOUSE	MACRO domain-containing protein 1 OS=Mus musculus GN=Macrod1 PE=2 SV=2 [MASS=35294]	6	13	1.0	3.09E+09	3.45E+09	4.60E+09	3.42E+09	3.16E+09	3.51E+09	3.57E+09	2.68E+09	3.02E+09	2.96E+09	3.36E+09	3.87E+09	3.57E+09	0	0	[illegible]

P00405	COX2_MOUSE	Cytochrome c oxidase subunit 2 OS=Mus musculus GN=Mtco2 PE=1 SV=1 [MASS=25976]	8	13	1.0	4.10E+10	7.02E+10	5.41E+10	3.96E+10	5.32E+10	6.86E+10	5.25E+10	6.04E+10	5.04E+10	5.08E+10	5.41E+10	4.36E+10	4.00E+10	0	0	0	0	0	0	0	0	0	0	0	0	0	2, 4, 6, 2, 5, 3, 4, 5, 6, 6, 8	1, 12, 13, 3, 4	13, 13, 21, 17, 21, 21, 4, 7	COX2_MOUSE, SP:P00405	SP|P00405|COX2_MOUSE
Q9DCJ5	NDUA8_MOUSE	NADH dehydrogenase [ubiquinone] 1 alpha subcomplex subunit 8 OS=Mus musculus GN=Ndufa8 PE=1 SV=3 [MASS=19992]	7	13	1.0	6.52E+09	7.27E+09	7.91E+09	7.49E+09	5.65E+09	6.21E+09	6.69E+09	6.07E+09	6.45E+09	7.45E+09	6.34E+09	6.63E+09	4.85E+09	0	0	0	0	0	0	0	0	0	0	0	0	1.26E-29	3, 4, 5, 6	1, 1, 13, 13, 3, 4, 9	1, 1, 13, 13, 3, 4, 9	NDUA8_MOUSE, SP:Q9DCJ5	SP|Q9DCJ5|NDUA8_MOUSE
P62702	RS4X_MOUSE	40S ribosomal protein S4, X isoform OS=Mus musculus GN=Rps4x PE=2 SV=2 [MASS=29598]	2	13	1.0	4.14E+08	3.61E+08	3.57E+08	3.31E+08	3.79E+08	3.66E+08	2.81E+08	4.22E+08	3.76E+08	3.88E+08	3.53E+08	2.85E+08	3.07E+08	8.75E-06	2.88E-05	3.27E-04	5.38E-03	1.41E-02	7.02E-06	9.31E-03	1.51E-04	3.82E-03	1.03E-05	3.62E-02	1.45E-04	9.21E-01	3, 4	13, 6	13, 6	RS4X_MOUSE, SP:P62702	SP|P62702|RS4X_MOUSE
Q62425	NDUA4_MOUSE	NADH dehydrogenase [ubiquinone] 1 alpha subcomplex subunit 4 OS=Mus musculus GN=Ndufa4 PE=1 SV=2 [MASS=9327]	3	13	1.0	6.68E+09	1.45E+10	1.03E+10	8.02E+09	8.55E+09	1.18E+10	1.03E+10	1.12E+10	7.92E+09	9.04E+09	9.34E+09	6.96E+09	7.22E+09	0	0	0	0	0	0	0	0	0	0	0	0	0	2, 2, 4, 6, 4	1, 12, 2, 5, 13	1, 12, 13, 14	NDUA4_MOUSE, SP:Q62425	SP|Q62425|NDUA4_MOUSE
P07310	KCRM_MOUSE	Creatine kinase M-type OS=Mus musculus GN=Ckm PE=1 SV=1 [MASS=43045]	13	13	1.0	4.80E+10	5.99E+10	5.59E+10	4.86E+10	5.17E+10	5.81E+10	5.31E+10	4.98E+10	4.61E+10	5.13E+10	4.70E+10	4.81E+10	4.34E+10	0	0	0	0	0	0	0	0	0	0	0	0	0	3, 3, 5, 4, 5, 5, 7	1, 1, 11, 13, 10, 10, 12, 12, 13, 3, 12, 5, 7, 12, 7, 13, 8	1, 10, 11, 11, 12, 12, 13, 14, 13, 15, 13, 22, 20, 23, 3, 26, 26, 7, 8	KCRM_MOUSE, SP:P07310	SP|P07310|KCRM_MOUSE
Q9CZW5	TOM70_MOUSE	Mitochondrial import receptor subunit TOM70 OS=Mus musculus GN=Tomm70a PE=1 SV=1 [MASS=67521]	2	5	1.0	9.03E+07	9.17E+07	6.88E+07	7.63E+07	6.07E+07	4.14E+07	7.44E+07	6.14E+07	9.93E+07	6.16E+07	4.05E+07	1.25E+08	5.24E+07	0.004	0.008	0.008	0.038	0.078	0.002	0.013	0.004	0.036	0.027	0.004	0.024	0.098	3	1, 5	1, 5	TOM70_MOUSE, SP:Q9CZW5	SP|Q9CZW5|TOM70_MOUSE
Q9D6U8	F162A_MOUSE	UPF0389 protein FAM162A OS=Mus musculus GN=Fam162a PE=2 SV=1 [MASS=17725]	2	13	1.0	7.03E+08	9.76E+08	8.13E+08	6.27E+08	7.77E+08	8.46E+08	7.34E+08	9.57E+08	6.96E+08	7.54E+08	7.90E+08	6.53E+08	6.81E+08	9.85E-11	1.36E-13	3.65E-09	1.42E-07	1.00E-07	7.10E-18	2.42E-09	1.63E-09	2.23E-07	2.72E-14	9.31E-07	6.56E-12	1.83E-03	3, 3, 5	13, 6, 13	13, 13, 6	F162A_MOUSE, SP:Q9D6U8	SP|Q9D6U8|F162A_MOUSE
Q3V132	ADT4_MOUSE	ADP/ATP translocase 4 OS=Mus musculus GN=Slc25a31 PE=2 SV=1 [MASS=35258]	1	11	1.0	3.63E+08	6.59E+08	4.70E+08	4.53E+08	1.30E+09	7.12E+08	5.21E+08	5.68E+08	4.80E+08	5.07E+08	5.25E+08	5.83E+08	5.41E+08	6.52E-05	3.67E-07	3.59E-06	1.10E-12	4.23E-06	2.88E-11	4.46E-06	5.18E-06	1.87E-04	8.64E-09	8.99E-06	2.02E-06	2.06E-02	4	11	11	ADT4_MOUSE, SP:Q3V132	SP|Q3V132|ADT4_MOUSE
Q9CQ69	QCR8_MOUSE	Cytochrome b-c1 complex subunit 8 OS=Mus musculus GN=Uqcrq PE=1 SV=3 [MASS=9768]	2	13	1.0	2.74E+09	3.25E+09	3.46E+09	2.96E+09	1.39E+09	2.33E+09	3.04E+09	2.82E+09	2.69E+09	2.55E+09	2.59E+09	2.99E+09	2.82E+09	1.53E-39	0	8.41E-41	7.27E-14	1.13E-21	0	5.47E-37	1.92E-35	2.70E-25	0	3.33E-31	3.07E-33	2.87E-17	5, 6	13, 2	13, 2	QCR8_MOUSE, SP:Q9CQ69	SP|Q9CQ69|QCR8_MOUSE
P19123	TNNC1_MOUSE	Troponin C, slow skeletal and cardiac muscles OS=Mus musculus GN=Tnnc1 PE=2 SV=1 [MASS=18420]	4	13	1.0	3.15E+09	3.63E+09	3.00E+09	3.01E+09	3.87E+09	3.27E+09	3.69E+09	3.71E+09	2.59E+09	4.01E+09	1.97E+09	4.39E+09	3.89E+09	0	0	5.61E-42	4.07E-37	4.08E-30	0	0	5.94E-35	1.01E-38	5.50E-34	0	0	9.49E-24	4, 5, 6	1, 11, 13, 3, 9	1, 11, 12, 3, 13	TNNC1_MOUSE, SP:P19123	SP|P19123|TNNC1_MOUSE
P68368	TBA4A_MOUSE	Tubulin alpha-4A chain OS=Mus musculus GN=Tuba4a PE=1 SV=1 [MASS=49924]	4	13	1.0	2.95E+09	3.38E+09	4.00E+09	3.33E+09	4.24E+09	4.19E+09	3.64E+09	3.67E+09	3.76E+09	3.57E+09	3.93E+09	3.09E+09	3.70E+09	0	0	0	0	5.33E-40	0	0	0	4.35E-37	0	2.44E-34	0	2.36E-22	4, 4, 7	13, 4, 8	13, 4, 8	TBA4A_MOUSE, SP:P68368	SP|P68368|TBA4A_MOUSE
Q9QYR9	ACOT2_MOUSE	Acyl-coenzyme A thioesterase 2, mitochondrial OS=Mus musculus GN=Acot2 PE=1 SV=1 [MASS=49652]	5	13	1.0	3.70E+09	4.76E+09	4.52E+09	3.51E+09	4.10E+09	4.28E+09	4.91E+09	4.31E+09	4.52E+09	3.76E+09	5.05E+09	3.63E+09	2.27E+09	0	0	0	0	2.80E-42	0	0	0	1.46E-40	0	8.41E-42	0	1.86E-14	2, 4, 7	11, 13, 5, 9	11, 13, 5, 9	ACOT2_MOUSE, SP:Q9QYR9	SP|Q9QYR9|ACOT2_MOUSE
O88441	MTX2_MOUSE	Metaxin-2 OS=Mus musculus GN=Mtx2 PE=1 SV=1 [MASS=29758]	2	8	1.0	8.51E+08	9.23E+08	1.09E+09	1.02E+09	1.04E+09	8.32E+08	1.10E+09	6.90E+08	9.34E+08	8.31E+08	8.92E+08	7.78E+08	7.70E+08	7.94E-12	9.12E-16	2.45E-14	4.15E-09	9.13E-07	8.40E-13	7.43E-13	2.64E-11	5.69E-07	6.32E-14	2.21E-07	4.24E-07	8.12E-04	3, 4	1, 8	1, 8	MTX2_MOUSE, SP:O88441	SP|O88441|MTX2_MOUSE
Q9CXV1	DHSD_MOUSE	Succinate dehydrogenase [ubiquinone] cytochrome b small subunit, mitochondrial OS=Mus musculus GN=Sdhd PE=2 SV=2 [MASS=17014]	2	13	1.0	1.45E+09	1.50E+09	1.31E+09	1.48E+09	1.19E+09	1.36E+09	1.51E+09	1.51E+09	1.08E+09	1.50E+09	1.18E+09	1.42E+09	1.51E+09	1.30E-22	6.90E-22	2.37E-22	5.66E-12	5.26E-13	4.22E-27	6.13E-20	3.06E-15	1.71E-15	2.99E-21	9.79E-16	5.06E-19	3.68E-09	3, 3, 4	10, 13, 8	10, 13, 8	DHSD_MOUSE, SP:Q9CXV1	SP|Q9CXV1|DHSD_MOUSE
Q64433	CH10_MOUSE	10 kDa heat shock protein, mitochondrial OS=Mus musculus GN=Hspe1 PE=1 SV=2 [MASS=10963]	2	13	1.0	3.13E+09	2.89E+09	2.39E+09	3.54E+09	3.69E+09	2.76E+09	3.47E+09	2.50E+09	2.86E+09	3.13E+09	2.56E+09	3.32E+09	3.02E+09	0	3.04E-38	0	1.63E-36	3.52E-26	0	0	1.80E-38	1.21E-31	0	9.19E-36	1.23E-30	7.41E-19	3, 5	11, 13	11, 13	CH10_MOUSE, SP:Q64433	SP|Q64433|CH10_MOUSE
Q06185	ATP5I_MOUSE	ATP synthase subunit e, mitochondrial OS=Mus musculus GN=Atp5i PE=1 SV=2 [MASS=8236]	3	13	1.0	1.39E+10	1.50E+10	1.43E+10	1.70E+10	1.95E+10	1.45E+10	1.66E+10	1.32E+10	1.32E+10	1.45E+10	1.35E+10	1.55E+10	1.39E+10	0	0	0	0	0	0	0	0	0	0	0	0	0	2, 3, 4, 3, 7	1, 2, 11, 13, 10, 2	11, 20, 17, 20	ATP5I_MOUSE, SP:Q06185	SP|Q06185|ATP5I_MOUSE
Q9D6J6	NDUV2_MOUSE	NADH dehydrogenase [ubiquinone] flavoprotein 2, mitochondrial OS=Mus musculus GN=Ndufv2 PE=1 SV=2 [MASS=27285]	8	13	1.0	1.22E+10	1.57E+10	1.50E+10	1.18E+10	1.56E+10	1.63E+10	1.40E+10	1.30E+10	1.29E+10	1.39E+10	1.48E+10	1.25E+10	1.05E+10	0	0	0	0	0	0	0	0	0	0	0	0	0	2, 5, 3, 4, 4, 6, 5, 7	12, 13, 9, 13	12, 13, 13, 9	NDUV2_MOUSE, SP:Q9D6J6	SP|Q9D6J6|NDUV2_MOUSE
Q9CQ91	NDUA3_MOUSE	NADH dehydrogenase [ubiquinone] 1 alpha subcomplex subunit 3 OS=Mus musculus GN=Ndufa3 PE=1 SV=1 [MASS=9331]	2	13	1.0	9.71E+08	1.84E+09	1.21E+09	1.14E+09	1.27E+09	1.16E+09	1.22E+09	1.50E+09	9.26E+08	1.26E+09	1.18E+09	9.41E+08	7.99E+08	3.37E-15	1.32E-20	3.35E-17	4.68E-13	5.57E-11	8.85E-34	4.59E-16	5.41E-13	5.51E-13	1.30E-21	3.48E-10	4.20E-19	2.74E-04	4, 6	13	13	NDUA3_MOUSE, SP:Q9CQ91	SP|Q9CQ91|NDUA3_MOUSE
P14148	RL7_MOUSE	60S ribosomal protein L7 OS=Mus musculus GN=Rpl7 PE=1 SV=2 [MASS=31419]	1	1	1.0	3.00E+07	6.09E+07	7.88E+07	5.33E+07	3.72E+07	5.47E+07	4.83E+07	5.51E+07	6.78E+07	4.45E+07	7.64E+07	4.01E+07	4.35E+07	0.064	0.006	0.022	0.083	0.052	0.010	0.037	0.014	0.064	0.004	0.068	0.031	0.121	3	1	1	RL7_MOUSE, SP:P14148	SP|P14148|RL7_MOUSE
O08528	HXK2_MOUSE	Hexokinase-2 OS=Mus musculus GN=Hk2 PE=1 SV=1 [MASS=102535]	7	12	1.0	2.60E+09	2.38E+09	2.23E+09	2.95E+09	2.30E+09	2.88E+09	2.48E+09	2.82E+09	2.92E+09	2.80E+09	2.61E+09	2.75E+09	2.57E+09	6.45E-41	5.05E-38	0	7.03E-24	2.32E-28	0	4.16E-33	1.68E-41	1.24E-29	0	4.26E-31	3.41E-36	2.49E-16	2, 3, 4, 6	1, 10, 11, 12, 2, 5, 8	1, 10, 11, 12, 2, 5, 8	HXK2_MOUSE, SP:O08528	SP|O08528|HXK2_MOUSE
Q9D023	BR44_MOUSE	Brain protein 44 OS=Mus musculus GN=Brp44 PE=1 SV=1 [MASS=14286]	5	13	1.0	2.22E+09	3.48E+09	3.23E+09	2.52E+09	3.08E+09	2.97E+09	2.83E+09	3.13E+09	2.27E+09	2.49E+09	3.04E+09	2.34E+09	2.13E+09	4.89E-34	0	6.53E-38	5.20E-32	1.17E-28	0	7.60E-37	1.73E-31	8.13E-26	0	5.27E-26	4.36E-39	2.79E-13	2, 4, 5	11, 13, 3	11, 13, 16, 3	BR44_MOUSE, SP:Q9D023	SP|Q9D023|BR44_MOUSE
Q9D6J5	NDUB8_MOUSE	NADH dehydrogenase [ubiquinone] 1 beta subcomplex subunit 8, mitochondrial OS=Mus musculus GN=Ndufb8 PE=1 SV=1 [MASS=21876]	4	13	1.0	7.60E+09	8.94E+09	8.70E+09	8.08E+09	7.19E+09	8.21E+09	7.66E+09	8.38E+09	7.05E+09	8.36E+09	8.01E+09	7.01E+09	6.58E+09	0	0	0	0	0	0	0	0	0	0	0	0	2.80E-41	3, 5, 5, 6, 6, 7	10, 13, 8, 9, 13	10, 13, 14, 13, 9, 8	NDUB8_MOUSE, SP:Q9D6J5	SP|Q9D6J5|NDUB8_MOUSE
Q4VAE3	TMM65_MOUSE	Transmembrane protein 65 OS=Mus musculus GN=Tmem65 PE=2 SV=1 [MASS=24918]	2	11	1.0	3.93E+08	4.82E+08	4.73E+08	5.20E+08	4.76E+08	5.14E+08	5.31E+08	4.17E+08	3.99E+08	3.46E+08	5.01E+08	3.64E+08	3.59E+08	1.85E-05	2.18E-07	2.42E-07	4.17E-04	3.67E-04	3.37E-08	2.15E-06	6.66E-05	1.21E-02	1.54E-08	3.51E-03	1.70E-04	3.80E-01	2, 4	11, 8	11, 8	TMM65_MOUSE, SP:Q4VAE3	SP|Q4VAE3|TMM65_MOUSE
Q9CPQ8	ATP5L_MOUSE	ATP synthase subunit g, mitochondrial OS=Mus musculus GN=Atp5l PE=1 SV=1 [MASS=11424]	4	13	1.0	1.06E+10	1.75E+10	1.39E+10	1.74E+10	1.08E+10	1.16E+10	1.44E+10	1.30E+10	1.14E+10	1.17E+10	1.17E+10	1.02E+10	9.59E+09	0	0	0	0	0	0	0	0	0	0	0	0	0	2, 3, 6, 4, 6	13, 5, 7, 6	13, 5, 7, 6	ATP5L_MOUSE, SP:Q9CPQ8	SP|Q9CPQ8|ATP5L_MOUSE
Q9CQR4	ACO13_MOUSE	Acyl-coenzyme A thioesterase 13 OS=Mus musculus GN=Acot13 PE=1 SV=1 [MASS=15183]	5	13	1.0	5.87E+09	5.52E+09	5.31E+09	5.24E+09	5.35E+09	4.89E+09	4.97E+09	4.72E+09	4.53E+09	5.34E+09	4.32E+09	5.61E+09	5.55E+09	0	0	0	0	0	0	0	0	0	0	0	0	3.62E-35	3, 4, 5, 5	1, 12, 13, 2, 3, 13	1, 12, 18, 2, 30	ACO13_MOUSE, SP:Q9CQR4	SP|Q9CQR4|ACO13_MOUSE
Q91WS0	CISD1_MOUSE	CDGSH iron sulfur domain-containing protein 1 OS=Mus musculus GN=Cisd1 PE=1 SV=1 [MASS=12097]	2	13	1.0	4.19E+09	4.22E+09	4.25E+09	5.53E+09	4.43E+09	3.50E+09	4.31E+09	3.29E+09	3.60E+09	3.87E+09	4.07E+09	3.75E+09	3.52E+09	0	0	0	0	4.83E-34	0	0	0	1.88E-40	0	1.12E-41	1.40E-41	2.14E-22	4, 5	13	13	CISD1_MOUSE, SP:Q91WS0	SP|Q91WS0|CISD1_MOUSE
P56135	ATPK_MOUSE	ATP synthase subunit f, mitochondrial OS=Mus musculus GN=Atp5j2 PE=1 SV=3 [MASS=10344]	2	13	1.0	1.24E+10	1.94E+10	1.66E+10	1.15E+10	1.36E+10	1.87E+10	1.49E+10	1.73E+10	1.44E+10	1.49E+10	1.68E+10	1.23E+10	1.13E+10	0	0	0	0	0	0	0	0	0	0	0	0	0	4, 5, 7	13, 6, 11	11, 6, 23	ATPK_MOUSE, SP:P56135	SP|P56135|ATPK_MOUSE
Q9D2G2	ODO2_MOUSE	Dihydrolipoyllysine-residue succinyltransferase component of 2-oxoglutarate dehydrogenase complex, mitochondrial OS=Mus musculus GN=Dlst PE=1 SV=1 [MASS=48994]	8	13	1.0	1.80E+10	1.94E+10	2.00E+10	1.95E+10	2.14E+10	1.98E+10	2.00E+10	1.74E+10	1.71E+10	1.92E+10	1.76E+10	1.98E+10	1.84E+10	0	0	0	0	0	0	0	0	0	0	0	0	0	2, 3, 3, 4, 4, 4, 5, 6, 7, 7	12, 13, 13, 3, 11, 8	12, 13, 13, 24, 24, 3, 8	ODO2_MOUSE, SP:Q9D2G2	SP|Q9D2G2|ODO2_MOUSE
P48787	TNNI3_MOUSE	Troponin I, cardiac muscle OS=Mus musculus GN=Tnni3 PE=1 SV=2 [MASS=24259]	7	13	1.0	5.46E+10	5.33E+10	6.57E+10	6.38E+10	7.12E+10	5.92E+10	6.08E+10	5.10E+10	5.57E+10	5.87E+10	5.66E+10	5.76E+10	6.49E+10	0	0	0	0	0	0	0	0	0	0	0	0	0	2, 6, 3, 3, 6, 7, 4, 5, 6	13, 2, 11, 13, 3, 5, 4, 11, 4, 7, 5, 13	11, 30, 13, 5, 24, 24, 4, 3, 5, 42	TNNI3_MOUSE, SP:P48787	SP|P48787|TNNI3_MOUSE
Q9Z239	PLM_MOUSE	Phospholemman OS=Mus musculus GN=Fxyd1 PE=1 SV=1 [MASS=10323]	1	12	0.99	3.09E+08	2.48E+08	2.10E+08	2.45E+08	2.71E+08	2.56E+08	2.15E+08	3.05E+08	2.09E+08	2.79E+08	1.79E+08	2.91E+08	2.37E+08	6.00E-04	1.73E-02	1.02E-02	1.13E-01	2.89E-01	1.38E-03	1.00E-01	7.63E-02	8.78E-02	4.58E-02	3.63E-02	8.19E-03	0.004	4	12	12	PLM_MOUSE, SP:Q9Z239	SP|Q9Z239|PLM_MOUSE
Q9CQC7	NDUB4_MOUSE	NADH dehydrogenase [ubiquinone] 1 beta subcomplex subunit 4 OS=Mus musculus GN=Ndufb4 PE=1 SV=3 [MASS=15081]	3	13	1.0	8.47E+09	1.08E+10	8.89E+09	9.95E+09	9.23E+09	7.54E+09	9.64E+09	8.22E+09	7.00E+09	8.23E+09	7.27E+09	7.20E+09	6.62E+09	0	0	0	0	0	0	0	0	0	0	0	0	0	4, 5, 5, 8	11, 13	11, 13	NDUB4_MOUSE, SP:Q9CQC7	SP|Q9CQC7|NDUB4_MOUSE
Q64310	SURF4_MOUSE	Surfeit locus protein 4 OS=Mus musculus GN=Surf4 PE=2 SV=1 [MASS=30381]	2	2	1.0	1.84E+08	1.90E+08	2.08E+08	1.22E+08	1.45E+08	1.55E+08	1.20E+08	2.17E+08	1.97E+08	2.04E+08	2.05E+08	1.86E+08	1.92E+08	7.77E-02	1.54E-02	0.001	0.003	0.003	1.79E-02	0.003	9.79E-02	5.73E-01	8.85E-03	6.41E-01	1.12E-01	0.007	2, 3	1, 2	1, 2	SURF4_MOUSE, SP:Q64310	SP|Q64310|SURF4_MOUSE
P35980	RL18_MOUSE	60S ribosomal protein L18 OS=Mus musculus GN=Rpl18 PE=2 SV=3 [MASS=21644]	2	3	1.0	1.90E+08	1.44E+08	1.81E+08	2.09E+08	1.86E+08	1.45E+08	1.69E+08	2.08E+08	1.62E+08	1.77E+08	1.64E+08	1.95E+08	1.74E+08	6.09E-02	5.17E-02	3.97E-02	0.001	0.004	1.58E-01	4.68E-01	3.68E-01	0.001	6.42E-02	5.01E-01	1.50E-01	0.010	2, 3	1, 3	1, 3	RL18_MOUSE, SP:P35980	SP|P35980|RL18_MOUSE
P63101	1433Z_MOUSE	14-3-3 protein zeta/delta OS=Mus musculus GN=Ywhaz PE=1 SV=1 [MASS=27771]	3	13	1.0	2.21E+09	2.37E+09	2.21E+09	1.98E+09	2.83E+09	2.23E+09	1.95E+09	2.51E+09	2.34E+09	2.41E+09	1.98E+09	2.11E+09	2.20E+09	7.96E-36	1.95E-38	5.10E-31	3.57E-30	3.83E-22	0	1.51E-26	4.85E-34	6.73E-26	5.43E-37	2.49E-24	1.12E-32	5.31E-14	4, 4, 5, 4, 6	1, 13, 13, 4, 12	1, 13, 12, 4, 13	1433Z_MOUSE, SP:P63101	SP|P63101|1433Z_MOUSE
Q99LC3	NDUAA_MOUSE	NADH dehydrogenase [ubiquinone] 1 alpha subcomplex subunit 10, mitochondrial OS=Mus musculus GN=Ndufa10 PE=1 SV=1 [MASS=40603]	12	13	1.0	2.66E+10	3.33E+10	3.08E+10	2.79E+10	2.99E+10	2.96E+10	2.98E+10	3.05E+10	2.55E+10	2.86E+10	2.59E+10	2.61E+10	2.25E+10	0	0	0	0	0	0	0	0	0	0	0	0	0	2, 5, 4, 5, 5, 6, 5, 7, 7, 8	10, 10, 13, 11, 13, 12, 13, 6, 13	10, 10, 13, 11, 13, 12, 13, 14, 16, 6	NDUAA_MOUSE, SP:Q99LC3	SP|Q99LC3|NDUAA_MOUSE
P56379	68MP_MOUSE	6.8 kDa mitochondrial proteolipid OS=Mus musculus GN=Mp68 PE=2 SV=1 [MASS=6698]	1	11	0.99	4.57E+09	5.12E+09	4.66E+09	6.22E+09	4.64E+09	3.71E+09	4.34E+09	4.80E+09	3.51E+09	3.89E+09	4.33E+09	3.52E+09	4.45E+09	0	0	0	2.97E-40	5.70E-25	0	3.78E-40	1.99E-32	5.54E-25	3.37E-38	1.88E-29	5.61E-42	4.40E-25	5	11	11	68MP_MOUSE, SP:P56379	SP|P56379|68MP_MOUSE
P35293	RAB18_MOUSE	Ras-related protein Rab-18 OS=Mus musculus GN=Rab18 PE=2 SV=2 [MASS=23035]	1	1	0.96	3.63E+07	3.28E+07	3.35E+07	2.84E+07	3.43E+07	3.70E+07	2.75E+07	4.18E+07	4.09E+07	3.97E+07	3.82E+07	1.42E+07	5.35E+07	0.045	0.046	0.070	0.091	0.090	0.041	0.088	0.044	0.075	0.031	0.186	0.051	0.096	2	1	1	RAB18_MOUSE, SP:P35293	SP|P35293|RAB18_MOUSE
Q9CXZ1	NDUS4_MOUSE	NADH dehydrogenase [ubiquinone] iron-sulfur protein 4, mitochondrial OS=Mus musculus GN=Ndufs4 PE=1 SV=3 [MASS=19785]	7	13	1.0	8.50E+09	9.72E+09	8.61E+09	9.57E+09	9.99E+09	9.03E+09	8.69E+09	8.70E+09	7.26E+09	8.98E+09	7.90E+09	9.00E+09	7.65E+09	0	0	0	0	0	0	0	0	0	0	0	0	0	2, 3, 4, 4, 5, 7	10, 11, 12, 13	10, 11, 12, 13	NDUS4_MOUSE, SP:Q9CXZ1	SP|Q9CXZ1|NDUS4_MOUSE
P99027	RLA2_MOUSE	60S acidic ribosomal protein P2 OS=Mus musculus GN=Rplp2 PE=1 SV=3 [MASS=11651]	3	13	1.0	2.13E+09	2.18E+09	2.11E+09	1.93E+09	2.24E+09	1.97E+09	2.01E+09	2.38E+09	2.23E+09	2.32E+09	2.35E+09	1.78E+09	1.80E+09	6.00E-34	9.48E-36	8.57E-30	1.71E-23	2.64E-19	8.66E-40	4.08E-27	3.14E-32	1.02E-24	0	3.74E-20	1.13E-30	2.88E-11	3, 5, 6, 7	1, 13, 13, 9	1, 13, 13, 9	RLA2_MOUSE, SP:P99027	SP|P99027|RLA2_MOUSE
P21550	ENOB_MOUSE	Beta-enolase OS=Mus musculus GN=Eno3 PE=1 SV=3 [MASS=47025]	9	13	1.0	4.16E+10	4.03E+10	4.16E+10	3.95E+10	4.97E+10	4.34E+10	4.56E+10	4.08E+10	4.10E+10	4.21E+10	3.60E+10	3.87E+10	3.46E+10	0	0	0	0	0	0	0	0	0	0	0	0	0	2, 3, 3, 4, 5, 5, 5, 6, 6, 7, 7, 8	1, 2, 3, 11, 10, 13, 12, 12, 13, 13, 2, 5, 13, 8	12, 12, 13, 13, 13, 33, 13, 5, 14, 18, 2, 8	ENOB_MOUSE, SP:P21550	SP|P21550|ENOB_MOUSE
O09111	NDUBB_MOUSE	NADH dehydrogenase [ubiquinone] 1 beta subcomplex subunit 11, mitochondrial OS=Mus musculus GN=Ndufb11 PE=1 SV=2 [MASS=17444]	4	13	1.0	2.65E+09	2.94E+09	2.75E+09	2.36E+09	3.04E+09	2.77E+09	2.80E+09	2.66E+09	2.15E+09	2.50E+09	2.52E+09	2.59E+09	2.49E+09	5.61E-42	0	2.12E-36	4.36E-32	1.76E-27	0	1.15E-37	2.73E-31	1.27E-26	0	2.15E-29	2.28E-34	8.10E-16	4, 5, 5, 6	12, 12, 13, 6, 7	12, 12, 14, 6, 7	NDUBB_MOUSE, SP:O09111	SP|O09111|NDUBB_MOUSE
Q9CZB0	C560_MOUSE	Succinate dehydrogenase cytochrome b560 subunit, mitochondrial OS=Mus musculus GN=Sdhc PE=2 SV=1 [MASS=18382]	4	12	1.0	2.62E+09	3.16E+09	4.20E+09	2.55E+09	1.23E+09	2.24E+09	2.04E+09	2.42E+09	2.21E+09	2.45E+09	3.11E+09	3.16E+09	2.02E+09	4.46E-39	0	9.55E-38	2.03E-12	3.88E-21	0	8.58E-27	1.16E-30	3.77E-25	0	1.52E-35	9.34E-30	1.16E-12	3, 4, 5	10, 12, 2	10, 12, 2	C560_MOUSE, SP:Q9CZB0	SP|Q9CZB0|C560_MOUSE
P53986	MOT1_MOUSE	Monocarboxylate transporter 1 OS=Mus musculus GN=Slc16a1 PE=1 SV=1 [MASS=53267]	4	13	1.0	1.49E+09	1.36E+09	1.42E+09	1.38E+09	1.53E+09	1.23E+09	1.36E+09	1.32E+09	1.36E+09	1.30E+09	1.30E+09	1.42E+09	1.44E+09	5.44E-24	1.04E-24	1.73E-21	4.65E-16	7.03E-12	1.56E-25	2.64E-18	8.08E-20	1.30E-13	2.68E-24	3.63E-16	5.62E-17	8.36E-09	3, 5	11, 13	11, 13	MOT1_MOUSE, SP:P53986	SP|P53986|MOT1_MOUSE
P52480	KPYM_MOUSE	Pyruvate kinase isozymes M1/M2 OS=Mus musculus GN=Pkm2 PE=1 SV=4 [MASS=57845]	14	13	1.0	1.89E+10	2.51E+10	2.53E+10	2.00E+10	2.03E+10	2.58E+10	2.20E+10	2.16E+10	2.07E+10	2.18E+10	2.24E+10	1.97E+10	1.89E+10	0	0	0	0	0	0	0	0	0	0	0	0	0	2, 3, 3, 5, 3, 6, 4, 4, 5, 4, 6, 5, 6	1, 1, 13, 1, 4, 10, 13, 11, 13, 4, 5, 13, 7, 8, 13	1, 1, 13, 1, 4, 10, 13, 11, 13, 13, 5, 13, 8, 18, 4, 7	KPYM_MOUSE, SP:P52480	SP|P52480|KPYM_MOUSE
Q9DC70	NDUS7_MOUSE	NADH dehydrogenase [ubiquinone] iron-sulfur protein 7, mitochondrial OS=Mus musculus GN=Ndufs7 PE=1 SV=1 [MASS=24683]	3	13	1.0	4.24E+09	5.23E+09	4.96E+09	3.88E+09	4.17E+09	4.97E+09	3.98E+09	4.50E+09	4.00E+09	4.34E+09	4.24E+09	3.86E+09	3.66E+09	0	0	0	1.40E-42	0	0	0	0	0	0	0	0	1.95E-23	3, 4, 5	13	13	NDUS7_MOUSE, SP:Q9DC70	SP|Q9DC70|NDUS7_MOUSE
P02089	HBB2_MOUSE	Hemoglobin subunit beta-2 OS=Mus musculus GN=Hbb-b2 PE=1 SV=2 [MASS=15878]	1	1	1.0	9.52E+06	2.34E+07	1.86E+07	9.85E+06	8.69E+06	6.79E+06	1.29E+07	1.46E+07	1.65E+07	1.06E+07	2.20E+07	9.26E+06	5.03E+07	0.196	0.107	0.195	0.253	0.286	0.074	0.181	0.141	0.231	0.080	0.237	0.169	0.103	3	1	1	HBB2_MOUSE, SP:P02089	SP|P02089|HBB2_MOUSE
O55126	NIPS2_MOUSE	Protein NipSnap homolog 2 OS=Mus musculus GN=Gbas PE=2 SV=1 [MASS=32932]	4	13	1.0	5.42E+09	5.57E+09	5.27E+09	6.44E+09	7.34E+09	5.75E+09	6.88E+09	4.87E+09	4.43E+09	5.28E+09	4.67E+09	6.07E+09	5.47E+09	0	0	0	0	0	0	0	0	0	0	0	0	2.47E-34	2, 3, 3, 4	13, 5, 8	13, 13, 18, 18, 8	NIPS2_MOUSE, SP:O55126	SP|O55126|NIPS2_MOUSE
P32020	NLTP_MOUSE	Non-specific lipid-transfer protein OS=Mus musculus GN=Scp2 PE=1 SV=3 [MASS=59125]	2	13	1.0	6.10E+08	5.05E+08	4.59E+08	5.11E+08	6.60E+08	5.39E+08	6.56E+08	5.86E+08	4.33E+08	5.57E+08	4.52E+08	5.83E+08	5.33E+08	5.82E-09	4.18E-07	4.25E-07	3.84E-06	1.97E-04	1.21E-08	4.84E-08	2.26E-05	4.69E-05	1.37E-07	8.34E-06	8.67E-07	2.16E-02	3	12, 13	12, 13	NLTP_MOUSE, SP:P32020	SP|P32020|NLTP_MOUSE
Q9CQ54	NDUC2_MOUSE	NADH dehydrogenase [ubiquinone] 1 subunit C2 OS=Mus musculus GN=Ndufc2 PE=1 SV=1 [MASS=14164]	5	13	1.0	5.28E+09	5.38E+09	6.58E+09	5.57E+09	4.32E+09	4.87E+09	4.72E+09	5.06E+09	3.88E+09	4.81E+09	4.66E+09	5.28E+09	4.99E+09	0	0	0	0	0	0	0	0	0	0	0	0	1.00E-32	2, 3, 4, 5, 4, 6, 5	11, 13, 12, 13, 5, 8	11, 13, 12, 13, 5, 8	NDUC2_MOUSE, SP:Q9CQ54	SP|Q9CQ54|NDUC2_MOUSE
Q9DC69	NDUA9_MOUSE	NADH dehydrogenase [ubiquinone] 1 alpha subcomplex subunit 9, mitochondrial OS=Mus musculus GN=Ndufa9 PE=1 SV=1 [MASS=42509]	18	13	1.0	2.65E+10	3.29E+10	2.97E+10	2.94E+10	2.87E+10	2.80E+10	2.95E+10	3.08E+10	2.60E+10	2.63E+10	2.73E+10	2.80E+10	2.25E+10	0	0	0	0	0	0	0	0	0	0	0	0	0	2, 3, 3, 4, 4, 4, 5, 5, 5, 6, 6, 8	11, 11, 13, 13, 4, 5, 6, 13, 6, 7, 7, 9, 13	10, 13, 11, 11, 13, 13, 13, 9, 17, 5, 7	NDUA9_MOUSE, SP:Q9DC69	SP|Q9DC69|NDUA9_MOUSE
P62204	CALM_MOUSE	Calmodulin OS=Mus musculus GN=Calm1 PE=1 SV=2 [MASS=16838]	1	1	1.0	1.08E+08	6.89E+07	8.50E+07	1.09E+08	7.18E+07	5.10E+07	1.10E+08	6.14E+07	1.44E+08	8.39E+07	2.03E+08	7.12E+07	2.91E+07	0.002	0.004	0.002	0.027	0.057	0.006	0.003	6.94E-01	0.018	8.56E-03	0.023	0.024	0.168	7	1	1	CALM_MOUSE, SP:P62204	SP|P62204|CALM_MOUSE
P99028	QCR6_MOUSE	Cytochrome b-c1 complex subunit 6, mitochondrial OS=Mus musculus GN=Uqcrh PE=1 SV=2 [MASS=10435]	2	11	1.0	1.54E+09	1.84E+09	9.13E+08	1.72E+09	7.76E+08	1.10E+08	1.15E+09	7.31E+08	1.34E+09	1.36E+09	2.10E+09	4.16E+09	3.30E+09	1.46E-23	0	1.77E-25	5.08E-07	0.024	4.21E-31	2.31E-14	4.16E-18	1.76E-13	2.00E-35	0	3.31E-08	1.05E-20	3, 7	11, 2	11, 2	QCR6_MOUSE, SP:P99028	SP|P99028|QCR6_MOUSE
Q8R0Y8	S2542_MOUSE	Solute carrier family 25 member 42 OS=Mus musculus GN=Slc25a42 PE=2 SV=1 [MASS=35241]	1	2	0.99	3.91E+07	9.57E+07	8.84E+07	4.99E+07	1.10E+08	9.78E+07	9.85E+07	7.08E+07	7.65E+07	7.53E+07	8.55E+07	5.05E+07	4.29E+07	0.044	0.006	0.028	0.010	0.017	0.004	0.007	0.014	0.028	0.007	0.052	0.024	0.126	4	2	2	S2542_MOUSE, SP:Q8R0Y8	SP|Q8R0Y8|S2542_MOUSE
P14115	RL27A_MOUSE	60S ribosomal protein L27a OS=Mus musculus GN=Rpl27a PE=1 SV=4 [MASS=16589]	1	13	1.0	2.32E+08	2.15E+08	2.60E+08	2.02E+08	2.23E+08	2.15E+08	2.26E+08	2.47E+08	2.46E+08	2.38E+08	2.18E+08	2.32E+08	2.47E+08	1.20E-02	1.88E-03	5.13E-02	3.75E-01	6.62E-01	6.19E-03	6.39E-02	1.75E-02	2.32E-01	5.49E-03	1.74E-01	4.43E-02	0.003	3	13	13	RL27A_MOUSE, SP:P14115	SP|P14115|RL27A_MOUSE
Q9EQ20	MMSA_MOUSE	Methylmalonate-semialdehyde dehydrogenase [acylating], mitochondrial OS=Mus musculus GN=Aldh6a1 PE=1 SV=1 [MASS=57915]	17	13	1.0	1.13E+10	1.28E+10	1.27E+10	1.39E+10	1.49E+10	1.59E+10	1.51E+10	1.14E+10	1.07E+10	1.18E+10	1.17E+10	1.13E+10	1.13E+10	0	0	0	0	0	0	0	0	0	0	0	0	0	2, 3, 4, 4, 5, 5, 7	10, 13, 12, 12, 13, 13, 2, 4, 9, 6, 13, 7	10, 13, 12, 12, 13, 13, 13, 6, 2, 4, 9, 7	MMSA_MOUSE, SP:Q9EQ20	SP|Q9EQ20|MMSA_MOUSE
P62259	1433E_MOUSE	14-3-3 protein epsilon OS=Mus musculus GN=Ywhae PE=1 SV=1 [MASS=29174]	8	13	1.0	4.15E+09	4.68E+09	4.14E+09	3.77E+09	5.40E+09	4.39E+09	4.66E+09	4.77E+09	4.53E+09	4.41E+09	4.07E+09	5.03E+09	4.38E+09	0	0	0	0	0	0	0	0	0	0	0	0	8.59E-29	2, 3, 3, 4, 4, 6	10, 13, 11, 12, 13, 13, 5, 7, 8, 9	10, 13, 11, 12, 13, 13, 5, 7, 8, 9	1433E_MOUSE, SP:P62259	SP|P62259|1433E_MOUSE
Q60931	VDAC3_MOUSE	Voltage-dependent anion-selective channel protein 3 OS=Mus musculus GN=Vdac3 PE=1 SV=1 [MASS=30753]	6	13	1.0	7.35E+09	9.04E+09	7.92E+09	8.92E+09	8.80E+09	7.68E+09	7.23E+09	7.50E+09	6.44E+09	7.39E+09	6.70E+09	7.20E+09	6.78E+09	0	0	0	0	0	0	0	0	0	0	0	0	1.40E-42	2, 3, 4, 5, 5	13, 4, 6	13, 14, 4, 7	VDAC3_MOUSE, SP:Q60931	SP|Q60931|VDAC3_MOUSE
Q9D6R2	IDH3A_MOUSE	Isocitrate dehydrogenase [NAD] subunit alpha, mitochondrial OS=Mus musculus GN=Idh3a PE=1 SV=1 [MASS=39638]	12	13	1.0	4.93E+10	4.98E+10	5.11E+10	4.47E+10	4.72E+10	5.09E+10	4.57E+10	4.79E+10	4.63E+10	4.83E+10	4.70E+10	4.69E+10	4.24E+10	0	0	0	0	0	0	0	0	0	0	0	0	0	2, 3, 3, 6, 4, 4, 7, 5, 5, 6, 5, 8, 6, 7, 8	1, 12, 1, 8, 13, 2, 3, 6, 7	1, 15, 1, 8, 12, 13, 15, 3, 30, 68, 74, 7	IDH3A_MOUSE, SP:Q9D6R2	SP|Q9D6R2|IDH3A_MOUSE
P03911	NU4M_MOUSE	NADH-ubiquinone oxidoreductase chain 4 OS=Mus musculus GN=Mtnd4 PE=1 SV=1 [MASS=51882]	6	13	1.0	7.19E+09	7.63E+09	7.01E+09	7.69E+09	6.25E+09	7.38E+09	6.54E+09	7.68E+09	7.20E+09	6.37E+09	7.46E+09	6.89E+09	5.47E+09	0	0	0	0	0	0	0	0	0	0	0	0	4.87E-36	3, 3, 4, 4, 5, 6, 7, 7	1, 2, 8, 12, 13, 13, 6, 9	13, 14, 22, 6, 9	NU4M_MOUSE, SP:P03911	SP|P03911|NU4M_MOUSE
Q06890	CLUS_MOUSE	Clusterin OS=Mus musculus GN=Clu PE=1 SV=1 [MASS=51655]	2	13	1.0	9.47E+07	1.25E+08	9.28E+07	1.24E+08	1.55E+08	1.19E+08	1.45E+08	1.45E+08	1.09E+08	1.24E+08	1.09E+08	9.47E+07	1.34E+08	0.003	0.003	0.001	0.002	0.008	3.85E-01	0.001	0.003	0.006	8.47E-01	0.011	0.001	0.020	2, 3	1, 13	1, 13	CLUS_MOUSE, SP:Q06890	SP|Q06890|CLUS_MOUSE
P62806	H4_MOUSE	Histone H4 OS=Mus musculus GN=Hist1h4a PE=1 SV=2 [MASS=11367]	4	13	1.0	2.45E+10	2.75E+10	2.46E+10	2.44E+10	2.33E+10	1.89E+10	2.01E+10	2.56E+10	2.13E+10	2.33E+10	2.12E+10	1.98E+10	1.72E+10	0	0	0	0	0	0	0	0	0	0	0	0	0	2, 5, 3, 5, 5	1, 13, 11, 13, 7, 12, 9	11, 12, 11, 13, 14, 9	H4_MOUSE, SP:P62806	SP|P62806|H4_MOUSE
Q9D172	ES1_MOUSE	ES1 protein homolog, mitochondrial OS=Mus musculus GN=D10Jhu81e PE=1 SV=1 [MASS=28090]	4	13	1.0	6.21E+09	8.56E+09	7.54E+09	6.33E+09	6.39E+09	9.11E+09	6.69E+09	6.47E+09	6.39E+09	7.11E+09	7.97E+09	6.30E+09	5.53E+09	0	0	0	0	0	0	0	0	0	0	0	0	6.17E-35	3, 6, 7	10, 13	10, 13	ES1_MOUSE, SP:Q9D172	SP|Q9D172|ES1_MOUSE
Q9DCX2	ATP5H_MOUSE	ATP synthase subunit d, mitochondrial OS=Mus musculus GN=Atp5h PE=1 SV=3 [MASS=18749]	11	13	1.0	8.43E+10	9.03E+10	9.03E+10	8.78E+10	8.38E+10	8.85E+10	8.94E+10	8.02E+10	8.26E+10	8.49E+10	7.86E+10	8.80E+10	7.38E+10	0	0	0	0	0	0	0	0	0	0	0	0	0	2, 5, 3, 3, 4, 5, 4, 4, 5, 5, 5, 6, 6, 7, 8	1, 3, 11, 13, 12, 13, 4, 6, 10, 7, 11, 13, 7, 12, 8, 13	10, 6, 13, 14, 25, 32, 31, 36, 4, 5	ATP5H_MOUSE, SP:Q9DCX2	SP|Q9DCX2|ATP5H_MOUSE
P20152	VIME_MOUSE	Vimentin OS=Mus musculus GN=Vim PE=1 SV=3 [MASS=53687]	12	13	1.0	9.44E+09	9.88E+09	9.96E+09	1.19E+10	1.30E+10	8.60E+09	8.53E+09	1.04E+10	1.41E+10	1.30E+10	1.16E+10	1.07E+10	1.32E+10	0	0	0	0	0	0	0	0	0	0	0	0	0	2, 3, 3, 5, 3, 6, 4, 4, 5, 4, 6, 5	1, 9, 11, 11, 13, 12, 13, 5, 12, 7, 13	1, 9, 11, 11, 13, 12, 12, 5, 13, 13, 7, 14	VIME_MOUSE, SP:P20152	SP|P20152|VIME_MOUSE
Q9DCW4	ETFB_MOUSE	Electron transfer flavoprotein subunit beta OS=Mus musculus GN=Etfb PE=1 SV=3 [MASS=27623]	13	13	1.0	4.39E+10	4.86E+10	5.16E+10	5.01E+10	4.91E+10	4.68E+10	4.97E+10	3.81E+10	3.84E+10	4.32E+10	4.12E+10	4.69E+10	3.87E+10	0	0	0	0	0	0	0	0	0	0	0	0	0	2, 5, 4, 4, 5, 4, 6, 5, 6, 9	1, 10, 11, 12, 12, 13, 13, 3, 3, 6, 13, 5, 7	11, 12, 12, 13, 13, 14, 16, 6, 3, 5, 7	ETFB_MOUSE, SP:Q9DCW4	SP|Q9DCW4|ETFB_MOUSE
Q64523	H2A1F_MOUSE	Histone H2A type 2-C OS=Mus musculus GN=Hist2h2ac PE=1 SV=3 [MASS=13988]	1	12	1.0	8.09E+09	1.37E+10	1.10E+10	7.74E+09	1.22E+10	1.20E+10	9.98E+09	1.37E+10	1.12E+10	1.00E+10	1.11E+10	7.94E+09	6.33E+09	0	0	0	0	0	0	0	0	0	0	0	0	4.79E-40	6, 7	9, 12	12, 9	H2A1F_MOUSE, SP:Q8CGP5	SP|Q8CGP5|H2A1F_MOUSE,SP|Q8CGP7|H2A1K_MOUSE,SP|P22752|H2A1_MOUSE,SP|Q8BFU2| H2A3_MOUSE,SP|Q6GSS7|H2A2A_MOUSE,SP|Q8R1M2|H2AJ_MOUSE,SP|Q64523|H2A2C_MOUS[illegible]
Q9Z1P6	NDUA7_MOUSE	NADH dehydrogenase [ubiquinone] 1 alpha subcomplex subunit 7 OS=Mus musculus GN=Ndufa7 PE=1 SV=3 [MASS=12576]	1	13	1.0	1.78E+09	1.33E+09	1.23E+09	1.56E+09	1.78E+09	1.34E+09	1.54E+09	1.33E+09	1.22E+09	1.44E+09	1.08E+09	1.39E+09	1.49E+09	2.94E-25	4.63E-19	9.28E-22	4.93E-17	9.84E-12	2.56E-22	1.28E-18	9.62E-16	7.73E-14	8.29E-18	4.94E-14	1.29E-15	6.34E-08	4	13	13	NDUA7_MOUSE, SP:Q9Z1P6	SP|Q9Z1P6|NDUA7_MOUSE
P14152	MDHC_MOUSE	Malate dehydrogenase, cytoplasmic OS=Mus musculus GN=Mdh1 PE=1 SV=3 [MASS=36511]	12	13	1.0	6.71E+10	6.87E+10	7.03E+10	5.90E+10	6.12E+10	6.36E+10	6.18E+10	5.71E+10	5.49E+10	5.91E+10	5.78E+10	6.04E+10	5.24E+10	0	0	0	0	0	0	0	0	0	0	0	0	0	2, 2, 3, 4, 7, 3, 3, 5, 4, 4, 5, 4, 7, 5, 6, 8	1, 11, 12, 13, 2, 4, 6, 13, 4, 10, 5, 13, 7, 13	10, 5, 11, 12, 12, 13, 13, 13, 7, 2, 27, 5	MDHC_MOUSE, SP:P14152	SP|P14152|MDHC_MOUSE
P20108	PRDX3_MOUSE	Thioredoxin-dependent peroxide reductase, mitochondrial OS=Mus musculus GN=Prdx3 PE=1 SV=1 [MASS=28127]	5	13	1.0	7.88E+09	9.75E+09	8.98E+09	8.90E+09	9.96E+09	1.04E+10	1.00E+10	8.13E+09	8.55E+09	9.00E+09	1.01E+10	8.34E+09	7.35E+09	0	0	0	0	0	0	0	0	0	0	0	0	0	2, 4, 4, 4, 5, 4, 6, 5	12, 13, 2, 13, 7, 12	12, 12, 7, 13, 15, 2	PRDX3_MOUSE, SP:P20108	SP|P20108|PRDX3_MOUSE
P03899	NU3M_MOUSE	NADH-ubiquinone oxidoreductase chain 3 OS=Mus musculus GN=Mtnd3 PE=3 SV=3 [MASS=13219]	1	10	0.99	1.80E+08	1.93E+08	7.59E+08	2.58E+08	5.81E+07	2.71E+07	7.75E+07	6.91E+07	1.10E+08	1.21E+08	1.60E+08	4.95E+08	4.28E+08	8.61E-02	2.32E-12	4.98E-03	0.041	0.125	1.46E-02	0.012	0.003	0.006	7.07E-02	8.40E-05	0.018	1.19E-01	4	10	10	NU3M_MOUSE, SP:P03899	SP|P03899|NU3M_MOUSE
P02088	HBB1_MOUSE	Hemoglobin subunit beta-1 OS=Mus musculus GN=Hbb-b1 PE=1 SV=2 [MASS=15840]	5	13	1.0	7.28E+10	4.72E+10	5.96E+10	3.24E+10	6.38E+10	6.16E+10	5.69E+10	5.23E+10	5.12E+10	6.55E+10	4.53E+10	3.25E+10	5.32E+10	0	0	0	0	0	0	0	0	0	0	0	0	0	2, 2, 3, 5, 7, 4, 5, 6, 7	1, 1, 3, 7, 9, 11, 12, 13, 6, 13	1, 12, 13, 33, 43	HBB1_MOUSE, SP:P02088	SP|P02088|HBB1_MOUSE
Q9CR21	ACPM_MOUSE	Acyl carrier protein, mitochondrial OS=Mus musculus GN=Ndufab1 PE=1 SV=1 [MASS=17370]	3	13	1.0	7.95E+09	6.61E+09	6.26E+09	8.07E+09	8.25E+09	6.65E+09	8.33E+09	5.67E+09	6.92E+09	8.21E+09	5.75E+09	9.05E+09	8.18E+09	0	0	0	0	0	0	0	0	0	0	0	0	0	3, 5, 4, 5	10, 12, 8, 13	10, 12, 13, 9	ACPM_MOUSE, SP:Q9CR21	SP|Q9CR21|ACPM_MOUSE
P70670	NACAM_MOUSE	Nascent polypeptide-associated complex subunit alpha, muscle-specific form OS=Mus musculus GN=Naca PE=1 SV=1 [MASS=220600]	22	13	1.0	2.65E+09	2.63E+09	2.66E+09	2.67E+09	2.70E+09	2.43E+09	2.35E+09	2.63E+09	2.49E+09	2.87E+09	2.32E+09	2.59E+09	2.61E+09	0	0	0	2.17E-29	1.38E-24	0	1.11E-32	2.70E-37	1.45E-31	0	1.54E-30	5.88E-35	5.14E-17	2, 3, 3, 4, 4, 5	1, 10, 11, 12, 13, 2, 3, 3, 13, 4, 7, 8, 9	1, 10, 11, 12, 13, 13, 3, 2, 3, 4, 7, 8, 9	NACAM_MOUSE, SP:P70670	SP|P70670|NACAM_MOUSE
Q9ESD7	DYSF_MOUSE	Dysferlin OS=Mus musculus GN=Dysf PE=1 SV=3 [MASS=237909]	1	2	0.99	6.46E+07	6.15E+07	7.10E+07	6.78E+07	9.57E+07	8.44E+07	6.61E+07	9.66E+07	8.26E+07	1.02E+08	6.82E+07	8.54E+07	6.69E+07	0.013	0.008	0.012	0.014	0.022	0.010	0.020	0.008	0.011	0.007	0.015	0.007	0.073	3	2	2	DYSF_MOUSE, SP:Q9ESD7	SP|Q9ESD7|DYSF_MOUSE
P23242	CXA1_MOUSE	Gap junction alpha-1 protein OS=Mus musculus GN=Gja1 PE=1 SV=2 [MASS=43004]	4	12	1.0	2.03E+09	1.58E+09	1.06E+09	1.41E+09	1.20E+09	1.01E+09	1.15E+09	1.33E+09	9.39E+08	1.17E+09	9.74E+08	1.03E+09	9.15E+08	1.64E-32	6.80E-18	1.16E-21	4.37E-12	2.49E-09	1.96E-29	4.90E-15	4.93E-13	7.77E-12	7.41E-18	4.72E-11	4.77E-17	8.67E-05	3, 3, 4, 5	12, 4, 8, 10, 9	10, 8, 12, 4, 9	CXA1_MOUSE, SP:P23242	SP|P23242|CXA1_MOUSE
P84228	H32_MOUSE	Histone H3.2 OS=Mus musculus GN=Hist1h3b PE=1 SV=2 [MASS=15388]	1	1	1.0	1.03E+08	8.96E+07	1.35E+08	7.68E+07	3.83E+07	2.92E+07	3.45E+07	2.24E+07	4.26E+07	3.53E+07	1.52E+08	6.38E+07	1.49E+07	0.002	3.67E-01	0.008	0.080	0.119	0.002	0.068	0.042	0.088	1.06E-01	0.029	0.120	0.246	6	1	1	H32_MOUSE, SP:P84228	SP|P84228|H32_MOUSE
Q9D3D9	ATPD_MOUSE	ATP synthase subunit delta, mitochondrial OS=Mus musculus GN=Atp5d PE=1 SV=1 [MASS=17600]	2	13	1.0	1.97E+10	1.50E+10	1.50E+10	1.74E+10	2.14E+10	1.64E+10	1.81E+10	1.46E+10	1.51E+10	1.72E+10	1.15E+10	1.93E+10	1.56E+10	0	0	0	0	0	0	0	0	0	0	0	0	0	5	13, 3	13, 14, 3	ATPD_MOUSE, SP:Q9D3D9	SP|Q9D3D9|ATPD_MOUSE
P47955	RLA1_MOUSE	60S acidic ribosomal protein P1 OS=Mus musculus GN=Rplp1 PE=1 SV=1 [MASS=11475]	1	13	1.0	5.46E+08	8.41E+08	6.70E+08	4.56E+08	8.21E+08	8.58E+08	7.14E+08	9.39E+08	8.02E+08	8.12E+08	7.65E+08	5.86E+08	5.61E+08	4.90E-08	6.97E-11	2.73E-06	5.88E-08	9.12E-08	6.54E-15	5.88E-09	5.24E-11	6.07E-08	1.39E-13	6.61E-06	1.61E-11	1.33E-02	3, 5	10, 13	10, 13	RLA1_MOUSE, SP:P47955	SP|P47955|RLA1_MOUSE
Q9CQZ6	NDUB3_MOUSE	NADH dehydrogenase [ubiquinone] 1 beta subcomplex subunit 3 OS=Mus musculus GN=Ndufb3 PE=1 SV=1 [MASS=11692]	3	13	1.0	3.67E+09	3.83E+09	3.50E+09	4.19E+09	4.20E+09	2.93E+09	4.04E+09	3.49E+09	2.84E+09	3.46E+09	2.65E+09	3.77E+09	3.33E+09	0	0	0	1.96E-41	1.53E-27	0	0	2.25E-38	1.07E-34	0	4.23E-40	2.80E-42	3.19E-20	4, 5	10, 13	10, 13	NDUB3_MOUSE, SP:Q9CQZ6	SP|Q9CQZ6|NDUB3_MOUSE
A2AMM0	MURC_MOUSE	Muscle-related coiled-coil protein OS=Mus musculus GN=Murc PE=2 SV=1 [MASS=41008]	4	13	1.0	8.71E+08	8.02E+08	1.10E+09	9.20E+08	9.03E+08	6.90E+08	8.53E+08	7.16E+08	9.97E+08	9.82E+08	9.33E+08	1.19E+09	1.23E+09	1.63E-13	7.98E-19	6.72E-14	7.02E-09	4.69E-06	1.50E-14	5.27E-11	4.50E-14	6.67E-10	4.01E-17	2.59E-13	1.32E-08	2.62E-07	3, 3, 4, 4	13, 2, 8	13, 2, 8	MURC_MOUSE, SP:A2AMM0	SP|A2AMM0|MURC_MOUSE
Q9D0F9	PGM1_MOUSE	Phosphoglucomutase-1 OS=Mus musculus GN=Pgm1 PE=1 SV=3 [MASS=61517]	15	13	1.0	7.83E+09	7.73E+09	7.70E+09	8.07E+09	8.79E+09	7.97E+09	7.24E+09	7.77E+09	7.32E+09	7.44E+09	6.54E+09	7.27E+09	7.33E+09	0	0	0	0	0	0	0	0	0	0	0	0	0	2, 3, 3, 6, 4, 5, 6	1, 13, 2, 3, 4, 5, 6, 8, 13	1, 13, 13, 8, 2, 3, 4, 5, 6	PGM1_MOUSE, SP:Q9D0F9	SP|Q9D0F9|PGM1_MOUSE
Q8K3J1	NDUS8_MOUSE	NADH dehydrogenase [ubiquinone] iron-sulfur protein 8, mitochondrial OS=Mus musculus GN=Ndufs8 PE=1 SV=1 [MASS=24038]	5	13	1.0	9.74E+09	1.24E+10	1.20E+10	1.08E+10	1.15E+10	9.90E+09	1.23E+10	1.03E+10	1.02E+10	1.01E+10	1.08E+10	8.14E+09	6.62E+09	0	0	0	0	0	0	0	0	0	0	0	0	2.80E-42	2, 3, 5, 6	1, 11, 12, 13, 3	1, 12, 13, 14	NDUS8_MOUSE, SP:Q8K3J1	SP|Q8K3J1|NDUS8_MOUSE
Q8BSY0	ASPH_MOUSE	Aspartyl/asparaginyl beta-hydroxylase OS=Mus musculus GN=Asph PE=2 SV=1 [MASS=83042]	1	7	1.0	6.62E+07	4.81E+07	5.62E+07	3.08E+07	6.01E+07	7.61E+07	5.02E+07	6.73E+07	4.65E+07	9.16E+07	4.75E+07	4.92E+07	4.27E+07	0.011	0.015	0.062	0.039	0.027	0.018	0.034	0.034	0.014	0.019	0.048	0.019	0.122	2	5	7	ASPH_MOUSE, SP:Q8BSY0	SP|Q8BSY0|ASPH_MOUSE
P51660	DHB4_MOUSE	Peroxisomal multifunctional enzyme type 2 OS=Mus musculus GN=Hsd17b4 PE=1 SV=3 [MASS=79481]	3	13	1.0	3.70E+08	2.82E+08	2.82E+08	3.67E+08	3.74E+08	2.80E+08	3.30E+08	3.12E+08	2.47E+08	2.98E+08	2.69E+08	2.84E+08	2.71E+08	3.78E-05	5.56E-04	6.97E-05	5.57E-03	1.16E-01	2.14E-04	1.59E-03	1.42E-02	3.98E-02	4.15E-04	3.34E-02	4.44E-03	0.002	3, 4	1, 13, 8	1, 13, 8	DHB4_MOUSE, SP:P51660	SP|P51660|DHB4_MOUSE
Q6PB66	LPPRC_MOUSE	Leucine-rich PPR motif-containing protein, mitochondrial OS=Mus musculus GN=Lrpprc PE=1 SV=2 [MASS=156614]	18	13	1.0	4.25E+09	5.82E+09	5.35E+09	4.36E+09	5.11E+09	5.62E+09	4.94E+09	5.42E+09	5.00E+09	5.05E+09	5.29E+09	4.63E+09	4.03E+09	0	0	0	0	0	0	0	0	0	0	0	0	1.14E-26	2, 3, 4, 4, 5, 4, 6, 5, 6	1, 10, 11, 11, 13, 12, 13, 13, 2, 3, 5, 6, 8, 9	1, 10, 11, 11, 13, 12, 13, 13, 2, 3, 5, 6, 8, 9	LPPRC_MOUSE, SP:Q6PB66	SP|Q6PB66|LPPRC_MOUSE
P68040	GBLP_MOUSE	Guanine nucleotide-binding protein subunit beta-2-like 1 OS=Mus musculus GN=Gnb2l1 PE=1 SV=3 [MASS=35076]	3	3	1.0	3.17E+08	3.60E+08	3.79E+08	2.71E+08	2.48E+08	2.64E+08	2.25E+08	3.93E+08	3.74E+08	3.37E+08	4.16E+08	2.56E+08	3.35E+08	5.04E-04	1.81E-05	4.42E-03	2.21E-01	2.22E-01	1.20E-05	8.40E-02	2.16E-04	1.80E-02	8.93E-07	9.93E-02	4.55E-04	6.14E-01	3, 4	1, 3	1, 3	GBLP_MOUSE, SP:P68040	SP|P68040|GBLP_MOUSE
Q9WUM5	SUCA_MOUSE	Succinyl-CoA ligase [GDP-forming] subunit alpha, mitochondrial OS=Mus musculus GN=Suclg1 PE=1 SV=4 [MASS=36155]	6	13	1.0	1.20E+10	1.17E+10	1.28E+10	1.18E+10	9.33E+09	1.16E+10	1.12E+10	1.03E+10	1.05E+10	1.13E+10	1.04E+10	1.17E+10	9.60E+09	0	0	0	0	0	0	0	0	0	0	0	0	0	3, 3, 4, 3, 5, 4, 4, 5, 4, 7	12, 12, 13, 13, 2, 5, 13, 5	12, 12, 14, 13, 13, 15, 13, 2, 5, 5	SUCA_MOUSE, SP:Q9WUM5	SP|Q9WUM5|SUCA_MOUSE
Q8C129	LCAP_MOUSE	Leucyl-cystinyl aminopeptidase OS=Mus musculus GN=Lnpep PE=1 SV=1 [MASS=117303]	1	2	1.0	5.02E+07	8.62E+07	6.70E+07	7.32E+07	8.54E+07	7.87E+07	6.25E+07	1.11E+08	6.90E+07	7.73E+07	6.50E+07	8.39E+07	6.43E+07	0.025	0.009	0.010	0.018	0.026	0.003	0.022	0.014	0.023	0.008	0.016	0.004	0.078	3	2	2	LCAP_MOUSE, SP:Q8C129	SP|Q8C129|LCAP_MOUSE
P09103	PDIA1_MOUSE	Protein disulfide-isomerase OS=Mus musculus GN=P4hb PE=1 SV=1 [MASS=57143]	7	13	1.0	9.00E+08	1.05E+09	1.08E+09	1.01E+09	1.29E+09	1.09E+09	1.03E+09	1.37E+09	1.34E+09	1.17E+09	1.05E+09	1.05E+09	1.23E+09	4.25E-14	1.06E-18	1.96E-15	1.73E-13	2.24E-10	1.52E-19	1.12E-13	2.52E-19	3.55E-12	1.67E-19	1.16E-11	9.50E-18	2.29E-07	2, 3, 4, 5, 6	1, 12, 13, 2, 6, 7	1, 12, 13, 6, 7, 9	PDIA1_MOUSE, SP:P09103	SP|P09103|PDIA1_MOUSE
P08752	GNAI2_MOUSE	Guanine nucleotide-binding protein G(i), alpha-2 subunit OS=Mus musculus GN=Gnai2 PE=1 SV=4 [MASS=40471]	4	13	1.0	3.48E+08	4.34E+08	4.53E+08	4.27E+08	4.00E+08	4.73E+08	4.00E+08	4.14E+08	4.01E+08	4.31E+08	3.92E+08	3.76E+08	3.98E+08	9.14E-05	3.76E-07	6.88E-06	2.89E-03	9.32E-04	2.00E-07	1.51E-04	5.27E-05	1.13E-03	1.41E-06	2.30E-03	1.68E-04	1.91E-01	3, 4	13, 2, 3, 5	13, 2, 3, 5	GNAI2_MOUSE, SP:P08752	SP|P08752|GNAI2_MOUSE
Q9DCM2	GSTK1_MOUSE	Glutathione S-transferase kappa 1 OS=Mus musculus GN=Gstk1 PE=1 SV=3 [MASS=25704]	10	13	1.0	4.50E+09	5.86E+09	4.78E+09	5.21E+09	4.78E+09	5.29E+09	5.30E+09	4.56E+09	4.04E+09	4.47E+09	4.44E+09	4.06E+09	3.00E+09	0	0	0	0	0	0	0	0	0	0	0	0	3.66E-19	2, 3, 3, 4, 4, 6	1, 10, 11, 13, 4, 11, 6	1, 10, 11, 12, 4, 13, 6	GSTK1_MOUSE, SP:Q9DCM2	SP|Q9DCM2|GSTK1_MOUSE
Q8BMS4	COQ3_MOUSE	Hexaprenyldihydroxybenzoate methyltransferase, mitochondrial OS=Mus musculus GN=Coq3 PE=2 SV=1 [MASS=40956]	3	13	1.0	7.82E+08	6.81E+08	7.76E+08	7.57E+08	8.28E+08	6.68E+08	7.01E+08	6.79E+08	6.10E+08	6.65E+08	6.46E+08	7.37E+08	5.84E+08	4.57E-12	5.25E-13	2.84E-11	4.19E-08	7.84E-06	3.42E-12	7.48E-09	3.37E-08	2.35E-06	1.68E-11	8.54E-08	4.03E-08	8.84E-03	3, 4, 5	1, 13	1, 13	COQ3_MOUSE, SP:Q8BMS4	SP|Q8BMS4|COQ3_MOUSE
P08228	SODC_MOUSE	Superoxide dismutase [Cu-Zn] OS=Mus musculus GN=Sod1 PE=1 SV=2 [MASS=15943]	4	13	1.0	1.46E+10	1.25E+10	1.17E+10	1.59E+10	1.51E+10	1.11E+10	1.49E+10	1.02E+10	1.10E+10	1.21E+10	1.13E+10	1.15E+10	1.08E+10	0	0	0	0	0	0	0	0	0	0	0	0	0	4, 4, 5, 4, 6, 5	1, 13, 11, 13	1, 13, 11, 13	SODC_MOUSE, SP:P08228	SP|P08228|SODC_MOUSE
Q8CHT0	AL4A1_MOUSE	Delta-1-pyrroline-5-carboxylate dehydrogenase, mitochondrial OS=Mus musculus GN=Aldh4a1 PE=1 SV=2 [MASS=61810]	8	13	1.0	3.10E+09	3.04E+09	3.01E+09	3.33E+09	4.07E+09	3.93E+09	4.13E+09	2.92E+09	2.72E+09	2.87E+09	2.67E+09	3.36E+09	3.33E+09	0	0	0	0	6.78E-40	0	0	3.22E-40	3.02E-31	0	2.58E-39	1.24E-38	7.60E-22	3, 4, 5	12, 13, 13, 2, 4, 5	12, 13, 13, 2, 4, 5	AL4A1_MOUSE, SP:Q8CHT0	SP|Q8CHT0|AL4A1_MOUSE
Q9DCV4	RMD1_MOUSE	Regulator of microtubule dynamics protein 1 OS=Mus musculus GN=Fam82b PE=2 SV=2 [MASS=35000]	4	13	1.0	1.09E+09	1.19E+09	1.08E+09	1.03E+09	1.07E+09	1.15E+09	9.12E+08	1.09E+09	9.65E+08	1.06E+09	1.09E+09	8.99E+08	8.23E+08	3.50E-17	2.74E-18	9.00E-16	7.76E-11	6.48E-11	5.48E-22	6.78E-12	1.30E-13	6.84E-11	5.39E-20	9.49E-10	9.89E-14	1.79E-04	2, 3, 4, 5	12, 13, 5, 9	12, 13, 5, 9	RMD1_MOUSE, SP:Q9DCV4	SP|Q9DCV4|RMD1_MOUSE
Q8K370	ACD10_MOUSE	Acyl-CoA dehydrogenase family member 10 OS=Mus musculus GN=Acad10 PE=2 SV=1 [MASS=118978]	7	13	1.0	1.78E+09	2.60E+09	2.31E+09	2.09E+09	2.87E+09	3.04E+09	3.02E+09	2.14E+09	1.92E+09	2.10E+09	2.38E+09	2.05E+09	1.78E+09	6.33E-29	2.83E-40	5.88E-33	7.65E-31	1.53E-30	0	1.68E-41	3.41E-28	1.56E-22	0	1.08E-23	5.02E-28	3.31E-11	2, 3, 4, 5, 6	13, 8, 9, 11	11, 9, 13, 8	ACD10_MOUSE, SP:Q8K370	SP|Q8K370|ACD10_MOUSE
P47802	MTX1_MOUSE	Metaxin-1 OS=Mus musculus GN=Mtx1 PE=1 SV=1 [MASS=35624]	2	10	1.0	1.39E+08	1.29E+08	1.33E+08	1.31E+08	1.44E+08	1.30E+08	1.22E+08	1.33E+08	1.14E+08	1.08E+08	1.16E+08	1.20E+08	1.05E+08	4.83E-01	4.20E-01	8.00E-01	0.003	0.006	3.07E-01	0.002	0.002	0.009	5.86E-01	0.005	0.002	0.034	3	10, 7	10, 7	MTX1_MOUSE, SP:P47802	SP|P47802|MTX1_MOUSE
Q925I1	ATAD3_MOUSE	ATPase family AAA domain-containing protein 3 OS=Mus musculus GN=Atad3 PE=1 SV=1 [MASS=66742]	2	13	1.0	2.18E+08	2.09E+08	1.46E+08	2.04E+08	2.29E+08	1.40E+08	1.70E+08	1.88E+08	1.82E+08	1.72E+08	1.55E+08	1.87E+08	1.58E+08	2.06E-02	2.62E-01	4.31E-02	2.99E-01	0.005	6.84E-03	4.23E-01	1.69E-01	0.001	9.66E-02	6.12E-01	3.37E-01	0.013	2, 3	13, 2	13, 2	ATAD3_MOUSE, SP:Q925I1	SP|Q925I1|ATAD3_MOUSE
P62264	RS14_MOUSE	40S ribosomal protein S14 OS=Mus musculus GN=Rps14 PE=2 SV=3 [MASS=16273]	2	12	1.0	3.15E+08	2.21E+08	2.52E+08	2.30E+08	2.74E+08	1.82E+08	2.61E+08	2.86E+08	2.86E+08	3.03E+08	2.60E+08	2.80E+08	2.65E+08	3.56E-04	2.15E-03	1.47E-02	8.49E-02	0.001	3.67E-03	1.69E-02	3.42E-03	3.55E-02	6.27E-04	3.85E-02	1.08E-02	0.002	2, 3	12	12	RS14_MOUSE, SP:P62264	SP|P62264|RS14_MOUSE
Q62167	DDX3X_MOUSE	ATP-dependent RNA helicase DDX3X OS=Mus musculus GN=Ddx3x PE=1 SV=3 [MASS=73101]	1	1	0.99	7.84E+07	5.60E+07	6.14E+07	5.08E+07	7.55E+07	8.26E+07	4.92E+07	8.03E+07	8.33E+07	7.37E+07	7.54E+07	5.39E+07	7.88E+07	0.007	0.012	0.025	0.024	0.023	0.012	0.036	0.008	0.025	0.005	0.042	0.012	0.057	4	1	1	DDX3X_MOUSE, SP:Q62167	SP|Q62167|DDX3X_MOUSE
P10649	GSTM1_MOUSE	Glutathione S-transferase Mu 1 OS=Mus musculus GN=Gstm1 PE=1 SV=2 [MASS=25970]	4	13	1.0	3.17E+09	3.55E+09	3.48E+09	4.65E+09	4.77E+09	3.87E+09	3.91E+09	2.80E+09	2.78E+09	3.07E+09	2.81E+09	2.73E+09	2.87E+09	0	0	0	0	3.45E-38	0	0	3.56E-40	1.11E-32	0	3.62E-31	2.63E-36	2.38E-18	3, 4, 5	11, 12, 13	11, 12, 13	GSTM1_MOUSE, SP:P10649	SP|P10649|GSTM1_MOUSE
P62855	RS26_MOUSE	40S ribosomal protein S26 OS=Mus musculus GN=Rps26 PE=2 SV=3 [MASS=13015]	1	2	1.0	3.55E+07	7.34E+07	6.54E+07	3.86E+07	5.60E+07	5.87E+07	5.45E+07	7.73E+07	6.19E+07	6.13E+07	6.08E+07	5.18E+07	8.23E+07	0.048	0.010	0.044	0.045	0.046	0.005	0.029	0.018	0.037	0.010	0.044	0.014	0.053	2	2	2	RS26_MOUSE, SP:P62855	SP|P62855|RS26_MOUSE
Q9D8E6	RL4_MOUSE	60S ribosomal protein L4 OS=Mus musculus GN=Rpl4 PE=1 SV=3 [MASS=47153]	2	12	1.0	1.60E+08	2.68E+08	2.61E+08	3.85E+08	2.43E+08	3.36E+08	2.92E+08	3.25E+08	3.46E+08	3.38E+08	3.88E+08	2.48E+08	3.04E+08	2.24E-01	1.70E-03	4.17E-05	2.10E-01	2.90E-02	4.76E-04	6.29E-03	4.24E-04	1.44E-02	2.10E-06	1.03E-01	3.18E-03	9.75E-01	2, 4	12, 7	12, 7	RL4_MOUSE, SP:Q9D8E6	SP|Q9D8E6|RL4_MOUSE
Q9CPR4	RL17_MOUSE	60S ribosomal protein L17 OS=Mus musculus GN=Rpl17 PE=2 SV=3 [MASS=21423]	1	12	0.99	8.05E+07	1.21E+08	1.31E+08	7.41E+07	7.28E+07	1.38E+08	9.04E+07	1.31E+08	1.50E+08	1.32E+08	1.46E+08	8.94E+07	5.92E+07	0.006	4.59E-01	0.009	0.026	0.005	4.60E-01	0.007	5.64E-01	0.004	1.42E-01	0.013	0.002	0.085	3	12	12	RL17_MOUSE, SP:Q9CPR4	SP|Q9CPR4|RL17_MOUSE
P25444	RS2_MOUSE	40S ribosomal protein S2 OS=Mus musculus GN=Rps2 PE=1 SV=3 [MASS=31231]	3	8	1.0	4.55E+08	5.29E+08	5.50E+08	4.43E+08	5.52E+08	5.42E+08	4.66E+08	5.66E+08	6.64E+08	5.80E+08	5.51E+08	5.34E+08	4.65E+08	1.98E-06	1.56E-08	5.29E-06	6.89E-05	2.04E-04	5.53E-09	2.53E-05	7.07E-09	2.70E-05	1.98E-09	3.95E-05	1.82E-06	7.01E-02	2, 3, 5	1, 7, 8	1, 7, 8	RS2_MOUSE, SP:P25444	SP|P25444|RS2_MOUSE
P47963	RL13_MOUSE	60S ribosomal protein L13 OS=Mus musculus GN=Rpl13 PE=2 SV=3 [MASS=24305]	2	13	1.0	2.56E+08	2.08E+08	2.78E+08	2.21E+08	2.50E+08	2.29E+08	1.78E+08	2.54E+08	2.65E+08	2.40E+08	2.43E+08	2.56E+08	2.23E+08	4.00E-03	7.36E-04	2.17E-02	1.62E-01	4.39E-01	7.50E-03	3.10E-01	7.93E-03	2.09E-01	1.47E-03	7.89E-02	3.19E-02	0.004	3, 4	11, 13	11, 13	RL13_MOUSE, SP:P47963	SP|P47963|RL13_MOUSE
P63260	ACTG_MOUSE	Actin, cytoplasmic 2 OS=Mus musculus GN=Actg1 PE=1 SV=1 [MASS=41793]	5	13	1.0	1.13E+10	1.00E+10	9.81E+09	1.08E+10	1.33E+10	9.67E+09	9.78E+09	1.10E+10	1.23E+10	1.25E+10	9.09E+09	1.08E+10	1.16E+10	0	0	0	0	0	0	0	0	0	0	0	0	0	2, 4, 6, 3, 5, 5, 6	1, 12, 12, 13, 3, 12, 13, 4	12, 12, 13, 13, 15, 2, 4	ACTG_MOUSE, SP:P63260	SP|P63260|ACTG_MOUSE,SP|P60710|ACTB_MOUSE
P28654	PGS2_MOUSE	Decorin OS=Mus musculus GN=Dcn PE=2 SV=1 [MASS=39809]	4	10	1.0	5.97E+08	4.90E+08	5.51E+08	6.97E+08	7.32E+08	5.42E+08	6.68E+08	4.83E+08	4.95E+08	5.00E+08	5.69E+08	4.22E+08	4.66E+08	4.81E-09	6.53E-09	2.82E-10	4.49E-07	1.57E-04	1.61E-08	2.03E-08	1.86E-06	1.76E-04	5.11E-10	6.00E-04	1.82E-05	6.13E-02	2, 3, 4	1, 10, 4, 9, 9	1, 10, 4, 9, 9	PGS2_MOUSE, SP:P28654	SP|P28654|PGS2_MOUSE
P07356	ANXA2_MOUSE	Annexin A2 OS=Mus musculus GN=Anxa2 PE=1 SV=2 [MASS=38676]	6	13	1.0	1.69E+09	1.82E+09	1.66E+09	1.59E+09	1.92E+09	1.47E+09	1.72E+09	1.81E+09	2.03E+09	1.95E+09	1.70E+09	1.80E+09	1.67E+09	2.05E-27	5.86E-29	5.83E-25	2.92E-20	3.11E-14	2.43E-34	2.10E-23	9.88E-30	8.60E-21	7.64E-32	1.32E-20	1.51E-23	3.06E-10	2, 3, 4, 4	11, 13, 8	11, 13, 8	ANXA2_MOUSE, SP:P07356	SP|P07356|ANXA2_MOUSE
Q9D8Y1	T126A_MOUSE	Transmembrane protein 126A OS=Mus musculus GN=Tmem126a PE=2 SV=1 [MASS=21539]	1	1	1.0	4.54E+07	7.33E+07	5.17E+07	5.77E+07	5.04E+07	5.63E+07	4.75E+07	6.42E+07	5.92E+07	4.24E+07	5.46E+07	4.21E+07	3.87E+07	0.033	0.022	0.021	0.060	0.056	0.006	0.044	0.024	0.074	0.017	0.069	0.026	0.139	4	1	1	T126A_MOUSE, SP:Q9D8Y1	SP|Q9D8Y1|T126A_MOUSE
P63325	RS10_MOUSE	40S ribosomal protein S10 OS=Mus musculus GN=Rps10 PE=1 SV=1 [MASS=18916]	1	13	0.99	1.10E+08	3.11E+08	2.22E+08	1.26E+08	2.32E+08	1.67E+08	2.00E+08	2.86E+08	2.00E+08	1.82E+08	1.84E+08	1.47E+08	1.07E+08	0.002	8.50E-03	9.90E-01	2.78E-01	0.002	7.03E-05	1.46E-01	8.83E-02	0.001	2.32E-02	0.002	1.15E-02	0.033	4	13	13	RS10_MOUSE, SP:P63325	SP|P63325|RS10_MOUSE
Q64521	GPDM_MOUSE	Glycerol-3-phosphate dehydrogenase, mitochondrial OS=Mus musculus GN=Gpd2 PE=1 SV=2 [MASS=80953]	1	2	0.99	1.90E+07	3.75E+07	3.07E+07	2.46E+07	4.73E+07	3.40E+07	4.03E+07	3.27E+07	3.39E+07	3.30E+07	2.93E+07	2.72E+07	1.26E+07	0.110	0.054	0.085	0.058	0.099	0.032	0.051	0.060	0.094	0.051	0.108	0.075	0.263	2	2	2	GPDM_MOUSE, SP:Q64521	SP|Q64521|GPDM_MOUSE
P62270	RS18_MOUSE	40S ribosomal protein S18 OS=Mus musculus GN=Rps18 PE=2 SV=3 [MASS=17719]	1	9	0.99	1.20E+08	6.11E+07	6.04E+07	8.81E+07	5.98E+07	4.76E+07	6.33E+07	7.48E+07	6.02E+07	6.47E+07	1.01E+08	7.12E+07	8.99E+07	0.001	0.012	0.005	0.039	0.064	0.009	0.021	0.019	0.033	0.001	0.023	0.015	0.046	2	9	9	RS18_MOUSE, SP:P62270	SP|P62270|RS18_MOUSE
P18242	CATD_MOUSE	Cathepsin D OS=Mus musculus GN=Ctsd PE=1 SV=1 [MASS=44954]	3	13	1.0	1.09E+09	1.92E+09	1.53E+09	1.58E+09	1.61E+09	1.91E+09	2.21E+09	1.89E+09	1.84E+09	1.68E+09	1.56E+09	1.57E+09	1.04E+09	2.76E-17	2.55E-26	1.70E-24	6.50E-17	7.74E-19	8.47E-36	8.30E-30	1.27E-26	7.67E-18	6.19E-29	8.65E-18	1.89E-24	5.15E-06	3, 4, 8	12, 13	12, 13	CATD_MOUSE, SP:P18242	SP|P18242|CATD_MOUSE
P62141	PP1B_MOUSE	Serine/threonine-protein phosphatase PP1-beta catalytic subunit OS=Mus musculus GN=Ppp1cb PE=1 SV=3 [MASS=37187]	2	13	1.0	4.03E+08	3.00E+08	2.77E+08	3.59E+08	1.44E+08	2.44E+08	3.26E+08	2.63E+08	3.76E+08	3.35E+08	3.51E+08	3.58E+08	3.21E+08	1.30E-05	8.59E-04	1.18E-04	0.003	3.05E-01	1.24E-04	2.22E-03	1.62E-04	1.64E-02	1.22E-05	4.57E-03	2.58E-02	7.56E-01	3, 4	13, 3	13, 3	PP1B_MOUSE, SP:P62141	SP|P62141|PP1B_MOUSE
P84099	RL19_MOUSE	60S ribosomal protein L19 OS=Mus musculus GN=Rpl19 PE=1 SV=1 [MASS=23466]	1	4	0.99	6.40E+07	4.60E+07	7.06E+07	6.23E+07	3.70E+07	5.47E+07	5.28E+07	5.18E+07	6.83E+07	6.03E+07	5.53E+07	4.76E+07	5.74E+07	0.012	0.007	0.015	0.083	0.051	0.020	0.031	0.014	0.038	0.012	0.051	0.035	0.088	2	4	4	RL19_MOUSE, SP:P84099	SP|P84099|RL19_MOUSE
P08113	ENPL_MOUSE	Endoplasmin OS=Mus musculus GN=Hsp90b1 PE=1 SV=2 [MASS=92475]	2	13	1.0	2.47E+08	4.17E+08	3.91E+08	2.52E+08	3.73E+08	3.29E+08	3.39E+08	4.98E+08	4.52E+08	4.24E+08	4.47E+08	2.68E+08	2.83E+08	5.46E-03	4.99E-06	5.98E-03	5.67E-03	3.36E-02	4.04E-07	1.14E-03	7.95E-06	1.31E-03	1.12E-07	5.38E-02	1.07E-05	0.001	4	12, 13, 9	12, 13, 9	ENPL_MOUSE, SP:P08113	SP|P08113|ENPL_MOUSE
P51150	RAB7A_MOUSE	Ras-related protein Rab-7a OS=Mus musculus GN=Rab7a PE=1 SV=2 [MASS=23490]	1	5	0.88	8.11E+07	6.96E+07	4.72E+07	6.24E+07	6.34E+07	2.79E+07	3.33E+07	5.09E+07	4.15E+07	5.79E+07	5.46E+07	3.34E+07	3.46E+07	0.006	0.023	0.015	0.035	0.122	0.006	0.069	0.043	0.041	0.013	0.086	0.036	0.147	2	5	5	RAB7A_MOUSE, SP:P51150	SP|P51150|RAB7A_MOUSE
Q8BW75	AOFB_MOUSE	Amine oxidase [flavin-containing] B OS=Mus musculus GN=Maob PE=1 SV=3 [MASS=58543]	8	13	1.0	1.99E+09	1.83E+09	1.90E+09	2.21E+09	2.25E+09	2.28E+09	2.23E+09	1.72E+09	1.74E+09	1.94E+09	1.81E+09	1.94E+09	1.44E+09	3.70E-32	4.98E-33	8.09E-35	5.21E-24	7.07E-23	1.68E-34	1.15E-30	1.74E-25	6.93E-21	5.56E-34	2.77E-22	2.02E-22	9.14E-09	2, 3, 4, 5, 6	1, 3, 11, 12, 13	1, 3, 11, 12, 13	AOFB_MOUSE, SP:Q8BW75	SP|Q8BW75|AOFB_MOUSE
P62908	RS3_MOUSE	40S ribosomal protein S3 OS=Mus musculus GN=Rps3 PE=1 SV=1 [MASS=26674]	5	13	1.0	9.16E+08	9.32E+08	9.57E+08	9.52E+08	9.34E+08	8.34E+08	7.55E+08	1.00E+09	9.80E+08	9.95E+08	8.50E+08	9.67E+08	9.85E+08	4.22E-14	9.73E-16	2.68E-14	3.44E-09	1.81E-07	7.70E-17	1.85E-09	1.08E-13	5.35E-10	3.49E-15	1.86E-10	2.24E-12	1.55E-05	2, 3, 4	12, 13, 2, 8	12, 13, 2, 8	RS3_MOUSE, SP:P62908	SP|P62908|RS3_MOUSE
Q9CQN6	TM14C_MOUSE	Transmembrane protein 14C OS=Mus musculus GN=Tmem14c PE=1 SV=1 [MASS=11642]	1	5	1.0	1.10E+08	2.23E+08	1.55E+08	1.67E+08	1.91E+08	1.66E+08	1.62E+08	2.29E+08	1.23E+08	1.35E+08	1.31E+08	1.57E+08	1.22E+08	0.002	1.74E-01	2.32E-01	8.80E-01	0.002	4.57E-03	6.05E-01	0.002	0.004	3.51E-01	0.002	8.38E-02	0.025	6	5	5	TM14C_MOUSE, SP:Q9CQN6	SP|Q9CQN6|TM14C_MOUSE
Q9CPQ3	TOM22_MOUSE	Mitochondrial import receptor subunit TOM22 homolog OS=Mus musculus GN=Tomm22 PE=2 SV=3 [MASS=15537]	1	13	0.99	2.29E+08	1.65E+08	1.78E+08	1.57E+08	2.05E+08	1.45E+08	1.56E+08	1.77E+08	1.71E+08	1.99E+08	1.52E+08	1.89E+08	1.30E+08	1.19E-02	5.39E-02	2.77E-01	5.69E-01	0.004	5.34E-02	6.56E-01	2.47E-01	6.42E-01	1.06E-01	5.78E-01	4.05E-01	0.021	4	13	13	TOM22_MOUSE, SP:Q9CPQ3	SP|Q9CPQ3|TOM22_MOUSE
P63276	RS17_MOUSE	40S ribosomal protein S17 OS=Mus musculus GN=Rps17 PE=1 SV=2 [MASS=15524]	2	2	1.0	3.81E+08	5.94E+08	5.44E+08	3.36E+08	5.70E+08	5.22E+08	4.41E+08	6.53E+08	5.83E+08	6.25E+08	4.17E+08	4.01E+08	4.35E+08	3.18E-04	1.78E-06	1.65E-03	5.02E-04	1.46E-03	6.49E-07	7.99E-04	3.04E-06	9.88E-05	3.01E-05	6.65E-03	1.11E-06	1.78E-01	3, 7	2	2	RS17_MOUSE, SP:P63276	SP|P63276|RS17_MOUSE
P01837	IGKC_MOUSE	Ig kappa chain C region OS=Mus musculus PE=1 SV=1 [MASS=11778]	2	7	1.0	4.28E+07	9.26E+07	9.78E+07	2.47E+08	1.11E+08	2.91E+08	3.03E+08	3.37E+07	6.31E+07	5.19E+08	3.88E+08	1.46E+08	3.42E+08	0.034	0.002	7.69E-03	0.008	9.04E-02	0.002	4.22E-03	0.017	1.14E-04	2.15E-06	0.002	0.073	5.01E-01	2, 3	5, 7	5, 7	IGKC_MOUSE, SP:P01837	SP|P01837|IGKC_MOUSE
Q99JI6	RAP1A_MOUSE	Ras-related protein Rap-1b OS=Mus musculus GN=Rap1b PE=2 SV=2 [MASS=20825]	3	13	1.0	8.24E+08	9.19E+08	7.26E+08	7.27E+08	1.15E+09	9.54E+08	8.58E+08	1.03E+09	9.70E+08	8.35E+08	7.74E+08	9.17E+08	8.29E+08	9.79E-13	7.44E-12	9.08E-11	1.14E-11	7.50E-09	1.13E-16	4.85E-11	1.36E-13	2.79E-08	8.40E-14	6.11E-10	8.03E-13	1.63E-04	3, 3, 5	10, 13, 9, 12	10, 12, 9, 13	RAP1A_MOUSE, SP:P62835	SP|P62835|RAP1A_MOUSE,SP|Q99JI6|RAP1B_MOUSE

P41105	RL28_MOUSE	60S ribosomal protein L28 OS=Mus musculus GN=Rpl28 PE=1 SV=2 [MASS=15733]	1	13	0.99	2.64E+07	2.91E+07
P05201	AATC_MOUSE	Aspartate aminotransferase, cytoplasmic OS=Mus musculus GN=Got1 PE=1 SV=2 [MASS=46231]	18	13	1.0	5.31E+10	6.48E+10
Q9DCT8	CRIP2_MOUSE	Cysteine-rich protein 2 OS=Mus musculus GN=Crip2 PE=1 SV=1 [MASS=22727]	2	13	1.0	6.10E+09	6.77E+09
Q9QUI0	RHOA_MOUSE	Transforming protein RhoA OS=Mus musculus GN=Rhoa PE=1 SV=1 [MASS=21782]	1	2	1.0	1.63E+08	2.46E+08
P62075	TIM13_MOUSE	Mitochondrial import inner membrane translocase subunit Tim13 OS=Mus musculus GN=Timm13 PE=1 SV=1 [MASS=10458]	1	10	1.0	6.92E+08	6.78E+08
Q9ET78	JPH2_MOUSE	Junctophilin-2 OS=Mus musculus GN=Jph2 PE=1 SV=2 [MASS=74692]	4	13	1.0	4.20E+08	4.23E+08
P48036	ANXA5_MOUSE	Annexin A5 OS=Mus musculus GN=Anxa5 PE=1 SV=1 [MASS=35752]	5	12	1.0	8.27E+08	1.40E+09
P24369	PPIB_MOUSE	Peptidyl-prolyl cis-trans isomerase B OS=Mus musculus GN=Ppib PE=2 SV=2 [MASS=23713]	2	12	1.0	3.14E+08	4.22E+08
Q8QZS1	HIBCH_MOUSE	3-hydroxyisobutyryl-CoA hydrolase, mitochondrial OS=Mus musculus GN=Hibch PE=1 SV=1 [MASS=43037]	11	13	1.0	4.85E+09	5.76E+09
P35564	CALX_MOUSE	Calnexin OS=Mus musculus GN=Canx PE=1 SV=1 [MASS=67277]	2	13	1.0	2.53E+08	2.98E+08
Q61171	PRDX2_MOUSE	Peroxiredoxin-2 OS=Mus musculus GN=Prdx2 PE=1 SV=3 [MASS=21778]	4	13	1.0	7.29E+09	5.48E+09
P16546	SPTA2_MOUSE	Spectrin alpha chain, brain OS=Mus musculus GN=Sptan1 PE=1 SV=4 [MASS=284595]	34	13	1.0	9.06E+09	9.92E+09
P62880	GBB2_MOUSE	Guanine nucleotide-binding protein G(I)/G(S)/G(T) subunit beta-2 OS=Mus musculus GN=Gnb2 PE=1 SV=3 [MASS=37331]	1	1	1.0	3.96E+07	2.54E+07
P47857	K6PF_MOUSE	6-phosphofructokinase, muscle type OS=Mus musculus GN=Pfkm PE=1 SV=3 [MASS=85268]	11	13	1.0	7.71E+09	8.06E+09
Q9WUB3	PYGM_MOUSE	Glycogen phosphorylase, muscle form OS=Mus musculus GN=Pygm PE=1 SV=3 [MASS=97286]	25	13	1.0	2.05E+10	2.35E+10
Q9D1G1	RAB1B_MOUSE	Ras-related protein Rab-1B OS=Mus musculus GN=Rab1b PE=1 SV=1 [MASS=22187]	3	6	1.0	3.40E+08	5.14E+08
Q91ZA3	PCCA_MOUSE	Propionyl-CoA carboxylase alpha chain, mitochondrial OS=Mus musculus GN=Pcca PE=2 SV=2 [MASS=79921]	10	13	1.0	2.39E+09	2.24E+09
O35683	NDUA1_MOUSE	NADH dehydrogenase [ubiquinone] 1 alpha subcomplex subunit 1 OS=Mus musculus GN=Ndufa1 PE=2 SV=1 [MASS=8138]	1	13	1.0	2.37E+09	2.74E+09
P31001	DESM_MOUSE	Desmin OS=Mus musculus GN=Des PE=1 SV=3 [MASS=53497]	22	13	1.0	6.42E+10	6.61E+10
P63028	TCTP_MOUSE	Translationally-controlled tumor protein OS=Mus musculus GN=Tpt1 PE=1 SV=1 [MASS=19462]	3	12	1.0	1.99E+09	2.31E+09
Q9QY76	VAPB_MOUSE	Vesicle-associated membrane protein-associated protein B OS=Mus musculus GN=Vapb PE=2 SV=3 [MASS=26946]	2	13	1.0	6.08E+08	6.90E+08
P53026	RL10A_MOUSE	60S ribosomal protein L10a OS=Mus musculus GN=Rpl10a PE=1 SV=3 [MASS=24916]	1	7	0.81	9.80E+07	7.87E+07
P35700	PRDX1_MOUSE	Peroxiredoxin-1 OS=Mus musculus GN=Prdx1 PE=1 SV=1 [MASS=22176]	5	13	1.0	6.21E+09	5.85E+09
Q9DB15	RM12_MOUSE	39S ribosomal protein L12, mitochondrial OS=Mus musculus GN=Mrpl12 PE=1 SV=2 [MASS=21708]	3	13	1.0	1.32E+09	1.29E+09
Q8BH64	EHD2_MOUSE	EH domain-containing protein 2 OS=Mus musculus GN=Ehd2 PE=1 SV=1 [MASS=61174]	9	13	1.0	1.46E+09	2.40E+09
Q6PIE5	AT1A2_MOUSE	Sodium/potassium-transporting ATPase subunit alpha-2 OS=Mus musculus GN=Atp1a2 PE=1 SV=1 [MASS=112217]	1	9	1.0	9.70E+07	8.57E+07
P51410	RL9_MOUSE	60S ribosomal protein L9 OS=Mus musculus GN=Rpl9 PE=2 SV=2 [MASS=21881]	2	12	1.0	2.14E+08	2.43E+08
P59017	B2L13_MOUSE	Bcl-2-like protein 13 OS=Mus musculus GN=Bcl2l13 PE=1 SV=1 [MASS=46669]	3	13	1.0	5.31E+08	7.43E+08
Q9Z2I8	SUCB2_MOUSE	Succinyl-CoA ligase [GDP-forming] subunit beta, mitochondrial OS=Mus musculus GN=Suclg2 PE=2 SV=3 [MASS=46840]	8	13	1.0	1.62E+09	1.94E+09
Q8BMK4	CKAP4_MOUSE	Cytoskeleton-associated protein 4 OS=Mus musculus GN=Ckap4 PE=2 SV=2 [MASS=63692]	1	9	0.99	2.86E+07	1.88E+07
Q99JB2	STML2_MOUSE	Stomatin-like protein 2 OS=Mus musculus GN=Stoml2 PE=1 SV=1 [MASS=38385]	1	1	0.99	6.54E+07	9.33E+07
P29699	FETUA_MOUSE	Alpha-2-HS-glycoprotein OS=Mus musculus GN=Ahsg PE=1 SV=1 [MASS=37325]	5	11	1.0	5.58E+08	5.33E+08
P09411	PGK1_MOUSE	Phosphoglycerate kinase 1 OS=Mus musculus GN=Pgk1 PE=1 SV=4 [MASS=44550]	10	13	1.0	1.41E+10	1.66E+10
Q505D7	OPA3_MOUSE	Optic atrophy 3 protein homolog OS=Mus musculus GN=Opa3 PE=1 SV=1 [MASS=20110]	1	9	1.0	1.39E+08	1.50E+08
Q8VE95	CH082_MOUSE	UPF0598 protein C8orf82 homolog OS=Mus musculus PE=2 SV=1 [MASS=24330]	2	13	1.0	1.73E+08	1.55E+08
Q8VD26	TM143_MOUSE	Transmembrane protein 143 OS=Mus musculus GN=Tmem143 PE=2 SV=1 [MASS=51595]	2	9	1.0	1.43E+08	1.97E+08
P47962	RL5_MOUSE	60S ribosomal protein L5 OS=Mus musculus GN=Rpl5 PE=1 SV=3 [MASS=34400]	2	4	0.97	2.03E+08	2.33E+08
P61014	PPLA_MOUSE	Cardiac phospholamban OS=Mus musculus GN=Pln PE=2 SV=1 [MASS=6094]	1	13	1.0	6.30E+09	2.99E+09
Q8CIB5	FERM2_MOUSE	Fermitin family homolog 2 OS=Mus musculus GN=Fermt2 PE=1 SV=1 [MASS=77800]	4	9	1.0	3.02E+08	4.03E+08
P97351	RS3A_MOUSE	40S ribosomal protein S3a OS=Mus musculus GN=Rps3a PE=1 SV=3 [MASS=29885]	2	6	1.0	1.81E+08	1.46E+08
Q9CQN3	TOM6_MOUSE	Mitochondrial import receptor subunit TOM6 homolog OS=Mus musculus GN=Tomm6 PE=2 SV=1 [MASS=7865]	1	2	0.99	9.01E+07	1.24E+08
P62821	RAB1A_MOUSE	Ras-related protein Rab-1A OS=Mus musculus GN=Rab1A PE=1 SV=3 [MASS=22678]	1	11	1.0	1.12E+08	1.30E+08
Q8CG76	ARK72_MOUSE	Aflatoxin B1 aldehyde reductase member 2 OS=Mus musculus GN=Akr7a2 PE=1 SV=2 [MASS=40598]	2	13	1.0	6.37E+08	5.38E+08
Q8BP67	RL24_MOUSE	60S ribosomal protein L24 OS=Mus musculus GN=Rpl24 PE=2 SV=2 [MASS=17779]	1	4	0.96	1.01E+08	1.05E+08
Q9CZX8	RS19_MOUSE	40S ribosomal protein S19 OS=Mus musculus GN=Rps19 PE=1 SV=3 [MASS=16085]	1	8	0.99	1.29E+08	1.68E+08
Q9CQ92	FIS1_MOUSE	Mitochondrial fission 1 protein OS=Mus musculus GN=Fis1 PE=1 SV=1 [MASS=17009]	2	12	1.0	4.44E+08	4.48E+08
Q8K0E8	FIBB_MOUSE	Fibrinogen beta chain OS=Mus musculus GN=Fgb PE=2 SV=1 [MASS=54752]	6	13	1.0	1.25E+09	2.57E+09
O35855	BCAT2_MOUSE	Branched-chain-amino-acid aminotransferase, mitochondrial OS=Mus musculus GN=Bcat2 PE=2 SV=2 [MASS=44127]	8	13	1.0	3.20E+09	3.76E+09
Q9R0Y5	KAD1_MOUSE	Adenylate kinase isoenzyme 1 OS=Mus musculus GN=Ak1 PE=1 SV=1 [MASS=21539]	7	13	1.0	1.75E+10	1.72E+10
P61027	RAB10_MOUSE	Ras-related protein Rab-10 OS=Mus musculus GN=Rab10 PE=1 SV=1 [MASS=22541]	1	4	1.0	8.78E+07	7.92E+07
P14142	GTR4_MOUSE	Solute carrier family 2, facilitated glucose transporter member 4 OS=Mus musculus GN=Slc2a4 PE=1 SV=2 [MASS=54783]	1	3	0.99	2.18E+08	2.25E+08
P63030	BR44L_MOUSE	Brain protein 44-like protein OS=Mus musculus GN=Brp44l PE=1 SV=1 [MASS=12455]	2	13	1.0	3.35E+09	4.49E+09
O88322	NID2_MOUSE	Nidogen-2 OS=Mus musculus GN=Nid2 PE=1 SV=1 [MASS=154248]	2	7	1.0	1.49E+08	2.30E+08
P14602	HSPB1_MOUSE	Heat shock protein beta-1 OS=Mus musculus GN=Hspb1 PE=1 SV=3 [MASS=23014]	6	13	1.0	4.76E+09	5.03E+09
Q9D880	TIM50_MOUSE	Mitochondrial import inner membrane translocase subunit TIM50 OS=Mus musculus GN=Timm50 PE=1 SV=1 [MASS=39776]	1	13	0.99	1.16E+08	9.46E+07
Q91VT4	CBR4_MOUSE	Carbonyl reductase family member 4 OS=Mus musculus GN=Cbr4 PE=2 SV=2 [MASS=25414]	1	4	1.0	5.40E+07	1.44E+08
Q6ZWY3	RS27L_MOUSE	40S ribosomal protein S27-like protein OS=Mus musculus GN=Rps27l PE=2 SV=3 [MASS=9477]	1	12	0.99	1.77E+08	2.10E+08
P16332	MUTA_MOUSE	Methylmalonyl-CoA mutase, mitochondrial OS=Mus musculus GN=Mut PE=2 SV=1 [MASS=82965]	5	12	1.0	7.60E+08	9.62E+08
P09541	MYL4_MOUSE	Myosin light chain 4 OS=Mus musculus GN=Myl4 PE=2 SV=3 [MASS=21159]	5	12	1.0	3.52E+09	2.12E+09
Q9CQN1	TRAP1_MOUSE	Heat shock protein 75 kDa, mitochondrial OS=Mus musculus GN=Trap1 PE=1 SV=1 [MASS=80208]	9	12	1.0	1.26E+09	1.45E+09
P70296	PEBP1_MOUSE	Phosphatidylethanolamine-binding protein 1 OS=Mus musculus GN=Pebp1 PE=1 SV=3 [MASS=20830]	7	13	1.0	9.34E+09	1.10E+10
Q8C3X2	CC90B_MOUSE	Coiled-coil domain-containing protein 90B, mitochondrial OS=Mus musculus GN=Ccdc90b PE=2 SV=1 [MASS=29597]	2	11	1.0	2.24E+08	3.19E+08
Q99L13	3HIDH_MOUSE	3-hydroxyisobutyrate dehydrogenase, mitochondrial OS=Mus musculus GN=Hibadh PE=2 SV=1 [MASS=35440]	5	13	1.0	2.78E+09	3.24E+09
Q99M87	DNJA3_MOUSE	DnaJ homolog subfamily A member 3, mitochondrial OS=Mus musculus GN=Dnaja3 PE=1 SV=1 [MASS=52443]	1	13	1.0	1.44E+08	2.13E+08
Q99MN9	PCCB_MOUSE	Propionyl-CoA carboxylase beta chain, mitochondrial OS=Mus musculus GN=Pccb PE=1 SV=1 [MASS=58394]	11	13	1.0	3.34E+09	4.10E+09
Q9JI91	ACTN2_MOUSE	Alpha-actinin-2 OS=Mus musculus GN=Actn2 PE=1 SV=1 [MASS=103653]	33	13	1.0	7.98E+10	9.04E+10
Q8BIJ6	SYIM_MOUSE	Isoleucyl-tRNA synthetase, mitochondrial OS=Mus musculus GN=Iars2 PE=2 SV=1 [MASS=112803]	5	13	1.0	9.38E+08	1.16E+09
P53702	CCHL_MOUSE	Cytochrome c-type heme lyase OS=Mus musculus GN=Hccs PE=2 SV=1 [MASS=31007]	1	13	0.99	9.80E+07	7.32E+07
P45376	ALDR_MOUSE	Aldose reductase OS=Mus musculus GN=Akr1b1 PE=1 SV=3 [MASS=35732]	8	13	1.0	6.87E+09	9.40E+09
Q9D1L0	CHCH2_MOUSE	Coiled-coil-helix-coiled-coil-helix domain-containing protein 2, mitochondrial OS=Mus musculus GN=Chchd2 PE=1 SV=1 [MASS=15661]	1	1	0.99	8.34E+06	7.44E+06
Q9WVK4	EHD1_MOUSE	EH domain-containing protein 1 OS=Mus musculus GN=Ehd1 PE=1 SV=1 [MASS=60602]	6	13	1.0	7.12E+08	9.34E+08
P11438	LAMP1_MOUSE	Lysosome-associated membrane glycoprotein 1 OS=Mus musculus GN=Lamp1 PE=1 SV=2 [MASS=43865]	1	3	0.99	9.52E+07	6.82E+07
Q9EQP2	EHD4_MOUSE	EH domain-containing protein 4 OS=Mus musculus GN=Ehd4 PE=1 SV=1 [MASS=61480]	6	13	1.0	1.54E+09	1.46E+09
Q99KR7	PPIF_MOUSE	Peptidyl-prolyl cis-trans isomerase, mitochondrial OS=Mus musculus GN=Ppif PE=2 SV=1 [MASS=21737]	3	13	1.0	1.45E+09	1.36E+09
Q91VN4	CHCH6_MOUSE	Coiled-coil-helix-coiled-coil-helix domain-containing protein 6 OS=Mus musculus GN=Chchd6 PE=2 SV=1 [MASS=29799]	1	3	0.99	2.19E+07	1.95E+07
Q9CRD0	OCAD1_MOUSE	OCIA domain-containing protein 1 OS=Mus musculus GN=Ociad1 PE=1 SV=1 [MASS=27610]	1	6	0.98	1.77E+08	1.67E+08
Q9DBL1	ACDSB_MOUSE	Short/branched chain specific acyl-CoA dehydrogenase, mitochondrial OS=Mus musculus GN=Acadsb PE=1 SV=1 [MASS=47874]	7	13	1.0	1.99E+09	2.10E+09
P61982	1433G_MOUSE	14-3-3 protein gamma OS=Mus musculus GN=Ywhag PE=1 SV=2 [MASS=28302]	3	13	1.0	2.54E+09	2.82E+09
Q9D5T0	ATAD1_MOUSE	ATPase family AAA domain-containing protein 1 OS=Mus musculus GN=Atad1 PE=1 SV=1 [MASS=40744]	3	13	1.0	4.64E+08	5.29E+08
Q9JLZ3	AUHM_MOUSE	Methylglutaconyl-CoA hydratase, mitochondrial OS=Mus musculus GN=Auh PE=2 SV=1 [MASS=33395]	2	11	1.0	4.87E+08	5.92E+08
Q3ULD5	MCCB_MOUSE	Methylcrotonoyl-CoA carboxylase beta chain, mitochondrial OS=Mus musculus GN=Mccc2 PE=2 SV=1 [MASS=61379]	7	13	1.0	1.52E+09	1.40E+09
P35762	CD81_MOUSE	CD81 antigen OS=Mus musculus GN=Cd81 PE=1 SV=1 [MASS=25828]	1	11	0.99	2.21E+08	2.68E+08
Q99LP6	GRPE1_MOUSE	GrpE protein homolog 1, mitochondrial OS=Mus musculus GN=Grpel1 PE=2 SV=1 [MASS=24307]	1	13	1.0	1.54E+08	3.10E+08
Q8CGK3	LONM_MOUSE	Lon protease homolog, mitochondrial OS=Mus musculus GN=Lonp1 PE=1 SV=2 [MASS=105842]	14	13	1.0	3.10E+09	3.84E+09
Q6P3A8	ODBB_MOUSE	2-oxoisovalerate dehydrogenase subunit beta, mitochondrial OS=Mus musculus GN=Bckdhb PE=2 SV=2 [MASS=42880]	2	13	1.0	7.41E+08	8.11E+08
P18760	COF1_MOUSE	Cofilin-1 OS=Mus musculus GN=Cfl1 PE=1 SV=3 [MASS=18559]	3	9	1.0	3.26E+08	3.27E+08
P45591	COF2_MOUSE	Cofilin-2 OS=Mus musculus GN=Cfl2 PE=1 SV=1 [MASS=18709]	3	13	1.0	8.86E+08	1.20E+09
P50171	DHB8_MOUSE	Estradiol 17-beta-dehydrogenase 8 OS=Mus musculus GN=Hsd17b8 PE=1 SV=1 [MASS=26645]	5	13	1.0	8.72E+08	9.76E+08
Q62446	FKBP3_MOUSE	FK506-binding protein 3 OS=Mus musculus GN=Fkbp3 PE=1 SV=2 [MASS=25148]	1	11	0.99	6.95E+07	6.23E+07
Q9CZS1	AL1B1_MOUSE	Aldehyde dehydrogenase X, mitochondrial OS=Mus musculus GN=Aldh1b1 PE=2 SV=1 [MASS=57553]	1	13	0.95	3.75E+08	6.48E+08
P19324	SERPH_MOUSE	Serpin H1 OS=Mus musculus GN=Serpinh1 PE=1 SV=2 [MASS=46589]	3	13	1.0	1.57E+09	1.63E+09
Q8VDD5	MYH9_MOUSE	Myosin-9 OS=Mus musculus GN=Myh9 PE=1 SV=4 [MASS=226371]	8	11	1.0	1.04E+09	1.05E+09
Q9D1I5	MCEE_MOUSE	Methylmalonyl-CoA epimerase, mitochondrial OS=Mus musculus GN=Mcee PE=2 SV=1 [MASS=19017]	2	13	1.0	5.76E+08	6.00E+08
Q9CXJ4	ABCB8_MOUSE	ATP-binding cassette sub-family B member 8, mitochondrial OS=Mus musculus GN=Abcb8 PE=2 SV=1 [MASS=77999]	3	7	1.0	4.22E+08	4.86E+08
Q9DBG6	RPN2_MOUSE	Dolichyl-diphosphooligosaccharide--protein glycosyltransferase subunit 2 OS=Mus musculus GN=Rpn2 PE=2 SV=1 [MASS=69062]	2	3	1.0	1.70E+08	1.65E+08
P61205	ARF2_MOUSE	ADP-ribosylation factor 3 OS=Mus musculus GN=Arf3 PE=2 SV=2 [MASS=20601]	2	13	1.0	6.04E+08	7.32E+08
O35857	TIM44_MOUSE	Mitochondrial import inner membrane translocase subunit TIM44 OS=Mus musculus GN=Timm44 PE=2 SV=1 [MASS=51176]	4	13	1.0	4.59E+08	8.64E+08
Q5U458	DJC11_MOUSE	DnaJ homolog subfamily C member 11 OS=Mus musculus GN=Dnajc11 PE=2 SV=1 [MASS=63247]	1	3	0.99	4.17E+07	5.94E+07
P04919	B3AT_MOUSE	Band 3 anion transport protein OS=Mus musculus GN=Slc4a1 PE=1 SV=1 [MASS=103135]	1	3	0.99	3.53E+07	1.11E+07
P11087	CO1A1_MOUSE	Collagen alpha-1(I) chain OS=Mus musculus GN=Col1a1 PE=1 SV=4 [MASS=138031]	3	13	1.0	1.28E+09	8.51E+08
Q99J39	DCMC_MOUSE	Malonyl-CoA decarboxylase, mitochondrial OS=Mus musculus GN=Mlycd PE=2 SV=1 [MASS=54736]	5	13	1.0	8.71E+08	1.01E+09
P62918	RL8_MOUSE	60S ribosomal protein L8 OS=Mus musculus GN=Rpl8 PE=2 SV=2 [MASS=28024]	1	6	0.92	2.43E+08	1.53E+08
P99024	TBB5_MOUSE	Tubulin beta-5 chain OS=Mus musculus GN=Tubb5 PE=1 SV=1 [MASS=49671]	3	13	1.0	5.27E+08	5.97E+08
P17742	PPIA_MOUSE	Peptidyl-prolyl cis-trans isomerase A OS=Mus musculus GN=Ppia PE=1 SV=2 [MASS=17971]	6	13	1.0	4.21E+09	4.13E+09
P27546	MAP4_MOUSE	Microtubule-associated protein 4 OS=Mus musculus GN=Map4 PE=1 SV=3 [MASS=117428]	6	11	1.0	4.78E+08	5.49E+08
P00416	COX3_MOUSE	Cytochrome c oxidase subunit 3 OS=Mus musculus GN=Mtco3 PE=3 SV=1 [MASS=29937]	1	12	1.0	1.68E+08	4.68E+08
Q68FD5	CLH_MOUSE	Clathrin heavy chain 1 OS=Mus musculus GN=Cltc PE=1 SV=3 [MASS=191555]	17	13	1.0	2.07E+09	3.11E+09
Q60605	MYL6_MOUSE	Myosin light polypeptide 6 OS=Mus musculus GN=Myl6 PE=1 SV=3 [MASS=16930]	3	13	1.0	8.17E+08	8.07E+08
Q80Y14	GLRX5_MOUSE	Glutaredoxin-related protein 5 OS=Mus musculus GN=Glrx5 PE=2 SV=2 [MASS=16292]	1	3	1.0	5.37E+07	8.66E+07
Q91YQ5	RPN1_MOUSE	Dolichyl-diphosphooligosaccharide--protein glycosyltransferase subunit 1 OS=Mus musculus GN=Rpn1 PE=2 SV=1 [MASS=68528]	2	2	1.0	3.94E+07	6.70E+07
P34914	HYES_MOUSE	Epoxide hydrolase 2 OS=Mus musculus GN=Ephx2 PE=1 SV=2 [MASS=62515]	13	13	1.0	7.19E+09	8.50E+09
Q9JK42	PDK2_MOUSE	[Pyruvate dehydrogenase [lipoamide]] kinase isozyme 2, mitochondrial OS=Mus musculus GN=Pdk2 PE=1 SV=1 [MASS=46068]	7	13	1.0	1.14E+09	1.34E+09
P55096	ABCD3_MOUSE	ATP-binding cassette sub-family D member 3 OS=Mus musculus GN=Abcd3 PE=1 SV=1 [MASS=75482]	2	1	1.0	7.73E+07	7.64E+07
Q9R0H0	ACOX1_MOUSE	Peroxisomal acyl-coenzyme A oxidase 1 OS=Mus musculus GN=Acox1 PE=1 SV=4 [MASS=74634]	1	1	0.99	1.41E+08	9.20E+07
P21981	TGM2_MOUSE	Protein-glutamine gamma-glutamyltransferase 2 OS=Mus musculus GN=Tgm2 PE=1 SV=4 [MASS=77061]	5	13	1.0	1.79E+09	2.38E+09
Q9CXI0	COQ5_MOUSE	Ubiquinone biosynthesis methyltransferase COQ5, mitochondrial OS=Mus musculus GN=Coq5 PE=2 SV=2 [MASS=37335]	2	13	1.0	4.23E+08	4.92E+08
Q99MR8	MCCA_MOUSE	Methylcrotonoyl-CoA carboxylase subunit alpha, mitochondrial OS=Mus musculus GN=Mccc1 PE=2 SV=2 [MASS=79343]	16	13	1.0	4.83E+09	4.91E+09
Q9WTQ8	TIM23_MOUSE	Mitochondrial import inner membrane translocase subunit Tim23 OS=Mus musculus GN=Timm23 PE=2 SV=1 [MASS=21978]	1	3	0.98	4.22E+07	7.52E+07
Q8R2G4	NAR3_MOUSE	Ecto-ADP-ribosyltransferase 3 OS=Mus musculus GN=Art3 PE=2 SV=2 [MASS=42015]	5	13	1.0	6.55E+08	6.69E+08
P49813	TMOD1_MOUSE	Tropomodulin-1 OS=Mus musculus GN=Tmod1 PE=2 SV=1 [MASS=40484]	7	13	1.0	2.82E+09	2.81E+09
Q9WVA2	TIM8A_MOUSE	Mitochondrial import inner membrane translocase subunit Tim8 A OS=Mus musculus GN=Timm8a1 PE=1 SV=1 [MASS=11042]	1	6	1.0	4.12E+08	2.41E+08
P19157	GSTP1_MOUSE	Glutathione S-transferase P 1 OS=Mus musculus GN=Gstp1 PE=1 SV=2 [MASS=23609]	1	13	1.0	7.75E+08	9.33E+08
Q8VE22	RT23_MOUSE	28S ribosomal protein S23, mitochondrial OS=Mus musculus GN=Mrps23 PE=2 SV=1 [MASS=20348]	1	11	0.99	8.84E+07	7.85E+07
Q3URD3	SLMAP_MOUSE	Sarcolemmal membrane-associated protein OS=Mus musculus GN=Slmap PE=1 SV=2 [MASS=96933]	3	9	1.0	2.66E+08	3.15E+08
O35206	COFA1_MOUSE	Collagen alpha-1(XV) chain OS=Mus musculus GN=Col15a1 PE=1 SV=2 [MASS=140471]	4	7	1.0	3.73E+08	3.77E+08

Q8VCF0	MAVS_MOUSE	Mitochondrial antiviral-signaling protein OS=Mus musculus GN=Mavs PE=1 SV=1 [MASS=53399]	1	1	[illegible]
Q8BFP9	PDK1_MOUSE	[Pyruvate dehydrogenase [lipoamide]] kinase isozyme 1, mitochondrial OS=Mus musculus GN=Pdk1 PE=2 SV=1 [MASS=48926]	2	2	[illegible]
P53395	ODB2_MOUSE	Lipoamide acyltransferase component of branched-chain alpha-keto acid dehydrogenase complex, mitochondrial OS=Mus musculus GN=Dbt PE=2 SV=1 [MASS=53160]	5	13	[illegible]
Q61598	GDIB_MOUSE	Rab GDP dissociation inhibitor beta OS=Mus musculus GN=Gdi2 PE=1 SV=1 [MASS=50537]	5	13	[illegible]
Q9WTP7	KAD3_MOUSE	GTP:AMP phosphotransferase mitochondrial OS=Mus musculus GN=Ak3 PE=1 SV=3 [MASS=25426]	7	13	[illegible]
P70414	NAC1_MOUSE	Sodium/calcium exchanger 1 OS=Mus musculus GN=Slc8a1 PE=1 SV=1 [MASS=108035]	2	9	[illegible]
Q149B8	CA170_MOUSE	Uncharacterized protein C1orf170 homolog OS=Mus musculus PE=2 SV=1 [MASS=88451]	1	13	[illegible]
P82347	SGCD_MOUSE	Delta-sarcoglycan OS=Mus musculus GN=Sgcd PE=1 SV=1 [MASS=32133]	1	12	[illegible]
O70622	RTN2_MOUSE	Reticulon-2 OS=Mus musculus GN=Rtn2 PE=1 SV=1 [MASS=51346]	1	13	[illegible]
P02468	LAMC1_MOUSE	Laminin subunit gamma-1 OS=Mus musculus GN=Lamc1 PE=1 SV=2 [MASS=177298]	12	13	[illegible]
P47757	CAPZB_MOUSE	F-actin-capping protein subunit beta OS=Mus musculus GN=Capzb PE=1 SV=3 [MASS=31345]	2	3	[illegible]
P06728	APOA4_MOUSE	Apolipoprotein A-IV OS=Mus musculus GN=Apoa4 PE=2 SV=2 [MASS=45029]	1	9	[illegible]
Q00623	APOA1_MOUSE	Apolipoprotein A-I OS=Mus musculus GN=Apoa1 PE=1 SV=1 [MASS=30587]	4	13	[illegible]
Q9JKS4	LDB3_MOUSE	LIM domain-binding protein 3 OS=Mus musculus GN=Ldb3 PE=1 SV=1 [MASS=76431]	18	13	[illegible]
Q9R112	SQRD_MOUSE	Sulfide:quinone oxidoreductase, mitochondrial OS=Mus musculus GN=Sqrdl PE=2 SV=2 [MASS=50340]	1	2	[illegible]
P06909	CFAH_MOUSE	Complement factor H OS=Mus musculus GN=Cfh PE=1 SV=1 [MASS=139082]	1	2	[illegible]
P10107	ANXA1_MOUSE	Annexin A1 OS=Mus musculus GN=Anxa1 PE=1 SV=2 [MASS=38734]	1	4	[illegible]
Q6PHZ2	KCC2D_MOUSE	Calcium/calmodulin-dependent protein kinase type II delta chain OS=Mus musculus GN=Camk2d PE=1 SV=1 [MASS=56369]	2	10	[illegible]
Q9D7B6	ACAD8_MOUSE	Isobutyryl-CoA dehydrogenase, mitochondrial OS=Mus musculus GN=Acad8 PE=2 SV=2 [MASS=45020]	3	12	[illegible]
Q9QYJ0	DNJA2_MOUSE	DnaJ homolog subfamily A member 2 OS=Mus musculus GN=Dnaja2 PE=1 SV=1 [MASS=45745]	1	8	[illegible]
Q91V41	RAB14_MOUSE	Ras-related protein Rab-14 OS=Mus musculus GN=Rab14 PE=1 SV=3 [MASS=23897]	1	11	[illegible]
Q5M8N4	D39U1_MOUSE	Epimerase family protein SDR39U1 OS=Mus musculus GN=Sdr39u1 PE=1 SV=1 [MASS=32995]	3	3	[illegible]
Q8VEK3	HNRPU_MOUSE	Heterogeneous nuclear ribonucleoprotein U OS=Mus musculus GN=Hnrnpu PE=1 SV=1 [MASS=87917]	4	11	[illegible]
P38060	HMGCL_MOUSE	Hydroxymethylglutaryl-CoA lyase, mitochondrial OS=Mus musculus GN=Hmgcl PE=1 SV=1 [MASS=34161]	3	13	[illegible]
P09405	NUCL_MOUSE	Nucleolin OS=Mus musculus GN=Ncl PE=1 SV=2 [MASS=76723]	4	12	[illegible]
O08600	NUCG_MOUSE	Endonuclease G, mitochondrial OS=Mus musculus GN=Endog PE=2 SV=1 [MASS=32190]	3	10	[illegible]
P48678	LMNA_MOUSE	Lamin-A/C OS=Mus musculus GN=Lmna PE=1 SV=2 [MASS=74237]	11	13	[illegible]
P28650	PURA1_MOUSE	Adenylosuccinate synthetase isozyme 1 OS=Mus musculus GN=Adssl1 PE=1 SV=2 [MASS=50254]	6	13	[illegible]
Q924D0	RT4I1_MOUSE	Reticulon-4-interacting protein 1, mitochondrial OS=Mus musculus GN=Rtn4ip1 PE=1 SV=2 [MASS=43371]	3	13	[illegible]
O88531	PPT1_MOUSE	Palmitoyl-protein thioesterase 1 OS=Mus musculus GN=Ppt1 PE=2 SV=2 [MASS=34490]	1	5	[illegible]
Q02819	NUCB1_MOUSE	Nucleobindin-1 OS=Mus musculus GN=Nucb1 PE=1 SV=2 [MASS=53408]	1	2	[illegible]
P62071	RRAS2_MOUSE	Ras-related protein R-Ras2 OS=Mus musculus GN=Rras2 PE=1 SV=1 [MASS=23399]	2	12	[illegible]
Q3V384	LACE1_MOUSE	Lactation elevated protein 1 OS=Mus musculus GN=Lace1 PE=2 SV=1 [MASS=54313]	1	10	[illegible]
Q921H8	THIKA_MOUSE	3-ketoacyl-CoA thiolase A, peroxisomal OS=Mus musculus GN=Acaa1a PE=2 SV=1 [MASS=43953]	2	13	[illegible]
Q9D6S7	RRFM_MOUSE	Ribosome-recycling factor, mitochondrial OS=Mus musculus GN=Mrrf PE=1 SV=1 [MASS=29050]	2	7	[illegible]
Q9WVA4	TAGL2_MOUSE	Transgelin-2 OS=Mus musculus GN=Tagln2 PE=1 SV=4 [MASS=22395]	5	13	[illegible]
Q8BXV2	BRI3B_MOUSE	BRI3-binding protein OS=Mus musculus GN=Bri3bp PE=2 SV=1 [MASS=28263]	2	12	[illegible]
O09117	SYPL1_MOUSE	Synaptophysin-like protein 1 OS=Mus musculus GN=Sypl1 PE=1 SV=2 [MASS=28899]	1	12	[illegible]
Q9WTP6	KAD2_MOUSE	Adenylate kinase 2, mitochondrial OS=Mus musculus GN=Ak2 PE=1 SV=5 [MASS=26469]	3	13	[illegible]
Q02788	CO6A2_MOUSE	Collagen alpha-2(VI) chain OS=Mus musculus GN=Col6a2 PE=2 SV=3 [MASS=110334]	8	13	[illegible]
Q8VCM7	FIBG_MOUSE	Fibrinogen gamma chain OS=Mus musculus GN=Fgg PE=2 SV=1 [MASS=49391]	8	13	[illegible]
P97823	LYPA1_MOUSE	Acyl-protein thioesterase 1 OS=Mus musculus GN=Lypla1 PE=1 SV=1 [MASS=24688]	2	13	[illegible]
Q62351	TFR1_MOUSE	Transferrin receptor protein 1 OS=Mus musculus GN=Tfrc PE=1 SV=1 [MASS=85731]	2	3	[illegible]
Q59J78	MIMIT_MOUSE	Mimitin, mitochondrial OS=Mus musculus GN=Ndufaf2 PE=2 SV=1 [MASS=19628]	1	7	[illegible]
Q8R1I1	QCR9_MOUSE	Cytochrome b-c1 complex subunit 9 OS=Mus musculus GN=Uqcr10 PE=1 SV=1 [MASS=7445]	2	13	[illegible]
P17665	COX7C_MOUSE	Cytochrome c oxidase subunit 7C, mitochondrial OS=Mus musculus GN=Cox7c PE=1 SV=1 [MASS=7333]	1	13	[illegible]
P03930	ATP8_MOUSE	ATP synthase protein 8 OS=Mus musculus GN=Mtatp8 PE=1 SV=1 [MASS=7766]	3	5	[illegible]
P56392	CX7A1_MOUSE	Cytochrome c oxidase polypeptide 7A1, mitochondrial OS=Mus musculus GN=Cox7a1 PE=2 SV=1 [MASS=8986]	3	13	[illegible]
P15116	CADH2_MOUSE	Cadherin-2 OS=Mus musculus GN=Cdh2 PE=1 SV=1 [MASS=99761]	3	13	[illegible]
P00158	CYB_MOUSE	Cytochrome b OS=Mus musculus GN=Mt-Cyb PE=2 SV=1 [MASS=43209]	1	13	[illegible]
Q8R0N6	HOT_MOUSE	Hydroxyacid-oxoacid transhydrogenase, mitochondrial OS=Mus musculus GN=Adhfe1 PE=2 SV=2 [MASS=49937]	1	1	[illegible]
P35279	RAB6A_MOUSE	Ras-related protein Rab-6A OS=Mus musculus GN=Rab6a PE=1 SV=4 [MASS=23590]	1	1	[illegible]
Q9CPX8	QCR10_MOUSE	Cytochrome b-c1 complex subunit 10 OS=Mus musculus GN=Uqcr PE=2 SV=1 [MASS=6539]	1	13	[illegible]
P61979	HNRPK_MOUSE	Heterogeneous nuclear ribonucleoprotein K OS=Mus musculus GN=Hnrnpk PE=1 SV=1 [MASS=50976]	8	13	[illegible]
P62245	RS15A_MOUSE	40S ribosomal protein S15a OS=Mus musculus GN=Rps15a PE=2 SV=2 [MASS=14839]	1	1	[illegible]
Q9CQF4	CF203_MOUSE	Uncharacterized protein C6orf203 homolog OS=Mus musculus PE=1 SV=1 [MASS=27847]	1	6	[illegible]
P14211	CALR_MOUSE	Calreticulin OS=Mus musculus GN=Calr PE=1 SV=1 [MASS=47994]	3	12	[illegible]
Q61595	KTN1_MOUSE	Kinectin OS=Mus musculus GN=Ktn1 PE=1 SV=1 [MASS=152591]	1	2	[illegible]
Q61102	ABCB7_MOUSE	ATP-binding cassette sub-family B member 7, mitochondrial OS=Mus musculus GN=Abcb7 PE=1 SV=2 [MASS=82594]	1	1	[illegible]
P10493	NID1_MOUSE	Nidogen-1 OS=Mus musculus GN=Nid1 PE=1 SV=1 [MASS=136622]	9	13	[illegible]
Q9D517	PLCC_MOUSE	1-acyl-sn-glycerol-3-phosphate acyltransferase gamma OS=Mus musculus GN=Agpat3 PE=1 SV=2 [MASS=43296]	1	7	[illegible]
Q921I1	TRFE_MOUSE	Serotransferrin OS=Mus musculus GN=Tf PE=1 SV=1 [MASS=76723]	14	13	[illegible]
Q61292	LAMB2_MOUSE	Laminin subunit beta-2 OS=Mus musculus GN=Lamb2 PE=2 SV=1 [MASS=196352]	7	13	[illegible]
Q8BJ03	COX15_MOUSE	Cytochrome c oxidase assembly protein COX15 homolog OS=Mus musculus GN=Cox15 PE=2 SV=1 [MASS=45853]	1	1	[illegible]
Q9CZ44	NSF1C_MOUSE	NSFL1 cofactor p47 OS=Mus musculus GN=Nsfl1c PE=1 SV=1 [MASS=40710]	2	7	[illegible]
O09167	RL21_MOUSE	60S ribosomal protein L21 OS=Mus musculus GN=Rpl21 PE=2 SV=3 [MASS=18562]	1	8	[illegible]
Q9CQV1	TIM16_MOUSE	Mitochondrial import inner membrane translocase subunit Tim16 OS=Mus musculus GN=Timm16 PE=2 SV=1 [MASS=13785]	1	2	[illegible]
Q9EQU5	SET_MOUSE	Protein SET OS=Mus musculus GN=Set PE=1 SV=1 [MASS=33378]	1	7	[illegible]
P59999	ARPC4_MOUSE	Actin-related protein 2/3 complex subunit 4 OS=Mus musculus GN=Arpc4 PE=1 SV=3 [MASS=19667]	2	9	[illegible]
Q63918	SDPR_MOUSE	Serum deprivation-response protein OS=Mus musculus GN=Sdpr PE=1 SV=3 [MASS=46764]	4	13	[illegible]
Q9D0S9	HINT2_MOUSE	Histidine triad nucleotide-binding protein 2 OS=Mus musculus GN=Hint2 PE=2 SV=1 [MASS=17320]	3	13	[illegible]
Q78J03	MSRB2_MOUSE	Methionine-R-sulfoxide reductase B2, mitochondrial OS=Mus musculus GN=Msrb2 PE=2 SV=1 [MASS=19157]	1	11	[illegible]
O35143	ATIF1_MOUSE	ATPase inhibitor, mitochondrial OS=Mus musculus GN=Atpif1 PE=2 SV=1 [MASS=12145]	1	12	[illegible]
P29758	OAT_MOUSE	Ornithine aminotransferase, mitochondrial OS=Mus musculus GN=Oat PE=1 SV=1 [MASS=48354]	6	11	[illegible]
Q80U63	MFN2_MOUSE	Mitofusin-2 OS=Mus musculus GN=Mfn2 PE=1 SV=3 [MASS=86187]	1	2	[illegible]
Q9DC61	MPPA_MOUSE	Mitochondrial-processing peptidase subunit alpha OS=Mus musculus GN=Pmpca PE=1 SV=1 [MASS=58279]	1	2	[illegible]
P01867	IGG2B_MOUSE	Ig gamma-2B chain C region OS=Mus musculus GN=Igh-3 PE=1 SV=3 [MASS=44259]	2	7	[illegible]
Q9CXW2	RT22_MOUSE	28S ribosomal protein S22, mitochondrial OS=Mus musculus GN=Mrps22 PE=2 SV=1 [MASS=41192]	1	2	[illegible]
P23927	CRYAB_MOUSE	Alpha-crystallin B chain OS=Mus musculus GN=Cryab PE=1 SV=2 [MASS=20069]	6	13	[illegible]
Q05793	PGBM_MOUSE	Basement membrane-specific heparan sulfate proteoglycan core protein OS=Mus musculus GN=Hspg2 PE=1 SV=1 [MASS=398292]	20	13	[illegible]
Q61001	LAMA5_MOUSE	Laminin subunit alpha-5 OS=Mus musculus GN=Lama5 PE=1 SV=3 [MASS=404009]	1	1	[illegible]
P09055	ITB1_MOUSE	Integrin beta-1 OS=Mus musculus GN=Itgb1 PE=1 SV=1 [MASS=88231]	3	11	[illegible]
Q9EQH3	VPS35_MOUSE	Vacuolar protein sorting-associated protein 35 OS=Mus musculus GN=Vps35 PE=1 SV=1 [MASS=91712]	4	11	[illegible]
P10126	EF1A1_MOUSE	Elongation factor 1-alpha 1 OS=Mus musculus GN=Eef1a1 PE=1 SV=3 [MASS=50113]	4	13	[illegible]
P50580	PA2G4_MOUSE	Proliferation-associated protein 2G4 OS=Mus musculus GN=Pa2g4 PE=1 SV=3 [MASS=43699]	2	6	[illegible]
P35951	LDLR_MOUSE	Low-density lipoprotein receptor OS=Mus musculus GN=Ldlr PE=1 SV=1 [MASS=95134]	1	1	[illegible]
O88569	ROA2_MOUSE	Heterogeneous nuclear ribonucleoproteins A2/B1 OS=Mus musculus GN=Hnrnpa2b1 PE=1 SV=2 [MASS=37402]	6	13	[illegible]
P47754	CAZA2_MOUSE	F-actin-capping protein subunit alpha-2 OS=Mus musculus GN=Capza2 PE=1 SV=3 [MASS=32967]	5	13	[illegible]
Q8BWF0	SSDH_MOUSE	Succinate-semialdehyde dehydrogenase, mitochondrial OS=Mus musculus GN=Aldh5a1 PE=1 SV=1 [MASS=55968]	4	12	[illegible]
Q8CI94	PYGB_MOUSE	Glycogen phosphorylase, brain form OS=Mus musculus GN=Pygb PE=1 SV=3 [MASS=96729]	12	13	[illegible]
O70252	HMOX2_MOUSE	Heme oxygenase 2 OS=Mus musculus GN=Hmox2 PE=2 SV=1 [MASS=35738]	1	1	[illegible]
Q8R0F8	FAHD1_MOUSE	Fumarylacetoacetate hydrolase domain-containing protein 1 OS=Mus musculus GN=Fahd1 PE=2 SV=1 [MASS=25158]	3	13	[illegible]
O88587	COMT_MOUSE	Catechol O-methyltransferase OS=Mus musculus GN=Comt PE=1 SV=1 [MASS=29496]	1	13	[illegible]
Q99LX0	PARK7_MOUSE	Protein DJ-1 OS=Mus musculus GN=Park7 PE=1 SV=1 [MASS=20021]	9	13	[illegible]
A2ASS6	TITIN_MOUSE	Titin OS=Mus musculus GN=Ttn PE=1 SV=1	583	13	[illegible]
P57780	ACTN4_MOUSE	Alpha-actinin-4 OS=Mus musculus GN=Actn4 PE=1 SV=1	3	13	[illegible]
Q60668	HNRPD_MOUSE	Heterogeneous nuclear ribonucleoprotein D0 OS=Mus musculus GN=Hnrnpd PE=1 SV=2	1	3	[illegible]
P13541	MYH3_MOUSE	Myosin-3 OS=Mus musculus GN=Myh3 PE=2 SV=2	1	3	[illegible]
Q3UTJ2	SRBS2_MOUSE	Sorbin and SH3 domain-containing protein 2 OS=Mus musculus GN=Sorbs2 PE=1 SV=2	4	12	[illegible]
Q91Z83	MYH7_MOUSE	Myosin-7 OS=Mus musculus GN=Myh7 PE=1 SV=1	12	13	[illegible]
Q8R081	HNRPL_MOUSE	Heterogeneous nuclear ribonucleoprotein L OS=Mus musculus GN=Hnrnpl PE=1 SV=2	1	1	[illegible]
Q9JJW5	MYOZ2_MOUSE	Myozenin-2 OS=Mus musculus GN=Myoz2 PE=1 SV=1	9	13	[illegible]
P46638	RB11B_MOUSE	Ras-related protein Rab-11B OS=Mus musculus GN=Rab11b PE=1 SV=3	1	3	[illegible]
Q93092	TALDO_MOUSE	Transaldolase OS=Mus musculus GN=Taldo1 PE=1 SV=2	2	13	[illegible]
Q8BGK2	ARHL1_MOUSE	[Protein ADP-ribosylarginine] hydrolase-like protein 1 OS=Mus musculus GN=Adprhl1 PE=2 SV=1	4	12	[illegible]
P26043	RADI_MOUSE	Radixin OS=Mus musculus GN=Rdx PE=1 SV=2	1	9	[illegible]
Q9DBC7	KAP0_MOUSE	cAMP-dependent protein kinase type I-alpha regulatory subunit OS=Mus musculus GN=Prkar1a PE=1 SV=3	3	6	[illegible]
Q99020	ROAA_MOUSE	Heterogeneous nuclear ribonucleoprotein A/B OS=Mus musculus GN=Hnrnpab PE=1 SV=1	2	13	[illegible]
P26883	FKB1A_MOUSE	Peptidyl-prolyl cis-trans isomerase FKBP1A OS=Mus musculus GN=Fkbp1a PE=2 SV=2	1	12	[illegible]
O08553	DPYL2_MOUSE	Dihydropyrimidinase-related protein 2 OS=Mus musculus GN=Dpysl2 PE=1 SV=2	5	13	[illegible]
O88844	IDHC_MOUSE	Isocitrate dehydrogenase [NADP] cytoplasmic OS=Mus musculus GN=Idh1 PE=1 SV=1	3	9	[illegible]
Q8BG05	ROA3_MOUSE	Heterogeneous nuclear ribonucleoprotein A3 OS=Mus musculus GN=Hnrnpa3 PE=1 SV=1	3	13	[illegible]
O08638	MYH11_MOUSE	Myosin-11 OS=Mus musculus GN=Myh11 PE=1 SV=1	2	6	[illegible]
O08709	PRDX6_MOUSE	Peroxiredoxin-6 OS=Mus musculus GN=Prdx6 PE=1 SV=3	6	13	[illegible]
P34884	MIF_MOUSE	Macrophage migration inhibitory factor OS=Mus musculus GN=Mif PE=1 SV=2	1	8	[illegible]
Q9D0E1	HNRPM_MOUSE	Heterogeneous nuclear ribonucleoprotein M OS=Mus musculus GN=Hnrnpm PE=1 SV=3	4	4	[illegible]
P68254	1433T_MOUSE	14-3-3 protein theta OS=Mus musculus GN=Ywhaq PE=1 SV=1	1	13	[illegible]
P23492	PNPH_MOUSE	Purine nucleoside phosphorylase OS=Mus musculus GN=Np PE=1 SV=2	2	4	[illegible]
Q8R3Q6	CCD58_MOUSE	Coiled-coil domain-containing protein 58 OS=Mus musculus GN=Ccdc58 PE=2 SV=1	1	4	[illegible]
Q60675	LAMA2_MOUSE	Laminin subunit alpha-2 OS=Mus musculus GN=Lama2 PE=1 SV=1	15	13	[illegible]
Q9CPU0	LGUL_MOUSE	Lactoylglutathione lyase OS=Mus musculus GN=Glo1 PE=1 SV=3	4	13	[illegible]
Q6IRU2	TPM4_MOUSE	Tropomyosin alpha-4 chain OS=Mus musculus GN=Tpm4 PE=2 SV=3	4	13	[illegible]
Q99PT1	GDIR1_MOUSE	Rho GDP-dissociation inhibitor 1 OS=Mus musculus GN=Arhgdia PE=1 SV=3	4	13	[illegible]
Q8VHX6	FLNC_MOUSE	Filamin-C OS=Mus musculus GN=Flnc PE=1 SV=3	18	12	[illegible]
Q8BG32	PSD11_MOUSE	26S proteasome non-ATPase regulatory subunit 11 OS=Mus musculus GN=Psmd11 PE=1 SV=3	1	5	[illegible]

Q64727	VINC_MOUSE	Vinculin OS=Mus musculus GN=Vcl PE=1 SV=4	21	13	1.0	1.01E+10	1.00E+10	[illegible]
P17182	ENOA_MOUSE	Alpha-enolase OS=Mus musculus GN=Eno1 PE=1 SV=3	8	13	1.0	7.34E+09	8.26E+09	[illegible]
Q8BZF8	PGM5_MOUSE	Phosphoglucomutase-like protein 5 OS=Mus musculus GN=Pgm5 PE=1 SV=2	5	13	1.0	8.06E+08	9.09E+08	[illegible]
Q91X72	HEMO_MOUSE	Hemopexin OS=Mus musculus GN=Hpx PE=1 SV=1	6	13	1.0	1.43E+09	5.16E+09	[illegible]
P14733	LMNB1_MOUSE	Lamin-B1 OS=Mus musculus GN=Lmnb1 PE=1 SV=3	3	13	1.0	4.03E+08	3.34E+08	[illegible]
P50396	GDIA_MOUSE	Rab GDP dissociation inhibitor alpha OS=Mus musculus GN=Gdi1 PE=1 SV=3	3	4	1.0	1.96E+08	2.54E+08	[illegible]
Q7TPR4	ACTN1_MOUSE	Alpha-actinin-1 OS=Mus musculus GN=Actn1 PE=2 SV=1	1	3	1.0	5.32E+07	2.92E+07	[illegible]
P26231	CTNA1_MOUSE	Catenin alpha-1 OS=Mus musculus GN=Ctnna1 PE=1 SV=1	4	11	1.0	5.87E+08	6.85E+08	[illegible]
P28665	MUG1_MOUSE	Murinoglobulin-1 OS=Mus musculus GN=Mug1 PE=1 SV=3	5	13	1.0	6.54E+08	5.10E+08	[illegible]
O55131	SEPT7_MOUSE	Septin-7 OS=Mus musculus GN=Sept7 PE=1 SV=1	2	10	1.0	1.49E+08	2.43E+08	[illegible]
Q8CI51	PDLI5_MOUSE	PDZ and LIM domain protein 5 OS=Mus musculus GN=Pdlim5 PE=1 SV=3	7	13	1.0	5.98E+09	8.06E+09	[illegible]
Q7TMK9	HNRPQ_MOUSE	Heterogeneous nuclear ribonucleoprotein Q OS=Mus musculus GN=Syncrip PE=1 SV=2	1	2	1.0	8.56E+07	8.47E+07	[illegible]
Q9R0P5	DEST_MOUSE	Destrin OS=Mus musculus GN=Dstn PE=1 SV=3	1	11	1.0	1.54E+08	1.20E+08	[illegible]
A2AAJ9	OBSCN_MOUSE	Obscurin OS=Mus musculus GN=Obscn PE=2 SV=2	13	13	1.0	1.10E+09	1.41E+09	[illegible]
O08529	CAN2_MOUSE	Calpain-2 catalytic subunit OS=Mus musculus GN=Capn2 PE=2 SV=4	1	2	0.99	2.22E+07	1.49E+07	[illegible]
Q8BTM8	FLNA_MOUSE	Filamin-A OS=Mus musculus GN=Flna PE=1 SV=4	9	13	1.0	8.28E+09	7.79E+09	[illegible]
P10518	HEM2_MOUSE	Delta-aminolevulinic acid dehydratase OS=Mus musculus GN=Alad PE=1 SV=1	2	1	1.0	1.57E+08	1.19E+08	[illegible]
P07309	TTHY_MOUSE	Transthyretin OS=Mus musculus GN=Ttr PE=1 SV=1	3	12	1.0	5.61E+08	3.46E+08	[illegible]
Q6ZWX6	IF2A_MOUSE	Eukaryotic translation initiation factor 2 subunit 1 OS=Mus musculus GN=Eif2s1 PE=1 SV=3	2	1	1.0	1.15E+08	1.39E+08	[illegible]
P16045	LEG1_MOUSE	Galectin-1 OS=Mus musculus GN=Lgals1 PE=1 SV=3	5	13	1.0	2.41E+09	1.83E+09	[illegible]
Q62234	MYOM1_MOUSE	Myomesin-1 OS=Mus musculus GN=Myom1 PE=1 SV=2	43	13	1.0	1.93E+10	2.32E+10	[illegible]
Q9DBP5	KCY_MOUSE	UMP-CMP kinase OS=Mus musculus GN=Cmpk1 PE=1 SV=1	1	2	0.99	1.73E+08	1.30E+08	[illegible]
P21619	LMNB2_MOUSE	Lamin-B2 OS=Mus musculus GN=Lmnb2 PE=1 SV=2	1	1	0.99	1.22E+07	8.81E+06	[illegible]
Q62261	SPTB2_MOUSE	Spectrin beta chain, brain 1 OS=Mus musculus GN=Sptbn1 PE=1 SV=2	35	13	1.0	6.27E+09	7.22E+09	[illegible]
Q9WTI7	MYO1C_MOUSE	Myosin-Ic OS=Mus musculus GN=Myo1c PE=1 SV=2	1	1	0.99	3.27E+07	3.70E+07	[illegible]
P99026	PSB4_MOUSE	Proteasome subunit beta type-4 OS=Mus musculus GN=Psmb4 PE=1 SV=1	2	6	1.0	4.16E+08	3.77E+08	[illegible]
P97313	PRKDC_MOUSE	DNA-dependent protein kinase catalytic subunit OS=Mus musculus GN=Prkdc PE=1 SV=2	1	9	0.86	1.48E+09	1.63E+09	[illegible]
Q04592	PCSK5_MOUSE	Proprotein convertase subtilisin/kexin type 5 OS=Mus musculus GN=Pcsk5 PE=1 SV=2	1	10	0.99	3.23E+08	2.64E+08	[illegible]
Q61838	A2M_MOUSE	Alpha-2-macroglobulin OS=Mus musculus GN=A2m PE=1 SV=2	4	13	1.0	3.32E+08	5.51E+08	[illegible]
Q6P069	SORCN_MOUSE	Sorcin OS=Mus musculus GN=Sri PE=2 SV=1	1	4	0.98	4.68E+07	6.20E+07	[illegible]
Q99JY9	ARP3_MOUSE	Actin-related protein 3 OS=Mus musculus GN=Actr3 PE=1 SV=3	2	1	1.0	1.24E+08	1.14E+08	[illegible]
Q9JLV1	BAG3_MOUSE	BAG family molecular chaperone regulator 3 OS=Mus musculus GN=Bag3 PE=1 SV=1	7	13	1.0	1.45E+09	1.45E+09	[illegible]
Q9JM76	ARPC3_MOUSE	Actin-related protein 2/3 complex subunit 3 OS=Mus musculus GN=Arpc3 PE=1 SV=3	1	13	0.99	1.44E+08	1.88E+08	[illegible]
Q9R062	GLYG_MOUSE	Glycogenin-1 OS=Mus musculus GN=Gyg1 PE=2 SV=3	3	9	1.0	3.83E+08	4.42E+08	[illegible]
P26039	TLN1_MOUSE	Talin-1 OS=Mus musculus GN=Tln1 PE=1 SV=1	10	11	1.0	1.12E+09	1.16E+09	[illegible]
P62962	PROF1_MOUSE	Profilin-1 OS=Mus musculus GN=Pfn1 PE=1 SV=2	3	13	1.0	2.28E+09	3.26E+09	[illegible]
P32921	SYWC_MOUSE	Tryptophanyl-tRNA synthetase, cytoplasmic OS=Mus musculus GN=Wars PE=1 SV=2	2	10	1.0	1.20E+08	2.01E+08	[illegible]
Q9D1D4	TMEDA_MOUSE	Transmembrane emp24 domain-containing protein 10 OS=Mus musculus GN=Tmed10 PE=2 SV=1	1	12	0.99	9.46E+07	1.02E+08	[illegible]
Q9EQK5	MVP_MOUSE	Major vault protein OS=Mus musculus GN=Mvp PE=2 SV=3	2	2	1.0	1.11E+08	1.13E+08	[illegible]
O55222	ILK_MOUSE	Integrin-linked protein kinase OS=Mus musculus GN=Ilk PE=1 SV=2	1	2	0.99	6.37E+07	1.05E+08	[illegible]
Q76MZ3	2AAA_MOUSE	Serine/threonine-protein phosphatase 2A 65 kDa regulatory subunit A alpha isoform OS=Mus musculus GN=Ppp2r1a PE=1 SV=3	4	8	1.0	1.03E+09	1.21E+09	[illegible]
Q9WVJ2	PSD13_MOUSE	26S proteasome non-ATPase regulatory subunit 13 OS=Mus musculus GN=Psmd13 PE=1 SV=1	3	4	1.0	1.83E+08	2.39E+08	[illegible]
Q8K4G5	ABLM1_MOUSE	Actin-binding LIM protein 1 OS=Mus musculus GN=Ablim1 PE=1 SV=1	7	13	1.0	1.16E+09	1.41E+09	[illegible]
P37804	TAGL_MOUSE	Transgelin OS=Mus musculus GN=Tagln PE=1 SV=3	3	10	1.0	4.73E+08	4.71E+08	[illegible]
P13020	GELS_MOUSE	Gelsolin OS=Mus musculus GN=Gsn PE=1 SV=3	2	11	1.0	1.85E+08	1.66E+08	[illegible]
P17563	SBP1_MOUSE	Selenium-binding protein 1 OS=Mus musculus GN=Selenbp1 PE=1 SV=2	2	13	1.0	1.77E+08	1.26E+08	[illegible]
Q01149	CO1A2_MOUSE	Collagen alpha-2(I) chain OS=Mus musculus GN=Col1a2 PE=2 SV=2	1	13	0.99	2.98E+08	1.88E+08	[illegible]
Q9R0P9	UCHL1_MOUSE	Ubiquitin carboxyl-terminal hydrolase isozyme L1 OS=Mus musculus GN=Uchl1 PE=1 SV=1	2	5	1.0	1.76E+08	1.62E+08	[illegible]
Q9CZJ2	HS12B_MOUSE	Heat shock 70 kDa protein 12B OS=Mus musculus GN=Hspa12b PE=1 SV=1	1	1	0.92	4.10E+07	5.16E+07	[illegible]
O70373	XIRP1_MOUSE	Xin actin-binding repeat-containing protein 1 OS=Mus musculus GN=Xirp1 PE=1 SV=2	2	12	1.0	6.93E+07	9.98E+07	[illegible]
B2RRE4	Z518B_MOUSE	Zinc finger protein 518B OS=Mus musculus GN=Znf518b PE=2 SV=1	1	4	0.94	8.12E+08	4.42E+08	[illegible]
P61971	NTF2_MOUSE	Nuclear transport factor 2 OS=Mus musculus GN=Nutf2 PE=2 SV=1	1	8	0.94	1.90E+08	2.05E+08	[illegible]
Q9JHU2	PALMD_MOUSE	Palmdelphin OS=Mus musculus GN=Palmd PE=1 SV=1	1	7	0.99	4.02E+07	6.51E+07	[illegible]
P08122	CO4A2_MOUSE	Collagen alpha-2(IV) chain OS=Mus musculus GN=Col4a2 PE=2 SV=4	5	13	1.0	1.43E+09	1.35E+09	[illegible]
Q9JJU8	SH3L1_MOUSE	SH3 domain-binding glutamic acid-rich-like protein OS=Mus musculus GN=Sh3bgrl PE=2 SV=1	1	5	0.99	8.27E+07	7.14E+07	[illegible]
P35385	HSPB7_MOUSE	Heat shock protein beta-7 OS=Mus musculus GN=Hspb7 PE=1 SV=2	3	13	1.0	7.65E+08	9.29E+08	[illegible]
P40124	CAP1_MOUSE	Adenylyl cyclase-associated protein 1 OS=Mus musculus GN=Cap1 PE=1 SV=3	2	13	1.0	3.49E+08	3.32E+08	[illegible]
Q8R1S0	COQ6_MOUSE	Ubiquinone biosynthesis monooxygenase COQ6 OS=Mus musculus GN=Coq6 PE=2 SV=2	3	7	1.0	5.56E+08	6.53E+08	[illegible]
Q9DBJ1	PGAM1_MOUSE	Phosphoglycerate mutase 1 OS=Mus musculus GN=Pgam1 PE=1 SV=3	5	13	1.0	1.36E+09	1.40E+09	[illegible]
P97447	FHL1_MOUSE	Four and a half LIM domains protein 1 OS=Mus musculus GN=Fhl1 PE=2 SV=3	2	12	1.0	3.12E+08	2.72E+08	[illegible]
Q9CQV8	1433B_MOUSE	14-3-3 protein beta/alpha OS=Mus musculus GN=Ywhab PE=1 SV=3	5	13	1.0	1.07E+09	1.04E+09	[illegible]
Q60994	ADIPO_MOUSE	Adiponectin OS=Mus musculus GN=Adipoq PE=1 SV=1	3	13	1.0	2.08E+09	1.94E+09	[illegible]
P06745	G6PI_MOUSE	Glucose-6-phosphate isomerase OS=Mus musculus GN=Gpi PE=1 SV=4	14	13	1.0	1.79E+10	2.14E+10	[illegible]
Q8CHP8	PGP_MOUSE	Phosphoglycolate phosphatase OS=Mus musculus GN=Pgp PE=2 SV=1	1	7	0.96	9.42E+07	6.77E+07	[illegible]
Q04857	CO6A1_MOUSE	Collagen alpha-1(VI) chain OS=Mus musculus GN=Col6a1 PE=2 SV=1	7	13	1.0	2.60E+09	2.73E+09	[illegible]
Q60692	PSB6_MOUSE	Proteasome subunit beta type-6 OS=Mus musculus GN=Psmb6 PE=1 SV=3	1	12	0.97	1.46E+08	1.04E+08	[illegible]
Q61316	HSP74_MOUSE	Heat shock 70 kDa protein 4 OS=Mus musculus GN=Hspa4 PE=1 SV=1	9	13	1.0	9.66E+08	1.11E+09	[illegible]
Q9QUM9	PSA6_MOUSE	Proteasome subunit alpha type-6 OS=Mus musculus GN=Psma6 PE=1 SV=1	1	11	1.0	2.46E+08	2.65E+08	[illegible]
Q9Z1Q5	CLIC1_MOUSE	Chloride intracellular channel protein 1 OS=Mus musculus GN=Clic1 PE=1 SV=3	2	9	1.0	1.23E+08	1.40E+08	[illegible]
Q9ES97	RTN3_MOUSE	Reticulon-3 OS=Mus musculus GN=Rtn3 PE=1 SV=2	1	13	0.99	6.42E+08	5.25E+08	[illegible]
O70400	PDLI1_MOUSE	PDZ and LIM domain protein 1 OS=Mus musculus GN=Pdlim1 PE=2 SV=4	2	6	1.0	2.13E+08	2.33E+08	[illegible]
Q02013	AQP1_MOUSE	Aquaporin-1 OS=Mus musculus GN=Aqp1 PE=1 SV=3	3	7	1.0	9.29E+08	6.46E+08	[illegible]
P60843	IF4A1_MOUSE	Eukaryotic initiation factor 4A-I OS=Mus musculus GN=Eif4a1 PE=2 SV=1	2	13	1.0	4.96E+08	5.38E+08	[illegible]
Q02257	PLAK_MOUSE	Junction plakoglobin OS=Mus musculus GN=Jup PE=2 SV=3	13	13	1.0	1.83E+09	2.23E+09	[illegible]
Q61033	LAP2A_MOUSE	Lamina-associated polypeptide 2, isoforms alpha/zeta OS=Mus musculus GN=Tmpo PE=1 SV=3	1	1	1.0	5.67E+07	4.43E+07	[illegible]
Q9CXW4	RL11_MOUSE	60S ribosomal protein L11 OS=Mus musculus GN=Rpl11 PE=1 SV=4	2	13	1.0	3.86E+08	3.76E+08	[illegible]
Q9WUM4	COR1C_MOUSE	Coronin-1C OS=Mus musculus GN=Coro1c PE=1 SV=2	1	1	0.99	6.25E+07	9.21E+07	[illegible]
P62774	MTPN_MOUSE	Myotrophin OS=Mus musculus GN=Mtpn PE=2 SV=2	1	4	0.99	8.36E+07	6.14E+07	[illegible]
P68510	1433F_MOUSE	14-3-3 protein eta OS=Mus musculus GN=Ywhah PE=1 SV=2	2	13	1.0	2.53E+08	1.86E+08	[illegible]
P07759	SPA3K_MOUSE	Serine protease inhibitor A3K OS=Mus musculus GN=Serpina3k PE=1 SV=2	3	13	1.0	1.44E+09	1.17E+09	[illegible]
P29341	PABP1_MOUSE	Polyadenylate-binding protein 1 OS=Mus musculus GN=Pabpc1 PE=1 SV=1	2	5	1.0	2.14E+08	2.54E+08	[illegible]
Q921F2	TADBP_MOUSE	TAR DNA-binding protein 43 OS=Mus musculus GN=Tardbp PE=1 SV=1	2	4	1.0	1.08E+08	9.58E+07	[illegible]
P24527	LKHA4_MOUSE	Leukotriene A-4 hydrolase OS=Mus musculus GN=Lta4h PE=1 SV=3	1	7	0.99	7.82E+07	1.72E+08	[illegible]
P09528	FRIH_MOUSE	Ferritin heavy chain OS=Mus musculus GN=Fth1 PE=1 SV=2	6	13	1.0	3.88E+09	4.15E+09	[illegible]
Q60864	STIP1_MOUSE	Stress-induced-phosphoprotein 1 OS=Mus musculus GN=Stip1 PE=1 SV=1	8	13	1.0	1.98E+09	2.39E+09	[illegible]
P83940	ELOC_MOUSE	Transcription elongation factor B polypeptide 1 OS=Mus musculus GN=Tceb1 PE=1 SV=1	1	4	0.99	1.03E+08	4.47E+07	[illegible]
P51885	LUM_MOUSE	Lumican OS=Mus musculus GN=Lum PE=1 SV=2	4	12	1.0	1.24E+09	1.45E+09	[illegible]
P61164	ACTZ_MOUSE	Alpha-centractin OS=Mus musculus GN=Actr1a PE=2 SV=1	1	7	1.0	1.73E+08	2.45E+08	[illegible]
Q8R5C5	ACTY_MOUSE	Beta-centractin OS=Mus musculus GN=Actr1b PE=1 SV=1	1	7	1.0	1.05E+08	1.90E+08	[illegible]
Q05816	FABP5_MOUSE	Fatty acid-binding protein, epidermal OS=Mus musculus GN=Fabp5 PE=1 SV=3	1	4	0.85	2.17E+08	1.77E+08	[illegible]
Q04447	KCRB_MOUSE	Creatine kinase B-type OS=Mus musculus GN=Ckb PE=1 SV=1	2	13	1.0	4.28E+08	5.42E+08	[illegible]
P40630	TFAM_MOUSE	Transcription factor A, mitochondrial OS=Mus musculus GN=Tfam PE=2 SV=2	2	10	1.0	1.63E+08	1.38E+08	[illegible]
Q9WVE8	PACN2_MOUSE	Protein kinase C and casein kinase substrate in neurons protein 2 OS=Mus musculus GN=Pacsin2 PE=2 SV=1	2	6	1.0	1.77E+08	1.24E+08	[illegible]
O55023	IMPA1_MOUSE	Inositol monophosphatase OS=Mus musculus GN=Impa1 PE=2 SV=1	1	7	0.88	6.11E+09	4.89E+09	[illegible]
O35887	CALU_MOUSE	Calumenin OS=Mus musculus GN=Calu PE=1 SV=1	1	8	1.0	8.67E+07	6.12E+07	[illegible]
Q9D020	5NT3_MOUSE	Cytosolic 5'-nucleotidase 3 OS=Mus musculus GN=Nt5c3 PE=1 SV=4	4	13	1.0	3.22E+08	3.89E+08	[illegible]
Q9CVB6	ARPC2_MOUSE	Actin-related protein 2/3 complex subunit 2 OS=Mus musculus GN=Arpc2 PE=1 SV=3	2	3	1.0	3.16E+08	2.93E+08	[illegible]
P00920	CAH2_MOUSE	Carbonic anhydrase 2 OS=Mus musculus GN=Ca2 PE=1 SV=4	4	13	1.0	1.08E+09	7.71E+08	[illegible]
Q8VDQ1	PTGR2_MOUSE	Prostaglandin reductase 2 OS=Mus musculus GN=Ptgr2 PE=1 SV=2	4	12	1.0	7.07E+08	8.23E+08	[illegible]
P80314	TCPB_MOUSE	T-complex protein 1 subunit beta OS=Mus musculus GN=Cct2 PE=1 SV=4	1	1	1.0	4.46E+07	9.71E+07	[illegible]
Q9CPY7	AMPL_MOUSE	Cytosol aminopeptidase OS=Mus musculus GN=Lap3 PE=1 SV=3	4	12	1.0	4.19E+08	4.40E+08	[illegible]
P29391	FRIL1_MOUSE	Ferritin light chain 1 OS=Mus musculus GN=Ftl1 PE=1 SV=2	2	12	1.0	3.09E+08	4.25E+08	[illegible]
P97443	SMYD1_MOUSE	SET and MYND domain-containing protein 1 OS=Mus musculus GN=Smyd1 PE=1 SV=2	7	13	1.0	3.30E+09	3.78E+09	[illegible]
P82350	SGCA_MOUSE	Alpha-sarcoglycan OS=Mus musculus GN=Sgca PE=1 SV=1	1	7	0.99	1.67E+08	2.39E+08	[illegible]
O88342	WDR1_MOUSE	WD repeat-containing protein 1 OS=Mus musculus GN=Wdr1 PE=1 SV=3	1	13	0.99	2.13E+08	1.87E+08	[illegible]
Q02053	UBA1_MOUSE	Ubiquitin-like modifier-activating enzyme 1 OS=Mus musculus GN=Uba1 PE=1 SV=1	11	13	1.0	2.27E+09	2.69E+09	[illegible]
Q8R4N0	CLYBL_MOUSE	Citrate lyase subunit beta-like protein, mitochondrial OS=Mus musculus GN=Clybl PE=2 SV=2	5	13	1.0	1.37E+09	1.52E+09	[illegible]
Q8K310	MATR3_MOUSE	Matrin-3 OS=Mus musculus GN=Matr3 PE=1 SV=1	2	2	1.0	1.41E+08	1.74E+08	[illegible]
Q62417	SRBS1_MOUSE	Sorbin and SH3 domain-containing protein 1 OS=Mus musculus GN=Sorbs1 PE=1 SV=2	4	13	1.0	3.40E+08	5.56E+08	[illegible]
P97371	PSME1_MOUSE	Proteasome activator complex subunit 1 OS=Mus musculus GN=Psme1 PE=2 SV=2	6	13	1.0	9.19E+08	1.02E+09	[illegible]
Q8BGY2	IF5A1_MOUSE	Eukaryotic translation initiation factor 5A-2 OS=Mus musculus GN=Eif5a2 PE=2 SV=3	1	13	0.99	2.80E+08	6.95E+08	[illegible]
P07901	HS90A_MOUSE	Heat shock protein HSP 90-alpha OS=Mus musculus GN=Hsp90aa1 PE=1 SV=4	2	12	1.0	2.93E+08	2.61E+08	[illegible]
Q9Z2U1	PSA5_MOUSE	Proteasome subunit alpha type-5 OS=Mus musculus GN=Psma5 PE=1 SV=1	3	13	1.0	9.42E+08	8.99E+08	[illegible]
Q7TMM9	TBB2A_MOUSE	Tubulin beta-2A chain OS=Mus musculus GN=Tubb2a PE=1 SV=1	1	12	1.0	1.63E+08	9.83E+07	[illegible]
P05125	ANF_MOUSE	Atrial natriuretic factor OS=Mus musculus GN=Nppa PE=2 SV=1	2	6	1.0	3.19E+08	2.38E+08	[illegible]
P02469	LAMB1_MOUSE	Laminin subunit beta-1 OS=Mus musculus GN=Lamb1-1 PE=1 SV=2	15	13	1.0	3.69E+09	3.85E+09	[illegible]
Q9D7X3	DUS3_MOUSE	Dual specificity protein phosphatase 3 OS=Mus musculus GN=Dusp3 PE=1 SV=1	1	2	0.99	2.52E+06	1.74E+06	[illegible]
Q9QYB1	CLIC4_MOUSE	Chloride intracellular channel protein 4 OS=Mus musculus GN=Clic4 PE=1 SV=3	2	8	1.0	1.34E+08	1.54E+08	[illegible]
Q99JY3	GIMA4_MOUSE	GTPase IMAP family member 4 OS=Mus musculus GN=Gimap4 PE=2 SV=1	1	7	0.99	7.74E+07	1.35E+08	[illegible]
Q01730	RSU1_MOUSE	Ras suppressor protein 1 OS=Mus musculus GN=Rsu1 PE=2 SV=3	2	13	0.99	4.33E+08	4.68E+08	[illegible]
Q9Z2X1	HNRPF_MOUSE	Heterogeneous nuclear ribonucleoprotein F OS=Mus musculus GN=Hnrnpf PE=1 SV=3	1	7	1.0	4.44E+07	3.12E+07	[illegible]
Q60854	SPB6_MOUSE	Serpin B6 OS=Mus musculus GN=Serpinb6 PE=2 SV=1	3	10	1.0	4.57E+08	5.36E+08	[illegible]
Q9CPV4	GLOD4_MOUSE	Glyoxalase domain-containing protein 4 OS=Mus musculus GN=Glod4 PE=2 SV=1	2	8	1.0	2.36E+08	2.37E+08	[illegible]
P63330	PP2AA_MOUSE	Serine/threonine-protein phosphatase 2A catalytic subunit alpha isoform OS=Mus musculus GN=Ppp2ca PE=1 SV=1	1	13	1.0	2.62E+08	3.26E+08	[illegible]
Q64105	SPRE_MOUSE	Sepiapterin reductase OS=Mus musculus GN=Spr PE=1 SV=1	2	11	1.0	2.90E+08	5.79E+08	[illegible]

Q9JHW2	NIT2_MOUSE	Nitrilase homolog 2 OS=Mus musculus GN=Nit2 PE=1 SV=1	4	12	1.0	3.30E+08	5.53E+08	[illegible]
P01869	IGH1M_MOUSE	Ig gamma-1 chain C region, membrane-bound form OS=Mus musculus GN=Ighg1 PE=1 SV=2	2	9	1.0	1.94E+08	2.95E+08	[illegible]
P56565	S10A1_MOUSE	Protein S100-A1 OS=Mus musculus GN=S100a1 PE=1 SV=2	2	13	1.0	6.75E+08	1.35E+09	[illegible]
Q91ZJ5	UGPA_MOUSE	UTP--glucose-1-phosphate uridylyltransferase OS=Mus musculus GN=Ugp2 PE=2 SV=3	8	13	1.0	1.38E+09	2.08E+09	[illegible]
P97429	ANXA4_MOUSE	Annexin A4 OS=Mus musculus GN=Anxa4 PE=2 SV=3	1	11	1.0	1.88E+07	2.14E+07	[illegible]
Q9D8N0	EF1G_MOUSE	Elongation factor 1-gamma OS=Mus musculus GN=Eef1g PE=1 SV=3	5	13	1.0	1.61E+09	1.71E+09	[illegible]
O70433	FHL2_MOUSE	Four and a half LIM domains protein 2 OS=Mus musculus GN=Fhl2 PE=1 SV=1	10	13	1.0	3.98E+09	2.80E+09	[illegible]
P56375	ACYP2_MOUSE	Acylphosphatase-2 OS=Mus musculus GN=Acyp2 PE=2 SV=1	4	10	1.0	5.70E+08	6.56E+08	[illegible]
P50247	SAHH_MOUSE	Adenosylhomocysteinase OS=Mus musculus GN=Ahcy PE=1 SV=3	3	13	1.0	4.93E+08	5.21E+08	[illegible]
Q9D662	SC23B_MOUSE	Protein transport protein Sec23B OS=Mus musculus GN=Sec23b PE=2 SV=1	1	4	0.64	1.48E+07	3.40E+07	[illegible]
Q8CDN6	TXNL1_MOUSE	Thioredoxin-like protein 1 OS=Mus musculus GN=Txnl1 PE=2 SV=3	2	6	1.0	2.46E+08	2.09E+08	[illegible]
O55234	PSB5_MOUSE	Proteasome subunit beta type-5 OS=Mus musculus GN=Psmb5 PE=1 SV=3	2	9	1.0	1.73E+08	1.47E+08	[illegible]
Q8BWB1	SYP2L_MOUSE	Synaptopodin 2-like protein OS=Mus musculus GN=Synpo2l PE=2 SV=1	3	6	1.0	4.26E+08	3.20E+08	[illegible]
P70349	HINT1_MOUSE	Histidine triad nucleotide-binding protein 1 OS=Mus musculus GN=Hint1 PE=1 SV=3	1	13	0.99	1.21E+09	1.21E+09	[illegible]
Q8VIJ6	SFPQ_MOUSE	Splicing factor, proline- and glutamine-rich OS=Mus musculus GN=Sfpq PE=1 SV=1	2	12	1.0	1.21E+08	8.75E+07	[illegible]
P80315	TCPD_MOUSE	T-complex protein 1 subunit delta OS=Mus musculus GN=Cct4 PE=1 SV=3	4	13	1.0	2.16E+08	3.95E+08	[illegible]
P62827	RAN_MOUSE	GTP-binding nuclear protein Ran OS=Mus musculus GN=Ran PE=1 SV=3	1	12	1.0	3.42E+08	4.14E+08	[illegible]
P36536	SAR1A_MOUSE	GTP-binding protein SAR1a OS=Mus musculus GN=Sar1a PE=2 SV=1	1	7	0.99	1.21E+08	1.32E+08	[illegible]
Q9CQC9	SAR1B_MOUSE	GTP-binding protein SAR1b OS=Mus musculus GN=Sar1b PE=1 SV=1	1	1	0.94	7.55E+07	8.27E+07	[illegible]
Q9Z2W0	DNPEP_MOUSE	Aspartyl aminopeptidase OS=Mus musculus GN=Dnpep PE=2 SV=1	2	7	1.0	1.18E+08	1.84E+08	[illegible]
P80318	TCPG_MOUSE	T-complex protein 1 subunit gamma OS=Mus musculus GN=Cct3 PE=1 SV=1	6	11	1.0	4.84E+08	6.81E+08	[illegible]
P10639	THIO_MOUSE	Thioredoxin OS=Mus musculus GN=Txn PE=1 SV=3	1	12	0.99	5.65E+08	4.85E+08	[illegible]
P31786	ACBP_MOUSE	Acyl-CoA-binding protein OS=Mus musculus GN=Dbi PE=1 SV=2	2	13	1.0	5.68E+09	6.01E+09	[illegible]
P00397	COX1_MOUSE	Cytochrome c oxidase subunit 1 OS=Mus musculus GN=Mtco1 PE=3 SV=1	2	13	1.0	3.09E+09	4.09E+09	[illegible]
Q5EBG6	HSPB6_MOUSE	Heat shock protein beta-6 OS=Mus musculus GN=Hspb6 PE=2 SV=1	5	13	1.0	2.52E+09	2.50E+09	[illegible]
Q7TQI3	OTUB1_MOUSE	Ubiquitin thioesterase OTUB1 OS=Mus musculus GN=Otub1 PE=1 SV=2	3	10	1.0	2.89E+08	3.73E+08	[illegible]
O08997	ATOX1_MOUSE	Copper transport protein ATOX1 OS=Mus musculus GN=Atox1 PE=2 SV=1	1	6	0.99	2.60E+08	2.98E+08	[illegible]
Q9R0P3	ESTD_MOUSE	S-formylglutathione hydrolase OS=Mus musculus GN=Esd PE=2 SV=1	3	13	1.0	1.47E+09	1.59E+09	[illegible]
P46471	PRS7_MOUSE	26S protease regulatory subunit 7 OS=Mus musculus GN=Psmc2 PE=1 SV=5	1	4	1.0	6.61E+07	6.51E+07	[illegible]
Q6P5E4	UGGG1_MOUSE	UDP-glucose:glycoprotein glucosyltransferase 1 OS=Mus musculus GN=Ugcgl1 PE=1 SV=3	1	2	0.99	2.02E+07	1.19E+07	[illegible]
P22599	A1AT2_MOUSE	Alpha-1-antitrypsin 1-2 OS=Mus musculus GN=Serpina1b PE=1 SV=2	3	13	1.0	8.16E+08	6.17E+08	[illegible]
Q00897	A1AT4_MOUSE	Alpha-1-antitrypsin 1-4 OS=Mus musculus GN=Serpina1d PE=2 SV=1	3	13	1.0	1.14E+09	8.78E+08	[illegible]
Q00898	A1AT5_MOUSE	Alpha-1-antitrypsin 1-5 OS=Mus musculus GN=Serpina1e PE=1 SV=1	3	13	1.0	2.01E+09	1.46E+09	[illegible]
Q8BG95	MYPT2_MOUSE	Protein phosphatase 1 regulatory subunit 12B OS=Mus musculus GN=Ppp1r12b PE=1 SV=2	3	13	1.0	2.93E+08	2.10E+08	[illegible]
P21107	TPM3_MOUSE	Tropomyosin alpha-3 chain OS=Mus musculus GN=Tpm3 PE=1 SV=2	1	1	0.99	2.70E+08	1.41E+08	[illegible]
Q9CQX2	CYB5B_MOUSE	Cytochrome b5 type B OS=Mus musculus GN=Cyb5b PE=1 SV=1 [MASS=16318]	1	10	0.99	5.82E+07	6.07E+07	[illegible]
Q9R0Q7	TEBP_MOUSE	Prostaglandin E synthase 3 OS=Mus musculus GN=Ptges3 PE=1 SV=1 [MASS=18721]	1	9	1.0	8.29E+07	1.67E+08	[illegible]
Q8JZN5	ACAD9_MOUSE	Acyl-CoA dehydrogenase family member 9, mitochondrial OS=Mus musculus GN=Acad9 PE=2 SV=1 [MASS=68707]	4	10	1.0	1.94E+09	2.37E+09	[illegible]
P85094	ISC2A_MOUSE	Isochorismatase domain-containing protein 2A, mitochondrial OS=Mus musculus GN=Isoc2a PE=2 SV=1 [MASS=22417]	3	11	1.0	5.79E+08	9.84E+08	[illegible]
Q9D1H6	NDUF4_MOUSE	NADH dehydrogenase [ubiquinone] 1 alpha subcomplex assembly factor 4 OS=Mus musculus GN=Ndufaf4 PE=2 SV=1 [MASS=20082]	1	7	1.0	4.75E+07	1.56E+08	[illegible]
Q9CPU2	NDUB2_MOUSE	NADH dehydrogenase [ubiquinone] 1 beta subcomplex subunit 2, mitochondrial OS=Mus musculus GN=Ndufb2 PE=2 SV=1 [MASS=11967]	1	1	1.0	1.17E+08	1.52E+08	[illegible]
Q9QYA2	TOM40_MOUSE	Mitochondrial import receptor subunit TOM40 homolog OS=Mus musculus GN=Tomm40 PE=1 SV=3 [MASS=37895]	2	11	1.0	4.71E+08	3.93E+08	[illegible]
Q8R164	BPHL_MOUSE	Valacyclovir hydrolase OS=Mus musculus GN=Bphl PE=2 SV=1 [MASS=32851]	3	13	1.0	5.46E+08	7.34E+08	[illegible]
Q8BWM0	PGES2_MOUSE	Prostaglandin E synthase 2 OS=Mus musculus GN=Ptges2 PE=1 SV=2 [MASS=43307]	1	9	1.0	2.42E+08	1.98E+08	[illegible]
O88544	CSN4_MOUSE	COP9 signalosome complex subunit 4 OS=Mus musculus GN=Cops4 PE=1 SV=1	1	4	0.82	8.70E+07	6.27E+07	[illegible]
O09061	PSB1_MOUSE	Proteasome subunit beta type-1 OS=Mus musculus GN=Psmb1 PE=1 SV=1	5	12	1.0	8.39E+08	9.56E+08	[illegible]
Q9R1P3	PSB2_MOUSE	Proteasome subunit beta type-2 OS=Mus musculus GN=Psmb2 PE=1 SV=1	2	4	1.0	2.31E+08	3.49E+08	[illegible]
Q9R1P4	PSA1_MOUSE	Proteasome subunit alpha type-1 OS=Mus musculus GN=Psma1 PE=1 SV=1	1	13	1.0	2.29E+08	1.59E+08	[illegible]
P50136	ODBA_MOUSE	2-oxoisovalerate dehydrogenase subunit alpha, mitochondrial OS=Mus musculus GN=Bckdha PE=1 SV=1	7	13	1.0	1.49E+09	1.77E+09	[illegible]
Q9R1P1	PSB3_MOUSE	Proteasome subunit beta type-3 OS=Mus musculus GN=Psmb3 PE=1 SV=1	1	6	1.0	6.19E+07	1.11E+08	[illegible]
Q8VDM4	PSMD2_MOUSE	26S proteasome non-ATPase regulatory subunit 2 OS=Mus musculus GN=Psmd2 PE=1 SV=1	5	13	1.0	7.89E+08	5.78E+08	[illegible]
P54775	PRS6B_MOUSE	26S protease regulatory subunit 6B OS=Mus musculus GN=Psmc4 PE=1 SV=1	1	1	1.0	5.18E+07	7.18E+07	[illegible]
O35295	PURB_MOUSE	Transcriptional activator protein Pur-beta OS=Mus musculus GN=Purb PE=1 SV=3	1	5	0.99	2.16E+07	1.40E+07	[illegible]
Q9Z1E4	GYS1_MOUSE	Glycogen [starch] synthase, muscle OS=Mus musculus GN=Gys1 PE=1 SV=2	3	8	1.0	4.11E+08	5.94E+08	[illegible]
P14685	PSMD3_MOUSE	26S proteasome non-ATPase regulatory subunit 3 OS=Mus musculus GN=Psmd3 PE=2 SV=2	2	1	1.0	6.45E+07	9.92E+07	[illegible]
Q64481	CP3AG_MOUSE	Cytochrome P450 3A16 OS=Mus musculus GN=Cyp3a16 PE=2 SV=1	1	12	0.92	4.96E+08	5.35E+08	[illegible]
O88685	PRS6A_MOUSE	26S protease regulatory subunit 6A OS=Mus musculus GN=Psmc3 PE=2 SV=1	5	11	1.0	4.80E+08	5.69E+08	[illegible]
Q9JJL8	SYSM_MOUSE	Seryl-tRNA synthetase, mitochondrial OS=Mus musculus GN=Sars2 PE=2 SV=1	1	9	0.99	6.26E+07	5.78E+07	[illegible]
Q99KJ8	DCTN2_MOUSE	Dynactin subunit 2 OS=Mus musculus GN=Dctn2 PE=1 SV=3	4	8	1.0	2.13E+08	3.09E+08	[illegible]
P62334	PRS10_MOUSE	26S protease regulatory subunit S10B OS=Mus musculus GN=Psmc6 PE=1 SV=1	2	12	1.0	1.33E+08	1.90E+08	[illegible]
P62192	PRS4_MOUSE	26S protease regulatory subunit 4 OS=Mus musculus GN=Psmc1 PE=1 SV=1	1	1	0.94	7.97E+07	6.90E+07	[illegible]
Q8JZQ9	EIF3B_MOUSE	Eukaryotic translation initiation factor 3 subunit B OS=Mus musculus GN=Eif3b PE=1 SV=1	2	10	1.0	9.71E+07	7.24E+07	[illegible]
P23116	EIF3A_MOUSE	Eukaryotic translation initiation factor 3 subunit A OS=Mus musculus GN=Eif3a PE=1 SV=4	1	1	0.81	4.55E+06	3.76E+07	[illegible]
Q9QZD9	EIF3I_MOUSE	Eukaryotic translation initiation factor 3 subunit I OS=Mus musculus GN=Eif3i PE=1 SV=1	1	1	0.99	1.81E+07	1.28E+07	[illegible]
Q8BX70	VP13C_MOUSE	Vacuolar protein sorting-associated protein 13C OS=Mus musculus GN=Vps13c PE=2 SV=2	1	7	0.97	1.57E+09	1.03E+09	[illegible]
Q3U1J4	DDB1_MOUSE	DNA damage-binding protein 1 OS=Mus musculus GN=Ddb1 PE=1 SV=2	2	4	1.0	7.95E+07	6.85E+07	[illegible]
O70251	EF1B_MOUSE	Elongation factor 1-beta OS=Mus musculus GN=Eef1b PE=1 SV=5	3	9	1.0	3.80E+08	5.28E+08	[illegible]
Q9DCH4	EIF3F_MOUSE	Eukaryotic translation initiation factor 3 subunit F OS=Mus musculus GN=Eif3f PE=1 SV=1	2	5	1.0	1.18E+08	8.24E+07	[illegible]
P35908	K22E_HUMAN	Keratin, type II cytoskeletal 2 epidermal OS=Homo sapiens GN=KRT2 PE=1 SV=2	15	5	1.0	2.95E+09	1.88E+09	[illegible]
P13645	K1C10_HUMAN	Keratin, type I cytoskeletal 10 OS=Homo sapiens GN=KRT10 PE=1 SV=6	16	13	1.0	3.29E+10	4.22E+09	[illegible]
P35527	K1C9_HUMAN	Keratin, type I cytoskeletal 9 OS=Homo sapiens GN=KRT9 PE=1 SV=3	14	11	1.0	1.92E+10	3.51E+09	[illegible]
P04264	K2C1_HUMAN	Keratin, type II cytoskeletal 1 OS=Homo sapiens GN=KRT1 PE=1 SV=6	15	13	1.0	4.46E+10	5.33E+09	[illegible]
P02533	K1C14_HUMAN	Keratin, type I cytoskeletal 14 OS=Homo sapiens GN=KRT14 PE=1 SV=4	1	4	1.0	1.70E+08	3.36E+07	[illegible]
P13647	K2C5_HUMAN	Keratin, type II cytoskeletal 5 OS=Homo sapiens GN=KRT5 PE=1 SV=3	4	10	1.0	1.00E+09	2.39E+08	[illegible]
P08779	K1C16_HUMAN	Keratin, type I cytoskeletal 16 OS=Homo sapiens GN=KRT16 PE=1 SV=4	1	2	1.0	4.89E+08	3.27E+07	[illegible]
Q9D967	MGDP1_MOUSE	Magnesium-dependent phosphatase 1 OS=Mus musculus GN=Mdp1 PE=1 SV=1	1	8	1.0	2.00E+08	1.97E+08	[illegible]
Q9Z1Z0	USO1_MOUSE	General vesicular transport factor p115 OS=Mus musculus GN=Uso1 PE=1 SV=2	1	8	0.99	5.24E+07	3.62E+07	[illegible]
Q03173	ENAH_MOUSE	Protein enabled homolog OS=Mus musculus GN=Enah PE=1 SV=2	1	9	0.78	7.53E+08	4.66E+08	[illegible]
P97855	G3BP1_MOUSE	Ras GTPase-activating protein-binding protein 1 OS=Mus musculus GN=G3bp1 PE=1 SV=1	2	5	1.0	1.60E+08	1.83E+08	[illegible]
Q9Z1Q9	SYVC_MOUSE	Valyl-tRNA synthetase OS=Mus musculus GN=Vars PE=2 SV=1	2	2	1.0	7.12E+07	6.18E+07	[illegible]
Q9DCD0	6PGD_MOUSE	6-phosphogluconate dehydrogenase, decarboxylating OS=Mus musculus GN=Pgd PE=2 SV=3	2	4	1.0	9.13E+08	1.97E+08	[illegible]
Q9CZM2	RL15_MOUSE	60S ribosomal protein L15 OS=Mus musculus GN=Rpl15 PE=2 SV=4	1	12	1.0	6.03E+07	4.25E+07	[illegible]
P56399	UBP5_MOUSE	Ubiquitin carboxyl-terminal hydrolase 5 OS=Mus musculus GN=Usp5 PE=1 SV=1	2	2	1.0	1.61E+08	1.95E+08	[illegible]
Q9ERK4	XPO2_MOUSE	Exportin-2 OS=Mus musculus GN=Cse1l PE=2 SV=1	2	4	1.0	1.50E+08	1.30E+08	[illegible]
Q64737	PUR2_MOUSE	Trifunctional purine biosynthetic protein adenosine-3 OS=Mus musculus GN=Gart PE=2 SV=2	3	9	1.0	2.60E+08	3.88E+08	[illegible]
P45377	ALD2_MOUSE	Aldose reductase-related protein 2 OS=Mus musculus GN=Akr1b8 PE=1 SV=2	1	11	0.99	2.00E+08	1.83E+08	[illegible]
Q8BKC5	IPO5_MOUSE	Importin-5 OS=Mus musculus GN=Ipo5 PE=1 SV=3	2	5	1.0	1.01E+08	1.46E+08	[illegible]
P12367	KAP2_MOUSE	cAMP-dependent protein kinase type II-alpha regulatory subunit OS=Mus musculus GN=Prkar2a PE=1 SV=2	1	13	0.99	1.41E+08	1.92E+08	[illegible]
P54116	STOM_MOUSE	Erythrocyte band 7 integral membrane protein OS=Mus musculus GN=Stom PE=1 SV=3	1	1	1.0	5.47E+07	6.50E+07	[illegible]
P80316	TCPE_MOUSE	T-complex protein 1 subunit epsilon OS=Mus musculus GN=Cct5 PE=1 SV=1	3	6	1.0	2.06E+08	2.63E+08	[illegible]
Q9QZ23	NFU1_MOUSE	NFU1 iron-sulfur cluster scaffold homolog, mitochondrial OS=Mus musculus GN=Nfu1 PE=1 SV=2	1	10	0.99	9.46E+07	1.69E+08	[illegible]
Q8K1M6	DNM1L_MOUSE	Dynamin-1-like protein OS=Mus musculus GN=Dnm1l PE=1 SV=2	9	12	1.0	1.31E+09	1.70E+09	[illegible]
Q8CGC7	SYEP_MOUSE	Bifunctional aminoacyl-tRNA synthetase OS=Mus musculus GN=Eprs PE=2 SV=3	2	9	1.0	3.19E+08	3.31E+08	[illegible]
Q9QUR6	PPCE_MOUSE	Prolyl endopeptidase OS=Mus musculus GN=Prep PE=2 SV=1	1	11	0.99	1.42E+08	1.67E+08	[illegible]
P58774	TPM2_MOUSE	Tropomyosin beta chain OS=Mus musculus GN=Tpm2 PE=1 SV=1	1	8	1.0	6.78E+07	5.17E+07	[illegible]
P26040	EZRI_MOUSE	Ezrin OS=Mus musculus GN=Ezr PE=1 SV=3	2	13	1.0	4.82E+08	3.61E+08	[illegible]
Q8K0D5	EFGM_MOUSE	Elongation factor G, mitochondrial OS=Mus musculus GN=Gfm1 PE=2 SV=1	2	12	1.0	4.39E+08	2.78E+08	[illegible]
Q9DC77	SMPX_MOUSE	Small muscular protein OS=Mus musculus GN=Smpx PE=2 SV=2	3	13	1.0	5.82E+08	5.45E+08	[illegible]
P11276	FINC_MOUSE	Fibronectin OS=Mus musculus GN=Fn1 PE=1 SV=3	1	1	1.0	2.39E+07	5.30E+07	[illegible]
Q9CZY3	UB2V1_MOUSE	Ubiquitin-conjugating enzyme E2 variant 1 OS=Mus musculus GN=Ube2v1 PE=1 SV=1	1	7	1.0	6.94E+07	7.13E+07	[illegible]
P84089	ERH_MOUSE	Enhancer of rudimentary homolog OS=Mus musculus GN=Erh PE=1 SV=1	1	13	0.99	2.81E+07	1.99E+07	[illegible]
P51859	HDGF_MOUSE	Hepatoma-derived growth factor OS=Mus musculus GN=Hdgf PE=1 SV=2	2	5	1.0	1.41E+08	1.56E+08	[illegible]
Q8CGY6	UN45B_MOUSE	Protein unc-45 homolog B OS=Mus musculus GN=Unc45b PE=2 SV=1	1	11	1.0	5.49E+07	3.25E+07	[illegible]
Q9CQ45	NENF_MOUSE	Neudesin OS=Mus musculus GN=Nenf PE=1 SV=1	1	1	0.86	3.54E+07	2.77E+07	[illegible]
Q91VI7	RINI_MOUSE	Ribonuclease inhibitor OS=Mus musculus GN=Rnh1 PE=2 SV=1	2	11	1.0	1.87E+08	2.46E+08	[illegible]
P54822	PUR8_MOUSE	Adenylosuccinate lyase OS=Mus musculus GN=Adsl PE=2 SV=1	2	1	1.0	1.61E+08	2.65E+08	[illegible]
P80313	TCPH_MOUSE	T-complex protein 1 subunit eta OS=Mus musculus GN=Cct7 PE=1 SV=1	3	8	1.0	2.49E+08	2.88E+08	[illegible]
Q99KB8	GLO2_MOUSE	Hydroxyacylglutathione hydrolase, mitochondrial OS=Mus musculus GN=Hagh PE=2 SV=2	2	13	1.0	3.55E+08	2.42E+08	[illegible]
P70168	IMB1_MOUSE	Importin subunit beta-1 OS=Mus musculus GN=Kpnb1 PE=1 SV=1	2	7	1.0	3.57E+07	1.02E+08	[illegible]
Q91VR5	DDX1_MOUSE	ATP-dependent RNA helicase DDX1 OS=Mus musculus GN=Ddx1 PE=2 SV=1	3	8	1.0	2.85E+08	3.60E+08	[illegible]
P62869	ELOB_MOUSE	Transcription elongation factor B polypeptide 2 OS=Mus musculus GN=Tceb2 PE=1 SV=1	1	1	0.99	1.18E+08	1.89E+08	[illegible]
Q61584	FXR1_MOUSE	Fragile X mental retardation syndrome-related protein 1 OS=Mus musculus GN=Fxr1 PE=1 SV=2	2	12	1.0	1.74E+08	1.42E+08	[illegible]
Q99KC8	VMA5A_MOUSE	von Willebrand factor A domain-containing protein 5A OS=Mus musculus GN=Vwa5a PE=1 SV=2	1	7	0.99	1.32E+07	4.32E+07	[illegible]
Q9CYR0	SSBP_MOUSE	Single-stranded DNA-binding protein, mitochondrial OS=Mus musculus GN=Ssbp1 PE=2 SV=1	3	7	1.0	4.52E+08	4.53E+08	[illegible]
Q61937	NPM_MOUSE	Nucleophosmin OS=Mus musculus GN=Npm1 PE=1 SV=1	2	11	1.0	2.79E+08	4.86E+08	[illegible]
Q9WTX5	SKP1_MOUSE	S-phase kinase-associated protein 1 OS=Mus musculus GN=Skp1 PE=1 SV=3	3	2	1.0	2.47E+08	2.38E+08	[illegible]
Q05920	PYC_MOUSE	Pyruvate carboxylase, mitochondrial OS=Mus musculus GN=Pc PE=1 SV=1	1	4	1.0	3.10E+07	3.66E+07	[illegible]
Q9JHU4	DYHC1_MOUSE	Cytoplasmic dynein 1 heavy chain 1 OS=Mus musculus GN=Dync1h1 PE=1 SV=1	10	12	1.0	8.21E+08	8.98E+08	[illegible]
P26516	PSD7_MOUSE	26S proteasome non-ATPase regulatory subunit 7 OS=Mus musculus GN=Psmd7 PE=1 SV=2	1	1	0.99	1.92E+07	4.61E+07	[illegible]
Q78ZA7	NP1L4_MOUSE	Nucleosome assembly protein 1-like 4 OS=Mus musculus GN=Nap1l4 PE=1 SV=1	3	11	1.0	4.07E+08	4.84E+08	[illegible]
Q9D8S4	ORN_MOUSE	Oligoribonuclease, mitochondrial OS=Mus musculus GN=Rexo2 PE=1 SV=2	1	2	0.96	3.43E+07	5.11E+07	[illegible]
Q9CYZ2	TPD54_MOUSE	Tumor protein D54 OS=Mus musculus GN=Tpd52l2 PE=1 SV=1	1	5	0.99	6.22E+07	1.35E+08	[illegible]
Q11011	PSA_MOUSE	Puromycin-sensitive aminopeptidase OS=Mus musculus GN=Npepps PE=1 SV=1	3	4	1.0	3.96E+08	4.17E+08	[illegible]
Q60634	FLOT2_MOUSE	Flotillin-2 OS=Mus musculus GN=Flot2 PE=1 SV=2	1	2	0.97	7.27E+07	7.30E+07	[illegible]
Q923D2	BLVRB_MOUSE	Flavin reductase OS=Mus musculus GN=Blvrb PE=2 SV=3	1	10	0.99	8.81E+07	5.95E+07	[illegible]

P46412	GPX3_MOUSE	Glutathione peroxidase 3 OS=Mus musculus GN=Gpx3 PE=2 SV=2	3	5	1.0	3.57E+08	4.48E+08	3.39E+08	3.77E+08	3.63E+08	4.25E+08	4.36E+08	3.59E+08	3.68E+08	4.11E+08	4.61E+08	2.99E+08	3.39E+08	1.01E-04	1.05E-04	7.10E-05	1.03E-02	3.96E-03	2.98E-07	6.16E-05	2.62E-04	2.53E-03	1.27E-07	2.93E-02	1.41E-03	5.87E-01	3, 4	3, 5	3, 5	GPX3_MOUSE, SP:P46412	SP|P46412|GPX3_MOUSE
P62960	YBOX1_MOUSE	Nuclease-sensitive element-binding protein 1 OS=Mus musculus GN=Ybx1 PE=1 SV=3	2	13	1.0	1.98E+08	1.57E+08	1.59E+08	1.82E+08	1.87E+08	1.27E+08	1.41E+08	1.65E+08	1.98E+08	2.05E+08	1.94E+08	1.72E+08	1.59E+08	4.12E-02	1.23E-01	9.98E-02	9.41E-01	0.007	7.72E-02	0.001	9.13E-02	5.32E-01	1.44E-02	9.40E-01	6.22E-01	0.012	3, 4	13, 4	13, 4	YBOX1_MOUSE, SP:P62960	SP|P62960|YBOX1_MOUSE
Q99KQ4	NAMPT_MOUSE	Nicotinamide phosphoribosyltransferase OS=Mus musculus GN=Nampt PE=1 SV=1	4	13	1.0	6.86E+08	1.07E+09	9.59E+08	7.26E+08	1.05E+09	1.16E+09	9.81E+08	1.12E+09	8.30E+08	9.13E+08	9.43E+08	8.81E+08	7.22E+08	1.65E-10	2.78E-16	8.11E-11	1.09E-10	4.14E-11	9.90E-20	6.86E-13	1.35E-11	3.26E-09	2.53E-17	1.40E-09	3.21E-14	8.97E-04	2, 4	13, 2, 6, 13	13, 13, 6, 2, 23	NAMPT_MOUSE, SP:Q99KQ4	SP|Q99KQ4|NAMPT_MOUSE
Q8BVI4	DHPR_MOUSE	Dihydropteridine reductase OS=Mus musculus GN=Qdpr PE=1 SV=2	3	12	1.0	5.51E+08	5.88E+08	5.77E+08	5.74E+08	5.77E+08	4.90E+08	6.52E+08	4.45E+08	5.61E+08	5.16E+08	5.06E+08	5.94E+08	5.09E+08	3.16E-08	2.20E-09	2.41E-08	2.60E-05	6.01E-04	1.70E-10	3.29E-08	1.75E-07	1.13E-04	8.49E-09	4.62E-06	6.31E-05	3.02E-02	2, 3, 3, 4	10, 2, 3, 12	10, 12, 3, 2	DHPR_MOUSE, SP:Q8BVI4	SP|Q8BVI4|DHPR_MOUSE
Q9WV55	VAPA_MOUSE	Vesicle-associated membrane protein-associated protein A OS=Mus musculus GN=Vapa PE=1 SV=2	1	6	1.0	3.16E+07	3.01E+07	2.70E+07	3.44E+07	2.45E+07	2.62E+07	2.79E+07	3.54E+07	3.82E+07	3.52E+07	2.39E+07	2.90E+07	2.59E+07	0.063	0.075	0.057	0.142	0.139	0.057	0.098	0.056	0.094	0.081	0.111	0.074	0.186	4	6	6	VAPA_MOUSE, SP:Q9WV55	SP|Q9WV55|VAPA_MOUSE
Q9WV98	TIM9_MOUSE	Mitochondrial import inner membrane translocase subunit Tim9 OS=Mus musculus GN=Timm9 PE=1 SV=1	1	13	0.99	7.25E+08	5.65E+08	4.35E+08	6.77E+08	4.44E+08	4.58E+08	6.46E+08	5.08E+08	5.69E+08	4.65E+08	5.54E+08	6.00E+08	4.56E+08	1.28E-10	1.39E-06	1.33E-09	1.12E-03	1.54E-03	1.10E-09	7.16E-08	2.21E-07	5.72E-04	1.86E-09	5.71E-06	1.08E-05	7.96E-02	5	13	13	TIM9_MOUSE, SP:Q9WV98	SP|Q9WV98|TIM9_MOUSE
Q9CQ65	MTAP_MOUSE	S-methyl-5'-thioadenosine phosphorylase OS=Mus musculus GN=Mtap PE=2 SV=1	1	2	0.99	8.39E+07	1.00E+08	8.64E+07	1.03E+08	7.73E+07	9.25E+07	8.01E+07	7.99E+07	9.67E+07	8.22E+07	8.50E+07	1.04E+08	9.40E+07	0.006	0.004	0.003	0.025	0.019	0.002	0.012	0.005	0.021	0.003	0.008	0.013	0.043	4	2	2	MTAP_MOUSE, SP:Q9CQ65	SP|Q9CQ65|MTAP_MOUSE
Q99MN1	SYK_MOUSE	Lysyl-tRNA synthetase OS=Mus musculus GN=Kars PE=1 SV=1	2	9	1.0	6.37E+07	1.05E+08	7.67E+07	8.09E+07	8.43E+07	1.09E+08	7.26E+07	8.99E+07	7.83E+07	1.02E+08	9.28E+07	6.85E+07	6.14E+07	0.013	0.005	0.006	0.018	0.011	9.89E-01	0.014	0.009	0.011	0.002	0.025	0.008	0.081	2, 3	5, 9	5, 9	SYK_MOUSE, SP:Q99MN1	SP|Q99MN1|SYK_MOUSE
Q9DCM0	ETHE1_MOUSE	Protein ETHE1, mitochondrial OS=Mus musculus GN=Ethe1 PE=1 SV=2	1	4	1.0	9.89E+07	1.91E+08	1.62E+08	1.27E+08	1.02E+08	2.30E+08	1.94E+08	1.78E+08	1.41E+08	1.57E+08	1.71E+08	1.49E+08	1.12E+08	0.003	1.22E-01	9.92E-01	0.011	4.38E-01	1.86E-02	1.81E-01	8.01E-01	0.002	4.45E-02	0.002	4.19E-01	0.030	5	4	4	ETHE1_MOUSE, SP:Q9DCM0	SP|Q9DCM0|ETHE1_MOUSE
Q9CQZ1	HSBP1_MOUSE	Heat shock factor-binding protein 1 OS=Mus musculus GN=Hsbp1 PE=2 SV=1	1	1	0.92	5.36E+07	6.68E+07	6.81E+07	6.50E+07	7.63E+07	7.38E+07	6.62E+07	8.49E+07	7.31E+07	7.12E+07	6.59E+07	7.30E+07	5.95E+07	0.023	0.010	0.015	0.025	0.031	0.008	0.020	0.013	0.028	0.009	0.023	0.012	0.086	4	1	1	HSBP1_MOUSE, SP:Q9CQZ1	SP|Q9CQZ1|HSBP1_MOUSE
Q922D8	C1TC_MOUSE	C-1-tetrahydrofolate synthase, cytoplasmic OS=Mus musculus GN=Mthfd1 PE=1 SV=3	1	2	0.63	2.73E+07	4.56E+07	3.81E+07	2.37E+07	2.31E+07	3.51E+07	2.84E+07	2.65E+07	3.45E+07	3.16E+07	3.38E+07	2.23E+07	2.77E+07	0.098	0.053	0.119	0.170	0.117	0.042	0.115	0.075	0.117	0.063	0.147	0.129	0.182	3	2	2	C1TC_MOUSE, SP:Q922D8	SP|Q922D8|C1TC_MOUSE
P15532	NDKA_MOUSE	Nucleoside diphosphate kinase A OS=Mus musculus GN=Nme1 PE=1 SV=1	2	4	1.0	1.41E+08	1.37E+08	1.47E+08	1.32E+08	1.47E+08	1.14E+08	1.22E+08	1.40E+08	1.93E+08	1.40E+08	1.59E+08	1.33E+08	1.37E+08	4.56E-01	2.41E-01	8.21E-01	0.003	0.010	2.28E-01	0.002	1.17E-01	0.003	7.60E-02	0.003	0.001	0.019	2, 3	1, 4	1, 4	NDKA_MOUSE, SP:P15532	SP|P15532|NDKA_MOUSE
Q9DCL9	PUR6_MOUSE	Multifunctional protein ADE2 OS=Mus musculus GN=Paics PE=1 SV=3	1	3	0.97	8.42E+07	1.64E+08	1.33E+08	8.36E+07	8.82E+07	1.72E+08	1.07E+08	1.55E+08	9.58E+07	1.34E+08	1.25E+08	6.92E+07	7.95E+07	0.008	0.001	0.008	0.021	0.003	2.91E-01	0.006	0.008	0.006	0.001	0.031	0.002	0.062	3	3	3	PUR6_MOUSE, SP:Q9DCL9	SP|Q9DCL9|PUR6_MOUSE
Q99JW4	LIMS1_MOUSE	LIM and senescent cell antigen-like-containing domain protein 1 OS=Mus musculus GN=Lims1 PE=1 SV=3	1	1	0.78	6.65E+07	6.11E+07	7.11E+07	6.38E+07	8.67E+07	5.34E+07	5.63E+07	6.32E+07	7.89E+07	5.36E+07	7.63E+07	5.17E+07	6.44E+07	0.015	0.011	0.022	0.021	0.061	0.013	0.036	0.011	0.055	0.006	0.051	0.027	0.083	2	1	1	LIMS1_MOUSE, SP:Q99JW4	SP|Q99JW4|LIMS1_MOUSE
Q3UM45	PP1R7_MOUSE	Protein phosphatase 1 regulatory subunit 7 OS=Mus musculus GN=Ppp1r7 PE=1 SV=2	2	8	1.0	1.21E+08	1.38E+08	1.50E+08	1.65E+08	2.24E+08	1.86E+08	1.53E+08	1.47E+08	1.75E+08	1.61E+08	1.30E+08	1.55E+08	1.61E+08	0.001	2.13E-01	2.10E-01	3.51E-01	0.001	2.25E-01	8.32E-01	2.42E-01	0.002	3.49E-01	0.002	0.001	0.012	3	2, 2, 8	2, 2, 8	PP1R7_MOUSE, SP:Q3UM45	SP|Q3UM45|PP1R7_MOUSE
Q91V64	ISOC1_MOUSE	Isochorismatase domain-containing protein 1 OS=Mus musculus GN=Isoc1 PE=2 SV=1	2	13	1.0	1.89E+08	2.83E+08	2.10E+08	2.17E+08	2.82E+08	3.03E+08	2.25E+08	2.22E+08	2.53E+08	2.16E+08	2.01E+08	1.85E+08	1.60E+08	6.14E-02	1.48E-02	2.58E-02	7.01E-02	6.63E-02	2.28E-04	6.11E-02	1.23E-02	4.00E-01	1.16E-02	6.75E-01	9.35E-02	0.012	3, 4	13, 3	13, 3	ISOC1_MOUSE, SP:Q91V64	SP|Q91V64|ISOC1_MOUSE
Q62523	ZYX_MOUSE	Zyxin OS=Mus musculus GN=Zyx PE=1 SV=1	1	2	0.99	2.74E+07	1.48E+07	1.61E+07	1.66E+07	4.53E+07	1.90E+07	2.07E+07	1.68E+07	2.78E+07	2.82E+07	2.29E+07	2.55E+07	2.09E+07	0.070	0.121	0.130	0.063	0.168	0.122	0.120	0.079	0.112	0.074	0.115	0.150	0.207	2	2	2	ZYX_MOUSE, SP:Q62523	SP|Q62523|ZYX_MOUSE
Q9R053	SCNBA_MOUSE	Sodium channel protein type 11 subunit alpha OS=Mus musculus GN=Scn11a PE=1 SV=1	1	2	0.63	3.52E+08	6.45E+08	4.09E+08	6.45E+08	3.08E+08	4.91E+08	4.04E+08	5.40E+08	2.91E+08	4.03E+08	4.44E+08	4.18E+08	4.57E+08	8.32E-05	2.76E-06	1.96E-09	3.42E-02	5.81E-04	1.80E-11	1.34E-04	2.95E-03	2.43E-03	1.52E-07	7.05E-04	3.15E-06	7.17E-02	4, 6	1	2	SCNBA_MOUSE, SP:Q9R053	SP|Q9R053|SCNBA_MOUSE
P49312	ROA1_MOUSE	Heterogeneous nuclear ribonucleoprotein A1 OS=Mus musculus GN=Hnrnpa1 PE=1 SV=2	1	5	0.91	6.44E+07	9.99E+07	6.46E+07	5.73E+07	6.33E+07	5.99E+07	7.00E+07	9.29E+07	8.01E+07	8.39E+07	7.83E+07	5.48E+07	4.33E+07	0.012	0.010	0.019	0.035	0.044	0.001	0.016	0.009	0.018	0.004	0.040	0.008	0.120	3	5	5	ROA1_MOUSE, SP:P49312	SP|P49312|ROA1_MOUSE
Q9DCZ1	GMPR1_MOUSE	GMP reductase 1 OS=Mus musculus GN=Gmpr PE=2 SV=1	1	5	1.0	3.29E+07	4.51E+07	3.82E+07	2.91E+07	3.84E+07	4.32E+07	3.27E+07	6.20E+07	5.07E+07	4.05E+07	4.54E+07	4.59E+07	3.81E+07	0.054	0.036	0.068	0.079	0.074	0.021	0.070	0.029	0.073	0.021	0.054	0.024	0.135	3	5	5	GMPR1_MOUSE, SP:Q9DCZ1	SP|Q9DCZ1|GMPR1_MOUSE
P23506	PIMT_MOUSE	Protein-L-isoaspartate(D-aspartate) O-methyltransferase OS=Mus musculus GN=Pcmt1 PE=1 SV=3	2	13	1.0	2.22E+08	3.19E+08	2.89E+08	1.88E+08	2.14E+08	3.19E+08	2.45E+08	2.59E+08	2.83E+08	2.85E+08	3.26E+08	2.10E+08	1.81E+08	1.68E-02	4.46E-04	8.79E-02	4.88E-01	4.65E-02	4.14E-05	3.07E-02	4.01E-03	6.19E-02	3.11E-05	3.28E-01	2.77E-02	0.009	4	10, 13	10, 13	PIMT_MOUSE, SP:P23506	SP|P23506|PIMT_MOUSE
Q9WVB0	RBPMS_MOUSE	RNA-binding protein with multiple splicing OS=Mus musculus GN=Rbpms PE=2 SV=1	1	4	0.99	6.02E+07	3.30E+07	5.41E+07	5.27E+07	7.72E+07	4.02E+07	6.62E+07	4.70E+07	6.13E+07	4.44E+07	4.74E+07	5.71E+07	4.88E+07	0.015	0.016	0.022	0.023	0.081	0.041	0.018	0.018	0.063	0.019	0.037	0.042	0.106	3	4	4	RBPMS_MOUSE, SP:Q9WVB0	SP|Q9WVB0|RBPMS_MOUSE
Q9D0M5	DYL2_MOUSE	Dynein light chain 2, cytoplasmic OS=Mus musculus GN=Dynll2 PE=1 SV=1	2	13	1.0	4.12E+08	5.33E+08	7.41E+08	5.77E+08	5.56E+08	3.81E+08	4.93E+08	3.84E+08	3.99E+08	4.79E+08	4.05E+08	6.93E+08	7.31E+08	9.09E-06	2.90E-12	3.72E-08	6.33E-05	1.04E-02	4.20E-09	9.96E-06	6.94E-05	3.78E-04	1.08E-06	3.35E-07	5.62E-04	8.53E-04	2, 3	13, 3	13, 3	DYL2_MOUSE, SP:Q9D0M5	SP|Q9D0M5|DYL2_MOUSE
P61161	ARP2_MOUSE	Actin-related protein 2 OS=Mus musculus GN=Actr2 PE=1 SV=1	1	4	0.99	1.17E+08	1.04E+08	1.07E+08	1.26E+08	1.38E+08	1.28E+08	1.11E+08	1.11E+08	1.19E+08	1.25E+08	1.17E+08	9.78E+07	1.27E+08	0.002	0.002	0.001	0.004	0.007	0.001	0.004	0.002	0.006	7.85E-01	0.011	0.005	0.024	2	4	4	ARP2_MOUSE, SP:P61161	SP|P61161|ARP2_MOUSE
P56959	FUS_MOUSE	RNA-binding protein FUS OS=Mus musculus GN=Fus PE=2 SV=1	2	13	1.0	1.27E+08	1.41E+08	1.32E+08	1.32E+08	1.54E+08	1.38E+08	1.11E+08	1.58E+08	1.54E+08	1.38E+08	1.28E+08	1.30E+08	1.13E+08	8.25E-01	4.35E-01	7.80E-01	0.002	0.005	1.86E-01	0.003	4.80E-01	0.004	3.38E-01	0.003	8.16E-01	0.029	3	13, 4	13, 4	FUS_MOUSE, SP:P56959	SP|P56959|FUS_MOUSE
P28271	ACOC_MOUSE	Cytoplasmic aconitate hydratase OS=Mus musculus GN=Aco1 PE=1 SV=2	3	6	1.0	1.12E+08	1.27E+08	1.06E+08	8.53E+07	1.19E+08	9.78E+07	8.59E+07	1.39E+08	9.02E+07	7.38E+07	8.61E+07	8.77E+07	1.10E+08	0.002	0.001	0.006	0.007	0.015	3.52E-01	0.009	0.006	0.025	0.003	0.013	0.002	0.031	2, 3	1, 6	1, 6	ACOC_MOUSE, SP:P28271	SP|P28271|ACOC_MOUSE
Q9D0I9	SYRC_MOUSE	Arginyl-tRNA synthetase, cytoplasmic OS=Mus musculus GN=Rars PE=2 SV=2	1	2	0.9	1.27E+07	1.64E+07	1.28E+07	1.90E+07	2.27E+07	2.07E+07	1.06E+07	1.98E+07	1.09E+07	1.51E+07	2.22E+07	1.12E+07	1.74E+07	0.170	0.163	0.125	0.145	0.164	0.125	0.214	0.203	0.195	0.086	0.224	0.142	0.233	3	2	2	SYRC_MOUSE, SP:Q9D0I9	SP|Q9D0I9|SYRC_MOUSE
Q9QXM0	ABHD2_MOUSE	Abhydrolase domain-containing protein 2 OS=Mus musculus GN=Abhd2 PE=2 SV=1	1	3	0.89	2.06E+08	9.71E+07	7.59E+07	2.20E+08	1.07E+08	1.07E+08	1.30E+08	9.43E+07	1.31E+08	1.16E+08	1.19E+08	1.52E+08	1.98E+08	3.25E-02	0.006	2.33E-02	0.009	0.011	0.001	0.002	0.001	0.007	5.09E-01	0.002	0.007	0.006	4	3	3	ABHD2_MOUSE, SP:Q9QXM0	SP|Q9QXM0|ABHD2_MOUSE
Q9DAK9	PHP14_MOUSE	14 kDa phosphohistidine phosphatase OS=Mus musculus GN=Phpt1 PE=1 SV=1	2	13	1.0	5.01E+08	5.55E+08	4.93E+08	6.98E+08	3.66E+08	4.58E+08	5.96E+08	3.87E+08	5.74E+08	5.21E+08	4.36E+08	6.21E+08	5.39E+08	2.33E-07	7.25E-08	3.14E-10	6.97E-03	1.29E-03	1.00E-09	2.29E-07	1.19E-07	1.04E-04	2.23E-07	2.29E-06	4.10E-04	1.86E-02	4	13, 9	13, 9	PHP14_MOUSE, SP:Q9DAK9	SP|Q9DAK9|PHP14_MOUSE
Q9ESL4	MLTK_MOUSE	Mitogen-activated protein kinase kinase kinase MLT OS=Mus musculus GN=Mltk PE=1 SV=1	1	6	0.99	6.19E+07	1.08E+08	7.10E+07	5.05E+07	8.85E+07	9.78E+07	8.35E+07	9.92E+07	8.29E+07	9.26E+07	9.49E+07	6.32E+07	6.22E+07	0.014	0.008	0.026	0.016	0.015	0.001	0.010	0.008	0.014	0.002	0.030	0.007	0.080	4	6	6	MLTK_MOUSE, SP:Q9ESL4	SP|Q9ESL4|MLTK_MOUSE
P48722	HS74L_MOUSE	Heat shock 70 kDa protein 4L OS=Mus musculus GN=Hspa4l PE=1 SV=2	1	1	0.99	3.70E+07	2.64E+07	2.49E+07	3.43E+07	3.89E+07	2.36E+07	1.75E+07	2.78E+07	2.63E+07	3.19E+07	3.19E+07	2.56E+07	2.54E+07	0.044	0.073	0.052	0.077	0.142	0.060	0.140	0.085	0.098	0.044	0.115	0.092	0.184	3	1	1	HS74L_MOUSE, SP:P48722	SP|P48722|HS74L_MOUSE
P06801	MAOX_MOUSE	NADP-dependent malic enzyme OS=Mus musculus GN=Me1 PE=1 SV=1	2	7	1.0	1.27E+08	1.62E+08	1.42E+08	1.31E+08	1.58E+08	1.47E+08	1.51E+08	1.51E+08	1.62E+08	1.38E+08	1.83E+08	1.43E+08	8.24E+07	7.90E-01	2.66E-01	8.07E-01	0.002	0.004	6.33E-02	7.90E-01	3.50E-01	0.004	2.41E-02	0.002	9.92E-01	0.053	4, 5	5, 7	5, 7	MAOX_MOUSE, SP:P06801	SP|P06801|MAOX_MOUSE
Q9WUZ7	SH3BG_MOUSE	SH3 domain-binding glutamic acid-rich protein OS=Mus musculus GN=Sh3bgr PE=2 SV=1	1	13	1.0	4.09E+08	6.67E+08	6.24E+08	5.07E+08	7.22E+08	6.76E+08	6.30E+08	6.34E+08	7.29E+08	6.86E+08	6.12E+08	7.30E+08	5.82E+08	8.44E-06	3.05E-10	3.24E-07	6.06E-07	5.93E-06	5.62E-12	7.25E-08	4.80E-10	1.28E-06	7.89E-11	1.02E-07	1.49E-07	8.94E-03	5, 6	12, 13	12, 13	SH3BG_MOUSE, SP:Q9WUZ7	SP|Q9WUZ7|SH3BG_MOUSE
P62627	DLRB1_MOUSE	Dynein light chain roadblock-type 1 OS=Mus musculus GN=Dynlrb1 PE=1 SV=3	2	1	1.0	1.26E+08	2.03E+08	1.58E+08	1.14E+08	1.56E+08	1.60E+08	1.24E+08	1.73E+08	1.81E+08	1.65E+08	1.65E+08	1.51E+08	1.46E+08	0.001	1.87E-01	0.002	0.002	0.003	1.37E-02	0.002	2.10E-01	0.002	8.05E-02	0.002	5.87E-01	0.016	2, 3	1	1	DLRB1_MOUSE, SP:P62627	SP|P62627|DLRB1_MOUSE
P31230	AIMP1_MOUSE	Aminoacyl tRNA synthetase complex-interacting multifunctional protein 1 OS=Mus musculus GN=Aimp1 PE=1 SV=2	2	7	1.0	2.09E+08	1.81E+08	2.07E+08	1.98E+08	1.64E+08	2.05E+08	1.60E+08	2.17E+08	1.88E+08	2.11E+08	2.14E+08	1.80E+08	1.71E+08	2.96E-02	1.94E-02	5.78E-02	0.002	8.48E-01	3.03E-02	6.16E-01	1.45E-01	4.82E-01	6.41E-03	8.18E-01	1.15E-01	0.010	2, 3	1, 7	1, 7	AIMP1_MOUSE, SP:P31230	SP|P31230|AIMP1_MOUSE
Q9D7N9	APMAP_MOUSE	Adipocyte plasma membrane-associated protein OS=Mus musculus GN=Apmap PE=1 SV=1	1	3	0.99	8.41E+07	8.69E+07	1.02E+08	1.10E+08	1.43E+08	1.19E+08	1.21E+08	1.25E+08	1.03E+08	1.17E+08	9.41E+07	1.10E+08	1.30E+08	0.005	0.002	0.002	0.003	0.008	0.002	0.002	0.004	0.007	0.002	0.007	0.002	0.021	4	3	3	APMAP_MOUSE, SP:Q9D7N9	SP|Q9D7N9|APMAP_MOUSE
O88487	DC1I2_MOUSE	Cytoplasmic dynein 1 intermediate chain 2 OS=Mus musculus GN=Dync1i2 PE=2 SV=1	1	6	0.98	5.35E+07	3.81E+07	4.57E+07	4.36E+07	5.79E+07	3.03E+07	4.33E+07	3.13E+07	4.90E+07	3.81E+07	3.10E+07	5.33E+07	3.96E+07	0.021	0.026	0.036	0.043	0.117	0.033	0.047	0.032	0.082	0.048	0.044	0.083	0.137	3	6	6	DC1I2_MOUSE, SP:O88487	SP|O88487|DC1I2_MOUSE
Q99KP6	PRP19_MOUSE	Pre-mRNA-processing factor 19 OS=Mus musculus GN=Prpf19 PE=2 SV=1	1	3	0.66	3.83E+07	3.89E+07	6.20E+07	3.68E+07	3.80E+07	6.59E+07	6.94E+07	5.47E+07	5.56E+07	6.76E+07	5.80E+07	4.21E+07	3.29E+07	0.044	0.013	0.051	0.085	0.039	0.036	0.018	0.026	0.032	0.013	0.066	0.034	0.156	4	3	3	PRP19_MOUSE, SP:Q99KP6	SP|Q99KP6|PRP19_MOUSE
P55264	ADK_MOUSE	Adenosine kinase OS=Mus musculus GN=Adk PE=1 SV=2	5	12	1.0	1.54E+09	1.71E+09	1.65E+09	1.86E+09	1.73E+09	1.88E+09	1.62E+09	1.77E+09	1.89E+09	1.66E+09	1.85E+09	1.78E+09	1.44E+09	7.84E-25	9.92E-29	4.18E-29	3.13E-18	1.07E-18	2.99E-32	6.23E-22	7.88E-28	1.13E-17	1.03E-34	1.68E-20	4.72E-23	7.65E-09	3, 4, 4, 5, 5, 7	10, 10, 12, 11, 12	10, 10, 12, 11, 12	ADK_MOUSE, SP:P55264	SP|P55264|ADK_MOUSE
P28474	ADHX_MOUSE	Alcohol dehydrogenase class-3 OS=Mus musculus GN=Adh5 PE=1 SV=3	1	4	0.99	2.51E+07	1.84E+07	1.11E+08	1.70E+07	1.74E+07	6.71E+06	1.12E+07	3.11E+06	1.25E+07	1.25E+07	2.43E+07	5.61E+07	7.13E+07	0.080	0.001	0.127	0.171	0.284	0.097	0.194	0.174	0.209	0.069	0.038	0.329	0.066	3	3	4	ADHX_MOUSE, SP:P28474	SP|P28474|ADHX_MOUSE
O89023	TPP1_MOUSE	Tripeptidyl-peptidase 1 OS=Mus musculus GN=Tpp1 PE=1 SV=2	1	5	0.77	1.04E+08	1.21E+08	1.14E+08	1.56E+08	1.14E+08	9.22E+07	1.78E+08	7.87E+07	1.56E+08	1.48E+08	1.36E+08	1.52E+08	1.13E+08	0.002	9.74E-01	3.08E-01	0.008	0.017	4.67E-01	3.20E-01	4.47E-01	0.003	2.30E-01	0.002	0.013	0.029	4	5	5	TPP1_MOUSE, SP:O89023	SP|O89023|TPP1_MOUSE
P49443	PPM1B_MOUSE	Protein phosphatase 1A OS=Mus musculus GN=Ppm1a PE=1 SV=1	1	10	0.99	1.43E+08	8.95E+07	8.64E+07	1.21E+08	1.20E+08	1.06E+08	1.03E+08	9.13E+07	1.17E+08	1.12E+08	9.79E+07	1.19E+08	1.11E+08	4.08E-01	0.003	0.001	0.006	0.012	0.002	0.005	0.002	0.008	0.001	0.005	0.008	0.030	3	10	10	PPM1B_MOUSE, SP:P36993	SP|P36993|PPM1B_MOUSE,SP|P49443|PPM1A_MOUSE
Q9CQ60	6PGL_MOUSE	6-phosphogluconolactonase OS=Mus musculus GN=Pgls PE=2 SV=1	3	13	1.0	4.22E+08	5.10E+08	4.33E+08	4.44E+08	5.85E+08	5.33E+08	4.85E+08	4.86E+08	4.81E+08	4.76E+08	4.13E+08	3.80E+08	4.30E+08	5.82E-06	1.29E-06	4.55E-06	2.35E-05	2.17E-04	8.43E-09	9.93E-06	3.44E-06	3.72E-04	7.68E-07	2.31E-03	1.97E-05	1.19E-01	3, 5	1, 13, 3	1, 13, 3	6PGL_MOUSE, SP:Q9CQ60	SP|Q9CQ60|6PGL_MOUSE
Q9CZ30	OLA1_MOUSE	Obg-like ATPase 1 OS=Mus musculus GN=Ola1 PE=1 SV=1	1	7	0.99	1.63E+07	5.55E+07	4.79E+07	3.65E+07	5.04E+07	4.85E+07	6.33E+07	6.06E+07	5.28E+07	5.03E+07	5.11E+07	7.23E+07	5.53E+07	0.128	0.022	0.047	0.053	0.062	0.012	0.020	0.026	0.052	0.015	0.022	0.025	0.092	3	7	7	OLA1_MOUSE, SP:Q9CZ30	SP|Q9CZ30|OLA1_MOUSE
Q9WUK2	IF4H_MOUSE	Eukaryotic translation initiation factor 4H OS=Mus musculus GN=Eif4h PE=1 SV=3	1	13	0.99	3.18E+08	3.91E+08	3.77E+08	4.20E+08	5.17E+08	4.14E+08	4.35E+08	4.22E+08	4.61E+08	4.14E+08	4.40E+08	3.86E+08	3.69E+08	3.22E-04	1.11E-05	9.46E-06	1.30E-04	3.98E-03	1.63E-06	4.72E-05	6.70E-06	1.81E-03	1.80E-07	1.79E-03	1.33E-04	3.17E-01	6	13	13	IF4H_MOUSE, SP:Q9WUK2	SP|Q9WUK2|IF4H_MOUSE
Q9R0Q6	ARC1A_MOUSE	Actin-related protein 2/3 complex subunit 1A OS=Mus musculus GN=Arpc1a PE=1 SV=1	1	6	1.0	2.85E+07	6.92E+07	4.67E+07	2.86E+07	2.63E+07	5.22E+07	4.61E+07	4.89E+07	4.22E+07	6.18E+07	4.88E+07	3.39E+07	1.93E+07	0.067	0.023	0.069	0.121	0.056	0.006	0.040	0.041	0.036	0.017	0.084	0.039	0.216	3	6	6	ARC1A_MOUSE, SP:Q9R0Q6	SP|Q9R0Q6|ARC1A_MOUSE
Q9QUR7	PIN1_MOUSE	Peptidyl-prolyl cis-trans isomerase NIMA-interacting 1 OS=Mus musculus GN=Pin1 PE=1 SV=1	1	2	0.99	7.01E+07	9.07E+07	1.05E+08	5.49E+07	3.48E+07	9.28E+07	7.22E+07	1.21E+08	7.35E+07	1.07E+08	8.50E+07	6.28E+07	6.49E+07	0.010	0.002	0.021	0.091	0.017	0.002	0.015	0.011	0.009	0.003	0.031	0.003	0.076	3	2	2	PIN1_MOUSE, SP:Q9QUR7	SP|Q9QUR7|PIN1_MOUSE
Q922B2	SYDC_MOUSE	Aspartyl-tRNA synthetase, cytoplasmic OS=Mus musculus GN=Dars PE=2 SV=1	1	2	0.96	1.21E+08	1.19E+08	8.40E+07	1.31E+08	1.60E+08	1.02E+08	1.07E+08	1.18E+08	1.18E+08	1.08E+08	9.74E+07	1.43E+08	1.46E+08	0.001	0.005	9.15E-01	0.002	0.014	5.55E-01	0.005	0.002	0.010	0.002	0.003	0.003	0.018	4	2	2	SYDC_MOUSE, SP:Q922B2	SP|Q922B2|SYDC_MOUSE
Q9JLR9	HIG1A_MOUSE	HIG1 domain family member 1A OS=Mus musculus GN=Higd1a PE=2 SV=1	1	13	0.99	1.45E+08	2.51E+08	2.14E+08	2.30E+08	2.61E+08	2.15E+08	2.33E+08	2.45E+08	2.05E+08	2.01E+08	2.01E+08	2.01E+08	2.48E+08	3.83E-01	1.16E-02	1.54E-02	1.24E-01	6.39E-01	9.32E-04	4.49E-02	7.11E-02	6.07E-01	1.04E-02	4.01E-01	4.37E-02	0.003	5	13	13	HIG1A_MOUSE, SP:Q9JLR9	SP|Q9JLR9|HIG1A_MOUSE
Q9D819	IPYR_MOUSE	Inorganic pyrophosphatase OS=Mus musculus GN=Ppa1 PE=1 SV=1	2	7	1.0	1.45E+08	1.95E+08	1.53E+08	1.47E+08	1.40E+08	1.39E+08	1.76E+08	1.53E+08	2.06E+08	1.51E+08	2.14E+08	1.15E+08	1.65E+08	4.32E-01	1.94E-01	4.99E-01	0.004	0.005	1.36E-02	3.81E-01	7.06E-02	0.003	5.91E-03	0.006	9.65E-01	0.012	3	3, 7	3, 7	IPYR_MOUSE, SP:Q9D819	SP|Q9D819|IPYR_MOUSE
Q8BK64	AHSA1_MOUSE	Activator of 90 kDa heat shock protein ATPase homolog 1 OS=Mus musculus GN=Ahsa1 PE=2 SV=2	1	4	1.0	1.02E+08	1.79E+08	1.53E+08	1.12E+08	1.69E+08	1.65E+08	1.71E+08	1.54E+08	1.82E+08	1.70E+08	1.54E+08	1.34E+08	1.17E+08	0.003	1.93E-01	0.002	0.002	0.002	4.14E-02	4.29E-01	1.92E-01	0.002	1.09E-01	0.003	9.69E-01	0.028	4	4	4	AHSA1_MOUSE, SP:Q8BK64	SP|Q8BK64|AHSA1_MOUSE
O54734	OST48_MOUSE	Dolichyl-diphosphooligosaccharide--protein glycosyltransferase 48 kDa subunit OS=Mus musculus GN=Ddost PE=1 SV=1	1	1	1.0	4.14E+07	7.83E+07	4.62E+07	5.08E+07	5.25E+07	6.88E+07	4.47E+07	7.40E+07	5.97E+07	4.50E+07	6.27E+07	4.39E+07	4.78E+07	0.036	0.024	0.025	0.050	0.034	0.004	0.043	0.020	0.063	0.009	0.058	0.015	0.109	2	1	1	OST48_MOUSE, SP:O54734	SP|O54734|OST48_MOUSE
Q99K48	NONO_MOUSE	Non-POU domain-containing octamer-binding protein OS=Mus musculus GN=Nono PE=1 SV=3	1	10	0.99	8.01E+07	7.94E+07	8.83E+07	7.52E+07	7.63E+07	9.40E+07	4.90E+07	8.43E+07	1.06E+08	7.69E+07	7.22E+07	7.03E+07	7.57E+07	0.006	0.003	0.008	0.023	0.016	0.004	0.036	0.003	0.022	0.005	0.023	0.010	0.060	3	10	10	NONO_MOUSE, SP:Q99K48	SP|Q99K48|NONO_MOUSE
P68037	UB2L3_MOUSE	Ubiquitin-conjugating enzyme E2 L3 OS=Mus musculus GN=Ube2l3 PE=2 SV=1	3	13	1.0	4.57E+08	5.64E+08	5.63E+08	4.36E+08	6.18E+08	5.75E+08	5.82E+08	5.50E+08	5.46E+08	5.74E+08	4.72E+08	5.51E+08	5.43E+08	1.15E-06	3.31E-09	4.70E-06	8.33E-06	6.79E-05	4.88E-10	3.18E-07	2.77E-07	2.32E-05	3.68E-08	1.46E-05	1.97E-06	1.69E-02	4, 5	12, 13, 5	12, 13, 5	UB2L3_MOUSE, SP:P68037	SP|P68037|UB2L3_MOUSE
P97372	PSME2_MOUSE	Proteasome activator complex subunit 2 OS=Mus musculus GN=Psme2 PE=2 SV=4	1	4	1.0	1.07E+08	1.33E+08	1.16E+08	1.37E+08	1.50E+08	1.24E+08	1.04E+08	1.59E+08	1.66E+08	1.26E+08	1.13E+08	1.25E+08	9.37E+07	0.003	0.002	9.08E-01	0.004	0.010	4.90E-01	0.008	6.51E-01	0.007	0.002	0.006	0.001	0.048	5	4	4	PSME2_MOUSE, SP:P97372	SP|P97372|PSME2_MOUSE
Q9CWJ9	PUR9_MOUSE	Bifunctional purine biosynthesis protein PURH OS=Mus musculus GN=Atic PE=1 SV=2	1	1	0.81	4.31E+07	5.09E+07	5.21E+07	4.68E+07	7.11E+07	3.37E+07	4.65E+07	4.29E+07	3.99E+07	4.11E+07	4.96E+07	4.25E+07	3.21E+07	0.032	0.018	0.029	0.027	0.100	0.015	0.039	0.046	0.071	0.017	0.061	0.049	0.156	3	1	1	PUR9_MOUSE, SP:Q9CWJ9	SP|Q9CWJ9|PUR9_MOUSE
Q9WV32	ARC1B_MOUSE	Actin-related protein 2/3 complex subunit 1B OS=Mus musculus GN=Arpc1b PE=1 SV=3	1	3	0.99	2.44E+07	3.19E+07	3.17E+07	2.27E+07	1.85E+07	2.50E+07	2.02E+07	4.64E+07	4.37E+07	4.90E+07	3.20E+07	3.59E+07	2.56E+07	0.083	0.051	0.094	0.163	0.135	0.044	0.124	0.039	0.055	0.044	0.078	0.043	0.183	2	3	3	ARC1B_MOUSE, SP:Q9WV32	SP|Q9WV32|ARC1B_MOUSE
Q99JX4	EIF3M_MOUSE	Eukaryotic translation initiation factor 3 subunit M OS=Mus musculus GN=Eif3m PE=2 SV=1	1	2	0.99	1.47E+07	2.78E+07	1.83E+07	1.34E+07	8.94E+06	8.62E+06	2.68E+07	2.00E+07	1.75E+07	1.65E+07	1.57E+07	1.56E+07	9.98E+06	0.141	0.106	0.155	0.248	0.258	0.055	0.091	0.132	0.176	0.115	0.174	0.130	0.286	2	2	2	EIF3M_MOUSE, SP:Q99JX4	SP|Q99JX4|EIF3M_MOUSE
Q9CQ89	CUTA_MOUSE	Protein CutA OS=Mus musculus GN=Cuta PE=2 SV=3	1	9	0.96	9.04E+07	1.52E+08	1.44E+08	8.89E+07	1.45E+08	1.77E+08	1.43E+08	1.41E+08	1.16E+08	1.41E+08	1.38E+08	9.67E+07	1.20E+08	0.004	2.70E-01	0.005	0.003	0.002	1.14E-01	0.001	0.002	0.003	2.37E-01	0.010	0.001	0.026	3	9	9	CUTA_MOUSE, SP:Q9CQ89	SP|Q9CQ89|CUTA_MOUSE
Q80UU9	PGRC2_MOUSE	Membrane-associated progesterone receptor component 2 OS=Mus musculus GN=Pgrmc2 PE=1 SV=2	1	1	0.99	3.37E+07	3.30E+07	1.80E+07	2.52E+07	3.52E+07	2.07E+07	1.60E+07	1.89E+07	3.09E+07	2.08E+07	2.03E+07	2.76E+07	2.01E+07	0.051	0.108	0.082	0.088	0.157	0.041	0.151	0.069	0.148	0.086	0.106	0.136	0.212	3	1	1	PGRC2_MOUSE, SP:Q80UU9	SP|Q80UU9|PGRC2_MOUSE
P17047	LAMP2_MOUSE	Lysosome-associated membrane glycoprotein 2 OS=Mus musculus GN=Lamp2 PE=2 SV=1	1	1	0.93	1.73E+07	2.45E+07	3.05E+07	1.40E+07	1.67E+07	2.52E+07	9.72E+06	1.51E+07	1.62E+07	1.74E+07	1.85E+07	1.88E+07	1.07E+07	0.121	0.054	0.150	0.176	0.134	0.067	0.211	0.141	0.170	0.097	0.152	0.163	0.279	2	1	1	LAMP2_MOUSE, SP:P17047	SP|P17047|LAMP2_MOUSE
P05977	MYL1_MOUSE	Myosin light chain 1/3, skeletal muscle isoform OS=Mus musculus GN=Myl1 PE=1 SV=2	1	9	1.0	9.36E+07	6.52E+07	6.97E+07	6.03E+07	1.00E+08	6.50E+07	6.86E+07	9.32E+07	1.04E+08	1.28E+08	1.34E+08	8.89E+07	1.18E+08	0.003	0.008	0.016	0.012	0.038	0.008	0.017	0.003	0.005	2.56E-01	0.013	0.008	0.027	2	9	9	MYL1_MOUSE, SP:P05977	SP|P05977|MYL1_MOUSE
Q99MR9	PPR3A_MOUSE	Protein phosphatase 1 regulatory subunit 3A OS=Mus musculus GN=Ppp1r3a PE=1 SV=1	2	8	1.0	6.42E+07	5.73E+07	6.30E+07	9.74E+07	7.11E+07	8.19E+07	5.96E+07	6.00E+07	4.00E+07	5.97E+07	5.07E+07	7.97E+07	5.02E+07	0.013	0.011	0.003	0.027	0.023	0.011	0.023	0.046	0.038	0.016	0.017	0.028	0.104	2, 3	1, 8	1, 8	PPR3A_MOUSE, SP:Q99MR9	SP|Q99MR9|PPR3A_MOUSE
P61750	ARF4_MOUSE	ADP-ribosylation factor 4 OS=Mus musculus GN=Arf4 PE=2 SV=2	1	2	1.0	5.43E+07	4.70E+07	3.20E+07	4.51E+07	2.86E+07	4.04E+07	4.25E+07	6.00E+07	5.48E+07	4.22E+07	6.22E+07	4.78E+07	4.16E+07	0.020	0.050	0.032	0.111	0.080	0.019	0.047	0.024	0.069	0.009	0.050	0.025	0.125	4	2	2	ARF4_MOUSE, SP:P61750	SP|P61750|ARF4_MOUSE
Q91V12	BACH_MOUSE	Cytosolic acyl coenzyme A thioester hydrolase OS=Mus musculus GN=Acot7 PE=1 SV=2	2	5	1.0	1.87E+08	2.57E+08	2.08E+08	1.55E+08	1.78E+08	2.04E+08	1.82E+08	2.05E+08	1.53E+08	1.91E+08	1.84E+08	1.44E+08	1.50E+08	6.82E-02	1.53E-02	3.09E-01	0.001	8.59E-01	7.24E-04	2.70E-01	5.00E-01	8.13E-01	2.38E-02	0.002	1.63E-01	0.015	2, 4	4, 5	4, 5	BACH_MOUSE, SP:Q91V12	SP|Q91V12|BACH_MOUSE
Q60865	CAPR1_MOUSE	Caprin-1 OS=Mus musculus GN=Caprin1 PE=1 SV=2	1	1	0.98	3.17E+07	4.24E+07	3.72E+07	3.88E+07	2.66E+07	1.37E+07	2.81E+07	5.28E+07	4.09E+07	3.66E+07	2.91E+07	4.15E+07	2.43E+07	0.057	0.038	0.042	0.120	0.207	0.024	0.086	0.044	0.083	0.051	0.063	0.033	0.189	3	1	1	CAPR1_MOUSE, SP:Q60865	SP|Q60865|CAPR1_MOUSE
Q99L47	F10A1_MOUSE	Hsc70-interacting protein OS=Mus musculus GN=St13 PE=2 SV=1	1	13	1.0	1.18E+08	1.19E+08	1.15E+08	1.45E+08	1.53E+08	1.10E+08	1.27E+08	9.97E+07	1.32E+08	1.21E+08	1.19E+08	1.09E+08	1.31E+08	0.001	9.45E-01	4.60E-01	0.002	0.010	5.18E-01	0.002	0.001	0.006	5.30E-01	0.007	0.006	0.021	2	12	13	F10A1_MOUSE, SP:Q99L47	SP|Q99L47|F10A1_MOUSE
Q4VAA2	CDV3_MOUSE	Protein CDV3 OS=Mus musculus GN=Cdv3 PE=1 SV=2	2	8	1.0	1.15E+08	8.82E+07	1.18E+08	1.16E+08	1.62E+08	1.45E+08	1.58E+08	1.12E+08	1.66E+08	1.64E+08	1.65E+08	1.72E+08	2.18E+08	0.001	8.43E-01	0.001	0.002	0.004	0.002	6.23E-01	2.97E-01	0.002	5.57E-02	9.55E-01	0.004	0.004	3, 4	2, 8	2, 8	CDV3_MOUSE, SP:Q4VAA2	SP|Q4VAA2|CDV3_MOUSE
O35343	IMA4_MOUSE	Importin subunit alpha-4 OS=Mus musculus GN=Kpna4 PE=2 SV=1	1	13	0.99	8.87E+07	8.72E+07	8.51E+07	8.03E+07	1.08E+08	1.04E+08	6.66E+07	1.21E+08	1.05E+08	9.17E+07	9.78E+07	1.06E+08	1.27E+08	0.004	0.004	0.007	0.009	0.012	0.002	0.018	0.003	0.014	0.001	0.007	0.003	0.022	5	13	13	IMA4_MOUSE, SP:O35343	SP|O35343|IMA4_MOUSE
P62843	RS15_MOUSE	40S ribosomal protein S15 OS=Mus musculus GN=Rps15 PE=2 SV=2	1	7	0.99	1.88E+08	1.54E+08	1.63E+08	1.47E+08	1.00E+08	9.76E+07	1.44E+08	1.96E+08	2.20E+08	1.46E+08	3.12E+08	1.69E+08	8.67E+07	7.27E-02	1.18E-01	4.60E-01	0.012	0.016	1.07E-01	0.001	4.41E-02	0.003	8.24E-05	0.001	2.38E-01	0.049	6	7	7	RS15_MOUSE, SP:P62843	SP|P62843|RS15_MOUSE
P15626	GSTM2_MOUSE	Glutathione S-transferase Mu 2 OS=Mus musculus GN=Gstm2 PE=1 SV=2	3	13	1.0	8.94E+08	1.24E+09	1.17E+09	9.24E+08	8.57E+08	1.18E+09	1.22E+09	8.62E+08	9.02E+08	8.11E+08	1.11E+09	1.14E+09	9.00E+08	9.56E-14	1.92E-19	1.16E-13	2.92E-08	5.71E-11	3.26E-22	1.02E-15	2.79E-12	7.57E-08	1.24E-19	2.43E-12	2.50E-10	6.22E-05	3, 5	11, 13, 2	11, 13, 2	GSTM2_MOUSE, SP:P15626	SP|P15626|GSTM2_MOUSE
P63280	UBC9_MOUSE	SUMO-conjugating enzyme UBC9 OS=Mus musculus GN=Ube2i PE=1 SV=1	1	3	0.93	2.65E+07	3.01E+07	3.48E+07	3.16E+07	2.31E+07	2.69E+07	3.52E+07	3.17E+07	4.61E+07	2.53E+07	3.19E+07	1.86E+07	1.63E+07	0.074	0.043	0.060	0.136	0.126	0.048	0.063	0.035	0.125	0.044	0.153	0.078	0.235	2	3	3	UBC9_MOUSE, SP:P63280	SP|P63280|UBC9_MOUSE
P42669	PURA_MOUSE	Transcriptional activator protein Pur-alpha OS=Mus musculus GN=Pura PE=1 SV=1	2	13	1.0	2.95E+08	4.66E+08	3.26E+08	2.74E+08	3.48E+08	4.36E+08	3.33E+08	4.47E+08	3.43E+08	3.71E+08	3.75E+08	2.92E+08	3.25E+08	1.07E-03	1.58E-04	3.26E-03	1.28E-02	2.91E-03	1.08E-07	2.00E-03	5.76E-04	6.77E-03	6.12E-06	2.98E-02	7.75E-05	6.96E-01	3, 4	13, 7	13, 7	PURA_MOUSE, SP:P42669	SP|P42669|PURA_MOUSE
Q9QVP4	MLRA_MOUSE	Myosin regulatory light chain 2, atrial isoform OS=Mus musculus GN=Myl7 PE=2 SV=1	5	10	1.0	2.70E+09	2.09E+09	5.57E+08	1.60E+09	1.89E+09	1.28E+09	4.87E+08	6.73E+08	7.89E+08	3.86E+08	1.79E+09	3.46E+08	1.87E+09	0	2.30E-08	2.14E-24	9.70E-20	3.40E-12	1.37E-38	3.35E-05	2.02E-10	6.70E-03	1.58E-32	1.01E-02	1.26E-07	9.15E-12	2, 3, 4, 4, 4, 5, 4, 6	1, 1, 7, 3, 6, 10	1, 1, 7, 10, 6, 3	MLRA_MOUSE, SP:Q9QVP4	SP|Q9QVP4|MLRA_MOUSE
Q922J3	CLIP1_MOUSE	CAP-Gly domain-containing linker protein 1 OS=Mus musculus GN=Clip1 PE=1 SV=1	1	5	1.0	1.84E+07	1.10E+07	1.53E+07	1.70E+07	2.32E+07	1.77E+07	1.54E+07	9.16E+06	1.98E+07	2.48E+07	1.73E+07	1.87E+07	2.27E+07	0.114	0.127	0.127	0.136	0.176	0.158	0.156	0.118	0.127	0.104	0.152	0.223	0.198	2	5	5	CLIP1_MOUSE, SP:Q922J3	SP|Q922J3|CLIP1_MOUSE
Q9JI75	NQO2_MOUSE	Ribosyldihydronicotinamide dehydrogenase [quinone] OS=Mus musculus GN=Nqo2 PE=2 SV=3	2	12	1.0	3.73E+08	4.13E+08	3.06E+08	3.03E+08	4.41E+08	3.72E+08	3.48E+08	3.30E+08	2.96E+08	2.92E+08	2.89E+08	2.85E+08	2.47E+08	3.27E-05	2.12E-04	8.10E-04	9.01E-04	1.09E-02	4.89E-07	8.27E-04	2.40E-03	4.79E-02	1.61E-04	3.20E-02	2.46E-03	0.003	4, 6	12, 6	12, 6	NQO2_MOUSE, SP:Q9JI75	SP|Q9JI75|NQO2_MOUSE
P43024	CX6A1_MOUSE	Cytochrome c oxidase subunit 6A1, mitochondrial OS=Mus musculus GN=Cox6a1 PE=1 SV=2	1	13	1.0	1.06E+09	8.51E+08	8.14E+08	8.30E+08	8.17E+08	8.68E+08	7.46E+08	9.17E+08	1.09E+09	8.90E+08	8.96E+08	8.49E+08	6.89E+08	2.50E-16	5.66E-13	6.06E-12	6.97E-08	6.66E-08	1.02E-14	3.68E-09	3.96E-15	9.70E-09	1.70E-15	5.48E-09	4.95E-11	1.60E-03	5, 7	13	13	CX6A1_MOUSE, SP:P43024	SP|P43024|CX6A1_MOUSE
P05132	KAPCA_MOUSE	cAMP-dependent protein kinase catalytic subunit alpha OS=Mus musculus GN=Prkaca PE=1 SV=3	1	2	0.99	5.08E+07	1.15E+08	1.14E+08	5.74E+07	9.62E+07	7.09E+07	9.07E+07	8.94E+07	6.36E+07	6.34E+07	6.98E+07	5.71E+07	4.83E+07	0.108	0.020	0.078	0.045	0.074	0.012	0.051	0.104	0.107	0.062	0.088	0.034	0.131	4	2	2	KAPCA_MOUSE, SP:P05132	SP|P05132|KAPCA_MOUSE
Q9WV03	FA50A_MOUSE	Protein FAM50A OS=Mus musculus GN=Fam50a PE=2 SV=1	1	3	0.93	1.53E+08	2.66E+08	2.36E+08	1.25E+08	2.87E+08	2.17E+08	2.01E+08	2.15E+08	2.32E+08	2.68E+08	3.84E+08	2.12E+08	2.49E+08	2.65E-01	4.10E-03	9.83E-01	5.83E-02	5.86E-01	4.41E-04	1.34E-01	2.49E-02	9.14E-02	2.09E-06	2.84E-01	1.13E-01	0.002	6	3	3	FA50A_MOUSE, SP:Q9WV03	SP|Q9WV03|FA50A_MOUSE
Q91Z53	GRHPR_MOUSE	Glyoxylate reductase/hydroxypyruvate reductase OS=Mus musculus GN=Grhpr PE=1 SV=1	4	9	1.0	4.77E+08	5.41E+08	5.04E+08	5.74E+08	6.35E+08	5.32E+08	5.17E+08	4.92E+08	5.04E+08	5.23E+08	5.39E+08	4.56E+08	5.62E+08	1.08E-06	1.09E-07	6.38E-08	8.61E-06	2.74E-04	3.64E-09	5.28E-06	2.29E-06	1.28E-04	4.17E-09	3.04E-04	2.02E-05	1.43E-02	2, 3	1, 2, 4, 9	1, 2, 4, 9	GRHPR_MOUSE, SP:Q91Z53	SP|Q91Z53|GRHPR_MOUSE
Q8R1G2	CMBL_MOUSE	Carboxymethylenebutenolidase homolog OS=Mus musculus GN=Cmbl PE=2 SV=1	2	11	1.0	3.94E+08	3.96E+08	3.73E+08	4.03E+08	5.07E+08	4.64E+08	3.54E+08	3.26E+08	4.07E+08	4.05E+08	3.30E+08	3.93E+08	3.26E+08	2.22E-05	3.05E-05	2.56E-05	1.87E-04	1.48E-03	3.20E-06	1.16E-03	7.23E-05	2.79E-03	4.37E-05	1.97E-03	4.96E-03	6.88E-01	3, 5	11, 8	11, 8	CMBL_MOUSE, SP:Q8R1G2	SP|Q8R1G2|CMBL_MOUSE
O35114	SCRB2_MOUSE	Lysosome membrane protein 2 OS=Mus musculus GN=Scarb2 PE=1 SV=3	1	4	0.99	6.75E+07	6.52E+07	5.11E+07	7.30E+07	7.29E+07	7.02E+07	5.82E+07	8.93E+07	7.50E+07	6.52E+07	6.33E+07	7.17E+07	6.33E+07	0.011	0.019	0.009	0.026	0.033	0.008	0.025	0.011	0.033	0.008	0.023	0.009	0.079	4	4	4	SCRB2_MOUSE, SP:O35114	SP|O35114|SCRB2_MOUSE
P56812	PDCD5_MOUSE	Programmed cell death protein 5 OS=Mus musculus GN=Pdcd5 PE=1 SV=3	1	6	0.93	8.32E+07	7.38E+07	5.88E+07	5.49E+07	6.15E+07	7.39E+07	5.09E+07	5.31E+07	6.76E+07	6.84E+07	7.43E+07	6.86E+07	7.52E+07	0.006	0.015	0.022	0.038	0.031	0.006	0.035	0.016	0.031	0.005	0.026	0.036	0.062	2	6	6	PDCD5_MOUSE, SP:P56812	SP|P56812|PDCD5_MOUSE
O88746	TOM1_MOUSE	Target of Myb protein 1 OS=Mus musculus GN=Tom1 PE=1 SV=1	2	2	1.0	1.02E+08	1.18E+08	9.38E+07	7.94E+07	1.05E+08	1.02E+08	1.34E+08	1.31E+08	1.40E+08	1.00E+08	9.27E+07	1.10E+08	1.12E+08	0.002	0.002	0.007	0.010	0.013	5.32E-01	0.001	8.24E-01	0.011	0.002	0.007	0.002	0.029	3	1, 2	1, 2	TOM1_MOUSE, SP:O88746	SP|O88746|TOM1_MOUSE
P28656	NP1L1_MOUSE	Nucleosome assembly protein 1-like 1 OS=Mus musculus GN=Nap1l1 PE=1 SV=2	1	3	0.99	7.96E+07	1.35E+08	1.06E+08	6.27E+07	7.90E+07	1.18E+08	9.88E+07	1.34E+08	1.22E+08	1.03E+08	1.45E+08	8.11E+07	8.26E+07	0.006	0.002	0.015	0.022	0.009	2.75E-01	0.006	0.002	0.010	1.58E-01	0.017	0.002	0.053	3	3	3	NP1L1_MOUSE, SP:P28656	SP|P28656|NP1L1_MOUSE
O55028	BCKD_MOUSE	[3-methyl-2-oxobutanoate dehydrogenase [lipoamide]] kinase, mitochondrial OS=Mus musculus GN=Bckdk PE=1 SV=1	2	13	1.0	1.96E+08	1.74E+08	1.68E+08	2.39E+08	1.94E+08	1.99E+08	1.95E+08	1.64E+08	2.01E+08	2.02E+08	1.92E+08	2.39E+08	1.83E+08	5.76E-02	1.70E-01	1.39E-02	8.67E-01	0.001	4.81E-02	2.40E-01	9.70E-02	6.44E-01	2.25E-02	1.59E-01	7.18E-01	0.008	4	13, 7	13, 7	BCKD_MOUSE, SP:O55028	SP|O55028|BCKD_MOUSE
O35215	DOPD_MOUSE	D-dopachrome decarboxylase OS=Mus musculus GN=Ddt PE=1 SV=3	3	13	1.0	5.27E+08	6.43E+08	8.35E+08	5.44E+08	6.08E+08	6.89E+08	5.07E+08	5.37E+08	4.82E+08	5.18E+08	5.65E+08	6.15E+08	5.83E+08	8.94E-08	4.58E-14	8.30E-08	1.23E-05	4.69E-06	1.79E-11	4.48E-06	3.16E-06	1.16E-04	6.53E-10	2.47E-06	3.43E-06	8.85E-03	4	11, 13, 3	11, 13, 3	DOPD_MOUSE, SP:O35215	SP|O35215|DOPD_MOUSE
P62849	RS24_MOUSE	40S ribosomal protein S24 OS=Mus musculus GN=Rps24 PE=1 SV=1	2	9	1.0	1.13E+08	1.77E+08	2.05E+08	1.11E+08	1.61E+08	1.93E+08	1.53E+08	1.66E+08	1.65E+08	1.63E+08	1.75E+08	1.33E+08	1.40E+08	0.001	1.84E-02	0.002	0.002	0.001	3.64E-02	7.76E-01	3.37E-01	0.002	4.03E-02	0.003	6.36E-01	0.018	3, 4	5, 9	5, 9	RS24_MOUSE, SP:P62849	SP|P62849|RS24_MOUSE
P62267	RS23_MOUSE	40S ribosomal protein S23 OS=Mus musculus GN=Rps23 PE=2 SV=3	1	13	0.99	1.13E+08	1.56E+08	1.64E+08	1.41E+08	1.33E+08	1.59E+08	1.04E+08	1.72E+08	1.30E+08	1.61E+08	1.55E+08	1.35E+08	1.36E+08	0.002	1.23E-01	5.72E-01	0.005	0.003	9.14E-02	0.004	0.001	0.002	1.03E-01	0.003	5.24E-01	0.019	3	13	13	RS23_MOUSE, SP:P62267	SP|P62267|RS23_MOUSE
Q3UHA3	SPTCS_MOUSE	Spatacsin OS=Mus musculus GN=Spg11 PE=2 SV=2	1	1	0.99	1.65E+08	9.68E+06	5.04E+07	8.35E+07	4.87E+08	1.64E+07	1.26E+08	1.77E+07	2.33E+07	7.27E+07	9.07E+06	6.39E+07	1.29E+08	3.79E-01	0.051	0.012	8.44E-04	0.343	0.314	0.005	0.182	0.047	0.297	0.071	0.245	0.038	3	1	1	SPTCS_MOUSE, SP:Q3UHA3	SP|Q3UHA3|SPTCS_MOUSE
Q02357	ANK1_MOUSE	Ankyrin-1 OS=Mus musculus GN=Ank1 PE=1 SV=2	1	10	0.98	2.47E+09	1.48E+09	1.15E+09	1.83E+09	1.16E+09	1.20E+09	2.50E+09	9.94E+08	1.51E+09	1.40E+09	9.17E+08	1.85E+09	1.24E+09	2.20E-34	2.61E-18	1.34E-25	3.32E-11	6.57E-11	8.71E-25	1.03E-29	5.04E-20	8.30E-14	9.00E-16	2.56E-19	7.38E-12	3.95E-07	4	10	10	ANK1_MOUSE, SP:Q02357	SP|Q02357|ANK1_MOUSE
Q8R1Q8	DC1L1_MOUSE	Cytoplasmic dynein 1 light intermediate chain 1 OS=Mus musculus GN=Dync1li1 PE=1 SV=1	1	1	1.0	3.13E+07	4.94E+07	4.38E+07	3.13E+07	5.69E+07	3.95E+07	3.19E+07	4.52E+07	4.20E+07	4.32E+07	4.79E+07	4.99E+07	5.09E+07	0.058	0.027	0.062	0.043	0.083	0.017	0.073	0.042	0.067	0.019	0.047	0.045	0.102	2	1	1	DC1L1_MOUSE, SP:Q8R1Q8	SP|Q8R1Q8|DC1L1_MOUSE
Q91VK1	BZW2_MOUSE	Basic leucine zipper and W2 domain-containing protein 2 OS=Mus musculus GN=Bzw2 PE=1 SV=1	1	1	0.99	4.13E+07	3.74E+07	2.53E+07	3.16E+07	2.00E+07	3.02E+07	2.63E+07	2.62E+07	2.28E+07	1.87E+07	3.41E+07	2.36E+07	8.21E+06	0.036	0.071	0.060	0.154	0.112	0.032	0.093	0.101	0.161	0.039	0.124	0.098	0.305	4	1	1	BZW2_MOUSE, SP:Q91VK1	SP|Q91VK1|BZW2_MOUSE
Q99LT0	DPY30_MOUSE	Protein dpy-30 homolog OS=Mus musculus GN=Dpy30 PE=2 SV=1	1	8	0.99	7.29E+07	6.63E+07	5.58E+07	6.04E+07	9.31E+07	7.19E+07	5.64E+07	7.82E+07	7.47E+07	6.52E+07	6.00E+07	6.04E+07	7.31E+07	0.008	0.015	0.016	0.014	0.031	0.007	0.027	0.011	0.032	0.010	0.033	0.013	0.064	3	8	8	DPY30_MOUSE, SP:Q99LT0	SP|Q99LT0|DPY30_MOUSE
Q02248	CTNB1_MOUSE	Catenin beta-1 OS=Mus musculus GN=Ctnnb1 PE=1 SV=1	1	6	1.0	9.43E+07	1.47E+08	1.12E+08	1.10E+08	1.16E+08	1.10E+08	1.01E+08	1.73E+08	1.35E+08	1.14E+08	1.43E+08	1.49E+08	1.29E+08	0.004	0.001	0.002	0.008	0.011	1.64E-01	0.005	0.001	0.008	2.00E-01	0.002	5.43E-01	0.022	5	6	6	CTNB1_MOUSE, SP:Q02248	SP|Q02248|CTNB1_MOUSE
Q9CZ04	CSN7A_MOUSE	COP9 signalosome complex subunit 7a OS=Mus musculus GN=Cops7a PE=1 SV=2	1	1	0.93	4.20E+07	6.32E+07	5.69E+07	5.26E+07	6.43E+07	7.02E+07	6.43E+07	8.48E+07	6.67E+07	6.70E+07	6.80E+07	6.92E+07	5.54E+07	0.035	0.014	0.023	0.034	0.032	0.008	0.020	0.015	0.031	0.007	0.025	0.010	0.092	3	1	1	CSN7A_MOUSE, SP:Q9CZ04	SP|Q9CZ04|CSN7A_MOUSE
Q3THW5	H2AZ_MOUSE	Histone H2A.V OS=Mus musculus GN=H2afv PE=1 SV=3	1	1	0.81	1.21E+08	1.22E+08	1.20E+08	9.14E+07	1.58E+08	9.44E+07	8.40E+07	1.18E+08	9.10E+07	1.14E+08	8.17E+07	1.09E+08	1.01E+08	0.001	0.001	0.005	0.002	0.018	5.70E-01	0.011	0.007	0.008	0.004	0.008	0.004	0.039	2	1	1	H2AZ_MOUSE, SP:P0C0S6	SP|P0C0S6|H2AZ_MOUSE,SP|Q3THW5|H2AV_MOUSE
O54950	AAKG1_MOUSE	5'-AMP-activated protein kinase subunit gamma-1 OS=Mus musculus GN=Prkag1 PE=2 SV=2	1	5	1.0	1.07E+08	1.36E+08	1.02E+08	1.23E+08	1.37E+08	1.49E+08	1.28E+08	1.38E+08	1.18E+08	1.04E+08	1.21E+08	1.26E+08	1.11E+08	0.002	0.002	0.001	0.004	0.004	2.88E-01	0.002	0.002	0.011	6.72E-01	0.004	0.002	0.031	3	2, 5	2, 5	AAKG1_MOUSE, SP:O54950	SP|O54950|AAKG1_MOUSE
Q9DBU0	TM9S1_MOUSE	Transmembrane 9 superfamily member 1 OS=Mus musculus GN=Tm9sf1 PE=2 SV=2	1	1	0.72	6.85E+07	1.43E+08	7.79E+07	2.17E+07	1.91E+08	3.41E+08	3.37E+07	1.85E+08	1.23E+08	3.65E+07	1.57E+08	2.72E+07	5.98E+07	0.011	0.006	0.107	8.56E-01	2.50E-02	1.69E-01	0.072	0.002	0.088	8.93E-02	0.117	3.33E-01	0.086	5	1	1	TM9S1_MOUSE, SP:Q9DBU0	SP|Q9DBU0|TM9S1_MOUSE
Q9CQX8	RT36_MOUSE	28S ribosomal protein S36, mitochondrial OS=Mus musculus GN=Mrps36 PE=1 SV=1	2	12	1.0	2.18E+09	2.33E+09	2.27E+09	2.34E+09	2.04E+09	1.86E+09	1.90E+09	1.92E+09	1.85E+09	2.33E+09	2.11E+09	1.87E+09	1.49E+09	3.87E-33	9.67E-37	1.73E-34	8.54E-21	8.60E-18	2.16E-40	9.25E-25	6.24E-26	6.13E-24	5.50E-37	1.20E-20	7.09E-24	5.70E-09	4, 5	12, 3	12, 3	RT36_MOUSE, SP:Q9CQX8	SP|Q9CQX8|RT36_MOUSE
Q8WTY4	CPIN1_MOUSE	Anamorsin OS=Mus musculus GN=Ciapin1 PE=2 SV=1	1	2	0.98	1.61E+08	1.41E+08	1.44E+08	1.79E+08	2.75E+08	1.24E+08	1.46E+08	1.33E+08	2.90E+08	1.57E+08	1.33E+08	1.57E+08	1.14E+08	2.06E-01	2.69E-01	1.22E-01	8.91E-02	0.007	1.92E-01	0.001	3.27E-03	0.002	2.90E-01	0.002	0.002	0.028	6	2	2	CPIN1_MOUSE, SP:Q8WTY4	SP|Q8WTY4|CPIN1_MOUSE
Q8VBT3	OSCAR_MOUSE	Osteoclast-associated immunoglobulin-like receptor OS=Mus musculus GN=Oscar PE=2 SV=1	1	2	0.93	2.87E+08	6.75E+07	1.87E+07	1.50E+07	2.68E+07	2.40E+07	1.25E+07	7.26E+08	2.71E+07	1.88E+07	1.56E+07	2.64E+07	2.29E+07	1.18E-03	0.113	0.157	0.124	0.146	0.007	0.197	0.088	0.168	0.134	0.117	1.15E-08	0.202	5	2	2	OSCAR_MOUSE, SP:Q8VBT3	SP|Q8VBT3|OSCAR_MOUSE
Q9DCS3	MECR_MOUSE	Trans-2-enoyl-CoA reductase, mitochondrial OS=Mus musculus GN=Mecr PE=2 SV=2	1	2	0.99	1.28E+08	1.58E+08	1.35E+08	1.89E+08	1.72E+08	1.53E+08	1.53E+08	1.55E+08	1.42E+08	1.27E+08	1.46E+08	1.41E+08	1.15E+08	7.66E-01	3.60E-01	7.46E-02	0.001	0.003	7.27E-02	7.24E-01	7.57E-01	0.005	1.33E-01	0.002	8.53E-01	0.028	6	2	2	MECR_MOUSE, SP:Q9DCS3	SP|Q9DCS3|MECR_MOUSE
P61211	ARL1_MOUSE	ADP-ribosylation factor-like protein 1 OS=Mus musculus GN=Arl1 PE=2 SV=1	1	4	1.0	3.50E+07	6.52E+07	5.35E+07	4.45E+07	4.59E+07	5.16E+07	4.36E+07	7.18E+07	6.09E+07	5.51E+07	7.60E+07	6.21E+07	5.16E+07	0.051	0.018	0.035	0.063	0.058	0.008	0.047	0.019	0.046	0.005	0.032	0.017	0.101	2, 3	1, 4	1, 4	ARL1_MOUSE, SP:P61211	SP|P61211|ARL1_MOUSE
Q60850	S23A3_MOUSE	Solute carrier family 23 member 3 OS=Mus musculus GN=Slc23a3 PE=2 SV=1	1	3	0.98	1.12E+08	9.08E+07	5.77E+07	1.55E+08	7.21E+07	1.18E+08	1.04E+08	1.05E+08	1.58E+08	1.50E+08	1.19E+08	1.15E+08	1.02E+08	0.001	0.014	2.93E-01	0.027	0.008	0.002	0.004	3.96E-01	0.003	5.09E-01	0.005	0.005	0.036	5	3	3	S23A3_MOUSE, SP:Q60850	SP|Q60850|S23A3_MOUSE
Q91YS8	KCC1A_MOUSE	Calcium/calmodulin-dependent protein kinase type 1 OS=Mus musculus GN=Camk1 PE=1 SV=1	1	1	0.98	2.41E+07	3.95E+07	3.50E+07	2.06E+07	2.12E+07	3.93E+07	3.76E+07	3.34E+07	3.24E+07	2.76E+07	3.24E+07	1.46E+07	1.41E+07	0.084	0.043	0.105	0.148	0.084	0.029	0.057	0.064	0.115	0.043	0.183	0.073	0.252	2	1	1	KCC1A_MOUSE, SP:Q91YS8	SP|Q91YS8|KCC1A_MOUSE
Q6P1F6	2ABA_MOUSE	Serine/threonine-protein phosphatase 2A 55 kDa regulatory subunit B alpha isoform OS=Mus musculus GN=Ppp2r2a PE=1 SV=1	1	8	0.99	6.40E+07	4.65E+07	4.54E+07	6.49E+07	8.04E+07	4.09E+07	6.14E+07	4.92E+07	6.15E+07	5.39E+07	4.04E+07	7.90E+07	5.02E+07	0.013	0.025	0.013	0.021	0.080	0.020	0.022	0.018	0.047	0.028	0.018	0.039	0.103	2	8	8	2ABA_MOUSE, SP:Q6P1F6	SP|Q6P1F6|2ABA_MOUSE
Q99LD4	CSN1_MOUSE	COP9 signalosome complex subunit 1 OS=Mus musculus GN=Gps1 PE=1 SV=1	1	1	1.0	3.63E+07	8.61E+07	7.49E+07	5.53E+07	3.03E+07	6.95E+07	4.53E+07	5.96E+07	6.82E+07	8.76E+07	8.57E+07	4.98E+07	4.14E+07	0.047	0.007	0.021	0.108	0.034	0.003	0.043	0.014	0.017	0.003	0.049	0.027	0.127	3	1	1	CSN1_MOUSE, SP:Q99LD4	SP|Q99LD4|CSN1_MOUSE
Q5F285	CQ061_MOUSE	UPF0451 protein C17orf61 homolog OS=Mus musculus PE=2 SV=1	1	5	0.99	7.99E+07	1.36E+08	1.28E+08	8.89E+07	6.35E+08	1.19E+08	9.80E+07	1.10E+08	7.71E+07	1.07E+08	1.28E+08	9.33E+07	7.52E+07	0.006	5.36E-01	0.005	0.035	0.008	2.27E-01	0.006	0.010	0.009	3.49E-01	0.011	0.004	0.061	4	5	5	CQ061_MOUSE, SP:Q5F285	SP|Q5F285|CQ061_MOUSE
O88491	NSD1_MOUSE	Histone-lysine N-methyltransferase, H3 lysine-36 and H4 lysine-20 specific OS=Mus musculus GN=Nsd1 PE=1 SV=1	1	7	0.66	2.40E+08	2.57E+08	2.43E+08	3.94E+08	2.59E+08	2.69E+08	3.18E+08	2.55E+08	2.55E+08	2.58E+08	3.00E+08	2.90E+08	1.99E+08	9.64E-03	4.17E-03	3.96E-05	1.55E-01	1.75E-01	1.04E-03	3.15E-03	1.38E-02	1.42E-01	1.48E-04	3.53E-02	3.56E-02	0.006	3	7	7	NSD1_MOUSE, SP:O88491	SP|O88491|NSD1_MOUSE
Q8VCI5	PEX19_MOUSE	Peroxisomal biogenesis factor 19 OS=Mus musculus GN=Pex19 PE=1 SV=1	1	3	0.99	9.67E+07	8.99E+07	7.00E+07	6.20E+07	6.95E+07	8.64E+07	6.68E+07	9.51E+07	1.15E+08	7.76E+07	1.03E+08	7.17E+07	6.34E+07	0.003	0.007	0.015	0.029	0.022	0.002	0.018	0.002	0.022	0.001	0.022	0.007	0.078	7	3	3	PEX19_MOUSE, SP:Q8VCI5	SP|Q8VCI5|PEX19_MOUSE
Q6ZQ73	CAND2_MOUSE	Cullin-associated NEDD8-dissociated protein 2 OS=Mus musculus GN=Cand2 PE=1 SV=2	2	12	1.0	1.30E+08	1.73E+08	1.70E+08	1.61E+08	1.59E+08	1.79E+08	1.44E+08	1.98E+08	1.81E+08	1.84E+08	1.73E+08	1.72E+08	1.82E+08	7.10E-01	8.09E-02	2.39E-01	0.002	0.002	3.73E-02	0.001	1.70E-01	9.79E-01	3.91E-02	9.52E-01	2.03E-01	0.008	2, 3, 4	1, 5, 12	1, 12, 5	CAND2_MOUSE, SP:Q6ZQ73	SP|Q6ZQ73|CAND2_MOUSE
P48774	GSTM5_MOUSE	Glutathione S-transferase Mu 5 OS=Mus musculus GN=Gstm5 PE=1 SV=1	2	13	1.0	1.30E+08	2.07E+08	1.76E+08	1.13E+08	1.43E+08	2.20E+08	1.56E+08	1.54E+08	1.32E+08	1.67E+08	1.63E+08	1.20E+08	1.19E+08	6.99E-01	6.12E-02	0.002	0.003	5.62E-01	8.06E-03	6.69E-01	0.001	0.002	6.25E-02	0.005	9.07E-01	0.026	2, 3	13, 6	13, 6	GSTM5_MOUSE, SP:P48774	SP|P48774|GSTM5_MOUSE
Q8VCR7	ABHEB_MOUSE	Abhydrolase domain-containing protein 14B OS=Mus musculus GN=Abhd14b PE=2 SV=1	2	5	1.0	4.86E+07	4.39E+07	5.05E+07	4.14E+07	3.97E+07	4.36E+07	6.53E+07	3.24E+07	4.09E+07	4.91E+07	3.95E+07	4.45E+07	3.88E+07	0.025	0.019	0.037	0.075	0.073	0.022	0.019	0.044	0.054	0.029	0.057	0.075	0.133	2	2, 5	2, 5	ABHEB_MOUSE, SP:Q8VCR7	SP|Q8VCR7|ABHEB_MOUSE
Q3ULJ0	GPD1L_MOUSE	Glycerol-3-phosphate dehydrogenase 1-like protein OS=Mus musculus GN=Gpd1l PE=1 SV=2	1	13	1.0	1.87E+08	1.39E+08	1.23E+08	1.53E+08	1.83E+08	1.41E+08	1.42E+08	1.77E+08	1.38E+08	1.48E+08	1.08E+08	1.28E+08	1.20E+08	6.48E-02	6.34E-01	3.24E-01	0.001	0.005	1.85E-01	0.001	8.85E-01	0.003	8.58E-01	0.004	4.14E-01	0.026	4	13	13	GPD1L_MOUSE, SP:Q3ULJ0	SP|Q3ULJ0|GPD1L_MOUSE
Q9WV35	ABEC2_MOUSE	Probable C->U-editing enzyme APOBEC-2 OS=Mus musculus GN=Apobec2 PE=1 SV=1	3	8	1.0	1.09E+09	1.09E+09	1.05E+09	8.17E+08	1.28E+09	1.18E+09	9.45E+08	1.12E+09	9.66E+08	8.59E+08	8.52E+08	9.31E+08	7.83E+08	2.69E-17	6.12E-18	2.42E-12	3.23E-13	2.81E-11	3.52E-20	2.17E-12	1.06E-13	1.34E-08	1.34E-15	3.46E-10	3.49E-14	3.30E-04	2, 4, 3, 4	4, 5, 6, 8	4, 5, 6, 8	ABEC2_MOUSE, SP:Q9WV35	SP|Q9WV35|ABEC2_MOUSE
P97927	LAMA4_MOUSE	Laminin subunit alpha-4 OS=Mus musculus GN=Lama4 PE=1 SV=2	1	13	1.0	9.20E+07	1.39E+08	8.83E+07	8.15E+07	6.73E+07	1.15E+08	7.91E+07	9.27E+07	1.25E+08	1.18E+08	1.13E+08	7.89E+07	8.19E+07	0.004	0.004	0.007	0.035	0.010	2.68E-01	0.013	0.002	0.007	8.53E-01	0.019	0.009	0.055	3	13	13	LAMA4_MOUSE, SP:P97927	SP|P97927|LAMA4_MOUSE
Q9Z2Y8	PROSC_MOUSE	Proline synthetase co-transcribed bacterial homolog protein OS=Mus musculus GN=Prosc PE=1 SV=1	2	13	1.0	1.75E+08	2.51E+08	2.18E+08	2.12E+08	2.37E+08	2.65E+08	2.49E+08	2.07E+08	1.86E+08	2.19E+08	2.15E+08	1.98E+08	2.47E+08	1.08E-01	9.27E-03	2.94E-02	2.30E-01	1.71E-01	9.88E-04	2.55E-02	1.42E-01	3.63E-01	5.32E-03	4.36E-01	1.50E-01	0.003	2, 4	10, 13	10, 13	PROSC_MOUSE, SP:Q9Z2Y8	SP|Q9Z2Y8|PROSC_MOUSE
Q64152	BTF3_MOUSE	Transcription factor BTF3 OS=Mus musculus GN=Btf3 PE=2 SV=3	1	4	0.99	9.33E+07	1.07E+08	1.02E+08	1.20E+08	1.63E+08	9.57E+07	1.04E+08	1.41E+08	1.27E+08	9.90E+07	8.07E+07	1.15E+08	1.06E+08	0.004	0.003	0.002	0.002	0.019	0.002	0.006	0.002	0.014	0.006	0.007	0.002	0.035	2	4	4	BTF3_MOUSE, SP:Q64152	SP|Q64152|BTF3_MOUSE
P61202	CSN2_MOUSE	COP9 signalosome complex subunit 2 OS=Mus musculus GN=Cops2 PE=1 SV=1	1	2	0.94	5.76E+07	6.65E+07	3.84E+07	6.96E+07	8.19E+07	5.06E+07	6.60E+07	6.46E+07	4.59E+07	5.09E+07	8.10E+07	3.92E+07	4.17E+07	0.017	0.036	0.011	0.020	0.058	0.007	0.018	0.035	0.051	0.003	0.069	0.021	0.125	2	2	2	CSN2_MOUSE, SP:P61202	SP|P61202|CSN2_MOUSE
Q9DCU6	RM04_MOUSE	39S ribosomal protein L4, mitochondrial OS=Mus musculus GN=Mrpl4 PE=2 SV=1	1	1	1.0	3.74E+07	7.27E+07	5.11E+07	3.60E+07	3.07E+07	5.60E+07	3.73E+07	5.86E+07	3.30E+07	4.68E+07	5.77E+07	2.95E+07	2.08E+07	0.044	0.021	0.050	0.106	0.052	0.006	0.060	0.066	0.061	0.012	0.100	0.028	0.209	2	1	1	RM04_MOUSE, SP:Q9DCU6	SP|Q9DCU6|RM04_MOUSE
Q91VM9	IPYR2_MOUSE	Inorganic pyrophosphatase 2, mitochondrial OS=Mus musculus GN=Ppa2 PE=2 SV=1	5	13	1.0	1.18E+09	1.28E+09	1.30E+09	1.32E+09	1.31E+09	1.19E+09	1.25E+09	1.27E+09	1.04E+09	1.08E+09	1.18E+09	1.01E+09	1.12E+09	1.08E-18	3.98E-22	3.68E-20	2.47E-13	3.44E-11	1.28E-23	2.07E-16	1.69E-14	6.10E-11	1.53E-21	7.53E-11	5.52E-16	1.63E-06	2, 3, 3, 4	11, 12, 13, 3, 3, 9	11, 12, 13, 3, 3, 9	IPYR2_MOUSE, SP:Q91VM9	SP|Q91VM9|IPYR2_MOUSE
Q60631	GRB2_MOUSE	Growth factor receptor-bound protein 2 OS=Mus musculus GN=Grb2 PE=1 SV=1	1	8	1.0	3.62E+07	6.10E+07	5.23E+07	3.93E+07	3.99E+07	7.05E+07	4.92E+07	5.80E+07	5.96E+07	6.49E+07	6.80E+07	4.39E+07	3.70E+07	0.045	0.018	0.041	0.075	0.032	0.009	0.035	0.020	0.033	0.006	0.058	0.027	0.139	3	8	8	GRB2_MOUSE, SP:Q60631	SP|Q60631|GRB2_MOUSE
Q04891	SOX13_MOUSE	Transcription factor SOX-13 OS=Mus musculus GN=Sox13 PE=2 SV=4	1	6	0.69	8.77E+08	1.51E+09	1.20E+09	9.07E+08	9.77E+08	1.21E+09	9.02E+08	1.14E+09	1.04E+09	1.16E+09	1.35E+09	1.09E+09	9.06E+08	2.38E-13	1.20E-19	2.16E-13	1.31E-09	2.73E-11	1.79E-26	1.80E-11	2.10E-14	1.24E-11	1.02E-23	8.85E-12	5.20E-14	5.34E-05	4	6	6	SOX13_MOUSE, SP:Q04891	SP|Q04891|SOX13_MOUSE
Q91YE8	SYNP2_MOUSE	Synaptopodin-2 OS=Mus musculus GN=Synpo2 PE=1 SV=2	3	12	1.0	1.50E+08	1.53E+08	1.51E+08	1.27E+08	1.59E+08	1.73E+08	1.44E+08	1.89E+08	1.70E+08	1.88E+08	1.74E+08	1.72E+08	1.63E+08	2.92E-01	1.72E-01	9.30E-01	0.002	0.002	9.35E-02	9.90E-01	2.54E-01	8.57E-01	3.43E-02	9.23E-01	2.64E-01	0.011	2, 3	12, 2, 4	12, 2, 4	SYNP2_MOUSE, SP:Q91YE8	SP|Q91YE8|SYNP2_MOUSE

P60840	ENSA_MOUSE	Alpha-endosulfine OS=Mus musculus GN=Ensa PE=1 SV=1	1	10	0.99	9.83E+07	9.82E+07	1.04E+08	1.17E+08	1.29E+08	7.66E+07	1.24E+08	7.07E+07	1.21E+08	1.02E+08	1.06E+08	1.10E+08	8.69E+07	0.003	0.002	0.002	0.005	0.027	0.002	0.003	0.002	0.011	0.001	0.007	0.018	0.049	3	10	10	ENSA_MOUSE, SP:P60840	SP|P60840|ENSA_MOUSE,SP|P56212|ARP19_MOUSE
Q9JK92	HSPB8_MOUSE	Heat shock protein beta-8 OS=Mus musculus GN=Hspb8 PE=1 SV=1	1	13	1.0	3.05E+08	2.95E+08	2.97E+08	3.30E+08	3.56E+08	3.60E+08	3.86E+08	4.39E+08	5.14E+08	3.57E+08	4.79E+08	4.31E+08	3.61E+08	5.31E-04	3.05E-04	2.95E-04	9.19E-03	1.53E-02	1.18E-04	2.40E-04	9.86E-07	8.13E-03	3.07E-08	4.77E-04	7.47E-05	3.57E-01	6	13	13	HSPB8_MOUSE, SP:Q9JK92	SP|Q9JK92|HSPB8_MOUSE
Q62448	IF4G2_MOUSE	Eukaryotic translation initiation factor 4 gamma 2 OS=Mus musculus GN=Eif4g2 PE=1 SV=2	1	2	0.9	1.21E+07	3.33E+07	2.90E+07	1.27E+07	3.34E+07	2.94E+07	2.82E+07	3.26E+07	2.44E+07	3.09E+07	2.05E+07	3.00E+07	1.86E+07	0.165	0.059	0.162	0.094	0.116	0.041	0.086	0.094	0.102	0.087	0.098	0.076	0.221	2	2	2	IF4G2_MOUSE, SP:Q62448	SP|Q62448|IF4G2_MOUSE
Q9CXT8	MPPB_MOUSE	Mitochondrial-processing peptidase subunit beta OS=Mus musculus GN=Pmpcb PE=2 SV=1	2	2	1.0	2.24E+08	2.03E+08	1.75E+08	2.48E+08	2.40E+08	1.65E+08	2.29E+08	1.96E+08	2.15E+08	1.92E+08	1.95E+08	2.10E+08	1.73E+08	1.62E-02	8.54E-02	8.92E-03	2.36E-01	0.003	1.09E-02	6.21E-02	5.61E-02	8.32E-01	1.58E-02	3.44E-01	2.46E-01	0.010	2, 5	1, 2	2	MPPB_MOUSE, SP:Q9CXT8	SP|Q9CXT8|MPPB_MOUSE
O35682	MYADM_MOUSE	Myeloid-associated differentiation marker OS=Mus musculus GN=Myadm PE=2 SV=2	2	13	1.0	3.06E+08	2.31E+08	2.32E+08	2.26E+08	2.45E+08	1.87E+08	1.62E+08	2.40E+08	2.16E+08	2.17E+08	2.37E+08	2.21E+08	2.03E+08	5.30E-04	5.10E-03	1.72E-02	1.85E-01	0.001	2.38E-03	5.41E-01	4.50E-02	3.82E-01	1.87E-03	2.18E-01	4.95E-02	0.006	3, 4	12, 13	12, 13	MYADM_MOUSE, SP:O35682	SP|O35682|MYADM_MOUSE
P35235	PTN11_MOUSE	Tyrosine-protein phosphatase non-receptor type 11 OS=Mus musculus GN=Ptpn11 PE=1 SV=2	1	12	0.99	3.64E+07	3.12E+07	3.39E+07	2.97E+07	2.99E+07	3.39E+07	2.89E+07	3.66E+07	2.66E+07	3.46E+07	2.57E+07	3.74E+07	3.29E+07	0.045	0.045	0.066	0.106	0.100	0.046	0.083	0.084	0.089	0.063	0.073	0.064	0.153	2	12	12	PTN11_MOUSE, SP:P35235	SP|P35235|PTN11_MOUSE
P63044	VAMP2_MOUSE	Vesicle-associated membrane protein 2 OS=Mus musculus GN=Vamp2 PE=1 SV=2	1	3	0.99	6.08E+07	7.97E+07	6.47E+07	6.30E+07	8.25E+07	5.31E+07	6.12E+07	7.50E+07	6.60E+07	6.37E+07	6.97E+07	6.69E+07	7.19E+07	0.014	0.010	0.014	0.019	0.054	0.004	0.022	0.015	0.034	0.006	0.026	0.015	0.065	2	3	3	VAMP2_MOUSE, SP:P63044	SP|P63044|VAMP2_MOUSE,SP|P63024|VAMP3_MOUSE
Q9EQI8	RM46_MOUSE	39S ribosomal protein L46, mitochondrial OS=Mus musculus GN=Mrpl46 PE=2 SV=1	1	3	0.99	9.10E+07	1.33E+08	1.16E+08	1.18E+08	1.33E+08	1.39E+08	1.19E+08	1.27E+08	1.52E+08	1.02E+08	9.63E+07	1.30E+08	1.07E+08	0.005	0.001	0.002	0.005	0.005	3.81E-01	0.003	7.01E-01	0.011	0.003	0.004	0.003	0.034	5	3	3	RM46_MOUSE, SP:Q9EQI8	SP|Q9EQI8|RM46_MOUSE
O35387	HAX1_MOUSE	HCLS1-associated protein X-1 OS=Mus musculus GN=Hax1 PE=1 SV=1	1	2	0.91	2.26E+07	1.72E+07	1.03E+07	1.52E+07	1.38E+07	1.88E+07	1.71E+07	2.08E+07	2.37E+07	1.94E+07	1.58E+07	2.15E+07	1.77E+07	0.091	0.175	0.140	0.199	0.169	0.104	0.143	0.097	0.157	0.115	0.136	0.125	0.226	3	2	2	HAX1_MOUSE, SP:O35387	SP|O35387|HAX1_MOUSE
P08730	K1C13_MOUSE	Keratin, type I cytoskeletal 13 OS=Mus musculus GN=Krt13 PE=1 SV=2	1	3	0.99	1.40E+08	7.90E+07	2.22E+08	8.43E+07	6.12E+07	1.81E+07	9.16E+07	4.39E+07	1.10E+08	8.06E+07	1.31E+08	1.07E+08	8.61E+07	4.89E-01	8.82E-03	0.006	0.040	0.184	0.004	0.007	0.003	0.021	3.25E-01	0.007	0.050	0.049	3	3	3	K1C13_MOUSE, SP:P08730	SP|P08730|K1C13_MOUSE
Q71LX4	TLN2_MOUSE	Talin-2 OS=Mus musculus GN=Tln2 PE=1 SV=2	5	10	1.0	5.19E+08	5.19E+08	4.80E+08	5.68E+08	5.64E+08	5.38E+08	4.87E+08	5.88E+08	5.22E+08	5.03E+08	5.24E+08	5.73E+08	5.58E+08	1.13E-07	1.29E-07	3.32E-08	3.80E-05	1.83E-04	4.40E-09	8.39E-06	7.43E-07	1.70E-04	4.15E-09	8.42E-06	6.35E-07	1.34E-02	2, 4, 5	1, 10, 2, 3	1, 10, 2, 3	TLN2_MOUSE, SP:Q71LX4	SP|Q71LX4|TLN2_MOUSE
Q61107	GBP4_MOUSE	Guanylate-binding protein 4 OS=Mus musculus GN=Gbp4 PE=1 SV=1	1	1	0.62	2.91E+07	2.45E+07	2.70E+07	2.37E+07	2.63E+07	1.63E+07	3.75E+07	2.52E+07	1.67E+07	2.58E+07	1.19E+07	3.72E+07	2.10E+07	0.065	0.065	0.089	0.121	0.186	0.067	0.057	0.138	0.122	0.148	0.074	0.102	0.207	2	1	1	GBP4_MOUSE, SP:Q61107	SP|Q61107|GBP4_MOUSE
P50543	S10AB_MOUSE	Protein S100-A11 OS=Mus musculus GN=S100a11 PE=2 SV=1	1	10	1.0	2.95E+08	3.61E+08	3.21E+08	2.57E+08	3.86E+08	3.52E+08	3.11E+08	4.01E+08	5.15E+08	4.06E+08	3.85E+08	3.51E+08	4.32E+08	1.36E-03	2.78E-04	8.76E-03	6.68E-03	2.91E-02	1.59E-05	6.09E-03	1.90E-06	3.00E-03	9.32E-06	7.61E-03	4.48E-04	1.19E-01	4	8, 10	10, 8	S10AB_MOUSE, SP:P50543	SP|P50543|S10AB_MOUSE
Q80W21	GSTM7_MOUSE	Glutathione S-transferase Mu 7 OS=Mus musculus GN=Gstm7 PE=1 SV=1	1	1	1.0	5.18E+07	7.60E+07	6.99E+07	6.43E+07	6.70E+07	8.77E+07	8.96E+07	6.74E+07	6.90E+07	5.55E+07	7.23E+07	5.32E+07	5.18E+07	0.022	0.008	0.013	0.031	0.020	0.004	0.007	0.013	0.044	0.005	0.042	0.019	0.100	4	1	1	GSTM7_MOUSE, SP:Q80W21	SP|Q80W21|GSTM7_MOUSE
Q9ES46	PARVB_MOUSE	Beta-parvin OS=Mus musculus GN=Parvb PE=1 SV=1	1	1	1.0	9.99E+07	1.37E+08	1.14E+08	1.15E+08	1.91E+08	1.50E+08	1.16E+08	1.43E+08	1.37E+08	1.30E+08	1.16E+08	1.44E+08	1.21E+08	0.003	9.79E-01	0.002	8.57E-01	0.004	2.19E-01	0.003	9.57E-01	0.005	6.36E-01	0.002	0.001	0.025	3, 4	1	1	PARVB_MOUSE, SP:Q9ES46	SP|Q9ES46|PARVB_MOUSE
Q00519	XDH_MOUSE	Xanthine dehydrogenase/oxidase OS=Mus musculus GN=Xdh PE=1 SV=4	2	4	1.0	7.87E+07	1.11E+08	1.03E+08	9.65E+07	1.47E+08	1.57E+08	1.29E+08	1.18E+08	1.19E+08	1.07E+08	1.14E+08	1.08E+08	1.03E+08	0.007	0.002	0.004	0.003	0.003	8.83E-01	0.002	0.002	0.010	7.99E-01	0.007	0.003	0.036	4, 5	1, 4	1, 4	XDH_MOUSE, SP:Q00519	SP|Q00519|XDH_MOUSE
P01635	KV5A3_MOUSE	Ig kappa chain V-V region K2 (Fragment) OS=Mus musculus PE=1 SV=1	1	5	0.99	1.37E+07	1.15E+07	6.35E+06	4.39E+07	1.82E+07	2.32E+07	4.56E+07	9.70E+06	1.27E+07	1.16E+08	4.97E+07	1.48E+07	7.70E+07	0.152	0.233	0.034	0.166	0.144	0.153	0.041	0.171	0.007	0.017	0.182	0.219	0.059	3	5	5	KV5A3_MOUSE, SP:P01635	SP|P01635|KV5A3_MOUSE
Q8CBY8	DCTN4_MOUSE	Dynactin subunit 4 OS=Mus musculus GN=Dctn4 PE=2 SV=1	1	5	0.99	4.99E+07	3.42E+07	2.69E+07	3.28E+07	2.65E+07	3.04E+07	2.79E+07	3.96E+07	4.35E+07	4.61E+07	4.13E+07	3.39E+07	3.70E+07	0.024	0.070	0.058	0.122	0.113	0.039	0.088	0.040	0.061	0.027	0.085	0.060	0.139	3	5	5	DCTN4_MOUSE, SP:Q8CBY8	SP|Q8CBY8|DCTN4_MOUSE
Q9CQ19	MYL9_MOUSE	Myosin regulatory light polypeptide 9 OS=Mus musculus GN=Myl9 PE=1 SV=3	1	12	1.0	3.82E+08	5.17E+08	5.05E+08	3.65E+08	7.70E+08	5.31E+08	4.39E+08	6.10E+08	6.09E+08	6.74E+08	6.69E+08	3.99E+08	3.80E+08	2.46E-05	4.44E-08	7.43E-05	1.69E-07	2.17E-04	5.50E-09	4.23E-05	3.23E-08	1.80E-06	5.95E-12	1.21E-03	3.30E-07	2.58E-01	5, 6	11, 12	11, 12	MYL9_MOUSE, SP:Q9CQ19	SP|Q9CQ19|MYL9_MOUSE,SP|Q3THE2|ML12B_MOUSE
P70428	EXT2_MOUSE	Exostosin-2 OS=Mus musculus GN=Ext2 PE=1 SV=1	1	9	0.78	2.11E+08	1.25E+08	7.24E+07	2.02E+08	1.34E+08	9.88E+07	1.99E+08	1.01E+08	1.08E+08	1.73E+08	1.18E+08	1.90E+08	1.17E+08	2.66E-02	0.007	4.84E-02	0.004	0.014	3.93E-01	1.53E-01	0.003	0.001	5.52E-01	5.80E-01	0.006	0.027	3	9	9	EXT2_MOUSE, SP:P70428	SP|P70428|EXT2_MOUSE
P35441	TSP1_MOUSE	Thrombospondin-1 OS=Mus musculus GN=Thbs1 PE=1 SV=1	2	3	1.0	9.10E+07	5.88E+07	6.72E+07	7.68E+07	2.39E+08	6.00E+07	5.43E+07	8.28E+07	5.73E+07	5.91E+07	4.11E+07	4.41E+07	4.86E+07	0.004	0.009	0.008	2.19E-01	0.044	0.010	0.029	0.022	0.039	0.026	0.058	0.011	0.107	2, 3	1, 3	1, 3	TSP1_MOUSE, SP:P35441	SP|P35441|TSP1_MOUSE
Q64487	PTPRD_MOUSE	Receptor-type tyrosine-protein phosphatase delta OS=Mus musculus GN=Ptprd PE=2 SV=1	1	1	0.94	3.40E+08	4.19E+08	3.60E+08	2.86E+08	2.54E+08	4.33E+08	2.66E+08	4.16E+08	4.17E+08	4.45E+08	4.56E+08	3.02E+08	2.83E+08	2.13E-04	4.07E-05	2.29E-03	1.80E-01	3.20E-03	1.21E-06	1.92E-02	4.48E-05	1.03E-03	1.88E-07	2.52E-02	2.56E-04	0.001	4	1	1	PTPRD_MOUSE, SP:Q64487	SP|Q64487|PTPRD_MOUSE
P26450	P85A_MOUSE	Phosphatidylinositol 3-kinase regulatory subunit alpha OS=Mus musculus GN=Pik3r1 PE=1 SV=1	1	5	0.85	1.83E+08	1.39E+08	1.07E+08	1.15E+08	1.58E+08	1.89E+08	1.27E+08	2.16E+08	1.46E+08	1.93E+08	2.34E+08	1.22E+08	1.28E+08	7.91E-02	0.001	0.002	0.002	0.001	1.97E-01	0.002	6.59E-01	7.67E-01	2.39E-03	0.004	1.14E-01	0.022	4	5	5	P85A_MOUSE, SP:P26450	SP|P26450|P85A_MOUSE
P11352	GPX1_MOUSE	Glutathione peroxidase 1 OS=Mus musculus GN=Gpx1 PE=1 SV=2	1	6	1.0	4.65E+07	5.41E+07	4.30E+07	4.25E+07	5.03E+07	4.43E+07	4.30E+07	5.83E+07	6.36E+07	4.51E+07	4.71E+07	4.78E+07	4.49E+07	0.028	0.028	0.036	0.053	0.071	0.013	0.046	0.017	0.062	0.019	0.050	0.027	0.116	3	6	6	GPX1_MOUSE, SP:P11352	SP|P11352|GPX1_MOUSE
Q6IFZ6	K2C1B_MOUSE	Keratin, type II cytoskeletal 1b OS=Mus musculus GN=Krt77 PE=1 SV=1	1	1	0.97	1.15E+07	1.34E+07	1.61E+07	9.59E+06	1.38E+07	1.52E+07	1.17E+07	1.98E+08	1.78E+07	1.28E+07	2.31E+07	1.02E+07	1.27E+07	0.187	0.148	0.214	0.210	0.204	0.165	0.214	0.147	0.224	0.086	0.240	2.17E-01	0.267	3	1	1	K2C1B_MOUSE, SP:Q6IFZ6	SP|Q6IFZ6|K2C1B_MOUSE
Q8CGB6	TENC1_MOUSE	Tensin-like C1 domain-containing phosphatase OS=Mus musculus GN=Tenc1 PE=1 SV=1	1	8	0.81	1.36E+09	1.33E+09	1.25E+09	1.15E+09	1.11E+09	1.20E+09	8.47E+08	1.14E+09	9.37E+08	1.02E+09	9.74E+08	9.44E+08	9.00E+08	3.57E-20	4.68E-20	1.38E-16	7.18E-11	4.88E-11	9.07E-23	1.75E-10	1.16E-12	4.44E-10	8.55E-17	6.95E-10	8.67E-14	6.75E-05	5	8	8	TENC1_MOUSE, SP:Q8CGB6	SP|Q8CGB6|TENC1_MOUSE
Q9D0F3	LMAN1_MOUSE	Protein ERGIC-53 OS=Mus musculus GN=Lman1 PE=2 SV=1	1	5	0.86	3.32E+07	4.27E+07	1.69E+07	4.50E+07	5.16E+07	4.56E+07	3.11E+07	4.60E+07	2.11E+07	5.34E+07	3.51E+07	2.79E+07	5.13E+07	0.056	0.147	0.036	0.053	0.070	0.026	0.080	0.117	0.048	0.040	0.117	0.045	0.104	2	5	5	LMAN1_MOUSE, SP:Q9D0F3	SP|Q9D0F3|LMAN1_MOUSE
Q9Z2U0	PSA7L_MOUSE	Proteasome subunit alpha type-7 OS=Mus musculus GN=Psma7 PE=1 SV=1	2	12	1.0	2.93E+08	3.81E+08	3.16E+08	2.69E+08	2.27E+08	3.58E+08	3.20E+08	3.46E+08	3.26E+08	3.32E+08	3.65E+08	2.50E+08	2.82E+08	9.31E-04	1.52E-04	3.31E-03	3.22E-01	1.69E-02	3.11E-06	2.41E-03	8.69E-04	1.68E-02	5.90E-06	9.50E-02	1.62E-03	0.001	2, 4	12, 3	12, 3	PSA7L_MOUSE, SP:Q9CWH6	SP|Q9CWH6|PSA7L_MOUSE,SP|Q9Z2U0|PSA7_MOUSE
P26645	MARCS_MOUSE	Myristoylated alanine-rich C-kinase substrate OS=Mus musculus GN=Marcks PE=1 SV=2	1	12	0.99	2.67E+07	2.10E+07	2.11E+07	1.78E+07	3.47E+07	1.94E+07	2.58E+07	2.67E+07	3.15E+07	3.24E+07	1.75E+07	3.66E+07	3.12E+07	0.073	0.090	0.121	0.089	0.165	0.082	0.095	0.067	0.096	0.103	0.076	0.096	0.159	3	12	12	MARCS_MOUSE, SP:P26645	SP|P26645|MARCS_MOUSE
Q3UH68	LIMC1_MOUSE	LIM and calponin homology domains-containing protein 1 OS=Mus musculus GN=Limch1 PE=1 SV=2	1	1	0.99	4.22E+07	4.12E+07	2.97E+07	3.95E+07	4.57E+07	3.18E+07	3.39E+07	3.66E+07	3.13E+07	3.39E+07	3.32E+07	3.39E+07	3.72E+07	0.035	0.057	0.041	0.062	0.107	0.026	0.067	0.068	0.092	0.042	0.084	0.064	0.139	3	1	1	LIMC1_MOUSE, SP:Q3UH68	SP|Q3UH68|LIMC1_MOUSE
Q6R0H7	GNAS1_MOUSE	Guanine nucleotide-binding protein G(s) subunit alpha isoforms XLas OS=Mus musculus GN=Gnas PE=2 SV=1	2	6	1.0	1.31E+08	1.84E+08	1.74E+08	1.38E+08	1.65E+08	1.70E+08	1.32E+08	1.71E+08	1.49E+08	1.57E+08	2.40E+08	1.38E+08	1.66E+08	6.81E-01	6.82E-02	6.12E-01	0.002	0.002	2.26E-02	0.002	5.94E-01	0.002	1.68E-03	0.003	5.16E-01	0.011	2, 5	1, 6	1, 6	GNAS1_MOUSE, SP:Q6R0H7	SP|Q6R0H7|GNAS1_MOUSE,SP|P63094|GNAS2_MOUSE
Q2VLH6	C163A_MOUSE	Scavenger receptor cysteine-rich type 1 protein M130 OS=Mus musculus GN=Cd163 PE=1 SV=1	1	6	0.69	1.25E+08	8.60E+07	1.02E+08	9.64E+07	6.93E+07	9.71E+07	8.43E+07	7.09E+07	9.48E+07	9.12E+07	6.48E+07	9.41E+07	9.84E+07	8.95E-01	0.002	0.003	0.029	0.015	0.003	0.009	0.005	0.014	0.008	0.011	0.017	0.039	3	6	6	C163A_MOUSE, SP:Q2VLH6	SP|Q2VLH6|C163A_MOUSE
Q4VAC9	PKHG3_MOUSE	Pleckstrin homology domain-containing family G member 3 OS=Mus musculus GN=Plekhg3 PE=1 SV=1	1	1	0.63	2.86E+07	3.56E+07	1.31E+08	3.53E+07	2.02E+07	1.54E+07	2.98E+07	1.87E+07	2.97E+07	2.01E+07	2.83E+07	9.34E+07	6.60E+07	0.068	5.15E-01	0.052	0.154	0.193	0.036	0.081	0.074	0.153	0.055	0.011	0.139	0.074	4	1	1	PKHG3_MOUSE, SP:Q4VAC9	SP|Q4VAC9|PKHG3_MOUSE
Q9Z1J3	NFS1_MOUSE	Cysteine desulfurase, mitochondrial OS=Mus musculus GN=Nfs1 PE=2 SV=2	1	8	1.0	6.12E+07	6.41E+07	8.13E+07	8.41E+07	8.28E+07	6.34E+07	8.26E+07	3.77E+07	6.72E+07	7.06E+07	8.15E+07	6.65E+07	7.36E+07	0.014	0.004	0.006	0.019	0.040	0.008	0.010	0.014	0.027	0.003	0.027	0.061	0.063	3	8	8	NFS1_MOUSE, SP:Q9Z1J3	SP|Q9Z1J3|NFS1_MOUSE
Q91YP0	L2HDH_MOUSE	L-2-hydroxyglutarate dehydrogenase, mitochondrial OS=Mus musculus GN=L2hgdh PE=2 SV=1	1	6	0.99	2.31E+07	3.45E+07	2.28E+07	2.68E+07	1.06E+07	4.88E+07	3.30E+07	3.49E+07	2.63E+07	1.97E+07	1.78E+07	2.40E+07	1.29E+07	0.088	0.083	0.076	0.228	0.062	0.038	0.070	0.086	0.155	0.102	0.123	0.068	0.261	2	6	6	L2HDH_MOUSE, SP:Q91YP0	SP|Q91YP0|L2HDH_MOUSE
P35505	FAAA_MOUSE	Fumarylacetoacetase OS=Mus musculus GN=Fah PE=1 SV=1	1	3	0.99	7.93E+07	9.02E+07	9.02E+07	9.14E+07	1.17E+08	1.11E+08	1.29E+08	8.69E+07	9.16E+07	8.36E+07	7.78E+07	9.09E+07	6.62E+07	0.006	0.003	0.004	0.007	0.010	0.002	0.002	0.005	0.018	0.004	0.012	0.010	0.074	5	3	3	FAAA_MOUSE, SP:P35505	SP|P35505|FAAA_MOUSE
Q924M7	MPI_MOUSE	Mannose-6-phosphate isomerase OS=Mus musculus GN=Mpi PE=2 SV=1	5	13	1.0	9.79E+08	1.12E+09	1.10E+09	9.02E+08	1.10E+09	1.07E+09	9.60E+08	1.06E+09	1.04E+09	1.04E+09	9.86E+08	8.46E+08	8.10E+08	1.85E-15	6.39E-19	9.65E-14	3.46E-11	4.03E-10	1.06E-20	1.27E-12	7.33E-15	1.08E-10	3.54E-18	3.53E-09	1.87E-13	2.09E-04	2, 3, 7	10, 13, 2, 9	10, 13, 2, 9	MPI_MOUSE, SP:Q924M7	SP|Q924M7|MPI_MOUSE
Q60759	GCDH_MOUSE	Glutaryl-CoA dehydrogenase, mitochondrial OS=Mus musculus GN=Gcdh PE=2 SV=1	4	11	1.0	5.23E+08	6.58E+08	8.10E+08	5.88E+08	4.67E+08	7.04E+08	5.63E+08	5.95E+08	5.31E+08	5.94E+08	6.31E+08	6.25E+08	5.38E+08	1.07E-07	1.40E-13	1.68E-08	5.40E-04	3.27E-06	9.15E-12	7.44E-07	5.93E-07	1.60E-05	3.59E-11	2.03E-06	5.42E-07	1.93E-02	2, 3, 3, 4, 4	1, 11, 6, 7	1, 11, 6, 7	GCDH_MOUSE, SP:Q60759	SP|Q60759|GCDH_MOUSE
Q71RI9	KAT3_MOUSE	Kynurenine--oxoglutarate transaminase 3 OS=Mus musculus GN=Ccbl2 PE=2 SV=1	4	10	1.0	5.93E+08	6.45E+08	6.77E+08	7.22E+08	7.68E+08	8.47E+08	7.62E+08	5.04E+08	5.22E+08	5.77E+08	6.56E+08	5.23E+08	4.81E+08	7.39E-09	4.45E-11	1.09E-10	2.32E-07	1.18E-07	2.18E-11	1.09E-09	8.72E-07	2.89E-05	1.36E-11	4.32E-05	1.20E-05	5.99E-02	2, 3, 4	1, 10, 2	1, 10, 2	KAT3_MOUSE, SP:Q71RI9	SP|Q71RI9|KAT3_MOUSE
Q9JMC3	DNJA4_MOUSE	DnaJ homolog subfamily A member 4 OS=Mus musculus GN=Dnaja4 PE=2 SV=1	1	3	1.0	7.63E+07	6.02E+07	5.82E+07	5.39E+07	5.10E+07	7.65E+07	6.07E+07	5.16E+07	8.52E+07	6.91E+07	5.86E+07	7.44E+07	7.63E+07	0.007	0.013	0.021	0.052	0.027	0.010	0.023	0.007	0.029	0.011	0.020	0.035	0.060	5	3	3	DNJA4_MOUSE, SP:Q9JMC3	SP|Q9JMC3|DNJA4_MOUSE
O70571	PDK4_MOUSE	[Pyruvate dehydrogenase [lipoamide]] kinase isozyme 4, mitochondrial OS=Mus musculus GN=Pdk4 PE=2 SV=1	3	12	1.0	2.77E+08	2.11E+08	3.40E+08	2.55E+08	4.01E+08	3.05E+08	2.58E+08	2.80E+08	4.29E+08	3.76E+08	3.63E+08	2.60E+08	2.36E+08	2.81E-03	1.11E-04	1.03E-02	4.36E-03	8.45E-02	2.35E-02	3.30E-02	2.71E-05	7.66E-03	9.46E-06	1.07E-01	1.98E-02	0.004	2, 4	1, 12, 9	1, 12, 9	PDK4_MOUSE, SP:O70571	SP|O70571|PDK4_MOUSE
Q9DBG5	PLIN3_MOUSE	Perilipin-3 OS=Mus musculus GN=Plin3 PE=1 SV=1	8	13	1.0	1.59E+09	2.07E+09	1.69E+09	1.37E+09	1.90E+09	1.95E+09	1.72E+09	1.79E+09	1.50E+09	1.53E+09	1.55E+09	1.45E+09	1.48E+09	2.87E-24	1.96E-27	1.54E-19	4.17E-19	7.93E-19	2.56E-37	1.76E-22	8.51E-21	1.70E-15	7.17E-27	4.66E-16	1.66E-22	6.98E-09	2, 3, 6, 7	1, 1, 5, 13, 2, 4	1, 1, 5, 13, 2, 4	PLIN3_MOUSE, SP:Q9DBG5	SP|Q9DBG5|PLIN3_MOUSE
A2ARV4	LRP2_MOUSE	Low-density lipoprotein receptor-related protein 2 OS=Mus musculus GN=Lrp2 PE=1 SV=1	1	4	1.0	4.52E+08	3.32E+08	4.07E+08	2.75E+08	9.64E+07	5.05E+08	2.89E+08	2.51E+08	4.15E+08	3.26E+08	4.08E+08	4.60E+08	2.09E+08	1.85E-06	3.30E-06	2.64E-03	0.013	4.41E-04	2.38E-05	6.88E-03	3.61E-05	1.97E-02	8.86E-07	2.35E-04	3.52E-02	0.005	5	3	4	LRP2_MOUSE, SP:A2ARV4	SP|A2ARV4|LRP2_MOUSE
Q61646	HPT_MOUSE	Haptoglobin OS=Mus musculus GN=Hp PE=1 SV=1	3	7	1.0	1.30E+08	9.33E+08	1.09E+08	4.34E+08	5.02E+07	2.32E+08	2.57E+08	4.45E+08	3.41E+07	5.60E+08	1.56E+08	5.16E+07	3.69E+08	0.001	0.002	8.72E-06	0.068	4.37E-01	8.03E-17	2.76E-02	0.078	4.29E-05	1.24E-01	0.055	7.43E-05	3.41E-01	2, 4	1, 2, 7	1, 2, 7	HPT_MOUSE, SP:Q61646	SP|Q61646|HPT_MOUSE
P48193	41_MOUSE	Protein 4.1 OS=Mus musculus GN=Epb41 PE=1 SV=2	1	4	0.91	4.58E+07	2.96E+07	2.01E+07	2.56E+07	4.33E+07	3.52E+07	2.27E+07	2.55E+07	4.11E+07	2.80E+07	3.42E+07	4.06E+07	3.90E+07	0.029	0.096	0.081	0.067	0.096	0.050	0.110	0.044	0.113	0.039	0.065	0.102	0.133	3	4	4	41_MOUSE, SP:P48193	SP|P48193|41_MOUSE
Q9ESW4	AGK_MOUSE	Acylglycerol kinase, mitochondrial OS=Mus musculus GN=Agk PE=1 SV=1	2	1	1.0	1.64E+08	2.07E+08	1.47E+08	1.77E+08	1.64E+08	1.65E+08	1.83E+08	2.07E+08	1.20E+08	1.29E+08	1.70E+08	1.32E+08	1.43E+08	2.37E-01	4.11E-01	1.84E-01	0.002	0.003	1.90E-02	3.77E-01	0.002	0.006	8.73E-02	0.004	2.20E-01	0.018	3, 4	1	1	AGK_MOUSE, SP:Q9ESW4	SP|Q9ESW4|AGK_MOUSE
Q921T2	TOIP1_MOUSE	Torsin-1A-interacting protein 1 OS=Mus musculus GN=Tor1aip1 PE=1 SV=1	1	2	0.99	2.88E+07	3.20E+07	3.05E+07	2.84E+07	4.12E+07	4.52E+07	3.47E+07	3.45E+07	2.91E+07	3.70E+07	2.27E+07	3.41E+07	3.95E+07	0.065	0.054	0.070	0.072	0.069	0.043	0.064	0.075	0.082	0.075	0.083	0.069	0.131	3	2	2	TOIP1_MOUSE, SP:Q921T2	SP|Q921T2|TOIP1_MOUSE
Q91WK0	LRRF1_MOUSE	Leucine-rich repeat flightless-interacting protein 2 OS=Mus musculus GN=Lrrfip2 PE=1 SV=1	1	4	0.99	3.00E+07	1.84E+07	1.12E+08	2.64E+07	3.26E+07	2.00E+07	3.12E+07	1.26E+08	2.03E+07	2.60E+07	2.05E+07	2.72E+07	3.60E+07	0.063	0.001	0.078	0.097	0.163	0.098	0.075	0.115	0.122	0.086	0.109	0.002	0.142	4	4	4	LRRF1_MOUSE, SP:Q3UZ39	SP|Q3UZ39|LRRF1_MOUSE,SP|Q91WK0|LRRF2_MOUSE
Q9D1B9	RM28_MOUSE	39S ribosomal protein L28, mitochondrial OS=Mus musculus GN=Mrpl28 PE=2 SV=3	1	6	0.99	1.27E+08	1.37E+08	1.06E+08	1.22E+08	8.84E+07	1.17E+08	9.59E+07	1.15E+08	1.11E+08	1.19E+08	1.49E+08	1.17E+08	9.10E+07	8.55E-01	0.001	0.001	0.016	0.009	2.27E-01	0.006	0.003	0.006	1.54E-01	0.005	0.004	0.045	4	6	6	RM28_MOUSE, SP:Q9D1B9	SP|Q9D1B9|RM28_MOUSE
P00330	ADH1_YEAST	Alcohol dehydrogenase I (EC 1.1.1.1) OS=Saccharomyces cerevisiae GN= ADH1 PE=1 SV=5	12	13	1.0	7.06E+10	6.84E+10	6.91E+10	8.69E+10	1.12E+11	1.39E+11	8.93E+10	9.38E+10	8.09E+10	1.22E+11	6.86E+10	9.83E+10	1.86E+11	0	0	0	0	0	0	0	0	0	0	0	0	0	2, 3, 5, 2, 3, 7, 3, 4, 4, 5, 5, 6	1, 11, 1, 12, 11, 12, 13, 4, 4, 13, 9, 13	11, 13, 15, 19, 23, 34, 4	ADH1_YEAST, SP:P00330	SP|P00330|ADH1_YEAST
Q5SQM0	EMAL6_MOUSE	Echinoderm microtubule-associated protein-like 6 OS=Mus musculus GN=Eml6 PE=2 SV=1	1	2	0.99	7.50E+07	1.02E+08	5.73E+07	7.39E+07	1.03E+08	5.25E+07	6.40E+07	7.52E+07	1.12E+08	1.39E+08	6.05E+07	5.79E+07	5.11E+07	0.008	0.016	0.009	0.011	0.057	0.001	0.021	0.003	0.004	0.011	0.037	0.015	0.102	4	2	2	EMAL6_MOUSE, SP:Q5SQM0	SP|Q5SQM0|EMAL6_MOUSE
P54923	ADPRH_MOUSE	[Protein ADP-ribosylarginine] hydrolase OS=Mus musculus GN=Adprh PE=2 SV=1	1	1	0.85	5.25E+07	1.04E+08	6.60E+07	3.20E+07	4.96E+07	1.01E+08	7.06E+07	6.72E+07	7.30E+07	6.19E+07	7.63E+07	4.72E+07	7.34E+07	0.022	0.010	0.061	0.055	0.014	0.001	0.016	0.012	0.037	0.005	0.052	0.021	0.064	4	1	1	ADPRH_MOUSE, SP:P54923	SP|P54923|ADPRH_MOUSE
O88696	CLPP_MOUSE	Putative ATP-dependent Clp protease proteolytic subunit, mitochondrial OS=Mus musculus GN=Clpp PE=2 SV=1	2	13	1.0	3.60E+08	3.67E+08	3.35E+08	4.32E+08	3.99E+08	3.27E+08	4.17E+08	3.37E+08	3.72E+08	4.00E+08	3.52E+08	3.70E+08	3.24E+08	2.16E-04	3.99E-04	4.23E-05	6.80E-03	5.56E-02	5.21E-05	2.12E-04	3.82E-04	5.80E-03	1.20E-04	6.26E-03	8.14E-03	9.07E-01	4, 5	13, 5	13, 5	CLPP_MOUSE, SP:O88696	SP|O88696|CLPP_MOUSE
O35972	RM23_MOUSE	39S ribosomal protein L23, mitochondrial OS=Mus musculus GN=Mrpl23 PE=2 SV=1	1	4	1.0	5.10E+07	5.98E+07	5.91E+07	5.33E+07	6.70E+07	5.31E+07	5.90E+07	6.59E+07	5.40E+07	5.67E+07	6.61E+07	3.74E+07	3.78E+07	0.023	0.013	0.022	0.031	0.055	0.010	0.024	0.026	0.043	0.007	0.074	0.021	0.137	3	4	4	RM23_MOUSE, SP:O35972	SP|O35972|RM23_MOUSE
Q9DBG3	AP2B1_MOUSE	AP-2 complex subunit beta OS=Mus musculus GN=Ap2b1 PE=1 SV=1	1	1	1.0	5.68E+07	1.36E+08	1.16E+08	6.90E+07	1.06E+08	1.40E+08	9.31E+07	1.15E+08	9.61E+07	1.22E+08	1.14E+08	8.11E+07	9.89E+07	0.017	8.68E-01	0.011	0.010	0.005	2.17E-01	0.007	0.005	0.006	6.66E-01	0.016	0.003	0.038	2, 3	1	1	AP2B1_MOUSE, SP:Q9DBG3	SP|Q9DBG3|AP2B1_MOUSE,SP|O35643|AP1B1_MOUSE
Q9WV60	GSK3B_MOUSE	Glycogen synthase kinase-3 beta OS=Mus musculus GN=Gsk3b PE=1 SV=2	1	1	0.99	1.24E+08	6.49E+07	7.32E+07	1.10E+08	7.52E+07	6.71E+07	7.65E+07	7.16E+07	1.11E+08	8.60E+07	8.25E+07	9.31E+07	1.20E+08	8.91E-01	0.006	0.002	0.024	0.035	0.007	0.012	0.003	0.017	0.003	0.011	0.016	0.025	3	1	1	GSK3B_MOUSE, SP:Q9WV60	SP|Q9WV60|GSK3B_MOUSE
Q8C156	CND2_MOUSE	Condensin complex subunit 2 OS=Mus musculus GN=Ncaph PE=2 SV=1	1	9	1.0	8.91E+08	8.06E+08	7.35E+08	5.71E+08	1.93E+08	7.72E+08	8.17E+08	6.71E+08	7.00E+08	8.27E+08	5.78E+08	8.88E+08	6.91E+08	3.03E-13	9.25E-12	4.93E-08	7.92E-01	9.05E-07	5.93E-14	4.03E-10	2.63E-09	5.82E-08	7.21E-10	2.51E-09	8.32E-08	1.75E-03	4	9	9	CND2_MOUSE, SP:Q8C156	SP|Q8C156|CND2_MOUSE
P31750	AKT1_MOUSE	RAC-alpha serine/threonine-protein kinase OS=Mus musculus GN=Akt1 PE=1 SV=1	1	2	0.99	2.14E+07	3.15E+07	2.57E+07	2.88E+07	2.92E+07	2.83E+07	2.77E+07	4.47E+07	3.28E+07	4.58E+07	4.68E+07	2.05E+07	2.82E+07	0.098	0.072	0.069	0.109	0.121	0.046	0.088	0.064	0.061	0.020	0.142	0.047	0.172	3	2	2	AKT1_MOUSE, SP:P31750	SP|P31750|AKT1_MOUSE
Q8BGC4	ZADH2_MOUSE	Zinc-binding alcohol dehydrogenase domain-containing protein 2 OS=Mus musculus GN=Zadh2 PE=2 SV=1	4	13	1.0	7.41E+08	7.05E+08	7.44E+08	6.45E+08	8.47E+08	9.60E+08	8.40E+08	7.45E+08	7.57E+08	6.69E+08	8.73E+08	7.69E+08	7.56E+08	1.55E-11	1.66E-12	1.48E-09	2.01E-08	4.94E-09	7.86E-13	5.89E-11	1.42E-10	1.87E-06	4.21E-16	2.89E-08	3.85E-09	4.99E-04	2, 5, 3, 4, 5	13, 2, 4, 8, 6, 6, 12	12, 2, 12, 6, 13, 6	ZADH2_MOUSE, SP:Q8BGC4	SP|Q8BGC4|ZADH2_MOUSE
Q3UGR5	HDHD2_MOUSE	Haloacid dehalogenase-like hydrolase domain-containing protein 2 OS=Mus musculus GN=Hdhd2 PE=1 SV=1	1	1	1.0	1.40E+07	1.94E+07	2.49E+07	1.87E+07	1.73E+07	1.95E+07	1.99E+07	2.28E+07	2.54E+07	2.66E+07	1.58E+07	1.59E+07	1.81E+07	0.148	0.075	0.116	0.172	0.165	0.092	0.126	0.090	0.119	0.116	0.172	0.115	0.224	3	1	1	HDHD2_MOUSE, SP:Q3UGR5	SP|Q3UGR5|HDHD2_MOUSE
O35127	C10_MOUSE	Protein C10 OS=Mus musculus GN=Grcc10 PE=2 SV=1	1	2	0.99	2.96E+07	3.52E+07	4.25E+07	2.85E+07	7.91E+07	4.41E+07	4.49E+07	5.73E+07	4.85E+07	5.72E+07	3.94E+07	4.28E+07	4.55E+07	0.069	0.033	0.078	0.023	0.077	0.043	0.046	0.036	0.044	0.036	0.066	0.030	0.118	4	2	2	C10_MOUSE, SP:O35127	SP|O35127|C10_MOUSE
O35943	FRDA_MOUSE	Frataxin, mitochondrial OS=Mus musculus GN=Fxn PE=2 SV=1	2	11	1.0	3.55E+08	2.36E+08	3.44E+08	3.70E+08	4.24E+08	4.08E+08	3.51E+08	3.02E+08	3.80E+08	3.22E+08	3.73E+08	2.72E+08	3.96E+08	8.57E-05	5.65E-05	8.05E-05	1.76E-03	5.55E-03	2.36E-03	9.73E-04	1.41E-04	2.51E-02	4.58E-06	5.72E-02	7.45E-03	2.33E-01	2, 4	10, 11	10, 11	FRDA_MOUSE, SP:O35943	SP|O35943|FRDA_MOUSE
O70548	TELT_MOUSE	Telethonin OS=Mus musculus GN=Tcap PE=2 SV=1	2	12	1.0	3.85E+08	4.05E+08	4.71E+08	3.64E+08	5.59E+08	4.41E+08	4.34E+08	4.64E+08	5.77E+08	5.37E+08	5.79E+08	4.55E+08	5.43E+08	2.42E-05	2.41E-07	9.40E-05	4.77E-05	2.15E-03	1.05E-06	5.56E-05	1.19E-07	7.26E-05	4.50E-10	2.61E-04	3.93E-05	1.76E-02	3, 4	12, 4	12, 4	TELT_MOUSE, SP:O70548	SP|O70548|TELT_MOUSE
Q91WK1	SPRY4_MOUSE	SPRY domain-containing protein 4 OS=Mus musculus GN=Spryd4 PE=2 SV=1	1	7	1.0	1.05E+08	1.60E+08	1.41E+08	1.23E+08	1.54E+08	1.45E+08	1.43E+08	1.49E+08	1.85E+08	1.26E+08	1.42E+08	1.39E+08	1.00E+08	0.003	4.11E-01	0.001	0.003	0.004	1.03E-01	0.001	1.91E-01	0.006	2.48E-01	0.003	0.001	0.038	5	7	7	SPRY4_MOUSE, SP:Q91WK1	SP|Q91WK1|SPRY4_MOUSE
Q9CXJ1	SYEM_MOUSE	Probable glutamyl-tRNA synthetase, mitochondrial OS=Mus musculus GN=Ears2 PE=2 SV=1	1	12	0.73	1.84E+09	1.01E+09	1.55E+09	2.49E+09	2.22E+09	1.45E+09	1.70E+09	1.05E+09	2.16E+09	1.49E+09	2.09E+09	1.86E+09	2.04E+09	1.53E-26	4.45E-24	1.91E-33	2.12E-21	3.24E-13	5.48E-17	5.35E-21	8.69E-28	1.26E-14	1.29E-33	1.86E-19	1.70E-12	3.51E-12	4	12	12	SYEM_MOUSE, SP:Q9CXJ1	SP|Q9CXJ1|SYEM_MOUSE
P82348	SGCG_MOUSE	Gamma-sarcoglycan OS=Mus musculus GN=Sgcg PE=1 SV=2	1	6	0.99	2.41E+08	2.87E+08	2.26E+08	2.41E+08	2.33E+08	2.60E+08	1.98E+08	2.67E+08	2.23E+08	2.49E+08	2.16E+08	2.30E+08	1.95E+08	8.97E-03	8.65E-03	1.18E-02	2.88E-01	2.11E-01	2.34E-04	1.69E-01	3.92E-02	1.72E-01	6.36E-03	1.81E-01	2.32E-02	0.007	4	6	6	SGCG_MOUSE, SP:P82348	SP|P82348|SGCG_MOUSE
P24472	GSTA4_MOUSE	Glutathione S-transferase A4 OS=Mus musculus GN=Gsta4 PE=1 SV=3	2	8	1.0	7.12E+08	9.26E+08	8.86E+08	7.22E+08	7.83E+08	7.99E+08	8.18E+08	8.80E+08	6.64E+08	6.70E+08	6.08E+08	7.72E+08	6.20E+08	1.57E-07	4.51E-12	1.87E-06	2.71E-04	1.87E-06	2.60E-12	1.48E-08	3.48E-05	5.82E-04	5.43E-09	4.75E-06	1.19E-09	3.54E-02	3, 6	6, 8	6, 8	GSTA4_MOUSE, SP:P24472	SP|P24472|GSTA4_MOUSE
Q9QZZ6	DERM_MOUSE	Dermatopontin OS=Mus musculus GN=Dpt PE=2 SV=1	1	8	0.99	1.85E+08	1.12E+08	9.91E+07	1.58E+08	2.06E+08	1.09E+08	1.37E+08	1.02E+08	1.43E+08	1.61E+08	9.20E+07	1.52E+08	1.51E+08	8.85E-02	0.002	3.01E-01	5.93E-01	0.011	8.02E-01	0.001	7.93E-01	0.002	0.002	0.002	0.006	0.015	3	8	8	DERM_MOUSE, SP:Q9QZZ6	SP|Q9QZZ6|DERM_MOUSE
Q8K4S1	PLCE1_MOUSE	1-phosphatidylinositol-4,5-bisphosphate phosphodiesterase epsilon-1 OS=Mus musculus GN=Plce1 PE=1 SV=2	2	7	1.0	6.74E+09	2.77E+09	3.66E+09	8.20E+09	6.89E+09	4.90E+09	8.46E+09	3.69E+09	2.96E+09	5.14E+09	3.60E+09	9.11E+09	1.04E+10	0	1.21E-40	0	0	2.61E-38	1.20E-27	0	6.72E-30	4.20E-42	6.73E-41	0	1.35E-34	0	2, 3, 5, 4	13, 7, 8	71	PLCE1_MOUSE, SP:Q8K4S1	SP|Q8K4S1|PLCE1_MOUSE
P11531	DMD_MOUSE	Dystrophin OS=Mus musculus GN=Dmd PE=1 SV=2	21	13	1.0	2.10E+09	2.46E+09	2.23E+09	2.04E+09	2.19E+09	2.33E+09	2.10E+09	2.33E+09	2.25E+09	2.43E+09	2.48E+09	2.32E+09	2.19E+09	2.08E-33	7.54E-37	1.55E-31	3.15E-23	2.29E-22	0	2.85E-28	5.05E-32	1.60E-25	0	1.67E-26	2.19E-29	6.98E-14	2, 3, 4, 4, 5, 7	1, 1, 13, 10, 11, 12, 13, 2, 3, 5, 6, 8	1, 1, 13, 10, 11, 12, 13, 2, 3, 5, 6, 8	DMD_MOUSE, SP:P11531	SP|P11531|DMD_MOUSE
Q62009	POSTN_MOUSE	Periostin OS=Mus musculus GN=Postn PE=1 SV=2	2	1	1.0	5.04E+07	4.36E+07	3.87E+07	5.31E+07	4.68E+07	3.39E+07	4.45E+07	5.57E+07	6.56E+07	4.95E+07	4.79E+07	4.78E+07	8.81E+07	0.025	0.044	0.024	0.062	0.105	0.026	0.046	0.017	0.056	0.020	0.053	0.032	0.048	2	1	1	POSTN_MOUSE, SP:Q62009	SP|Q62009|POSTN_MOUSE
Q8C6K9	CO6A6_MOUSE	Collagen alpha-6(VI) chain OS=Mus musculus GN=Col6a6 PE=1 SV=2	1	3	0.98	2.51E+07	3.78E+07	6.16E+07	3.62E+07	4.46E+07	4.98E+07	7.83E+07	4.45E+07	7.26E+07	5.39E+07	6.41E+07	6.06E+07	2.34E+07	0.080	0.011	0.049	0.065	0.060	0.033	0.011	0.012	0.047	0.008	0.033	0.047	0.195	4	3	3	CO6A6_MOUSE, SP:Q8C6K9	SP|Q8C6K9|CO6A6_MOUSE
Q62407	SPEG_MOUSE	Striated muscle-specific serine/threonine-protein kinase OS=Mus musculus GN=Speg PE=1 SV=2	1	10	1.0	1.06E+08	1.15E+08	1.07E+08	1.09E+08	1.04E+08	1.19E+08	1.02E+08	1.42E+08	1.21E+08	1.05E+08	1.31E+08	1.23E+08	1.12E+08	0.002	0.001	0.002	0.010	0.008	6.26E-01	0.005	0.002	0.010	2.84E-01	0.004	0.001	0.030	5	10	10	SPEG_MOUSE, SP:Q62407	SP|Q62407|SPEG_MOUSE
P01027	CO3_MOUSE	Complement C3 OS=Mus musculus GN=C3 PE=1 SV=2	9	10	1.0	5.78E+09	8.95E+09	8.01E+09	5.98E+09	4.99E+09	6.25E+09	7.81E+09	7.34E+09	6.77E+09	7.45E+09	6.44E+09	5.55E+09	3.10E+09	0	0	0	0	0	0	0	0	0	0	0	0	9.18E-20	2, 3, 4, 5, 8	1, 10, 2, 3, 8, 9	1, 10, 2, 3, 8, 9	CO3_MOUSE, SP:P01027	SP|P01027|CO3_MOUSE
Q9WUR9	KAD4_MOUSE	Adenylate kinase isoenzyme 4, mitochondrial OS=Mus musculus GN=Ak3l1 PE=2 SV=1	2	4	1.0	2.39E+08	1.67E+08	2.44E+08	2.89E+08	2.79E+08	2.31E+08	2.98E+08	1.94E+08	2.15E+08	2.14E+08	1.78E+08	1.94E+08	2.44E+08	7.70E-03	3.04E-03	1.49E-03	7.33E-02	4.09E-01	4.85E-02	4.85E-03	4.83E-02	4.23E-01	3.05E-02	4.85E-01	2.32E-01	0.003	3	3, 4	3, 4	KAD4_MOUSE, SP:Q9WUR9	SP|Q9WUR9|KAD4_MOUSE
Q9Z2M7	PMM2_MOUSE	Phosphomannomutase 2 OS=Mus musculus GN=Pmm2 PE=2 SV=1	1	3	0.94	4.22E+07	2.49E+07	3.64E+07	3.63E+07	4.23E+07	4.30E+07	3.89E+07	4.64E+07	4.94E+07	5.11E+07	2.45E+07	3.45E+07	4.64E+07	0.035	0.040	0.049	0.070	0.075	0.067	0.055	0.031	0.051	0.069	0.083	0.044	0.113	2	3	3	PMM2_MOUSE, SP:Q9Z2M7	SP|Q9Z2M7|PMM2_MOUSE
Q8K4F5	ABHDB_MOUSE	Abhydrolase domain-containing protein 11 OS=Mus musculus GN=Abhd11 PE=2 SV=1	2	11	1.0	5.74E+08	5.58E+08	5.01E+08	4.79E+08	4.38E+08	5.04E+08	4.40E+08	4.62E+08	5.10E+08	4.96E+08	5.43E+08	4.59E+08	3.76E+08	2.00E-08	9.07E-08	1.40E-06	1.55E-03	8.42E-04	1.18E-09	5.99E-05	1.58E-06	2.73E-04	2.19E-09	3.80E-04	5.01E-05	3.89E-01	4	11, 2	11, 2	ABHDB_MOUSE, SP:Q8K4F5	SP|Q8K4F5|ABHDB_MOUSE
Q64669	NQO1_MOUSE	NAD(P)H dehydrogenase [quinone] 1 OS=Mus musculus GN=Nqo1 PE=1 SV=3	1	7	1.0	9.56E+07	1.04E+08	1.10E+08	1.50E+08	1.23E+08	1.33E+08	1.45E+08	1.15E+08	1.31E+08	1.65E+08	1.69E+08	1.45E+08	2.56E+08	0.003	0.001	3.67E-01	0.006	0.006	0.001	9.78E-01	0.001	0.002	4.63E-02	0.002	0.004	0.002	3	7	7	NQO1_MOUSE, SP:Q64669	SP|Q64669|NQO1_MOUSE
Q9CR24	NUDT8_MOUSE	Nucleoside diphosphate-linked moiety X motif 8, mitochondrial OS=Mus musculus GN=Nudt8 PE=1 SV=1	1	2	0.95	6.07E+07	5.13E+07	7.35E+07	7.93E+07	1.02E+08	7.16E+07	9.63E+07	7.84E+07	8.67E+07	8.97E+07	8.56E+07	5.88E+07	1.05E+08	0.015	0.007	0.007	0.011	0.032	0.016	0.006	0.007	0.016	0.003	0.035	0.014	0.035	2	2	2	NUDT8_MOUSE, SP:Q9CR24	SP|Q9CR24|NUDT8_MOUSE
Q9D7J9	ECHD3_MOUSE	Enoyl-CoA hydratase domain-containing protein 3, mitochondrial OS=Mus musculus GN=Echdc3 PE=2 SV=1	2	2	1.0	1.42E+08	1.59E+08	2.31E+08	1.37E+08	2.36E+08	1.96E+08	2.16E+08	1.24E+08	1.83E+08	1.40E+08	1.26E+08	1.47E+08	1.33E+08	4.22E-01	1.14E-02	6.51E-01	2.40E-01	0.001	7.52E-02	8.17E-02	1.63E-01	0.003	3.64E-01	0.002	0.003	0.020	3, 5	1, 2	1, 2	ECHD3_MOUSE, SP:Q9D7J9	SP|Q9D7J9|ECHD3_MOUSE
Q8VDK1	NIT1_MOUSE	Nitrilase homolog 1 OS=Mus musculus GN=Nit1 PE=2 SV=1	1	10	0.99	4.63E+07	7.72E+07	7.08E+07	5.80E+07	3.23E+07	7.21E+07	7.11E+07	6.18E+07	6.32E+07	7.24E+07	7.16E+07	5.28E+07	3.28E+07	0.028	0.007	0.018	0.097	0.031	0.004	0.015	0.017	0.026	0.005	0.042	0.024	0.153	3	10	10	NIT1_MOUSE, SP:Q8VDK1	SP|Q8VDK1|NIT1_MOUSE
Q8VE38	OXND1_MOUSE	Oxidoreductase NAD-binding domain-containing protein 1 OS=Mus musculus GN=Oxnad1 PE=2 SV=2	1	6	0.94	1.84E+07	3.47E+07	2.30E+07	1.75E+07	2.14E+07	2.82E+07	2.25E+07	2.87E+07	2.36E+07	1.73E+07	2.66E+07	1.49E+07	1.53E+07	0.114	0.081	0.124	0.146	0.121	0.038	0.111	0.098	0.171	0.060	0.180	0.089	0.243	2	5	6	OXND1_MOUSE, SP:Q8VE38	SP|Q8VE38|OXND1_MOUSE
Q8K3A0	HSC20_MOUSE	Iron-sulfur cluster co-chaperone protein HscB, mitochondrial OS=Mus musculus GN=Hscb PE=2 SV=1	1	1	0.99	6.53E+07	7.84E+07	6.96E+07	1.10E+08	6.22E+07	6.47E+07	9.18E+07	6.57E+07	5.70E+07	7.16E+07	5.90E+07	5.05E+07	6.84E+07	0.012	0.008	0.002	0.036	0.038	0.004	0.007	0.022	0.026	0.010	0.046	0.021	0.070	3	1	1	HSC20_MOUSE, SP:Q8K3A0	SP|Q8K3A0|HSC20_MOUSE
Q91YY4	ATPF2_MOUSE	ATP synthase mitochondrial F1 complex assembly factor 2 OS=Mus musculus GN=Atpaf2 PE=2 SV=1	1	1	0.88	5.58E+07	1.21E+08	1.01E+08	3.38E+07	4.77E+07	7.65E+07	7.31E+07	6.96E+07	5.98E+07	8.79E+07	9.72E+07	7.93E+07	4.52E+07	0.023	0.002	0.070	0.072	0.031	6.51E-01	0.016	0.022	0.018	0.002	0.019	0.022	0.123	4	1	1	ATPF2_MOUSE, SP:Q91YY4	SP|Q91YY4|ATPF2_MOUSE
Q8C8R3	ANK2_MOUSE	Ankyrin-2 OS=Mus musculus GN=Ank2 PE=1 SV=2	1	2	0.98	8.45E+07	5.26E+07	5.18E+07	7.79E+07	5.76E+07	4.87E+07	4.33E+07	5.99E+07	5.82E+07	3.41E+07	3.75E+07	6.64E+07	3.96E+07	0.005	0.018	0.007	0.042	0.062	0.014	0.045	0.021	0.091	0.033	0.027	0.026	0.131	2	2	2	ANK2_MOUSE, SP:Q8C8R3	SP|Q8C8R3|ANK2_MOUSE
Q3U0B3	DHR11_MOUSE	Dehydrogenase/reductase SDR family member 11 OS=Mus musculus GN=Dhrs11 PE=2 SV=1	1	1	0.79	7.34E+07	4.94E+07	3.55E+07	5.54E+07	5.75E+07	4.13E+07	6.66E+07	4.59E+07	4.83E+07	5.47E+07	3.87E+07	4.67E+07	2.95E+07	0.008	0.042	0.020	0.042	0.079	0.017	0.018	0.032	0.046	0.030	0.053	0.044	0.167	2	1	1	DHR11_MOUSE, SP:Q3U0B3	SP|Q3U0B3|DHR11_MOUSE
Q9D6Y7	MSRA_MOUSE	Peptide methionine sulfoxide reductase OS=Mus musculus GN=Msra PE=1 SV=1	2	13	1.0	2.07E+08	2.31E+08	1.91E+08	2.57E+08	1.59E+08	2.04E+08	2.71E+08	1.90E+08	2.33E+08	2.16E+08	2.50E+08	2.12E+08	2.46E+08	3.45E-02	3.55E-02	6.30E-03	0.002	8.68E-01	2.73E-03	1.37E-02	2.72E-02	4.22E-01	1.11E-03	3.13E-01	2.85E-01	0.003	2, 3	1, 13	1, 13	MSRA_MOUSE, SP:Q9D6Y7	SP|Q9D6Y7|MSRA_MOUSE
Q8BRH0	TMTC3_MOUSE	Transmembrane and TPR repeat-containing protein 3 OS=Mus musculus GN=Tmtc3 PE=2 SV=2	1	10	0.98	1.89E+09	1.95E+09	2.09E+09	1.57E+09	2.15E+09	2.08E+09	1.95E+09	1.95E+09	1.93E+09	1.43E+09	1.40E+09	1.66E+09	1.32E+09	1.89E-28	2.50E-33	2.85E-23	1.26E-21	1.27E-19	1.30E-33	6.21E-25	2.77E-26	1.62E-14	1.53E-24	5.00E-18	6.25E-24	8.59E-08	6	10	10	TMTC3_MOUSE, SP:Q8BRH0	SP|Q8BRH0|TMTC3_MOUSE
P54797	T10_MOUSE	Ser/Thr-rich protein T10 in DGCR region OS=Mus musculus GN=T10 PE=2 SV=1	1	1	0.77	1.22E+08	1.57E+08	1.53E+08	1.02E+08	2.15E+08	1.54E+08	1.45E+08	1.53E+08	1.45E+08	1.68E+08	1.27E+08	1.18E+08	9.72E+07	0.001	1.80E-01	0.003	4.48E-01	0.003	9.02E-02	0.001	7.45E-01	0.002	3.82E-01	0.005	9.87E-01	0.040	5	1	1	T10_MOUSE, SP:P54797	SP|P54797|T10_MOUSE
Q9CZR8	EFTS_MOUSE	Elongation factor Ts, mitochondrial OS=Mus musculus GN=Tsfm PE=2 SV=1	3	9	1.0	8.26E+08	7.96E+08	7.32E+08	6.62E+08	5.95E+08	7.97E+08	6.55E+08	8.04E+08	7.32E+08	6.96E+08	7.64E+08	6.02E+08	6.09E+08	5.40E-13	3.05E-12	7.76E-10	1.52E-05	2.84E-07	1.33E-14	2.66E-08	3.54E-10	9.00E-07	6.32E-14	3.44E-06	6.62E-10	5.53E-03	5, 8	4, 7, 9	4, 7, 9	EFTS_MOUSE, SP:Q9CZR8	SP|Q9CZR8|EFTS_MOUSE
O09131	GSTO1_MOUSE	Glutathione S-transferase omega-1 OS=Mus musculus GN=Gsto1 PE=2 SV=2	4	13	1.0	1.36E+09	1.52E+09	1.57E+09	1.83E+09	1.30E+09	1.68E+09	1.69E+09	1.47E+09	1.71E+09	1.56E+09	1.74E+09	1.52E+09	1.38E+09	1.23E-21	9.38E-27	4.33E-28	2.56E-13	2.18E-16	3.62E-28	1.10E-22	1.05E-24	2.03E-16	3.11E-32	3.36E-17	6.95E-19	2.26E-08	2, 4, 5	11, 13, 12, 2, 9	11, 13, 12, 2, 9	GSTO1_MOUSE, SP:O09131	SP|O09131|GSTO1_MOUSE
Q8K0Z7	TACO1_MOUSE	Translational activator of cytochrome c oxidase 1 OS=Mus musculus GN=Taco1 PE=2 SV=1	2	3	1.0	1.66E+08	2.42E+08	2.51E+08	1.76E+08	1.88E+08	1.80E+08	1.81E+08	1.78E+08	2.34E+08	1.96E+08	2.83E+08	1.77E+08	1.16E+08	1.69E-01	2.83E-03	1.35E-01	9.97E-01	0.002	1.77E-03	2.98E-01	2.70E-02	7.36E-01	2.57E-04	8.73E-01	4.46E-01	0.028	4, 6	1, 1, 3	1, 1, 3	TACO1_MOUSE, SP:Q8K0Z7	SP|Q8K0Z7|TACO1_MOUSE
Q9EPB5	SERHL_MOUSE	Serine hydrolase-like protein OS=Mus musculus GN=Serhl PE=2 SV=1	2	4	1.0	1.40E+08	1.52E+08	1.18E+08	1.24E+08	1.11E+08	1.14E+08	1.29E+08	1.68E+08	1.24E+08	1.48E+08	1.65E+08	1.52E+08	1.34E+08	4.70E-01	8.38E-01	0.001	0.008	0.010	1.05E-01	0.002	0.002	0.003	5.73E-02	0.002	5.68E-01	0.020	2, 6	1, 4	1, 4	SERHL_MOUSE, SP:Q9EPB5	SP|Q9EPB5|SERHL_MOUSE
Q9WVL0	MAAI_MOUSE	Maleylacetoacetate isomerase OS=Mus musculus GN=Gstz1 PE=1 SV=1	3	10	1.0	6.36E+08	7.41E+08	6.62E+08	5.83E+08	7.64E+08	8.27E+08	7.45E+08	5.93E+08	6.13E+08	5.76E+08	6.40E+08	6.19E+08	5.10E+08	9.80E-10	5.42E-11	1.62E-08	1.93E-07	1.35E-07	1.87E-13	1.41E-09	2.67E-08	2.24E-05	1.75E-11	2.15E-06	5.16E-07	2.90E-02	3, 4	7, 8, 9, 10	10, 9, 7, 8	MAAI_MOUSE, SP:Q9WVL0	SP|Q9WVL0|MAAI_MOUSE
Q9JIY5	HTRA2_MOUSE	Serine protease HTRA2, mitochondrial OS=Mus musculus GN=Htra2 PE=1 SV=2	1	5	0.99	8.64E+07	9.73E+07	1.02E+08	1.26E+08	1.25E+08	7.26E+07	1.18E+08	9.68E+07	7.84E+07	1.11E+08	1.07E+08	1.07E+08	1.08E+08	0.005	0.002	0.001	0.005	0.030	0.001	0.003	0.009	0.008	9.18E-01	0.007	0.009	0.032	3	5	5	HTRA2_MOUSE, SP:Q9JIY5	SP|Q9JIY5|HTRA2_MOUSE
Q62000	MIME_MOUSE	Mimecan OS=Mus musculus GN=Ogn PE=2 SV=1	1	3	0.75	5.77E+07	4.46E+07	3.99E+07	4.38E+07	6.43E+07	5.87E+07	4.48E+07	3.03E+07	5.52E+07	5.39E+07	3.44E+07	4.60E+07	4.90E+07	0.017	0.034	0.034	0.034	0.046	0.022	0.043	0.024	0.047	0.039	0.054	0.083	0.107	2	3	3	MIME_MOUSE, SP:Q62000	SP|Q62000|MIME_MOUSE
Q9D1H9	MFAP4_MOUSE	Microfibril-associated glycoprotein 4 OS=Mus musculus GN=Mfap4 PE=1 SV=1	2	13	1.0	4.65E+08	5.22E+08	5.08E+08	4.57E+08	4.79E+08	4.89E+08	5.74E+08	4.83E+08	5.25E+08	5.36E+08	5.41E+08	4.27E+08	3.41E+08	9.84E-07	4.62E-08	2.47E-06	3.69E-04	6.31E-04	4.59E-09	4.98E-07	7.35E-07	7.21E-05	2.31E-09	5.85E-04	1.95E-05	5.18E-01	2, 4, 3	13, 2, 13	13, 13, 2	MFAP4_MOUSE, SP:Q9D1H9	SP|Q9D1H9|MFAP4_MOUSE
P70695	F16P2_MOUSE	Fructose-1,6-bisphosphatase isozyme 2 OS=Mus musculus GN=Fbp2 PE=2 SV=2	1	9	1.0	6.54E+07	7.94E+07	8.43E+07	7.91E+07	1.07E+08	1.12E+08	8.13E+07	9.36E+07	9.48E+07	8.35E+07	9.41E+07	8.44E+07	4.30E+07	0.012	0.004	0.007	0.009	0.010	0.004	0.010	0.005	0.018	0.002	0.015	0.007	0.121	4	9	9	F16P2_MOUSE, SP:P70695	SP|P70695|F16P2_MOUSE
Q60841	RELN_MOUSE	Reelin OS=Mus musculus GN=Reln PE=1 SV=2	1	9	0.99	2.13E+08	1.78E+08	1.72E+08	2.09E+08	2.80E+08	2.61E+08	1.69E+08	1.89E+08	1.70E+08	2.00E+08	1.38E+08	1.62E+08	1.33E+08	3.81E-02	1.30E-01	5.30E-02	8.77E-02	2.49E-01	6.35E-02	5.46E-01	3.81E-01	8.26E-01	3.85E-01	0.002	3.96E-01	0.022	3	9	9	RELN_MOUSE, SP:Q60841	SP|Q60841|RELN_MOUSE
Q3UHB1	NT5D3_MOUSE	5'-nucleotidase domain-containing protein 3 OS=Mus musculus GN=Nt5dc3 PE=2 SV=1	1	6	0.99	5.60E+07	9.50E+07	7.47E+07	6.17E+07	6.91E+07	4.52E+07	8.00E+07	5.84E+07	6.99E+07	7.13E+07	5.57E+07	4.98E+07	4.62E+07	0.018	0.006	0.016	0.030	0.070	0.002	0.011	0.013	0.028	0.013	0.050	0.027	0.114	4	5	6	NT5D3_MOUSE, SP:Q3UHB1	SP|Q3UHB1|NT5D3_MOUSE
Q9D404	OXSM_MOUSE	3-oxoacyl-[acyl-carrier-protein] synthase, mitochondrial OS=Mus musculus GN=Oxsm PE=2 SV=1	2	1	1.0	1.63E+08	1.64E+08	1.51E+08	1.41E+08	1.99E+08	1.94E+08	1.87E+08	1.75E+08	1.77E+08	1.79E+08	1.83E+08	1.90E+08	1.52E+08	2.17E-01	2.27E-01	6.08E-01	7.02E-01	0.001	7.84E-02	2.40E-01	2.19E-01	0.001	2.83E-02	6.15E-01	4.82E-01	0.014	3, 4	1	1	OXSM_MOUSE, SP:Q9D404	SP|Q9D404|OXSM_MOUSE
Q9JLT4	TRXR2_MOUSE	Thioredoxin reductase 2, mitochondrial OS=Mus musculus GN=Txnrd2 PE=1 SV=4	2	8	1.0	1.62E+08	1.35E+08	1.32E+08	1.77E+08	1.62E+08	1.31E+08	1.52E+08	1.37E+08	1.22E+08	1.33E+08	1.16E+08	1.86E+08	1.45E+08	1.85E-01	4.32E-01	1.24E-01	0.002	0.006	2.29E-01	7.91E-01	0.002	0.004	5.92E-01	6.46E-01	0.002	0.016	2, 3	4, 8	4, 8	TRXR2_MOUSE, SP:Q9JLT4	SP|Q9JLT4|TRXR2_MOUSE
P47811	MK14_MOUSE	Mitogen-activated protein kinase 14 OS=Mus musculus GN=Mapk14 PE=1 SV=3	1	3	0.97	5.70E+07	5.27E+07	7.04E+07	6.12E+07	8.20E+07	7.59E+07	7.93E+07	4.42E+07	6.59E+07	4.35E+07	3.20E+07	8.38E+07	6.93E+07	0.022	0.009	0.019	0.024	0.031	0.020	0.014	0.020	0.077	0.062	0.018	0.057	0.074	4	3	3	MK14_MOUSE, SP:P47811	SP|P47811|MK14_MOUSE
Q3URS9	CCD51_MOUSE	Coiled-coil domain-containing protein 51 OS=Mus musculus GN=Ccdc51 PE=2 SV=1	1	11	0.99	1.58E+07	3.97E+07	4.84E+07	4.96E+07	5.75E+07	4.39E+07	5.13E+07	5.27E+07	3.71E+07	5.49E+07	2.76E+07	6.62E+07	3.98E+07	0.132	0.021	0.026	0.042	0.072	0.028	0.032	0.052	0.045	0.056	0.027	0.034	0.130	4	11	11	CCD51_MOUSE, SP:Q3URS9	SP|Q3URS9|CCD51_MOUSE
Q8QZR5	ALAT1_MOUSE	Alanine aminotransferase 1 OS=Mus musculus GN=Gpt PE=2 SV=3	1	3	0.89	6.06E+07	5.25E+07	7.38E+07	9.64E+07	9.74E+07	6.83E+07	6.73E+07	7.02E+07	5.74E+07	7.57E+07	7.18E+07	6.35E+07	5.75E+07	0.016	0.007	0.004	0.013	0.039	0.018	0.020	0.026	0.027	0.008	0.031	0.021	0.090	2	3	3	ALAT1_MOUSE, SP:Q8QZR5	SP|Q8QZR5|ALAT1_MOUSE
P21614	VTDB_MOUSE	Vitamin D-binding protein OS=Mus musculus GN=Gc PE=1 SV=2	3	10	1.0	4.30E+08	5.08E+08	5.76E+08	5.76E+08	6.23E+08	6.65E+08	5.59E+08	5.20E+08	5.90E+08	5.86E+08	5.22E+08	6.00E+08	4.80E+08	3.92E-06	1.99E-09	2.49E-08	7.63E-06	7.34E-06	7.02E-09	7.16E-07	6.47E-08	1.74E-05	3.94E-09	3.74E-06	5.52E-06	4.83E-02	3, 6	10, 2, 3	10, 2, 3	VTDB_MOUSE, SP:P21614	SP|P21614|VTDB_MOUSE
P31428	DPEP1_MOUSE	Dipeptidase 1 OS=Mus musculus GN=Dpep1 PE=1 SV=1	1	5	0.99	9.70E+07	1.29E+08	1.25E+08	8.96E+07	9.11E+07	1.64E+08	1.24E+08	1.34E+08	9.64E+07	1.15E+08	1.48E+08	7.32E+07	6.46E+07	0.003	6.56E-01	0.005	0.015	0.003	3.71E-01	0.002	0.005	0.007	1.38E-01	0.022	0.002	0.076	4	5	5	DPEP1_MOUSE, SP:P31428	SP|P31428|DPEP1_MOUSE
Q3UQ84	SYTM_MOUSE	Threonyl-tRNA synthetase, mitochondrial OS=Mus musculus GN=Tars2 PE=2 SV=1	1	1	0.95	8.63E+06	2.34E+07	1.39E+07	1.01E+07	1.11E+07	8.70E+06	1.30E+07	1.96E+07	1.19E+07	3.69E+06	2.11E+07	5.60E+06	5.59E+06	0.204	0.138	0.189	0.223	0.257	0.071	0.176	0.178	0.330	0.082	0.287	0.132	0.336	2	1	1	SYTM_MOUSE, SP:Q3UQ84	SP|Q3UQ84|SYTM_MOUSE
P40336	VP26A_MOUSE	Vacuolar protein sorting-associated protein 26A OS=Mus musculus GN=Vps26a PE=1 SV=1	1	4	0.99	9.60E+07	9.28E+07	8.48E+07	6.57E+07	1.01E+08	7.29E+07	7.32E+07	5.62E+07	7.21E+07	5.77E+07	8.21E+07	7.35E+07	6.24E+07	0.003	0.004	0.013	0.011	0.030	0.002	0.014	0.012	0.041	0.003	0.021	0.029	0.079	3	4	4	VP26A_MOUSE, SP:P40336	SP|P40336|VP26A_MOUSE
P01873	MUCM_MOUSE	Ig mu chain C region membrane-bound form OS=Mus musculus GN=Igh-6 PE=2 SV=3	1	2	0.99	7.12E+06	1.35E+07	1.49E+07	3.05E+07	3.52E+07	4.73E+07	7.80E+07	3.31E+07	2.98E+07	5.92E+07	4.65E+07	5.67E+07	4.07E+07	0.226	0.130	0.063	0.088	0.064	0.132	0.011	0.072	0.039	0.020	0.037	0.073	0.127	3	2	2	MUCM_MOUSE, SP:P01873	SP|P01873|MUCM_MOUSE,SP|P01872|IGHM_MOUSE
Q14C51	PTCD3_MOUSE	Pentatricopeptide repeat-containing protein 3, mitochondrial OS=Mus musculus GN=Ptcd3 PE=2 SV=2	1	5	0.98	1.25E+07	3.55E+07	3.12E+07	1.84E+07	3.14E+07	2.33E+07	2.03E+07	2.34E+07	2.08E+07	3.89E+07	3.19E+07	2.41E+07	1.01E+07	0.160	0.052	0.118	0.101	0.143	0.036	0.123	0.112	0.077	0.044	0.122	0.111	0.285	2	5	5	PTCD3_MOUSE, SP:Q14C51	SP|Q14C51|PTCD3_MOUSE
P32261	ANT3_MOUSE	Antithrombin-III OS=Mus musculus GN=Serpinc1 PE=1 SV=1	5	7	1.0	4.30E+08	6.31E+08	5.61E+08	3.89E+08	4.51E+08	6.12E+08	6.31E+08	4.79E+08	4.89E+08	4.96E+08	5.81E+08	5.00E+08	3.90E+08	6.43E-06	4.77E-08	5.00E-05	9.42E-04	3.84E-05	4.90E-11	9.58E-08	3.43E-06	2.57E-04	5.22E-10	1.32E-04	2.84E-05	2.69E-01	2, 3, 4	1, 3, 5, 7	1, 3, 5, 7	ANT3_MOUSE, SP:P32261	SP|P32261|ANT3_MOUSE
O08677	KNG1_MOUSE	Kininogen-1 OS=Mus musculus GN=Kng1 PE=1 SV=1	2	11	1.0	2.03E+08	2.91E+08	3.10E+08	3.84E+08	4.02E+08	4.36E+08	3.92E+08	3.07E+08	3.33E+08	4.32E+08	2.91E+08	3.08E+08	5.13E+08	3.59E-02	1.94E-04	4.13E-05	2.86E-03	2.47E-03	1.57E-04	2.17E-04	6.77E-04	1.17E-03	1.67E-04	1.75E-02	5.83E-03	3.12E-02	2	11, 8	11, 8	KNG1_MOUSE, SP:O08677	SP|O08677|KNG1_MOUSE
Q8BH86	CN159_MOUSE	UPF0317 protein C14orf159 homolog, mitochondrial OS=Mus musculus PE=2 SV=1	4	13	1.0	3.30E+08	4.37E+08	3.96E+08	4.23E+08	4.52E+08	4.63E+08	4.55E+08	3.13E+08	3.31E+08	3.02E+08	3.49E+08	3.00E+08	2.21E+08	2.13E-04	4.68E-06	8.26E-06	7.21E-04	1.14E-03	1.63E-07	2.37E-05	6.84E-04	3.69E-02	1.02E-05	2.17E-02	4.35E-03	0.004	2, 3, 4	13, 2, 6, 8	13, 2, 6, 8	CN159_MOUSE, SP:Q8BH86	SP|Q8BH86|CN159_MOUSE
Q9D2N4	DTNA_MOUSE	Dystrobrevin alpha OS=Mus musculus GN=Dtna PE=1 SV=1	1	2	1.0	1.06E+07	3.73E+07	3.18E+07	2.53E+07	3.46E+07	1.23E+07	1.60E+07	4.02E+07	2.96E+07	2.40E+07	3.49E+07	2.23E+07	2.69E+07	0.180	0.050	0.082	0.090	0.219	0.032	0.151	0.073	0.131	0.037	0.131	0.055	0.177	3	2	2	DTNA_MOUSE, SP:Q9D2N4	SP|Q9D2N4|DTNA_MOUSE
Q8BMF3	MAON_MOUSE	NADP-dependent malic enzyme, mitochondrial OS=Mus musculus GN=Me3 PE=1 SV=1	4	11	1.0	1.01E+09	1.07E+09	1.03E+09	9.96E+08	1.08E+09	1.15E+09	1.08E+09	1.20E+09	1.10E+09	1.02E+09	1.11E+09	1.02E+09	9.01E+08	6.78E-16	1.76E-17	3.32E-15	6.16E-11	5.50E-11	9.05E-20	3.10E-14	1.14E-15	2.13E-10	2.03E-20	3.32E-11	3.29E-15	4.84E-05	2, 4, 5	11, 2, 4	11, 2, 4	MAON_MOUSE, SP:Q8BMF3	SP|Q8BMF3|MAON_MOUSE
Q3UV70	PDP1_MOUSE	[Pyruvate dehydrogenase [acetyl-transferring]]-phosphatase 1, mitochondrial OS=Mus musculus GN=Pdp1 PE=2 SV=1	1	4	0.99	2.03E+07	5.05E+07	3.72E+07	2.50E+07	5.21E+07	3.98E+07	4.44E+07	4.42E+07	3.56E+07	3.32E+07	3.59E+07	2.63E+07	1.03E+07	0.102	0.038	0.083	0.050	0.082	0.016	0.043	0.056	0.094	0.035	0.112	0.047	0.284	2	4	4	PDP1_MOUSE, SP:Q3UV70	SP|Q3UV70|PDP1_MOUSE
Q60973	RBBP7_MOUSE	Histone-binding protein RBBP7 OS=Mus musculus GN=Rbbp7 PE=1 SV=1	1	1	0.63	3.58E+07	3.95E+07	3.57E+07	3.26E+07	3.78E+07	4.45E+07	2.77E+07	3.56E+07	3.45E+07	4.16E+07	4.60E+07	2.80E+07	2.32E+07	0.046	0.041	0.057	0.081	0.071	0.029	0.087	0.059	0.070	0.020	0.105	0.066	0.195	2	1	1	RBBP7_MOUSE, SP:Q60973	SP|Q60973|RBBP7_MOUSE,SP|Q60972|RBBP4_MOUSE
Q9DBF1	AL7A1_MOUSE	Alpha-aminoadipic semialdehyde dehydrogenase OS=Mus musculus GN=Aldh7a1 PE=2 SV=3	1	4	0.99	4.74E+07	8.75E+07	6.88E+07	4.41E+07	4.04E+07	8.57E+07	6.47E+07	9.49E+07	4.75E+07	7.37E+07	6.10E+07	3.75E+07	2.96E+07	0.027	0.008	0.034	0.075	0.021	0.003	0.020	0.034	0.025	0.010	0.074	0.007	0.167	2	4	4	AL7A1_MOUSE, SP:Q9DBF1	SP|Q9DBF1|AL7A1_MOUSE
Q03734	SPA3M_MOUSE	Serine protease inhibitor A3M OS=Mus musculus GN=Serpina3m PE=1 SV=1	1	13	1.0	1.35E+09	1.01E+09	1.60E+09	1.67E+09	2.13E+09	2.33E+09	1.65E+09	1.10E+09	2.87E+09	1.43E+09	1.54E+09	1.64E+09	1.60E+09	2.00E-21	2.66E-27	9.81E-26	3.34E-22	8.32E-23	1.69E-18	4.76E-22	1.96E-40	6.53E-15	3.71E-28	1.62E-18	6.81E-14	7.73E-10	6	13	13	SPA3M_MOUSE, SP:Q03734	SP|Q03734|SPA3M_MOUSE
Q8BYM8	SYCM_MOUSE	Probable cysteinyl-tRNA synthetase, mitochondrial OS=Mus musculus GN=Cars2 PE=2 SV=2	1	3	0.62	1.08E+08	5.56E+07	7.95E+07	7.16E+07	5.41E+07	5.11E+07	5.79E+07	7.05E+07	5.96E+07	5.75E+07	5.76E+07	6.05E+07	5.71E+07	0.002	0.005	0.010	0.047	0.057	0.012	0.025	0.020	0.041	0.011	0.033	0.017	0.089	3	3	3	SYCM_MOUSE, SP:Q8BYM8	SP|Q8BYM8|SYCM_MOUSE
Q8VBT1	TXLNB_MOUSE	Beta-taxilin OS=Mus musculus GN=Txlnb PE=2 SV=2	1	6	1.0	5.93E+07	4.86E+07	8.19E+07	6.07E+07	2.51E+07	3.10E+06	3.51E+07	1.48E+07	3.51E+07	2.85E+07	3.92E+07	1.00E+08	6.91E+07	0.015	0.004	0.016	0.127	0.349	0.017	0.063	0.057	0.111	0.029	0.009	0.165	0.069	4	6	6	TXLNB_MOUSE, SP:Q8VBT1	SP|Q8VBT1|TXLNB_MOUSE
P10637	TAU_MOUSE	Microtubule-associated protein tau OS=Mus musculus GN=Mapt PE=1 SV=3	1	1	0.99	2.64E+07	2.27E+07	2.07E+07	2.28E+07	1.84E+07	1.34E+07	1.64E+07	2.05E+07	3.17E+07	2.02E+07	3.07E+07	1.15E+07	1.33E+07	0.074	0.093	0.093	0.164	0.209	0.074	0.148	0.066	0.151	0.047	0.211	0.127	0.257	2	1	1	TAU_MOUSE, SP:P10637	SP|P10637|TAU_MOUSE
Q8K411	PREP_MOUSE	Presequence protease, mitochondrial OS=Mus musculus GN=Pitrm1 PE=2 SV=1	1	9	0.77	5.69E+07	4.51E+07	3.62E+07	3.79E+07	3.84E+07	3.65E+07	3.26E+07	4.15E+07	5.22E+07	3.66E+07	5.74E+07	3.17E+07	4.21E+07	0.018	0.041	0.045	0.080	0.092	0.022	0.071	0.027	0.084	0.011	0.092	0.053	0.124	2	9	9	PREP_MOUSE, SP:Q8K411	SP|Q8K411|PREP_MOUSE
Q8CC35	SYNPO_MOUSE	Synaptopodin OS=Mus musculus GN=Synpo PE=1 SV=2	1	1	0.99	3.95E+07	5.63E+07	3.80E+07	2.70E+07	4.35E+07	3.75E+07	2.67E+07	4.50E+07	4.58E+07	5.11E+07	6.15E+07	4.25E+07	5.63E+07	0.039	0.037	0.077	0.067	0.089	0.012	0.093	0.036	0.051	0.009	0.061	0.047	0.091	3	1	1	SYNPO_MOUSE, SP:Q8CC35	SP|Q8CC35|SYNPO_MOUSE

O88492	PLIN4_MOUSE	Perilipin-4 OS=Mus musculus GN=Plin4 PE=1 SV=1	4	12	1.0	3.50E+09	5.84E+09	2.32E+09	3.39E+09	6.32E+09	4.48E+09	3.93E+09	5.33E+09	4.46E+09	5.68E+09	3.04E+09	2.41E+09	1.50E+09	0	9.48E-38	0	0	5.61E-42	0	0	0	0	0	5.96E-27	0	4.13E-09	3, 8	10, 12, 6	10, 12, 6	PLIN4_MOUSE, SP:O88492	SP|O88492|PLIN4_MOUSE
Q65CL1	CTNA3_MOUSE	Catenin alpha-3 OS=Mus musculus GN=Ctnna3 PE=1 SV=2	2	2	1.0	1.85E+08	2.31E+08	2.03E+08	1.66E+08	1.49E+08	1.48E+08	1.77E+08	2.12E+08	2.01E+08	1.76E+08	2.41E+08	2.18E+08	2.39E+08	6.84E-02	1.73E-02	1.87E-01	0.003	0.004	2.22E-03	3.09E-01	7.86E-02	0.001	1.45E-03	2.31E-01	1.24E-01	0.003	4	1, 2	1, 2	CTNA3_MOUSE, SP:Q65CL1	SP|Q65CL1|CTNA3_MOUSE
P04186	CFAB_MOUSE	Complement factor B OS=Mus musculus GN=Cfb PE=1 SV=2	1	1	0.99	2.61E+07	5.30E+07	3.23E+07	3.52E+07	1.88E+07	4.75E+07	3.65E+07	3.71E+07	4.30E+07	3.36E+07	4.37E+07	2.46E+07	6.92E+06	0.075	0.049	0.050	0.161	0.064	0.014	0.060	0.040	0.092	0.023	0.120	0.062	0.319	4	1	1	CFAB_MOUSE, SP:P04186	SP|P04186|CFAB_MOUSE
Q9ET54	PALLD_MOUSE	Palladin OS=Mus musculus GN=Palld PE=1 SV=2	1	2	0.96	2.81E+07	2.50E+07	2.77E+07	3.45E+07	2.29E+07	2.32E+07	2.23E+07	4.40E+07	3.23E+07	2.15E+07	2.92E+07	3.05E+07	4.85E+07	0.068	0.063	0.052	0.137	0.144	0.065	0.112	0.065	0.144	0.051	0.095	0.047	0.107	2	1	2	PALLD_MOUSE, SP:Q9ET54	SP|Q9ET54|PALLD_MOUSE
Q8R2Q4	RRF2M_MOUSE	Ribosome-releasing factor 2, mitochondrial OS=Mus musculus GN=Gfm2 PE=2 SV=2	1	1	0.99	4.01E+07	4.79E+07	6.72E+07	4.93E+07	6.11E+07	6.73E+07	6.05E+07	7.14E+07	7.33E+07	5.94E+07	8.12E+07	7.57E+07	8.69E+07	0.039	0.009	0.027	0.038	0.036	0.019	0.023	0.012	0.039	0.003	0.020	0.017	0.048	4	1	1	RRF2M_MOUSE, SP:Q8R2Q4	SP|Q8R2Q4|RRF2M_MOUSE
Q5SW19	K0664_MOUSE	Protein KIAA0664 OS=Mus musculus GN=Kiaa0664 PE=2 SV=2	2	7	1.0	1.21E+08	7.40E+07	7.70E+07	1.02E+08	7.58E+07	6.06E+07	1.04E+08	8.27E+07	9.31E+07	7.29E+07	9.54E+07	6.52E+07	5.65E+07	0.001	0.006	0.003	0.025	0.044	0.005	0.005	0.005	0.027	0.002	0.029	0.012	0.092	2, 3	3, 7	3, 7	K0664_MOUSE, SP:Q5SW19	SP|Q5SW19|K0664_MOUSE
Q3V0Q1	DYH12_MOUSE	Dynein heavy chain 12, axonemal OS=Mus musculus GN=Dnah12 PE=2 SV=2	1	4	0.67	3.16E+06	0.00E+00	9.26E+06	1.08E+07	8.42E+06	1.60E+06	0.00E+00	1.23E+06	0.00E+00	0.00E+00	1.64E+07	0.00E+00	4.96E+06	0.317	0.194	0.186	0.257	0.403	0.500	0.500	0.500	0.500	0.113	0.500	0.408	0.346	2	4	4	DYH12_MOUSE, SP:Q3V0Q1	SP|Q3V0Q1|DYH12_MOUSE
O55111	DSG2_MOUSE	Desmoglein-2 OS=Mus musculus GN=Dsg2 PE=1 SV=2	1	8	0.96	1.53E+07	2.23E+07	2.11E+07	2.00E+07	2.73E+07	2.77E+07	2.15E+07	2.04E+07	1.43E+07	1.43E+07	1.21E+07	2.38E+07	1.77E+07	0.136	0.091	0.108	0.117	0.123	0.077	0.116	0.157	0.193	0.147	0.124	0.127	0.227	2	8	8	DSG2_MOUSE, SP:O55111	SP|O55111|DSG2_MOUSE
Q9WU79	PROD_MOUSE	Proline dehydrogenase, mitochondrial OS=Mus musculus GN=Prodh PE=2 SV=1	1	1	0.99	5.22E+07	9.03E+07	2.09E+07	5.12E+07	1.65E+07	5.30E+07	4.54E+07	6.06E+07	2.58E+07	3.85E+07	3.68E+07	3.12E+07	2.08E+07	0.021	0.092	0.024	0.177	0.054	0.002	0.041	0.088	0.078	0.033	0.093	0.025	0.208	4	1	1	PROD_MOUSE, SP:Q9WU79	SP|Q9WU79|PROD_MOUSE
Q8BH61	F13A_MOUSE	Coagulation factor XIII A chain OS=Mus musculus GN=F13a1 PE=2 SV=3	1	1	0.99	3.42E+07	5.91E+07	3.81E+07	3.12E+07	3.70E+07	3.63E+07	2.74E+07	4.72E+07	4.81E+07	5.80E+07	4.46E+07	1.86E+07	2.39E+07	0.051	0.037	0.062	0.084	0.093	0.010	0.090	0.033	0.041	0.022	0.155	0.042	0.192	3	1	1	F13A_MOUSE, SP:Q8BH61	SP|Q8BH61|F13A_MOUSE
Q9CQL5	RM18_MOUSE	39S ribosomal protein L18, mitochondrial OS=Mus musculus GN=Mrpl18 PE=2 SV=1	1	6	0.97	4.96E+07	4.30E+07	5.04E+07	4.28E+07	4.82E+07	4.29E+07	3.98E+07	3.22E+07	3.81E+07	4.08E+07	5.27E+07	4.01E+07	4.49E+07	0.025	0.020	0.036	0.057	0.075	0.024	0.053	0.050	0.072	0.015	0.067	0.077	0.116	2	6	6	RM18_MOUSE, SP:Q9CQL5	SP|Q9CQL5|RM18_MOUSE
Q9DB27	MCTS1_MOUSE	Malignant T cell-amplified sequence 1 OS=Mus musculus GN=Mcts1 PE=2 SV=1	1	1	0.77	2.48E+07	3.20E+07	3.89E+07	1.78E+07	3.89E+07	3.43E+07	5.42E+07	4.59E+07	6.09E+07	3.38E+07	2.92E+07	5.25E+07	2.33E+07	0.081	0.035	0.121	0.078	0.098	0.043	0.029	0.019	0.091	0.051	0.043	0.044	0.194	4	1	1	MCTS1_MOUSE, SP:Q9DB27	SP|Q9DB27|MCTS1_MOUSE
Q920Q6	MSI2H_MOUSE	RNA-binding protein Musashi homolog 2 OS=Mus musculus GN=Msi2 PE=1 SV=1	1	1	0.63	3.05E+07	6.48E+07	6.11E+07	5.04E+07	5.01E+07	7.83E+07	5.23E+07	5.90E+07	5.07E+07	4.62E+07	4.83E+07	2.74E+07	3.97E+07	0.128	0.041	0.055	0.100	0.047	0.045	0.068	0.067	0.105	0.078	0.164	0.065	0.162	4	1	1	MSI2H_MOUSE, SP:Q920Q6	SP|Q920Q6|MSI2H_MOUSE
Q8C0M9	ASGL1_MOUSE	L-asparaginase OS=Mus musculus GN=Asrgl1 PE=1 SV=1	3	2	1.0	2.05E+08	1.89E+08	2.04E+08	2.40E+08	2.45E+08	2.16E+08	2.25E+08	1.84E+08	2.09E+08	2.42E+08	1.84E+08	2.14E+08	2.43E+08	3.07E-02	1.71E-02	9.74E-03	1.84E-01	6.01E-01	1.64E-02	5.79E-02	5.83E-02	1.92E-01	2.25E-02	2.68E-01	3.23E-01	0.003	2	1, 2	1, 2	ASGL1_MOUSE, SP:Q8C0M9	SP|Q8C0M9|ASGL1_MOUSE
Q3TBW2	RM10_MOUSE	39S ribosomal protein L10, mitochondrial OS=Mus musculus GN=Mrpl10 PE=2 SV=2	1	2	0.99	6.06E+07	1.10E+08	8.86E+07	8.03E+07	8.92E+07	8.58E+07	9.41E+07	9.20E+07	1.11E+08	1.01E+08	7.81E+07	8.64E+07	5.01E+07	0.015	0.003	0.007	0.016	0.021	8.01E-01	0.007	0.003	0.011	0.004	0.014	0.008	0.105	5	2	2	RM10_MOUSE, SP:Q3TBW2	SP|Q3TBW2|RM10_MOUSE
P03893	NU2M_MOUSE	NADH-ubiquinone oxidoreductase chain 2 OS=Mus musculus GN=Mtnd2 PE=1 SV=1	1	13	1.0	4.66E+08	6.75E+08	6.68E+08	5.55E+08	4.95E+08	6.55E+08	4.68E+08	6.46E+08	5.08E+08	5.90E+08	7.54E+08	5.18E+08	4.63E+08	1.04E-06	6.73E-11	6.16E-08	2.46E-04	1.18E-05	6.27E-12	1.73E-05	1.43E-06	1.85E-05	1.92E-13	4.49E-05	1.23E-07	6.76E-02	4, 5	11, 13	11, 13	NU2M_MOUSE, SP:P03893	SP|P03893|NU2M_MOUSE
Q9D9R9	F186A_MOUSE	Protein FAM186A OS=Mus musculus GN=FAM186A PE=2 SV=2	1	9	1.0	4.94E+08	7.54E+08	7.94E+08	4.60E+08	6.02E+08	5.97E+08	5.06E+08	5.03E+08	6.12E+08	5.39E+08	5.67E+08	5.47E+08	3.83E+08	2.10E-06	4.82E-12	7.16E-06	6.58E-05	1.30E-04	3.74E-12	1.42E-05	1.82E-07	1.87E-04	3.61E-09	6.12E-05	3.52E-05	3.21E-01	4	9	9	F186A_MOUSE, SP:Q9D9R9	SP|Q9D9R9|F186A_MOUSE
Q6PD03	2A5A_MOUSE	Serine/threonine-protein phosphatase 2A 56 kDa regulatory subunit alpha isoform OS=Mus musculus GN=Ppp2r5a PE=1 SV=1	2	9	1.0	1.32E+08	9.61E+07	1.08E+08	1.03E+08	1.06E+08	1.05E+08	9.89E+07	1.10E+08	1.24E+08	1.30E+08	8.14E+07	1.03E+08	1.07E+08	6.70E-01	0.001	0.003	0.010	0.012	0.002	0.005	0.002	0.005	0.003	0.008	0.004	0.033	2	1, 4	1, 9	2A5A_MOUSE, SP:Q6PD03	SP|Q6PD03|2A5A_MOUSE
Q9ES83	POPD1_MOUSE	Blood vessel epicardial substance OS=Mus musculus GN=Bves PE=2 SV=1	2	6	1.0	1.65E+08	1.69E+08	1.08E+08	1.19E+08	1.22E+08	1.11E+08	9.75E+07	1.43E+08	1.14E+08	1.58E+08	1.11E+08	1.12E+08	1.05E+08	1.56E-01	0.001	0.001	0.006	0.010	4.42E-02	0.006	0.002	0.002	7.43E-01	0.006	0.001	0.034	2, 3	2, 6	2, 6	POPD1_MOUSE, SP:Q9ES83	SP|Q9ES83|POPD1_MOUSE
Q6A0A9	F120A_MOUSE	Constitutive coactivator of PPAR-gamma-like protein 1 OS=Mus musculus GN=FAM120A PE=1 SV=2	1	2	0.99	3.90E+07	2.53E+07	1.27E+07	1.94E+07	1.50E+07	2.20E+07	1.10E+07	2.89E+07	2.35E+07	2.30E+07	3.00E+07	1.62E+07	2.17E+07	0.041	0.153	0.114	0.191	0.151	0.065	0.199	0.099	0.138	0.050	0.172	0.089	0.204	2	2	2	F120A_MOUSE, SP:Q6A0A9	SP|Q6A0A9|F120A_MOUSE
Q00896	A1AT1_MOUSE	Alpha-1-antitrypsin 1-3 OS=Mus musculus GN=Serpina1c PE=1 SV=2	2	13	1.0	1.03E+09	6.72E+08	9.68E+08	1.11E+09	1.08E+09	1.16E+09	1.34E+09	6.35E+08	1.03E+09	1.00E+09	7.53E+08	1.39E+09	8.15E+08	4.23E-16	2.83E-16	7.74E-17	6.61E-11	5.57E-11	5.15E-12	1.06E-17	1.86E-14	4.04E-10	1.58E-13	1.90E-15	1.59E-07	2.08E-04	3, 3, 6	13, 2, 13	13, 13, 2	A1AT1_MOUSE, SP:P07758	SP|P07758|A1AT1_MOUSE,SP|Q00896|A1AT3_MOUSE
Q8VDT9	RM50_MOUSE	39S ribosomal protein L50, mitochondrial OS=Mus musculus GN=Mrpl50 PE=2 SV=2	1	1	0.99	1.00E+08	8.97E+07	9.05E+07	6.72E+07	6.37E+07	1.33E+08	1.13E+08	9.62E+07	1.07E+08	1.17E+08	1.03E+08	6.97E+07	7.58E+07	0.003	0.004	0.014	0.039	0.006	0.003	0.004	0.004	0.008	0.002	0.026	0.009	0.062	4	1	1	RM50_MOUSE, SP:Q8VDT9	SP|Q8VDT9|RM50_MOUSE
P28653	PGS1_MOUSE	Biglycan OS=Mus musculus GN=Bgn PE=2 SV=1	1	1	0.99	8.22E+07	1.34E+08	1.05E+08	9.56E+07	9.41E+07	1.16E+08	1.13E+08	1.63E+08	1.38E+08	1.38E+08	1.50E+08	8.68E+07	1.27E+08	0.006	0.002	0.004	0.015	0.012	4.42E-01	0.004	0.001	0.004	1.94E-01	0.015	9.13E-01	0.024	3	1	1	PGS1_MOUSE, SP:P28653	SP|P28653|PGS1_MOUSE
Q9QUH0	GLRX1_MOUSE	Glutaredoxin-1 OS=Mus musculus GN=Glrx PE=1 SV=3	2	7	1.0	3.87E+08	3.53E+08	4.06E+08	3.42E+08	3.61E+08	3.48E+08	3.18E+08	3.31E+08	3.92E+08	3.70E+08	4.28E+08	3.66E+08	2.76E+08	1.15E-04	2.95E-05	9.02E-04	2.35E-02	5.56E-02	1.09E-04	6.44E-03	2.45E-04	1.36E-02	3.47E-06	7.42E-03	8.16E-03	0.002	2, 6	1, 7	1, 7	GLRX1_MOUSE, SP:Q9QUH0	SP|Q9QUH0|GLRX1_MOUSE
P43276	H15_MOUSE	Histone H1.5 OS=Mus musculus GN=Hist1h1b PE=1 SV=2	1	1	0.85	9.80E+07	3.73E+07	1.01E+08	7.45E+07	7.28E+07	4.73E+07	5.00E+07	6.82E+07	6.73E+07	5.68E+07	6.28E+07	4.14E+07	4.83E+07	0.005	0.003	0.012	0.029	0.071	0.044	0.044	0.017	0.047	0.012	0.068	0.021	0.113	3	1	1	H15_MOUSE, SP:P43276	SP|P43276|H15_MOUSE
Q8CBW3	ABI1_MOUSE	Abl interactor 1 OS=Mus musculus GN=Abi1 PE=1 SV=3	1	13	0.95	3.77E+08	2.77E+08	2.09E+08	4.58E+08	4.39E+08	3.72E+08	5.68E+08	2.66E+08	4.09E+08	3.64E+08	3.86E+08	3.03E+08	2.92E+08	3.73E-05	1.47E-02	2.92E-06	1.17E-03	1.25E-02	3.25E-04	8.55E-07	5.19E-05	7.45E-03	2.80E-06	2.09E-02	2.24E-02	0.001	4	13	13	ABI1_MOUSE, SP:Q8CBW3	SP|Q8CBW3|ABI1_MOUSE
Q62383	SPT6H_MOUSE	Transcription elongation factor SPT6 OS=Mus musculus GN=Supt6h PE=1 SV=2	1	4	0.91	9.27E+07	1.41E+08	4.77E+07	1.63E+08	1.81E+08	1.25E+08	8.61E+07	9.92E+07	1.25E+08	1.60E+08	1.02E+08	6.94E+07	1.10E+08	0.004	0.023	2.55E-01	0.001	0.008	2.13E-01	0.009	0.002	0.002	0.001	0.025	0.007	0.032	2	4	4	SPT6H_MOUSE, SP:Q62383	SP|Q62383|SPT6H_MOUSE
Q7TST5	LAMP3_MOUSE	Lysosome-associated membrane glycoprotein 3 OS=Mus musculus GN=Lamp3 PE=2 SV=1	1	8	0.67	6.25E+08	4.93E+08	4.93E+08	5.58E+08	6.94E+08	6.49E+08	5.47E+08	5.62E+08	6.33E+08	5.76E+08	5.13E+08	6.11E+08	6.39E+08	1.96E-09	7.68E-08	5.12E-08	1.31E-06	1.21E-05	1.49E-08	1.17E-06	1.53E-08	2.48E-05	7.04E-09	3.03E-06	1.53E-06	3.61E-03	7	8	8	LAMP3_MOUSE, SP:Q7TST5	SP|Q7TST5|LAMP3_MOUSE
P06330	HVM51_MOUSE	Ig heavy chain V region AC38 205.12 OS=Mus musculus PE=1 SV=1	1	1	1.0	1.90E+07	1.82E+07	1.84E+07	7.64E+07	2.22E+07	9.07E+07	1.06E+08	1.05E+07	8.53E+06	1.80E+08	7.51E+07	5.49E+07	8.95E+07	0.121	0.112	0.008	0.147	0.018	0.107	0.004	0.233	0.001	0.005	0.042	0.220	0.047	2	1	1	HVM51_MOUSE, SP:P06330	SP|P06330|HVM51_MOUSE
Q8BUE4	AIFM2_MOUSE	Apoptosis-inducing factor 2 OS=Mus musculus GN=Aifm2 PE=2 SV=1	1	8	0.67	6.86E+08	8.09E+08	8.66E+08	7.59E+08	5.30E+08	7.23E+08	6.98E+08	8.18E+08	6.72E+08	6.33E+08	7.10E+08	1.14E+09	1.12E+09	4.82E-10	8.62E-14	9.20E-11	1.32E-04	2.86E-06	4.91E-14	1.73E-08	8.51E-09	7.82E-06	3.47E-12	3.39E-12	9.84E-10	2.10E-06	5	8	8	AIFM2_MOUSE, SP:Q8BUE4	SP|Q8BUE4|AIFM2_MOUSE
Q9CY27	TECR_MOUSE	Trans-2,3-enoyl-CoA reductase OS=Mus musculus GN=Tecr PE=1 SV=1	1	13	0.97	3.80E+07	4.82E+07	3.91E+07	4.42E+07	6.01E+07	6.73E+07	4.29E+07	5.17E+07	5.95E+07	5.10E+07	6.01E+07	6.18E+07	6.64E+07	0.042	0.034	0.033	0.039	0.035	0.018	0.046	0.020	0.051	0.010	0.031	0.035	0.073	3	13	13	TECR_MOUSE, SP:Q9CY27	SP|Q9CY27|TECR_MOUSE
P01864	GCAB_MOUSE	Ig gamma-2A chain C region secreted form OS=Mus musculus PE=1 SV=1	1	3	1.0	1.46E+07	5.67E+07	3.32E+07	9.19E+07	2.65E+07	1.79E+08	1.82E+08	2.02E+07	2.32E+07	2.07E+08	1.08E+08	7.97E+07	1.59E+08	0.142	0.047	0.004	0.120	0.002	0.011	2.62E-01	0.099	5.12E-01	8.70E-01	0.017	0.128	0.012	4	3	3	GCAB_MOUSE, SP:P01864	SP|P01864|GCAB_MOUSE
O35598	ADA10_MOUSE	Disintegrin and metalloproteinase domain-containing protein 10 OS=Mus musculus GN=Adam10 PE=1 SV=1	1	10	0.97	3.94E+09	1.00E+10	3.94E+09	5.25E+09	9.06E+09	2.34E+09	1.09E+09	5.75E+09	6.52E+09	7.65E+09	3.82E+09	4.25E+09	4.38E+09	0	0	0	0	9.56E-23	0	2.18E-14	0	0	0	0	0	1.10E-27	10	10	10	ADA10_MOUSE, SP:O35598	SP|O35598|ADA10_MOUSE
Q8CE46	PUS7L_MOUSE	Pseudouridylate synthase 7 homolog-like protein OS=Mus musculus GN=Pus7l PE=1 SV=1	1	8	0.95	8.07E+09	4.74E+09	6.59E+09	6.27E+09	7.30E+09	7.06E+09	7.11E+09	5.33E+09	8.51E+09	5.64E+09	3.42E+09	6.81E+09	8.19E+09	0	0	0	0	0	0	0	0	0	0	0	0	0	6	7	8	PUS7L_MOUSE, SP:Q8CE46	SP|Q8CE46|PUS7L_MOUSE
Q99L02	PA1_MOUSE	PAXIP1-associated protein 1 OS=Mus musculus GN=Pa1 PE=1 SV=1	1	9	0.92	5.98E+08	2.85E+08	4.39E+08	5.06E+08	5.25E+08	5.18E+08	4.59E+08	3.56E+08	5.74E+08	4.45E+08	4.92E+08	4.99E+08	7.30E+08	7.83E-09	9.21E-07	4.56E-07	1.19E-04	3.35E-04	2.12E-04	2.45E-05	1.59E-07	8.53E-04	2.33E-08	8.15E-05	1.18E-03	8.56E-04	5	9	9	PA1_MOUSE, SP:Q99L02	SP|Q99L02|PA1_MOUSE
Q99K51	PLST_MOUSE	Plastin-3 OS=Mus musculus GN=Pls3 PE=1 SV=3	1	3	0.76	6.99E+07	1.15E+08	9.44E+07	7.39E+07	6.55E+07	9.35E+07	4.87E+07	1.43E+08	1.18E+08	1.21E+08	9.89E+07	6.09E+07	8.89E+07	0.013	0.003	0.011	0.038	0.019	9.27E-01	0.043	0.002	0.007	0.002	0.038	0.002	0.048	3	3	3	PLST_MOUSE, SP:Q99K51	SP|Q99K51|PLST_MOUSE,SP|Q61233|PLSL_MOUSE
P61148	FGF1_MOUSE	Heparin-binding growth factor 1 OS=Mus musculus GN=Fgf1 PE=2 SV=1	1	12	0.99	6.39E+07	5.22E+07	6.06E+07	6.15E+07	6.81E+07	6.98E+07	7.75E+07	5.68E+07	4.73E+07	5.73E+07	2.92E+07	7.41E+07	5.44E+07	0.012	0.012	0.015	0.030	0.033	0.014	0.012	0.033	0.042	0.051	0.021	0.029	0.094	4	12	12	FGF1_MOUSE, SP:P61148	SP|P61148|FGF1_MOUSE
Q05769	PGH2_MOUSE	Prostaglandin G/H synthase 2 OS=Mus musculus GN=Ptgs2 PE=1 SV=1	1	5	0.88	5.72E+08	1.57E+09	7.89E+08	6.19E+08	6.31E+08	6.42E+08	7.22E+08	1.31E+09	4.97E+08	1.06E+09	5.66E+08	7.92E+08	6.77E+08	2.72E-08	1.10E-12	1.04E-08	9.30E-06	1.83E-05	3.10E-27	6.62E-09	2.75E-06	1.70E-10	1.48E-09	3.05E-08	5.74E-16	2.19E-03	4	5	5	PGH2_MOUSE, SP:Q05769	SP|Q05769|PGH2_MOUSE
Q8C115	PKHH2_MOUSE	Pleckstrin homology domain-containing family H member 2 OS=Mus musculus GN=Plekhh2 PE=2 SV=2	1	13	0.95	3.15E+08	2.09E+08	3.02E+08	2.91E+08	4.39E+08	3.97E+08	4.70E+08	2.18E+08	3.31E+08	3.17E+08	1.80E+08	4.38E+08	3.60E+08	4.24E-04	2.93E-04	1.57E-03	1.17E-03	6.71E-03	7.26E-03	1.94E-05	7.83E-04	2.61E-02	2.86E-02	4.90E-04	1.09E-01	3.83E-01	4	13	13	PKHH2_MOUSE, SP:Q8C115	SP|Q8C115|PKHH2_MOUSE
Q91ZT5	FGD4_MOUSE	FYVE, RhoGEF and PH domain-containing protein 4 OS=Mus musculus GN=Fgd4 PE=1 SV=1	1	11	0.94	4.16E+08	4.03E+08	4.21E+08	3.80E+08	3.82E+08	3.94E+08	3.89E+08	3.86E+08	3.85E+08	3.57E+08	3.41E+08	4.32E+08	3.66E+08	7.82E-06	1.79E-06	4.76E-05	4.89E-03	7.02E-03	1.07E-06	2.46E-04	1.06E-04	8.88E-03	1.78E-05	5.31E-04	4.61E-04	3.43E-01	6	11	11	FGD4_MOUSE, SP:Q91ZT5	SP|Q91ZT5|FGD4_MOUSE
Q9DBS4	ACOXL_MOUSE	Acyl-coenzyme A oxidase-like protein OS=Mus musculus GN=Acoxl PE=2 SV=1	1	3	0.92	6.61E+07	6.54E+07	9.38E+07	8.96E+07	1.27E+08	1.35E+08	1.09E+08	4.62E+07	1.30E+08	8.80E+07	1.25E+08	9.14E+07	8.38E+07	0.013	0.003	0.005	0.005	0.006	0.009	0.004	0.001	0.018	4.75E-01	0.013	0.050	0.052	3	3	3	ACOXL_MOUSE, SP:Q9DBS4	SP|Q9DBS4|ACOXL_MOUSE
Q8BND5	QSOX1_MOUSE	Sulfhydryl oxidase 1 OS=Mus musculus GN=Qsox1 PE=2 SV=1	1	2	0.86	4.35E+08	1.88E+08	4.98E+08	5.67E+08	1.56E+08	2.37E+08	3.40E+08	1.32E+08	3.01E+08	2.98E+08	2.15E+08	2.58E+08	1.77E+08	6.67E-06	2.35E-07	1.29E-07	0.002	3.97E-01	2.23E-02	1.61E-03	2.79E-03	4.86E-02	6.78E-03	8.44E-02	0.002	0.009	5	2	2	QSOX1_MOUSE, SP:Q8BND5	SP|Q8BND5|QSOX1_MOUSE
P86049	RBM46_MOUSE	Probable RNA-binding protein 46 OS=Mus musculus GN=Rbm46 PE=4 SV=1	1	2	0.98	1.32E+09	1.26E+08	2.78E+09	4.40E+08	5.99E+07	7.47E+07	8.20E+07	2.21E+09	1.33E+08	1.43E+08	6.24E+07	4.74E+07	1.06E+08	2.62E-20	0	5.58E-06	0.043	0.033	5.77E-01	0.012	0.001	0.004	0.012	0.058	3.58E-27	0.035	6	2	2	RBM46_MOUSE, SP:P86049	SP|P86049|RBM46_MOUSE
Q61087	LAMB3_MOUSE	Laminin subunit beta-3 OS=Mus musculus GN=Lamb3 PE=2 SV=1	1	5	0.81	1.97E+09	1.91E+09	1.57E+09	1.73E+09	1.89E+09	1.37E+09	1.59E+09	1.63E+09	1.45E+09	1.79E+09	1.86E+09	1.73E+09	1.50E+09	9.85E-29	5.81E-25	8.73E-25	9.94E-19	8.78E-13	3.61E-32	4.61E-20	1.28E-19	7.09E-18	3.33E-31	1.62E-18	8.40E-20	6.67E-09	5	5	5	LAMB3_MOUSE, SP:Q61087	SP|Q61087|LAMB3_MOUSE
Q61831	MK10_MOUSE	Mitogen-activated protein kinase 10 OS=Mus musculus GN=Mapk10 PE=1 SV=2	1	12	0.95	3.86E+08	4.76E+08	4.25E+08	5.95E+08	5.35E+08	3.75E+08	4.30E+08	4.02E+08	3.83E+08	4.12E+08	4.20E+08	5.09E+08	3.93E+08	3.81E-05	3.40E-06	3.87E-08	1.40E-04	1.31E-02	9.70E-08	1.03E-04	1.73E-04	2.74E-03	1.13E-06	9.77E-05	3.49E-04	2.45E-01	4	12	12	MK10_MOUSE, SP:Q61831	SP|Q61831|MK10_MOUSE
P09813	APOA2_MOUSE	Apolipoprotein A-II OS=Mus musculus GN=Apoa2 PE=1 SV=1	1	10	0.99	2.18E+08	1.09E+08	1.43E+08	1.47E+08	1.92E+08	1.52E+08	2.34E+08	9.84E+07	1.86E+08	1.76E+08	1.35E+08	1.99E+08	1.41E+08	3.68E-02	3.80E-01	6.29E-01	0.001	0.004	0.001	6.98E-02	1.85E-01	0.002	3.67E-01	6.12E-01	0.008	0.019	3	10	10	APOA2_MOUSE, SP:P09813	SP|P09813|APOA2_MOUSE
P54320	ELN_MOUSE	Elastin OS=Mus musculus GN=Eln PE=2 SV=1	1	1	0.99	1.51E+07	1.06E+07	9.51E+06	9.05E+06	1.35E+07	9.66E+06	8.62E+06	1.76E+07	1.61E+07	1.45E+07	8.01E+06	1.17E+07	1.30E+07	0.137	0.184	0.202	0.201	0.246	0.162	0.224	0.142	0.192	0.196	0.208	0.145	0.260	2	1	1	ELN_MOUSE, SP:P54320	SP|P54320|ELN_MOUSE
P82349	SGCB_MOUSE	Beta-sarcoglycan OS=Mus musculus GN=Sgcb PE=1 SV=1	1	2	1.0	3.20E+07	4.38E+07	6.27E+07	5.45E+07	5.88E+07	5.16E+07	4.65E+07	3.26E+07	4.21E+07	5.15E+07	4.98E+07	3.40E+07	4.56E+07	0.071	0.015	0.029	0.046	0.064	0.031	0.049	0.051	0.058	0.024	0.094	0.093	0.125	2	2	2	SGCB_MOUSE, SP:P82349	SP|P82349|SGCB_MOUSE
Q9R0X4	ACOT9_MOUSE	Acyl-coenzyme A thioesterase 9, mitochondrial OS=Mus musculus GN=Acot9 PE=1 SV=1	1	2	0.99	7.00E+07	6.99E+07	1.07E+08	6.22E+07	4.91E+07	6.67E+07	5.80E+07	7.09E+07	7.50E+07	8.29E+07	6.12E+07	9.85E+07	1.08E+08	0.010	0.001	0.015	0.057	0.037	0.006	0.026	0.011	0.019	0.011	0.010	0.017	0.033	2	2	2	ACOT9_MOUSE, SP:Q9R0X4	SP|Q9R0X4|ACOT9_MOUSE,SP|Q32MW3|ACO10_MOUSE
O88833	CP4AA_MOUSE	Cytochrome P450 4A10 OS=Mus musculus GN=Cyp4a10 PE=2 SV=1	1	12	0.64	2.38E+08	5.06E+08	4.94E+08	3.67E+08	6.79E+08	5.34E+08	7.13E+08	5.39E+08	5.21E+08	6.34E+08	3.18E+08	6.52E+08	3.30E+08	8.36E-03	9.00E-08	7.78E-05	2.26E-06	2.19E-04	1.06E-08	6.60E-09	9.07E-07	6.01E-06	4.99E-05	1.12E-06	3.72E-06	6.20E-01	5	12	12	CP4AA_MOUSE, SP:O88833	SP|O88833|CP4AA_MOUSE
Q811L6	MAST4_MOUSE	Microtubule-associated serine/threonine-protein kinase 4 OS=Mus musculus GN=Mast4 PE=1 SV=2	1	3	0.7	1.17E+09	7.38E+08	1.08E+09	1.12E+09	1.12E+09	1.57E+09	1.27E+09	9.36E+08	1.66E+09	9.04E+08	2.47E+09	1.07E+09	9.85E+08	2.10E-18	2.93E-18	6.42E-17	2.26E-11	4.71E-15	2.48E-13	8.60E-17	1.75E-23	4.81E-09	0	1.03E-11	1.33E-11	1.35E-05	8	3	3	MAST4_MOUSE, SP:Q811L6	SP|Q811L6|MAST4_MOUSE
Q9ERV1	MKRN2_MOUSE	Probable E3 ubiquitin-protein ligase makorin-2 OS=Mus musculus GN=Mkrn2 PE=2 SV=1	1	8	0.77	2.48E+09	1.66E+09	1.51E+09	2.11E+09	2.13E+09	2.09E+09	1.79E+09	2.29E+09	1.80E+09	2.02E+09	1.84E+09	2.20E+09	2.33E+09	3.25E-34	1.25E-23	3.23E-29	1.15E-20	4.10E-19	1.40E-27	5.93E-22	1.33E-23	9.89E-20	3.17E-30	6.80E-23	1.64E-26	6.28E-14	4	8	8	MKRN2_MOUSE, SP:Q9ERV1	SP|Q9ERV1|MKRN2_MOUSE
P97433	RGNEF_MOUSE	Rho-guanine nucleotide exchange factor OS=Mus musculus GN=Rgnef PE=1 SV=1	1	7	0.78	6.61E+08	3.44E+08	5.63E+08	5.38E+08	6.90E+08	6.50E+08	5.17E+08	4.07E+08	8.22E+08	4.45E+08	6.71E+08	4.99E+08	5.96E+08	7.55E-10	6.38E-09	1.52E-07	1.76E-06	1.40E-05	1.56E-05	3.77E-06	3.79E-11	8.84E-04	1.35E-11	8.25E-05	2.65E-04	7.74E-03	5	7	7	RGNEF_MOUSE, SP:P97433	SP|P97433|RGNEF_MOUSE
O89103	C1QR1_MOUSE	Complement component C1q receptor OS=Mus musculus GN=Cd93 PE=1 SV=1	1	13	1.0	5.60E+09	8.72E+09	8.42E+09	5.65E+09	5.40E+09	7.21E+09	5.17E+09	7.54E+09	5.62E+09	7.13E+09	9.41E+09	6.66E+09	5.22E+09	0	0	0	0	0	0	0	0	0	0	0	0	1.28E-31	5, 6	11, 13	11, 24	C1QR1_MOUSE, SP:O89103	SP|O89103|C1QR1_MOUSE
Q769J6	ATS13_MOUSE	A disintegrin and metalloproteinase with thrombospondin motifs 13 OS=Mus musculus GN=Adamts13 PE=2 SV=1	1	1	0.74	9.54E+07	2.52E+08	8.57E+08	1.48E+08	4.09E+07	6.42E+07	1.01E+08	8.54E+07	8.71E+07	1.11E+08	9.77E+07	3.44E+08	2.89E+08	0.003	4.04E-14	4.36E-01	0.078	0.042	1.20E-03	0.005	0.007	0.009	0.002	6.21E-03	0.011	0.001	5	1	1	ATS13_MOUSE, SP:Q769J6	SP|Q769J6|ATS13_MOUSE
Q8BZ05	ARAP2_MOUSE	Arf-GAP with Rho-GAP domain, ANK repeat and PH domain-containing protein 2 OS=Mus musculus GN=Arap2 PE=1 SV=2	1	11	0.81	1.04E+09	1.08E+09	1.05E+09	1.49E+09	1.66E+09	1.53E+09	1.30E+09	1.17E+09	1.26E+09	1.69E+09	1.08E+09	1.78E+09	2.54E+09	1.31E-14	6.60E-16	2.03E-20	1.91E-15	1.69E-13	7.91E-18	1.72E-15	3.20E-16	5.56E-16	7.63E-18	5.51E-18	1.55E-13	1.58E-14	2	11	11	ARAP2_MOUSE, SP:Q8BZ05	SP|Q8BZ05|ARAP2_MOUSE
A2ARA8	ITA8_MOUSE	Integrin alpha-8 OS=Mus musculus GN=Itga8 PE=1 SV=1	1	4	0.89	4.64E+08	6.17E+08	5.56E+08	4.13E+08	4.67E+08	4.60E+08	5.14E+08	4.97E+08	5.69E+08	4.96E+08	5.93E+08	3.73E+08	2.14E+08	1.08E-06	6.68E-09	1.37E-05	5.14E-04	1.34E-03	8.13E-11	3.82E-06	1.65E-07	2.12E-04	2.39E-10	2.65E-03	1.30E-05	0.005	4	4	4	ITA8_MOUSE, SP:A2ARA8	SP|A2ARA8|ITA8_MOUSE
P02463	CO4A1_MOUSE	Collagen alpha-1(IV) chain OS=Mus musculus GN=Col4a1 PE=2 SV=4	3	5	1.0	1.82E+08	2.14E+08	4.73E+08	2.26E+08	1.35E+08	1.50E+08	2.09E+08	1.58E+08	2.07E+08	2.08E+08	2.14E+08	3.40E+08	3.11E+08	1.34E-01	2.83E-07	2.55E-02	0.005	0.004	9.59E-03	1.49E-01	9.30E-02	5.91E-01	9.95E-03	7.98E-03	9.96E-01	8.90E-01	3, 4	1, 5	1, 5	CO4A1_MOUSE, SP:P02463	SP|P02463|CO4A1_MOUSE
Q3TQQ9	CA112_MOUSE	Uncharacterized protein C1orf112 homolog OS=Mus musculus PE=2 SV=2	1	11	0.82	2.85E+08	6.06E+08	3.06E+08	4.68E+08	5.45E+08	1.52E+08	2.00E+08	3.30E+08	5.65E+08	7.11E+08	3.05E+08	4.02E+08	5.40E+08	1.45E-03	2.67E-04	2.00E-06	7.08E-05	0.004	1.97E-10	1.57E-01	2.41E-07	9.23E-07	1.11E-04	1.27E-03	2.98E-03	1.94E-02	4	11	11	CA112_MOUSE, SP:Q3TQQ9	SP|Q3TQQ9|CA112_MOUSE
P12979	MYOG_MOUSE	Myogenin OS=Mus musculus GN=Myog PE=2 SV=2	1	9	1.0	3.78E+09	4.73E+09	4.28E+09	3.34E+09	4.27E+09	3.28E+09	3.12E+09	3.83E+09	3.21E+09	2.89E+09	3.93E+09	2.85E+09	2.35E+09	0	0	0	3.15E-38	7.80E-29	0	4.12E-36	3.07E-39	1.42E-27	0	5.69E-29	2.80E-41	4.45E-14	5	9	9	MYOG_MOUSE, SP:P12979	SP|P12979|MYOG_MOUSE
Q9ERP3	TRI54_MOUSE	Tripartite motif-containing protein 54 OS=Mus musculus GN=Trim54 PE=1 SV=1	1	3	0.99	9.63E+07	1.50E+08	1.10E+08	8.73E+07	1.38E+08	1.58E+08	1.17E+08	1.70E+08	1.27E+08	1.67E+08	1.13E+08	1.01E+08	1.14E+08	0.003	0.001	0.005	0.004	0.003	1.59E-01	0.003	0.002	0.002	8.89E-01	0.009	6.04E-01	0.029	3	3	3	TRI54_MOUSE, SP:Q9ERP3	SP|Q9ERP3|TRI54_MOUSE,SP|Q38HM4|TRI63_MOUSE
Q9CY94	PSF3_MOUSE	DNA replication complex GINS protein PSF3 OS=Mus musculus GN=Gins3 PE=2 SV=1	1	5	0.64	1.32E+08	1.69E+08	1.58E+08	1.93E+08	3.23E+08	1.19E+08	1.77E+08	9.95E+07	1.51E+08	2.45E+08	8.49E+07	1.65E+08	1.63E+08	7.38E-01	1.93E-01	7.34E-02	2.71E-02	0.009	7.30E-02	3.92E-01	6.29E-01	2.03E-01	0.004	0.001	0.007	0.012	4	5	5	PSF3_MOUSE, SP:Q9CY94	SP|Q9CY94|PSF3_MOUSE
Q9JIY0	PKHO1_MOUSE	Pleckstrin homology domain-containing family O member 1 OS=Mus musculus GN=Plekho1 PE=1 SV=1	1	7	0.96	6.22E+08	7.48E+08	6.37E+08	4.80E+08	2.84E+08	5.45E+08	5.34E+08	6.68E+08	5.03E+08	4.25E+08	5.80E+08	3.59E+08	4.04E+08	1.95E-09	1.75E-10	8.83E-07	6.35E-02	1.48E-04	1.62E-13	1.68E-06	1.34E-06	1.29E-03	2.96E-10	3.77E-03	5.17E-08	1.74E-01	7	7	7	PKHO1_MOUSE, SP:Q9JIY0	SP|Q9JIY0|PKHO1_MOUSE
Q9DCC8	TOM20_MOUSE	Mitochondrial import receptor subunit TOM20 homolog OS=Mus musculus GN=Tomm20 PE=1 SV=1	1	2	1.0	1.17E+08	1.24E+08	1.19E+08	1.30E+08	9.64E+07	1.05E+08	1.10E+08	1.34E+08	1.28E+08	9.44E+07	1.67E+08	1.28E+08	1.19E+08	0.001	8.29E-01	8.38E-01	0.013	0.012	3.91E-01	0.003	0.001	0.013	5.17E-02	0.004	0.002	0.026	5	2	2	TOM20_MOUSE, SP:Q9DCC8	SP|Q9DCC8|TOM20_MOUSE
Q80XJ3	TTC28_MOUSE	Tetratricopeptide repeat protein 28 OS=Mus musculus GN=Ttc28 PE=2 SV=2	1	4	0.79	9.01E+08	6.79E+08	7.00E+08	9.23E+08	6.70E+08	6.62E+08	7.91E+08	6.34E+08	7.18E+08	8.36E+08	7.45E+08	1.00E+09	7.83E+08	1.57E-13	2.94E-11	1.83E-13	3.13E-06	1.07E-05	7.79E-12	7.33E-10	1.29E-09	4.07E-08	5.69E-13	1.13E-10	2.11E-07	3.92E-04	5	4	4	TTC28_MOUSE, SP:Q80XJ3	SP|Q80XJ3|TTC28_MOUSE
Q3U276	SDHF1_MOUSE	Succinate dehydrogenase assembly factor 1, mitochondrial OS=Mus musculus GN=Sdhaf1 PE=2 SV=1	1	1	0.97	8.62E+06	8.61E+06	6.50E+06	8.37E+06	1.18E+07	8.37E+06	1.09E+07	8.71E+06	8.09E+06	1.17E+07	6.63E+06	1.32E+07	1.21E+07	0.204	0.229	0.212	0.217	0.261	0.187	0.197	0.223	0.216	0.218	0.194	0.228	0.267	2	1	1	SDHF1_MOUSE, SP:Q3U276	SP|Q3U276|SDHF1_MOUSE
Q6PAR0	KLD10_MOUSE	Kelch domain-containing protein 10 OS=Mus musculus GN=Klhdc10 PE=2 SV=1	1	2	0.92	2.32E+09	5.22E+08	1.22E+09	1.67E+08	1.76E+08	3.43E+08	3.07E+09	1.31E+09	1.00E+09	5.04E+08	6.34E+08	2.92E+08	2.57E+08	2.42E-35	2.29E-20	1.99E-01	0.001	2.43E-02	4.81E-09	3.60E-39	5.32E-14	1.71E-04	3.67E-11	2.83E-02	1.84E-16	0.002	7	2	2	KLD10_MOUSE, SP:Q6PAR0	SP|Q6PAR0|KLD10_MOUSE
Q8BIZ1	ANS1B_MOUSE	Ankyrin repeat and sterile alpha motif domain-containing protein 1B OS=Mus musculus GN=Anks1b PE=1 SV=2	1	13	0.99	1.47E+09	1.21E+09	1.05E+09	1.54E+09	1.39E+09	1.31E+09	1.48E+09	1.30E+09	1.14E+09	1.19E+09	1.06E+09	1.50E+09	8.96E+08	1.28E-20	3.90E-16	5.50E-21	3.24E-13	8.40E-12	4.60E-20	9.09E-18	6.80E-15	2.01E-11	1.06E-17	1.83E-15	2.79E-15	8.45E-05	4	13	13	ANS1B_MOUSE, SP:Q8BIZ1	SP|Q8BIZ1|ANS1B_MOUSE
Q8BTY1	KAT1_MOUSE	Kynurenine--oxoglutarate transaminase 1 OS=Mus musculus GN=Ccbl1 PE=2 SV=1	1	1	1.0	2.69E+07	2.75E+07	4.30E+07	3.85E+07	4.20E+07	4.92E+07	2.47E+07	3.84E+07	2.90E+07	4.00E+07	2.05E+07	3.57E+07	1.69E+07	0.074	0.028	0.044	0.071	0.061	0.058	0.100	0.076	0.075	0.086	0.078	0.059	0.231	2	1	1	KAT1_MOUSE, SP:Q8BTY1	SP|Q8BTY1|KAT1_MOUSE
Q80TF3	PCD19_MOUSE	Protocadherin-19 OS=Mus musculus GN=Pcdh19 PE=2 SV=2	1	5	1.0	1.19E+09	5.26E+08	9.31E+08	1.74E+09	1.55E+09	3.05E+08	5.04E+08	4.16E+08	1.06E+09	1.21E+09	4.71E+08	1.14E+09	8.11E+08	3.98E-18	3.06E-15	2.00E-25	1.62E-15	6.59E-02	4.94E-09	6.24E-06	1.31E-14	3.41E-12	5.86E-08	3.09E-12	1.92E-04	2.42E-04	4	5	5	PCD19_MOUSE, SP:Q80TF3	SP|Q80TF3|PCD19_MOUSE
Q9CQ85	TIM22_MOUSE	Mitochondrial import inner membrane translocase subunit Tim22 OS=Mus musculus GN=Timm22 PE=2 SV=1	1	1	0.91	4.93E+07	6.19E+07	5.83E+07	5.54E+07	5.88E+07	5.82E+07	5.04E+07	5.31E+07	6.10E+07	4.67E+07	4.45E+07	3.99E+07	4.76E+07	0.027	0.015	0.021	0.042	0.047	0.010	0.036	0.020	0.061	0.024	0.070	0.035	0.111	3	1	1	TIM22_MOUSE, SP:Q9CQ85	SP|Q9CQ85|TIM22_MOUSE
Q8CDI7	CC150_MOUSE	Coiled-coil domain-containing protein 150 OS=Mus musculus GN=Ccdc150 PE=2 SV=1	1	3	0.8	4.49E+08	4.34E+08	1.30E+08	2.01E+08	5.48E+08	1.23E+09	6.58E+08	6.74E+08	3.94E+08	5.20E+08	2.13E+08	2.48E+08	1.09E+08	1.68E-06	4.74E-01	4.60E-02	5.64E-05	1.05E-11	2.02E-07	2.87E-08	6.85E-05	1.02E-04	5.64E-03	9.60E-02	4.34E-08	0.031	7	3	3	CC150_MOUSE, SP:Q8CDI7	SP|Q8CDI7|CC150_MOUSE
Q9Z1N9	UN13B_MOUSE	Protein unc-13 homolog B OS=Mus musculus GN=Unc13b PE=2 SV=1	1	5	0.98	1.99E+07	3.95E+07	1.46E+08	2.40E+07	2.15E+07	2.11E+07	2.68E+07	1.76E+07	1.63E+07	8.17E+06	3.87E+07	3.97E+07	1.64E+07	0.185	4.42E-01	0.102	0.197	0.202	0.048	0.130	0.200	0.328	0.045	0.072	0.198	0.259	5	5	5	UN13B_MOUSE, SP:Q9Z1N9	SP|Q9Z1N9|UN13B_MOUSE
Q8BRH4	MLL3_MOUSE	Histone-lysine N-methyltransferase MLL3 OS=Mus musculus GN=Mll3 PE=2 SV=2	2	5	1.0	9.53E+08	1.64E+09	1.25E+09	9.03E+08	7.94E+08	1.45E+09	1.26E+09	1.11E+09	1.19E+09	1.38E+09	9.67E+08	8.96E+08	8.47E+08	3.72E-14	2.50E-19	4.46E-13	2.91E-07	4.57E-13	2.80E-27	1.02E-15	9.91E-16	1.78E-13	5.28E-16	3.54E-09	4.75E-13	1.65E-04	2, 4	3, 5	3, 5	MLL3_MOUSE, SP:Q8BRH4	SP|Q8BRH4|MLL3_MOUSE
E9Q557	DESP_MOUSE	Desmoplakin OS=Mus musculus GN=Dsp PE=3 SV=1	22	13	1.0	2.35E+09	3.42E+09	3.28E+09	2.52E+09	2.68E+09	3.00E+09	2.77E+09	3.45E+09	2.70E+09	2.54E+09	3.33E+09	2.97E+09	3.14E+09	2.30E-38	0	5.68E-40	8.50E-29	3.35E-30	0	5.00E-38	1.04E-39	1.93E-27	0	6.30E-35	0	1.25E-20	2, 2, 4, 2, 5, 3, 4, 5, 6	1, 10, 11, 12, 13, 2, 2, 8, 3, 3, 7, 4, 5, 6, 8, 9	1, 10, 11, 12, 13, 2, 2, 8, 3, 3, 7, 4, 5, 6, 8, 9	DESP_MOUSE, SP:E9Q557	SP|E9Q557|DESP_MOUSE
Q9DC07	LNEBL_MOUSE	LIM zinc-binding domain-containing Nebulette OS=Mus musculus GN=Nebl PE=2 SV=1	3	13	1.0	1.25E+09	1.34E+09	1.42E+09	1.25E+09	1.62E+09	1.31E+09	1.41E+09	1.27E+09	1.26E+09	1.32E+09	1.17E+09	1.20E+09	1.43E+09	1.08E-19	5.33E-24	6.75E-19	7.91E-17	1.40E-12	1.28E-24	9.60E-19	5.01E-18	1.04E-13	4.34E-21	2.21E-13	5.12E-16	1.24E-08	3, 4, 5	10, 13, 9	10, 13, 9	LNEBL_MOUSE, SP:Q9DC07	SP|Q9DC07|LNEBL_MOUSE
Q9QXS1	PLEC_MOUSE	Plectin OS=Mus musculus GN=Plec PE=1 SV=2	23	13	1.0	2.51E+09	2.63E+09	2.78E+09	2.58E+09	2.52E+09	2.63E+09	2.47E+09	2.57E+09	2.97E+09	2.84E+09	2.81E+09	2.95E+09	2.81E+09	2.52E-41	0	3.36E-41	2.96E-27	1.03E-26	0	2.47E-34	0	4.48E-31	0	7.58E-35	3.64E-34	2.07E-18	2, 2, 4, 3, 4, 5, 6	1, 11, 13, 2, 2, 7, 3, 7, 8, 9	1, 11, 13, 2, 2, 7, 3, 7, 8, 9	PLEC_MOUSE, SP:Q9QXS1	SP|Q9QXS1|PLEC_MOUSE
Q8R104	SIR3_MOUSE	NAD-dependent protein deacetylase sirtuin-3 OS=Mus musculus GN=Sirt3 PE=2 SV=1	1	6	1.0	8.22E+07	5.00E+07	3.39E+07	4.22E+07	2.71E+07	6.87E+07	6.14E+07	5.44E+07	7.31E+07	4.62E+07	7.31E+07	4.76E+07	3.36E+07	0.006	0.046	0.037	0.119	0.034	0.016	0.022	0.011	0.060	0.005	0.051	0.032	0.152	4	6	6	SIR3_MOUSE, SP:Q8R104	SP|Q8R104|SIR3_MOUSE
E9Q401	RYR2_MOUSE	Ryanodine receptor 2 OS=Mus musculus GN=Ryr2 PE=1 SV=1	26	13	1.0	2.75E+09	4.05E+09	3.37E+09	2.90E+09	2.69E+09	3.49E+09	2.82E+09	3.94E+09	2.99E+09	3.14E+09	3.32E+09	3.06E+09	2.83E+09	0	0	0	4.96E-29	1.68E-35	0	5.02E-39	0	2.73E-34	0	5.77E-36	0	1.65E-18	2, 3, 4, 5	10, 11, 13, 2, 3, 4, 5, 6, 7, 8, 9	10, 11, 13, 2, 3, 4, 5, 6, 7, 8, 9	RYR2_MOUSE, SP:E9Q401	SP|E9Q401|RYR2_MOUSE
Q8VCT4	CES1D_MOUSE	Carboxylesterase 1D OS=Mus musculus GN=Ces1d PE=1 SV=1	6	13	1.0	8.56E+08	8.15E+08	9.99E+08	9.62E+08	1.38E+09	1.20E+09	1.21E+09	7.55E+08	9.20E+08	8.87E+08	8.91E+08	9.13E+08	5.66E+08	3.12E-13	9.57E-17	1.76E-14	3.24E-14	1.79E-11	1.57E-14	4.80E-16	6.93E-13	9.58E-09	3.74E-16	7.72E-10	4.19E-09	1.29E-02	2, 3, 4, 5	1, 11, 12, 13, 3, 4, 5	1, 11, 12, 13, 3, 4, 5	CES1D_MOUSE, SP:Q8VCT4	SP|Q8VCT4|CES1D_MOUSE
Q8K2C6	SIR5_MOUSE	NAD-dependent protein deacylase sirtuin-5, mitochondrial OS=Mus musculus GN=Sirt5 PE=1 SV=1	1	12	0.99	1.70E+08	1.87E+08	1.63E+08	1.69E+08	1.96E+08	2.19E+08	2.01E+08	1.82E+08	1.69E+08	2.34E+08	1.69E+08	1.72E+08	2.05E+08	1.35E-01	1.06E-01	1.69E-01	7.30E-01	5.72E-01	1.93E-02	1.39E-01	2.74E-01	2.44E-01	4.82E-02	9.65E-01	3.54E-01	0.005	4	12	12	SIR5_MOUSE, SP:Q8K2C6	SP|Q8K2C6|SIR5_MOUSE
P11983	TCPA_MOUSE	T-complex protein 1 subunit alpha OS=Mus musculus GN=Tcp1 PE=1 SV=3	5	13	1.0	2.94E+08	4.51E+08	4.29E+08	3.99E+08	4.75E+08	5.21E+08	4.31E+08	5.35E+08	4.29E+08	4.96E+08	4.19E+08	3.73E+08	4.29E+08	1.04E-03	1.30E-06	2.35E-05	4.02E-04	2.89E-04	1.14E-07	5.83E-05	2.16E-05	2.02E-04	5.36E-07	2.73E-03	3.60E-06	1.16E-01	2, 3, 3, 4, 4	1, 1, 3, 13, 9	1, 1, 3, 13, 9	TCPA_MOUSE, SP:P11983	SP|P11983|TCPA_MOUSE
Q9ESB3	HRG_MOUSE	Histidine-rich glycoprotein OS=Mus musculus GN=Hrg PE=1 SV=2	1	8	0.99	1.74E+08	1.22E+08	1.00E+08	2.72E+08	1.40E+08	1.70E+08	3.99E+08	1.43E+08	1.64E+08	3.60E+08	1.01E+08	1.29E+08	1.03E+08	1.11E-01	0.002	2.86E-03	0.003	0.002	4.32E-01	1.71E-04	3.26E-01	7.80E-03	0.001	0.004	0.001	0.035	5	8	8	HRG_MOUSE, SP:Q9ESB3	SP|Q9ESB3|HRG_MOUSE
Q9DB60	PGFS_MOUSE	Prostamide/prostaglandin F synthase OS=Mus musculus GN=Fam213b PE=1 SV=1	3	8	1.0	2.04E+08	2.31E+08	2.21E+08	2.18E+08	1.37E+08	1.71E+08	2.51E+08	1.92E+08	3.11E+08	2.54E+08	4.44E+08	3.69E+08	3.00E+08	3.33E-02	8.47E-03	2.37E-02	0.004	0.002	2.35E-03	2.39E-02	1.38E-03	1.38E-01	1.25E-07	2.89E-03	2.45E-01	0.001	3, 5	1, 8	1, 8	PGFS_MOUSE, SP:Q9DB60	SP|Q9DB60|PGFS_MOUSE
Q0II04	NEBL_MOUSE	Nebulette OS=Mus musculus GN=Nebl PE=2 SV=1	7	13	1.0	3.71E+09	4.21E+09	3.90E+09	3.72E+09	3.82E+09	3.74E+09	3.73E+09	3.26E+09	3.56E+09	3.82E+09	3.81E+09	3.48E+09	3.52E+09	0	0	0	8.83E-41	1.96E-37	0	0	0	3.08E-41	0	3.53E-40	2.80E-42	6.05E-23	2, 3, 4, 5	1, 13, 6	1, 13, 6	NEBL_MOUSE, SP:Q0II04	SP|Q0II04|NEBL_MOUSE
P70404	IDHG1_MOUSE	Isocitrate dehydrogenase [NAD] subunit gamma 1, mitochondrial OS=Mus musculus GN=Idh3g PE=1 SV=1	11	13	1.0	9.61E+09	1.42E+10	1.39E+10	9.79E+09	8.37E+09	1.22E+10	1.07E+10	1.13E+10	9.58E+09	1.17E+10	1.15E+10	1.02E+10	9.10E+09	0	0	0	0	0	0	0	0	0	0	0	0	0	2, 3, 4, 4, 5, 5, 7, 8	1, 11, 13, 2, 3, 5, 6, 13, 8, 9	1, 11, 13, 13, 6, 19, 2, 3, 5, 8, 9	IDHG1_MOUSE, SP:P70404	SP|P70404|IDHG1_MOUSE
P0CG49	UBC_MOUSE	Polyubiquitin-B OS=Mus musculus GN=Ubb PE=1 SV=1	3	13	1.0	4.55E+09	4.56E+09	4.71E+09	5.12E+09	4.11E+09	3.68E+09	4.55E+09	4.25E+09	4.66E+09	4.58E+09	4.62E+09	5.00E+09	4.76E+09	0	0	0	0	6.84E-36	0	0	0	0	0	0	0	1.60E-30	2, 4, 3, 6	12, 13, 4, 12	12, 12, 4, 13	UBC_MOUSE, SP:P0CG50	SP|P0CG50|UBC_MOUSE,SP|P0CG49|UBB_MOUSE,SP|P62983|RS27A_MOUSE,SP|P62984|RL40_MOUSE
Q9CRB8	MTFP1_MOUSE	Mitochondrial fission process protein 1 OS=Mus musculus GN=Mtfp1 PE=1 SV=1	3	13	1.0	1.20E+09	1.51E+09	1.47E+09	1.32E+09	1.54E+09	1.52E+09	1.65E+09	1.38E+09	1.33E+09	1.39E+09	1.36E+09	1.50E+09	1.27E+09	3.69E-18	1.65E-24	1.73E-19	1.55E-15	2.87E-14	2.99E-27	1.94E-21	2.32E-18	4.18E-14	5.42E-24	3.20E-16	3.46E-17	4.40E-07	2, 5	12, 13, 3	12, 13, 4	MTFP1_MOUSE, SP:Q9CRB8	SP|Q9CRB8|MTFP1_MOUSE
P42125	ECI1_MOUSE	Enoyl-CoA delta isomerase 1, mitochondrial OS=Mus musculus GN=Eci1 PE=2 SV=1	11	13	1.0	2.42E+10	2.64E+10	2.60E+10	2.96E+10	2.46E+10	2.49E+10	2.81E+10	2.17E+10	2.19E+10	2.48E+10	2.38E+10	2.58E+10	2.13E+10	0	0	0	0	0	0	0	0	0	0	0	0	0	2, 3, 4, 4, 5, 5, 6	10, 11, 12, 13, 2, 5, 6, 11, 9	10, 11, 11, 6, 12, 13, 14, 2	ECI1_MOUSE, SP:P42125	SP|P42125|ECI1_MOUSE
Q8BLF1	NCEH1_MOUSE	Neutral cholesterol ester hydrolase 1 OS=Mus musculus GN=Nceh1 PE=1 SV=1	2	8	1.0	2.64E+08	4.79E+08	3.29E+08	3.04E+08	3.24E+08	4.32E+08	3.68E+08	3.75E+08	3.73E+08	3.21E+08	3.94E+08	3.27E+08	3.02E+08	2.87E-03	8.57E-05	8.39E-04	2.24E-02	2.59E-03	2.97E-08	4.56E-04	1.59E-04	2.23E-02	1.51E-06	1.00E-02	6.21E-04	9.96E-01	3, 5	1, 8	1, 8	NCEH1_MOUSE, SP:Q8BLF1	SP|Q8BLF1|NCEH1_MOUSE
Q6QR59	F115E_MOUSE	Protein FAM115E OS=Mus musculus GN=Fam115e PE=2 SV=1	1	13	1.0	9.43E+08	1.16E+09	1.13E+09	8.32E+08	7.50E+08	8.55E+08	8.44E+08	1.02E+09	8.23E+08	6.96E+08	1.03E+09	5.08E+08	5.61E+08	1.40E-14	6.62E-19	2.10E-12	3.25E-07	8.85E-08	6.84E-21	8.37E-11	2.33E-11	1.08E-06	1.60E-18	5.53E-05	1.42E-12	1.30E-02	6	12	13	F115E_MOUSE, SP:Q6QR59	SP|Q6QR59|F115E_MOUSE
Q8C0D0	TRUB1_MOUSE	Probable tRNA pseudouridine synthase 1 OS=Mus musculus GN=Trub1 PE=2 SV=1	1	8	0.99	4.87E+08	4.00E+08	4.59E+08	4.43E+08	4.04E+08	4.02E+08	3.34E+08	4.52E+08	4.31E+08	4.07E+08	3.53E+08	5.30E+08	5.21E+08	1.09E-06	8.85E-07	1.00E-05	6.04E-03	1.27E-02	2.68E-06	3.26E-03	4.55E-05	6.17E-03	2.02E-05	8.19E-05	1.24E-04	7.46E-02	4	8	8	TRUB1_MOUSE, SP:Q8C0D0	SP|Q8C0D0|TRUB1_MOUSE
Q9WUR2	ECI2_MOUSE	Enoyl-CoA delta isomerase 2, mitochondrial OS=Mus musculus GN=Eci2 PE=1 SV=2	7	13	1.0	2.29E+09	2.57E+09	2.45E+09	2.26E+09	2.56E+09	2.74E+09	2.34E+09	2.41E+09	2.09E+09	2.34E+09	2.09E+09	2.11E+09	1.87E+09	5.28E-37	2.80E-42	3.01E-35	4.29E-27	3.18E-27	0	1.31E-31	2.86E-30	5.66E-25	1.17E-38	4.09E-24	2.92E-31	1.04E-11	2, 3, 4, 4, 6, 6	1, 12, 13, 2, 9, 13	1, 12, 13, 13, 9, 2	ECI2_MOUSE, SP:Q9WUR2	SP|Q9WUR2|ECI2_MOUSE
A2A5N8	LMBL1_MOUSE	Lethal(3)malignant brain tumor-like protein 1 OS=Mus musculus GN=L3mbtl1 PE=2 SV=1	1	12	0.98	6.31E+08	4.92E+08	5.80E+08	7.25E+08	7.65E+08	6.20E+08	6.23E+08	6.45E+08	6.87E+08	6.99E+08	6.88E+08	5.25E+08	6.67E+08	3.76E-09	3.78E-09	1.93E-10	3.36E-07	3.57E-05	2.46E-08	1.56E-07	3.76E-09	1.55E-06	5.49E-12	6.47E-05	1.64E-07	3.28E-03	5	12	12	LMBL1_MOUSE, SP:A2A5N8	SP|A2A5N8|LMBL1_MOUSE
P00761	TRYP_PIG	Trypsin OS=Sus scrofa PE=1 SV=1	5	13	1.0	2.11E+11	2.09E+11	1.98E+11	1.91E+11	2.05E+11	1.94E+11	1.99E+11	1.91E+11	1.70E+11	2.38E+11	1.81E+11	2.08E+11	2.02E+11	0	0	0	0	0	0	0	0	0	0	0	0	0	2, 7, 3, 7, 8, 6, 6, 8	1, 13, 12, 13, 6	13, 23, 13, 26, 25, 26, 6	TRYP_PIG, SP:P00761	SP|P00761|TRYP_PIG
Q6RUT8	CC154_MOUSE	Coiled-coil domain-containing protein 154 OS=Mus musculus GN=Ccdc154 PE=2 SV=2	1	6	0.74	5.80E+09	4.20E+09	6.28E+09	5.21E+09	5.70E+09	6.38E+09	4.91E+09	4.96E+09	6.13E+09	7.12E+09	6.57E+09	7.79E+09	8.24E+09	0	0	0	0	0	0	0	0	0	0	0	0	0	6	6	6	CC154_MOUSE, SP:Q6RUT8	SP|Q6RUT8|CC154_MOUSE
Q9QUK3	CLN8_MOUSE	Protein CLN8 OS=Mus musculus GN=Cln8 PE=2 SV=1	1	9	0.97	3.43E+09	2.53E+09	1.43E+09	3.60E+09	4.06E+09	2.36E+09	2.74E+09	2.81E+09	3.89E+09	3.88E+09	2.26E+09	2.60E+09	2.24E+09	0	8.93E-24	0	2.47E-40	1.21E-22	0	4.12E-35	0	6.61E-39	3.61E-39	1.53E-28	1.72E-34	6.13E-14	8	9	9	CLN8_MOUSE, SP:Q9QUK3	SP|Q9QUK3|CLN8_MOUSE
Q8BGY7	F210A_MOUSE	Protein FAM210A OS=Mus musculus GN=Fam210a PE=2 SV=1	1	7	0.99	6.04E+07	8.59E+07	6.16E+07	8.05E+07	5.23E+07	3.77E+07	5.98E+07	9.63E+07	4.66E+07	4.10E+07	6.22E+07	4.58E+07	2.73E+07	0.014	0.011	0.007	0.049	0.088	0.003	0.023	0.034	0.071	0.009	0.054	0.007	0.176	4	7	7	F210A_MOUSE, SP:Q8BGY7	SP|Q8BGY7|F210A_MOUSE
Q3UIJ9	MYZAP_MOUSE	Myocardial zonula adherens protein OS=Mus musculus GN=Myzap PE=1 SV=1	1	1	0.96	3.08E+07	4.32E+07	2.40E+07	3.37E+07	3.23E+07	3.32E+07	3.65E+07	2.94E+07	2.55E+07	3.22E+07	3.16E+07	3.37E+07	3.28E+07	0.060	0.077	0.054	0.098	0.102	0.023	0.060	0.089	0.097	0.045	0.084	0.086	0.153	2	1	1	MYZAP_MOUSE, SP:Q3UIJ9	SP|Q3UIJ9|MYZAP_MOUSE
P07361	A1AG2_MOUSE	Alpha-1-acid glycoprotein 2 OS=Mus musculus GN=Orm2 PE=1 SV=1	1	5	0.99	1.44E+07	2.12E+08	1.55E+07	4.56E+07	5.87E+06	6.65E+07	3.82E+07	9.69E+07	2.49E+06	1.08E+08	3.42E+07	7.01E+06	2.61E+07	0.143	0.127	0.031	0.292	0.036	6.41E-03	0.056	0.342	0.009	0.039	0.266	0.007	0.181	3	5	5	A1AG2_MOUSE, SP:P07361	SP|P07361|A1AG2_MOUSE
Q9CZ42	NNRD_MOUSE	ATP-dependent (S)-NAD(P)H-hydrate dehydratase OS=Mus musculus GN=Carkd PE=1 SV=1	2	12	1.0	3.14E+08	4.69E+08	3.91E+08	3.46E+08	3.58E+08	4.35E+08	4.18E+08	3.74E+08	3.75E+08	3.35E+08	3.61E+08	3.55E+08	3.59E+08	3.66E-04	5.36E-06	1.62E-04	9.08E-03	2.43E-03	4.10E-08	8.29E-05	1.37E-04	1.54E-02	6.12E-06	4.28E-03	6.33E-04	3.87E-01	3, 6	12, 5	12, 5	NNRD_MOUSE, SP:Q9CZ42	SP|Q9CZ42|NNRD_MOUSE
Q61644	PACN1_MOUSE	Protein kinase C and casein kinase substrate in neurons protein 1 OS=Mus musculus GN=Pacsin1 PE=1 SV=1	1	8	0.82	1.03E+09	1.08E+09	1.08E+09	2.15E+09	1.45E+09	9.77E+08	1.11E+09	9.11E+08	1.06E+09	1.10E+09	1.19E+09	1.57E+09	2.14E+09	1.72E-15	1.94E-17	6.76E-31	1.91E-14	6.52E-09	6.72E-19	4.46E-14	1.54E-14	7.12E-11	9.81E-21	6.09E-17	5.91E-11	4.35E-13	7	8	8	PACN1_MOUSE, SP:Q61644	SP|Q61644|PACN1_MOUSE
P00848	ATP6_MOUSE	ATP synthase subunit a OS=Mus musculus GN=Mtatp6 PE=3 SV=1	1	13	1.0	6.15E+08	6.03E+08	4.24E+08	6.32E+08	4.61E+08	3.14E+08	3.98E+08	4.44E+08	3.40E+08	5.02E+08	4.38E+08	5.50E+08	3.85E+08	4.94E-09	1.82E-06	5.31E-09	6.43E-04	5.07E-02	2.07E-10	1.95E-04	5.49E-04	2.01E-04	2.71E-07	2.11E-05	7.92E-05	2.52E-01	5	13	13	ATP6_MOUSE, SP:P00848	SP|P00848|ATP6_MOUSE
Q8R127	SCPDL_MOUSE	Saccharopine dehydrogenase-like oxidoreductase OS=Mus musculus GN=Sccpdh PE=2 SV=1	1	4	0.97	2.63E+08	3.47E+08	3.39E+08	2.64E+08	3.27E+08	3.58E+08	3.04E+08	3.54E+08	3.21E+08	2.63E+08	2.15E+08	3.81E+08	2.38E+08	4.90E-03	1.16E-04	6.07E-03	3.00E-02	2.21E-02	3.21E-05	6.73E-03	1.94E-03	1.51E-01	1.37E-02	3.12E-03	2.12E-03	0.003	4	4	4	SCPDL_MOUSE, SP:Q8R127	SP|Q8R127|SCPDL_MOUSE
Q8CDR2	RSH6A_MOUSE	Radial spoke head protein 6 homolog A OS=Mus musculus GN=Rsph6a PE=2 SV=1	1	5	0.92	6.23E+08	5.11E+08	4.68E+08	5.32E+08	6.19E+08	7.41E+08	5.06E+08	7.29E+08	4.34E+08	6.04E+08	4.90E+08	5.43E+08	8.29E+08	2.70E-09	2.54E-07	1.54E-07	1.02E-05	1.52E-06	8.06E-09	4.95E-06	1.85E-05	1.29E-05	2.16E-08	2.21E-05	1.04E-08	1.78E-04	6	5	5	RSH6A_MOUSE, SP:Q8CDR2	SP|Q8CDR2|RSH6A_MOUSE
D3YZF7	VS10L_MOUSE	V-set and immunoglobulin domain-containing protein 10-like OS=Mus musculus GN=Vsig10l PE=4 SV=1	1	9	0.88	1.33E+08	3.74E+08	2.07E+08	1.19E+08	1.45E+08	2.26E+08	1.66E+08	2.39E+08	1.48E+08	2.26E+08	1.59E+08	1.78E+08	1.24E+08	6.18E-01	1.66E-02	0.001	0.003	4.83E-01	4.44E-06	4.77E-01	6.23E-01	3.11E-01	8.09E-02	8.07E-01	5.56E-02	0.024	3	9	9	VS10L_MOUSE, SP:D3YZF7	SP|D3YZF7|VS10L_MOUSE
Q9D7J4	COX20_MOUSE	Cytochrome c oxidase protein 20 homolog OS=Mus musculus GN=Cox20 PE=2 SV=1	1	4	0.99	1.88E+08	2.52E+08	1.63E+08	2.34E+08	1.17E+08	2.00E+08	2.26E+08	2.31E+08	2.08E+08	2.08E+08	2.38E+08	1.52E+08	1.07E+08	1.59E-01	2.98E-01	3.15E-02	0.012	0.001	3.21E-03	1.25E-01	1.55E-01	8.98E-01	9.32E-03	0.003	1.31E-01	0.040	5	4	4	COX20_MOUSE, SP:Q9D7J4	SP|Q9D7J4|COX20_MOUSE
P24549	AL1A7_MOUSE	Retinal dehydrogenase 1 OS=Mus musculus GN=Aldh1a1 PE=1 SV=5	1	1	0.99	1.10E+07	2.72E+07	2.37E+07	1.66E+07	1.94E+07	3.22E+07	2.87E+07	3.32E+07	3.71E+07	2.23E+07	2.91E+07	1.92E+07	3.10E+07	0.176	0.078	0.130	0.158	0.105	0.057	0.084	0.052	0.140	0.052	0.149	0.073	0.160	2	1	1	AL1A7_MOUSE, SP:O35945	SP|O35945|AL1A7_MOUSE,SP|P24549|AL1A1_MOUSE
Q0VF22	CC138_MOUSE	Coiled-coil domain-containing protein 138 OS=Mus musculus GN=Ccdc138 PE=2 SV=1	1	1	0.88	1.81E+08	2.35E+08	3.91E+08	1.39E+08	2.37E+08	3.28E+08	2.60E+08	3.84E+08	2.31E+08	1.83E+08	1.53E+08	1.22E+08	1.44E+08	8.42E-02	0.036	5.71E-01	2.38E-01	3.43E-02	1.99E-03	1.77E-02	2.67E-02	0.001	9.96E-02	0.004	4.51E-04	0.016	7	1	1	CC138_MOUSE, SP:Q0VF22	SP|Q0VF22|CC138_MOUSE
Q62147	SSPN_MOUSE	Sarcospan OS=Mus musculus GN=Sspn PE=2 SV=1	1	13	0.99	1.51E+07	1.12E+07	1.25E+07	1.36E+07	1.80E+07	1.35E+07	1.55E+07	1.54E+07	8.72E+06	1.30E+07	7.24E+06	1.80E+07	2.45E+07	0.138	0.152	0.154	0.167	0.209	0.156	0.155	0.215	0.205	0.208	0.158	0.162	0.189	2	13	13	SSPN_MOUSE, SP:Q62147	SP|Q62147|SSPN_MOUSE
Q8CAK1	CAF17_MOUSE	Putative transferase CAF17 homolog, mitochondrial OS=Mus musculus GN=Iba57 PE=2 SV=1	1	1	1.0	3.02E+07	4.50E+07	4.90E+07	4.73E+07	7.68E+07	6.12E+07	6.21E+07	4.01E+07	5.32E+07	5.56E+07	4.09E+07	5.48E+07	5.17E+07	0.062	0.021	0.029	0.023	0.043	0.022	0.021	0.026	0.044	0.027	0.040	0.056	0.100	4	1	1	CAF17_MOUSE, SP:Q8CAK1	SP|Q8CAK1|CAF17_MOUSE
P42925	PXMP2_MOUSE	Peroxisomal membrane protein 2 OS=Mus musculus GN=Pxmp2 PE=2 SV=2	1	12	0.99	1.42E+08	1.62E+08	1.46E+08	1.64E+08	1.36E+08	1.48E+08	1.35E+08	1.81E+08	1.14E+08	1.31E+08	1.32E+08	1.39E+08	1.26E+08	4.36E-01	2.42E-01	2.22E-01	0.004	0.004	6.76E-02	0.001	0.002	0.005	3.02E-01	0.003	3.84E-01	0.023	4	12	12	PXMP2_MOUSE, SP:P42925	SP|P42925|PXMP2_MOUSE
Q5FW52	MLIP_MOUSE	Muscular LMNA-interacting protein OS=Mus musculus GN=Mlip PE=1 SV=1	1	1	0.99	8.38E+07	6.34E+07	7.54E+07	6.82E+07	6.93E+07	5.50E+07	7.01E+07	5.88E+07	6.62E+07	6.28E+07	6.34E+07	7.09E+07	6.09E+07	0.005	0.006	0.011	0.029	0.051	0.008	0.016	0.015	0.035	0.008	0.023	0.027	0.082	2	1	1	MLIP_MOUSE, SP:Q5FW52	SP|Q5FW52|MLIP_MOUSE
P59808	SASH1_MOUSE	SAM and SH3 domain-containing protein 1 OS=Mus musculus GN=Sash1 PE=1 SV=1	1	8	1.0	1.52E+08	1.37E+08	1.52E+08	1.41E+08	1.43E+08	1.19E+08	2.02E+08	1.75E+08	1.08E+08	1.49E+08	8.78E+07	2.00E+08	1.96E+08	2.80E-01	1.78E-01	5.27E-01	0.003	0.008	2.07E-01	1.33E-01	0.003	0.003	0.002	4.17E-01	4.48E-01	0.006	4	8	8	SASH1_MOUSE, SP:P59808	SP|P59808|SASH1_MOUSE
Q7TN75	PEG10_MOUSE	Retrotransposon-derived protein PEG10 OS=Mus musculus GN=Peg10 PE=1 SV=2	1	2	0.97	1.13E+08	9.68E+07	1.49E+08	1.05E+08	1.60E+08	2.40E+08	1.79E+08	1.38E+08	2.11E+08	1.17E+08	2.97E+08	1.32E+08	1.20E+08	0.002	2.35E-01	0.003	0.002	3.42E-01	0.002	3.28E-01	6.21E-02	0.007	1.36E-04	0.003	0.002	0.026	4	2	2	PEG10_MOUSE, SP:Q7TN75	SP|Q7TN75|PEG10_MOUSE
Q60590	A1AG1_MOUSE	Alpha-1-acid glycoprotein 1 OS=Mus musculus GN=Orm1 PE=1 SV=1	1	1	0.99	3.45E+07	2.96E+08	5.82E+07	8.69E+07	5.00E+07	1.24E+08	9.05E+07	1.66E+08	5.31E+07	1.59E+08	7.78E+07	4.62E+07	5.03E+07	0.049	0.013	0.005	0.053	0.007	1.31E-04	0.007	0.026	0.002	0.004	0.053	6.01E-01	0.103	3	1	1	A1AG1_MOUSE, SP:Q60590	SP|Q60590|A1AG1_MOUSE
Q9WVQ5	MTNB_MOUSE	Probable methylthioribulose-1-phosphate dehydratase OS=Mus musculus GN=Apip PE=1 SV=1	1	1	0.92	4.84E+07	1.04E+08	6.31E+07	5.14E+07	5.93E+07	9.66E+07	7.05E+07	7.46E+07	5.96E+07	7.00E+07	8.44E+07	5.64E+07	5.26E+07	0.026	0.011	0.024	0.040	0.015	0.001	0.015	0.020	0.028	0.003	0.038	0.015	0.098	2	1	1	MTNB_MOUSE, SP:Q9WVQ5	SP|Q9WVQ5|MTNB_MOUSE
Q8BUY5	TIDC1_MOUSE	Translocase of inner mitochondrial membrane domain-containing protein 1 OS=Mus musculus GN=Timmdc1 PE=2 SV=1	1	12	0.99	1.51E+08	1.09E+08	1.15E+08	1.30E+08	6.43E+07	1.01E+08	1.06E+08	8.54E+07	8.78E+07	1.16E+08	8.91E+07	1.33E+08	1.06E+08	2.93E-01	9.10E-01	8.42E-01	0.034	0.013	8.34E-01	0.004	0.006	0.007	0.002	0.003	0.010	0.033	4	12	12	TIDC1_MOUSE, SP:Q8BUY5	SP|Q8BUY5|TIDC1_MOUSE
Q9WV02	RBMX_MOUSE	RNA-binding motif protein, X chromosome OS=Mus musculus GN=Rbmx PE=1 SV=1	1	2	0.99	1.72E+07	3.14E+07	3.23E+07	3.21E+07	4.91E+07	3.79E+07	3.80E+07	4.45E+07	2.50E+07	4.71E+07	1.25E+07	4.41E+07	4.03E+07	0.156	0.054	0.063	0.055	0.087	0.050	0.058	0.096	0.060	0.171	0.060	0.046	0.129	3	2	2	RBMX_MOUSE, SP:Q9WV02	SP|Q9WV02|RBMX_MOUSE,SP|Q91VM5|RMXL1_MOUSE
Q8BMG7	RBGPR_MOUSE	Rab3 GTPase-activating protein non-catalytic subunit OS=Mus musculus GN=Rab3gap2 PE=1 SV=2	1	5	0.99	1.10E+08	1.34E+08	1.19E+08	9.86E+07	6.65E+07	1.12E+08	1.03E+08	1.22E+08	1.04E+08	9.67E+07	1.03E+08	7.01E+07	1.01E+08	0.002	7.59E-01	0.003	0.031	0.010	2.36E-01	0.005	0.003	0.012	0.001	0.024	0.003	0.036	3	5	5	RBGPR_MOUSE, SP:Q8BMG7	SP|Q8BMG7|RBGPR_MOUSE
P05366	SAA1_MOUSE	Serum amyloid A-1 protein OS=Mus musculus GN=Saa1 PE=2 SV=2	1	3	0.97	6.65E+06	1.02E+08	8.35E+06	2.12E+07	1.99E+07	1.50E+07	4.54E+06	6.45E+07	1.06E+07	5.20E+07	9.42E+06	1.98E+07	9.70E+06	0.265	0.229	0.110	0.163	0.208	0.001	0.318	0.209	0.052	0.200	0.154	0.023	0.295	2	3	3	SAA1_MOUSE, SP:P05366	SP|P05366|SAA1_MOUSE
Q6URW6	MYH14_MOUSE	Myosin-14 OS=Mus musculus GN=Myh14 PE=1 SV=1	1	1	1.0	2.45E+07	1.82E+07	2.92E+07	2.50E+07	2.22E+07	1.46E+07	1.50E+07	2.94E+07	2.36E+07	1.87E+07	1.46E+07	2.57E+07	2.60E+07	0.082	0.058	0.083	0.141	0.200	0.098	0.159	0.097	0.161	0.124	0.114	0.086	0.182	2	1	1	MYH14_MOUSE, SP:Q6URW6	SP|Q6URW6|MYH14_MOUSE
Q99LF4	RTCB_MOUSE	tRNA-splicing ligase RtcB homolog OS=Mus musculus GN=D10Wsu52e PE=2 SV=1	2	1	1.0	1.64E+08	1.29E+08	1.15E+08	1.18E+08	1.51E+08	1.22E+08	1.31E+08	1.24E+08	1.36E+08	1.31E+08	1.07E+08	1.35E+08	1.14E+08	6.29E-01	0.002	0.002	0.004	0.009	6.25E-01	0.003	0.001	0.007	0.002	0.005	0.004	0.033	2, 3	1	1	RTCB_MOUSE, SP:Q99LF4	SP|Q99LF4|RTCB_MOUSE
Q6ZWR6	SYNE1_MOUSE	Nesprin-1 OS=Mus musculus GN=Syne1 PE=1 SV=2	1	7	0.98	5.61E+07	5.54E+07	6.28E+07	7.83E+07	5.78E+07	1.03E+08	5.56E+07	6.92E+07	6.34E+07	8.50E+07	3.30E+07	1.06E+08	1.50E+08	0.018	0.011	0.007	0.042	0.013	0.012	0.027	0.017	0.018	0.041	0.007	0.018	0.015	3	7	7	SYNE1_MOUSE, SP:Q6ZWR6	SP|Q6ZWR6|SYNE1_MOUSE
Q8BYL4	SYYM_MOUSE	Tyrosine--tRNA ligase, mitochondrial OS=Mus musculus GN=Yars2 PE=2 SV=2	1	1	0.99	3.68E+07	5.90E+07	1.71E+07	3.13E+07	5.58E+07	3.16E+07	5.20E+07	4.82E+07	5.32E+07	3.67E+07	5.10E+07	5.35E+07	4.39E+07	0.045	0.118	0.062	0.045	0.108	0.010	0.032	0.026	0.083	0.016	0.042	0.041	0.119	2	1	1	SYYM_MOUSE, SP:Q8BYL4	SP|Q8BYL4|SYYM_MOUSE

Q8K4Z3	NNRE_MOUSE	NAD(P)H-hydrate epimerase OS=Mus musculus GN=Apoa1bp PE=1 SV=1	1	3	1.0	1.10E+08	1.36E+08	9.67E+07	1.18E+08	5.00E+07	7.61E+07	1.32E+08	8.90E+07	1.19E+08	8.64E+07	2.19E+08	9.18E+07	2.55E+07	0.002	0.002	0.001	0.054	0.027	2.12E-01	0.002	0.002	0.017	4.34E-03	0.012	0.009	0.184	5	3	3	NNRE_MOUSE, SP:Q8K4Z3	SP|Q8K4Z3|NNRE_MOUSE
P15105	GLNA_MOUSE	Glutamine synthetase OS=Mus musculus GN=Glul PE=1 SV=6	1	5	0.8	2.80E+07	5.65E+07	3.19E+07	3.70E+07	3.78E+06	1.70E+07	3.71E+07	2.60E+07	4.52E+07	4.63E+07	4.62E+07	4.65E+07	4.44E+07	0.069	0.050	0.046	0.329	0.182	0.012	0.058	0.036	0.060	0.020	0.053	0.099	0.117	2	5	5	GLNA_MOUSE, SP:P15105	SP|P15105|GLNA_MOUSE
Q8VEH8	ERLEC_MOUSE	Endoplasmic reticulum lectin 1 OS=Mus musculus GN=Erlec1 PE=2 SV=1	1	8	0.97	4.56E+07	1.63E+07	3.55E+07	3.71E+07	4.00E+07	2.47E+07	3.37E+07	1.73E+07	3.85E+07	2.54E+07	2.57E+07	3.37E+07	3.94E+07	0.029	0.042	0.046	0.075	0.136	0.111	0.068	0.049	0.124	0.063	0.084	0.147	0.132	2	8	8	ERLEC_MOUSE, SP:Q8VEH8	SP|Q8VEH8|ERLEC_MOUSE
P56383	AT5G2_MOUSE	ATP synthase lipid-binding protein, mitochondrial OS=Mus musculus GN=Atp5g2 PE=2 SV=2	1	6	0.99	5.25E+07	4.09E+07	1.36E+07	4.00E+07	4.18E+07	3.51E+07	3.36E+07	4.05E+07	4.33E+07	2.63E+07	3.48E+07	2.00E+07	2.38E+07	0.025	0.159	0.048	0.074	0.105	0.032	0.077	0.048	0.130	0.046	0.158	0.062	0.199	3, 4	1, 2	6	AT5G2_MOUSE, SP:P56383	SP|P56383|AT5G2_MOUSE,SP|P56384|AT5G3_MOUSE,SP|Q9CR84|AT5G1_MOUSE
Q9JIQ3	DBLOH_MOUSE	Diablo homolog, mitochondrial OS=Mus musculus GN=Diablo PE=1 SV=1	1	1	0.99	5.24E+07	7.18E+07	4.97E+07	5.82E+07	4.76E+07	3.67E+07	4.66E+07	6.19E+07	5.08E+07	4.74E+07	8.12E+07	5.49E+07	4.04E+07	0.021	0.020	0.018	0.058	0.091	0.005	0.039	0.029	0.058	0.003	0.039	0.024	0.129	5	1	1	DBLOH_MOUSE, SP:Q9JIQ3	SP|Q9JIQ3|DBLOH_MOUSE
Q8C165	P20D1_MOUSE	Probable carboxypeptidase PM20D1 OS=Mus musculus GN=Pm20d1 PE=2 SV=1	1	1	1.0	9.56E+06	8.39E+06	4.81E+06	1.20E+07	1.27E+07	1.00E+07	1.25E+07	4.04E+06	6.26E+06	1.23E+07	6.92E+06	9.43E+06	2.59E+06	0.193	0.264	0.169	0.209	0.242	0.190	0.181	0.252	0.211	0.214	0.234	0.306	0.387	2	1	1	P20D1_MOUSE, SP:Q8C165	SP|Q8C165|P20D1_MOUSE
Q5SW28	PI3R5_MOUSE	Phosphoinositide 3-kinase regulatory subunit 5 OS=Mus musculus GN=Pik3r5 PE=2 SV=1	1	1	0.67	1.31E+07	2.28E+07	1.28E+07	1.13E+07	2.24E+07	2.63E+07	2.53E+07	3.89E+08	1.25E+07	1.42E+07	3.14E+07	1.55E+07	1.93E+07	0.189	0.215	0.214	0.190	0.161	0.128	0.139	0.229	0.242	0.076	0.199	5.57E-04	0.224	3	1	1	PI3R5_MOUSE, SP:Q5SW28	SP|Q5SW28|PI3R5_MOUSE
Q3U962	CO5A2_MOUSE	Collagen alpha-2(V) chain OS=Mus musculus GN=Col5a2 PE=1 SV=1	1	1	0.65	5.69E+07	4.66E+07	5.10E+07	4.14E+07	3.66E+07	2.25E+07	1.31E+07	5.78E+07	3.62E+07	3.81E+07	5.59E+07	2.64E+07	1.22E+07	0.017	0.019	0.037	0.084	0.148	0.019	0.175	0.054	0.079	0.012	0.111	0.028	0.266	3	1	1	CO5A2_MOUSE, SP:Q3U962	SP|Q3U962|CO5A2_MOUSE
A2AX52	CO6A4_MOUSE	Collagen alpha-4(VI) chain OS=Mus musculus GN=Col6a4 PE=1 SV=2	1	2	0.9	1.91E+07	5.47E+07	6.43E+07	2.68E+07	4.12E+07	4.38E+07	5.71E+07	3.73E+07	4.27E+07	4.24E+07	5.24E+07	4.00E+07	1.74E+07	0.110	0.010	0.076	0.073	0.073	0.013	0.026	0.041	0.068	0.015	0.067	0.062	0.228	3	2	2	CO6A4_MOUSE, SP:A2AX52	SP|A2AX52|CO6A4_MOUSE
Q8CD54	PIEZ2_MOUSE	Piezo-type mechanosensitive ion channel component 2 OS=Mus musculus GN=Piezo2 PE=2 SV=2	1	3	1.0	9.71E+07	1.29E+08	4.86E+07	9.42E+07	1.27E+08	3.73E+08	2.35E+08	2.69E+08	1.15E+08	1.28E+08	1.31E+08	7.61E+07	1.21E+08	0.003	0.023	0.004	0.005	1.17E-02	3.27E-01	4.45E-02	0.002	0.005	2.96E-01	0.020	2.07E-02	0.025	3	3	3	PIEZ2_MOUSE, SP:Q8CD54	SP|Q8CD54|PIEZ2_MOUSE
O08523	TECTA_MOUSE	Alpha-tectorin OS=Mus musculus GN=Tecta PE=1 SV=2	1	4	0.7	3.83E+07	3.67E+07	5.10E+07	5.83E+07	5.59E+07	3.35E+07	4.32E+07	2.82E+07	2.85E+07	4.36E+07	3.94E+07	5.44E+07	3.92E+07	0.044	0.020	0.019	0.046	0.103	0.036	0.047	0.080	0.067	0.030	0.042	0.094	0.134	2	4	4	TECTA_MOUSE, SP:O08523	SP|O08523|TECTA_MOUSE
P40936	INMT_MOUSE	Indolethylamine N-methyltransferase OS=Mus musculus GN=Inmt PE=2 SV=1	2	2	1.0	1.44E+08	1.12E+08	1.09E+08	1.58E+08	1.63E+08	1.96E+08	1.93E+08	5.88E+07	9.32E+07	9.20E+07	1.14E+08	1.18E+08	1.29E+08	4.99E-01	0.002	3.10E-01	0.002	0.001	0.001	2.16E-01	0.007	0.017	0.001	0.006	0.035	0.022	2, 3	2	2	INMT_MOUSE, SP:P40936	SP|P40936|INMT_MOUSE
Q9JIL5	TULP4_MOUSE	Tubby-related protein 4 OS=Mus musculus GN=Tulp4 PE=2 SV=1	1	1	0.88	1.95E+07	2.43E+07	3.61E+07	3.72E+07	3.88E+07	3.67E+07	4.13E+07	2.48E+07	2.48E+07	4.17E+07	3.76E+07	2.46E+07	2.32E+07	0.107	0.040	0.046	0.078	0.091	0.068	0.049	0.092	0.070	0.032	0.120	0.105	0.195	2	1	1	TULP4_MOUSE, SP:Q9JIL5	SP|Q9JIL5|TULP4_MOUSE
Q3USS3	CC164_MOUSE	Coiled-coil domain-containing protein 164 OS=Mus musculus GN=Ccdc164 PE=2 SV=1	1	2	0.95	1.96E+07	6.65E+07	7.72E+06	5.52E+06	6.84E+07	5.59E+07	7.81E+06	8.49E+07	1.16E+07	7.81E+06	1.08E+08	1.31E+07	9.66E+06	0.123	0.243	0.290	0.031	0.052	0.009	0.290	0.223	0.281	0.001	0.210	0.011	0.295	3	2	2	CC164_MOUSE, SP:Q3USS3	SP|Q3USS3|CC164_MOUSE
Q6PDK8	DTX4_MOUSE	E3 ubiquitin-protein ligase DTX4 OS=Mus musculus GN=Dtx4 PE=1 SV=2	1	1	0.9	6.23E+07	4.94E+07	4.93E+07	6.28E+07	6.54E+07	5.90E+07	4.80E+07	5.10E+07	3.63E+07	4.74E+07	3.36E+07	6.50E+07	6.65E+07	0.014	0.021	0.015	0.033	0.046	0.017	0.038	0.055	0.059	0.040	0.029	0.036	0.075	3	1	1	DTX4_MOUSE, SP:Q6PDK8	SP|Q6PDK8|DTX4_MOUSE
Q9CY16	RT28_MOUSE	28S ribosomal protein S28, mitochondrial OS=Mus musculus GN=Mrps28 PE=2 SV=1	1	1	0.65	4.55E+07	2.97E+07	3.08E+07	3.93E+07	3.25E+07	3.01E+07	3.01E+07	3.67E+07	4.18E+07	4.00E+07	3.00E+07	3.03E+07	3.37E+07	0.029	0.054	0.042	0.097	0.113	0.050	0.079	0.042	0.074	0.049	0.096	0.064	0.150	2	1	1	RT28_MOUSE, SP:Q9CY16	SP|Q9CY16|RT28_MOUSE
Q8BTZ4	APC5_MOUSE	Anaphase-promoting complex subunit 5 OS=Mus musculus GN=Anapc5 PE=2 SV=1	1	11	0.99	5.30E+08	1.30E+09	6.77E+08	5.29E+08	7.95E+08	1.20E+09	7.23E+08	9.60E+08	6.78E+08	1.06E+09	8.56E+08	6.06E+08	6.82E+08	8.54E-08	4.32E-11	1.57E-07	9.75E-08	2.52E-11	1.33E-23	3.56E-09	3.48E-09	9.99E-11	1.86E-15	3.70E-06	6.53E-12	1.80E-03	5	9, 11	11, 9	APC5_MOUSE, SP:Q8BTZ4	SP|Q8BTZ4|APC5_MOUSE
O35188	X3CL1_MOUSE	Fractalkine OS=Mus musculus GN=Cx3cl1 PE=2 SV=3	1	6	0.9	1.97E+08	1.23E+08	1.07E+08	2.54E+08	1.81E+08	1.40E+08	1.38E+08	8.66E+07	1.54E+08	1.22E+08	1.13E+08	1.79E+08	1.82E+08	4.46E-02	0.001	6.38E-03	0.001	0.005	4.12E-01	0.001	4.82E-01	0.006	6.75E-01	7.74E-01	0.010	0.008	3	6	6	X3CL1_MOUSE, SP:O35188	SP|O35188|X3CL1_MOUSE
Q5SVL6	RPGP2_MOUSE	Rap1 GTPase-activating protein 2 OS=Mus musculus GN=Rap1gap2 PE=1 SV=1	1	6	0.82	2.29E+08	2.01E+08	1.43E+08	2.78E+08	2.13E+08	1.42E+08	1.95E+08	1.73E+08	1.42E+08	2.07E+08	1.84E+08	2.35E+08	2.44E+08	1.34E-02	3.11E-01	2.63E-03	5.17E-01	0.005	1.09E-02	1.94E-01	8.81E-01	5.95E-01	2.62E-02	1.74E-01	5.10E-01	0.003	4	6	6	RPGP2_MOUSE, SP:Q5SVL6	SP|Q5SVL6|RPGP2_MOUSE
Q9JK97	KCNQ4_MOUSE	Potassium voltage-gated channel subfamily KQT member 4 OS=Mus musculus GN=Kcnq4 PE=2 SV=2	1	11	0.97	1.68E+08	1.34E+08	1.61E+08	1.96E+08	2.36E+08	2.42E+08	1.69E+08	1.73E+08	1.68E+08	2.45E+08	8.18E+07	2.53E+08	4.29E+08	1.48E-01	1.20E-01	5.94E-02	2.50E-01	3.20E-01	2.45E-01	4.30E-01	2.91E-01	1.82E-01	0.003	8.89E-02	4.86E-01	1.20E-01	5	11	11	KCNQ4_MOUSE, SP:Q9JK97	SP|Q9JK97|KCNQ4_MOUSE
O70361	PER3_MOUSE	Period circadian protein homolog 3 OS=Mus musculus GN=Per3 PE=1 SV=2	1	13	0.65	2.11E+08	1.45E+08	2.37E+08	2.03E+08	4.01E+08	3.49E+08	2.82E+08	2.07E+08	4.51E+08	2.62E+08	2.38E+08	3.88E+08	2.57E+08	2.56E-02	4.51E-03	4.60E-02	3.13E-03	2.26E-02	1.53E-01	9.22E-03	1.11E-05	1.22E-01	1.92E-03	1.90E-03	1.60E-01	0.002	6	13	13	PER3_MOUSE, SP:O70361	SP|O70361|PER3_MOUSE
P70663	SPRL1_MOUSE	SPARC-like protein 1 OS=Mus musculus GN=Sparcl1 PE=1 SV=3	1	4	0.66	2.84E+08	1.92E+08	2.26E+08	2.74E+08	2.35E+08	2.09E+08	2.57E+08	2.46E+08	1.74E+08	2.06E+08	1.04E+08	3.15E+08	2.68E+08	1.39E-03	7.05E-03	2.88E-03	2.55E-01	7.42E-01	1.57E-02	2.09E-02	2.28E-01	5.32E-01	0.001	1.52E-02	4.26E-02	0.002	4	4	4	SPRL1_MOUSE, SP:P70663	SP|P70663|SPRL1_MOUSE
Q7TSC3	NEK5_MOUSE	Serine/threonine-protein kinase Nek5 OS=Mus musculus GN=Nek5 PE=2 SV=1	1	11	0.98	1.75E+08	1.43E+08	1.29E+08	1.75E+08	2.21E+08	2.58E+08	2.08E+08	2.22E+08	1.84E+08	2.67E+08	1.61E+08	2.55E+08	3.91E+08	1.17E-01	5.04E-01	1.44E-01	3.82E-01	2.17E-01	1.67E-01	1.16E-01	1.63E-01	1.03E-01	7.18E-02	8.83E-02	9.62E-02	2.34E-01	3	11	11	NEK5_MOUSE, SP:Q7TSC3	SP|Q7TSC3|NEK5_MOUSE
Q8C4S8	DEN2A_MOUSE	DENN domain-containing protein 2A OS=Mus musculus GN=Dennd2a PE=1 SV=1	1	9	0.82	3.48E+08	2.08E+08	2.02E+08	3.56E+08	3.39E+08	1.70E+08	2.40E+08	1.86E+08	2.36E+08	2.60E+08	1.72E+08	2.90E+08	3.01E+08	1.27E-04	2.12E-02	1.40E-04	1.67E-02	0.002	7.88E-03	3.92E-02	2.50E-02	1.28E-01	4.36E-02	3.31E-02	3.28E-01	0.001	4	9	9	DEN2A_MOUSE, SP:Q8C4S8	SP|Q8C4S8|DEN2A_MOUSE
Q80TL7	MON2_MOUSE	Protein MON2 homolog OS=Mus musculus GN=Mon2 PE=2 SV=2	1	1	0.99	3.82E+08	8.45E+08	5.26E+08	4.32E+08	4.89E+08	4.31E+08	5.20E+08	6.33E+08	3.78E+08	5.38E+08	3.33E+08	5.19E+08	3.31E+08	2.45E-05	1.90E-08	5.74E-06	2.68E-04	2.55E-03	2.86E-15	2.79E-06	1.24E-04	6.61E-05	2.15E-05	3.90E-05	1.62E-07	6.02E-01	7	1	1	MON2_MOUSE, SP:Q80TL7	SP|Q80TL7|MON2_MOUSE
Q8C8N3	VWC2_MOUSE	Brorin OS=Mus musculus GN=Vwc2 PE=1 SV=1	1	5	0.97	4.65E+08	3.76E+08	2.40E+08	3.58E+08	4.57E+08	9.73E+08	6.95E+08	5.83E+08	5.77E+08	5.86E+08	3.07E+08	4.16E+08	4.74E+08	1.04E-06	3.96E-03	1.05E-04	6.51E-04	5.61E-09	3.21E-06	9.51E-09	1.17E-07	1.94E-05	7.98E-05	7.79E-04	8.37E-07	5.50E-02	6	5	5	VWC2_MOUSE, SP:Q8C8N3	SP|Q8C8N3|VWC2_MOUSE
Q6NZL0	CF174_MOUSE	Uncharacterized protein C6orf174 homolog OS=Mus musculus PE=2 SV=2	1	8	0.9	2.87E+08	2.77E+08	3.25E+08	4.24E+08	3.25E+08	2.96E+08	3.63E+08	2.45E+08	2.68E+08	3.40E+08	3.73E+08	4.23E+08	4.40E+08	5.91E-03	5.23E-04	6.66E-05	4.00E-02	1.63E-01	1.65E-03	2.24E-03	1.90E-02	2.80E-02	1.69E-05	1.57E-03	1.01E-01	1.29E-01	5	8	8	CF174_MOUSE, SP:Q6NZL0	SP|Q6NZL0|CF174_MOUSE
Q8VF76	OL998_MOUSE	Olfactory receptor 998 OS=Mus musculus GN=Olfr998 PE=2 SV=2	1	7	0.8	4.54E+08	5.04E+08	5.81E+08	4.55E+08	7.44E+08	6.04E+08	6.50E+08	5.88E+08	6.36E+08	5.82E+08	5.43E+08	4.81E+08	5.42E+08	1.49E-06	1.97E-09	2.48E-06	3.80E-07	3.61E-05	9.25E-09	4.07E-08	1.38E-08	2.14E-05	1.72E-09	1.17E-04	6.97E-07	1.76E-02	6	7	7	OL998_MOUSE, SP:Q8VF76	SP|Q8VF76|OL998_MOUSE
P56475	GBRR1_MOUSE	Gamma-aminobutyric acid receptor subunit rho-1 OS=Mus musculus GN=Gabrr1 PE=2 SV=2	1	3	0.89	7.09E+08	5.55E+08	2.14E+09	5.54E+08	2.32E+08	1.85E+07	3.37E+08	1.97E+08	5.31E+08	4.76E+08	6.57E+08	1.44E+09	1.14E+09	1.01E-10	6.80E-35	8.47E-08	3.04E-01	0.186	1.43E-09	1.55E-03	7.43E-07	3.76E-04	1.77E-11	7.12E-16	2.38E-01	1.14E-06	6	3	3	GBRR1_MOUSE, SP:P56475	SP|P56475|GBRR1_MOUSE
Q8VCE4	CI040_MOUSE	Uncharacterized protein C9orf40 homolog OS=Mus musculus PE=2 SV=1	1	8	0.94	7.22E+08	7.05E+08	3.82E+08	8.41E+08	1.40E+09	5.90E+08	7.03E+08	5.07E+08	8.01E+08	8.23E+08	6.53E+08	7.03E+08	4.41E+08	3.15E-09	2.67E-03	1.97E-11	2.54E-13	2.42E-04	1.05E-09	4.07E-07	1.22E-08	3.85E-07	4.22E-09	1.90E-06	2.42E-04	1.24E-01	7	8	8	CI040_MOUSE, SP:Q8VCE4	SP|Q8VCE4|CI040_MOUSE
Q91ZB0	ALPK2_MOUSE	Alpha-protein kinase 2 OS=Mus musculus GN=Alpk2 PE=2 SV=3	1	3	0.72	8.62E+08	1.23E+09	6.82E+08	9.24E+08	3.32E+08	6.42E+08	8.76E+08	7.86E+08	1.04E+09	1.06E+09	9.06E+08	5.13E+08	3.30E+08	2.17E-13	2.96E-11	6.33E-14	1.76E-02	1.40E-05	1.79E-22	2.53E-11	1.40E-14	9.45E-11	2.19E-16	4.64E-05	1.32E-09	6.10E-01	8	3	3	ALPK2_MOUSE, SP:Q91ZB0	SP|Q91ZB0|ALPK2_MOUSE
Q8C5N3	CWC22_MOUSE	Pre-mRNA-splicing factor CWC22 homolog OS=Mus musculus GN=Cwc22 PE=1 SV=1	1	3	0.62	9.43E+08	9.43E+08	6.19E+08	1.12E+08	5.69E+08	2.92E+09	1.27E+09	1.60E+09	7.08E+08	9.95E+08	8.00E+08	2.85E+08	2.61E+08	3.49E-14	1.40E-09	0.004	8.46E-05	1.02E-26	3.01E-16	4.06E-16	3.00E-09	1.17E-09	1.15E-13	9.01E-02	1.82E-19	0.004	5	3	3	CWC22_MOUSE, SP:Q8C5N3	SP|Q8C5N3|CWC22_MOUSE
Q3UBG2	PCLI1_MOUSE	PTB-containing, cubilin and LRP1-interacting protein OS=Mus musculus GN=Pid1 PE=1 SV=2	1	1	0.62	6.85E+08	8.51E+08	9.85E+08	6.34E+08	7.50E+08	8.08E+08	8.93E+08	8.92E+08	1.03E+09	7.95E+08	8.64E+08	7.59E+08	4.74E+08	2.97E-10	3.30E-16	3.67E-09	3.89E-07	3.23E-07	4.36E-15	2.52E-11	2.95E-14	1.01E-07	2.55E-15	6.08E-08	7.74E-11	5.64E-02	6	1	1	PCLI1_MOUSE, SP:Q3UBG2	SP|Q3UBG2|PCLI1_MOUSE
Q80VW5	WHRN_MOUSE	Whirlin OS=Mus musculus GN=Whrn PE=1 SV=3	1	13	0.61	5.81E+08	3.48E+08	6.20E+08	5.78E+08	7.41E+08	8.38E+08	6.62E+08	4.85E+08	6.54E+08	4.85E+08	1.35E+09	4.91E+08	8.68E+08	2.31E-08	1.22E-09	5.40E-08	7.19E-07	2.33E-07	1.77E-05	5.59E-08	1.74E-08	3.61E-04	3.85E-22	1.17E-04	2.61E-05	1.19E-04	4	13	13	WHRN_MOUSE, SP:Q80VW5	SP|Q80VW5|WHRN_MOUSE
Q5NC57	F183B_MOUSE	Protein FAM183B OS=Mus musculus GN=Fam183b PE=2 SV=2	1	7	1.0	1.34E+09	5.21E+08	1.08E+09	1.03E+09	1.12E+09	1.07E+09	8.19E+08	5.61E+08	1.53E+09	9.34E+08	1.14E+09	1.06E+09	1.32E+09	2.76E-20	1.61E-17	4.37E-15	4.04E-11	8.63E-10	6.45E-09	2.99E-10	7.88E-21	3.42E-09	5.75E-20	2.57E-11	1.96E-06	9.99E-08	5	7	7	F183B_MOUSE, SP:Q5NC57	SP|Q5NC57|F183B_MOUSE
Q80UW5	MRCKG_MOUSE	Serine/threonine-protein kinase MRCK gamma OS=Mus musculus GN=Cdc42bpg PE=2 SV=2	1	2	0.89	1.66E+07	2.61E+07	3.16E+07	1.24E+07	0.00E+00	1.79E+07	2.54E+07	2.12E+07	2.81E+07	2.09E+07	2.99E+07	2.27E+07	1.67E+06	0.126	0.051	0.165	0.500	0.175	0.061	0.097	0.078	0.148	0.049	0.129	0.122	0.409	2	2	2	MRCKG_MOUSE, SP:Q80UW5	SP|Q80UW5|MRCKG_MOUSE
Q7TQK1	INT7_MOUSE	Integrator complex subunit 7 OS=Mus musculus GN=Ints7 PE=1 SV=1	1	5	0.99	8.54E+07	6.48E+07	9.31E+07	8.72E+07	1.28E+08	1.20E+08	8.70E+07	7.07E+07	1.20E+08	8.26E+07	1.13E+08	1.01E+08	9.85E+07	0.006	0.003	0.006	0.006	0.009	0.010	0.010	0.002	0.021	9.02E-01	0.010	0.020	0.040	5	5	5	INT7_MOUSE, SP:Q7TQK1	SP|Q7TQK1|INT7_MOUSE
Q8BHX3	BOREA_MOUSE	Borealin OS=Mus musculus GN=Cdca8 PE=2 SV=2	1	7	1.0	2.60E+08	3.25E+08	2.24E+08	1.17E+08	1.38E+08	2.25E+08	2.13E+08	6.27E+08	1.63E+08	1.93E+08	2.21E+08	1.23E+08	1.37E+08	3.41E-03	7.67E-03	0.001	0.004	5.03E-01	3.51E-05	9.19E-02	3.65E-01	7.68E-01	4.60E-03	0.004	1.96E-07	0.019	3, 4	2, 7	2, 7	BOREA_MOUSE, SP:Q8BHX3	SP|Q8BHX3|BOREA_MOUSE
P48542	IRK6_MOUSE	G protein-activated inward rectifier potassium channel 2 OS=Mus musculus GN=Kcnj6 PE=1 SV=1	1	2	0.62	1.67E+08	5.87E+07	1.20E+08	1.58E+08	1.82E+08	1.57E+08	1.33E+08	7.66E+07	1.68E+08	9.41E+07	1.79E+08	1.17E+08	1.55E+08	1.69E-01	7.78E-01	3.18E-01	0.001	0.003	0.011	0.002	2.98E-01	0.014	2.95E-02	0.005	0.014	0.013	5	2	2	IRK6_MOUSE, SP:P48542	SP|P48542|IRK6_MOUSE
Q64G17	AN32A_MOUSE	Putative acidic leucine-rich nuclear phosphoprotein 32 family member C OS=Mus musculus GN=Anp32c PE=5 SV=3	1	6	0.92	1.07E+08	9.69E+07	9.15E+07	1.43E+08	1.44E+08	1.11E+08	1.10E+08	1.05E+08	1.20E+08	1.08E+08	1.05E+08	7.71E+07	1.39E+08	0.003	0.005	8.39E-01	0.004	0.012	0.002	0.005	0.002	0.011	0.002	0.022	0.007	0.019	2	6	6	AN32A_MOUSE, SP:O35381	SP|O35381|AN32A_MOUSE,SP|Q64G17|AN32C_MOUSE
Q6P3A4	VSIG8_MOUSE	V-set and immunoglobulin domain-containing protein 8 OS=Mus musculus GN=Vsig8 PE=2 SV=2	1	2	0.7	2.37E+07	2.09E+07	2.15E+07	2.71E+07	3.48E+07	2.11E+07	2.53E+07	3.06E+07	2.16E+07	2.95E+07	1.18E+07	2.47E+07	2.22E+07	0.086	0.088	0.075	0.089	0.155	0.083	0.097	0.107	0.107	0.149	0.119	0.082	0.200	3	2	2	VSIG8_MOUSE, SP:Q6P3A4	SP|Q6P3A4|VSIG8_MOUSE
Q61234	SNTB1_MOUSE	Alpha-1-syntrophin OS=Mus musculus GN=Snta1 PE=1 SV=1	1	12	0.98	8.99E+07	9.13E+07	8.32E+07	9.65E+07	1.16E+08	9.20E+07	7.68E+07	1.03E+08	8.55E+07	1.10E+08	1.08E+08	1.13E+08	1.33E+08	0.004	0.004	0.003	0.007	0.017	0.002	0.012	0.007	0.008	8.86E-01	0.006	0.005	0.020	2	12	12	SNTB1_MOUSE, SP:Q99L88	SP|Q99L88|SNTB1_MOUSE,SP|Q61235|SNTB2_MOUSE,SP|Q61234|SNTA1_MOUSE
Q9Z1N5	DX39A_MOUSE	Spliceosome RNA helicase Ddx39b OS=Mus musculus GN=Ddx39b PE=1 SV=1	1	6	0.99	7.01E+07	1.71E+08	1.69E+08	5.47E+07	1.42E+08	1.46E+08	1.00E+08	1.39E+08	1.55E+08	1.41E+08	1.49E+08	8.57E+07	7.12E+07	0.009	8.33E-02	0.020	0.003	0.004	4.24E-02	0.005	4.72E-01	0.003	1.23E-01	0.014	0.002	0.066	3	6	6	DX39A_MOUSE, SP:Q8VDW0	SP|Q8VDW0|DX39A_MOUSE,SP|Q9Z1N5|DX39B_MOUSE
P97490	ADCY8_MOUSE	Adenylate cyclase type 8 OS=Mus musculus GN=Adcy8 PE=2 SV=1	1	7	0.86	8.09E+06	1.20E+07	1.10E+07	1.04E+07	1.14E+07	1.73E+07	1.44E+07	1.73E+07	1.07E+07	1.04E+07	1.73E+07	1.28E+07	5.95E+06	0.222	0.180	0.196	0.231	0.187	0.159	0.172	0.202	0.237	0.113	0.206	0.155	0.338	3	3	7	ADCY8_MOUSE, SP:P97490	SP|P97490|ADCY8_MOUSE
Q3ZRW6	CLUL1_MOUSE	Clusterin-like protein 1 OS=Mus musculus GN=Clul1 PE=2 SV=1	1	1	0.97	3.45E+06	7.17E+06	6.59E+07	6.16E+06	4.50E+06	9.09E+06	5.73E+06	2.91E+06	7.83E+06	5.48E+06	8.18E+06	2.15E+07	1.74E+07	0.304	0.009	0.248	0.331	0.258	0.222	0.284	0.236	0.306	0.202	0.138	0.342	0.229	3	1	1	CLUL1_MOUSE, SP:Q3ZRW6	SP|Q3ZRW6|CLUL1_MOUSE
Q80UF7	TCAM1_MOUSE	TIR domain-containing adapter molecule 1 OS=Mus musculus GN=Ticam1 PE=1 SV=1	1	5	0.67	2.46E+08	3.57E+08	1.60E+08	2.89E+08	2.51E+08	2.32E+08	2.14E+08	2.46E+08	3.23E+08	4.12E+08	2.51E+08	2.58E+08	2.08E+08	5.76E-03	1.17E-01	1.44E-03	1.59E-01	4.01E-01	6.80E-06	8.63E-02	9.19E-04	1.85E-03	9.65E-04	7.13E-02	4.06E-02	0.005	5	5	5	TCAM1_MOUSE, SP:Q80UF7	SP|Q80UF7|TCAM1_MOUSE
P70375	FA7_MOUSE	Coagulation factor VII OS=Mus musculus GN=F7 PE=1 SV=1	1	2	0.64	3.03E+08	3.48E+08	1.52E+08	2.99E+08	3.70E+08	1.89E+08	1.62E+08	2.36E+08	2.36E+08	3.48E+08	1.55E+08	2.04E+08	1.50E+08	5.73E-04	2.10E-01	9.67E-04	6.41E-03	0.001	1.04E-05	5.30E-01	2.20E-02	1.06E-02	8.97E-02	3.60E-01	7.10E-02	0.015	5	2	2	FA7_MOUSE, SP:P70375	SP|P70375|FA7_MOUSE
P43135	COT2_MOUSE	COUP transcription factor 2 OS=Mus musculus GN=Nr2f2 PE=1 SV=2	1	8	0.97	1.29E+08	1.37E+08	5.39E+07	1.60E+08	1.96E+08	1.16E+08	1.08E+08	1.27E+08	1.87E+08	1.54E+08	7.60E+07	9.72E+07	9.03E+07	7.97E-01	0.019	2.59E-01	7.71E-01	0.009	2.41E-01	0.004	1.55E-01	0.002	0.005	0.010	0.002	0.045	2	8	8	COT2_MOUSE, SP:P43135	SP|P43135|COT2_MOUSE
Q9QUS4	HEY2_MOUSE	Hairy/enhancer-of-split related with YRPW motif protein 2 OS=Mus musculus GN=Hey2 PE=1 SV=1	1	1	0.98	1.28E+07	4.15E+07	7.71E+06	1.86E+07	6.39E+06	1.84E+08	8.07E+07	5.26E+07	1.21E+07	2.12E+07	4.88E+07	2.19E+07	1.81E+07	0.157	0.209	0.116	0.282	0.001	0.026	0.010	0.176	0.146	0.017	0.133	0.034	0.224	4	1	1	HEY2_MOUSE, SP:Q9QUS4	SP|Q9QUS4|HEY2_MOUSE
Q8CH77	NAV1_MOUSE	Neuron navigator 1 OS=Mus musculus GN=Nav1 PE=1 SV=2	1	10	0.67	1.91E+08	1.76E+08	1.67E+08	1.94E+08	3.46E+08	1.87E+08	1.87E+08	1.92E+08	2.08E+08	2.14E+08	1.69E+08	2.74E+08	2.10E+08	1.21E-01	2.08E-01	1.31E-01	1.89E-02	0.002	9.03E-02	4.20E-01	1.38E-01	6.76E-01	1.75E-01	7.14E-02	4.27E-01	0.006	5	10	10	NAV1_MOUSE, SP:Q8CH77	SP|Q8CH77|NAV1_MOUSE
Q8BUE7	MUC20_MOUSE	Mucin-20 OS=Mus musculus GN=Muc20 PE=1 SV=1	1	2	0.96	8.54E+08	1.29E+08	2.04E+08	2.87E+08	9.27E+07	7.05E+07	7.69E+07	8.69E+07	8.31E+07	8.55E+07	7.12E+07	1.25E+08	7.22E+07	2.25E-12	1.88E-02	1.85E-03	0.014	0.032	3.17E-01	0.012	0.008	0.017	0.005	0.004	0.009	0.065	3	2	2	MUC20_MOUSE, SP:Q8BUE7	SP|Q8BUE7|MUC20_MOUSE
